# Supplementary material for: Conserved patterns of sequence diversification provide insight into the evolution of two-component systems in Enterobacteriaceae
Source: Microb Genom. 2024 Mar 19;10(3):001215. doi: 10.1099/mgen.0.001215 (PMC11004495; doi:10.1099/mgen.0.001215)
Supplement: Uncited Supplementary Material 1. [file mgen-10-01215-s001.pdf]

Supplemental Dataset 1: Conserved gene set sequences & alignments

PART 1: AMINO ACID SEQUENCES

Gene regulation:

1. H-NS

>Salmonella typhimurium
MSEALKILNNIRTLRAQARECTLETLEEMLEKLEVVVNERREEESAAAAEVEERTRKLQQYREMLIADGIDPNELLN
SMAAAKSGTKAKRAARPAKYSYVDENGETKTWTGQGRTPAVIKKAMEEQGKQLEDFLIKE

>Escherichia coli
MSEALKILNNIRTLRAQARECTLETLEEMLEKLEVVVNERREEESAAAAEVEERTRKLQQYREMLIADGIDPNELLN
SLAAVKSGTKAKRAQRPAKYSYVDENGETKTWTGQGRTPAVIKKAMDEQGKSLDDFLIKQ

>Citrobacter rodentium
MSEALKILNNIRTLRAQARECTLETLEEMLEKLEVVVNERREEESAAAAEVEERTRKLQQYREMLIADGIDPNELLN
SMAAVKSGTKAKRAARPAKYSYVDENGETKTWTGQGRTPAVIKKAMEEQGKQLEDFLIKE

>Klebsiella variicola
MSEALKILNNIRTLRAQARECTLETLEEMLEKLEVVVNERREEENAAAAEIEERTRKLQQYREMLIADGIDPNELLS
TMAAVKAGTKTKRAARPAKYSYVDENGETKTWTGQGRTPAVIKKAMDEQGKSLDDFLI

>Cronobacter turicensis
MSEALKILNNIRTLRAQARECTLETLEEMLEKLEVVVNERREEESAAAAEIEERTRKLQQYREMLIADGIDPNELLN
SMSAAKTGTKAKRAARPAKYRYTDENGESKTWTGQGRTPAVIKKAIDEQGKQLDDFLIKD

>Enterobacter cloacae
MSEALKILNNIRTLRAQARECTLETLEEMLEKLEVVVNERREEESAAAAEIEERTRKLQQYREMLIADGIDPNELLN
SMAAAKTGTKAKRAARPAKYSYVDENGETKTWTGQGRTPAVIKKAMDEQGKQLDDFLIKD

>Phytobacter diazotrophicus
MNEALKILNNIRTLRAQARECTLETLEEMLEKLEVVVNERREEESAAAAEIEERTRKLQQYREMLIADGIDPNELLN
SMVAAKTGTKAKRAARPAKYSYVDENGETKTWTGQGRTPAVIKKAMDEQGKQLEDFLIQE

>Kosakonia sacchari
MSEALKILNNIRTLRAQARECTLETLEEMLEKLEVVVNERREEESAAAAEVEERTRKLQQYREMLIADGIDPNELLN
SMVTAKTGTKAKRAARPAKYSYVDENGETKTWTGQGRTPAVIKKAMDEQGKQLDDFLIEE

>Huaxiibacter Chinensis
MSEALKILNNIRTLRAQARECTLETLEEMLEKLEVVVNERREEESAAAAEIEERTRKLQQYREMLIADGIDPNELLN
SMAAAKTSTKAKRAARPAKYSYVDENGETKTWTGQGRTPAVIKKAMDEQGKQLDDFLIKD

2. HupB

>Salmonella typhimurium
MNKSQLIEKIAAGADISKAAAGRALDAIIASVTESLKEGDDVALVGFGTFAVKERAARTGRNPQTGKEITIAAAKVP
SFRAGKALKDAVN

>Escherichia coli

MNKSQLIDKIAAGADISKAAAGRALDAIIASVTESLKEGDDVALVGFGTFAVKERAARTGRNPQTGKEITIAAAKVP
SFRAGKALKDAVN

>Citrobacter rodentium
MNKSQLIDKIAAGADISKAAAGRALDAIIASVTESLKEGDDVALVGFGTFSVKERAARTGRNPQTGKEITIAAAKVP
GFRAGKALKDAVN

> Klebsiella variicola
MNKSQLIDKIAAGADISKAAAGRALDALIASVTESLQAGDDVALVGFGTFAVKERAARTGRNPQTGKEITIAAAKVP
GFRAGKALKDAVN

>Cronobacter turicensis
MNKSQLIDKIAAGADISKAAAGRALDALIDSVTESLQSGDEVALVGFGTFAVRERAARTGRNPQTGKEITIAAAKVP
GFRAGKALKDAVN

>Enterobacter cloacae
MNKSQLIDKIAAGADISKAAAGRALDALIASVTESLQAGDDVALVGFGTFAVKERAARTGRNPQTGKEITIAAAKVP
GFRAGKALKDAVN

>Phytobacter diazotrophicus
MNKSQLIDKIAAGADISKAAAGRALDALIASVTESLKEGDDVALVGFGTFAVKERAARTGRNPQTGKEITIAAAKVP
GFRAGKALKDAVN

>Kosakonia sacchari
MNKSQLVDKIAADADISKAAAGRVLDAFIAAVTDSLKSGDDVALVGFGSFVVKERAARTGRNPQTGKEITIAAAKVP
GFRAGKALKDAVN

>Huaxiibacter chinensis
MNKSQLIDKIAAGADISKAAAGRALDALIASVTESLQEGDDVALVGFGTFAVKERAARTGRNPQTGKEITIAAAKVP
GFRAGKALKDAVN

3. AraC

>Salmonella typhimurium
MAETQNDPLLPGYSFNAHLVAGLTPIEANGYLDFFIDRPLGMKGYILNLTIRGEGVINNNGEQFVCRPGDILLFPPG
EIHHYGRHPDASEWYHQWVYFRPRAYWQEWLTWPTIFAQTGFFRPDEARQPHFSELFGQIISAGQGEGRYSELLAIN
LLEQLLLRRMAVINESLHPPMDSRVRDACQYISDHLADSHFDIASVAQHVCLSPSRLSHLFRQQLGISVLSWREDQR
ISQAKLLLSTTRMPIATVGRNVGFDDQLYFSRVFKKCTGASPSEFRAGCE

>Escherichia coli
MAEAQNDPLLPGYSFNAHLVAGLTPIEANGYLDFFIDRPLGMKGYILNLTIRGQGVVKNQGREFVCRPGDILLFPPG
EIHHYGRHPEAREWYHQWVYFRPRAYWHEWLNWPSIFANTGFFRPDEAHQPHFSDLFGQIINAGQGEGRYSELLAIN
LLEQLLLRRMEAINESLHPPMDNRVREACQYISDHLADSNFDIASVAQHVCLSPSRLSHLFRQQLGISVLSWREDQR
ISQAKLLLSTTRMPIATVGRNVGFDDQLYFSRVFKKCTGASPSEFRAGCEEKVNDVAVKLS

>Citrobacter rodentium
MAEPQNDPLLPGYSFNAHLVAGLTPIDANGYLDFFIDRPLGMKGYILNLTIRGEGVINNQGRQFVCRPGDILLFPPG
EIHHYGRHPEASQWYHQWVYFRPRAYWQEWLAWPSIFAQTGFFRPDEALQPHFNELFGQIISAGQGEGRYSELLAIN
LLEQLLLRRMEAINASLHPPMDSRVRDACQYISDHLADSHFDIASVAQHVCLSPSRLSHLFRQQLGISVLSWREDQR
ISQAKLLLSTTRMPIATVGRNVGFDDQLYFSRVFKKCTGASPSEFRAGCEEKVNDVSVKVS

>Klebsiella variicola
MAETQNDPLLPGYSFNAHLVTGLTPIEAQGYLDFFIDRPLGMKGYILNLTIRGEGVINNHGEQFVCRPGDMLLFPPG
EIHHYGRHPDASEWYHQWVYFRPRAYWHEWLNWPTIFAQTGFFRPDEQWQARFGELFGQIVDAGQGAGRYSELLAIN
LLEQLLLRRMEAINESLHPPLDNRVRDACQYISDHLADSHFDIASVAQHVCLSPSRLSHLFRQQLGISVLGWREDQR
ISQAKLLLSTTRMPIATVGRNVGFEDQLYFSRVFKKCTGASPSEFRAGCE

>Cronobacter turicensis
MAETQNDPLLPGYSFNAHLVAGLTPIEADGWLDFFIDRPLGMKGFILNLTVRGEGVVKNQGKEYLCRPGDMLLFPPG
EVHHYGRNPEAKEWYHQWVYFRPRAYWHEWLNWPAIFANTGFYRPDEAHHEQFSALFQAIIDAAQGPGRYAELLAIN
LLEQLLLRRVEAISESLKAPLDSRVRDACQYISDHLADSHFDIASVAQHVCLSPSRLSHLFRQQLGVSVLSWREDQR
ISQAKLLLSTTRMPIASVGRNVGFEDQLYFSRVFKKCTGASPSEFRAGCE

>Enterobacter cloacae
MAETQNDPLLPGYSFNAHLVAGLTPIEAEGYLDFYVDRPLGMKGYILNLTVRGEGIIKNNDQQFVCRPGDMLLFPPG
EIHHYGRHPDAKEWYHQWVYFRPRAYWQEWLSWPAIFAHTGFYRPDDAHLAQFRELFAQIIEAGQAGGRYAELLAIN
LLEQVLLRRMEAINASLNPPLDNRVRDACQYISDHLADSQFDIASVAQHVCLSPSRLSHLFRQQLGVSVLSWREDQR
ISQAKLLLSTTRMPIATVGRNVGFEDQLYFSRVFKKCTGASPSEFRAGCE

>Phytobacter diazotrophicus
MAESQNDPLLPGYSFNAHLVAGLTPIESEGYLDFFIDRPLGMKGYILNFTIRGEGVINNHGKQYVCRPGDMILFPPG
EIHHYGRHPDAKEWYHQWVYFRPRAYWHEWLSWPTIFAQTGFFRPEEPWLAQFSELFGQIIDAGQSGGRYAELLAIN
LLEQLLLRRMDAINESLHPPLDNRVRDACQYISDHLADNHFDIASVAQHVCLSPSRLSHLFRQQLGVSVLSWREDQR
ISQAKLLLSTTRMPIASVGRNVGFEDQLYFSRVFKKCTGASPSEFRAGCE

>Kosakonia sacchari
MAESQNDPLLPGYSFNAHLVAGLTPIEADGYLDFFIDRPLGMKGYILNLTVRGEGVVMNHGKQFVCRPGDILLFPPG
EIHHYGRHPDATEWYHQWVYFRPRAYWHEWLNWPTLFAQTGFYRPDDAHMERFRELFAQIIDAGQSVGRYAEHLAIN
LLEQLLLRRMEAINESLHPPLDNRVRDACQYISDHLADNHFDIASVAQHVCLSPSRLSHLFRQQLGVSVLSWREDQR
ISQAKLLLSTTRMPIASVGRNVGFDDQLYFSRVFKKCTGASPSEFRAGCE

>Huaxiibacter chinensis
MAETQNDPLLPGYSFNAHLVAGLTPIEAQGYLDFFVDRPLGMKGYILNITVRGEGVIKNNDKEFICRPGDILLFPPG
EIHHYGRHPEAKEWYHQWVYFRPRAYWHEWLAWPSIFAHTGFYRPDEAHQAQFRELFAQIIEAQAGGRYAELLAINL
LEQLLLRRMEAINASLNPPLDNRVRDACQYISDHLADSQFDIAGVAQHVCLSPSRLSHLFRQQLGVSVLSWREDQRI
SQAKLLLSTTRMPIASVGRNVGFEDQLYFSRVFKKCTGASPSEFRAGCE

4. SoxS

>Salmonella typhimurium
MSHQQIIQTLIEWIDEHIDQPLNIDVVAKKSGYSKWYLQRMFRTVTHQTLGEYIRQRRLLLAAVELRTTERPIFDIA
MDLGYVSQQTFSRVFRREFDRTPSDYRHRL

>Escherichia coli
MSHQKIIQDLIAWIDEHIDQPLNIDVVAKKSGYSKWYLQRMFRTVTHQTLGDYIRQRRLLLAAVELRTTERPIFDIA
MDLGYVSQQTFSRVFRRQFDRTPSDYRHRL

>Citrobacter rodentium
MSHQKIIQDLIAWIDEHIDQPLNIDVVAKKSGYSKWYLQRMFRTVTHQTLGDYIRQRRLLLAAVELRTTERPIFDIA
MDLGYVSQQTFSRVFRRQFDRTPSDYRHRL

>Klebsiella variicola
MSHQDIIQTLIEWIDEHIDQPLNIDIVARKSGYSKWYLQRMFRTVMHQTLGDYIRQRRLLLAAEALRTTQRPIFDIA
MDLGYVSQQTFSRVFRREFDRTPSDYRHQISA

>Cronobacter turicensis
MSHQEIIHALTQWIDEHIDQPLNIDVVAKKSGYSKWYLQRMFRTVMHQTLGEYIRKRRLQLAAQELRTTRRPIFDIA
MDYGYVSQQTFSRIFRRQFDRTPSDYRQSA

>Enterobacter cloacae

MSHQQIIQTLIEWIDEHIDQPLNIDVVAKKSGYSKWYLQRMFRTVMHQTLGEYIRQRRLLLAAQALRSTQRPIFDIA
MDLGYVSQQTFSRVFRREFDRTPSDYRHQLN

>Phytobacter diazotrophicus
MSHQQIIQTLTEWIDEHIDQPLNIDVVARKSGYSKWYLQRMFRTVMHQTLGDYIRQRRLLMAAEALRTTRRPIFDIA
MDLGYVSQQTFSRVFRREFDRTPSDYRHQA

>Kosakonia sacchari
MSHQQIIQTLIEWIDEHIDQPMNIDVVARKSGYSKWYLQRMFRTVMHQTLGDYIRQRRLLLAAEALRTTQRPIFDIA
MDLGYVSQQTFSRVFRREFDRTPSDYRHQA

>Huaxiibacter chinensis
MSHQQIIQTLIEWIDEHIDQPLSIDTVAKKSGYSKWYLQRMFRTVMHQTLGEYIRQRRLLLAAQALRTTKRPIFDIA
MDLGYVSQQTFSRVFRREFDRTPSDYRHQLH

5. PurR

>Salmonella typhimurium
MATIKDVAKRANVSTTTVSHVINKTRFVAEETRNAVWAAIKELHYSPSAVARSLKVNHTKSIGLLATSSEAAYFAEI
IEAVEKNCFQKGYTLILGNAWNNLEKQRAYLSMMAQKRVDGLLVMCSEYPEPLLSMLEEYRHIPMVVMDWGEAKADF
TDTVIDNAFAGGYMAGRYLVERGHRDIGVIPGPLERNTGAGRLAGFMKAMEEALINVPDNWIVQGDFEPESGYHAMQ
QILSQSHRPTAVFCGGDIMAMGALCAADEMGLRVPQDVSVIGYDNVRNARYFTPALTTIHQPKDSLGETAFNMLLDR
IVNKREESQSIEVHPRLVERRSVADGPFRDYRR

>Escherichia coli
MATIKDVAKRANVSTTTVSHVINKTRFVAEETRNAVWAAIKELHYSPSAVARSLKVNHTKSIGLLATSSEAAYFAEI
IEAVEKNCFQKGYTLILGNAWNNLEKQRAYLSMMAQKRVDGLLVMCSEYPEPLLAMLEEYRHIPMVVMDWGEAKADF
TDAVIDNAFEGGYMAGRYLIERGHREIGVIPGPLERNTGAGRLAGFMKAMEEAMIKVPESWIVQGDFEPESGYRAMQ
QILSQPHRPTAVFCGGDIMAMGALCAADEMGLRVPQDVSLIGYDNVRNARYFTPALTTIHQPKDSLGETAFNMLLDR
IVNKREEPQSIEVHPRLIERRSVADGPFRDYRR

>Citrobacter rodentium
MATIKDVAKRANVSTTTVSHVINKTRFVAEETRNAVWAAIKELHYSPSAVARSLKVNHTKSIGLLATSSEAAYFAEI
IEAVEKNCFQKGYTLILGNAWNNLEKQRAYLSMMAQKRVDGLLVMCSEYPDPLLAMLEEYRHIPMVVMDWGEAKADF
TDSVIDNAFEGGYMAGRYLVERGHREIGVIPGPLERNTGAGRLAGFMKAMEEALIKVPENWIVQGDFEPESGYRAMQ
QILSQSHRPTAVFCGGDIMAMGALCAADEMGLRVPQDVSLIGYDNVRNARFFTPALTTIHQPKDSLGETAFNMLLDR
IVNKREESQSIEVHPRLIERRSVADGPFRDYRR

>Klebsiella variicola
MATIKDVAKRANVSTTTVSHVINKTRFVAEETRNAVWAAIKELHYSPSAVARSLKVNHTKSIGLLATSSEAAYFAEI
IESVEKSCFQKGYTLILGNAWNDPEKQRAYLSMMAQKRVDGLLVMCSEYPDSVLSMLEEYRHIPMVVMDWGEAKADF
TDAVIDNAFQGGYIAGRYLIERGHREIGVIPGPLERNTGAGRLAGFMQAMKEAHISVPENWIVQGDFEPESGYRAMQ
QILSQQHRPTAVFCGGDIMAMGAICAADEMGLRVPQDISLIGYDNVRNARYFSPALTTIHQPKDSLGEAAFNMLLDR
IVNKREESQSIEVHPRLVERRSVADGPFVDYRR

>Cronobacter turicensis
MATIKDVAKRANVSTTTVSHVINKTRFVAEETRNAVWAAIKELHYSPSAVARSLKVNHTKSIGLLATSSEAPYFAEI
IEAVENSCFAKGYTLILGNAHNNLEKQRAYLSMMAQKRVDGLLVMCSEYPEPLLSTLEEYRHIPMVVMDWGEAKADF
TDSVIDNAFQGGYLAGRYLIERGHRDIGVIPGQLERNTGVGRFSGFMKALEEAAIKVPENWIVQGDFEPESGYRAMQ
QILSQPQRPTAVFCGGDIMAMGAICAADEMGLRVPQDISIIGYDNVRNARFFSPALTTIHQPKETLGQTAFNMLLDR
IVNKREESQTIEVHPRLIERRSVADGPFRDYRR

>Enterobacter cloacae
MATIKDVAKRANVSTTTVSHVINKTRFVAEETRNAVWAAIKELHYSPSAVARSLKVNHTKSIGLLATSSEAAYFAEI
IEAVEKNCFQKGYTLILGNAWNNIEKQRAYLSMMAQKRVDGLLVMCSEYPESVLSMLEEYRHIPMVVMDWGEARADF
TDSVIDNAFEGGYMAGRYLIERGHREIGVIPGPLERNTGAGRLAGFMKAMEEALITVPENWIVQGDFEPESGYRAMQ

QIVSQPHRPTAVFCGGDIMAMGALCAADELGLRVPQDISVIGYDNVRNARFFTPALTTIHQPKDSLGETAFNMLMDR
IVNKREESQSIEVHPRLIERRSVADGPFRDYRR

>Phytobacter diazotrophicus
MATIKDVAKRANVSTTTVSHVINKTRFVAEETRNAVWAAIKELHYSPSAVARSLKVNHTKSIGLLATSSEAAYFAEI
IEAVEKNCFQKGYTLILGNAWNNLEKQRAYLSMMAQKRVDGLLVMCSEYPESLLSMLEEYRHIPMVVMDWGEAKADF
TDTVIDNAFAGGYMAGRYLIERGHREIGVIPGPLERNTGAGRLSGFMKAMEEAHITVPENWIVQGDFEPESGYRAMQ
QILTQSSRPTAVFCGGDIMAMGALCAADEMGLRVPQDISVIGYDNVRNSRFFTPSLTTIHQPKDSLGETAFNMLLDR
IVNKREESQSIEVHPRLIERRSVADGPFRDYRR

>Kosakonia sacchari
MATIKDVAKRANVSTTTVSHVINKTRFVAEETRNAVWAAIKELHYSPSAVARSLKVNHTKSIGLLATSSEAAYFAEI
IEAVEKKCFQKGYTLILGNAWNSLEKQRAYLSMMAQKRVDGLLVMCSEYPESLLSMLEEYRHIPMVVMDWGEAKADF
TDSVIDNAFEGGYMAGRYLIDRGHRDIGVIPGPLERNTGAGRLAGFMKAMEEALITVPENWIVQGDFEPESGYRAMQ
QILGQAHRPTAVFCGGDIMAMGALCAADEMGLRVPQDISLIGYDNVRNSRFFTPALTTIHQPKDSLGETAFTMLLDR
IVNKREESQSIEVHPRLVERRSVADGPFRDYRR

>Huaxiibacter chinensis
MATIKDVAKRANVSTTTVSHVINKTRFVAEETRNAVWAAIKELHYSPSAVARSLKVNHTKTIGLLATSSEAAYFAEI
IEAVEQNCFDKGYTLILGNAWNSQEKQRAYLSMMAQKRVDGLLVMCSEYPESVLSMLEEYRHIPMVVMDWGDTRADF
TDSVNDNAFEGGYMAGRYLIDRGHREIGVIPGPLERNTGAGRLAGFMKAMEEAMITVPPQWIVQGDFEPESGYRAMQ
QIVSQQHRPTAVFCGGDIMAMGALCAADEMGLRVPQDISVIGYDNVRNARYFTPALTTIHQPKDSLGETAFNMLLDR
IVNKREESQSIEVHPRLIERRSVADGPFRDYRR

6. Crp

>Salmonella typhimurium
MVLGKPQTDPTLEWFLSHCHIHKYPSKSTLIHQGEKAETLYYIVKGSVAVLIKDEEGKEMILSYLNQGDFIGELGLF
EEGQERSAWVRAKTACEVAEISYKKFRQLIQVNPDILMRLSSQMARRLQVTSEKVGNLAFLDVTGRIAQTLLNLAKQ
PDAMTHPDGMQIKITRQEIGQIVGCSRETVGRILKMLEDQNLISAHGKTIVVYGTR

>Escherichia coli
MVLGKPQTDPTLEWFLSHCHIHKYPSKSTLIHQGEKAETLYYIVKGSVAVLIKDEEGKEMILSYLNQGDFIGELGLF
EEGQERSAWVRAKTACEVAEISYKKFRQLIQVNPDILMRLSAQMARRLQVTSEKVGNLAFLDVTGRIAQTLLNLAKQ
PDAMTHPDGMQIKITRQEIGQIVGCSRETVGRILKMLEDQNLISAHGKTIVVYGTR

>Citrobacter rodentium
MVLGKPQTDPTLEWFLSHCHIHKYPSKSTLIHQGEKAETLYYIVKGSVAVLIKDEEGKEMILSYLNQGDFIGELGLF
EEGQERSAWVRAKTACEVAEISYKKFRQLIQVNPDILMRLSSQMARRLQVTSEKVGNLAFLDVTGRIAQTLLNLAKQ
PDAMTHPDGMQIKITRQEIGQIVGCSRETVGRILKMLEDQNLISAHGKTIVVYGTR

>Klebsiella variicola
MVLGKPQTDPTLEWFLSHCHIHKYPSKSTLIHQGEKAETLYYIVKGSVAVLIKDEEGKEMILSYLNQGDFIGELGLF
EEGQERSAWVRAKTACEVAEISYKKFRQLIQVNPDILMRLSSQMARRLQVTSEKVGNLAFLDVTGRIAQTLLNLAKQ
PDAMTHPDGMQIKITRQEIGQIVGCSRETVGRILKMLEDQNLISAHGKTIVVYGTR

>Cronobacter turicensis
MVLGKPQTDPTLEWFLSHCHIHKYPSKSTLIHQGEKAETLYYIVKGSVAVLIKDEEGKEMILSYLNQGDFIGELGLF
EEGQERSAWVRAKTACEVAEISYKKFRQLIQVNPDILMRLSSQMARRLQVTSEKVGNLAFLDVTGRIAQTLLNLAKQ
PDAMTHPDGMQIKITRQEIGQIVGCSRETVGRILKMLEDQNLISAHGKTIVVYGTR

>Enterobacter cloacae
MVLGKPQTDPTLEWFLSHCHIHKYPSKSTLIHQGEKAETLYYIVKGSVAVLIKDEEGKEMILSYLNQGDFIGELGLF
EEGQERSAWVRAKTACEVAEISYKKFRQLIQVNPDILMRLSSQMARRLQVTSEKVGNLAFLDVTGRIAQTLLNLAKQ
PDAMTHPDGMQIKITRQEIGQIVGCSRETVGRILKMLEDQNLISAHGKTIVVYGTR

>Phytobacter diazotrophicus
MVLGKPQTDPTLEWFLSHCHIHKYPSKSTLIHQGEKAETLYYIVKGSVAVLIKDEEGKEMILSYLNQGDFIGELGLF
EEGQERSAWVRAKTACEVAEISYKKFRQLIQVNPDILMRLSSQMARRLQVTSEKVGNLAFLDVTGRIAQTLLNLAKQ
PDAMTHPDGMQIKITRQEIGQIVGCSRETVGRILKMLEDQNLISAHGKTIVVYGTR

>Kosakonia sacchari
MVLGKPQTDPTLEWFLSHCHIHKYPSKSTLIHQGEKAETLYYIVKGSVAVLIKDEEGKEMILSYLNQGDFIGELGLF
EEGQERSAWVRAKTACEVAEISYKKFRQLIQVNPDILMRLSSQMARRLQVTSEKVGNLAFLDVTGRIAQTLLNLAKQ
PDAMTHPDGMQIKITRQEIGQIVGCSRETVGRILKMLEDQNLISAHGKTIVVYGTR

>Huaxiibacter chinensis
MVLGKPQTDPTLEWFLSHCHIHKYPSKSTLIHQGEKAETLYYIVKGSVAVLIKDEEGKEMILSYLNQGDFIGELGLF
EEGQERSAWVRAKSACEVAEISYKKFRQLIQVNPDILMRLSSQMARRLQVTSEKVGNLAFLDVTGRIAQTLLNLAKQ
PDAMTHPDGMQIKITRQEIGQIVGCSRETVGRILKMLEDQNLISAHGKTIVVYGTR

7. Fnr

>Salmonella typhimurium
MIPEKRIIRRIQSGGCAIHCQDCSISQLCIPFTLNEHELDQLDNIIERKKPIQKGQTLFKAGDELKSLYAIRSGTIK
SYTITEQGDEQITGFHLAGDLVGFDAIGSGHHPSFAQALETSMVCEIPFETLDDLSGKMPNLRQQMMRLMSGEIKGD
QDMILLLSKKNAEERLAAFIYNLSRRFAQRGFSPREFRLTMTRGDIGNYLGLTVETISRLLGRFQKSGMLAVKGKYI
TIENSDALAALAGHTRNVA

>Escherichia coli
MIPEKRIIRRIQSGGCAIHCQDCSISQLCIPFTLNEHELDQLDNIIERKKPIQKGQTLFKAGDELKSLYAIRSGTIK
SYTITEQGDEQITGFHLAGDLVGFDAIGSGHHPSFAQALETSMVCEIPFETLDDLSGKMPNLRQQMMRLMSGEIKGD
QDMILLLSKKNAEERLAAFIYNLSRRFAQRGFSPREFRLTMTRGDIGNYLGLTVETISRLLGRFQKSGMLAVKGKYI
TIENNDALAQLAGHTRNVA

>Citrobacter rodentium
MIPEKRIIRRIQSGGCAIHCQDCSISQLCIPFTLNEHELDQLDNIIERKKPIQKGQTLFKAGDELKSLYAIRSGTIK
SYTITEQGDEQITGFHLAGDLVGFDAIGSGHHPSFAQALETSMVCEIPFETLDDLSGKMPNLRQQMMRLMSGEIKGD
QDMILLLSKKNAEERLAAFIYNLSRRFAQRGFSPREFRLTMTRGDIGNYLGLTVETISRLLGRFQKSGMLAVKGKYI
TIENSDALALLAGHTRNVA

>Klebsiella variicola
MIPEKRIIRRIQSGGCAIHCQDCSISQLCIPFTLNEHELDQLDNIIERKKPIQKGQTLFKAGDELKSLYAIRSGTIK
SYTITEQGDEQITGFHLAGDLVGFDAIGTGLHPSFAQALETSMVCEIPFETLDDLSGKMPNLRQQMMRLMSGEIKGD
QDMILLLSKKNAEERLAAFIYNLSRRFAQRGFSPREFRLTMTRGDIGNYLGLTVETISRLLGRFQKSGMLAVKGKYI
TIENSDLLAQLAGQARNVA

>Cronobacter turicensis
MIPEKRIIRRIQSGGCAIHCQDCSISQLCIPFTLNEHELDQLDNIIERKKPIQKGQTLFKAGDELKSLYAIRSGTIK
SYTITEQGDEQITGFHLAGDLVGFDAIGTAHHPSFAQALETSMVCEIPFETLDDLSGKMPSLRQQMMRLMSGEIKGD
QDMILLLSKKNAEERLAAFIYNLSRRFAERGFSPREFRLTMTRGDIGNYLGLTVETISRLLGRFQKSGMLAVKGKYI
TIENHEILAELAGQSRNVA

>Enterobacter cloacae
MIPEKRIIRRIQSGGCAIHCQDCSISQLCIPFTLNEHELDQLDNIIERKKPIQKGQTLFKAGDELKSLYAIRSGTIK
SYTITEQGDEQITGFHLAGDLVGFDAIGTGHHPSFAQALETSMVCEIPFETLDDLSGKMPNLRQQMMRLMSGEIKGD
QDMILLLSKKNAEERLAAFIYNLSRRFAERGFSPREFRLTMTRGDIGNYLGLTVETISRLLGRFQKSGMLAVKGKYI
TIENGEALAVLAGHARNVA

>Phytobacter diazotrophicus
MIPEKRIIRRIQSGGCAIHCQDCSISQLCIPFTLNEHELDQLDNIIERKKPIQKGQTLFKAGDELKSLYAIRSGTIK
SYTITEQGDEQITGFHLAGDLVGFDAIGTGHHPSFAQALETSMVCEIPFETLDDLSGKMPNLRQQMMRLMSGEIKGD

QDMILLLSKKNAEERLAAFIYNLSRRFAQRGFSPREFRLTMTRGDIGNYLGLTVETISRLLGRFQKSGMLAVKGKYI
TIENSDILAQLAGHSRNVA

>Kosakonia sacchari
MIPEKRIIRRIQSGGCAIHCQDCSISQLCIPFTLNEHELDQLDNIIERKKPIQKGQTLFKAGDELKSLYAIRSGTIK
SYTITEQGDEQITGFHLAGDLVGFDAIGTGHHPSFAQALETSMVCEIPFETLDDLSGKMPNLRQQMMRLMSGEIKGD
QDMILLLSKKNAEERLAAFIYNLSRRFAQRGFSPREFRLTMTRGDIGNYLGLTVETISRLLGRFQKSGMLAVKGKYI
TIENSDMLAQLAGHARNVA

>Huaxiibacter chinensis
MIPEKRIIRRIQSGGCAIHCQDCSISQLCIPFTLNEHELDQLDNIIERKKPIQKGQTLFKAGDELKSLYAIRSGTIK
SYTITEQGDEQITGFHLAGDLVGFDAIGTGHHPSFAQALETSMVCEIPFETLDDLSGKMPNLRQQMMRLMSGEIKGD
QDMILLLSKKNAEERLAAFIYNLSRRFAERGFSPREFRLTMTRGDIGNYLGLTVETISRLLGRFQKSGMLAVKGKYI
TIENGEALAVLAGHARNVA

8. FadR

>Salmonella typhimurium
MVIKAQSPAGFAEEYIIESIWNNRFPPGTILPAERELSELIGVTRTTLREVLQRLARDGWLTIQHGKPTKVNNFWET
SGLNILETLARLDHESVPQLIDNLLSVRTNISTIFIRTALRQHPDKAQEVLATAHEVADHADAFADLDYNIFRGLAF
ASGNPIYGLILNGMKGLYTRIGRHYFANPEARSLALGFYHKLSSLCEQGAHDQVYETVRRYGHDSGEIWHRMQKNLP
GDLAIQGR

>Escherichia coli
MVIKAQSPAGFAEEYIIESIWNNRFPPGTILPAERELSELIGVTRTTLREVLQRLARDGWLTIQHGKPTKVNNFWET
SGLNILETLARLDHESVPQLIDNLLSVRTNISTIFIRTAFRQHPDKAQEVLATANEVADHADAFAELDYNIFRGLAF
ASGNPIYGLILNGMKGLYTRIGRHYFANPEARSLALGFYHKLSALCSEGAHDQVYETVRRYGHESGEIWHRMQKNLP
GDLAIQGR

>Citrobacter rodentium
MVIKAQSPAGFAEEYIIESIWNNRFPPGTILPAERELSELIGVTRTTLREVLQRLARDGWLTIQHGKPTKVNNFWET
SGLNILETLARLDHESVPQLIDNLLSVRTNISTIFIRTAFRQHPDRAQEVLATAQEVADHADAFAELDYNIFRGLAF
ASGNPIYGLILNGMKGLYTRIGRHYFANPEARSLALGFYHKLSQLCLQGAHDQVYETVRRYGHDSGEIWHRMQKNLP
GDLAIQGR

>Klebsiella variicola
MVIKAQSPAGFAEEYIIESIWNNRFPPGSILPAERELSELIGVTRTTLREVLQRLARDGWLTIQHGKPTKVNNFWET
SGLNILETLARLDHDSVPQLIDNLLSVRTNISTIFIRTAFRQHPDKALAVLDSAREVEDHADAFADLDYNIFRGLAF
ASGNPIYGLILNGMKGLYTRIGRHYFSSPEARSLALGFYHQLAKVCEGGLHDQVYELVRRYGHDSGEIWHRMQKSLP
GDLAMNMR

>Cronobacter turicensis
MVIKAQSPAGFAEEYIVESIWNNRFPPGSILPAERELSELIGVTRTTLREVLQRLARDGWLTIQHGKPTKVNNFWET
SGLNILETLARLDHESVPQLIDNLLSVRTNIATIFIRTALRMHPERAREVLATADEVEDHADAFAELDYNIFRGLAF
ASGNPIYGLILNGMKGLYTRIGRHYFSNPEARSLALGFYHKLGTLSREGQHDQVYDVVRTYGRESGEIWHRMQKNLP
GDLAMHSR

>Enterobacter cloacae
MVIKAQSPAGFAEEYIIESIWNNRFPAGSILPAERELSELIGVTRTTLREVLQRLARDGWLTIQHGKPTKVNNFWET
SGLNILETLARLDHESVPQLIDNLLSVRTNIATIFIRTAFRQHPEDALQVLASANEVEDHADAFATLDYNVFRGLAF
ASGNPVYGLILNGMKGLYTRIGRHYFANPEARSLALGFYHKLSKLCTEGLHDQVYETVRRYGHDSGEIWHRMQKTLP
GDLAIQGR

>Phytobacter diazotrophicus
MVIKAQSPAGFAEEYIIESIWNNRFPPGSILPAERELSELIGVTRTTLREVLQRLARDGWLTIQHGKPTKVNNFWET
SGLNILETLARLDHESVPQLIDNLLSVRTNIATIFIRTAFRQHPEDALAVLASAREVEDHGDAFAELDYNIFRGLAF

ASGNPIYGLILNGMKGLYTRIGRHYFASPEARSLALGFYHRLAEICEQGLHDQVYETVRRYGHDSGEIWQRMHKTLP
GDLSINGR

>Kosakonia sacchari
MVIKAQSPAGFAEEYIIESIWNSRFPPGSILPAERELSELIGVTRTTLREVLQRLARDGWLTIQHGKPTKVNNFWET
SGLNILETLARLDHESVPQLIDNLLSVRTNISTIFIRTAFRQHPEDALKVLASAREVEDHADAFADLDYNIFRGLAF
ASGNPIYGLILNGMKGLYTRIGRHYFSSPEARSLALGFYHQLAQVCEEAQHDKVYEVVRRYGHDSGEIWHRMQKTMP
GDLALQSR

>Huaxiibacter chinensis
MVIKAQSPAGFAEEYIIESIWNNRFAPGTILPAERELSELIGVTRTTLREVLQRLARDGWLTIQHGKPTKVNNFWET
SGLNILETLARLDHESVPQLIDNLLSVRTNISTIFIRTAFRQHPDKALEVLATANAVEDHADAFASLDYNIFRGLAF
ASGNPIYGLIINGMKGLYTRIGRHYFANPEARSLALGFYHKLAELCTEGAHDQVYETVRRYGHDSGEIWHRMQKTLP
GDLAIHGH

9. GntR

>Salmonella typhimurium
MKKKRPVLQDVADRVGVTKMTVSRFLRNPEQVSVALRGKIAAALDELGYIPNRAPDILSNATSRAIGVLLPSLTNQV
FAEVLRGIEAVTDAHGYQTMLAHYGYKPEMEQERLESMLSWNIDGLILTERTHTPRTLKMIEVAGIPVVELMDSQSP
CLDIAVGFDNFEAARQMTAAIIARGHRHIAYLGARLDERTIIKQKGYEQAMRDAGLVPYSVMMEQSSSYSSGIELMR
QARREYPQLDGIFCTNDDLAVGAAFECQRLGLKIPDDMAIAGFHGHDIGQVMEPRLASVLTPRERMGSIGAERLLAR
IRGETVTPKMLDLGFTLSPGGSI

>Escherichia coli
MKKKRPVLQDVADRVGVTKMTVSRFLRNPEQVSVALRGKIAAALDELGYIPNRAPDILSNATSRAIGVLLPSLTNQV
FAEVLRGIESVTDAHGYQTMLAHYGYKPEMEQERLESMLSWNIDGLILTERTHTPRTLKMIEVAGIPVVELMDSKSP
CLDIAVGFDNFEAARQMTTAIIARGHRHIAYLGARLDERTIIKQKGYEQAMLDAGLVPYSVMVEQSSSYSSGIELIR
QARREYPQLDGVFCTNDDLAVGAAFECQRLGLKVPDDMAIAGFHGHDIGQVMEPRLASVLTPRERMGSIGAERLLAR
IRGESVTPKMLDLGFTLSPGGSI

>Citrobacter rodentium
MKKKRPVLQDVADRVGVTKMTVSRFLRNPEQVSVALRGKIAAALDELGYIPNRAPDILSNATSRAIGVLLPSLTNQV
FAEVLRGIEAVTDAHGYQTMLAHYGYKPEMEQERLESMLSWNIDGLILTERTHTPRTLKMIEVAGIPVVELMDSQSP
CLDIAVGFDNFEAARQMTAAIIARGHRHIAYLGARLDERTIIKQKGYEQAMLDAGLVPYSVMVEQSSSYSSGIELIR
QARREYPQLDGIFCTNDDLAVGAAFECQRLGLSIPRDMAIAGFHGHDIGQVMEPRLASVLTPRERMGSIGAERLLAR
IRGESVTPKMLDLGFTLSPGGSI

>Klebsiella variicola
MKKKRPVLQDVADLVGVTKMTVSRYLRNPEQVSEALRGKIAVALDELGYIPNRAPDILSNATSRAIGVLLPSLTNQV
FSEVLRGIESVTDAFGYQTMLAHYGYKPEMEEKRLESMLSWNIDGLILTERTHTPRTLKMIEVAGIPVVELMDSRSP
CLDIAVGFDNFEAARQMTAAIIARGHRHVAYLGARLDERTIIKQKGYEQAMLDAGMTPYSVMVEQSSSYSSGIELMR
QARREYPQLDGIFCTNDDLAVGAAFECQRLGLKIPDDMAIAGFHGHDIGQVMEPRLASVLTPRERMGRIGAERLLAR
IRGEAITPKMLDLGFTLSPGGSI

>Cronobacter turicensis
MKKRRPVLQDVADRVGVTKMTISRYLRNPEQVSLALRSKIAAALDELGYIPNRAPDILSNATSRAIGVLLPSLTNQV
FAEVLRGIESVIDAHGYQTMLAHYGYKPELEEERLESMLSWNIDGLILTERNHTPRTLKMIEVAGIPVVELMDSVSP
CLDIAVGFDNFDAARQMTAAIIARGHRHVAYLGARLDERTIMKQKGYEQAMLDAGLTPYSVMVEHSSSFSTGSELLR
QARREYPQLDSIFCTNDDLAIGAAFECQRLGLRIPDDMAIAGFHGHDIGQVMEPQLASVLTPRERMGRIGAERLLAR
IRGETVTPQMLDLGFTLSPGGSI

>Enterobacter cloacae
MKKKRPVLQDVADRVGVTKMTVSRFLRNPEQVSVALRGKIAAALDELGYIPNRAPDILSNATSRAVGVLLPSLTNQV
FAEVLRGIESVTDAFGYQTMLAHYGYKPELEEERLESMLSWNIDGLILTERTHTPRTLKMIEVAGIPVVELMDSQSP
CLDIAVGFDNFEAARQMTAAIIARGHRHIAYLGARLDERTIIKQKGYEQAMLDANLTPYSVMVEQSSSYTSGIELMR

QARREYPQLDGIFCTNDDLAVGAAFECQRLGLKIPDDMAIAGFHGHDIGQVMEPRLASVLTPRERMGRIGAERLLAR
IRGEAVTPKMLDLGFTLSPGGSI

>Phytobacter diazotrophicus
MKKKRPVLQDVADRVGVTKMTVSRFLRNPEQVSVALRSKIAAALDELGYIPNRAPDILSNATSRAVGVLLPSLTNQV
FAEVLRGIESVTDAFGYQTMLAHFGYKPELEEERLESMLSWNIDGLILTERTHTPRTLKMIEVAGIPVVELMDSKSP
CLDIAVGFDNFEAARQMTAAIIERGHRRVAYLGARLDERTIIKQKGYEQAMLDAGLTPYSVMVEQSSSYTAGIELMR
QARREYSQLDGIFCTNDDLAVGAAFECQRLGLKVPDDMAIAGFHGHDIGQVMEPRLASVLTPRERMGRIGAERLLAR
IRGESVTPKLLDLGFTLSPGGSI

>Kosakonia sacchari
MKKKRPVLQDVADRVGVTKMTVSRFLRNPEQVSVALRGKIAAALDELGYIPNRAPDILSNATSRAIGVLLPSLTNQV
FAEVLRGIESVTDAHGYQTMLAHYGYKAEMEEERLESMLSWNIDGLILTERTHTPRTLKMIEVAGIPVVELMDSRSP
CLDIAVGFDNFEAARQITTAIIARGHRRVAYLGARLDERTIIKQKGYEQAMLDAGLTPYSVMVEQSSSYTAGIELMR
QARREYPQLDGIFCTNDDLAVGAAFECQRLGLKIPQDMAIAGFHGHDIGQVMEPRLASVLTPRERMGRIGAERLLAR
IRGESVTPKMLDLGFTLSPGGSI

>Huaxiibacter chinensis
MKKKRPVLQDVADRVGVTKMTVSRFLRNPEQVSLALRGKIAAALDELGYIPNRAPDILSNATSRAVGVLLPSLTNQV
FAEVLRGIESVTDAFGYQTMLAHYGYKPELEEERLESMLSWNIDGLILTERTHTPRTLKMIEVAGIPVVELMDSQSP
CLDIAVGFDNYEAARQMTAAILARGHRHVAYLGARLDERTIIKQKGYEQAMLDANLTPYSVMVEQSSSYSSGIELMR
QARREYPQLDGIFCTNDDLAVGAAFECQRLGLKIPDDIAIAGFHGHDIGQVMEPRLASVLTPRERMGRIGAERLLAR
IRGEVVTPKMLDLGFTLSPGGSI

10. DeoR

>Salmonella typhimurium
METRRDERIGQLLQALKRSDKLHLKEAATLLGVSEMTIRRDLNHKSAPVVLLGGYIVLEPRSASHYLLSDQKSRLVE
EKRRAAQLAAGLVQAHQTVFIDCGTTTPWIIEAIDNDLPFTAVCYSLNTFLALQDKPHCRAILSGGEFHASNAIFKP
LDFHETLNNICPDIAFYSAAGVHTSKGATCFNLEELPVKHWAMTMAQCHVLVVDHSKFGKVRPARMGELSRFDTIIS
DRRPDEAFVAYAKAQQITLMY

>Escherichia coli
METRREERIGQLLQELKRSDKLHLKDAAALLGVSEMTIRRDLNNHSAPVVLLGGYIVLEPRSASHYLLSDQKSRLVE
EKRRAAKLAATLVEPDQTLFFDCGTTTPWIIEAIDNEIPFTAVCYSLNTFLALKEKPHCRAFLCGGEFHASNAIFKP
IDFQQTLNNFCPDIAFYSAAGVHVSKGATCFNLEELPVKHWAMSMAQKHVLVVDHSKFGKVRPARMGDLKRFDIVVS
DCCPEDEYVKYAQTQRIKLMY

>Citrobacter rodentium
METRRDERIGQLLQALKRSDKLHLKEAAALLGVSEMTIRRDLNSHSAPVVLLGGYIVLEPRGASHYLLSDQKSRLVE
EKRRAAQLAATLVQPHQTLFFDCGTTTPWIIEAIDNDMPFTAVCYSLNTFLALQEKPLCRAILSGGEFHASNAIFKP
LDTQETLSHICPDIAFYSAAGIHLSKGATCFNLEELPVKHWAMARAQYHALVADHSKFGKVRPARMGDLTRFDAVIS
DRCPDDEFVDFAKAQDIKLMY

>Klebsiella variicola
METRRDERISQLIQALKRSDKLHLKEAASLLGVSEMTIRRDLNGHSGPVVLLGGYIVLEPRSATHYLLSDQKTRLVE
EKRRAARHAAALLEAHQMAFFDCGTTTPWIIDAIDDALPFTGVCYSLNTFLALQEKPQCRAVLCGGEFHASNAIFMP
LSLEDTLSHLSPDIAFYSAAGIDCEQGATCYNLEELPVKHWAMRHARYHVLVVDHSKFGKVRPARMGALAKFDVIAS
DICPDDELVALAKAQQISLLY

>Cronobacter turicensis
METRRDERLAQLLQALKRQDKMHLKEAAALLGVSEMTIRRDLQGNDAPVTLLGGYIVLEPRGVAVSRYLLSDEQTRL
VEEKRHAAALAASLARPHQTLFFDCGTTTPWIIDALDDALPFTGICYSLNTFLALQEKPHCRVILCGGEFHASNAIF
KPLNFQETLRNLCPDIAFFSAAGVHPQYGATCFNLDELPVKHWALEMAQRHVIVADHSKFGQVRPACMGPLEAFDTI
ATDRMPGDAFIAWAQAANVSVMW

>Enterobacter cloacae

METRRDDRIAQLIQALKRSDKLHLKEAATLLGVSEMTIRRDLNSESAPVVLLGGYIVLEPRSASHYLISDQKTRLVE
EKRKAARLAASLVQPHQTLFFDCGTTTPWIIEAIDSSIPFTAVCYSLNTFLALQEKPECRVILCGGEFHASNAIFKP
LNLQDTLSNLCPDIAFYSAAGVSTRQGATCFNLEELPVKHWALSAAQYHVLVVDHSKFGKVRPARMGELAQFNAIVS
DCRPDEEYVTYAKAQQVKLMY

>Phytobacter diazotrophicus
METRRDERISQLLSALKRSDKLHLKEAAALLGVSEMTIRRDLSANSAPVVLLGGYIVLEPRTASHYLLSDQKTRLVD
EKRRAAELAAQLVQPHQMVFFDCGTTTPWIIEAIPADLPFTGVCYSLNTFLALQEKPLCRAILSGGEFHASNAIFQP
LNFKETLSYLRPDMAFYSAAGVHIEQGVTCFNLEELPLKHWAMASAQFHVLVVDHSKFGKVRPACMGDLSHFDAIVS
DCRPDEALVEFAKAEQIKLVF

>Kosakonia sacchari
METRRDERIGQLLAALKRSDKLHLKEAATLLGVSEMTIRRDLSANSAPVVLLGGYIVLEPRSASHYLLSDQKNRLVE
EKRHAAKLAAGLLKAHQMAFFDCGTTTPWIIEAIADDLPFTGVCYSLNTFLALQEKPQCRAILSGGEFHASNAIFKP
LNFNETLNNLRPDIAFYSAAGVHAEQGATCFNLEELPVKHWAMACAQYHVLVVDHSKFGKVRSACMGPLSRFDVLVS
DCRPDEALENEAKKHQLTLLY

>Huaxiibacter chinensis
METRRDDRIAQLLQALKRSDKLHLKEAAALLGVSEMTIRRDLNSEPGPVVLLGGYIVLEPRSASHYLINDQKTRLVE
EKRKAARLAASLVQPHQTLFFDCGTTTPWIIEAIDNALPFTAVCYSLNTFLALQEKPECRVILCGGEFHASNAIFKP
LDLQDTLTNLCPDIAFYSAAGVSVRQGATCFNLEELTVKHWAMNAAQYHVLVVDHSKFGKVRSARMGELARFDAIVS
DCRPDEELVAHAKAQQIKLMY

11. TreR

>Salmonella typhimurium
MQNRLTIKDIARLSGVGKSTVSRVLNNESGVSERTRERVEAVMNQHGFSPSRSARAMRGQSDKVVAIIVTRLDSLSE
NLAVQTMLPAFYEQGYDPIMMESQFSPTLVMEHLGMLRRRNIDGVVLFGFTGITEELIAPWKASLVLLARDAQGFAS
VCYDDEGAIHILMQRLYEQGHRNISFLGVPHSDITTGKRRHDAYLAFCKKHKLHPVAALPGLAMKQGYEHTASVIMP
DTTALVCATDTLALGASKYLQEQRIETLQLASVGNTPLIKFLHPEIVTVDPGYAEAGRQAASQLIEQINGRCDPRRI
VIPSTLA

>Escherichia coli
MQNRLTIKDIARLSGVGKSTVSRVLNNESGVSQLTRERVEAVMNQHGFSPSRSARAMRGQSDKVVAIIVTRLDSLSE
NLAVQTMLPAFYEQGYDPIMMESQFSPQLVAEHLGVLKRRNIDGVVLFGFTGITEEMLAHWQSSLVLLARDAKGFAS
VCYDDEGAIKILMQRLYDQGHRNISYLGVPHSDVTTGKRRHEAYLAFCKAHKLHPVAALPGLAMKQGYENVAKVITP
ETTALLCATDTLALGASKYLQEQRIDTLQLASVGNTPLMKFLHPEIVTVDPGYAEAGRQAACQLIAQVTGRSEPQQI
IIPATLS

>Citrobacter rodentium
MQNRLTIKDIARLSGVGKSTVSRVLNNESGVSERTRERVEAVMNQHGFSPSRSARAMRGQSDKVVAIIVTRLDSLSE
NLAVQTMLPVFYEQGYDPIMMESQFSAEKVDEHLGMLRRRNIDGVVLFGFTGIPDAMLAPWQSSLVLLARDAKGFAS
VCYDDEGAINMLMQRLYERGHRHISFLGVPHSDVTTGKRRHEAYLAFCRQHKLHPVAALPGLAMKQGYDYAASVMTP
ETSALLCATDTLALGASKYLQERRIDTLQLASVGNTPLMKFLHPEIITVDPGYAEAGRQAACQLIEQINDRCDLRQI
VIPSTLS

>Klebsiella variicola
MQNRLTIKDIARLSGVGKSTVSRVLNNESGVSERTRERVEAVMQQHGFSPSRSARAMRGQSDKVVAIIVTRLDSLSE
NLAVQTMLPAFYEQGYDPIMMESQFSPGLVEEHLGMLARRNIDGVVLFGFTGIDEAMLAPWRDTLVLMARDAPGFAS
VCYDDEGAITLLMQRLYDRGHRHISFLGVPHSDVTTGERRHLAYLAFCKKHRLTPTAALPGLGMKQGYDTVASVLTA
ETSALVCATDTLALGASKYLQQQGRAALQLASVGSTPLMKFLHPEILTVDPGYAESGRRAARQLIEQIAGSVEPRQI
VIPAALN

>Cronobacter turicensis
MQHRLTIKDIARLSGVGKSTVSRVLNNESGVSERTREKVEAVMRQHGFSPSRSARAMRGQSDKVVAIIVTRLDSLSE
NLAVQTILPALYEQGYDPIMLESRFSPALVQEHLGVLSRRHIDGVILFGFSGISEVLTTAWQESLVLLARDAPGFAS
VCYEDDGAIRLLMQRLYDHGHRHISFLGVPHSDITTGKRRHESYLNFCHAHGLTPCDTLPGLAMKQGYEHAADVLNE

ETTALVCATDTLAIGASKYLQQRGGAPVQLASVGNTPLLKFLHPEAIAVDPGYGEAGRAAAKQLIDQITGRAAPRQI
VIPARLD

>Enterobacter cloacae
MQNRLTIKDIARLSGVGKSTVSRVLNNESGVSERTRERVEAVMNQHGFSPSRSARAMRGQSDKVVAIIVSRLDSLSE
NLAVQTMLPAFYEQGYDPIMMESQFSPQLVEEHLGMLARRNIDGVVLFGFTGIKEEMLKPWQPSLVLLARDAHGFAS
VCYDDEGAIITLMQRLYEKGHRHISYLGVPHADVTTGKRRHEAYLAFCKKHNLSAVASLPGLGMKQGYEQVASVLTP
QTTALVCATDTLALGASKYLQEQRIDDLQVASVGSTPLMKFLHPEIITVDPGYAESGRQAAAQLIEQINGRAEPRQI
VIPVHLS

>Phytobacter diazotrophicus
MQNRLTIKDIARLSGVGKSTVSRVLNNESGVSERTRERVEAVVNQHGFSPSRSARAMRGQSDKVVAIIVSRLDSLSE
NLAVQTMLPTFYEQGYDPIMMESQFSPDLVEEHLHMLRRRNIDGVVLFGFTGVTEKLLSSWRASLVLLARDAKGFAS
VCYDDEGAINLLMQNLYDQGHRHISFIGVPHSDITTGKRRHEAYLAFCERHQLTPNFILPGLAMKHGYDNVASVLTP
DTSALLCATDTLALGASKYLQEQRIDNLQLASVGNTPLMKFLHPEIVTVDPGYAEAGRQAAAQLIDQVNGRTDPRQI
VIPAHLA

>Kosakonia sacchari
MQNRLTIKDIARLSGVGKSTVSRVLNNESGVSQRTRERVEAVVNQHGFSPSRSARAMRGQSDKVVAIIVSRLDSLSE
NLAVQTMLPAFYEQGYDPIMMESQFSPQLVEEHLQMLSHRNIDGVVLFGFTGVTESLLTAWQSSLVLLARDASGFAS
VCYDDDGAIQLLMENLYARGHRHISFIGVPHSDATTGKRRHDAYMAFCQKHQLEPTFALPGLAMKQGYEHVANVLTP
HTTALLCATDTLALGASKYLQEQRIDNLQLASVGNTPLMKFLHPEIVTVDPGYAEAGRQAAAQLIEQVNGRATPRQI
VIPARLV

>Huaxiibacter chinensis
MQNRLTIKDIARLSGVGKSTVSRVLNNESGVSERTRERVEAVMAQHGFSPSRSARAMRGQSDKVVAIIVSRLDSLSE
NLAVQTMLPAFYEQGYDPIMMESQFSTQMVEEHLGMLQRRNIDGVVLFGFSGIQDEMLKPWQASLVLMAREASGFAS
VCYDDEGAITLLLHRLYELGHRHISYLGVPHNDVTTGKRRHEAYLTFCKQHNLPAVASLPGLAMKQGYDKVASVLTT
QTTALVCATDTLALGASKYLQEQRIDSLQLASVGSTPLMKFLHPEIVTVNPGYGEAGRQAASQLIEQINGRSEPRQI
VIPSHLS

12. IhfB

>Salmonella typhimurium
MTKSELIERLATQQSHIPAKAVEDAVKEMLEHMASTLAQGERIEIRGFGSFSLHYRAPRTGRNPKTGDKVELEGKYV
PHFKPGKELRDRANIYG

>Escherichia coli
MTKSELIERLATQQSHIPAKTVEDAVKEMLEHMASTLAQGERIEIRGFGSFSLHYRAPRTGRNPKTGDKVELEGKYV
PHFKPGKELRDRANIYG

>Citrobacter rodentium
MTKSELIERLATQQSHIPAKAVEDAVKEMLEHMASTLAQGERIEIRGFGSFSLHYRAPRTGRNPKTGDKVELEGKYV
PHFKPGKELRDRANIYG

>Klebsiella variicola
MTKSELIERLASQQSHIPAKAVEDAVKEMLEHMASTLAQGERIEIRGFGSFSLHYRAPRTGRNPKTGDKVELEGKYV
PHFKPGKELRDRANIYEE

>Cronobacter turicensis
MTKSELIERLASQHSHIPAKAVEDAVKEMLEHMASTLAQGERIEIRGFGSFSLHYRAPRTGRNPKTGDKVELEGKYV
PHFKPGKELRDRANIYG

>Enterobacter cloacae
MTKSELIERLASQQPHIPAKAVEDAVKEMLEHMATTLAQGERIEIRGFGSFSLHYRAPRTGRNPKTGDKVELEGKYV
PHFKPGKELRDRANIYGN

>Phytobacter diazotrophicus
MTKSELIERLASQQSHIPAKAVEDAVKEMLEHMASTLAQGERIEIRGFGSFSLHYRAPRTGRNPKTGDKVDLEGKYV
PHFKPGKELRDRANIYEG

>Kosakonia sacchari
MTKSELIERLASQQSHIPAKAVEDAVKEMLEHMASTLAQGERIEIRGFGSFSLHYRAPRTGRNPKTGDKVDLEGKYV
PHFKPGKELRDRANIYE

>Huaxiibacter chinensis
MTKSELIERLASQQSHIPAKAVEDAVKEMLEHMASTLAQGERIEIRGFGSFSLHYRAPRTGRNPKTGDKVDLEGKYV
PHFKPGKELRDRANIYDN

13. RpoS

>Salmonella typhimurium
MSQNTLKVHDLNEDAEFDENGVEAFDEKALSEEEPSDNDLAEEELLSQGATQRVLDATQLYLGEIGYSPLLTAEEEV
YFARRALRGDVASRRRMIESNLRLVVKIARRYGNRGLALLDLIEEGNLGLIRAVEKFDPERGFRFSTYATWWIRQTI
ERAIMNQTRTIRLPIHIVKELNVYLRTARELSHKLDHEPSAEEIAEQLDKPVDDVSRMLRLNERITSVDTPLGGDSE
KALLDILADEKENGPEDTTQDDDMKQSIVKWLFELNAKQREVLARRFGLLGYEAATLEDVGREIGLTRERVRQIQVE
GLRRLREILQTQGLNIEALFRE

>Escherichia coli
MSQNTLKVHDLNEDAEFDENGVEVFDEKALVEQEPSDNDLAEEELLSQGATQRVLDATQLYLGEIGYSPLLTAEEEV
YFARRALRGDVASRRRMIESNLRLVVKIARRYGNRGLALLDLIEEGNLGLIRAVEKFDPERGFRFSTYATWWIRQTI
ERAIMNQTRTIRLPIHIVKELNVYLRTARELSHKLDHEPSAEEIAEQLDKPVDDVSRMLRLNERITSVDTPLGGDSE
KALLDILADEKENGPEDTTQDDDMKQSIVKWLFELNAKQREVLARRFGLLGYEAATLEDVGREIGLTRERVRQIQVE
GLRRLREILQTQGLNIEALFRE

>Citrobacter rodentium
MSQNTLKVHDLNEDAEFDENGVEVFDEKALVEEEPSDNDLAEEELLSQGATQRVLDATQLYLGEIGYSPLLTAEEEV
YFARRALRGDVASRRRMIESNLRLVVKIARRYGNRGLALLDLIEEGNLGLIRAVEKFDPERGFRFSTYATWWIRQTI
ERAIMNQTRTIRLPIHIVKELNVYLRTARELSHKLDHEPSAEEIAEQLDKPVDDVSRMLRLNERITSVDTPLGGDSE
KALLDILADEKDNGPEDTTQDDDMKQSIVKWLFELNAKQREVLARRFGLLGYEAATLEDVGREIGLTRERVRQIQVE
GLRRLREILQTQGLNIEALFRE

>Klebsiella variicola
MSQNTLKVHDLNEDAEFDENGIEVFDEKALVEEEPSDSDLAEEELLSQGATQRVLDATQLYLGEIGYSPLLTAEEEV
YFARRALRGDVASRRRMIESNLRLVVKIARRYSNRGLALLDLIEEGNLGLIRAVEKFDPERGFRFSTYATWWIRQTI
ERAIMNQTRTIRLPIHIVKELNVYLRTARELSHKLDHEPSAEEIAEQLDKPVDDVSRMLRLNERITSVDTPLGGDSE
KALLDILADEKENGPEDTTQDDDMKQSIVKWLFELNAKQREVLARRFGLLGYEAATLEDVGREIGLTRERVRQIQVE
GLRRLREILQGQGLNIEALFRE

>Cronobacter turicensis
MNQNTLKVHDLNEDAEFDENGVEAFDEKALVEEEPSDNDLAEEELLSQGSTQRVLDATQLYLGEIGYSPLLTAEEEV
YFARRALRGDVASRRRMIESNLRLVVKIARRYSNRGLALLDLIEEGNLGLIRAVEKFDPERGFRFSTYATWWIRQTI
ERAIMNQTRTIRLPIHIVKELNVYLRTARELSHKLDHEPSAEEIAEQLDKPVDDVSRMLRLNERITSVDTPLGGDSE
KALLDILADEKDNGPEDTTQDDDMKQSIVKWLFELNAKQREVLARRFGLLGYEAATLEDVGREIGLTRERVRQIQVE
GLRRLREILQGQGLNIEALFRE

>Enterobacter cloacae
MSQNTLKVHDLNEDAEFDENGAEAFDEKALVEEEPSDNDLAEEELLSQGATQRVLDATQLYLGEIGYSPLLTAEEEV
YFARRALRGDVASRRRMIESNLRLVVKIARRYGNRGLALLDLIEEGNLGLIRAVEKFDPERGFRFSTYATWWIRQTI
ERAIMNQTRTIRLPIHIVKELNVYLRTARELSHKLDHEPSAEEIAEQLDKPVDDVSRMLRLNERITSVDTPLGGDSE
KALLDILADEKDNGPEDTTQDDDMKQSIVKWLFELNAKQREVLARRFGLLGYEAATLEDVGREIGLTRERVRQIQVE
GLRRLREILQGQGLNIEALFRE

>Phytobacter diazotrophicus

MSQNTLKVHDLNEDAEFDENGVEAFDEKALVEEEPSDNDLAEEELLSQGATQRVLDATQLYLGEIGYSPLLTAEEEV
YFARRALRGDVASRRRMIESNLRLVVKIARRYSNRGLALLDLIEEGNLGLIRAVEKFDPERGFRFSTYATWWIRQTI
ERAIMNQTRTIRLPIHIVKELNVYLRTARELSHKLDHEPSAEEIAEQLDKPVDDVSRMLRLNERITSVDTPLGGDSE
KALLDILADEKENGPEDTTQDDDMKQSIVKWLFELNAKQREVLARRFGLLGYEAATLEDVGREIGLTRERVRQIQVE
GLRRLREILQAQGLNIEALFRE

>Kosakonia sacchari
MSQNTLKVHDLNEDAEFDENGVEAFDEKALVEEEPSDNDLAEEELLSQGATQRVLDATQLYLGEIGYSPLLTAEEEV
YFARRALRGDVASRRRMIESNLRLVVKIARRYSNRGLALLDLIEEGNLGLIRAVEKFDPERGFRFSTYATWWIRQTI
ERAIMNQTRTIRLPIHIVKELNVYLRTARELSHKLDHEPSAEEIAEQLDKPVDDVSRMLRLNERITSVDTPLGGDSE
KALLDILADEKDNGPEDTTQDDDMKQSIVKWLFELNAKQREVLARRFGLLGYEAATLEDVGREIGLTRERVRQIQVE
GLRRLREILQTQGLNIEALFRE

>Huaxiibacter chinensis
MSQNTLKVHDLNEDAEFDENGTESFDEKVLVEEEPSDNDLAEEELLSQGATQRVLDATQLYLGEIGYSPLLTAEEEV
YFARRALRGDVASRRRMIESNLRLVVKIARRYSNRGLALLDLIEEGNLGLIRAVEKFDPERGFRFSTYATWWIRQTI
ERAIMNQTRTIRLPIHIVKELNVYLRTARELSHKLDHEPSAEEIAEQLDKPVDDVSRMLRLNERITSVDTPLGGDSE
KALLDILADEKDNGPEDTTQDDDMKQSIVKWLFELNAKQREVLARRFGLLGYEAATLEDVGREIGLTRERVRQIQVE
GLRRLREILQGQGLNIEALFRE

14. ArgR

>Salmonella typhimurium
MRSSAKQEELVRAFKALLKEEKFSSQGEIVLALQDQGFENINQSKVSRMLTKFGAVRTRNAKMEMVYCLPAELGVPT
TSSPLKNLVLDIDYNDAVVVIHTSPGAAQLIARLLDSLGKAEGILGTIAGDDTIFTTPASGFSVRDLYEAILELFEQ
EL

>Escherichia coli
MRSSAKQEELVKAFKALLKEEKFSSQGEIVAALQEQGFDNINQSKVSRMLTKFGAVRTRNAKMEMVYCLPAELGVPT
TSSPLKNLVLDIDYNDAVVVIHTSPGAAQLIARLLDSLGKAEGILGTIAGDDTIFTTPANGFTVKDLYEAILELFDQ
EL

>Citrobacter rodentium
MRSSAKQEELVKAFKALLKEEKFSSQGEIVLALQDQGFDNINQSKVSRMLTKFGAVRTRNAKMEMVYCLPAELGVPT
TSSPLKNLVLDIDYNDAVVVIHTSPGAAQLIARLLDSLGKAEGILGTIAGDDTIFTTPANGFTVKDLYEAILELFEQ
EL

>Klebsiella variicola
MRSSAKQEELVKAFKALLKEEKFSSQGEIVQALQEEGFENINQSKVSRMLTKFGAVRTRNAKMEMVYCLPAELGVPT
TSSPLKNLVLDIDYNDAVVVIHTSPGAAQLIARLLDSLGKAEGILGSIAGDDTIFTTPARGFTVKDLHDAILVLFEQ
EL

>Cronobacter turicensis
MRSSAKQEELVKAFKALLKEEKFSSQGEIVQALQDQGFENINQSKVSRMLTKFGAVRTRNAKMEMVYCLPVELGVPT
TSSPLKNLVLDIDYNDAVVVIHTSPGAAQLIARLLDSLGKAEGILGTIAGDDTIFTTPARGFTVKDLYEAILMLFEQ
EL

>Enterobacter cloacae
MRSSSKQEELVKAFKALLKEEKFSSQGEIVQALQEQGFDNINQSKVSRMLTKFGAVRTRNAKMEMVYCLPAELGVPT
TSSPLKNLVLDIDHNDAVVVIHTSPGAAQLIARMLDSLGKTEGILGTIAGDDTIFTTPANGFSVKDLHEAILVLFEQ
EL

>Phytobacter diazotrophicus
MRSSAKQEELVKAFKALLKEEKFSSQGEIVLALQEQGFDNINQSKVSRMLTKFGAVRTRNAKMEMVYCLPVEMGVPT
TSSPLKNLVLDIDYNDAVVVIHTSPGAAQLIARLLDSLGKAEGILGTIAGDDTIFTTPASGFTVKELYEAILVLFEQ
EL

>Kosakonia sacchari
MRSSAKQEELVKAFKALLKEEKFSSQGEIVQALQEEGFDNINQSKVSRMLTKFGAVRTRNAKMEMVYCLPIELGVPT
TSSPLKNLVLDIDYNDAVVVIHTSPGAAQLIARLLDSLGKAEGILGTIAGDDTIFTTPAKGFTVKDLYEAILVLFEQ
EL

>Huaxiibacter chinensis
MRSTSKQEELIKAFKALLKEEKFGSQGEIVQALQEQGFDNINQSKVSRMLTKFGAVRTRNAKMEMVYCLPAELGVPT
TSSPLKNLVLDIDYNDAVVVIHTSPGAAQLIARLLDSLGKAEGILGTIAGDDTIFTTPASNFSVKELHEAILVLFEQ
EL

15. GalR

>Salmonella typhimurium
MATIKDVARLAGVSVATVSRVINDSPKASEASRLAVTSAMESLSYHPNANARALAQQATETLGLVVGDVSDPFFGAM
VKAVEQVAYHTGNFLLIGNGYHNEQKERQAIEQLIRHRCAALVVHAKMIPDADLASLMKQIPGMVLINRILPGLEHR
CVALDDRYGAWLATRHLIQQGHTRIGYICSNHTISDAEDRLRGYYDALAESHIPANDRLVTFGEPDESGGEQAMTEL
LGRGRNFTAVACYNDSMAAGAMGVLNDNGVGVPGEVSLIGFDDVLVSRYVRPRLTTIRYPIVTMATQAAELALALAG
KCPTPEVTHVFSPTLVRRHSVSTPTDTGHLSTTD

>Escherichia coli
MATIKDVARLAGVSVATVSRVINNSPKASEASRLAVHSAMESLSYHPNANARALAQQTTETVGLVVGDVSDPFFGAM
VKAVEQVAYHTGNFLLIGNGYHNEQKERQAIEQLIRHRCAALVVHAKMIPDADLASLMKQMPGMVLINRILPGFENR
CIALDDRYGAWLATRHLIQQGHTRIGYLCSNHSISDAEDRLQGYYDALAESGIAANDRLVTFGEPDESGGEQAMTEL
LGRGRNFTAVACYNDSMAAGAMGVLNDNGIDVPGEISLIGFDDVLVSRYVRPRLTTVRYPIVTMATQAAELALALAD
NRPLPEITNVFSPTLVRRHSVSTPSLEASHHATSD

>Citrobacter rodentium
MATIKDVARLAGVSVATVSRVINASPKASEASRLAVTSAMATLSYHPNANARALAQQSTETVGLVVGDVSDPFFGAM
VKAVEQVAYHTGNFLLIGNGYHNEQKERQAIEQLIRHRCAALVVHAKMIPDADLTALMEQMPGMVLINRILPGFEQR
CVALDDRYGAWLATRHLIQQGHTRIGYICSNHSISDAEDRLQGYYDALEESGIAVNDRLVTFGEPDESGGEQAMTEL
LGRGRNFTAVACYNDSMAAGAMGVLNDNGVEVPGEISLIGFDDVLVSRYVRPRLTTVRYPIVTMATQAAELALALAE
KRPLPDITHVFSPTLVRRHSVSSPAEPGPSASNE

>Klebsiella variicola
MATIKDVARLAGVSVATVSRVINNSPKASEASRQSVGAAMETLNYHPNANARALAQQSTETVGLVVGDVSDPFFGAM
VKAVEQVAYRTGNFLLIGNGYHNVQKERQAIEQLIRHRCAALVVHAKMIPDEELAGLMKQIPGMVLINRILPGYETR
CVALDDRYGAWLATRHLIQQGHTRIGYLCSNHHISDAEDRLQGYYAALEESGLPCNDRLVAFAEPDESGGELAMTEL
LGRGRHFSAVACYNDSMAAGAMGVLNDNGIDVPREISLIGFDDVLISRYVRPRLTTVRYPIVTMATQAAELALALAE
YRPAPEITHLFSPTLVRRHSVVAPLEGDKS

>Cronobacter turicensis
MPTIKDVARLAGVSVATVSRVINDSPKASDASRQAVLSAMEQLNYHPNANARALAQQTTETIGLVVGDVSDPFFGAM
VKAVEQVAYHTGNFLLIGNGYHSEQKERQAIEQLIRHRCAALVVHAKTIPDEDLIPLMKQIPGMVLINRILPGFEAR
CVALDDRYGAWLATRHLIQQGHTRIGYLCSNHAISDAEDRLQGYYDALREHDLPCNDRLVAWAEPDESGGEQAMTEL
LGQGKQFTAVACYNDSMAAGAMGVLNDNGIAVPQEISLIGFDDVLVSRYVRPRLTTIRYPIVTMATQAAELALALAD
NLPPPEITNIFTPTLVRRHSVVAPAETDKS

>Enterobacter cloacae
MATIKDVARLAGVSVATVSRVINDSPKASDASRQAVQNAMESLNYHPNANARALAQQSTETIGLVVGDVSDPFFGAM
VKAVEQVSYQTGNFLLIGNGYHNEQKERQAIEQLIRHRCAALVVHAKMIPDAELIHLMKQIPGMVIINRIIPGFEKR
CVALDDRYGAWLATRHLIQQGHTRIGYLCSNHPISDAEDRLQGYYDALRENGLPCNDRLVAYGEPDESGGEQAMTEL
LGRGRNFTAVASYNDSMAAGAMGVLNDNGIEVPGEISLIGFDDVLVSRYVRPRLTTVRYPIITMATQAAELALALAE
QRQPPEITHLFSPTLVRRHSVGAPAEAPDE

>Phytobacter diazotrophicus
MATIKDVARLAGVSVATVSRVINDSPKASEASRLAVQTAMESLSYHPNANARALAQQSTETVGLVVGDVSDPFFGAM
VKAVEQVASSTGNFLLIGNGYHNEQKERNAIEQLIRHRCAALVVHAKMVPDAELIALMKQIPGMVLINRILPGFEQR

CVSLDDRYGAWLATRHLIQQGHTRIGYLCSNHAISDAEDRLQGYYDALKENGLPCNERLVTFGEPDESGGEQAMTEL
LGRGKNFTAIACYNDSMAAGAMGVLNDNGIAVPDEISLIGFDDVLISRYVRPRLTTVRYPIVTMATQAAELALALAE
NRPLPDVTHVFNPTLVRRHSVVTPQDN

>Kosakonia sacchari
MATIKDVARLAGVSVATVSRVINDSPKASESSRQAVQSAMDALNYHPNANARALAQQSTETVGLVVGDVSDPFFGAM
VKAVEQVASATGNFLLIGNGYHNEEKERKAIEQLIRHRCAALVVHAKTLPDDELASLMKQMPGMVLINRILPGFEQR
CVALDDRYGAWLATRHLIQQGHTKIGYLCSNHAISDAEDRLQGYYDALKENGLPCNDRLVTFAEPDESGGEQAMTEL
LGRGRNFTAVACYNDSMAAGAMGVLNDNGIEVPREISLIGFDDVLVSRYVRPRLTTVRYPIVTMATQAAELALALAE
NRPLPEITHLFNPTLVRRHSVIAPQE

>Huaxiibacter chinensis
MATIKDVARMAGVSVATVSRVINHSPKASDTSRQAVMSAMETLNYHPNANARALAQQSTETVGLVVGDVSDPFFGAM
VKAVEHVAYETGNFLLIGNGYHNEHKERQAIEQLIRHRCAALVVHAKVLPDEELLHLMKQIPGMVLINRIIPGYEKR
CVALDDRYGAWLATRHLIQQGHTRIGYLCSNHQISDAEDRLQGYHDALREHGLPSNERLVTYAEPDESGGEQAMTEL
LGRGKNFTAVACYNDSMAAGAMGVLNDNGIDVPAEISLIGFDDVLVSRYVRPRLTTVRYPIVTMATQAAELALALAD
NRPLPDITHLFSPTLVRRHSVVAPPDAVSE

Transport:

1. LysP

>Salmonella typhimurium
MGSKTKTTEAPALRRELKARHLTMIAIGGSIGTGLFVASGATISQAGPGGALFSYILIGLMVYFLMTSLGELAAYMP
VSGSFATYGQNYVEEGFGFALGWNYWYNWAVTIAVDLVAAQLVMGWWFPDTPGWIWSALFLCVIFLLNYISVRGFGE
AEYWFSLIKVATVIIFIIVGVAMIIGIFKGVEPVGWSNWTTGDAPFAGGFAAMIGVAMIVGFSFQGTELIGIAAGES
ENPEKNIPRAVRQVFWRILLFYVFAILIISLIIPYTDPNLLRNDVKDISVSPFTLVFQHAGLLSAAAIMNAVILTAV
LSAGNSGMYASTRMLYTLACDGKAPRIFAKLSRGGVPRNALYATTVIAGLCFLTSMFGNQTVYLWLLNTSGMTGFIA
WLGIAISHYRFRRGYVLQGYDVNDLPYRSGFFPLGPIFAFVLCLIITLGQNYEAFLKDTIDWGGVAATYIGIPLFLL
IWFGYKLIKGTHFVRYSEMHFPERVKK

>Escherichia coli
MVSETKTTEAPGLRRELKARHLTMIAIGGSIGTGLFVASGATISQAGPGGALLSYMLIGLMVYFLMTSLGELAAYMP
VSGSFATYGQNYVEEGFGFALGWNYWYNWAVTIAVDLVAAQLVMSWWFPDTPGWIWSALFLGVIFLLNYISVRGFGE
AEYWFSLIKVTTVIVFIIVGVLMIIGIFKGAQPAGWSNWTIGEAPFAGGFAAMIGVAMIVGFSFQGTELIGIAAGES
EDPAKNIPRAVRQVFWRILLFYVFAILIISLIIPYTDPSLLRNDVKDISVSPFTLVFQHAGLLSAAAVMNAVILTAV
LSAGNSGMYASTRMLYTLACDGKAPRIFAKLSRGGVPRNALYATTVIAGLCFLTSMFGNQTVYLWLLNTSGMTGFIA
WLGIAISHYRFRRGYVLQGHDINDLPYRSGFFPLGPIFAFILCLIITLGQNYEAFLKDTIDWGGVAATYIGIPLFLI
IWFGYKLIKGTHFVRYSEMKFPQNDKK

>Citrobacter rodentium
MVSETKTTEAPALRRELKARHLTMIAIGGSIGTGLFVASGATISAAGPGGALFSYILIGLMVYFLMTSLGELAAFMP
VSGSFATYGQNYVEEGFGFALGWNYWYNWAVTIAVDLVAAQLVMNWWFPDTPGWIWSALFLGVIFLLNYISVRGFGE
AEYWFSLIKVATVIIFIIVGIMMIVGIFKGVQPVGWSNWTTGDAPFAGGFAAMIGVAMIVGFSFQGTELIGIAAGES
ENPEKNIPRAVRQVFWRILLFYVFAILIISLIIPYTDPSLLRNDVKDISVSPFTLVFQHAGLLSAAAVMNAVILTAV
LSAGNSGMYASTRMLYTLACDGKAPRIFAKLSRGGVPRNALYATTVIAGLCFLTSMFGNQTVYLWLLNTSGMTGFIA
WLGIAISHYRFRRGYVLQGNDVNDLPYRSGFFPLGPIFAFVLCLIITLGQNYEAFLKDTIDWGGVAATYIGIPLFLV
IWFGYKLVKGTHFVRYSEMRFPERLKK

>Klebsiella variicola
MVSETKTTEAPTLRRELKARHLTMIAIGGSIGTGLFVASGATISQAGPGGALLSYILIGLMVYFLMTSLGELAAFMP
VSGSFATYGQNYVEEGFGFALGWNYWYNWAVTIAVDLVASQLVMSYWFPDTPGWIWSALFLGIMFLLNWISVRGFGE
AEYWFSLIKVATVIIFIIVGVMMIVGIFKGSQPTGWSNWGIADAPFAGGFSAMIGVAMIVGFSFQGTELIGIAAGES
EDPEKNIPRAVRQVFWRILLFYVFAILIISLIIPYTDPSLLRNDVKDISVSPFTLVFQHAGLLSAAAIMNAVILTAV
LSAGNSGMYASTRMLYTLACDGKAPRIFSKLSRGGVPRNALYATTVIAALCFLTSMFGNQTVYLWLLNTSGMTGFIA

```
WLGIAISHYRFRRGYVMQGNDINNLPYRSGFFPLGPIFAFVLCLIITLGQNYEAFLKDTIDWGGVAATYIGIPLFLV
IWFGYKLAKGTRFVRYSEMTFPERFKR

>Cronobacter turicensis
MVSESKTTQAPGLRRELKARHLTMIAIGGSIGTGLFVASGATISQAGPGGALFSYILIGLMVYFLMTSLGELAAYMP
VSGSFSTYGQKYVEEGFGFALGWNYWYNWAVTIAVDLVASQLVMSYWFPDTPGWIWSALFLGIMFLLNVISVKGFGE
AEYWFSLIKVTTVIIFIVVGIAMIVGIFKGSEPAGWSNWKIGDAPFAGGFSAMIGVAMIVGFSFQGTELIGIAAGES
EEPEKNIPRAVRQVFWRILLFYVFAILIISLIIPYTDPSLLRNDVKDISVSPFTLVFEHAGLLGAAAIMNAVILTAV
LSAGNSGMYASTRMLYTLACDGKAPRIFSKLSKGGVPRNALYATTIIAGLCFLTSMFGNQTVYLWLLNTSGMTGFIA
WLGIAISHYRFRRGYVLQGNDINDLPYRSGFFPLGPIFAFVLCLIITLGQNYQAFLADTIDWAAVAATYIGIPLFLI
IWFGYKLTKGSRFVSYRDMEFPDHLKK

>Enterobacter cloacae
MVSETKTTEAPALRRELKARHLTMIAIGGSIGTGLFVASGATISAAGPGGALFSYILIGLMVYFLMTSLGELAAYMP
VSGSFSTYGQKYVEEGFGFALGWNYWYNWAVTIAVDLVAAQLVMTWWFPDTPGWIWSALFLAVIFLLNYISVRGFGE
AEYWFSLIKVATVIIFIVVGVAMIVGIFKGAEPAGWSNWTIGDAPFAGGFSAMIGVAMIVGFSFQGTELIGIAAGES
ENPEKNIPRAVRQVFWRILLFYVFAILIISLIIPYTDPSLLRNDVKDISVSPFTLVFQHAGLLSAAAVMNAVILTAV
LSAGNSGMYASTRMLYTLACDGKAPRIFAKLSRGGVPRNALYATTVIAGLCFLTSMFGNQTVYLWLLNTSGMTGFIA
WLGIAISHYRFRRGYVMQGHDLNNLPYRSGFFPLGPIFAFILCLIITLGQNYEAFLADTIDWGAVTATYIGIPLFLV
IWFGYKLMKGTHFVRYSEMEFPERFNK

>Phytobacter diazotrophicus
MVSETKTTQAPTLRRELKARHLTMIAIGGSIGTGLFVASGATISQAGPGGALFSYILIGLMVYFLMTSLGELAAYMP
VSGSFATYGQQYVEEGFGFALGWNYWYNWAVTIAVDLVASQLVMTWWFPDTPGWIWSAVFLCVIFLLNYISVKGFGE
AEYWFSLIKVTTVIIFIIVGVLMIVGIFKGDQPAGWSNWQVGDAPFAGGLSAMIGVAMIVGFSFQGTELIGIAAGES
EDPEKNIPRAVRQVFWRILLFYVFAILVISLIIPYTDPSLLRNDVKDISVSPFTLVFEHAGLLSAAAVMNAVILTAV
LSAGNSGMYASTRMLYTLACDGKAPRIFAKLSKGGVPRNALYATTVIAGLCFLTSMFGNQTVYLWLLNTSGMTGFIA
WLGIAISHYRFRRGYVMQGHDLNNLPYRSGFFPLGPIFAFVLCLIITLGQNYQAFLADTIDWGAVAATYIGIPLFLI
IWFGYKLSKGTHFVSYKDMVFPERFKK

>Kosakonia sacchari
MVSETKTTEAPTLRRELKARHLTMIAIGGSIGTGLFVASGATISSAGPGGALFSYILIGLMVYFLMTSLGELAAFMP
VSGSFSTYGQKYVEEGFGFALGWNYWYNWAVTIAVDLVASQLVMTWWFPDTPGWIWSALFLAVIFLLNYISVKGFGE
AEYWFSLIKVATVIIFIIVGVMMIVGIFQGEKPAGWSNWAIGDAPFAGGFSAMIGVAMIVGFSFQGTELIGIAAGES
EDPEKNIPRAVRQVFWRILLFYVFAILIISLIIPYTDPSLLRNDVKDISVSPFTLVFEHAGLLSAAAVMNAVILTAV
LSAGNSGMYASTRMLYTLACDGKAPRIFAKLSKGGVPRNALYATTVIAALCFLTSMFGNQTVYLWLLNTSGMTGFIA
WLGIAISHYRFRRGYVAQGHSLDNLPYRSGFFPLGPIFAFVLCLIITLGQNYQAFLADSIDWGAVAATYIGIPLFLI
IWLGYKFSKGTRFVRYSEMEFPERFTK

>Huaxiibacter chinensis
MVSETKTTEAPALRRALKARHLTMIAIGGSIGTGLFVASGATISAAGPGGALFSYILIGLMVYFLMTSLGELAAYMP
VSGSFSTYGQKYVEEGFGFALGWNYWYNWAVTIAVDLVAAQLVMTWWFPDTPGWIWSALFLAVIFLLNYISVRGFGE
AEYWFSLIKVTTVIIFIVVGVAMIVGIFKGAEPAGWSNWAIGDAPFAGGFAAMIGVAMIVGFSFQGTELIGIAAGES
EDPEKNIPRAVRQVFWRILLFYVFAILIISLIIPYTDPSLLRNDVKDISVSPFTLVFQHAGLLSAAAVMNAVILTAV
LSAGNSGMYASTRMLYTLACDGKAPRIFAKLSRGGVPRNALYATTVVAGLCFLTSMFGNQTVYLWLLNTSGMTGFIA
WLGIAISHYRFRRGYVLQGHDINDLPYRSGFFPLGPIFAFVLCLIITLGQNYEAFLSDTIDWGGVMATYIGIPLFLI
IWFGYKLSKGTRFVRYSEMDFPGRFKK
```

2. btuB

```
>Salmonella typhimurium
MIKKATLLTAFSVTAFSAWAQDTSPDTLVVTANRFQQPRSAVLAPVTIVTRQDIERWQSTSVNDVLRRLPGVDIAQS
GGAGQNSSIFIRGTNSSHVLVLIDGVRLNLAGVSGSADLSQFPVSLVQRIEYIRGPRSAIYGSDAIGGVVNIITTRD
NPGTELTAGWGSNSYQNYDISTQQQLGENTRATLIGDYEYTKGFDVVAKGGTGMQAQPDRDGFLSKTLYGALEHTFS
DRWSGFVRGYGYDNRTDYDAYYSPGSPLIDTRKLYSQSWDAGLHFNGERIQSQLVSSYSHSKDYNYDPHYGRYDTSA
TLDEMKQYNVQWTNSVVVGHGNVGAGVDWQKQTTTPGTGYVPEGYDQRNTGVYLTGLQQLGDFTLEAAARSDDNSQF
GRHGTWQTSAGWEFIEGYRFIASYGTSYKAPNLGQLYGYYGNPNLNPEKSKQWEGAFEGLTAGVSWRISGYRNDIND
```

MIDYDDHLQKYYNEGKARIKGIEATANFDTGPLTHTVSYDYVDARNAITDTPLPRRSKQMAKYQLDWDVYDFDWGMT
YQYLGSRYDSDYSAYPYRTVKMGGVSLWDLTVAYPVTSHLTVRGKIANLFDKDYETVYGYQTAGREYTLSGSYTF

>Escherichia coli
MIKKASLLTACSVTAFSAWAQDTSPDTLVVTANRFEQPRSTVLAPTTVVTRQDIDRWQSTSVNDVLRRLPGVDITQN
GGSGQLSSIFIRGTNASHVLVLIDGVRLNLAGVSGSADLSQFPIALVQRVEYIRGPRSAVYGSDAIGGVVNIITTRD
EPGTEISAGWGSNSYQNYDVSTQQQLGDKTRVTLLGDYAHTHGYDVVAYGNTGTQAQTDNDGFLSKTLYGALEHNFT
DAWSGFVRGYGYDNRTNYDAYYSPGSPLLDTRKLYSQSWDAGLRYNGELIKSQLITSYSHSKDYNYDPHYGRYDSSA
TLDEMKQYTVQWANNVIVGHGSIGAGVDWQKQTTTPGTGYVEDGYDQRNTGIYLTGLQQVGDFTFEGAARSDDNSQF
GRHGTWQTSAGWEFIEGYRFIASYGTSYKAPNLGQLYGFYGNPNLDPEKSKQWEGAFEGLTAGVNWRISGYRNDVSD
LIDYDDHTLKYYNEGKARIKGVEATANFDTGPLTHTVSYDYVDARNAITDTPLLRRAKQQVKYQLDWQLYDFDWGIT
YQYLGTRYDKDYSSYPYQTVKMGGVSLWDLAVAYPVTSHLTVRGKIANLFDKDYETVYGYQTAGREYTLSGSYTF

>Citrobacter rodentium
MIKKMSLLTALSVTAFSGWAQDATPDTLVVTANRFQQPVNTVLAPTDIVTRQDIERWQSRTVLDVMRRLPGVDVAQN
GGMGQSSSLFVRGTEAKHVLVLVDGIPVARPGITNNSDIDQIPVSLVQRIEYIRGPRSAIYGSGAIGGVVNIITMAG
EERSQINAGVGSKGYQQYDGAWRQRFGDTVVTAAGAYQTTKGFDVQPRSSYSGDGDRDGYRNKLFWGSVEHKFNDNF
DGYFRGYGYASNADYDQGNWGYDGGNDEHQNYTQSWDTGLRFNSGIYSSQLVANYQRIKDYNYSSLNGRYATGYSLD
DMEQRYIQWGNNVVVGHGAISAGVDWKQEKLMSFGEYGTDNYKRDNTGLYLTGQQQIDSVTLEASGREDHDEQFGWH
GTWQTAAGWQFVDDYKVTLSYGTGFLAPSLGQQFGAERFGIASNPNLKPEESKQWEAGLEGLTGPVDWRLSTYRYKI
QNLIDYNDNTYFNVKSATIKGLEWTGNLTTGPVDHRLTLQYVDPRDDETDQQLYRRAKQQVKYELSGEVYELGWDVT
YQYIGERYDYDYDNSRRVKMGGLSVWDIGLSYPVTSHLTVRGKIANLFDKDYETVYGYQTAGREYTLSGSYTF

>Klebsiella variicola
MIKKASLMTALSVTAFSGWAQDSNSDTLVVTANRFQQPVNTVLAPTDIVTRDDIDRWQSKDLNDVMRRLPGVDIARN
GGMGQSASLYVRGTEARHVLVLIDGVPMARPGISNGVDISQIPISLVQRVEYIRGPRSAVYGSGAIGGVVNIITMTD
SDRSQVNVGMGSNGYQTYDGAINKRFGDTVVTAAGAYQTTKGFNVQPNSPYSGDSDRDGYRNKLFLGGVQHKFDDNF
SGFFRGYGYTANTDYDQGSYGYVGGNDERQNYTQSWDTGLRYSSGIYSSQLIANYQRIKDYNYSSDAGRYAAGATLD
DMEQRYIQWGNNIEVGHGAISGGVDWKQEKLTSSSTTLSDAYKRDTTGLYLTGQQQIDSVTLEASGREDHDEQFGWH
GTWQTAAGWEFIDGYRATLSYGTGFLAPSLGQQYGATRFASFYGPGIASNPNLKPEESKQWEAGIDGLTGPLDWRIS
AYHYEIQNLIDYKNNQYINVKSATIKGLEWTGNVTTGPVEHHLTLQYVDPRDDDTGKVLYRRAKQQVKYELTGQIYD
LDWNVMYQYLGKRYDDDYDNGRDVKMGGLSLWDVGLSYPVTSHLTVRGKIANLFDKDYETVYGYQSAGREYTLSGSY
TF

>Cronobacter turicensis
MIKKISLLTALSVTAFSGWAQDSGSNSLVVTANRFQQPVNTVLAPIDVVTREQIDKWQSKTVLDVLRRLPGVDISQN
GGMGQLASVYVRGTDSRHALILMDGVPVSRPNLSNDSNLYQIPISLIQRIEFIRGPRSAVYGSGAIGGVINIITQSD
DEVSRIDAGVGSKGYQEYSGTLRQRFGDTVVTAMGAYQTTHGFNVQPGSTWRHDNDRDGYRNKTFFGSLQHHFSDNV
DGFFRGYGYANNADYDVGSPPFSPAYSADENQYDNQSWDTGLRYNADIYSSQLIASFQKLKSYNYSSLYGRYQDGTT
LDRMEQRYIQWGNNLIVGHGSVSAGVDWKQEKLTSATQADTDRYKRDTTGIYLTGQQQVLEHVTLEASGREDHDEQF
GWHGTWQTAAGWEFVDGYRATLSYGTGFLAPGLTQQFGSPQYGIASNPNLQPEKSRQWEVGIEGITGPLDWRLSTWR
YKIDNLISYDNDAYYNVKSATIKGVEWTGNLTTGPVSHQLTLQYIDPRDDETHERLQRRARQQVKYNLSGEVSDFGW
DITYQYIGEREDTNFNTFPSEKVKLGSVSLWDVAVSYPVTSHLTVRGKIANLFDKDYETAYGYATAGREYNLSGSYT
F

>Enterobacter cloacae
MIKKVSLLTALSVTAFSGWAQDSADSLVVTANRFEQPAKTVLAPTSVVTREDIERWQAKSVVEIMSRLPGVDIAQSG
GLGATSSTFIRGTESRHVLVLIDGVPLNSAGISNVPDLSQIPTSLIQRIEYVRGPRSALYGSDAIGGVINIITGRDK
PGAEISAGVGSKGYQTYDGAFQQVLNKTKITMAGNYTYTRGFDIAAQDAPRQPDRDGFMSKSLYGSVEQQITDSVSG
FFRGFGYDNRTAYDGYDHYDANFMVDGRPDTRQLYSQNWDTGLRYSQGIFQSQLVAGYGRSKDQNYDPKKGRYADSA
TMDDVKQYTTQWLNTIEVGHGNIGTGLDWQKQKTQAGTGYLEKGYEQRNTGVFASAMQQFNSVTLEAAARNDDNSNF
GNHGTWQTSAAWEFVDGYRVIASYGTAFKAPTMSQIHSASYGNPDLKPEESKQWEGGFEGLTGPVNWRITGYRNDID
NLISSDPHTYRYYNVDEARIKGVEATAQFETGPVGHQISYDYVDPRNAKTHEVLARRSKQQVKYQLDWQVWDLDWNL
AYRYLGTRYDVAIDPDTYASERVKMGGVSLWDVAVSYPVTSHLTVRGKIANLFDKDYETVYGYQTAGREYTLSGSYT
F

>Phytobacter diazotrophicus

MIKKASLLTALSVTAFSGWAQDGNPDTLVVTANRFQQPVNTVLAPTDVVTREDIQRWQSKDLNDVMRRLPGVDIAQY
GGIGQSSSLFVRGTEARHVLVLIDGVPMARAGIGNAIDIGQIPVSLVQRIEYIRGPRSAVYGSGAIGGVVNIITMSD
DERAQINAGMGTNGYQNYDGAINQRFGDTMVTAAGAYQTTKGFNVQPDSTYADDSDRDGYRNKLFWGGVQHKFNDSV
SGFFRGYGYSSNTDYDQGSGGYAGGGDESQNYTQSWDTGLNYHSGIYSSQLIANYQHLKNYNYNAQDGRYAASASLD
NMEQRYIQWGNNVEVGHGSVSGGVDWKQEKLQSSGTSSTDIYKRDTTGLYLTGQQQIDKVTLEASGREDHDEQFGWH
GTWQTAAGWEFVDDYRVTLLRDRIPGAIAWAAVWRGAVWHCFQPEFETGRVEAMGSRA

>Kosakonia sacchari
MIKKASLLTALSVTAFSGWAQDSSSDNLVVTANRFQQPAKTVLAPTEVVTREDIDRWQSRSLVDVLRRLPGVDIAQN
GGLGQTASVYVRGTEAKHLLLLIDGVPVARSGISNDPELNQIPLSLVQRIEYIRGPRSAVYGSGAIGGVVNVITMTG
NEKSQINAGVGSKGYQTYDGTLRQRFGDTVATAAGSYTSTRGFNVQPGSTWDHDEDRDGYRNKTFWGSLQHKFNDNF
DGFFRGYNFSNNVDYDLGSAPFSADYSADERQLFVQGWDTGLNFAQGIYSSQLLASYQKSKDYNYSSIYGRYNDGTT
LDDMEQRYIQWGNNVNVGKGSVGAGVDWKQERLVSSNSTTRDAYERENTGLYLNGMQQFGDVTLEASGREDKDDEFG
WHGTWQTAAGWEFVENYRLTVSYGTGFLAPSLGQQFGAKRFGIASNPNLKPEESRQWEAGVEGLTGPLDWRLSAYRY
EIENLITYSDTAYYNINSATIKGVEWTGSVDTGIFSHRVTLQYIDPRDDETNEVLPRRAKRQAKYQLDWSVLNVDMD
LAWEYFGKRYDNRTSTYNPEQRILPSYSMVDFSASYPVTSHLTVRGKIANLFDKEYETAYGYQTAGREYTLSGSYTF

>Huaxiibacter chinensis
MIKKVSLMTALSVTAFSGWAQDSADSLVVTANRFQQPVNTVLAPTDVVTREDIDRWQSRTVLDVMRRLPGVDIAQNG
GMGQSSSLFVRGTEAKHVLVLVDGIPVARPGISNSSDIDQIPVSLVQRIEYIRGPRSAIYGSGAIGGVVNIITMTGE
ERSQINAGVGSKGYQEYDGTYRKRFDDTVVTAAGAYQTTKGFNVQPHSTYSGDNDRDGYRNKLFWGGIEHKFNDYFD
GSFRGYGYASNADYDQGNYGYDGGNDEHQNYTQSWDAAMRFHSGIYSSQLVANYQRIKDYNYSSQTWRYAPGYSLDD
MEQRYIQWGNNVIVGHGAISGGVDWKQEKLMSLGTTSSDHYKRDNTGLYLTGQQQIDSVTLEASGREDHDEQFGWHG
TWQTAAGWEFVEDYKVTLSYGTGFLAPSLGQQFGAERFGISSNPNLKPEESKQWEAGLEGLTGPVDWRLSTYRYEIQ
NLIDYNNNAYFNVKSATIKGLEWTANVTTGPVDHRLTLQYVDPRDDETGKQLYRRAKQQVKYDLSGQAYSLGWDVTW
QYIGERYDYDYDNARRVQMGGVSLWDVALSYPVTSHLTVRGKIANLFDKDYETVYGYQTAGREYTLSGSYTF

3. GlnH

>Salmonella typhimurium
MKSLLKVSLAALTLAFAVSSHAADKKLVVATDTAFVPFEFKQGDKYVGFDVDLWDAIAKELKLDYTLKPMDFSGIIP
ALQTKNIDLALAGITITDERKKAIDFSDGYYKSGLLVMVKANNNDIKSVKDLDGKVVAVKSGTGSVDYAKANIKTKD
LRQFPNIDNAYMELGTNRADAVLHDTPNILYFIKTAGNGQFKAVGESLEAQQYGVAFPKGSDELREKVNGALKTLRE
NGTYNEIYKKWFGTEPK

>Escherichia coli
MKSVLKVSLAALTLAFAVSSHAADKKLVVATDTAFVPFEFKQGDKYVGFDVDLWAAIAKELKLDYELKPMDFSGIIP
ALQTKNVDLALAGITITDERKKAIDFSDGYYKSGLLVMVKANNNDVKSVKDLDGKVVAVKSGTGSVDYAKANIKTKD
LRQFPNIDNAYMELGTNRADAVLHDTPNILYFIKTAGNGQFKAVGDSLEAQQYGIAFPKGSDELRDKVNGALKTLRE
NGTYNEIYKKWFGTEPK

>Citrobacter rodentium
MKSVLKVSLAALTLAFAVSSHAADKKLVVATDTAFVPFEFKQGDKYVGFDIDLWAAVAKELKLDYELKPMDFSGIIP
ALQTKNVDLALAGITITDERKKAIDFSDGYYKSGLLVMVKANNNDIKSVKDLDGKTLAVKSGTGSVDYAKANIKTKD
LRQFPNIDNAYMELGTNRADAVLHDTPNILYFIKTAGNGQFKAVGESLEAQQYGIAFPKGSDDLREKVNGALKTLRD
NGTYNEIYKKWFGTEPK

>Klebsiella variicola
MKSVFKVSLAALTLAFAVSSHAANKTLVVATDTAFVPFEFKQGDKYVGFDVDLWAAIAKELKLDYTLKPMDFSGIIP
ALQTKNIDLALAGITITDERKKAIDFSDGYYKSGLLVMVNANNNDIKDVKDLNGKVVAVKSGTGSVDYAKANIKTKD
LRQFPNIDNAYMELGTGRADAVLHDTPNILYFIKTAGNGKFKAVGESLEAQNYGIAFPKGSDELREKVNGALKTLRE
NGTYNEIYKKWFGTEPK

>Cronobacter turicensis

MKSVFKVSLAALTLAFAVSSQAADKLVVATDTAFVPFEFKQGDKYVGFDVDLWAAIAKELKLDYTLKPMDFSGIIPA
LQTKNVDVALAGITITDERKKAIDFSDGYYTSGLLVMVKANNNDVKSVKDLDGKVLAVKSGTGSVDYAKANIKTKDL
RQFPNIDNAYMELGTNRADAVLHDTPNILYFIKTAGNGQFKAVGESLAGQQYGIAFPKGSDDLRNKVNGALKNLKEN
GTYNEIYKKWFGTEPK

>Enterobacter cloacae
MKSVLKVSLAALTLAFAVSSQAADKLVVATDTAFVPFEFKQGDKYVGFDVDLWAAIAKELKLDYTLKPMDFSGIIPA
LQTKNVDLALAGITITDERKKAIDFSDGYYKSGLLVMVKADNNDVKSVKDLDGKVVAVKSGTGSVDYAKANIKTKDL
RQFPNIDNAYMELGTNRADAVLHDTPNILYFIKTAGNGKFKAVGESLEAQQYGIAFPKGSDDLRTKVNGALKTLKEN
GTYNEIYKKWFGTEPK

>Phytobacter diazotrophicus
MKSVFKLSLATLALAFAVSSQAANKLVVATDTAFVPFEFKQGDKYVGFDVDLWAAIAKELKLDYELKPMDFSGIIPA
LQTKNVDLALAGITITEERKKAVEFSDGYYKSGLMVMVKANNNDVKSVKDLDGKVVAVKSGTGSVDYAKANIKTKDL
RQFPNIDNAYMELGTNRADAVLHDTPNILYFIKTAGNGQFKAVGDSLEAQQYGIAFPKGSDELREKVNGALKTLRDN
GTYNEIYKKWFGTEPK

>Kosakonia sacchari
MKSVLKFSLAALALAFAVSSQAADKKLVVATDTAFVPFEFKQGNQYVGFDVDLWAAIAKELKLDYELKPMDFSGIIP
ALQTKNIDLALAGITITEERKKAIDFSDGYYKSGLLVMVKANNNDVKSVKDLDGKVLAVKSGTGSVDYAKANIKTKD
LRQFPNIDNAYMELGTGRADAVLHDTPNILYFIKTAGNGKFKAVGDSLEAQQYGIAFPKGSDELREKVNGALKTLHD
NGTYNEIYKKWFGTEPK

>Huaxiibacter chinensis
MKSVLKVSLAALTLAFAVSSHAADKLVVATDTAFVPFEFKQGDKYVGFDVDLWAAVAKELKLDYTLKPMDFSGIIPA
LQTKNIDLALAGITITEERKKAIDFSDGYYKSGLLVMVKADNNDVKSVKDLDGKVVAVKSGTGSVDYAKANIKTKDL
RQFPNIDNAYMELGTNRADAVLHDTPNILYFIKTAGSGKFKAVGESLEAQQYGIAFPKGSDELRNKVNGALKTLKEN
GTYNEIYKKWFGTEPK

4. ModC

>Salmonella typhimurium
MLELNFSQTLGTHCLTLNETLPASGITAIFGVSGAGKTSLINAISGLTRPQKGRIALNGRVLHDAENGICLTPEKRR
IGYVFQDARLFPHYKVRGNLRYGMAKSMTGQFDKLVSLLGIEALLDRLPGSLSGGEKQRVAIGRALLTAPELLLLDE
PLASLDIPRKRELLPYLQRLAREINIPMLYVSHSLDEILHLADKVMVLEDGQVKAFGPLEEVWGSSVMHPWLPKEQQ
SSILKVSVLEHHPHYAMTALALGDQHLWVNKLNQPLQSTLRIRIQASDVSLVLQPPQQTSIRNVLRAKVANCYDDNG
QVEVQLEIGGRTLWARISPWARDELNIKPGLWLYAQVKSVSITA

>Escherichia coli
MLELNFSQTLGNHCLTINETLPANGITAIFGVSGAGKTSLINAISGLTRPQKGRIVLNGRVLNDAEKGICLTPEKRR
VGYVFQDARLFPHYKVRGNLRYGMSKSMVDQFDKLVALLGIEPLLDRLPGSLSGGEKQRVAIGRALLTAPELLLLDE
PLASLDIPRKRELLPYLQRLTREINIPMLYVSHSLDEILHLADRVMVLENGQVKAFGALEEVWGSSVMNPWLPKEQQ
SSILKVTVLEHHPHYAMTALALGDQHLWVNKLDEPLQAALRIRIQASDVSLVLQPPQQTSIRNVLRAKVVNSYDDNG
QVEVELEVGGKTLWARISPWARDELAIKPGLWLYAQIKSVSITA

>Citrobacter rodentium
MLELHFSQTLGAHCLTIDETLPASGITAIFGVSGAGKTSLINAISGLTKPQKGRIVLNGRVLNDAENGICLTPEKRR
VGYVFQDARLFPHYKVRGNLRYGMAKSMVSQFDKLVALLGIEPLLDRLPGGLSGGEKQRVAIGRALLTAPELLLLDE
PLASLDIPRKRELLPYLQRLAREINIPMLYVSHSLDEILHLADKVMVLENGQVKAFGALEEVWGSSVMHPWLPKEQQ
SSILKVSVLEHHPHYAMTALALGDQHVWVNKLDEPLHTALRIRIQASDVSLVLQPPQQSSIRNVLRAKVANCYDVDG
QVEVQLEVGGKTLWARISPWARDELTIKPGLWLYAQIKSVSITA

>Klebsiella variicola
MLELDFTQILGSHCLQIRETLPASGITAIFGVSGAGKTSLINAISGLTRPQTGRIVLSGRVLNDTAQRICLAPEQRR
IGYVFQDARLFPHYKVRGNLRYGMAKSMVSQFDKLVELLGIAPLLDRLPGRLSGGEKQRVAIGRALLTAPELLLLDE
PLASLDIPRKRELLPYLQRLAQEIHIPMLYVSHSLDEIQHLADRVLVLEAGKVKAFGPLEEVWSSSVMHPWLPAEQQ

STILSATVAAQHPQYAMTALTLGDQLLWVNRLERPAGDTARIRIQASDVSLTLAQPSGTSIRNILRAEVAQCLEVNG
QIEVQLRVSGRLLWARISPWARDDLAIAPGQQVFAQIKSVSIAA

>Cronobacter turicensis
MLELDFTQILGSHCLQIRETLPASGITAIFGVSGAGKTSLINAISGLTRPQTGRIVLSGRVLNDTAQRICLAPEQRR
IGYVFQDARLFPHYKVRGNLRYGMAKSMVSQFDKLVELLGIAPLLDRLPGRLSGGEKQRVAIGRALLTAPELLLLDE
PLASLDIPRKRELLPYLQRLAQEIHIPMLYVSHSLDEIQHLADRVLVLEAGKVKAFGPLEEVWSSSVMHPWLPAEQQ
STILSATVAAQHPQYAMTALTLGDQLLWVNRLERPAGDTARIRIQASDVSLTLAQPSGTSIRNILRAEVAQCLEVNG
QIEVQLRVSGRLLWARISPWARDDLAIAPGQQVFAQIKSVSIAA

>Enterobacter cloacae
MLELHFTQTLGNHTLTLDETLPATGITAIFGVSGAGKTSLINAIGGLTRPQAGRIVVNNRVLNDVENKIYLTPDKRR
IGYVFQDARLFPHYSVRGNLRYGMAKSMAAQFDKLVALLGIEPLLDRLPSSLSGGEKQRVAIGRALLTAPELLLLDE
PLASLDIPRKRELLPYLQRLAREIKIPMLYVSHSLDEILHLADNVLVLENGSVKAFGNLEEVWGSSVMHPWLPREQQ
SSILKVSVLEHHPHYAMTALALGDQHLWVNKIDTPLQSALRIRIQASDVSLVLQPPLQTSIRNILRAKVAECFDDNG
QVEVQLEVGSRTLWARISPWARDELGIKPGLWLYAQIKSVSITT

>Phytobacter diazotrophicus
MLELNFSQTLGTHRLQVAENLPASGITAVFGVSGAGKTSLINAISGLTRPEQGRIVLNDRVLYDAEKKICLPPEKRR
VGYVFQDARLFPHYNVLGNLRYGMAKSMAGQFDKLVALLGIEQLLERLPGGLSGGEKQRVAIGRALLTAPELLLLDE
PLASLDIPRKRELLPYLQRLAREINIPVLYVSHSLDEILHLADKVMVLEAGAVKAFGNLEDVWGSSVMNPWLPQEQQ
SSILKVSVLEQHPHYAMTALALGDQHIWVNRLDKPLQAAVRIRIQATDVSLVLQPSGNTSIRNTLRAKVVQNYDLDG
QVEVQLDVSGRTLWARISPWARDELNIKPGLWLYAQIKSVSITA

>Kosakonia sacchari
MLELNFTQTLGTHRLRIAEKLPASGITAIFGVSGAGKTSLINAIGGLTHPQQGRIALNNRVLFDTESKICLPPEKRR
VGYVFQDARLFPHYKVRGNLRYGMAKSMAGQFDKLVALLGIEPLLDRLPGGLSGGEKQRVAIGRALLTAPELMLLDE
PLASLDIPRKRELLPYLQRLAREINIPMLYVSHSLDEILHLADKVMVLEAGEVKAFGSLEDVWGSRVMNPWLPAEQQ
SSVLKVVVLEHHPHYAMTALALGDQHIWVNKVDLPLQSVLRIRIQATDVSLVLQPPMNTSIRNILRAKVAQCFDIDG
QVEVQLEVGSRTLWARISPWARDELAVKPGQWLYAQIKSVSITA

>Huaxiibacter chinensis
MLELNFTQTLGNHTLTLNETLPASGITAVFGVSGAGKTSLINAISGLTRPQTGRIVLNDRVLTDVDRKIFLAPERRR
IGYVFQDARLFPHYKVRGNLRYGMAKSMGAHFDHLVSLLGIEALLDRLPSSLSGGEKQRVAIGRALLTAPDLLLLDE
PLASLDIPRKRELLPYLQRLAREINIPMLYVSHSLDEILHLADKVLVLENGNVKAFGNLEEVWGSSVMHPWLPKEQQ
SSILKVSIMEHHPHYAMTALALGDQHLWVNRIDKPLQTALRIRIQASDVSLVLQPPLQTSIRNILRAKVAQCFDDNG
QVEVQLDVGSKTLWARISPWARDELGIKPGLWLYAQIKSVSITT

5. ZitB

>Salmonella typhimurium
MAHSHSHADSHLPKDNNARRLLFAFIVTAGFMLLEVVGGILSGSLALLADAGHMLTDAAALLFALLAVQFSRRPPTV
RHTFGWLRLTTLAAFVNAIALVVITLLIVWEAIERFYTPRPVAGNLMMVIAVAGLLANLFAFWILHRGSDEKNLNVR
AAALHVMGDLLGSVGAIVAALIIIWTGWTPADPILSILVSVLVLRSAWRLLKDSVNELLEGAPVSLDINALQRHLSR
EIPEVRNVHHVHVWMVGEKPVMTLHAQVIPPHDHDALLERIQDFLMHEYHIAHATIQMEYQVCHGPDCHLNQTSSGH
VHHH

>Escherichia coli
MAHSHSHTSSHLPEDNNARRLLYAFGVTAGFMLVEVVGGFLSGSLALLADAGHMLTDTAALLFALLAVQFSRRPPTI
RHTFGWLRLTTLAAFVNAIALVVITILIVWEAIERFRTPRPVEGGMMMAIAVAGLLANILSFWLLHHGSEEKNLNVR
AAALHVLGDLLGSVGAIIAALIIIWTGWTPADPILSILVSLLVLRSAWRLLKDSVNELLEGAPVSLDIAELKRRMCR
EIPEVRNVHHVHVWMVGEKPVMTLHVQVIPPHDHDALLDQIQHYLMDHYQIEHATIQMEYQPCHGPDCHLNEGVSGH
SHHHH

>Citrobacter rodentium
MAHAHSHSTPNLPEDNNARRLLLAFCVTAGFMLLEVAGGILSGSLALLADAGHMLTDAAALLFALLAVQFARRPPTV
RHTFGWLRLTTLAAFVNAIALVVITLLIVWEAIERFYTPRPVAGGMMMTIAIAGLLANILAFWILHRGSEEKNLNVR

AAALHVMGDLLGSVGAIAAALIIIWTGWTPADPILSILVSLLVLRSAWRLLKDSVNELLEGAPLSLDIAALRRRLSQ
EVPEVRNVHHVHVWMVGEKPVMTLHAQVIPPHDHDALLARIQHFLAHHYQIEHATIQMEYQPCHGADCHLNQTASGH
AHHHH

>Klebsiella variicola
MAHPHSHSPAQAPDSSNARRLRWAFIVTAGFMLIEAVGGAISGSLALLADAGHMLTDSAALLFALLAVRFASRPPNT
RHTFGWLRLTTLAAFLNAIALVVITILIVWEAIQRFQHPQPVAGVTMMVIAVAGLLANVLAFWILHRGSEEQNLNVR
AAALHVLGDLLGSVGAIVAAVVILTTGWTPVDPILSVLVSCLVLRSAWRLLKESLNELLEGAPRSLDVAALQRDIRR
SIPEVRDVHHVHVWLVGEKPVMTLHVQVVPPHDHDALLNRILHFLEHKYEIEHATVQMEYQPCSGPECHLNTMHAGH
DHHHHH

>Cronobacter turicensis
MAHSHTHDPHEHSHNSNSKRLLIAFLITAIFMVLEIAGGLLSGSLALLADAGHMFTDAAALLVALMAVRFARRSPNA
RHTFGLLRLTTLAAFVNALALLVITAIIVWEAIARFITPEPVAGGPMLGIAIAGLVANLLSFWILHRGSDEKNMNVR
AAALHVLGDLLGSVGAIVAAVIILWTGWTPIDPILSILVSCLVLRSAWRLLQESMNELLEGAPRAVDVEQLRRRLVR
EIPEARDVHHVHLWLVGEKPVMTLHVQVIPPHDHDALMASIHDYLRHHYQIAHATVQLEYQSCSVKDCDLNARDAKP
HAHGHSH

>Enterobacter cloacae
MAHTHSHASGDDNAKRLLLAFGVTATFMIIEVIGGLISGSLALLADAGHMLTDAAALLFALLAVQFARRPPNARHTF
GWLRLTTLAAFVNAIALVVITLLIVWEAIQRFRHPQPVAGMTMMAIAVAGLLANLLAFWILHRGSGEKNLNVRAAAL
HVMGDLLGSVGAIVAALVILYTGWTPVDPILSVLVSCLVLRSAWRLLKESVNELLEGAPASVDIAELKRNLHRSVPE
VRNVHHVHVWLVGEKPVMTLHVQVIPPHDHDALLERIQHFLEHHYEIGHATIQMEYQPCNGPDCHLNEAQSDHSHHH
HH

>Phytobacter diazotrophicus
MAHSHSHSHLPADANARRLLLAFSVTALFMLVEVVGGLVSGSLALLADAGHMLTDAAALFFALLAVHFAHRPSGPRR
TFGWLRLTTLAAFVNAIALVLITFFIVWEAIQRFYHPQPVAGMTMMVIAVMGLLANILAFWLLHRGNEERNLNVRAA
ALHVLGDLLGSVGAIAAALIIMFTGWTPADPILSILVSVLVLRSAWALLRESVNELLEGAPTSLDINALKRHLRRAI
SEVRDVHHVHVWLVGEKALMTLHVQVIPPHDHDGLLRQIEHFLVHHYQIEHVTIQMEYQPCSGPDCHLSETQPDHSH
SHHH

>Kosakonia sacchari
MAHSHSHTPADANARRLLLAFIVTAVFMVIEVVGGLISGSLALLADAGHMLTDAAALLFALLAVYFARQPPSTRRTF
GWLRLTTLAAFINALALVLITVWIVWEAIQRFTHPQPVAGLTMMLIAIAGLVANVLAFWLLHRGSEEKNLNVRAAAL
HVLGDLLGSVGAIAAALVILFTGWTPIDPILSVLVSALVLRSAWRLMKESVNELLEGAPGSMDIGALQRHLRRAIPE
VRDVHHVHVWLVGEKPLMTLHVQVIPPRDHDKLLGEIHHFLAHHYEIEHATVQMEYQPCSGPDCHLGETQPDHHHSH
SH

>Huaxiibacter chinensis
MAHSHSPASGDDNAKRLMLAFGVTALFMVIEVIGGLVSGSLALLADAGHMLTDAAALLFALMAVHFARRPPNTRHTF
GWLRLTTLAAFVNAIALVVITILIVWEAFQRFSHPQPVAGATMMAIAVAGLVANILSFWILHRGSAEQNLNVRAAAL
HVLGDLLGSVGAIVAALVILYTGWTPVDPILSVLVSCLVLRSAWRLLKESVNELLEGAPVSLNIPQLKRDLCRSVPE
VRNVHHVHVWQVGEKPVMTLHVQVVPPHDHDALLGRIQHFLAHHYEIGHATIQMEYQPCNGPDCHLNETQPGHTHHH
HH

Cell envelope/cell surface related:

1. OmpA

>Salmonella typhimurium
MKKTAIAIAVALAGFATVAQAAPKDNTWYAGAKLGWSQYHDTGFIHNDGPTHENQLGAGAFGGYQVNPYVGFEMGYD
WLGRMPYKGDNINGAYKAQGVQLTAKLGYPITDDLDVYTRLGGMVWRADTKSNVPGGPSTKDHDTGVSPVFAGGIEY
AITPEIATRLEYQWTNNIGDANTIGTRPDNGLLSVGVSYRFGQQEAAPVVAPAPAPAPEVQTKHFTLKSDVLFNFNK
STLKPEGQQALDQLYSQLSNLDPKDGSVVVLGFTDRIGSDAYNQGLSEKRAQSVVDYLISKGIPSDKISARGMGESN
PVTGNTCDNVKPRAALIDCLAPDRRVEIEVKGVKDVVTQPQA

>Escherichia coli
MKKTAIAIAVALAGFATVAQAAPKDNTWYTGAKLGWSQYHDTGFINNNGPTHENQLGAGAFGGYQVNPYVGFEMGYD
WLGRMPYKGSVENGAYKAQGVQLTAKLGYPITDDLDIYTRLGGMVWRADTKSNVYGKNHDTGVSPVFAGGVEYAITP
EIATRLEYQWTNNIGDAHTIGTRPDNGMLSLGVSYRFGQGEAAPVVAPAPAPAPEVQTKHFTLKSDVLFNFNKATLK
PEGQAALDQLYSQLSNLDPKDGSVVVLGYTDRIGSDAYNQGLSERRAQSVVDYLISKGIPADKISARGMGESNPVTG
NTCDNVKQRAALIDCLAPDRRVEIEVKGIKDVVTQPQA

>Citrobacter rodentium
MKKTAIAIAVALAGFATVAQAAPKDNTWYTGAKLGWSQYHDTGFIDNNGPTHENQLGAGAFGGYQVNPYVGFEMGYD
WLGRMPYKGSVENGAYKAQGVQLTAKLGYPITDDLDIYTRLGGMVWRADTKSHNNLTGASTKDHDTGVSPVFAGGIE
WAMTRDIATRLEYQWTNNIGDANTIGTRPDNGLLSVGVSYRFGQQEEAAPIAPAPAPAPEVQTKHFTLKSDVLFNFN
KATLKPEGQQALDQMYSQLSNLDPKDGSVVVLGFTDRIGSDTYNQGLSEKRAQSVVDYLISKGIPADKISARGMGES
NPVTGSTCDNVKARAALIDCLAPDRRVEIEVKGVKDVVTQPQA

>Klebsiella variicola
MKKTAIAIAVALAGFATVAQAAPKDNTWYAGGKLGWSQFHDTGWYNSSLNNNGPTHESQLGAGAFGGYQVNPYLGFE
MGYDWLGRMPYKGDQVNGAFKAQGVQLTAKLGYPITDDLDIYTRLGGMVWRADSSNSIAGDNHDTGVSPVFAGGVEW
AVTRDIATRLEYQWVNNIGDAGTVGVRPDNGMLSVGVSYRFGQEDAAPVVAPAPAPAPEVTTKHFTLKSDVLFNFNK
ATLKPEGQQALDQLYTQLSNMDPKDGSAVVMGFTDRIGSEAYNQQLSEKRAQSVVDYLVAKGIPAGKISARGMGKSN
PVTGNTCDNVKARAALIDCLAPDRRVEIEVKGYKEVVTQPAA

>Cronobacter turicensis
MKKTAIAIAVALAGFATVAQAAPKDNTWYAGGKLGWSQFHDTGFIPNDGPTHESQLGAGAFGGYQVNPYVGFEMGYD
WLGRMPYKGDTVNGAFKAQGVQLTAKLGYPVTDDLDVYTRLGGMVWRADSSSNIGGDDHDTGVSPVFAGGVEWAMTR
DIATRLEYQWVNNIGDAQTVGARPDNGMLSVGVSYRFGQQEDAAPVVAPAPAPAPEVQTKHFTLKSDVLFNFNKATL
KPEGQQALDQMYSQLSNLDPKDGSVVVLGFTDRIGSDSYNQGLSEKRAQSVVDYLISKGIPSNKISARGMGESNPVT
GNTCDNVKPRAALIECLGPDRRVEIEVKGVKDVVTQPQA

>Enterobacter cloacae
MKKTAIAIAVALAGFATVAQAAPKDNTWYAGGKLGWSQFHDTGWYNSANNNDGPTHESQLGAGAFGGYQVNPYVGFE
MGYDWLGRMPYKGNETNGAFKAQGVQLTAKLGYPVTDDLDVYTRLGGMVWRADSSNSNAGDDHDTGVSPVFAGGVEW
AMTRDIATRLEYQWVNNIGDGATVGVRPDNGMLSVGVSYRFGQQEDAAPVVAPAPAPAPEVQTKHFTLKSDVLFNFN
KATLKPEGQQALDQLYTQLSNLDPKDGSVVVLGFTDRIGSDAYNQKLSEKRAQSVVDYLISKGIPANKISPRGMGES
NPVTGNTCDNVKARAALIDCLAPDRRVEIEVKGVKDVVTQPAA

>Phytobacter diazotrophicus
MKKTAIAIAVALAGFATVAQAAPKDNTWYAGGKLGWSQFHDTGFIANDGPTHSSQLGAGAFGGYQVNPYVGFEMGYD
WLGRMPYKGDNVNGAFKAQGVQLTAKLGYPITDDLDIYTRLGGMVWRADAKDNSGFKDHDTGVSPVFAGGVEWAVTR
DIATRLEYQWVNNIGDANTVGTRPDNGMLSVGVSYRFGQQEEAAPVVAPAPAPAPEVQTKHFTLKSDVLFNFNKATL
KPEGQQALDQLYGQLSNLDPKDGSVVVLGFTDRIGSDAYNQGLSEKRAQSVVDYLVSKGIPANKISARGMGKSQPVT
GSTCDNVKARPALIDCLAPDRRVEIEVRGSKDVVTQPQA

>Kosakonia sacchari
MKKTAIAIAVALAGFATVAQAAPKDNTWYTGGKLGWSQYHDKGAVWQNDGPTHESQLGAGAFGGYQVNPYVGFELGY
DWLGRMPYKGDTVNGAFKAQGVQLTAKLGYPVTDDLDLYTRLGGMVWRADAQNNQGFKDHDTGVSPVFAGGVEWAVT
RDIATRLEYQWVNNIGDANTVGARPDNGMLSVGVSYRFGQQEDAAPVVAAPAPAPAPEVQTKHFTLKSDVLFNFNKA
TLKPEGQQALDQLYGQLSNLDPKDGSVVVLGFTDRIGSDAYNQGLSEKRAQSVVDYLVSKGIPANKISARGMGKSNP
VTGSTCDNVKPRAALIDCLAPDRRVEIEVKGSKDVVTQPQA

>Huaxiibacter chinensis
MKKTAIAIAVALAGFATVAQAAPKDNTWYAGGKLGWSQFHDTGWYNSSLNNDGPTHESQLGAGAFGGYQVNPYVGFE
MGYDWLGRMPYKGDNVNGAFKAQGVQLTAKLGYPVTDDLDVYTRLGGMVWRADSSNSIAGDDHDTGVSPVFAGGVEW
AMTRDIATRLEYQWVNNIGDGNTVGVRPDNGMLSVGVSYRFGQQEAAPVVAPAPAPAPEVQTKHFTLKSDVLFNFNK
ATLKPEGQQALDQLYSQLSNLDPKDGNVVVLGFTDRIGSDAYNQGLSEKRAQSVVDYLISKGIPANKISPRGMGESN
PVTGNTCDNVKPRAALIDCLAPDRRVEIEVKGIKDVVTQPAA

2. ExbB

>Salmonella typhimurium
MGNNLMQTDLSVWGMYQHADIVVKCVMIGLILASVVTWAIFFSKSVEFFTQKRRLKREQLQLADARSLDQASDIAAG
FSAKSLSAQLINEAQNELELSQGSEDNEGIKERTGFRLERRVAAVGRYMGRGNGYLATIGAISPFVGLFGTVWGIMN
SFIGIAQTQTTNLAVVAPGIAEALLATAIGLVAAIPAVVIYNIFARQIGSYKATLGDVAAQVLLLQSRDLDLNASAS
AQPVRAAQKLRVG

>Escherichia coli
MGNNLMQTDLSVWGMYQHADIVVKCVMIGLILASVVTWAIFFSKSVEFFNQKRRLKREQQLLAEARSLNQANDIAAD
FGSKSLSLHLLNEAQNELELSEGSDDNEGIKERTSFRLERRVAAVGRQMGRGNGYLATIGAISPFVGLFGTVWGIMN
SFIGIAQTQTTNLAVVAPGIAEALLATAIGLVAAIPAVVIYNVFARQIGGFKAMLGDVAAQVLLLQSRDLDLEASAA
AHPVRVAQKLRAG

>Citrobacter rodentium
MGNNLMQTDLSVWGMYQHADIVVKCVMIGLILASVVTWAIFFSKSVDFFSQRRRLKREQQQLAEARSLDQASDIAAS
FGAKSLNTQLLNEAQNELELSAGSEDNEGIKERTGFRLERRVAAAGRQMGRGNGYLATIGAISPFVGLFGTVWGIMN
SFIGIAQTQTTNLAVVAPGIAEALLATAIGLVAAIPAVVIYNIFARQIGGLKATLSDIAAQVLLLQSRDLDLDASAS
AHPVRTAQKLRVG

>Klebsiella variicola
MGNNLMQADLSVWGMYHHADIVVKVVMIGLILASVVTWAIFFGKGAEILASKRRLKREQQQLAEARSLDQASDIASA
FEAKSLTTQLINEAQNELELSAGAEDNEGIKERTGFRLERRVAAVGRHMGRGNGYLATIGAISPFVGLFGTVWGIMN
SFIGIAQTQTTNLAVVAPGIAEALLATAIGLFAAIPAVVIYNIFARMIGSYKASLGDVAAQVLLLQSRDLDLSASGV
KPVRSAQKLRVG

>Cronobacter turicensis
MGNNLMQTDLSVWGMYQHADIVVKIVMIGLILASVVTWAIFFSKSVELSGHKRRLKREQQELAGARTLDDAREMAER
FGPKSLSARLIEEAQNERELSAGCEDNEGIKERTGFRLERRVAAAGRHMGRGNGYLATIGAISPFVGLFGTVWGIMN
SFIGIAQSQTTNLAVVAPGIAEALLATAIGLIAAIPAVVIYNIFARTIGGYKATLGDVAAQVLLLQSRDLDLEASAH
AQPVRTAQKLRLG

>Enterobacter cloacae
MGNNLMQTDLSVWGMYHHADIVVKIVMIGLILASVITWAIFFGKSAELISQKRRLKREQQQLAEARSLDQASDMTSS
FHAKSLTTLLVNEAQNELELSAGSEDNEGIKERTGFRLERRVAAVGRHMGRGNGYLATIGAISPFVGLFGTVWGIMN
SFIGIAQTQTTNLAVVAPGIAEALLATAIGLVAAIPAVVIYNVFARMIGSYKATLGDVAAQVLLLQSRDLDLNASAA
KPVHAASKLRVG

>Phytobacter diazotrophicus
MGNNLMQADLSVWGMYQHADIVVKIVMIGLILASVVTWAIFFSKSAEMIAHKRRLKREQQLLADARSLDQASDIASA
FHARSLSTQLINEAQNELELSAGSEDNEGIKERTGFRLERRVAATGRHMGRGNGYLATIGAISPFVGLFGTVWGIMN
SFIGIAQTQTTNLAVVAPGIAEALLATAIGLVAAIPAVVIYNIFARMIGGYKATLGDVAAQVLLLQSRDLDLAASAA
AKPVHAAQKLRVG

>Kosakonia sacchari
MGNNLMQTDLSVWGMYQHADIVVKVVMIGLILASVVTWALFFSKSVEMISHKRRLKREQQQLADARTLDQASEIASA
FHARSLSTMLINEAQNELELSAGSEDNEGIKERTGFRLERRVAATGRHMGRGNGYLATIGAISPFIGLFGTVWGIMN
SFIGIAQTQTTNLAVVAPGIAEALLATAIGLVAAIPAVVIYNIFARMIGSYKATLGDVAAQVLLLQSRDLDLAASNA
QPVRTAQKLRVG

>Huaxiibacter chinensis
MGNNLMQTDLSVWGMYHHADIVVKIVMIGLILASVITWAIFFSKSVELISQKRRLKREQQQLAEARSLDQASDMTSS
FHAKSLTTLLVNEAQNELELSAGSEDNEGIKERTGFRLERRVAAIGRHMGRGNGYLATIGAISPFVGLFGTVWGIMN
SFIGIAQTQTTNLAVVAPGIAEALLATAIGLVAAIPAVVIYNIFARMIGSYKATLGDVAAQVLLLQSRDLDLNASSA
KPVHSASKLRVG

3. DsbC

>Salmonella typhimurium
MKKRFMMFTLLAAVFSGVAHADDAAIRQSLAKLGVQSTEIQASPVAGMKTVLTHSGVLYVTDDGKHIIQGPMYDVSG
AHPVNVTNKLLMSQLNALEKEMIVYKAPDEKHVITVFTDITCGYCHKLHEEMKDYNALGITVRYLAFPRQGLESQAE
QDMKSIWCAKDKNKAFDDAMAGKGVKPASCDVNIADHYALGVQLGVSGTPAIVLSNGYVVPGYQGPKEMKAFLDEHQ
KQTSGK

>Escherichia coli
MKKGFMLFTLLAAFSGFAQADDAAIQQTLAKMGIKSSDIQPAPVAGMKTVLTNSGVLYITDDGKHIIQGPMYDVSGT
APVNVTNKMLLKQLNALEKEMIVYKAPQEKHVITVFTDITCGYCHKLHEQMADYNALGITVRYLAFPRQGLDSDAEK
EMKAIWCAKDKNKAFDDVMAGKSVAPASCDVDIADHYALGVQLGVSGTPAVVLSNGTLVPGYQPPKEMKEFLDEHQK
MTSGK

>Citrobacter rodentium
MKKGLMIFTLLATAFSGLAHADDAAIRQSLTKLGVQSTDIQPAPVAGMKTVMTNSGVLYVTEDGKHIIQGPMYDVSG
ASPVNVTNQLLMKNLNALEKEMIVYKAPQEKHVITVFTDITCGYCHKLHEEMKDYNALGITVRYLAFPRQGLESQAE
KDMKSIWCAKDKNKAFDDAMAGKGVQAATCDIDIANHYALGVQFGVSGTPAMVLSNGYVVPGYQGPKELKAFLDAHQ
KQTSGK

>Klebsiella variicola
MKKGLLMFTLLAASLSGAAHADSAAIKQSLAKLGVQSTDIQPSPVSGMSTVLTDSGVLYVTDDGKHIIQGPMYDVSG
AQPINVTNQLLVGKLNALSNEMIVYKAPKEQHVITVFTDITCGYCHKLHEQMSDYNALGITVRYLAFPRQGLQSQAE
QDMKAIWCAKDRNKALDDAMNGKGVQPASCNVDIAKHYTLGVQMGVNGTPAMVLSNGMVLPGYQGPKELKAFLDEHK
KQTSGN

>Cronobacter turicensis
MKKTIALLSVTLAAFSGFAQADDAAIKRSLTKLGVANAEIQPSPLAGMKTVLTESGVIYVTEDGKHIIQGPLYDVSG
GQPVNVTNQMLMTKLNALEKEMIVYKAPQEKHVITVFTDITCGYCHKLHEEMKDYNALGITVRYLAFPRQGPRSEPA
KDMQAIWCAKDRNKAFDNAMGGGKVAAASCDVDTAKHYELGVQFGVQGTPAIVLSNGAMVPGYQGPKEMKAFLDEHQ
KQLQASGK

>Enterobacter cloacae
MKKSFALFTLLAASFTGFAHADDAAIKQSLTKLGVTSSDIQPAPVAGMKTVLTNSGVLYVTEDGKHIIQGPMYDVSG
AQPVNVTNQLLMKNLNALEKEMIVYKAAQEKHVITVFTDITCGYCHKLHEEMKDYNALGITVRYLAFPRAGVQSQPE
QDMKAIWCAKDRNKAFDDAMNGKGVQPASCDIDIANHYALGVQFGVTGTPAIVLSNGYVVPGYQGPKEMKEFLDAHQ
KQFGGK

>Phytobacter diazotrophicus
MKKGFIMFTLLAAALTSTAHADDAAIKQSLAKLGVQSSDIQPAPVAGMKTVLTNSGVLYVTDDGKHIIQGPMYDVSG
AQPVNVTNQLLTAHLNALEKEMIVYKAPQEKHVITVFTDITCGYCHKLHEEMADYNALGITVRYLAFPRQGLQSQAE
QDMKAIWCAKDRNKALDDAMSGKGVKPASCDLDIADHYALGVQFGVNGTPAVVLNDGYLVPGYQGPKEMKAFLDEHQ
KQTGGK

>Kosakonia sacchari
MKKGLVMFTLLAAAFSGAAHADDAAIKQSLAKLGVQSGEVLPAPVAGMKAVLTNSGVLYVTEDGKHIIQGPMYDVSG
AQPVNVTTKMLLPHLNALEKEMIVYKAPQEKHVITVFTDITCGYCQKLHSEMADYNALGITVRYLAFPRQGVPSEVE
NQMKAIWCAKDPKKAFDNAMEGKEVKPASCDIDIANHYALGVQFGVNGTPAIVLNDGYLVPGYQAPAEMKAFLDQHQ
KATGGK

>Huaxiibacter chinensis
MKKSFALFTLLAASFTGLAHADDAAIKQSLAKLGVTSSEIQPAPVAGMKTVLTNSGVLYVTEDGKHFIQGPMYDVSG
AQPVNVTNQLLMKNLNALEKEMIVYKAAQEKHVITVFTDITCGYCHKLHEEMKDYNALGITVRYLAFPRAGVQSQPE
QDMKAIWCAKDRNKAFDDAMNGKGVQPASCDIDIANHYALGVQFGVSGTPAIVLNNGYVVPGYQGPKEMKAFLDAHQ
KQFGGK

4. LolA

```
>Salmonella typhimurium
MKKMAIACALLSSVVASSVWADAASSLKSRLDKVSSFHATFTQKVTDGSGAAVQEGQGDLWVKRPNLFNWHMTQPDE
SILVSDGKTLWFYNPFVEQATATWLKDATGNTPFMLIARNQASDWQQYNIKQDGDNFVLTPKASNGNLKQFTINVGR
DGTIHQFSAVEQDDQRSAYQLKSQQNGAVDPSKFTFTPPQGVTIDDQRK

>Escherichia coli
MKKIAITCALLSSLVASSVWADAASDLKSRLDKVSSFHASFTQKVTDGSGAAVQEGQGDLWVKRPNLFNWHMTQPDE
SILVSDGKTLWFYNPFVEQATATWLKDATGNTPFMLIARNQSSDWQQYNIKQNGDDFVLTPKASNGNLKQFTINVGR
DGTIHQFSAVEQDDQRSSYQLKSQQNGAVDAAKFTFTPPQGVTVDDQRK

>Citrobacter rodentium
MKKIAITCALLSGFVVSSVQADAAGDLKSRLDKVSSFHASFTQKVTDGSGAAVQEGQGDLWVKRPNLFNWHMTQPDE
SILVSDGKTLWFYNPFVEQATATWLKDATGNTPFMLIARNQSSDWQQYNIRQNGDDFVLTPKTGNGNLKQFTINVGR
DGTIHQFSAVEQDDQRSSYQLKSQQNGAVDAAKFTFTPPQGVTVDDQRK

>Klebsiella variicola
MKKLAITCALLSGMVVSQVWADAASDLKSRLDKVSSFHASFTQKVTDGSGNAVQDGQGDLWVKRPNLFNWHMTQPDE
SVLVSDGKTLWFYNPFVEQATATWLKDATSNTPFMLIARNQSSDWQQYNIKQNGDDFVLTPKSGSGNLKQFTINVGR
DGTIHQFSAVEQDDQRSSYQLKSQQNGAVDASKFTFTPPKGVTVDDQRK

>Cronobacter turicensis
MKKIAVTCALLSAFAVSSVWADAAGDLKSRLDKVSSFHASFTQKVTDGSGAAVQEGQGDLWVKRPNLFNWHMTQPDE
SVLISDGKTLWFYNPFVEQASATWLKDATSNTPFMLIARNQSSDWQQYNIKQNGDDFVLMPKASSGNLKQFTINVSR
DGTINQFSAVEQDDQRSNYELKSQQNGAVDMSKFTFTPPQGVTVDDQRNK

>Enterobacter cloacae
MKKIAIACALLTSFVASSVWADAASDLKSRLDKVSSFHASFTQKVTDGSGNAVQEGQGDLWVKRPNLFNWHMTQPDE
SILVSDGKTLWFFNPFVEQATATWLKDATSNTPFMLIARNQASDWQQYNIKQNGDEFVLTPKGSNGNLKQFTINVST
NGTINQFGAVEQDDQRSSYQLKSQQNGAVDASKFTFTPPQGVTVDDQRK

>Phytobacter diazotrophicus
MKKIAITCALIGSFMVSSVWADASSDLKSRLDKVSSFHASFTQKVTDGSGAAVQEGQGDLWVKRPNLFNWHMTQPDE
SVLVSDGKTLWFYNPFVEQATATWLKDATSNTPFMLIARNQSSDWQQYNIQQNGDDFVLTPKSSNGNLKQFTINVGS
DGTIHQFSAIEQDDQKSNYQLKAQQNGTVDAAKFTFTPPKGVTVDDQRK

>Kosakonia sacchari
MKRVALVCALMGSLLVSNVWADASSDLKSRLDKVSSFHATFTQKVTDGSGAAVQEGQGDLWVKRPNLFNWHMTQPDE
SVLVSDGKTLWFYNPFIEQATATWLKDATGNTPFMLIARNQASDWQQYNIEQKGDDFVLTPKGNNGNLKQFTINVGR
DGTIHQFSAIEQDDQRSSYQLKSQQNGTVDASKFTFTPPKGVTVDDQRK

>Huaxiibacter chinensis
MKKIAIVGALLTSFVASSVWADAASDLKNRLDKVSSFHASFTQKVTDGSGNAVQEGQGDLWVKRPNLFNWHMTQPDE
SVLVSDGKTLWFYNPFVEQATATWLKDATSNTPFMLIARNQTSDWQQYNIKQTGDDFVLTPKAGNGNLKQFTINVSS
NGTINQFGAVEQDDQRSNYQLKTQQNGAVDASKFTFTPPQGVTVDDQRNK
```

5. mtgA

```
>Salmonella typhimurium
MSKRRIAPLTFLRRLLLRILAALAVFWGGGIALFSVVPVPFSAVMAERQISAWLGGEFGYVAHSDWVSMADISPWMG
LAVIAAEDQKFPEHWGFDVPAIEKALAHNERNESRIRGASTLSQQTAKNLFLWDGRSWVRKGLEAGLTLGIETVWSK
KRILTVYLNIAEFGDGIFGVEAAAQRYFHKPASRLSLSEAALLAAVLPNPIRYKANAPSGYVRSRQAWIMRQMRQLG
GESFMTRNQLN

>Escherichia coli
MSKSRLTVFSFVRRFLLRLMVVLAVFWGGGIALFSVAPVPFSAVMVERQVSAWLHGNFRYVAHSDWVSMDQISPWMG
LAVIAAEDQKFPEHWGFDVASIEKALAHNERNENRIRGASTISQQTAKNLFLWDGRSWVRKGLEAGLTLGIETVWSK
```

KRILTVYLNIAEFGDGVFGVEAAAQRYFHKPASKLTRSEAALLAAVLPNPLRFKVSSPSGYVRSRQAWILRQMYQLG
GEPFMQQHQLD

>Citrobacter rodentium
MKKGLIVFLRRLIVRVALALALFWGGGIALFSVAPVPFSAVMAERQIGAWLRGDFGYVAHSDWVSMDEISPWMGLAV
IAAEDQHFPAHWGFDVAAIEKALSHNERHENRIRGASTLSQQTVKNLFLWDGRSWLRKGLEAGLTLGVETVWSKKRI
LTVYLNIAEFGDGVFGVEAAAQRYFGKPASRLTQSEAALLAAVLPNPLRYKAAAPSGYVRSRQAWIMRQMRQLGGET
FMTRNKLY

>Klebsiella variicola
MRLRVAPFALLKRLALRLLLIAAVFWGGGIALFSVLPVPFSAVMLERQVSAWLSGDVHYLAHSDWVGMDEISPWMGL
AVIAAEDQKFPEHWGFDVSAIEKALAHNERHDTRIRGASTLSQQTAKNLFLWDGRSWLRKGLEAGLTVGIETVWSKK
RILTVYLNIAEFGDGIFGVEAAAQRYFHKPASQLTPGEAALLAAVLPNPIRYRADAPSGYVRSRQAWILRQMRQLGG
EGFMREHKLY

>Cronobacter turicensis
MSKARGSLFVRLRRLALRAVLAVLGVWIAGILLFSVVPVPFSAVMVERQFSAWFSGDFSYVAHSDWVSMDEISPWMG
LAVIAAEDQRFPEHWGFDVAAIQKAVSHNERSSRIRGASTLSQQTAKNLFLWDGRSWLRKGLEAGLTLGIETVWSKR
RILTVYLNIAEFGDGVFGVEEASQRYFNKPASRLSASEAALLAAVLPNPLRYKATAPSGYVRARQQWILRQMRQLGG
ESFMAQHTLR

>Enterobacter cloacae
MSRKFGAGAWVKRALLRIVLVLAVFWGGGLALFSIMPVPFSAVMVERQLGAWLSGDFSYVAHSDWVSMDEISPFMGL
AVIAAEDQKFPEHWGFDVAAIEKALAHNERHENRVRGASTLSQQTAKNLFLWDGRSWVRKGLEAGLTLGMETVWSKK
RILTVYLNIAEFGDGVFGVEAAAQRYFNKPASRLSMSEAALLAAVLPNPIRFKANAPSGYVRSRQAWIMRQMRQLGG
EGFMQRNKLM

>Phytobacter diazotrophicus
MRGARGPLMAKIKKLLLRVVLALGIFWGGGILLFSFLPVPFSAVMVERQFGAWLTGDFGYVAHSDWVGMDEISPWAA
LAVIAAEDQRFPDHWGLDFGAIEKALSHNEQHENRIRGASTISQQTIKNLLLWDGKSWVRKGFEAGLTLITEAVWTK
RRILTVYLNIAEFGDGIFGVEAASQRYFHKPASKLTMAEAALLAAVLPNPVRFRADAPSGYVRSRQAWIMRQMRQLG
GEEFLERNKLH

>Kosakonia sacchari
MRKWFCGPLKATLKRITLRILCVLALFWGGGIVLFSFLPVPFSAVMVERQLGAWFSGDFGYVAHSDWVGMDDISPWM
ALAVVAAEDQRFPDHWGLDFSAIEKALSHNERHENRIRGASTISQQTVKNLLLWDGKSWVRKGLEAGLTLGMEAVWT
KRRILTVYLNIAEFGDGVFGVEEASQRYFHKPASRLTMSEAALLAAVLPNPIRFQAAAPSGYVRSRQAWILRQMRQL
GGEAFLERNKLY

>Huaxiibacter chinensis
MSKRRSPLAWVKRVLLRLVLFFVVLWGGGIALFSVLPVPFSAVMVERQLGAWLTGNFSYVAHSDWTSMDAISPWMGL
AVIAAEDQKFPEHWGFDVAAIEKALAHNERNETRVRGASTLSQQTAKNLFLWDGRSWLRKGLEAGLTVGIETVWSKK
RILTVYLNIAEFGEGVFGVEAAAQRYFNKPASRLTMSEAALLAAVLPNPIRFKANAPSGYVRSRQAWILRQMRQLGG
EGFMARNQLM

Protein synthesis and translocation:

1. IleS

>Salmonella typhimurium
MSDYKSTLNLPETGFPMRGDLAKREPGMLARWTDDDLYGIIRAAKKGKKTFILHDGPPYANGSIHIGHSVNKILKDI
IVKSKGLSGFDSPYVPGWDCHGLPIELKVEQEFGKPGEKFTAAEFRAKCREYAATQVDGQRKDFIRLGVLGDWSHPY
LTMDFKTEANIIRALGRIIKNGHLHKGAKPVHWCVDCRSALAEAEVEYYDKTSPSIDVAFRAVDQDAVKAKFGLPGV
SGPVSLVIWTTTPWTLPANRAISLAPDFDYALVQIDGQAVILAKDLVESVMQRIGAAEYTILGTVKGAELELLRFTH
PFMGFDVPAILGDHVTLDAGTGAVHTAPGHGPDDYVIGQKYGLETANPVGPDGTYLPGTYPTLDGVNVFKANDIVIE
LLKEKGALLHVEKMQHSYPCCWRHKTPIIFRATPQWFVSMDKEGLRQQSLKEIKGVQWIPDWGQARIESMVANRPDW

CISRQRTWGVPMSLFVHKETQELLPIERTLAAMEEVAKRVEVDGIQAWWDLDPKEILGEDADQYEKVPDTLDVWFDS
GSTSYSVVDARPEFAGHAADMYLEGSDQHRGWFMSSLMISVAMKGKAPYRQVLTHGFTVDGQGRKMSKSIGNTVSPQ
DVMNKLGADILRLWVASTDYTGEMAVSDEILKRAADSYRRIRNTARFLLANLNGFNPATDMVKPEEMVVLDRWAVGC
AKTAQQEILKAYEAYDFHEVVQRLMRFCSVEMGSFYLDIIKDRQYTAKADSVARRSCQTALYHIAEALVRWMAPIMS
FTADEIWGYLPGEREKYVFTGEWYDGLFGLEENEEFNDAFWDDVRYIKDQVNKELENQKANGIKSNLEAKVTLKYAD
DANGTIKKLKLLGEEVRFIFITSQFVISEQAGGIDDENIQYNAGNTTVQAVVTRAEGDKCPRCWHYTTDVGKVAEHA
DICGRCVSNIAGNGEQRKFA

>Escherichia coli
MSDYKSTLNLPETGFPMRGDLAKREPGMLARWTDDDLYGIIRAAKKGKKTFILHDGPPYANGSIHIGHSVNKILKDI
IVKSKGLSGYDSPYVPGWDCHGLPIELKVEQEYGKPGEKFTAAEFRAKCREYAATQVDGQRKDFIRLGVLGDWSHPY
LTMDFKTEANIIRALGKIIGNGHLHKGAKPVHWCVDCRSALAEAEVEYYDKTSPSIDVAFQAVDQDALKAKFAVSNV
NGPISLVIWTTTPWTLPANRAISIAPDFDYALVQIDGQAVILAKDLVESVMQRIGVTDYTILGTVKGAELELLRFTH
PFMGFDVPAILGDHVTLDAGTGAVHTAPGHGPDDYVIGQKYGLETANPVGPDGTYLPGTYPTLDGVNVFKANDIVVA
LLQEKGALLHVEKMQHSYPCCWRHKTPIIFRATPQWFVSMDQKGLRAQSLKEIKGVQWIPDWGQARIESMVANRPDW
CISRQRTWGVPMSLFVHKDTEELHPRTLELMEEVAKRVEVDGIQAWWDLDAKEILGDEADQYVKVPDTLDVWFDSGS
THSSVVDVRPEFAGHAADMYLEGSDQHRGWFMSSLMISTAMKGKAPYRQVLTHGFTVDGQGRKMSKSIGNTVSPQDV
MNKLGADILRLWVASTDYTGEMAVSDEILKRAADSYRRIRNTARFLLANLNGFDPAKDMVKPEEMVVLDRWAVGCAK
AAQEDILKAYEAYDFHEVVQRLMRFCSVEMGSFYLDIIKDRQYTAKADSVARRSCQTALYHIAEALVRWMAPILSFT
ADEVWGYLPGEREKYVFTGEWYEGLFGLADSEAMNDAFWDELLKVRGEVNKVIEQARADKKVGGSLEAAVTLYAEPE
LSAKLTALGDELRFVLLTSGATVADYNDAPADAQQSEVLKGLKVALSKAEGEKCPRCWHYTQDVGKVAEHAEICGRC
VSNVAGDGEKRKFA

>Citrobacter rodentium
MSDYKSTLNLPETGFPMRGDLAKREPGMLARWTDDDLYGIIRAAKKGKKTFILHDGPPYANGSIHIGHSVNKILKDI
IVKSKGLSGFDSPYVPGWDCHGLPIELKVEQEYGKPGEKFTAAEFRAKCREYAATQVDGQRKDFIRLGVLGDWSHPY
LTMDFKTEANIIRALGKIIGNGHLHKGAKPVHWCVDCRSALAEAEVEYYDKTSPSIDVAFRAVDQNAVQAKFGLPGV
SGPISLVIWTTTPWTLPANRAISLAPDFDYALVQIDGQALILAKDLVESVMQRIGVADYSILGTVKGAELELLRFTH
PFMGFDVPAILGDHVTLDAGTGAVHTAGGHGPDDYTISLKYGLEIANPVGPDGAYLAGTYPGLDGVNVFKANDTIVE
LLREKGALLHVEKMQHSYPCCWRHKSPIIFRATPQWFISMDQKGLRAQSLKEIKGVQWIPDWGQARIESMVANRPDW
CISRQRTWGVPMALFVHKDTEELHPRAVELMEEVAKRVEVDGIQAWWDLDPKAILGDEADQYVKVPDTLDVWFDSGS
THASVVDVRPEFAGHAADMYLEGSDQHRGWFMSSLMISTAMKGKAPYRQVLTHGFTVDGQGRKMSKSIGNTVSPQDV
MNKLGADILRLWVASTDYTGEMAVSDEILKRAADSYRRIRNTARFLLANLNGFDPVKDMVKPEEMVVLDRWAVGCAK
AAQEDILKAYEDYDFHEVVQRLMRFCSVEMGSFYLDIIKDRQYTAKADSVARRSCQSALYHIAEALVRWMAPIMSFT
ADEIWGYLPGEREKYVFTGEWYEGLFGLGETEPMNDAFWDELLKVRGEVNKVIEQARADKKVGGSLEAAVTLYAEPE
LAAKLTALGDELRFVLLTSGAKVADYAEASADAQQSELLKGLKVALSKAEGEKCPRCWHYTTDVGKVAEHAEICGRC
VSNVAGDGEKRKFA

>Klebsiella variicola
MSDYKSTLNLPETGFPMRGDLAKREPGMLARWTDDDLYGIIRAAKKGKKTFILHDGPPYANGSIHIGHSVNKILKDI
IVKSKGLTGYDSPYVPGWDCHGLPIELKVEQQYGKPGEKFTAAEFRSKCREYAAEQIDGQRKDFIRLGVLGDWSRPY
LTMDFKTEANIIRALGKIIGNGHLHKGAKPVHWCVDCRSALAEAEVEYYDKTSPSIDVAFHAVDKAAVLAKFGVADV
NGPVSLVIWTTTPWTLPANRAISLSPEFDYALVQVDGQALILAKDLVESVMKRVGATDYTILAAVQGSELELMRFKH
PFLDFDVPAILGDHVTLDAGTGAVHTAGGHGPDDYTISQKYGLEIANPVGPDGAYLPGTYPSLDGINVFKANDIIVE
MLRDSGALLHVEKMQHSYPCCWRHKSPIIFRATPQWFVSMDQKGLRAQSLKEIKGVQWIPDWGQARIESMVANRPDW
CISRQRTWGVPMSLFVHKETHELHPRTLELMEEVAKRVEVDGIQAWWDLDSRDILGDDADSYEKVPDTLDVWFDSGS
THSSVVDVRPEFAGHAADMYLEGSDQHRGWFMSSLMISTAMKGKAPYRQVLTHGFTVDGQGRKMSKSIGNTVSPQDV
MNKLGADILRLWVASTDYTGEMAVSDEILKRAADSYRRIRNTARFLLANLNGFDPAKDMVKPEEMVVLDRWAVGCAQ
AAQEDILKAYESYDFHEVVQRLMRFCSIEMGSFYLDIIKDRQYTAKADSVARRSCQTALFHIVEALVRWMAPIMSFT
ADEIWGYLPGDREKYVFTGEWYKGLFGLADDEAMNDGFWDELLKVRGEVNKVIEQARADKKVGGSLEAAVTLYADAD
LAAKLNALGDELRFVLLTSGANVADYAQAPADAWQSDLLKGLKVVLSKAEGEKCPRCWHYTSDVGKVAEHAEICGRC
VSNVAGNGEQRKFA

>Cronobacter turicensis
MSDYKSTLNLPETGFPMRGDLAKREPGMLARWTDDDLYGIIRAAKKGKKTFILHDGPPYANGSIHIGHSVNKILKDI
IVKSKGLAGYDSPYVPGWDCHGLPIELKVEQEFGKPGEKFTAAEFRAKCREYAATQVDGQRKDFIRLGVLGDWSHPY
LTMDFKTEANIIRALGKIIGNGHLHKGAKPVHWCVDCRSALAEAEVEYYDKTSPSIDVAFNAVDAAAVTAKFGAQTV

NGPVSLVIWTTTPWTLPANRAISLHAEIDYVLVQIEGQALIVAKDLLESVMKRINVADYTVLGETKGAQLELMRFTH
PFMGFDVPAILGEHVTLDAGTGAVHTAGGHGPDDYTISQKYGLEIANPVGPDGAYLPGTYPGLDGVNVFKANDQIVE
LLREKGALLNVAKMVHSYPCCWRHKTPIIFRATPQWFVSMDQKGLRAQSLKEIKGVQWIPDWGQARIESMVANRPDW
CISRQRTWGVPMSLFVHKETQELHPRTLELMEDVAKRVEQDGIQAWWDLDPRDIMGDDADVYEKVPDTLDVWFDSGS
THSSVVDVRPEFSGHAADMYLEGSDQHRGWFMSSLMISTAMKGKAPYRQVLTHGFTVDGQGRKMSKSIGNTVSPQDV
MNKLGADILRLWVASTDYTSEMAVSDEILKRAADSYRRIRNTARFLLANLNGFDPVKDMVKPEEMVVLDRWAVGCAK
AAQDDIVKAYEAYDFHEVVQRLMRFCSIEMGSFYLDIIKDRQYTAKADSVARRSCQTALYHISEALVRWMAPILSFT
ADEVWGYLPGEREKYVFTGEWYDGLFGLADTEAMNDAYWDALLKVRGEVNKVIEQARADKKVGGSLEAAVTLYAEPE
LAAKLTALGEELRFVLLTSQAKVEDYASAAADAQQSELLKGLKVALAKAEGEKCPRCWHYTTDVGKVAEHAEICGRC
VSNVAGDGEKRKFA

>Enterobacter cloacae
MSDYKSTLNLPETGFPMRGDLAKREPGMLARWTDDDLYGIIRAAKKGKKTFILHDGPPYANGSIHIGHSVNKILKDI
IVKSKGLAGYDSPYVPGWDCHGLPIELKVEQEFGKPGEKFTAAEFRAKCREYAATQVDGQRADFIRLGVLGDWSHPY
LTMDFKTEANIIRALGKIIGNGHLHKGAKPVHWCVDCRSALAEAEVEYYDKTSPSIDVAFEAVDQDAIKGKFGLPGV
SGPISLVIWTTTPWTLPANRAISLSGEFEYALVQIDGQALILAKDLVESVLKRANITDYTVLGTVKGDALELMRFKH
PFLDFDVPAILGDHVTLEAGTGAVHTAGGHGPDDYNISLKYGLEIANPVGPDGSYLPGTYPSLDGINVFKANDIIVE
MLRERGALLHVEKMQHSYPCCWRHKTPIIFRATPQWFVSMDQKGLREQSLKEIKGVQWIPDWGQARIESMVANRPDW
CISRQRTWGVPMSLFVHKETHELHPNTLELMEEVAKRVEVDGIQAWWDLDARDILGADADNYEKVPDTLDVWFDSGS
THASVVDVRPEFAGHAADMYLEGSDQHRGWFMSSLMISTAMKGKAPYRQVLTHGFTVDGQGRKMSKSIGNTVSPQDV
MNKLGADILRLWVASTDYTGEMAVSDEILKRAADSYRRIRNTARFLLANLNGFDPAKDMVKPEEMVVLDRWAVGCAK
AAQEDIVKAYESYDFHEVVQRLMRFCSIEMGSFYLDIIKDRQYTAKADSVARRSCQTALYHIAEALVRWMAPIMSFT
ADEIWGYLPGEREKYVFTGEWYEGLFDLSTTEAMNDAFWDELLKVRGEVNKVIEQARADKKVGGSLEAAVTLYAEPE
LAAKLTALGDELRFVLLTSGAKVADYADASADAQQSELLKGLKVALSKAEGEKCPRCWHYTTDVGQVAEHADICGRC
VSNVAGDGEKRKFA

>Phytobacter diazotrophicus
MSDYKSTLNLPETGFPMRGDLAKREPGMLARWTDDDLYGIIRAAKKGKKTFILHDGPPYANGSIHIGHSVNKILKDI
IVKSKGLAGYDSPYVPGWDCHGLPIELKVEQEFGKPGEKFTAAEFRAKCREYAATQIDGQRKDFIRLGVLGDWEHPY
RTMDFKTEANIIRALGKIIGNGHLHKGAKPVHWCVDCRSALAEAEVEYYDKTSPSIDVAFHAVDVDAVKAKFGVASV
NGPVSLVIWTTTPWTLPANRAISLSPEFDYALVQVEGQALILAKDLLESVMKRAGISEFTVLGVVKGSDLELMRFKH
PFLDFDVPAILGDHVTLEAGTGAVHTAGGHGPDDYTISQKYGLEIANPVGPDGAYLPGTYPILDGVNVFKANDIIVN
LLRDNGSLLHVEKLQHSYPCCWRHKSPIIFRATPQWFVSMDQKGLRIQSLKEIDRIEQEGLAKENLSGWIPAWGKAR
IESMVANRPDWCISRQRTWGVPMALFVHKDTEELHPRTTELMEAVAKYVEKDGIQAWWDLDPREILGDDADNYVKVP
DTLDVWFDSGSTSYSVVDARPEFGGHAADMYLEGSDQHRGWFMSSLMISVAMKGKAPYRQVLTHGFTVDGQGRKMSK
SIGNTVSPQDVMNKLGADILRLWVASTDYTGEMAVSDEILKRAADSYRRIRNTARFLLANLNGFDPAKDMVKPEEMV
VLDRWAVGCAQAAQKDIISAYDSYDFHEVVQRLMRFCSIEMGSFYLDIIKDRQYTAKADSVARRSCQTALYHIAEAL
VRWMAPIMSFTADEIWGYLPGEREKYVFTGEWYQGLFGLAETEAMNDAYWEELLKVRGEVNKVIEQARADKKVGGSL
EAAVTLFAEPELSAKLTALGDELRFVLLTSGAKVEDYANACADAQQSELLKGLKVALSKAEGEKCPRCWHYTQDVGK
VAEHAEICGRCVSNVAGDGEKRKFA

>Kosakonia sacchari
MSDYKSTLNLPETGFPMRGDLAKREPGMLARWTDDDLYGIIRAAKKGKKTFILHDGPPYANGSIHIGHSVNKILKDI
IVKSKGLAGFDSPYVPGWDCHGLPIELKVEQEYGKPGEKFTAAEFRAKCREYAATQVDGQRKDFIRLGVLGDWSHPY
LTMDFKTEANIIRALGKIIGNGHLLKGAKPVHWCVDCRSALAEAEVEYYDKTSPSIDVAFQAVDQDAVKTKFGASTV
NGPISLVIWTTTPWTLPANRAISLSPEFDYALVQIDGQALILAKDLVDSVMKRIGVADYSVVGTVKGSELELMRFKH
PFLDFDVPAILGDHVTLEAGTGAVHTAGGHGPDDYTISLKYGLEIANPVGPDGTYLPGTYPTLDGVNVFKANDLIVA
LLTEKGALLHVEKMQHSYPCCWRHKSPIIFRATPQWFVSMDQKGLRAQSLSEIKGVQWIPDWGQARIESMVANRPDW
CISRQRTWGVPMSLFVHKDTEELHPRTLELIEAVAKRVEVDGIQAWWDLDPRDIMGDDADNYVKVPDTLDVWFDSGS
THASVVDVRPEFAGHAADMYLEGSDQHRGWFMSSLMISTAMKGKAPYRQVLTHGFTVDGQGRKMSKSIGNTVSPQEV
MNKLGADILRLWVASTDYTGEMAVSDEILKRAADSYRRIRNTARFLLANLNGFDPVKDMVKPEEMVVLDRWAVGCAK
AAQEDIVKAYESYDFHEVVQRLMRFCSIEMGSFYLDIIKDRQYTAKADSVARRSCQTALYHICEALVRWMAPIMSFT
ADEIWGYLPGSREKYVFTGEWYEGLFSLADSEAMNDAFWDELLKVRGEVNKVIEQARADKKVGGSLEAAVTLFAEPE
LAAKLTALGTELRFVLLTSGAKVADYASASADAQQSELLKGLKVALSKAEGEKCPRCWHYTTDVGQVAEHADICGRC
VSNVAGDGEKRKFA

>Huaxiibacter chinensis
MSDYKSTLNLPETGFPMRGDLAKREPGMLARWTDDDLYGIIRAAKKGKKTFILHDGPPYANGSIHIGHSVNKILKDI
IVKSKGLAGFDSPYVPGWDCHGLPIELKVEQEYGKPGEKFTAAEFRAKCREYAAAQVDGQRKDFIRLGVLGDWSRPY
LTMDFKTEANIIRALGKIIGNGHLHKGAKPVHWCVDCRSALAEAEVEYYDKTSPSIDVAFHAVDQDAVKAKFGVSSV
NGPVSLVIWTTTPWTLPANRAISLSADFEYALVQIDGQAVILAKDLVESVLKRAHIDNFTVLGTVNGAELELMRFKH
PFLDFDVPAILGDHVTLEAGTGAVHTAGGHGPDDYNISLKYGLEIANPVGPDGAYLPGTYPALDGINVFKANDIIVD
ILRTRGALLHVEKMQHSYPCCWRHKTPIIFRATPQWFISMDQKGLRAQSLKEIKGVQWIPDWGQARIESMVANRPDW
CISRQRTWGVPMSLFVHKETQELHPNTLELMEEVAKRVEVDGIQAWWDLDSRDILGADADSYEKVPDTLDVWFDSGS
THSSVVDVRPEFAGHAADMYLEGSDQHRGWFMSSLMISTAMKGKAPYRQVLTHGFTVDGQGRKMSKSIGNTVSPQDV
MNKLGADILRLWVASTDYTGEMAVSDEILKRAADSYRRIRNTARFLLANLNGFDPVNDMVKPEEMVVLDRWAVGCAQ
AAQDDILKAYESYDFHEVVQRLMRFCSIEMGSFYLDIIKDRQYTAKADSVARRSCQTALYHIAEALVRWMAPIMSFT
ADEIWGYLPGEREQYVFTGEWYEGLFGLADTEAMNDTFWDDLLKVRGEVNKVIEQARADKKVGGSLEAAVTLYAEPE
LAAKLTALGDELRFVLLTSGAQVADYAQASADAQQSELLKGLKVALSKADGEKCPRCWHYTTDIGQVAEHADICGRC
VSNIAGDGEKRKFA

2. EngA

>Salmonella typhimurium
MVPVVALVGRPNVGKSTLFNRLTRTRDALVADFPGLTRDRKYGRAEVEGREFICIDTGGIDGTEDGVETRMAEQSLL
AIEEADVVLFMVDARAGLMPADEAIAKHLRSREKPTFLVANKTDGLDPDQAVVDFYSLGLGEIYPIAASHGRGVLSL
LEHVLLPWMDDVAPQEEVDEDAEYWAQFEAEQNGEEAPEDDFDPQSLPIKLAIVGRPNVGKSTLTNRILGEERVVVY
DMPGTTRDSIYIPMERDEREYVLIDTAGVRKRGKITDAVEKFSVIKTLQAIEDANVVLLVIDAREGISDQDLSLLGF
ILNSGRSLVIVVNKWDGLSQEVKEQVKETLDFRLGFIDFARVHFISALHGSGVGNLFESVREAYDSSTRRVSTAMLT
RIMTMAVEDHQPPLVRGRRVKLKYAHAGGYNPPIVVIHGNQVKDLPDSYKRYLMNYFRKSLEVMGTPIRIQFKEGEN
PYANKRNTLTPTQMRKRKRLMKHIKKSK

>Escherichia coli
MVPVVALVGRPNVGKSTLFNRLTRTRDALVADFPGLTRDRKYGRAEIEGREFICIDTGGIDGTEDGVETRMAEQSLL
AIEEADVVLFMVDARAGLMPADEAIAKHLRSREKPTFLVANKTDGLDPDQAVVDFYSLGLGEIYPIAASHGRGVLSL
LEHVLLPWMEDLAPQEEVDEDAEYWAQFEAEENGEEEEEDDFDPQSLPIKLAIVGRPNVGKSTLTNRILGEERVVVY
DMPGTTRDSIYIPMERDGREYVLIDTAGVRKRGKITDAVEKFSVIKTLQAIEDANVVMLVIDAREGISDQDLSLLGF
ILNSGRSLVIVVNKWDGLSQEVKEQVKETLDFRLGFIDFARVHFISALHGSGVGNLFESVREAYDSSTRRVGTSMLT
RIMTMAVEDHQPPLVRGRRVKLKYAHAGGYNPPIVVIHGNQVKDLPDSYKRYLMNYFRKSLDVMGSPIRIQFKEGEN
PYANKRNTLTPTQMRKRKRLMKHIKKNK

>Citrobacter rodentium
MVPVVALVGRPNVGKSTLFNRLTRTRDALVADFPGLTRDRKYGRAEVEGREFICIDTGGIDGTEEGVETRMAEQSLL
AIEEADVVLFMVDARAGLMPADEAIARHLRSREKPTFLVANKTDGLDPDQAVVDFYSLGLGEIHPIAASHGRGVLSL
LEHVLLPWMDDVAPQEEVDEDAEYWAQFEADENGEEEPEDDFNPQDLPIKLAIVGRPNVGKSTLTNRILGEDRVVVY
DMPGTTRDSIYIPMERDEREYVLIDTAGVRKRGKITDAVEKFSVIKTLQAIEDANVVLLVIDAREGISDQDLSLLGF
ILNSGRSLVIVVNKWDGLSQEVKEQVKETLDFRLGFIDFARVHFISALHGSGVGNLFESVREAYDSSTRRVSTAMLT
RIMTMAVEDHQPPLVRGRRVKLKYAHAGGYNPPIVVIHGNQVKDLPDSYKRYLMNYFRKSLEVMGTPIRIQFKEGEN
PYANKRNTLTPTQMRKRKRLIKHIKKSK

>Klebsiella variicola
MIPVVALVGRPNVGKSTLFNRLTRTRDALVADFPGLTRDRKYGRAEVEGREFICIDTGGIDGTEEGVETRMAEQSLL
AIEEADVVLFMVDARAGLMPADIAIAKHLRSREKPTFLVANKTDGIDVDQAMADFWSLGLGDIYPIAASHGRGVTSL
LEQALLPWVDEISPQEEVDEDAEYWAKFEAEQNGEAEEEPEDDFNPQDLPIKLAIVGRPNVGKSTLTNRILGEDRVV
VYDMPGTTRDSIYIPMQRDEREYVLIDTAGVRKRGKITDVVEKFSVIKTLQAIEDANVVLLVIDAREGISDQDLSLL
GFILNSGRSLVIVVNKWDGLSQEVKEQVKETLDYRLGFIDFARVHFISALHGSGVGNLFESVREAYDSATRRVSTAM
LTRIMNMAAEDHQPPLVRGRRVKLKYAHAGGYNPPIVVIHGNQVKDLPDSYKRYLMNYFRKSLDVMGTPIRIQFKEG
ENPFANKRNTLTPNQMRKRKRLIKHIKKSK

>Cronobacter turicensis
MIPVVALVGRPNVGKSTLFNRLTRTRDALVADFPGLTRDRKYGRAEVEGREFICIDTGGIDGAEEGVETRMAEQSLL
AIEEADVVLFMVDARAGLMPADEAIAKHLRSRQKPTFLVANKTDGLDPDQAVADFYSLGLGEIHPIAASHGRGVTSL
LEHVLVPWMDDVDPPEEVDEEAEYWAQFEAEQNGEVVEEEEDDFNPQDLPIKLAIVGRPNVGKSTLTNRILGEDRVV

VFDMPGTTRDSIYIPMERDGREFVLIDTAGVRKRGKITDVVEKFSVIKTLQAIEDANVVLLVIDAREGISDQDLSLL
GFILNSGRSLVIVVNKWDGLSQEVKDEVKETLDYRLGFIDFARVHFISALHGSGVGNLFESVREAYDSSTRRVSTAL
LTRIMKMAEEDHQPPMVRGRRVKLKYAHAGGYNPPIVVIHGSQVKDLPDSYKRYLMNYFRKSLDVMGTPIRIQFKEG
ANPFANKRNTLTPNQLRKRKRLIKHIKKSK

>Enterobacter cloacae
MVPVVALVGRPNVGKSTLFNRLTRTRDALVADFPGLTRDRKYGRAEVEGREFICIDTGGIDGTEDGVETRMAEQSLL
AIEEADVVLFMVDARAGLMPADSAIAKHLRSREKPTFLVANKTDGIDADQAIADFWSLGLGDIYPIAASHGRGVTSL
LETVLLPWVDEVNPPEEVDEDAAYWAQFEEGEEGEEEEPEETFNPQDLPIKLAIVGRPNVGKSTLTNRILGEERVVV
YDMPGTTRDSIYIPMERDEREYVLIDTAGVRKRGKITDVVEKFSVIKTLQAIEDANVVLLVIDAREGISDQDLSLLG
FILNSGRSLVIVVNKWDGLSNEVREQVKETLDFRLGFIDFARVHFISALHGSGVGNLFDSVREAYDSSTRRQSTAML
TRIMNMAAEDHQPPLVRGRRVKLKYAHAGGYNPPIVVIHGNQVKDLPDSYKRYLMNYFRKSLDVMGTPIRIQFKEGD
NPFANKRNTLTPNQMRKRKRLIKHIKKSK

>Phytobacter diazotrophicus
MIPVVALVGRPNVGKSTLFNRLTRTRDALVADFPGLTRDRKYGRAEVEGREFICIDTGGIDGTEDGVETRMAEQSLL
AIEEADVVLFMVDARAGLMPADEAIAKHLRSREKPTFLVANKTDGLDPDQAVVDFYSLGLGEIHPIAASHGRGVVSL
LEHVLLPWMDEIDPPEELDEDAAYWAQFDEGKGEDGEEEPEDDFNPQDLPIKLAIVGRPNVGKSTLTNRILGEERVV
VYDMPGTTRDSIYIPMERDGREYVLIDTAGVRKRGKITDAVEKFSVIKTLQAIEDANVVMLVIDAREGISDQDLSLL
GFILNSGRSLVIVVNKWDGLSQEVKEQVKETLDFRLGFIDFARVHFISALHGSGVGNLFESVREAYDSSTRRQSTAM
LTRIMNMASEDHQPPLVRGRRVKLKYAHAGGYNPPIVVIHGNQVKDLPDSYKRYLMNYFRKSLDVMGTPIRIQFKEG
ENPYADKRNTLTPNQMRKRKRLIKHIKKSK

>Kosakonia sacchari
MVPVVALVGRPNVGKSTLFNRLTRTRDALVADFPGLTRDRKYGRAEVEGREFICIDTGGIDGTEDGVETRMAEQSLL
AIEEADVVLFMVDARAGLMPADSAIAKHLRSREKPTFLVANKTDGLDPDQAIADFYSLGLGEIHAIAASHGRGVTSL
LEHVLMPWMDELNPPEVVDEEAEYWAQFEANENGEKEEPEDDFNPQDLPIKLAIVGRPNVGKSTLTNRILGEDRVVV
YDMPGTTRDSIYIPMQRDEREYVLIDTAGVRKRGKITDVVEKFSVIKTLQAIEDANVVLLVIDAREGISDQDLSLLG
FILNSGRSLVIVVNKWDGLSQEVKEQVKETLDYRLGFIDFARVHFISALHGSGVGNLFESVREAYDSSTRRVSTALL
TRIMNMAAEDHQPPLVRGRRVKLKYAHAGGYNPPIVVIHGNQVKDLPDSYKRYLMNYFRKSLDVMGTPIRIQFKEGE
NPFAEKRNTLTPNQMRKRKRLIKHIKKGK

>Huaxiibacter Chinensis
MVPVVALVGRPNVGKSTLFNRLTRTRDALVADFPGLTRDRKYGRAEVEGREFICIDTGGIDGTEDGVETRMAEQSLL
AIEEADVVLFMVDARAGLMPADSAIAKHLRSREKPTFLVANKTDGIDADQAVADFWSLGLGDIYPIAASHGRGVTSL
LETVLLPWVDEVNPQEEVDEDAAYWAQFEEGVEGAEEPEDDFNPQDLPIKLAIVGRPNVGKSTLTNRILGEERVVVY
DMPGTTRDSIYIPMQRDEREYVLIDTAGVRKRGKITDVVEKFSVIKTLQAIEDANVVMLVIDAREGISDQDLSLLGF
ILNSGRSLVIVVNKWDGLSNDVREQVKEMLDFRLGFIDFARVHFISALHGSGVGNLFESVREAYDSSTRRQSTAMLT
RIMTMAAEDHQPPLVRGRRVKLKYAHAGGYNPPIVVIHGNQVKDLPDSYKRYLMNYFRKSLDVMGTPIRIQFKEGEN
PFANKRNTLTPNQMRKRKRLIKHIKKSK

3. SecY

>Salmonella typhimurium
MAKQPGLDFQSAKGGLGELKRRLLFVIGALIVFRIGSFIPIPGIDAAVLAKLLEQQRGTIIEMFNMFSGGALSRASI
FALGIMPYISASIIIQLLTVVHPTLAEIKKEGESGRRKISQYTRYGTLVLAIFQSIGIATGLPNMPGMQGLVMNPGF
AFYFTAVVSLVTGTMFLMWLGEQITERGIGNGISIIIFAGIVAGLPPAIAHTIEQARQGDLHFLVLLLVAVLVFAVT
FFVVFVERGQRRIVVNYAKRQQGRRVYAAQSTHLPLKVNMAGVIPAIFASSIILFPATIASWFGGGTGWNWLTTISL
YLQPGQPLYVLLYASAIIFFCFFYTALVFNPRETADNLKKSGAFVPGIRPGEQTAKYIDKVMTRLTLVGALYITFIC
LIPEFMRDAMKVPFYFGGTSLLIVVVVIMDFMAQVQTLMMSSQYESALKKANLKGYGR

>Escherichia coli
MAKQPGLDFQSAKGGLGELKRRLLFVIGALIVFRIGSFIPIPGIDAAVLAKLLEQQRGTIIEMFNMFSGGALSRASI
FALGIMPYISASIIIQLLTVVHPTLAEIKKEGESGRRKISQYTRYGTLVLAIFQSIGIATGLPNMPGMQGLVINPGF
AFYFTAVVSLVTGTMFLMWLGEQITERGIGNGISIIIFAGIVAGLPPAIAHTIEQARQGDLHFLVLLLVAVLVFAVT
FFVVFVERGQRRIVVNYAKRQQGRRVYAAQSTHLPLKVNMAGVIPAIFASSIILFPATIASWFGGGTGWNWLTTISL

YLQPGQPLYVLLYASAIIFFCFFYTALVFNPRETADNLKKSGAFVPGIRPGEQTAKYIDKVMTRLTLVGALYITFIC
LIPEFMRDAMKVPFYFGGTSLLIVVVVIMDFMAQVQTLMMSSQYESALKKANLKGYGR

>Citrobacter rodentium
MAKQPGLDFQSAKGGLGELKRRLLFVIGALIVFRIGSFIPIPGIDAAVLAKLLEQQRGTIIEMFNMFSGGALSRASI
FALGIMPYISASIIIQLLTVVHPTLAEIKKEGESGRRKISQYTRYGTLVLAIFQSIGIATGLPNMPGMQGLVMNPGF
AFYFTAVVSLVTGTMFLMWLGEQITERGIGNGISIIIFAGIVAGLPPAIAHTIEQARQGDLHFLVLLLVAVLVFAVT
FFVVFVERGQRRIVVNYAKRQQGRRVYAAQSTHLPLKVNMAGVIPAIFASSIILFPATIASWFGGGTGWNWLTTISL
YLQPGQPLYVLLYASAIIFFCFFYTALVFNPRETADNLKKSGAFVPGIRPGEQTAKYIDKVMTRLTLVGALYITFIC
LIPEFMRDAMKVPFYFGGTSLLIVVVVIMDFMAQVQTLMMSSQYESALKKANLKGYGR

>Klebsiella variicola
MAKQPGLDFQSAKGGLGELKRRLLFVVGALIVFRIGSFIPIPGIDAAVLAKLLEQQRGTIIEMFNMFSGGALSRASI
FALGIMPYISASIIVQLLTVVYQPLAELKKEGESGRRKISQYTRYGTLVLAIFQSIGIATGLPNMPGMQGLVINPGF
AFYFTAVVSLVTGTMFLMWLGEQITERGIGNGISILIFAGIVAGLPPAIAHTIEQARQGDLHFLLLLLVAVLVFAVT
FFVVFVERGQRRIVVNYAKRQQGRRVYAAQSTHLPLKVNMAGVIPAIFASSIILFPATITSWFGGGTGWNWLTTISL
YLQPGQPLYVLLYASAIIFFCFFYTALVFNPRETADNLKKSGAFVPGIRPGEQTAKYIDKVMTRLTLVGALYITFIC
LIPEFMRDAMKVPFYFGGTSLLIVVVVIMDFMAQVQTLMMSSQYESALKKANLKGYGR

>Cronobacter turicensis
MAKQPGLDFQSAKGGLGELKRRLLFVIGALIVFRIGSFIPIPGIDAAVLAKLLEQQRGTIIEMFNMFSGGALSRASI
FALGIMPYISASIIIQLLTVVHPTLAEMKKEGESGRRKISQYTRYGTLVLAIFQSIGIATGLPNMPGMQGLVINPGF
AFYFTAVVSLVTGTMFLMWLGEQITERGIGNGISIIIFAGIVAGLPPAIGHTIEQARQGDLHFLLLLLVAVLVFAVT
FFVVFVERGQRRIVVNYAKRQQGRRVYAAQSTHLPLKVNMAGVIPAIFASSIILFPATIASWFGGGTGWNWLTTISL
YLQPGQPLYVLLYASAIIFFCFFYTALVFNPRETADNLKKSGAFVPGIRPGEQTAKYIDKVMTRLTLVGALYITFIC
LIPEFMRDAMKVPFYFGGTSLLIVVVVIMDFMAQVQTLMMSSQYESALKKANLKGYGR

>Enterobacter cloacae
MAKQPGLDFQSAKGGFGELKRRLLFVIGALIVFRIGSFIPIPGIDAAVLAKLLEQQRGTIIEMFNMFSGGALSRASI
FALGIMPYISASIIIQLLTVVHPALAELKKEGESGRRKISQYTRYGTLVLAIFQSIGIATGLPNMPGMQGLVLNPGF
AFYFTAVVSLVTGTMFLMWLGEQITERGIGNGISIIIFAGIVAGLPPAIAHTIEQARQGDLHFLLLLLVAVLVFAVT
FFVVFVERGQRRIVVNYAKRQQGRRVYAAQSTHLPLKVNMAGVIPAIFASSIILFPATIASWFGGGTGWNWLTTISL
YLQPGQPLYVLLYASAIIFFCFFYTALVFNPRETADNLKKSGAFVPGIRPGEQTAKYIDKVMTRLTLVGALYITFIC
LIPEFMRDAMKVPFYFGGTSLLIVVVVIMDFMAQVQTLMMSSQYESALKKANLKGYGR

>Phytobacter diazotrophicus
MAKQPGLDFQSAKGGLGELKRRLLFVIGALIVFRIGSFIPIPGIDAAVLAKLLEQQRGTIIEMFNMFSGGALSRASI
FALGIMPYISASIIIQLLTVVHPALAELKKEGESGRRKISQYTRYGTLVLAIFQATGIATGLPNMPGMQGLVINPGF
AFYFTAVVSLVTGTMFLMWLGEQITERGIGNGISIIIFAGIVAGLPPAIAHTIEQARQGDLHFLLLLLVAVLVFAVT
FFVVFVERGQRRIVVNYAKRQQGRRVYAAQSTHLPLKVNMAGVIPAIFASSIILFPATIASWFGGGTGWNWLTTISL
YLQPGQPLYVLLYASAIIFFCFFYTALVFNPRETADNLKKSGAFVPGIRPGEQTAKYIDKVMTRLTLIGALYITFIC
LIPEFMRDAMKVPFYFGGTSLLIVVVVIMDFMAQVQTLMMSSQYESALKKANLKGYGR

>Kosakonia sacchari
MAKQPGLDFQSAKGGIGELKRRLLFVIGALIVFRIGSFIPIPGIDAAVLAKLLEQQRGTIIEMFNMFSGGALSRASI
FALGIMPYISASIIIQLLTVVHPALAELKKEGESGRRKISQYTRYGTLVLAIFQSIGIATGLPNMPGMQGLVMNPGF
AFYFTAVVSLVTGTMFLMWLGEQITERGIGNGISIIIFAGIVAGLPPAIAHTIEQARQGDLHFLLLLLVAVLVFAVT
FFVVFVERGQRRIVVNYAKRQQGRRVYAAQSTHLPLKVNMAGVIPAIFASSIILFPATIASWFGGGTGWNWLTTISL
YLQPGQPLYVLLYASAIIFFCFFYTALVFNPRETADNLKKSGAFVPGIRPGEQTAKYIDKVMTRLTLVGALYITFIC
LIPEFMRDAMKVPFYFGGTSLLIVVVVIMDFMAQVQTLMMSSQYESALKKANLKGYGR

>Huaxiibacter chinensis
MAKQPGLDFQSAKGGFGELKRRLLFVVGALIVFRIGSFIPIPGIDAAVLAKLLEQQRGTIIEMFNMFSGGALSRASI
FALGIMPYISASIIIQLLTVVHPALAELKKEGESGRRKISQYTRYGTLVLAIFQSIGIATGLPNMPGMQGLVINPGF
AFYFTAVVSLVTGTMFLMWLGEQITERGIGNGISIIIFAGIVAGLPPAIAHTIEQARQGDLHFLLLLLVAVLVFAVT
FFVVFVERGQRRIVVNYAKRQQGRRVYAAQSTHLPLKVNMAGVIPAIFASSIILFPATIASWFGGGTGWNWLTTISL

YLQPGQPLYVLLYASAIIFFCFFYTALVFNPRETADNLKKSGAFVPGIRPGEQTAKYIDKVMTRLTLVGALYITFIC
LIPEFMRDAMKVPFYFGGTSLLIVVVVIMDFMAQVQTLMMSSQYESALKKANLKGYGR

4. RbfA

>Salmonella typhimurium
MAKEFGRPQRVAQEMQKEIAIILQREIKDPRLGMMTTVSGVEMSRDLAYAKVFVTFLNDKDEDAVKAGIKALQEASG
FIRSLLGKAMRLRIVPELTFFYDNSLVEGMRMSNLVTNVVKHDEERRVNPDDSKED

>Escherichia coli
MAKEFGRPQRVAQEMQKEIALILQREIKDPRLGMMTTVSGVEMSRDLAYAKVYVTFLNDKDEDAVKAGIKALQEASG
FIRSLLGKAMRLRIVPELTFFYDNSLVEGMRMSNLVTSVVKHDEERRVNPDDSKED

>Citrobacter rodentium
MAKEFGRPQRVAQEMQKEIAIILQREIKDPRLGMMTTVSGVEMSRDLAYAKVYVTFLNDKDEDAVKAGIKALQEASG
FIRSLLGKAMRLRIVPELTFFYDNSLVEGMRMSNLVTNVVKHDEERRVNPDDSKED

>Klebsiella variicola
MAKEFGRPQRVAQEMQKEIAIILQREIKDPRLGMMTTVSGVEMSRDLAYAKVYVTFLNDKDEAAVKAGIKALQEASG
FIRSLLGKAMRLRIVPELTFFYDNSLVEGMRMSNLVTSVVKHDDERRVNPDDSKED

>Cronobacter turicensis
MAKEFGRPQRVAQEMQKEIAIILQREIKDPRVGLMTTVSGVEVSRDLAYAKVFVTFLNDKDDAAVKAGIKALQDASG
FIRSLLGKAMRLRIVPELTFFYDNSLVEGMRMSNLVTSVVKHDEERRVNPDDDKEE

>Enterobacter cloacae
MAKEFGRPQRVAQEMQKEIALILQREIKDPRVGMMTTVSGVEMSRDLAYAKVFVTFLNDQDEAAVKNGIKALQEASG
FIRSLLGKAMRLRIVPELTFFYDNSLVEGMRMSNLVTSVVKHDDERRVNPADDSKED

>Phytobacter diazotrophicus
MAKEFGRPQRVAQEMQKEIAIILQREIKDPRVGMMTTVSGVEVSRDLAYAKVFVTFLNDKDEAAVKAGIKALQEASG
FIRTLLGKAMRLRIVPELTFFYDNSLVEGMRMSNLVTSVVKHDDERRVNPTDDSKED

>Kosakonia sacchari
MAKEFGRPQRVAQELQKEIAIILQREIKDPRLGMMTTVSGVEVSRDLAYAKVFVTFLNDKDEASVKAGIKALQDASG
FIRSLLGKAMRLRIVPELTFFYDNSLVEGMRMSNLVTSVVKHDDERRVNPDDSKED

>Huaxiibacter chinensis
MAKEFGRPQRVGQEMQKEIALILQREIKDPRLGMMTTVSGVEMSRDLAYAKVFVTFLNDQDEAAVKNGIKALQEASG
FIRSLLGKAMRLRIVPELTFFYDNSLVEGMRMSNLVTSVVKHDDERRVNPADDSKED

5. InfB

>Salmonella typhimurium
MTDVTLKALAAERQVSVDRLVQQFADAGIRKSADDSVSAQEKQTLLAHLNREAVSGPDKLTLQRKTRSTLNIPGTGG
KSKSVQIEVRKKRTFVKRDPQEAERLAAEEQAQREAEEQARREAEEQAKREAQQKAEREAAEQAKREAAEKAKREAA
EKDKVSNQQTDDMTKTAQAEKARRENEAAELKRKAEEEARRKLEEEARRVAEEARRMAEENKWTATPEPVEDTSDYH
VTTSQHARQAEDENDREVEGGRGRGRNAKAARPAKKGKHAESKADREEARAAVRGGKGGKRKGSSLQQGFQKPAQAV
NRDVVIGETITVGELANKMAVKGSQVIKAMMKLGAMATINQVIDQETAQLVAEEMGHKVILRRENELEEAVMSDRDT
GAAAEPRAPVVTIMGHVDHGKTSLLDYIRSTKVASGEAGGITQHIGAYHVETDNGMITFLDTPGHAAFTSMRARGAQ
ATDIVVLVVAADDGVMPQTIEAIQHAKAAGVPVVVAVNKIDKPEADPDRVKNELSQYGILPEEWGGESQFVHVSAKA
GTGIDELLDAILLQAEVLELKAVRKGMASGAVIESFLDKGRGPVATVLVREGTLHKGDIVLCGFEYGRVRAMRNELG
QEVLEAGPSIPVEILGLSGVPAAGDEVTVVRDEKKAREVALYRQGKFREVKLARQQKSKLENMFANMTEGEVHEVNI
VLKADVQGSVEAISDSLLKLSTDEVKVKIIGSGVGGITETDATLAAASNAILVGFNVRADASARKVIESESLDLRYY
SVIYNLIDEVKAAMSGMLSPELKQQIIGLAEVRDVFKSPKFGAIAGCMVTEGTIKRHNPIRVLRDNVVIYEGELESL
RRFKDDVNEVRNGMECGIGVKNYNDVRVGDMIEVFEIIEIQRTIA

>Escherichia coli
MTDVTIKTLAAERQTSVERLVQQFADAGIRKSADDSVSAQEKQTLIDHLNQKNSGPDKLTLQRKTRSTLNIPGTGGK
SKSVQIEVRKKRTFVKRDPQEAERLAAEEQAQREAEEQARREAEESAKREAQQKAEREAAEQAKREAAEQAKREAAE
KDKVSNQQDDMTKNAQAEKARREQEAAELKRKAEEEARRKLEEEARRVAEEARRMAEENKWTDNAEPTEDSSDYHVT
TSQHARQAEDESDREVEGGRGRGRNAKAARPKKGNKHAESKADREEARAAVRGGKGGKRKGSSLQQGFQKPAQAVNR
DVVIGETITVGELANKMAVKGSQVIKAMMKLGAMATINQVIDQETAQLVAEEMGHKVILRRENELEEAVMSDRDTGA
AAEPRAPVVTIMGHVDHGKTSLLDYIRSTKVASGEAGGITQHIGAYHVETENGMITFLDTPGHAAFTSMRARGAQAT
DIVVLVVAADDGVMPQTIEAIQHAKAAQVPVVVAVNKIDKPEADPDRVKNELSQYGILPEEWGGESQFVHVSAKAGT
GIDELLDAILLQAEVLELKAVRKGMASGAVIESFLDKGRGPVATVLVREGTLHKGDIVLCGFEYGRVRAMRNELGQE
VLEAGPSIPVEILGLSGVPAAGDEVTVVRDEKKAREVALYRQGKFREVKLARQQKSKLENMFANMTEGEVHEVNIVL
KADVQGSVEAISDSLLKLSTDEVKVKIIGSGVGGITETDATLAAASNAILVGFNVRADASARKVIEAESLDLRYYSV
IYNLIDEVKAAMSGMLSPELKQQIIGLAEVRDVFKSPKFGAIAGCMVTEGVVKRHNPIRVLRDNVVIYEGELESLRR
FKDDVNEVRNGMECGIGVKNYNDVRTGDVIEVFEIIEIQRTIA

>Citrobacter rodentium
MTDVTVKALAAEIQTSVDRLVQQFADAGIPKSADDSVSAQEKQTLLAHLNRENGSGPDKLTLQRKTRSTLNIPGTGG
KSKSVQIEVRKKRTFVKRDPQEAERLAAEEQAQREAEEQARREAEEAAKREAQQKADREAAEQAKREAAEKAKREAA
EKDKVSNQQTDDMTKTAQAEKVRRENEAAELKRKAEEEARRKLEEEARRVAEEARRMAEENKWTDTAEPTEDSSDYH
VTTSQHARQAEDENDREVEGGRSRSRSTKAARPAKKGNKHAESKADREEARAAVRGGKGGKQRKGSALQQSFQKPAQ
AVNRDVVIGETITVGELANKMAVKGSQVIKAMMKLGAMATINQVIDQETAQLVAEEMGHKVILRRENELEEAVMSDR
DTGAAAEPRAPVVTIMGHVDHGKTSLLDYIRSTKVASGEAGGITQHIGAYHVETDNGMITFLDTPGHAAFTSMRARG
AQATDIVVLVVAADDGVMPQTIEAIQHAKAAGVPVVVAVNKIDKPEADPDRVKNELSQYGILPEEWGGESQFVHVSA
KAGTGIDELLDAILLQAEVLELKAVRKGMASGAVIESFLDKGRGPVATVLVREGTLNKGDIVLCGFEYGRVRAMRNE
LGQEVLEAGPSIPVEILGLSGVPAAGDEVTVVRDEKKAREVALYRQGKFREVKLARQQKSKLENMFANMTEGEVHEV
NIVLKADVQGSVEAISDSLLKLSTDEVKVKIIGSGVGGITETDATLAAASNAILVGFNVRADASARKVIEAENLDLR
YYSVIYNLIDEVKAAMSGMLSPELKQQIIGLAEVRDVFKSPKFGAIAGCMVTEGTIKRHNPIRVLRDNVVIYEGELE
SLRRFKDDVNEVRNGMECGIGVKNYNDVRVGDMIEVFEIIEIQRTIA

>Klebsiella variicola
MTDVTIKALASEIQTSVDRLIQQFADAGIRKSADDSVTAQEKQTLLTHLNREHGSAPDKLTLQRKTRSTLNIPGTGG
KSKSVQIEVRKKRTFVKRDPQEAERLAAEEQAQREAEEQARREAEEAAKREAQLKAEREAAEQAKREVADKAKREAA
EKDKVSNQHTDEMTKTAQAEKIRRENEAAELKRKSEEEARRKLEEEARRVAEEARRMAEENEKNWSETSDSPEDSSD
YHVTTSQHARQAEDDNDREVEGGRGRSRSSKAARPAKKGNKHAESKADREEARAAVRGGKGGKHRKGSALQQGFQKP
AQAVNRDVIIGETITVGELANKMAVKGSQVIKAMMKLGAMATINQVIDQETAQLVAEEMGHKVILRRENELEEAVMS
DRDTGAAAEPRAPVVTIMGHVDHGKTSLLDYIRSTKVASGEAGGITQHIGAYHVETDNGMITFLDTPGHAAFTSMRA
RGAQATDIVVLVVAADDGVMPQTIEAIQHAKAAQVPVVVAVNKIDKPEADPDRVKNELSQYGILPEEWGGESQFVHV
SAKAGTGIDDLLDAILLQAEVLELKAVRNGMASGAVIESFLDKGRGPVATVLVREGTLHKGDIVLCGFEYGRVRAMR
DELGREVLEAGPSIPVEILGLSGVPAAGDEVTVVRDEKKAREVALYRQGKFREVKLARQQKSKLENMFANMTEGEVH
EVNIVLKADVQGSVEAISDSLLKLSTDEVKVKIIGSGVGGITETDATLAAASNAILVGFNVRADASARKVIEAESLD
LRYYSVIYNLIDEVKAAMSGMLSPELKQQIIGLAEVRDVFKSPKFGAIAGCMVTEGTIKRHNPIRVLRDNVVIYEGE
LESLRRFKDDVNEVRNGMECGIGVKNYNDVRVGDMIEVFEIIEIQRSID

>Cronobacter turicensis
MTDVTVKALAAEIQTSVDRLVQQFADAGIPKSAEDSVTAQEKQALLAHLNREHGSGPDKLTLQRKTRSTLNIQGTGG
KSKSVQIEVRKKRTFVKRDPQEAERLAAEEQAKREAEEQARREAEEAAKREAEEKAKREAGDKAKREAEEQAKRDAA
DKAKREAAETSKVSNQQTDEVSKAAQAEKARREAEALDLKRKAEEEARRKLEENARRVAEEARRMAEENATKWESGS
EEESEDSSDYHVTTSQHARQAEDDSDREVEGGRSRARPAKAARQKKSNKHSESKADREEARAAVRGGKGGKRKGSSL
QQGFNKPAQAVNRDVVIGETITVGELANKMAVKGSQVIKAMMKLGAMATINQVIDQETAQLVAEEMGHKVILRRENE
LEEAVMSDRDMGAQAEPRAPVVTIMGHVDHGKTSLLDYIRSTKVASGEAGGITQHIGAYHVQTDNGMITFLDTPGHA
AFTAMRARGAQATDIVVLVVAADDGVMPQTIEAIQHAKAAKVPVVVAVNKIDKPDADPDRVKNELSQHGIIPEEWGG
ESQFVHVSAKAGTGIDELLDAILLQSEVLELHAVRKGMATGVVIESFLDKGRGPVATVLVREGTLNKGDIVLCGFEY
GRVRAMRNELNQEVQEAGPSIPVEILGLSGVPAAGDEVTVVRDEKKAREVALYRQGKFREVKLARQQKSKLENMFAN
MTEGEVHEVNIVLKADVQGSVEAISDSLLKLSTDEVKVKIVGSGVGGITETDATLAAASNAILVGFNVRADASARRV
IEAESLDLRYYSVIYNLIDEVKAAMSGMLSPELKQQIIGLAEVRDVFKSPKFGAIAGCMVTEGTIKRHNPIRVLRDN
VVIYEGELESLRRFKDDVNEVRNGMECGIGVKNYNDVRVGDMIEVFEIIEIQRSID

>Enterobacter cloacae

MTDVTVKSLAAEIQTSVDRLVQQFADAGIPKTADDSVTANEKQTLLAHLNREHGSAPDKLTLQRKTRSTLNIPGTGG
KSKSVQIEVRKTRTFVKRDPQEAERLAAEEQAQREAEEQAQREAEATAKREAELKAEREAAEKAKRDASEKVKREAA
EKDKVSNQQTDEMTKTAQAEKARRENEAAELKRKAEEEARRKLEEEARRVAEEARRMAEENEKNGIDTAEQSEDTSD
YHVTTSQHARQAEDDNDREVEGGRGRGRNAKAARPAKKGNKHAESKADREEARAAVRGGKGGKQRKGSSLQQGFQKP
AQAVNRDVVIGETITVGDLANKMAVKGSQVIKAMMKLGAMATINQVIDQETAQLVAEEMGHKVILRRENELEEAVMS
DRDTGAAAEPRAPVVTIMGHVDHGKTSLLDYIRSTKVASGEAGGITQHIGAYHVETDNGMITFLDTPGHAAFTSMRA
RGAQATDIVVLVVAADDGVMPQTIEAIQHAKAAGVPVVVAVNKIDKPEADPDRVKNELSQYGILPEEWGGESQFVHV
SAKAGTGIDELLDAILLQAEVLELKAIRNGMASGAVIESFLDKGRGPVATVLVREGTLHKGDIVLCGFEYGRVRAMR
NELGQEVLEAGPSIPVEILGLSGVPAAGDEVTVVRDEKKAREVALYRQGKFREVKLARQQKSKLENMFANMAEGEVH
EVNVVLKADVQGSVEAISDSLLKLSTDEVKVKIIGSGVGGITETDATLAAASNAILVGFNVRADASARRVIEAESLD
LRYYSVIYNLIDEVKAAMSGMLSPELKQQIIGLAEVRDVFKSPKFGAIAGCMVTEGTIKRHNPIRVLRDNVVIYEGE
LESLRRFKDDVNEVRNGMECGIGVKNYNDVRVGDMIEVFEIIEIQRSID

>Phytobacter diazotrophicus
MTDVTVKTLAAEIQTSVDRLVQQFADAGIRKSAEDSVTAQEKQTLLAHLNREHGSGPDKLTLQRKTRSTLSIQGTGG
KSKSVQIEVRKKRTFVKRDPQEAERLAAEEEAKREAEDKARREAEEAAKREAEEKAKRDAEERAKREADEKAKRESA
DKAKREAAENSKVSNQQSDEMTRTAQGEKARREAEAQDLKRKAEEEARRKLEENARRVAEEARRMAEENESRWNASS
DKEEESSDYHVTTSHHARQAEDDSDREVEGGRGRGRNAKAARPAKKGNKHAESKADREEARAAIRGGKGGKQRKGSA
LQQSFQKPAQAVNRDVVIGETLTVGELANKMAVKGSQVIKAMMKLGAMATINQVIDQETAQLVAEEMGHKVILRREN
ELEEAVMSDRDTGAAAEPRAPVVTIMGHVDHGKTSLLDYIRSTKVASGEAGGITQHIGAYHVETENGMITFLDTPGH
AAFTAMRARGAQATDIVVLVVAADDGVMPQTIEAIQHAKAAQVPVVVAVNKIDKPEADPDRVKNELSQYGIMPEEWG
GESQFVHVSAKAGTGIDELLDAILLQAEVLELKAIRTGMASGVVIESFLDKGRGPVASVLVREGTLHKGDIVLCGFE
YGRVRAMRDELGREVSEAGPSIPVEILGLSGVPAAGDEATVVRDEKKAREVALYRQGKFREVKLARQQKSKLENMFA
NMTEGEVHEVNVVLKADVQGSVEAITDSLLKLSTDEVKVKIVGSGVGGITETDATLATASNAILVGFNVRADASARR
VIEAEGLDLRYYSVIYNLIDEVKAAMSGMLSPELKQQIIGLAEVRDVFKSPKFGAIAGCMVTEGTIKRHNPIRVLRD
NVVIYEGELESLRRFKDDVNEVRNGMECGIGVKNYNDVRVGDMIEVFEIIEIQRSID

>Kosakonia sacchari
MTDVTVKTLAAEIQTSVDRLVQQFADAGIRKSAEDSVTAQEKQTLLAHLNREHGSTPDKLTLQRKTRSTLSIQGTGG
KSKSVQIEVRKKRTFVKRDPQEAERLAAEEEAMREAEEKARREAEEAAEREAEDKAKRDAEARAKREADEKAKRENA
ENAKREAAENSKVSNQQSDEMTRTAQAEKARRESEAQELKRKAEEEARRKLEENARRVAEEARRMAEQNEANWTAPA
SEEEETTGDYHVTTSQHARQAEDDNDREVEGGRGRSRTAKAARPKKGNKHAESKADREEARAAVRGGKGGRQRKGSA
LQQGFQKPAQAVNRDVVIGETLTVGELANKMAVKGSQVIKAMMKLGAMATINQVIDQETAQLVAEEMGHKVILRREN
ELEEAVMSDRDTGAAAEPRAPVVTIMGHVDHGKTSLLDYIRSTKVASGEAGGITQHIGAYHVETDNGMITFLDTPGH
AAFTAMRARGAQATDIVVLVVAADDGVMPQTIEAIQHAKAAQVPVVVAVNKIDKPEADPDRVKNELSQYGIIPEEWG
GESQFVHVSAKAGTGIDELLDAILLQAEVLELKAVRKGMASGVVIESFLDKGRGPVASVLVREGTLHKGDIVLCGFE
YGRVRAMRDELGREVTEAGPSIPVEILGLSGVPAAGDEATVVRDEKKAREVALYRQGKFREVKLARQQKSKLENMFA
NMTEGEVHEVNIVLKADVQGSVEAISDSLLKLSTDEVKVKIVGSGVGGITETDATLAAASNAILVGFNVRADASARR
VIEAEGLDLRYYSVIYNLIDEVKAAMSGMLSPELKQQIIGLAEVRDVFKSPKFGAIAGCMVTEGVVKRHNPIRVLRD
NVVIYEGELESLRRFKDDVNEVRNGMECGIGVKNYNDVRTGDMIEVFEIIEIQRTIE

>Huaxiibacter chinensis
MTDVTVKSLAAEIQTPVDRLVQQFADAGIPKSADDSVTAQEKQTLLAHLNREHGSTPDKLTLQRKTRSTLNIPGTGG
KSKSVQIEVRKTRTFVKRDPQEAERLAAEEQAQREAEEQAQREAEATAKREAELKAEREAAEKAKRDASEKAKRDAA
EKDKVSNQQTDEMTKTAQTEKARRENEAAELKRKAEEEARRKLEEDARRVAEEARRMAEENAGVWAEQEKAKGDEDK
SDYHVTTSQHARQAEDENDREVEAGRSRTRTAAKAARPQKKGNKHAESKADREEARAAGRGGKGGKRKGSTLQQGFQ
KPAQAVNRDVVIGETITVGELANKMAVKGSQVIKAMMKLGAMATINQVIDQETAQLVAEEMGHKVILRRENELEEAV
MSDRDTGAAAEARAPVVTIMGHVDHGKTSLLDYIRSTKVASGEAGGITQHIGAYHVETENGMITFLDTPGHAAFTSM
RARGAQATDIVVLVVAADDGVMPQTIEAIQHAKAAQVPVVVAVNKIDKPEADMDRVKNELSQYGVMPEEWGGESQFI
PVSAKAGTGIDDLLNAILLQAEVLELKAVRNGMASGAVIESFLDKGRGPVATVLVREGTLNKGDIVLCGFEYGRVRA
MRNELGQEVLEAGPSIPVEILGLSGVPAAGDEVTVVRDEKKAREVALYRQGKFREVKLARQQKSKLENMFANMTDGE
VHEVNVVLKADVQGSVEAISDSLLKLSTDEVKVKIIGSGVGGITETDATLAAASNAILVGFNVRADASARKVIDAES
LDLRYYSVIYHLIDEVKAAMSGMLSPELKQQIIGLAEVRDVFKSPKFGAIAGCMVTEGTIKRHNPIRVLRDNVVIYE
GELESLRRFKDDVNEVRNGMECGIGVKNYNDVRVGDMIEVFEIIEIQRSIA

DNA replication/repair and cell division:

1. RecA

>Salmonella typhimurium
MAIDENKQKALAAALGQIEKQFGKGSIMRLGEDRSMDVETISTGSLSLDIALGAGGLPMGRIVEIYGPESSGKTTLT
LQVIAAAQREGKTCAFIDAEHALDPVYARKLGVDIDNLLCSQPDTGEQALEICDALARSGAVDVIVVDSVAALTPKA
EIEGEIGDSHMGLAARMMSQAMRKLAGNLKQSNTLLIFINQIRMKIGVMFGNPETTTGGNALKFYASVRLDIRRIGA
VKEGDNVVGSETRVKVVKNKIAAPFKQAEFQILYGEGINFYGELVDLGVKEKLIEKAGAWYSYNGEKIGQGKANATT
WLKENPATAKEIEKRVRELLLSNQNATPDFAVDDSEGVAETNEDF

>Escherichia coli
MAIDENKQKALAAALGQIEKQFGKGSIMRLGEDRSMDVETISTGSLSLDIALGAGGLPMGRIVEIYGPESSGKTTLT
LQVIAAAQREGKTCAFIDAEHALDPIYARKLGVDIDNLLCSQPDTGEQALEICDALARSGAVDVIVVDSVAALTPKA
EIEGEIGDSHMGLAARMMSQAMRKLAGNLKQSNTLLIFINQIRMKIGVMFGNPETTTGGNALKFYASVRLDIRRIGA
VKEGENVVGSETRVKVVKNKIAAPFKQAEFQILYGEGINFYGELVDLGVKEKLIEKAGAWYSYKGEKIGQGKANATA
WLKDNPETAKEIEKKVRELLLSNPNSTPDFSVDDSEGVAETNEDF

>Citrobacter rodentium
MAIDENKQKALAAALGQIEKQFGKGSIMRLGEDRSMDVETISTGSLSLDIALGAGGLPMGRIVEIYGPESSGKTTLT
LQVIAAAQREGKTCAFIDAEHALDPVYARKLGVDIDNLLCSQPDTGEQALEICDALARSGAVDVIVVDSVAALTPKA
EIEGEIGDSHMGLAARMMSQAMRKLAGNLKQSNTLLIFINQIRMKIGVMFGNPETTTGGNALKFYASVRLDIRRIGA
VKEGDNVVGSETRVKVVKNKIAAPFKQAEFQILYGEGINFYGELVDLGVKEKLIEKAGAWYSYNGEKIGQGKANATT
WLKENPATAKEIEKKVRELLLNNQDSTPDFSVDDGEGVAETNEDF

>Klebsiella variicola
MAIDENKQKALAAALGQIEKQFGKGSIMRLGEDRSMDVETISTGSLSLDIALGAGGLPMGRIVEIYGPESSGKTTLT
LQVIAAAQREGKTCAFIDAEHALDPVYARKLGVDIDNLLCSQPDTGEQALEICDALARSGAVDVIVVDSVAALTPKA
EIEGEIGDSHMGLAARMMSQAMRKLAGNLKQSNTLLIFINQIRMKIGVMFGNPETTTGGNALKFYASVRLDIRRIGA
VKEGDNVVGSETRVKVVKNKIAAPFKQAEFQILYGEGINFFGELVDLGVKEKLIEKAGAWYSYNGDKIGQGKANAIT
WLKENPAAAKEIEKKVRELLLNNQDSTPDFAVDGNDAEETEQDF

>Cronobacter turicensis
MAIDENKQKALAAALGQIEKQFGKGSIMRLGEDRTMDVETISTGSLSLDIALGAGGLPMGRIVEIYGPESSGKTTLT
LQVIAAAQRAGKTCAFIDAEHALDPVYARKLGVDIDNLLCSQPDTGEQALEICDALARSGAVDVLVVDSVAALTPKA
EIEGEIGDSHMGLAARMMSQAMRKLAGNLKNSNTLLIFINQIRMKIGVMFGNPETTTGGNALKFYASVRLDIRRIGA
VKEGEEVVGSETRVKVVKNKVAAPFKQAEFQILYGEGINFYGELVDLGVKHKLIEKAGAWYSYNGEKIGQGKANATN
FLKENKPMADEIEKKLREMLLNNQDATPDFTVDDNDGGVEETNEEF

>Enterobacter cloacae
MAIDENKQKALAAALGQIEKQFGKGSIMRLGEDRTMDVETISTGSLSLDIALGAGGLPMGRIVEIYGPESSGKTTLT
LQVIAAAQREGKTCAFIDAEHALDPVYARKLGVDIDNLLCSQPDTGEQALEICDALARSGAVDVIVVDSVAALTPKA
EIEGEIGDSHMGLAARMMSQAMRKLAGNLKQSNTLLIFINQIRMKIGVMFGNPETTTGGNALKFYASVRLDIRRIGA
VKEGENVVGSETRVKVVKNKIAAPFKQAEFQILYGEGINFFGELVDLGVKEKLIEKAGAWYSYNGDKIGQGKANAIS
WLKENPAAAKEIEKKVRELLLNNQDSKPDFVVDGADAEETNEDF

>Phytobacter diazotrophicus
MAIDENKQKALAAALGQIEKQFGKGSIMRLGEDRSMDVETISTGSLSLDIALGAGGLPMGRIVEIYGPESSGKTTLT
LQVIAAAQREGKTCAFIDAEHALDPVYARKLGVDIDNLLCSQPDTGEQALEICDALARSGAVDVIVVDSVAALTPKA
EIEGEIGDSHMGLAARMMSQAMRKLAGNLKQSNTLLIFINQIRMKIGVMFGNPETTTGGNALKFYASVRLDIRRVGA
VKEGDNVVGSETRVKVVKNKIAAPFKQAEFQILYGEGINFYGELVDLGVKEKLIEKAGAWYSYNGEKIGQGKANATN
WLKENPATAKEIEKKVREILLSNPSSGADFSVDDSGEGVEETNEDF

>Kosakonia sacchari
MAIDENKQKALAAALGQIEKQFGKGSIMRLGEDRSMDVETISTGSLSLDIALGAGGLPMGRIVEIYGPESSGKTTLT
LQVIAAAQREGKTCAFIDAEHALDPVYARKLGVDIDNLLCSQPDTGEQALEICDALARSGAVDVLVVDSVAALTPKA
EIEGEIGDSHMGLAARMMSQAMRKLAGNLKQSNTLLIFINQIRMKIGVMFGNPETTTGGNALKFYASVRLDIRRIGA

VKEGDNVVGSETRVKVVKNKIAAPFKQAEFQILYGEGINFYGELVDLGVKEKLIEKAGAWYSYNGDKIGQGKANATT
WLKENPVAAKEIEKKLREILLSNQNSSAEFTADGNDEDVEETNEDF

>Huaxiibacter chinensis
MAIDENKQKALAAALGQIEKQFGKGSIMRLGEDRSMDVETISTGSLSLDIALGAGGLPMGRIVEIYGPESSGKTTLT
LQAIAAAQRQGKTCAFIDAEHALDPIYARKLGVDIDNLLCSQPDTGEQALEICDALARSGAVDVIVVDSVAALTPKA
EIEGEIGDSHMGLAARMMSQAMRKLAGNLKQSNTLLIFINQIRMKIGVMFGNPETTTGGNALKFYASVRLDIRRIGA
VKEGENVVGSETRVKVVKNKIAAPFKQAEFQILYGEGINFLGELVDLGVKEKLIEKAGAWYSYNGDKIGQGKANAIS
WLKENPAAAKEIEKKVRELLLSNPDSKPDFVVDAADASESNEDF

2. MutS

>Salmonella typhimurium
MNESFDKDFSNHTPMMQQYLKLKAQHPEILLFYRMGDFYELFYDDAKRASQLLDISLTKRGASAGEPIPMAGIPHHA
VENYLAKLVNQGESVAICEQIGDPATSKGPVERKVVRIVTPGTISDEALLQERQDNLLAAIWQDGKGYGYATLDISS
GRFRLSEPADRETMAAELQRTNPAELLYAEDFAEMALIEGRRGLRRRPLWEFEIDTARQQLNLQFGTRDLVGFGVEN
ASRGLCAAGCLLQYVKDTQRTSLPHIRSITMERQQDSIIMDAATRRNLEITQNLAGGVENTLAAVLDCTVTPMGSRM
LKRWLHMPVRNTDILRERQQTIGALQDTVSELQPVLRQVGDLERILARLALRTARPRDLARMRHAFQQLPELHAQLE
TVDSAPVQALRKKMGDFAELRDLLERAIIDAPPVLVRDGGVIAPGYHEELDEWRALADGATDYLDRLEIRERERTGL
DTLKVGYNAVHGYYIQISRGQSHLAPINYVRRQTLKNAERYIIPELKEYEDKVLTSKGKALALEKQLYDELFDLLLP
HLADLQQSANALAELDVLVNLAERAWTLNYTCPTFTDKPGIRITEGRHPVVEQVLNEPFIANPLNLSPQRRMLIITG
PNMGGKSTYMRQTALIALLAYIGSYVPAQNVEIGPIDRIFTRVGAADDLASGRSTFMVEMTETANILHNATENSLVL
MDEIGRGTSTYDGLSLAWACAENLANKIKALTLFATHYFELTQLPEKMEGVANVHLDALEHGDTIAFMHSVQDGAAS
KSYGLAVAALAGVPKEVIKRARQKLRELESISPNAAATQVDGTQMSLLAAPEETSPAVEALENLDPDSLTPRQALEW
IYRLKSLV

>Escherichia coli
MSAIENFDAHTPMMQQYLRLKAQHPEILLFYRMGDFYELFYDDAKRASQLLDISLTKRGASAGEPIPMAGIPYHAVE
NYLAKLVNQGESVAICEQIGDPATSKGPVERKVVRIVTPGTISDEALLQERQDNLLAAIWQDSKGFGYATLDISSGR
FRLSEPADRETMAAELQRTNPAELLYAEDFAEMSLIEGRRGLRRRPLWEFEIDTARQQLNLQFGTRDLVGFGVENAP
RGLCAAGCLLQYAKDTQRTTLPHIRSITMEREQDSIIMDAATRRNLEITQNLAGGAENTLASVLDCTVTPMGSRMLK
RWLHMPVRDTRVLLERQQTIGALQDFTAGLQPVLRQVGDLERILARLALRTARPRDLARMRHAFQQLPELRAQLETV
DSAPVQALREKMGEFAELRDLLERAIIDTPPVLVRDGGVIASGYNEELDEWRALADGATDYLERLEVRERERTGLDT
LKVGFNAVHGYYIQISRGQSHLAPINYMRRQTLKNAERYIIPELKEYEDKVLTSKGKALALEKQLYEELFDLLLPHL
EALQQSASALAELDVLVNLAERAYTLNYTCPTFIDKPGIRITEGRHPVVEQVLNEPFIANPLNLSPQRRMLIITGPN
MGGKSTYMRQTALIALMAYIGSYVPAQKVEIGPIDRIFTRVGAADDLASGRSTFMVEMTETANILHNATEYSLVLMD
EIGRGTSTYDGLSLAWACAENLANKIKALTLFATHYFELTQLPEKMEGVANVHLDALEHGDTIAFMHSVQDGAASKS
YGLAVAALAGVPKEVIKRARQKLRELESISPNAAATQVDGTQMSLLSVPEETSPAVEALENLDPDSLTPRQALEWIY
RLKSLV

>Citrobacter rodentium
MSTLENLDAHTPMMQQYLKLKAQHPEILLFYRMGDFYELFYDDAKRASQLLDISLTKRGASAGEPIPMAGIPHHAVE
NYLAKLVNQGESVAICEQIGDPATSKGPVERKVVRIVTPGTISDEALLQERQDNLLAAIWKDSKGYGYATLDISSGR
FRLSEPADRETMAAELQRTNPAELLYAEDFSEMALIEGRRGLRRRPLWEFEIDTARQQLNLQFGTRDLVGFGVENAP
RGLCAAGCLLQYVKDTQRTSLPHIRSITMERQQDSIIMDAATRRNLEITQNLAGGVENTLASVLDCTVTPMGSRMLK
RWLHMPVRDTRILTERQQTIGALQDLTAELQPVLRQVGDLERILARLALRTARPRDLARMRHAFQQLPELHAQLEGV
DSAPVQMLREKMGEFTELRELLERAIIDTPPVLVRDGGVIAPGYNEELDEWRALADGATDYLDKLEIRERERTGLDT
LKVGFNAVHGYYIQISRGQSHLAPINYTRRQTLKNAERYIIPELKEYEDKVLTSKGKALALEKQLYDELFDLLLPHL
ADLQQSASALAEIDVLVNLAERAYTLNYTCPIFTDKPGIRITEGRHPVVEQVLNEPFIANPLDLSPQRRMLIITGPN
MGGKSTYMRQTALIALLAYIGSYVPAQKVEIGPIDRIFTRVGAADDLASGRSTFMVEMTETANILHNATDNSLVLMD
EIGRGTSTYDGLSLAWACAENLANKIKALTLFATHYFELTQLPEKMEGVANVHLDALEHGDTIAFMHSVQDGAASKS
YGLAVAALAGVPKEVIKRARQKLRELESISPNAAATQVDGTQMSLLSIPEETSPAVEALENLDPDSLTPRQALEWIY
RLKSLV

>Klebsiella variicola
MSTIDNLDAHTPMMQQYLKLKAQHPDILLFYRMGDFYELFYDDAKRASQLLDISLTKRGASAGEPIPMAGIPHHAVE
NYLAKLVNQGESVAICEQIGDPATTKGPVERKVVRIVTPGTISDEALLQERQDNLLAAIWQDSKGFGYATLDISSGR

FRLSEPADRETMAAELQRTNPAELLYAEDFAESSLIEGRRGLRRRPLWEFEIDTARQQLNLQFGTRDLVGFGVENAP
RGLCAAGCLLQYVKDTQRTSLPHIRSITMERQQDSIIMDAATRRNLEITQNLAGGTDNTLASVLDCTVTPMGSRMLK
RWLHMPVRDTAVLVERQQTIGALQERYTELQPVLRQVGDLERILARLALRTARPRDLARMRHALQQLPLLRELLADV
DRQPVQKLREKMGEFTELRELLERSVIDAPPVLVRDGGVIAPGYSEELDEWRALADGATDYLDKLEIRERERLGLDT
LKVGYNAVHGYYIQISRGQSHLAPIHYVRRQTLKNAERYIIPELKEYEDKVLTSKGKALALEKQLYDELFDLLLPHL
ADLQTSASALAELDVLVNLAERAETLNYCCPTFSDKPGIRISEGRHPVVEQVLKEPFIANPLQLAPQRRMLIITGPN
MGGKSTYMRQTALIALLAYIGSYVPAQKVEIGPIDRIFTRVGAADDLASGRSTFMVEMTETANILHNATEHSLVLMD
EIGRGTSTYDGLSLAWACAENLANKIKALTLFATHYFELTQLPEKMEGVANVHLDALEHGDTIAFMHSVQDGAASKS
YGLAVAALAGVPKEVIKRARQKLRELESISPNAAATQVDGTQMSLLAAPEETSPAVEALENLDPDSLTPRQALEWIY
RLKSLV

>Cronobacter turicensis
MSTSETFDAHTPMMQQYLKLKAQHPDILLFYRMGDFYELFYDDAKRASQLLDISLTKRGASAGEPIPMAGVPHHAVE
NYLAKLVNLGESVAICEQIGDPATSKGPVERKVVRIVTPGTISDEALLQERQDNLLAAIWQDGKGFGYATLDISSGR
FRLTEPQDRETMAAELQRTNPAELLYAEDFAEMALIEGRRGLRRRPLWEFELDTARQQLNLQFGTRDLVGFGVENAP
RGLCAAGCLLQYVKDTQRTSLPHIRSITMERQQDGIIMDAATRRNLEITQNLAGGVENTLASVLDCTVTPMGSRMLK
RWLHMPVRDASVLRHRQQAIAALMEYSTDIQPVLRQVGDLERILARLALRTARPRDLARMRHAFQQLPTLNTLLTDI
DAGYVQTLREQMGEFTELRDLLERAIIEAPPVLVRDGGVIAPGYHAELDEWRALADGATDYLDRLEIREREKLGIDT
LKVGFNAVHGYFIQVSRGQSHMVPIHYVRRQTLKNAERYIIPELKEYEDKVLTSKGKALALEKQLYDELFDLLLPHL
AELQKSAAALAELDVLTNLAERADTLNYHCPTLTDKPGVRLVEGRHPVVERVLNEPFIANPLSLSPQRRMLIITGPN
MGGKSTYMRQTALIVLMAYIGSFVPAEQAEIGPIDRIFTRVGAADDLASGRSTFMVEMTETANILHNATEHSLVLMD
EIGRGTSTYDGLSLAWACAESLANRIKALTLFATHYFELTQLPEKMEGVANVHLDAIEHGDTIAFMHSVQDGAASKS
YGLAVAALAGVPKEVIKRARQKLRELESISGNAAATQVDGTQMSLLVAAEETSPAIEALENLDPDSLSPRQALEWIY
RLKSLV

>Enterobacter cloacae
MSTLENFDAHTPMMQQYLKLKAQHPEILLFYRMGDFYELFYDDAKRASQLLDISLTKRGASAGEPIPMAGIPHHAVE
NYLAKLVNQGESVAICEQIGDPATSKGPVERKVVRIVTPGTISDEALLQERQDNLLAALWQDGKGFGYATLDISSGR
FRLSEPADRETMAAELQRTNPAELLYAEDFAEMALIEGRRGLRRRPLWEFEIDTARQQLNLQFGTRDLIGFGVENAP
RGLCAAGCLLQYVKDTQRTALPHIRSITMERQQDSIIMDAATRRNLEITQNLAGGVENTLASVLDNTVTPMGSRMLK
RWLHMPVRDTDTLVCRQQTIAALQDRYTELQPVLRQVGDLERILARLALRTARPRDLARMRHAFQQLPELRAQLSDV
DSAPVQKLRETMGEFAELRELLERAIIDAPPVLVRDGGVIAPGYNEELDEWRALADGATDYLDKLEIRERERLGLDT
LKVGYNAVHGYYIQISRGQSHLAPIHYVRRQTLKNAERYIIPELKEYEDKVLTSKGKALALEKQLYDELFDMLMPHL
ADLQLSAGALAELDVLVNLAERAETLNYTCPTFTDKPGIRITEGRHPVVEQVLNEPFIANPLSLSPQRRMLIITGPN
MGGKSTYMRQTALIALLAYIGSYVPAQKVEIGPIDRIFTRVGAADDLASGRSTFMVEMTETANILHNATEHSLVLMD
EVGRGTSTYDGLSLAWACAENLANKIKAMTLFATHYFELTQLPEKMEGVANVHLDALEHGDTIAFMHTVQEGAASKS
YGLAVAALAGVPKEVIKRARQKLRELESLSPNAAATQIDGTQMSLLAPAEETSPAVEALENLDPDSLTPRQALEWIY
RLKSLV

>Phytobacter diazotrophicus
MSTLENFDAHTPMMQQYLKLKAQHPDILLFYRMGDFYELFYDDAKRASQLMDISLTKRGASAGEPIPMAGVPHHAVE
NYLAKLVSLGESVAICEQIGDPATSKGPVERKVVRIVTPGTISDEALLQERQDNLLAAIWQDSKGFGYATLDISSGR
FRVSEPADRETMAAELQRTNPAELLYAEDFAETSLIEGRRGLRRRPLWEFEIDTARQQLNLQFGTRDLVGFGVENAP
RGLCAAGCLLQYVKDTQRTALPHIRSITMERQQDSIIMDAATRRNLEITQNLAGGIENTLASVLDCTVTPMGSRMLK
RWLHMPVRDTKTLLERQQTIGALQENTGELQPVLRQVGDLERILARLALRTARPRDLARMRHAFQQLPELRAQLAQV
KSEPVQNLREKMGEFTELRELLERAIIDAPPVLVRDGGVIAPGYNAELDEWRALADGATDYLDRLEIREREKLGLDT
LKVGYNAVHGYYIQISRGQSHLAPIHYVRRQTLKNAERYIIPELKEYEDKVLTSKGKALALEKQLYDELFDKLLPHL
GDLQQSATALAELDVLVNLAERADTLNYCCPTFSDKPGIRIVEGRHPVVERVLNEPFIANPLNLSPQRRMLIITGPN
MGGKSTYMRQTALIALLAYIGSYVPAQKVEIGPIDRIFTRVGAADDLASGRSTFMVEMTETANILHNATENSLVLMD
EIGRGTSTYDGLSLAWACAENLANKIKALTLFATHYFELTQLPEKMEGVANVHLDALEHGDTIAFMHSVQDGAASKS
YGLAVAALAGVPKEVIKRARGKLRELESLSPSAGATQIDGTQMSLLAVPEETSPAMEALENLDPDSLTPRQALEWIY
RLKSLV

>Kosakonia sacchari
MSTLENFDAHTPMMQQYLKLKAQHPDILLFYRMGDFYELFYDDAKRASQLMDISLTKRGASAGEPIPMAGVPHHAVE
NYLAKLVSLGESVAICEQIGDPATSKGPVERKVVRIVTPGTISDEALLQERQDNLLAAVWQDSKGFGYATLDISSGR
FRVSEPTDRDTMAAELQRTNPAELLYAEDFAETALIEGRRGLRRRPLWEFEIDTARQQLNMQFGTRDLVGFGVENAP

RGLCAAGCLLQYVKDTQRTALPHIRSITMERQQDTIIMDAATRRNLEITQNLAGGVENTLSSVLDCTVTPMGSRMLK
RWLHMPIRDTKVLTERQQTISALQEHTSELQPVLRQVGDLERILARLALRTARPRDLARMRYAFQQLPELRSQLADI
DSAPVQALREKMGEFVELRELLERAIIEAPPVLVRDGGVIAPGYNAELDEWRGLADGATDYLDRLEVRERERLGLDT
LKVGYNAVHGYFIQISRGQSHLAPIHYVRRQTLKNAERYIIPELKEYEDKVLTSKGKALALEKQLYEELFDLLMPHL
ADLQQSAAALAELDVLVNLAERAYTLNYCCPTFSEKPGIRISEGRHPVVEQVLKEPFIANPLNLSPQRRMLIITGPN
MGGKSTYMRQTALIALLAYIGSYVPAQKVEIGPIDRIFTRVGAADDLASGRSTFMVEMTETANILHNATENSLVLMD
EIGRGTSTYDGLSLAWACAENLANKIKALTLFATHYFELTQLPEKMEGVANVHLDALEHGDTIAFMHSVQDGAASKS
YGLAVAALAGVPKEVIKRARGKLRELESLSPSAAATQIDGTQMSLLAAPEETNPAVEALENLDPDSLTPRQALEWIY
RLKNLL

>Huaxiibacter chinensis
MSTIDNLTAHTPMMQQYLKLKAQHPEILLFYRMGDFYELFYDDAKRASQLLDISLTKRGASAGEPIPMAGIPHHAVE
NYLAKLVNQGESVAICEQIGDPATSKGPVERKVVRIVTPGTISDEALLHERQDNLLAAVWQDSKGFGYATLDISSGR
FRLSEPADRETMAAELQRTNPAELLYAEDFAEFALIEGRRGLRRRPLWEFEIDTARQQLNLQFGTRDLIGFGVENAP
RGLCAAGCLLQYVKDTQRTALPHIRSITMERQQDSIIMDAATRRNLEITQNLAGGIDNTLASVLDSTVTPMGSRMLK
RWLHMPVRDTQVLVSRQQTIGALQDRFTELQPVLRQVGDLERILARLALRTARPRDLARMRHAFQQLPELRAQLAEV
DSAPVQKLRETMGEFTELRELLERAVVEAPPVLVRDGGVIAPGYHQELDEWRALADGATDYLDKLEIRERERLGLDT
LKVGYNAIHGYYIQISRGQSHLAPIHYVRRQTLKNAERYIIPELKEYEDKVLSSRGKALALEKQLYEELFDLLMPHL
GDLQQSASALAELDVLVNLAERADALNYTCPTFIDKPGIRITEGRHPVVEQVLREPFIANPLSLSPQRRMLIITGPN
MGGKSTYMRQTALIALLAYIGSYVPAQQVEIGPIDRIFTRVGAADDLASGRSTFMVEMTETANILHNATENSLVLMD
EIGRGTSTYDGLSLAWACAESLANKIKALTLFATHYFELTQLPEKMEGVANVHLDALEHGDTIAFMHTVQDGAASKS
YGLAVAALAGVPKEVIKRARQKLRELETLSPNAAATQVDGTQMSLLAPAEETSPAVEALENLDPDSLTPRQALEWIY
RLKSLV

3. DnaC

>Salmonella typhimurium
MKNVGDLMQRLQKMMPAHITPAFKTGEELLAWQKEQGEIRAAALARENRAMKMQRTFNRSGIRPLHQNCSFDNYRVE
CDGQMNALSKARQYVDEFDGNIASFVFSGKPGTGKNHLAAAICNELLLRGKSVLIITVADIMSAMKDTFSNRETSEE
QLLNDLSNVDLLVIDEIGVQTESRYEKVIINQIVDRRSSSKRPTGMLTNSNMEEMTKMLGERVMDRMRLGNSLWVNF
TWDSYRSRVTGKEY

>Escherichia coli
MKNVGDLMQRLQKMMPAHIKPAFKTGEELLAWQKEQGAIRSAALERENRAMKMQRTFNRSGIRPLHQNCSFENYRVE
CEGQMNALSKARQYVEEFDGNIASFIFSGKPGTGKNHLAAAICNELLLRGKSVLIITVADIMSAMKDTFRNSGTSEE
QLLNDLSNVDLLVIDEIGVQTESKYEKVIINQIVDRRSSSKRPTGMLTNSNMEEMTKLLGERVMDRMRLGNSLWVIF
NWDSYRSRVTGKEY

>Citrobacter rodentium
MKNVGDLMKRLQKMMPAHIKPAFTTGEELLAWQKQQGEIRAAALARENRAMKMQRTFNRSGIRPLHQNCSFDNYRVE
CEGQMNALSKARQYVEEFDGNIASFIFSGKPGTGKNHLAAAICNELLLRGKSVLIITVADIMSAMKETFSNRETSEE
QLLNDLSNVDLLVIDEIGVQTESRYEKVIINQIVDRRSSSKRPTGMLTNSNMEEMTKLLGERVMDRMRLGNSLWVIF
NWESYRSRVTGKEY

>Klebsiella variicola
MKNVGDLMKRLQKMMPAHIEPAFKTGEELLAWQKEQGRLRSEALERENRAMKMQRTFNRSGIRPLHQNCSFDNYRVE
CEGQMNALARARQYVEEFDGNIASFIFSGKPGTGKNHLAAAICNELLLRGKSVLIITVADIMSAMKDTFGNRETSEE
QLLSDLSKVDLLVIDEIGMQTESRYEKVIINQIVDRRSSSKRPTGMLTNSNMEEMNKLLGERVMDRMRLGNSLWVIF
NWESYRHRVTGKEY

>Cronobacter turicensis
MKNVTDLMMRLKRLMPPDVTPAFKTGEELIAWQQEQGRIRSEAIERENRAMKMQRTFNRSGIRPLHQNCSFDNYRVE
NDGQMNALSKARQYVEAFDDNIASFVFAGKPGTGKNHLAAAICNELLLRGKSVMIITVADIMSAMKDTFSSRETSEE
RLLKDLSSVDLLVIDEIGMQSESRYEKVIINQLVDRRSSSKRPTGMLTNQNMDEMTKLLGERVMDRMRLGNSLWVNF
NWDSYRSRVTGKEY

>Enterobacter cloacae

MKNVGDLMKRLQKMMPANVKPAFTTGEELLAWQKEQGEIRAAALARENRAMKMQRTFNRSGIRPLHQNCSFDNYKIE
TNGQMNALAAARQYVDEFDGNIASFIFSGKPGTGKNHLAAAICNELLLRGKSVLIITVADIMSAMKDTFSNRETSEE
QLLNDLSNVDLLVIDEIGVQTESRYEKVIINQIVDRRSSSKRPTGMLTNHNIDEMTRLLGERVMDRMKLGNSLYVIF
DWESYRSRVTGKEY

>Phytobacter diazotrophicus
MKNVAELMKRLQRMMPANTQPAFKNGEELMAWQKEQGKIRAAAIARENRAMKMQRTFNRSGIRPLHQNCSFDNYKVE
CEGQMRALSQARQYVAEFDGNIASFIFSGKPGTGKNHLAAAICNELLLHGKSVLIITVADIMSAMKETFGNRDNSEE
QLLNDLSSVDLLVIDEIGMQTESRYEKVIINQIVDRRSSSKRPTGMLTNSNLEEMNKLLGERVMDRMRLGNSLWVIF
NWDSYRSRVTGKEY

>Kosakonia sacchari
MKNVGELMKRLQKMMPANTEPAFKTGEELMAWQKQQGEIRSAALERENRAMKMQRTFNRSGIRPLHQNCSFDNYRVE
TEGQMRALSQARQYVEEFDGNIASFIFSGKPGTGKNHLAAAICNELLLRGKSVLIITVADIMSAMKETFGNKENSEE
QLLNDLSSVDLLVIDEIGMQTESRYEKVIINQIVDRRSSSKRPTGMLTNSNLEEMNKLLGERVMDRMRLGNSLWVIF
NWESYRSRVTGKEY

>Huaxiibacter chinensis
MKNVGDLMKRLQKMMPANVKPAFTTGEELLAWQKEQGQLRAAALARENRAMKMQRTFNRSGIRPLHQNCSFDNYKVE
SQGQMNALNLARQYVDEFDGNIASFIFSGKPGTGKNHLAAAICNELLLRGKSVLIITVADIMSAMKETFSNRETSEE
QLLNDLSNVDLLVIDEIGVQTESRYEKVIINQIVDRRSSSKRPTGMLTNHNIDDMTRLLGDRVMDRMKLGNSLYVIF
DWDSFRSRVTGKEY

4. FtsA

>Salmonella typhimurium
MIKATDRKLVVGLEIGTAKVAALVGEVLPDGMVNIIGVGSCPSRGMDKGGVNDLESVVKCVQRAIDQAELMADCQIS
SVYLALSGKHISCQNEIGMVPISEEEVTQEDVENVVHTAKSVRVRDEHRVLHVIPQEYAIDYQEGIKNPVGLSGVRM
QAKVHLITCHNDMAKNIVKAVERCGLKVDQLIFAGLAASYSVLTEDERELGVCVVDIGGGTMDIAVYTGGALRHTKV
IPYAGNVVTSDIAYAFGTPPSDAEAIKVRHGCALGSIVGKDESVEVPSVGGRPPRSLQRQTLAEVIEPRYTELLNLV
NEEILQLQEQLRQQGVKHHLAAGIVLTGGAAQIEGLAACAQRVFHTQVRIGAPLNITGLTDYAQEPYYSTAVGLLHY
GKESHLNGEAEVEKRVTASVGSWIKRLNSWLRKEF

>Escherichia coli
MIKATDRKLVVGLEIGTAKVAALVGEVLPDGMVNIIGVGSCPSRGMDKGGVNDLESVVKCVQRAIDQAELMADCQIS
SVYLALSGKHISCQNEIGMVPISEEEVTQEDVENVVHTAKSVRVRDEHRVLHVIPQEYAIDYQEGIKNPVGLSGVRM
QAKVHLITCHNDMAKNIVKAVERCGLKVDQLIFAGLASSYSVLTEDERELGVCVVDIGGGTMDIAVYTGGALRHTKV
IPYAGNVVTSDIAYAFGTPPSDAEAIKVRHGCALGSIVGKDESVEVPSVGGRPPRSLQRQTLAEVIEPRYTELLNLV
NEEILQLQEKLRQQGVKHHLAAGIVLTGGAAQIEGLAACAQRVFHTQVRIGAPLNITGLTDYAQEPYYSTAVGLLHY
GKESHLNGEAEVEKRVTASVGSWIKRLNSWLRKEF

>Citrobacter rodentium
MIKATDRKLVVGLEIGTAKVAALVGEVLPDGMINIIGVGSCPSRGMDKGGVNDLESVVKCVQRAIDQAELMADCQIS
SVYLALSGKHISCQNEIGMVPISEEEVTQEDVENVVHTAKSVRVRDEHRVLHVIPQEYAIDYQEGIKNPVGLSGVRM
QAKVHLITCHNDMAKNIVKAVERCGLKVDQLIFAGLAASYSVLTEDERELGVCVVDIGGGTMDIAVYTGGALRHTKV
IPYAGNVVTSDIAYAFGTPPSDAEAIKVRHGCALGSIVGKDESVEVPSVGGRPPRSLQRQTLAEVIEPRYTELLNLV
NEEILQLQEQLRQQGVKHHLAAGIVLTGGAAQIEGLAACAQRVFHTQVRIGAPLNITGLTDYAQEPYYSTAVGLLHY
GKESHLSGEADVEKRVTASVGSWIKRLNSWLRKEF

>Klebsiella variicola
MIKATDRKLVVGLEIGTAKVAALVGEVLPDGMINIIGVGSCPSRGMDKGGVNDLESVVKCVQRAIDQAELMADCQIS
SVYLALSGKHISCQNEIGMVPISEEEVTLDDVENVVHTAKSVRVRDEHRVLHVIPQEYAIDYQEGIKNPVGLSGVRM
QAKVHLITCHNDMAKNIVKAVERCGLKVDQLIFAGLAASYSVLTEDERELGVCVVDIGGGTMDIAVYTGGALRHTKV
IPYAGNVVTSDIAYAFGTPPSDAEAIKVRHGCALGSIVGKDENVEVPSVGGRPPRSLQRQTLAEVIEPRYTELLNLV
NEEILQLQEQLRQQGVKHHLAAGIVLTGGAAQIEGLAACAQRVFHTQVRIGAPLNITGLTDYAQEPYYSTAVGLLHY
GKESHLSGEAEVEKRVTASVGSWIKRLNSWLRKEF

>Cronobacter turicensis
MIKATDRKLVVGLEIGTAKVAALVGEVLPDGMINIIGVGSCPSRGMDKGGVNDLESVVKCVQRAIDQAELMADCQIS
SVYLALSGKHISCQNEIGMVPISEEEVTQEDVENVVHTAKSVRVRDEHRVLHVIPQEYAIDYQEGIKNPVGLSGVRM
QAKVHLITCHNDMAKNIVKAVERCGLKVDQLIFAGLASSYSVLTEDERELGVCVVDIGGGTMDIAVYTGGALRHTKV
IPYAGNVVTSDIAYAFGTPPSDAEAIKVRHGCALGSLVGKDESVEVPSVGGRPPRSLQRQTLAEVIEPRYTELLNLV
NEEILQLQEQLRQQGVKHHLAAGIVLTGGAAQIEGLAACAQRVFHTQVRIGQPLNITGLTDYAQEPYYSTAVGLLHY
GKESHLSGEAEVEKRTSVGSWIKRINSWLRKEF

>Enterobacter cloacae
MIKATDRKLVVGLEIGTAKVAALVGEVLPDGMVNIIGVGSCPSRGMDKGGVNDLESVVKCVQRAIDQAELMADCQIS
SVYLALSGKHISCQNEIGMVPISEEEVTQEDVENVVHTAKSVRVRDEHRVLHVIPQEYAIDYQEGIKNPVGLSGVRM
QAKVHLITCHNDMAKNIVKAVERCGLKVDQLIFAGLAASYSVLTEDERELGVCVVDIGGGTMDMAVYTGGALRHTKV
IPYAGNVVTSDIAYAFGTPPSDAEAIKVRHGCALGSIVGKDESVEVPSVGGRPPRSLQRQTLAEVIEPRYTELLNLV
NEEILQLQEQLRQQGVKHHLAAGIVLTGGAAQIEGLAACAQRVFHTQVRIGAPLNITGLTDYAQEPYYSTAVGLLHY
GKESHLSGEAEVEKRVSVGSWVKRLNNWLRKEF

>Phytobacter diazotrophicus
MIKATDRKLVVGLEIGTAKVAALVGEVLPDGMINIIGVGSCPSRGMDKGGVNDLESVVKCVQRAIDQAELMADCQIS
SVYLALSGKHISCQNEIGMVPISEEEVTQEDVENVVHTAKSVRVRDEHRVLHVIPQEYAIDYQEGIKNPVGLSGVRM
QAKVHLITCHNDMAKNIVKAVERCGLKVDQLIFAGLASSYSVLTEDERELGVCVVDIGGGTMDMAVYTGGALRHTKV
IPYAGNVVTSDIAYAFGTPPSDAEAIKVRHGCALGSIVGKDESVEVPSVGGRPPRSLQRQTLADVIEPRYTELLNLV
NDEILQLQEQLRQQGVKHHLAAGIVLTGGAAQIEGLAACAQRVFHTQVRIGAPLNITGLTDYAQEPYYSTAVGLLHY
GKETHLSGEAEVEKRASVGSWFKRINSWLRKEF

>Kosakonia sacchari
MIKATDRKLVVGLEIGTAKVAALVGEVLPDGIVNIIGVGSCPSRGMDKGGVNDLESVVKCVQRAIDQAELMADCQIS
SVYLALSGKHISCQNEIGMVPISEEEVTQEDVENVVHTAKSVRVRDEHRVLHVIPQEYAIDYQEGIKNPVGLSGVRM
QAKVHLITCHNDMAKNIVKAVERCGLKVDQLIFAGLASSYSVLTEDERELGVCVVDIGGGTMDMAVYTGGALRHTKV
IPYAGNVVTSDIAYAFGTPPSDAEAIKVRHGCALGSLVGKDESVEVPSVGGRPPRSLQRQTLADVIEPRYTELLNLV
NDEILQLQEQLRQQGVKHHLAAGIVLTGGAAQMEGLAACAQRVFHTQVRIGAPLNITGLTDYAQEPYYSTAVGLLHY
GKETHLSGEAEVEKRVSVGSWFKRINSWLRKEF

>Huaxiibacter chinensis
MIKATDRKLVVGLEIGTAKVAALVGEVLPDGMVNIIGVGSCPSRGMDKGGVNDLESVVKCVQRAIDQAELMADCQIS
SVYLALSGKHISCQNEIGMVPISEEEVTQEDVENVVHTAKSVRVRDEHRVLHVIPQEYAIDYQEGIKNPVGLSGVRM
QAKVHLITCHNDMAKNIVKAVERCGLKVDQLIFAGLAASYSVLTEDERELGVCVVDIGGGTMDIAVYTGGALRHTKV
IPYAGNVVTSDIAYAFGTPPSDAEAIKVRHGCALGSIVGKDESVEVPSVGGRPPRSLQRQTLAEVIEPRYTELLNLV
NEEILQLQEQLRQQGVKHHLAAGIVLTGGAAQIEGLAACAQRVFHTQVRIGAPLNITGLTDYAQEPYYSTAVGLLHY
GKESHLSGEAEVEKRASVGSWVKRLNSWLRKEF

5. MinD

>Salmonella typhimurium
MARIIVVTSGKGGVGKTTSSAAIATGLAQKGKKTVVIDFDIGLRNLDLIMGCERRVVYDFVNVIQGDATLNQALIKD
KRTENLFILPASQTRDKDALTREGVAKVLDSLKAMDFEFIVCDSPAGIETGALMALYFADEAIITTNPEVSSVRDSD
RILGILASKSRRAENGEEPIKEHLLLTRYNPGRVNKGDMLSMEDVLEILRIKLVGVIPEDQSVLRASNQGEPVILDA
TADAGKAYADTVDRLLGEERPFRFIEEEKKGFLKRLFGG

>Escherichia coli
MARIIVVTSGKGGVGKTTSSAAIATGLAQKGKKTVVIDFDIGLRNLDLIMGCERRVVYDFVNVIQGDATLNQALIKD
KRTENLYILPASQTRDKDALTREGVAKVLDDLKAMDFEFIVCDSPAGIETGALMALYFADEAIITTNPEVSSVRDSD
RILGILASKSRRAENGEEPIKEHLLLTRYNPGRVSRGDMLSMEDVLEILRIKLVGVIPEDQSVLRASNQGEPVILDI
NADAGKAYADTVERLLGEERPFRFIEEEKKGFLKRLFGG

>Citrobacter rodentium
MARIIVVTSGKGGVGKTTSSAAIATGLAQKGKKTVVIDFDIGLRNLDLIMGCERRVVYDFVNVIQGDATLNQALIKD
KRTENLFILPASQTRDKDALTRDGVAKVLDDLKAMDFEFIVCDSPAGIETGALMALYFADEAIITTNPEVSSVRDSD

RILGILASKSRRAENGEEPIKEHLLLTRYNPGRVNKGDMLSMEDVLEILRIKLVGVIPEDQSVLRASNQGEPVILDS
AADAGKAYADTVDRLLGEERPFRFIEEEKKGFLKRLFGG

>Klebsiella variicola
MARIIVVTSGKGGVGKTTSSAAIATGLAQKGKKTVVIDFDIGLRNLDLIMGCERRVVYDFVNVIQGDATLNQALIKD
KRTENLYILPASQTRDKDALTREGVDKVLEELKKMDFDFIVCDSPAGIETGALMALYFADEAIITTNPEVSSVRDSD
RILGILASKSRRAENGEEPIKEHLLLTRYNPGRVNKGDMLSMEDVLEILRINLVGVIPEDQSVLRASNQGEPVILDA
ASDAGKAYADTVERLLGEERPFRFIEEEKKGFLKRLFGG

>Cronobacter turicensis
MARIIVVTSGKGGVGKTTSSAAIATGLAQKGKKTVVIDFDIGLRNLDLIMGCERRVVYDFVNVIQGDATLNQALIRD
KRTESLYILPASQTRDKDALTREGVEKVLDELKKMEFDFIVCDSPAGIETGALMALYFADEAIITTNPEVSSVRDSD
RILGILSSKSRRAENGEAPIKEHLLLTRYNPGRVSKGDMLSMEDVLEILRIPLVGVIPEDQSVLRASNQGEPVILDD
TSDAGKAYADTVDRLMGEERPFRFIEEEKKGFLKRLFGG

>Enterobacter cloacae
MARIIVVTSGKGGVGKTTSSAAIATGLAQKGKKTVVIDFDIGLRNLDLIMGCERRVVYDFVNVIQGDATLNQALIKD
KRTENLYILPASQTRDKDALTREGVEKVLDELKKMEFDFIVCDSPAGIETGALMALYFADEAIITTNPEVSSVRDSD
RILGILASKSRRAENGEDPIKEHLLLTRYNPGRVNKGDMLSMEDVLEILRIKLVGVIPEDQSVLRASNQGEPVILDT
TADAGKAYADTVDRLLGEERPFRFIEEEKKGFLKRLFGG

>Phytobacter diazotrophicus
MARIIVVTSGKGGVGKTTSSAAIATGLAQKGKKTVVIDFDIGLRNLDLIMGCERRVVYDFVNVIQGDATLNQALIKD
KRTENLYILPASQTRDKDALTREGVEKVLDELQKMEFDFVICDSPAGIETGALMALYFADEAIITTNPEVSSVRDSD
RILGILASKSRRAENGQEPIKEHLLLTRYNPGRVNKGDMLSMEDVLEILRIKLVGVIPEDQSVLRASNQGEPVILDT
TADAGKAYADTVERLLGEDRPFRFIDEEKKGFLKRLFGG

>Kosakonia sacchari
MARIIVVTSGKGGVGKTTSSAAIATGLAQKGKKTVVIDFDIGLRNLDLIMGCERRVVYDFVNVIQGDATLNQALIKD
KRTENLYILPASQTRDKDALTREGVDKVLEDLKKMDFDFIVCDSPAGIETGALMALYFADEAIITTNPEVSSVRDSD
RILGILASKSRRAENGEEPIKEHLLLTRYNPGRVNRGDMLSMEDVLEILRIKLIGVIPEDQSVLRASNQGEPVILDT
NADAGKAYADAVERLLGEDRPFRFIEEEKKGFLKRLFGG

>Huaxiibacter chinensis
MARIIVVTSGKGGVGKTTSSAAIATGLAQKGKKTVVIDFDIGLRNLDLIMGCERRVVYDFVNVIQGDATLNQAMIKD
KRTENLYILPASQTRDKDALTREGVEKVLDELKKMEFDFVVCDSPAGIETGALMALYFADEAIITTNPEVSSVRDSD
RILGILASKSRRAENGQEPIKEHLLLTRYNPGRVNKGDMLSMEDVLEILRIKLVGVIPEDQSVLRASNQGEPVILDS
TADAGKAYADTVDRLLGEERPFRFIEEEKKGFLKRLFGG

Cellular homeostasis:

1. SodA

>Salmonella typhimurium
MSYTLPSLPYAYDALEPHFDKQTMEIHHTKHHQTYVNNANAALENLPEFASLPVEELITKLDQVPADKKTVLRNNAG
GHANHSLFWKGLKKGTTLQGDLKAAIERDFGSVDNFKAEFEKAAATRFGSGWAWLVLKGDKLAVVSTANQDSPLMGE
AISGASGFPILGLDVWEHAYYLKFQNRRPDYIKEFWNVVNWDEAAARFAAKK

>Escherichia coli
MSYTLPSLPYAYDALEPHFDKQTMEIHHTKHHQTYVNNANAALESLPEFANLPVEELITKLDQLPADKKTVLRNNAG
GHANHSLFWKGLKKGTTLQGDLKAAIERDFGSVDNFKAEFEKAAASRFGSGWAWLVLKGDKLAVVSTANQDSPLMGE
AISGASGFPIMGLDVWEHAYYLKFQNRRPDYIKEFWNVVNWDEAAARFAAKK

>Citrobacter rodentium

MSYTLPSLPYAYDALEPHFDKQTMEIHHTKHHQTYVNNANAALESLPEFASLPVEELITKLDQVPADKKTVLRNNAG
GHANHSLFWKGLKKGTTLQGDLKAAIERDFGSVEKFKEEFEKAAATRFGSGWAWLVLKGDKLAVVSTANQDSPLMGE
AISGASGFPILGLDVWEHAYYLKFQNRRPDYIKEFWNVVNWDEAAARFAAKK

>Klebsiella variicola
MSYTLPSLPYAYDALEPHFDKQTMEIHHTKHHQTYVNNANAALESLPEFANLSAEELITKLDQLPADKKTVLRNNAG
GHANHSLFWKGLKTGTTLQGDLKAAIERDFGSVDNFKAEFEKAAATRFGSGWAWLVLKGDKLAVVSTANQDSPLMGE
AISGASGFPILGLDVWEHAYYLKFQNRRPDYIKAFWDVVNWDEAAARFAAKK

>Cronobacter turicensis
MSYTLPSLPYAYDALEPHFDKQTMEIHHTKHHQTYVNNANAALESLPELANLPVEELIAKLDQVPADKKTVLRNNAG
GHANHSLFWKGLKKGTTLQGDLKAAIERDFGSVEKFKEEFEKAAATRFGSGWAWLVLKGDKLAVVSTANQDSPLMGE
AISGASGYPILGLDVWEHAYYLKFQNRRPDYIKEFWNVVNWDEAAARFASQK

>Enterobacter cloacae
MSYTLPSLPYAYDALEPHFDKQTMEIHHTKHHQTYVNNANAALESLPEFANLPVEELITKLDQLPADKKTVLRNNAG
GHANHSLFWKGLKTGTTLQGDLKAAIERDFGSVDNFKAEFEKAAATRFGSGWAWLVLKGDKLAVVSTANQDSPLMGE
AISGASGFPILGLDVWEHAYYLKFQNRRPDYIKAFWDVVNWDEAAARFAAKK

>Phytobacter diazotrophicus
MSYTLPSLPYAYDALEPHFDKQTMEIHHTKHHQTYVNNANAALESLPEFASLSAEELITKLDQLPADKKTVLRNNAG
GHANHSFFWKGLKKGTTLQGDLKAAIERDFGSVDNFKAEFEKAAATRFGSGWAWLVLKGDKLAVVSTANQDSPLMGE
AISGASGFPIVGLDVWEHAYYLKFQNRRPDYIKEFWNVVNWDEAAARFAAKK

>Kosakonia sacchari
MSYTLPALPYAYDALEPHFDKQTMEIHHTKHHQTYVNNANAALESLPEFASLPVEELITKLDQLPADKKTVLRNNAG
GHANHSLFWKGLKKGTTLQGDLKAAIERDFGSVDNFKAEFEKAAATRFGSGWAWLVLKGDKLAVVSTANQDSPLMGE
AISGASGFPIVGLDVWEHAYYLKFQNRRPDYIKEFWNVVNWDEAAARFAAKK

>Huaxiibacter chinensis
MSYTLPSLPYAYDALEPHFDKQTMEIHHTKHHQTYVNNANAALESLPEFASLPVEELITKLDQLPADKKTVLRNNAG
GHANHSLFWKGLKTGTTLQGDLKAAIERDFGSVDNFKAEFEKAAATRFGSGWAWLVLKGDKLAVVSTANQDSPLMGE
AISGASGFPIVGLDVWEHAYYLKFQNRRPDYIKAFWDVVNWDEAAARFAAKK

2. DnaK

>Salmonella typhimurium
MGKIIGIDLGTTNSCVAIMDGTQARVLENAEGDRTTPSIIAYTQDGETLVGQPAKRQAVTNPQNTLFAIKRLIGRRF
QDEEVQRDVSIMPYKIIGADNGDAWLDVKGQKMAPPQISAEVLKKMKKTAEDYLGEPVTEAVITVPAYFNDAQRQAT
KDAGRIAGLEVKRIINEPTAAALAYGLDKEVGNRTIAVYDLGGGTFDISIIEIDEVDGEKTFEVLATNGDTHLGGED
FDTRLINYLVDEFKKDQGIDLRNDPLAMQRLKEAAEKAKIELSSAQQTDVNLPYITADATGPKHMNIKVTRAKLESL
VEDLVNRSIEPLKVALQDAGLSVSDINDVILVGGQTRMPMVQKKVAEFFGKEPRKDVNPDEAVAIGAAVQGGVLTGD
VKDVLLLDVTPLSLGIETMGGVMTPLITKNTTIPTKHSQVFSTAEDNQSAVTIHVLQGERKRASDNKSLGQFNLDGI
NPAPRGMPQIEVTFDIDADGILHVSAKDKNSGKEQKITIKASSGLNEEEIQKMVRDAEANAESDRKFEELVQTRNQG
DHLLHSTRKQVEEAGDKLPADDKTAIESALNALETALKGEDKAAIEAKMQELAQVSQKLMEIAQQQHAQQQAGSADA
SANNAKDDDVVDAEFEEVKDKK

>Escherichia coli
MGKIIGIDLGTTNSCVAIMDGTTPRVLENAEGDRTTPSIIAYTQDGETLVGQPAKRQAVTNPQNTLFAIKRLIGRRF
QDEEVQRDVSIMPFKIIAADNGDAWVEVKGQKMAPPQISAEVLKKMKKTAEDYLGEPVTEAVITVPAYFNDAQRQAT
KDAGRIAGLEVKRIINEPTAAALAYGLDKGTGNRTIAVYDLGGGTFDISIIEIDEVDGEKTFEVLATNGDTHLGGED
FDSRLINYLVEEFKKDQGIDLRNDPLAMQRLKEAAEKAKIELSSAQQTDVNLPYITADATGPKHMNIKVTRAKLESL
VEDLVNRSIEPLKVALQDAGLSVSDIDDVILVGGQTRMPMVQKKVAEFFGKEPRKDVNPDEAVAIGAAVQGGVLTGD
VKDVLLLDVTPLSLGIETMGGVMTTLIAKNTTIPTKHSQVFSTAEDNQSAVTIHVLQGERKRAADNKSLGQFNLDGI
NPAPRGMPQIEVTFDIDADGILHVSAKDKNSGKEQKITIKASSGLNEDEIQKMVRDAEANAEADRKFEELVQTRNQG
DHLLHSTRKQVEEAGDKLPADDKTAIESALTALETALKGEDKAAIEAKMQELAQVSQKLMEIAQQQHAQQQTAGADA
SANNAKDDDVVDAEFEEVKDKK

>Citrobacter rodentium
MGKIIGIDLGTTNSCVAIMDGTQARVLENAEGDRTTPSIIAYTQDGETLVGQPAKRQAVTNPQNTLFAIKRLIGRRF
QDEEVQRDVSIMPYKIIGADNGDAWLDVKGQKMAPPQISAEVLKKMKKTAEDYLGEPVTEAVITVPAYFNDAQRQAT
KDAGRIAGLEVKRIINEPTAAALAYGLDKEVGNRTIAVYDLGGGTFDISIIEIDEVDGEKTFEVLATNGDTHLGGED
FDSRMINYLVEEFKKDQGIDLRNDPLAMQRLKEAAEKAKIELSSAQQTDVNLPYITADATGPKHMNIKVTRAKLESL
VEDLVNRSIEPLKVALQDAGLSVSDINDVILVGGQTRMPMVQKKVAEFFGKEPRKDVNPDEAVAIGAAVQGGVLTGD
VKDVLLLDVTPLSLGIETMGGVMTALISKNTTIPTKHSQVFSTAEDNQSAVTIHVLQGERKRASDNKSLGQFNLDGI
NPAPRGMPQIEVTFDIDADGILHVSAKDKNSGKEQKITIKASSGLNEEEIQKMVREAEANAESDRKFEELVQTRNQG
DHLLHSTRKQVEEAGDQLPADDKTAIESALSALETALKGEDKAAIEAKMQELAQVSQKLMEIAQQQHAQQQAGSADA
SANNAKDDDVVDAEFEEVKDKK

>Klebsiella variicola
MGKIIGIDLGTTNSCVAIMDGTTARVLENAEGDRTTPSIIAYTQDGETLVGQPAKRQAVTNPQNTLFAIKRLIGRRF
QDEEVQRDVSIMPYKIVAADNGDAWLDVKGTKTAPPQISAEVLKKMKKTAEDYLGEPVTEAVITVPAYFNDAQRQAT
KDAGRIAGLEVKRIINEPTAAALAYGLDKEVGNRTIAVYDLGGGTFDISIIEIDEVDGEKTFEVLATNGDTHLGGED
FDTRLINYLVDEFKKDQGIDLRNDPLAMQRLKEAAEKAKIELSSAQQTDVNLPYITADATGPKHMNIKVTRAKLESL
VEDLVNRSIEPLKVALQDAGLSVSDINDVILVGGQTRMPMVQKKVAEFFGKEPRKDVNPDEAVAIGAAVQGGVLTGD
VKDVLLLDVTPLSLGIETMGGVMTALINKNTTIPTKHSQVFSTAEDNQSAVTIHVLQGERKRASDNKSLGQFNLDGI
NPAPRGMPQIEVTFDIDADGILHVSAKDKNSGKEQKITIKASSGLNEEEIQKMVREAEANAESDRKFEELVQTRNQG
DHLLHSTRKQVEEAGDKLPADDKTAIESALTALETSLKGEDKADIEAKMQALAQASQKLMEIAQQQHAQQQAGSADA
QASNAKDDDVVDAEFEEVKDKK

>Cronobacter turicensis
MGKIIGIDLGTTNSCVAIMDGTQARVLENAEGDRTTPSIIAYTQDGETLVGQPAKRQAVTNPQNTLFAIKRLIGRRF
QDEEVQRDESIMPYKIISADNGDAWLDVKGQKMAPPQISAEVLKKMKKTAEDYLGEPVTEAVITVPAYFNDAQRQAT
KDAGRIAGLEVKRIINEPTAAALAYGLDKETGNRTIAVYDLGGGTFDISIIEIDEVDGEKTFEVLATNGDTHLGGED
FDSRLINYLVEEFKKDQGIDLRNDPLAMQRLKEAAEKAKIELSSAQQTDVNLPYITADATGPKHMNIKVTRAKLESL
VEDLVNRSIEPLKVALQDAGLSVSDINDVILVGGQTRMPMVQKKVAEFFGKEPRKDVNPDEAVAIGAAVQGGVLTGD
VKDVLLLDVTPLSLGIETMGGVMTPLITKNTTIPTKHSQVFSTAEDNQSAVTIHVLQGERKRAADNKSLGQFNLDGI
NPAPRGMPQIEVTFDIDADGILHVSAKDKNSGKEQKITIKASSGLNEDEIQKMVREAEANAEADRKFEELVQTRNQA
DHLVHSTRKQVEEAGDKLPADDKTAIESALSALEASLKGEDKADIEAKMQALAQVSQKLMEIAQQQHAQQQAGSADA
SANNAKDDDVVDAEFEEVKDKK

>Enterobacter cloacae
MGKIIGIDLGTTNSCVAIMDGTTARVLENAEGDRTTPSIIAYTQDGETLVGQPAKRQAVTNPQNTLFAIKRLIGRRF
QDEEVQRDVSIMPYKIIAADNGDAWLDVKGTKTAPPQISAEVLKKMKKTAEDYLGEPVTEAVITVPAYFNDAQRQAT
KDAGRIAGLEVKRIINEPTAAALAYGLDKEVGNRTIAVYDLGGGTFDISIIEIDEVDGEKTFEVLATNGDTHLGGED
FDTRLINYLVDEFKKDQGIDLRNDPLAMQRLKEAAEKAKIELSSAQQTDVNLPYITADATGPKHMNIKVTRAKLESL
VEDLVNRSIEPLKVALQDAGLSVSDINDVILVGGQTRMPMVQKKVAEFFGKEPRKDVNPDEAVAIGAAVQGGVLTGD
VKDVLLLDVTPLSLGIETMGGVMTALISKNTTIPTKHSQVFSTAEDNQSAVTIHVLQGERKRASDNKSLGQFNLDGI
NPAPRGMPQIEVTFDIDADGILHVSAKDKNSGKEQKITIKASSGLNEEEIQKMVREAEANAESDRKFEELVQTRNQG
DHLLHSTRKQVEEAGDKLPAEDKTAIEAALSALETSLKGEDKADIEAKMQELAQASQKLMEIAQQQHAQQQAGADAS
ANNAKDDDVVDAEFEEVKDKK

>Phytobacter diazotrophicus
MGKIIGIDLGTTNSCVAIMDGTTARVLENAEGDRTTPSIIAYTQDGETLVGQPAKRQAVTNPQNTLFAIKRLIGRRF
QDEEVQRDVSIMPYKIIAADNGDAWLDVKGTKTAPPQISAEVLKKMKKTAEDYLGEPVTEAVITVPAYFNDAQRQAT
KDAGRIAGLEVKRIINEPTAAALAYGLDKEVGNRTIAVYDLGGGTFDISIIEIDEVDGEKTFEVLATNGDTHLGGED
FDSRLINYLVEEFKKDQGIDLRNDPLAMQRLKEAAEKAKIELSSAQQTDVNLPYITADATGPKHMNIKVTRAKLESL
VEDLVNRSIEPLKVALQDAGLSVSDVQDVILVGGQTRMPMVQKKVAEFFGKEPRKDVNPDEAVAIGAAVQGGVLTGD
VKDVLLLDVTPLSLGIETMGGVMTALINKNTTIPTKHSQVFSTAEDNQSAVTIHVLQGERKRASDNKSLGQFNLDGI
NPAPRGMPQIEVTFDIDADGILHVSAKDKNSGKEQKITIKASSGLNEEEIQKMVREAEANAESDRKFEELVQTRNQG
DHLLHSTRKQVEEAGDKLPAEDKTAIEAALTALETSLKGEDKADIEAKMQALAQASQKLLEIAQQQHAQQQAGGADA
SANNAKDDDVVDAEFEEVKDKK

>Kosakonia sacchari

MGKIIGIDLGTTNSCVAIMDGAQARVLENAEGDRTTPSIIAYTQDGETLVGQPAKRQAVTNPQNTLFAIKRLIGRRF
QDEEVQRDESIMPYKIIAADNGDAWIDVKGTKMAPPQISAEVLKKMKKTAEDYLGEPVTEAVITVPAYFNDAQRQAT
KDAGRIAGLEVKRIINEPTAAALAYGLDKETGNRTIAVYDLGGGTFDISIIEIDEVDGEKTFEVLATNGDTHLGGED
FDSRLINYLVEEFKKDQGIDLRNDPLAMQRLKEAAEKAKIELSSAQQTDVNLPYITADATGPKHMNIKVTRAKLESL
VEDLVNRSIEPLKVALQDAGLSVSDINDVILVGGQTRMPMVQKKVAEFFGKEPRKDVNPDEAVAIGAAVQGGVLTGE
VKDVLLLDVTPLSLGIETMGGVMTALINKNTTIPTKHSQVFSTAEDNQSAVTIHVLQGERKRASDNKSLGQFNLDGI
NPAPRGMPQIEVTFDIDADGILHVSAKDKNSGKEQKITIKASSGLNEDEIQKMVRDAEANAEADRKFEELVQTRNQA
DHLLHSTRKQVEEAGDKLPAEDKTAIDAALSELETALKGEDKADIEAKMQALAQASQKLLEIAQQQHAQQQAGADAS
ANNAKDDDVVDAEFEEVKDKK

>Huaxiibacter chinensis
MGKIIGIDLGTTNSCVAIMDGTTARVLENAEGDRTTPSIIAYTQDGETLVGQPAKRQAVTNPQNTLFAIKRLIGRRF
QDEEVQRDEAIMPYKIIGADNGDAWIDVKGQKMAPPQISAEVLKKMKKTAEDYLGEPVTEAVITVPAYFNDAQRQAT
KDAGRIAGLEVKRIINEPTAAALAYGLDKEVGNRTIAVYDLGGGTFDISIIEIDEVDGEKTFEVLATNGDTHLGGED
FDSRMINYLVDEFKKDQGIDLRNDPLAMQRLKEAAEKAKIELSSAQQTDVNLPYITADASGPKHMNIKVTRAKLESL
VEDLVNRSIEPLKVALQDAGLSVSDIQDVILVGGQTRMPMVQKKVAEFFGKEPRKDVNPDEAVAIGAAVQGGVLTGE
VKDVLLLDVTPLSLGIETMGGVMTPLISKNTTIPTKHSQVFSTAEDNQSAVTIHVLQGERKRAADNKDLGQFNLDGI
SPAPRGMPQIEVTFDIDADGILHVSAKDKNSGKEQKITIKASSGLNEEEIEKMVRDAEANAESDRKFEELVQTRNQG
DHLLHSTRKQVEEVGEQLPADDKAAIETALSALETSLKGEDKADIEAKMQELAQASQKLMEIAQQQHAQQQAGAGAD
ASQNNAKDDDVVDAEFEEVKDKK

3. UspA

>Salmonella typhimurium
MAYKHILIAVDLSPESKVLVEKAVSMARPYNAKISLIHVDVNYSDLYTGLIDVNLGDMQKRISEETHHALTELSTNA
GYPITETLSGSGDLGQVLVDAIKKYDMDLVVCGHHQDFWSKLMSSARQLINTVHVDMLIVPLRDEEE

>Escherichia coli
MAYKHILIAVDLSPESKVLVEKAVSMARPYNAKVSLIHVDVNYSDLYTGLIDVNLGDMQKRISEETHHALTELSTNA
GYPITETLSGSGDLGQVLVDAIKKYDMDLVVCGHHQDFWSKLMSSARQLINTVHVDMLIVPLRDEEE

>Citrobacter rodentium
MAYKHILIAVDLSPESKVLVEKAVSMARPYNAKVSLIHVDVNYSDLYTGLIDVNLGDMQKRISEETHHALTELSTNA
GYPITETLSGSGDLGQVLVDAIKKYDMDLVVCGHHQDFWSKLMSSARQLINTVHVDMLIVPLRDEEE

>Klebsiella variicola
MAYKHILIAVDLSPESKVLVEKAVSMARPYNAKVSLIHVDVNYSDLYTGLIDVNLGDMQKRISEETHHALTELSTNA
GYPITETLSGSGDLGQVLVDAIKKYDMDLVVCGHHQDFWSKLMSSARQLINTVHVDMLIVPLRDEEDE

>Cronobacter turicensis
MAYKHILIAVDLSPESKVLVDKAVSMARPYNAKISLIHVDVNYSDLYTGLIDVNLGDMQKRISEETHHALTELSTNA
GYPITETLSGSGDLGQVLVDAIKKYDVDLVVCGHHQDFWSKLMSSARQLINTVHIDMLIVPLRDDEEA

>Enterobacter cloacae
MAYKHILIAVDLSPESKVLVDKAVSMARPYNAKVSLIHVDVNYSDLYTGLIDVNLGDMQKRISEETHHALSELSTNA
GYPITETLSGSGDLGQVLVDAIKKYDMDLVVCGHHQDFWSKLMSSARQLINTVHVDMLIVPLRDEEDE

>Phytobacter diazotrophicus
MAYKHILIAVDLSPESKLLVEKAVSMARPYNAKVSLIHVDVNYSDLYTGLIDVNLGDMQKRISDETHHALTELSTNA
GYPITETLSGSGDLGQVLVDAIKKYDMDLVVCGHHQDFWSKLMSSARQLINTVHVDMLIVPLKDEDEE

>Kosakonia sacchari
MAYKHILIAVDLSPESKLLVEKAVSMARPYNAKISLIHVDVNYSDLYTGLIDVNLGDMQKRISDETHQALTELSTNA
GYPITETLSGSGDLGQVLVDAIKKYDMDLVVCGHHQDFWSKLMSSARQLINTVHVDMLIVPLRDEEED

>Huaxiibacter chinensis
MAYKHILIAVDLSPESKVLVDKAVSMARPYNAKVSLIHVDVNYSDLYTGLIDVNLGDMQKRISEETHHALSELSTNA
GYPITETLSGSGDLGQVLVDAIKKYDMDLVVCGHHQDFWSKLMSSARQLINTVHVDMLIVPLRDEEDE

4. CspD

>Salmonella typhimurium
METGTVKWFNNAKGFGFICPEGGGEDIFAHYSTIQMDGYRTLKAGQSVRFDVHQGPKGNHASVIVPIEAEAVA

>Escherichia coli
MEKGTVKWFNNAKGFGFICPEGGGEDIFAHYSTIQMDGYRTLKAGQSVQFDVHQGPKGNHASVIVPVEVEAAVA

>Citrobacter rodentium
METGTVKWFNNAKGFGFICPEGGGEDIFAHYSTIQMDGYRTLKAGQPVQFDVHQGPKGNHASVIVPIEAEAVA

>Klebsiella variicola
MEMGTVKWFNNAKGFGFICPEGGGEDIFAHYSTIQMDGYRTLKAGQAVRFDVHQGPKGNHASVIVPVEAETAA

>Cronobacter turicensis
METGTVKWFNNAKGFGFICPEGGGEDIFAHYSTIQMDGYRTLKAGQVVRFDVHQGPKGNHASVIVPLEAEVAPAVA

>Enterobacter cloacae
MEMGTVKWFNNAKGFGFICPEGGGEDIFAHYSTIQMDGYRTLKAGQSVRFDVHQGPKGNHASLIVPIEAETVA

>Phytobacter diazotrophicus
METGTVKWFNNAKGFGFICPEGGGEDIFAHYSTIQMDGYRTLKAGQTVRFDVHQGPKGNHASLIVPVEAEAVA

>Kosakonia sacchari
METGTVKWFNNAKGFGFICPEGGGEDIFAHYSTIQMDGYRTLKAGQTVRFDVHQGPKGNHASVIVPVEVEAMA

>Huaxiibacter chinensis
METGIVKWFNNAKGFGFICPEGGGEDIFAHYSTIQMDGYRTLKAGQSVRFDVHQGPKGNHASLIVPLEAETVA

5. Lon

>Salmonella typhimurium
MNPERSERIEIPVLPLRDVVVYPHMVIPLFVGREKSIRCLEAAMDHDKKIMLVAQKEASTDEPGVNDLFTVGTVASI
LQMLKLPDGTVKVLVEGLQRARISALSDNGEHFSAKAEYLDSPAIDEREQEVLVRTAISQFEGYIKLNKKIPPEVLT
SLNSIDDPARLADTIAAHMPLKLADKQSVLEMSDVNERLEYLMAMMESEIDLLQVEKRIRNRVKKQMEKSQREYYLN
EQMKAIQKELGEMDDAPDENEALKRKIDAAKMPKEAKEKAEAELQKLKMMSPMSAEATVVRGYIDWMVQVPWNARSK
VKKDLRQAQEILDTDHYGLERVKDRILEYLAVQSRVNKIKGPILCLVGPPGVGKTSLGQSIAKATGRKYIRMALGGV
RDEAEIRGHRRTYIGSMPGKLIQKMAKVGVKNPLFLLDEIDKMSSDMRGDPASALLEVLDPEQNVAFSDHYLEVDYD
LSDVMFVATSNSMNIPAPLLDRMEVIRLSGYTEDEKLNIAKRHLLPKQIERNALKKGELTVDDSAIIGIIRYYTREA
GVRSLEREISKLCRKAVKQLLLDKSLKHIEINGDNLHDYLGVQRFDYGRADSENRVGQVTGLAWTEVGGDLLTIETA
CVPGKGKLTYTGSLGEVMQESIQAALTVVRARAEKLGINPDFYEKRDIHVHVPEGATPKDGPSAGIAMCTALVSCLT
GNPVRADVAMTGEITLRGQVLPIGGLKEKLLAAHRGGIKTVLIPFENKRDLEEIPDNVIADLDIHPVKRIEEVLTLA
LQNEPSGMQVVTAK

>Escherichia coli
MNPERSERIEIPVLPLRDVVVYPHMVIPLFVGREKSIRCLEAAMDHDKKIMLVAQKEASTDEPGVNDLFTVGTVASI
LQMLKLPDGTVKVLVEGLQRARISALSDNGEHFSAKAEYLESPTIDEREQEVLVRTAISQFEGYIKLNKKIPPEVLT
SLNSIDDPARLADTIAAHMPLKLADKQSVLEMSDVNERLEYLMAMMESEIDLLQVEKRIRNRVKKQMEKSQREYYLN
EQMKAIQKELGEMDDAPDENEALKRKIDAAKMPKEAKEKAEAELQKLKMMSPMSAEATVVRGYIDWMVQVPWNARSK
VKKDLRQAQEILDTDHYGLERVKDRILEYLAVQSRVNKIKGPILCLVGPPGVGKTSLGQSIAKATGRKYVRMALGGV
RDEAEIRGHRRTYIGSMPGKLIQKMAKVGVKNPLFLLDEIDKMSSDMRGDPASALLEVLDPEQNVAFSDHYLEVDYD
LSDVMFVATSNSMNIPAPLLDRMEVIRLSGYTEDEKLNIAKRHLLPKQIERNALKKGELTVDDSAIIGIIRYYTREA

GVRGLEREISKLCRKAVKQLLLDKSLKHIEINGDNLHDYLGVQRFDYGRADNENRVGQVTGLAWTEVGGDLLTIETA
CVPGKGKLTYTGSLGEVMQESIQAALTVVRARAEKLGINPDFYEKRDIHVHVPEGATPKDGPSAGIAMCTALVSCLT
GNPVRADVAMTGEITLRGQVLPIGGLKEKLLAAHRGGIKTVLIPFENKRDLEEIPDNVIADLDIHPVKRIEEVLTLA
LQNEPSGMQVVTAK

>Citrobacter rodentium
MNPERSERIEIPVLPLRDVVVYPHMVIPLFVGREKSIRCLEAAMDHDKKIMLVAQKEASTDEPGVNDLFTVGTVASI
LQMLKLPDGTVKVLVEGLQRARISALSDNGEHFSAKAEYLDSPAIDEREQEVLVRTAISQFEGYIKLNKKIPPEVLT
SLNSIDDPARLADTIAAHMPLKLADKQSVLEMSDVNERLEYLMAMMESEIDLLQVEKRIRNRVKKQMEKSQREYYLN
EQMKAIQKELGEMDDAPDENEALKRKIDAAKMPKEAKEKAEAELQKLKMMSPMSAEATVVRGYIDWMVQVPWNARSK
VKKDLRQAQEILDTDHYGLERVKDRILEYLAVQSRVNKIKGPILCLVGPPGVGKTSLGQSIAKATGRKYIRMALGGV
RDEAEIRGHRRTYIGSMPGKLIQKMAKVGVKNPLFLLDEIDKMSSDMRGDPASALLEVLDPEQNVAFSDHYLEVDYD
LSDVMFVATSNSMNIPAPLLDRMEVIRLSGYTEDEKLNIAKRHLLPKQIERNALKKGELTVEDSAIIGIIRYYTREA
GVRSLEREISKLCRKAVKQLLLDKSLRQIVIDGDNLHDYLGVQRFDYGRADNENRVGQVTGLAWTEVGGDLLTIETA
CVPGKGKLTYTGSLGEVMQESIQAALTVVRARAEKLGINPDFYEKRDIHVHVPEGATPKDGPSAGIAMCTALVSCLT
GNPVRADVAMTGEITLRGQVLPIGGLKEKLLAAHRGGIKTVLIPYENKRDLEEIPDNVIADLDIHPVKRIEEVLTLA
LQNEPSGMQVVTAK

>Klebsiella variicola
MNPERSERIEIPVLPLRDVVVYPHMVIPLFVGREKSIRCLEAAMDHDKKIMLVAQKEASTDEPGVNDLFTVGTVASI
LQMLKLPDGTVKVLVEGLQRARISALSDNGEHFSAKAEYLDSPAIDEREQEVLVRTAISQFEGYIKLNKKIPPEVLT
SLNSIDDPARLADTIAAHMPLKLADKQSVLEMSDVNERLEYLMAMMESEIDLLQVEKRIRNRVKKQMEKSQREYYLN
EQMKAIQKELGEMDDAPDENEALKRKIDAAKMPKEAKEKTEAELQKLKMMSPMSAEATVVRGYIDWMVHVPWNARSK
VKKDLRQAQEILDTDHYGLERVKDRILEYLAVQSRVNKIKGPILCLVGPPGVGKTSLGQSIAKATGRKYVRMALGGV
RDEAEIRGHRRTYIGSMPGKLIQKMAKVGVKNPLFLLDEIDKMSSDMRGDPASALLEVLDPEQNVAFNDHYLEVDYD
LSDVMFVATSNSMNIPAPLLDRMEVIRLSGYTEDEKLNIAKRHLLPKQIERNALKKGELTVDDSAIIGIIRYYTREA
GVRSLEREISKLCRKAVKQLLLDKSLKHIEINGDNLHDYLGVQRFDYGRADSENRVGQVTGLAWTEVGGDLLTIETA
CVPGKGKLTYTGSLGEVMQESIQAALTVVRSRADKLGINADFYEKRDIHVHVPEGATPKDGPSAGIAMCTALVSCLT
GNPVRADVAMTGEITLRGQVLPIGGLKEKLLAAHRGGIKTVLIPDENKRDLEEIPDNVIADLDIHPVKRIEEVLTLA
LQNEPFGMQVVTAK

>Cronobacter turicensis
MNPERSERIEIPVLPLRDVVVYPHMVIPLFVGREKSIRCLEAAMDNDKKVMLVAQKEASTDEPGVNDLFTVGTVASI
LQMLKLPDGTVKVLVEGLQRARITTLSDNGDHFAAKAEYLESPAIDEREQEVLVRTAISQFEGYIKLNKKIPPEVLT
SLNSIDDPARLADTIAAHMPLKLSDKQSVLEMSDINERLEYLMAMMESEIDLLQVEKRIRNRVKKQMEKSQREYYLN
EQMKAIQKELGEMDDAPDENEALKRKIDAAKMPKEAKEKAEAELQKLKMMSPMSAEATVVRGYIDWMVQVPWNARSK
VKKDLRQAQEILDTDHYGLERVKDRILEYLAVQSRVNKIKGPILCLVGPPGVGKTSLGQSIAKATGRKYVRMALGGV
RDEAEIRGHRRTYIGSMPGKLIQKMAKVGVKNPLFLLDEIDKMSSDMRGDPASALLEVLDPEQNVAFNDHYLEVDYD
LSDVMFVATSNSMNIPAPLLDRMEVIRLSGYTEDEKLNIAKRHLLPKQIERNALKENEIDVDDSAIIGIIRYYTREA
GVRSLEREISKLCRKAVKQLLLDKSLKRIEITGENLKDFLGVQRFDYGRADSENRVGQVTGLAWTEVGGDLLTIETA
CVPGKGKLTYTGSLGEVMQESIQAALTVVRARAEKLGINSDFYEKRDIHVHVPEGATPKDGPSAGIAMCTALVSCLT
GNPVRADVAMTGEITLRGQVLPIGGLKEKLLAAHRGGIKTVLIPDENKRDLEEIPENVIADLDIHPVKRIEEVLTLA
LQNAPYGMQVATAK

>Enterobacter cloacae
MNPERSERIEIPVLPLRDVVVYPHMVIPLFVGREKSIRCLEAAMDHDKKIMLVAQKEASTDEPGVNDLFTVGTVASI
LQMLKLPDGTVKVLVEGLQRARITTLSDNGEHFSAKAEYLDSPQLDEREQEVLVRTAISQFEGYIKLNKKIPPEVLT
SLNSIDDPARLADTIAAHMPLKLADKQSVLEMSDVNERLEYLMAMMESEIDLLQVEKRIRNRVKKQMEKSQREYYLN
EQMKAIQKELGEMDDAPDENEALKRKIDAAKMPKEAKEKAEAELQKLKMMSPMSAEATVVRGYIEWMVQVPWNARSK
VKKDLRQAQEILDTDHYGLERVKDRILEYLAVQSRVNKLKGPILCLVGPPGVGKTSLGQSIAKATGRKYIRMALGGV
RDEAEIRGHRRTYIGSMPGKLIQKMAKVGVKNPLFLLDEIDKMSSDMRGDPASALLEVLDPEQNVAFSDHYLEVDYD
LSDVMFVATSNSMNIPAPLLDRMEVIRLSGYTEDEKLNIAKQHLLPKQIERNALKANELTVEDSAIIGIIRYYTREA
GVRSLEREISKLCRKAVKQLLLDKSLKHITINGDNLHAYLGVQRFDYGRADNENRVGQVTGLAWTEVGGDLLTIETA
CVPGKGKLTYTGSLGEVMQESIQAALTVVRARAEKLGINPDFYEKRDIHVHVPEGATPKDGPSAGIAMCTALVSCLT
GNPVRADVAMTGEITLRGQVLPIGGLKEKLLAAHRGGIKTVLIPYENKRDLEEIPDNVIADLQIHPVKRIEEVLTLA
LQNEPSGMQVVTAK

>Phytobacter diazotrophicus
MNPERSERIEIPVLPLRDVVVYPHMVIPLFVGREKSIRCLEAAMDHDKKIMLVAQKEASTDEPGVNDLFTVGTVASI
LQMLKLPDGTVKVLVEGLQRARITTLSDNGEHFTAKAEYLDSPAIDEREQEVLVRTAISQFEGYIKLNKKIPPEVLT
SLNSIDDPARLADTIAAHMPLKLADKQSVLEMSDVNERLEYLMAMMESEIDLLQVEKRIRNRVKKQMEKSQREYYLN
EQMKAIQKELGEMDDAPDENEALKRKIDAAKMPKEAKEKAEAELQKLKMMSPMSAEATVVRGYIEWMIQVPWYARSK
VKKDLRQAQEVLDTDHYGLERVKDRILEYLAVQSRMNKLKGPILCLVGPPGVGKTSLGQSIAKATGRKYIRMALGGV
RDEAEIRGHRRTYIGSMPGKLIQKMAKVGVKNPLFLLDEIDKMSSDMRGDPASALLEVLDPEQNVAFSDHYLEVDYD
LSDVMFVATSNSMNIPAPLLDRMEVIRLSGYTEDEKLNIAKQHLLTKQIERNALKPTELTVDDSAIVGIIRYYTREA
GVRSLEREISKLCRKAVKQLLMDPTLKHIEINGDNLHDYLGVQRFDYGRADDENRVGQVTGLAWTEVGGDLLTIETA
CVPGKGKLTYTGSLGEVMQESIQAALTVVRARAEKLGINGDFYEKRDIHVHVPEGATPKDGPSAGIAMCTALVSCLT
GNPVRADVAMTGEITLRGQVLPIGGLKEKLLAAHRGGIKTVLIPYENKRDLEEIPENVIADLDIHPVKRIDEVLALA
LQNEPFGMQVATVK

>Kosakonia sacchari
MNPERSERIEIPVLPLRDVVVYPHMVIPLFVGREKSIRCLEAAMDHDKKIMLVAQKEASTDEPGVNDLFTVGTVASI
LQMLKLPDGTVKVLVEGLQRARITTLADNGEHFSAKAEYLDSPAIDEREQEVLVRTAISQFEGYIKLNKKIPPEVLT
SLNSIDDPARLADTIAAHMPLKLADKQSVLEMSDVNERLEYLMAMMESEIDLLQVEKRIRNRVKKQMEKSQREYYLN
EQMKAIQKELGEMDDAPDENEALKRKIDASKMPKEAKEKAESELQKLKMMSPMSAEATVVRGYIEWMVQVPWNARSK
VKKDLRQAQEILDTDHYGLERVKDRILEYLAVQSRMNKLKGPILCLVGPPGVGKTSLGQSIAKATGRKYIRMALGGV
RDEAEIRGHRRTYIGSMPGKLIQKMAKVGVKNPLFLLDEIDKMSSDMRGDPASALLEVLDPEQNVAFSDHYLEVDYD
LSDVMFVATSNSMNIPAPLLDRMEVIRLSGYTEDEKLNIAKRHLLTKQIERNALKPNELTVDDSAIVGIIRYYTREA
GVRGLEREISKLCRKAVKQLLLDPSLKHIEINGENLHDYLGVQRFDYGRADDENRVGQVTGLAWTEVGGDLLTIETA
CVPGKGKLTYTGSLGEVMQESIQAALTVVRARAEKLGINGDFYEKRDIHVHVPEGATPKDGPSAGIAMCTALVSCLT
GNPVRADVAMTGEITLRGQVLPIGGLKEKLLAAHRGGIKTVLIPYENKRDLEEIPDNVVADLDIHPVKRIEEVLALA
LQNEPFGMQVVTAK

>Huaxiibacter chinensis
MNPERSERIEIPVLPLRDVVVYPHMVIPLFVGREKSIRCLEAAMDHDKKIMLVAQKEASTDEPGVNDLFTVGTVASI
LQMLKLPDGTVKVLVEGLQRARITTLSDDGEHFSAKAEYLESPQLDEREQEVLVRTAISQFEGYIKLNKKIPPEVLT
SLNSIDDPARLADTIAAHMPLKLADKQSVLEMSDVNERLEYLMAMMESEIDLLQVEKRIRNRVKKQMEKSQREYYLN
EQMKAIQKELGEMDDAPDENEALKRKIDAAKMPKEAKEKAEAELQKLKMMSPMSAEATVVRGYIEWMVQVPWNARSK
VKKDLRQAQEILDTDHYGLERVKDRILEYLAVQSRVNKLKGPILCLVGPPGVGKTSLGQSIAKATGRKYIRMALGGV
RDEAEIRGHRRTYIGSMPGKLIQKMAKVGVKNPLFLLDEIDKMSSDMRGDPASALLEVLDPEQNVAFSDHYLEVDYD
LSDVMFVATSNSMNIPAPLLDRMEVIRLSGYTEDEKLNIAKRHLLSKQIERNALKESEITVEDSAIISIIRYYTREA
GVRSLEREISKLCRKAVKQLLLDKSLKHIVINGDNLHEYLGVQRFDYGRADNENRVGQVTGLAWTEVGGDLLTIETA
CVPGKGKLTYTGSLGEVMQESIQAALTVVRARAEKLGINPDFYEKRDIHVHVPEGATPKDGPSAGIAMCTALVSCLT
GNPVRADVAMTGEITLRGLVLPIGGLKEKLLAAHRGGIKTVLIPHENKRDLEEIPDNVIADLDIHPVKRIEEVLTLA
LQNEPSGMQVVTAK

Metabolism:

1. PurF

>Salmonella typhimurium
MCGIVGIAGVMPVNQSIYDALTVLQHRGQDAAGIITIDANNCFRLRKANGLVNDIFEARHMQRLQGNMGIGHVRYPT
AGSSSASEAQPFYVNSPYGITLAHNGNLTNAHELRKKLFEEKRRHINTTSDSEILLNIFASELDNFRHYPLEADNIF
AAIAATNRQIRGAYACVAMIIGHGMVAFRDPHGIRPLVLGKRDVGDGRTEYMVASESVALDTLGFEFLRDVAPGEAI
YITEKGQLFTRQCADNPVSNPCLFEYVYFARPDSFIDKISVYSARVNMGTKLGEKIAREWEDLDIDVVIPIPETSCD
IALEIARILGKPYRQGFVKNRYVGRTFIMPGQQLRRKSVRRKLNANRAEFRDKNVLLVDDSIVRGTTSEQIIEMARE
AGAKKVYLASAAPEIRFPNVYGIDMPTANELIAHGREVDEIRQIIGADGLIFQDLNDLIEAVRAENPDIQQFECSVF
NGIYVTKDVDQQYLDFLDSLRNDDAKAVLFQNEMENLEMHNEG

>Escherichia coli
MCGIVGIAGVMPVNQSIYDALTVLQHRGQDAAGIITIDANNCFRLRKANGLVSDVFEARHMQRLQGNMGIGHVRYPT
AGSSSASEAQPFYVNSPYGITLAHNGNLTNAHELRKKLFEEKRRHINTTSDSEILLNIFASELDNFRHYPLEADNIF

AAIAATNRLIRGAYACVAMIIGHGMVAFRDPNGIRPLVLGKRDIDENRTEYMVASESVALDTLGFDFLRDVAPGEAI
YITEEGQLFTRQCADNPVSNPCLFEYVYFARPDSFIDKISVYSARVNMGTKLGEKIAREWEDLDIDVVIPIPETSCD
IALEIARILGKPYRQGFVKNRYVGRTFIMPGQQLRRKSVRRKLNANRAEFRDKNVLLVDDSIVRGTTSEQIIEMARE
AGAKKVYLASAAPEIRFPNVYGIDMPSATELIAHGREVDEIRQIIGADGLIFQDLNDLIDAVRAENPDIQQFECSVF
NGVYVTKDVDQGYLDFLDTLRNDDAKAVQRQNEVENLEMHNEG

>Citrobacter rodentium
MCGIVGIAGVMPVNQSIYDALTVLQHRGQDAAGIITIDANNCFRLRKANGLVSDVFEARHMQRMQGNMGIGHVRYPT
AGSSSASEAQPFYVNSPYGITLAHNGNLTNAHELRKKLFEEKRRHINTTSDSEILLNIFASELDNFRHYPLEADNIF
AAIAATNRQIRGAYACVAMIIGHGMIAFRDPNGIRPLVLGKRDIGDGRTEYMVASESVALDTLGFEFLRDVAPGEAV
YITEKGQLFTRQCAENPVSNPCLFEYVYFARPDSFIDKISVYSARVNMGTKLGEKIAREWEDLDIDVVIPIPETSCD
IALEIARILGKPYRQGFVKNRYVGRTFIMPGQQLRRKSVRRKLNANRAEFRDKNVLLVDDSIVRGTTSEQIIEMARE
AGAKKVYLASAAPEIRFPNVYGIDMPTTNELIAHGREVDEIRQIIGADGLIFQDLNDLIEAVRAENPDIQQFECSVF
NGVYVTKDVDQQYLDFLDSLRNDDAKAVQRQNEVENLEMHNEG

>Klebsiella variicola
MCGIVGIAGVMPVNQSIYDALTVLQHRGQDAAGIITIDANNCFRLRKANGLVSDVFEARHMQRMQGNMGIGHVRYPT
AGSSSASEAQPFYVNSPYGITLAHNGNLTNAHELRKKLFEEKRRHINTTSDSEILLNIFASELDNFRHYPLEADNIF
AAIAATNRLIRGAYACVAMIIGHGMVAFRDPNGIRPLVLGKRDVGDGRTEYMVASESVALDTLGFEFLRDVAPGEAV
YITEKGQLYTRQCADNPVSNPCLFEYVYFARPDSFIDKISVYSARVNMGTKLGEKIAREWEDLDIDVVIPIPETSCD
IALEIARILDKPYRQGFVKNRYVGRTFIMPGQQLRRKSVRRKLNANRAEFRDKNVLLVDDSIVRGTTSEQIIEMARE
AGAKKVYLASAAPEIRFPNVYGIDMPTANELIAHGREVDEIRQIIGADGLIFQDLNDLIDAVRAENPDIQQFECSVF
NGVYVTRDVDQQYLDYLDSLRNDDAKAVQLQNEVENLEMHNEG

>Cronobacter turicensis
MCGIVGIAGVMPVNQSIYDALSVLQHRGQDAAGIITIDANNCFRLRKANGLVNDVFEARHMQRLQGNMGIGHVRYPT
AGSSSASEAQPFYVNSPYGITLAHNGNLTNAHELRKMLFEEKRRHINTTSDSEILLNIFASELDNFRHYPLEADNIF
AAIAAMNRLIRGAYACVAMIIGHGMVAFRDPNGIRPLVLGKRDAGNGRAEYMVASESVALDTLGFEFLRDVAPGEAV
YISEKGQLFTRQCADNPVSNPCLFEYVYFARPDSFIDKISVYSARVEMGKKLGEKIAREWEDLDIDVVIPIPETSCD
IALEIARILNKPYRQGFVKNRYVGRTFIMPGQHLRRKSVRRKLNANRAEFRDKNVLLVDDSIVRGTTSEQIIEMARE
AGAKKVYLASAAPEIRFPNVYGIDMPSANELIAHGREVDEIRQIIGADGLIFQDLDDLIEAVRAENPDIQQFECSVF
NGVYVTKDVDHQYLEYLESLRNDDAKAVMRQNEVENLEMHNEG

>Enterobacter cloacae
MCGIVGIAGFMPVNQSIYDALTVLQHRGQDAAGIITIDANNCFRLRKANGLVNDVFEARHMQRLQGNLGIGHVRYPT
AGSSSASEAQPFYVNSPYGITLAHNGNLTNAHELRKKLFEEKRRHINTTSDSEILLNVFASELDNFRHYPLEADNIF
AAVAATNRQIRGAYACVAMIIGHGMVAFRDPNGIRPLVLGKRDLGDGRTEYMVASESVALDTLGFEFLRDVAPGEAV
YITEKGQLFTRQCADNPVSNPCLFEYVYFARPDSFIDKISVYSARVNMGTKLGEKIAREWDDLDIDVVIPIPETSCD
IALEIARILDKPYRQGFVKNRYVGRTFIMPGQQLRRKSVRRKLNANRAEFRDKNVLLVDDSIVRGTTSEQIIEMARE
AGAKKVYLASAAPEIRFPNVYGIDMPTANELIAHGREVDEIRQIIGADGLIFQDLNDLIDAVRAENPDIQQFECSVF
NGVYVTKDVDQQYLDYLDSLRNDDAKAVQLQNDLESLEMHNEG

>Phytobacter diazotrophicus
MCGIVGIAGVMPVNQSIYDALTVLQHRGQDAAGIITIDANNCFRLRKANGLVNDVFEARHMQRLQGNMGIGHVRYPT
AGSSSASEAQPFYVNSPYGITLAHNGNLTNAHELRKKLFEEKRRHINTTSDSEILLNIFASELDNFRHYPLEADNIF
AAVAATNRQIRGAYACVAMIIGHGMVAFRDPNGIRPLVLGKRDIGDGRTEYMVASESVALDTLGFEFLRDIAPGEAV
YITEKGQLFTRQCADNPVSNPCLFEYVYFARPDSFIDKISVYSARVNMGTKLGEKIAREWEDLDIDVVIPIPETSCD
IALEIARILGKPYRQGFVKNRYVGRTFIMPGQQLRRKSVRRKLNANRAEFRDKNVLLVDDSIVRGTTSEQIIEMARE
AGAKKVYLASAAPEIRFPNVYGIDMPTANELIAHGREVDEIRQIIGADGLIFQDLTDLIDAVRAENPDIQQFECSVF
NGIYVTKDVDQGYLDYLDSLRNDDAKAVQMQNDLESLEMHNEG

>Kosakonia sacchari
MCGIVGIAGFMPVNQSIYDALTVLQHRGQDAAGIITIDANNCFRLRKANGMVSDVFEARHMQRMQGNMGIGHVRYPT
AGSSSASEAQPFYVNSPYGITLAHNGNLTNAHELRKKLFEEKRRHINTTSDSEILLNIFASELDNFRHYPLEADNIF
AAIAATNRQIRGAYACVAMIIGHGMVAFRDPNGIRPLVLGKREIGDGRTEYMVASESVALDTLGFEFLRDIAPGEAV
YITEKGQLFSRQCADNPVSNPCLFEYVYFARPDSFIDKISVYSARVNMGTKLGEKIAREWEDLDIDVVIPIPETSCD
IALEIARILGKPYRQGFVKNRYVGRTFIMPGQQLRRKSVRRKLNANRAEFRDKNVLLVDDSIVRGTTSEQIIEMARE

AGAKKVYLASAAPEIRFPNVYGIDMPTANELIAHGREVDEIRQIIGADGLIFQDLNDLIDAVRAENPDIQQFECSVF
NGVYVTKDVDQQYLDYLDSLRNDDAKAVQLQNEVESLEMHNEG

>Huaxiibacter chinensis
MCGIVGIAGFMPVNQSIYDALSVLQHRGQDAAGIITIDANNCFRLRKANGLVNDVFEARHMQRLQGNMGIGHVRYPT
AGSSSASEAQPFYVNSPYGITLAHNGNLTNAHELRKKLFEEKRRHINTTSDSEILLNIFASELDNFRHYPLEADNIF
AAVAATNRQIRGAYACVAMIIGHGMVAFRDPNGIRPLVLGKRDLGDGRTEYMVASESVALDTLGFEFLRDVAPGEAV
YIAEKGQLFTRQCADNPVSNPCLFEYVYFARPDSFIDKISVYSARVTMGTKLGEKIAREWDDLDIDVVIPIPETSCD
IALEIARILDKPYRQGFVKNRYVGRTFIMPGQQLRRKSVRRKLNANRAEFRDKNVLLVDDSIVRGTTSEQIIEMARE
AGAKKVYLASAAPEIRFPNVYGIDMPTANELIAHGREVDEIRQIIGADGLIFQDLDDLIEAVRAENPDIQQFECSVF
NGIYVTKDVDQQYLDYLDSLRNDDVKAIQMQNDLESLEMHNEG

2. HisC

>Salmonella typhimurium
MSTENTLSVADLARENVRNLVPYQSARRLGGNGDVWLNANEFPTAVEFQLTQQTLNRYPECQPKAVIENYAQYAGVK
PEQVLVSRGADEGIELVIRAFCEPGKDAILYCPPTYGMYSVSAETIGVERRTVPALENWQLDLQGISDNLDGTKVVF
VCSPNNPTGQLINPQDLRTLLELTRGKAIVVADEAYIEFCPQATLTGWLVEYPHLVILRTLSKAFALAGLRCGFTLA
NEEVINLLLKVIAPYPLSTPVADIAAQALCPQGINAMRDRVAQTVQERQYLVNALQQTACVEHVFDSETNYILARFT
ASSSVFKSLWDQGIILRDQNKQPSLSGCLRITVGTRQENQRVIDALRAEPV

>Escherichia coli
MSTVTITDLARENVRNLTPYQSARRLGGNGDVWLNANEYPTAVEFQLTQQTLNRYPECQPKAVIENYAQYAGVKPEQ
VLVSRGADEGIELLIRAFCEPGKDAILYCPPTYGMYSVSAETIGVECRTVPTLDNWQLDLQGISDKLDGVKVVYVCS
PNNPTGQLINPQDFRTLLELTRGKAIVVADEAYIEFCPQASLAGWLAEYPHLAILRTLSKAFALAGLRCGFTLANEE
VINLLMKVIAPYPLSTPVADIAAQALSPQGIVAMRERVAQIIAEREYLIAALKEIPCVEQVFDSETNYILARFKASS
AVFKSLWDQGIILRDQNKQPSLSGCLRITVGTREESQRVIDALRAEQV

>Citrobacter rodentium
MSTEKTFSVSELARENVRNLTPYQSARRLGGNGDVWLNANEFPTAVEFQLTAQTLNRYPECQPKAVIENYAQYAGVR
PEQVLVSRGADEGIELLIRAFCEPGKDAILYCPPTYGMYSVSAETIGVECRTVPTLADWQLDMQGIADKLDGVKVVY
VCSPNNPTGQLINPQDLRTLLELTRGKAIVVADEAYIEFCPQATLAGWLADYPHLVVLRTLSKAFALAGLRCGFTLA
NEEVINLLLKVIAPYPLSTPVADIAAQALSPQGINAMRERVAQILAERQYLVNALRDIPCVEQVFDSETNYILARFT
ASSSVFKSLWDQGIILRDQNKQPSLSGCLRITVGTREESQRVIDALRAEQV

>Klebsiella variicola
MSIEDLARANVRALTPYQSARRLGGKGDVWLNANEFPTAVAFQLTAQTMNRYPEPQPKAVIESYARYADVKPEQVLV
SRGADEGIELLIRAFCEPGKDALLYCPPTYGMYSVSAETIGVECRTVPTLADWQLDLPGIEAQLDGVKVVFVCSPNN
PTGQIIDPQSIRDLLEMTRDKAIVVADEAYIEFCPQATLAGWLSDYPHLVVVRTLSKAFALAGLRCGFTLANAEVIN
VLLKVIAPYPLSTPVADIAAQALSAEGIAAMRQRVAQILDERRYLVEQLRGIACVEQVFDSEANYVLARITASSAVF
KSLWDQGIILRDQNKQPSLSGCLRITIGTRAESQRVIDALTAENV

>Cronobacter turicensis
MSIEELARENVRNLTPYQSARRLGGNGDVWLNANEYPQAVEFQLTAQTLNRYPECQPKSVIERYAQYAGVKPEQVLV
SRGADEGIELLIRAFCEPGRDAVLYCPPTYGMYSVSAETIGVECRTVQATDGWQLDLPAIAGQLDGVKVIFVCSPNN
PTGQLINPQDLRTLLEMARGKAIVVADEAYIEFCPQATLAGWLGEYPHLVVLRTLSKAFALAGLRCGFTLANEEVIN
LLLKVIAPYPLSTPVADIAAQALSPEGIAAMRDRVAQVLANRDALIAGLRETPCVEAIFDSETNYVLARITASSAVF
KSLWDQGIILRDQNRQPTLSGCLRITVGTREECQRVIDALRDQPGLAATERV

>Enterobacter cloacae
MNIEELARENVRRLTPYQSARRLGGNGDVWLNANEFPTAVQFELSQQTLNRYPECQPKAVIENYAQYAGVKPEQVLV
SRGADEGIELLIRAFCEPGKDAVMYCQPTYGMYSVSAETFGVACRNVLSLENWQLDLQGIADNLDGVKVVFVCSPNN
PTGQIINPQDIRTLLEMTRGKALVVADEAYIEFCPQATLAGWLEEYPHLVVLRTLSKAFALAGLRCGFTLANKAVID
LLLKVIAPYPLSTPVADIAAQALTLQGINAMRERVAQILEERQYLVTALKAIPCVEQVFDSETNYLLVRFTASSAVF
KSLWDQGIILRDQNKQPSLSGCLRITVGTRSESQRLIDALKAEKV

>Phytobacter diazotrophicus

MSIEELARANVRALTPYQSARRLGGKGDVWLNANEFPTAVEFNFTQQTLNRYPECQPKAVIEGYAQYAGLKPEQVLV
SRGADEGIELLIRAFCEPGKDAILFCPPTYGMYSVSAETFGVEYRTALTLDGWQLDLQNIADQLENVKLVYVCSPNN
PTGQIINPQDIRAVLEMTRGKALVVADEAYIEFCPQATLAGWLEEYPHLVILRTLSKAFALAGLRCGFTLANEEVIN
LLMKVIAPYPLSTPVADIAAQALSPEGIVAMRERVAQIVAEREYLSTSLRDIPCVETVFDSETNYILVRFKASSAVF
KSLWDQGIILRDQNKQPTLSGCLRITIGTREESQRVIDALRAESV

>Kosakonia sacchari
MSIEELARKNVRELTPYQSARRLGGKGDVWLNANEFPTAVPFELTQQTLNRYPECQPKAVIESYAQYAGVKPEQVLV
SRGADEGIELLIRAFCEPGKDAVLFCPPTYGMYSVSAETLGVEYRTVPALSDWQLDLQGIADKLDGVKVIYVCSPNN
PTGQIINPQDFRVLLEMTRGKAIVVADEAYIEFCPQATLAGWISEYPNLVVLRTLSKAFALAGLRCGFTLANEDVIA
LLLKVIAPYPLSTPVADIAAQALSTQGIVAMRERVAQILVERQYLVTALRDIPCVEQVFDSETNYILVRLTASSAVF
KSLWDQGIILRDQNKQPSLSGCLRITIGTRAESQRVIDALRAEPV

>Huaxiibacter chinensis
MSIEELARENVRRLTPYQSARRLGGNGDVWLNANEFPTAVSFALTQQTLNRYPECQPKAVIENYARYAGVKPEQVLV
SRGADEGIELLIRAFCEPGKDAVMYCQPTYGMYSVSAETLGVECRNVLSLEDWQLDLPTITENLDGVKVVFVCSPNN
PTGQIINPQDIRTLLELTRGKALVVADEAYIEFCPQATLAGWLEEYPHLVVLRTLSKAFALAGLRCGFTLANQEVIN
LLMKVIAPYPLSTPVADIAAQALTPQGINAMRERVAQILLERQYLSNALKEIPCVEQVFESETNYILVRFTASSAVF
KSLWDQGIILRDQNKQPSLSGCLRITVGTREESQRVIDALKAEKV

3. Icd(A)

>Salmonella typhimurium
MESKVVVPVEGKKITLQNGKLNVPENPIIPFIEGDGIGVDVTPAMLKVVDAAVEKAYKGERKISWMEIYTGEKSTQV
YGQDVWLPAETLDLIRDYRVAIKGPLTTPVGGGIRSLNVALRQELDLYVCLRPVRYYQGTPSPVKHPELTDMVIFRE
NSEDIYAGIEWKADSADAEKVIKFLREEMGVKKIRFPEHCGIGIKPCSEEGTKRLVRAAIEYAITNDRDSVTLVHKG
NIMKFTEGAFKDWGYQLARDEFGGELIDGGPWLKIKNPNTGKEIVVKDVIADAFLQQILLRPAEYDVIACMNLNGDY
ISDALAAQVGGIGIAPGANIGDECALFEATHGTAPKYAGQDKVNPGSIILSAEMMLRHMQWFEAADLIVKGMEGAIA
AKTVTYDFERLMEGAKLLKCSEFGDAIIANM

>Escherichia coli
MESKVVVPAQGKKITLQNGKLNVPENPIIPYIEGDGIGVDVTPAMLKVVDAAVEKAYKGERKISWMEIYTGEKSTQV
YGQDVWLPAETLDLIREYRVAIKGPLTTPVGGGIRSLNVALRQELDLYICLRPVRYYQGTPSPVKHPELTDMVIFRE
NSEDIYAGIEWKADSADAEKVIKFLREEMGVKKIRFPEHCGIGIKPCSEEGTKRLVRAAIEYAIANDRDSVTLVHKG
NIMKFTEGAFKDWGYQLAREEFGGELIDGGPWLKVKNPNTGKEIVIKDVIADAFLQQILLRPAEYDVIACMNLNGDY
ISDALAAQVGGIGIAPGANIGDECALFEATHGTAPKYAGQDKVNPGSIILSAEMMLRHMGWTEAADLIVKGMEGAIN
AKTVTYDFERLMDGAKLLKCSEFGDAIIENM

>Citrobacter rodentium
MESKVVVPAEGKKITLQNGKLNVPENPIIPFIEGDGIGVDVTPAMLKVVDAAVEKAYKGERKISWMEIYTGEKSTQV
YGQDVWLPPETLDLIREYRVAIKGPLTTPVGGGIRSLNVALRQELDLYVCLRPVRYYQGTPSPVKHPELTDMVIFRE
NSEDIYAGIEWKADSADAEKVIKFLREEMGVKKIRFPEHCGIGIKPCSEEGTKRLVRAAIEYAITNDRDSVTLVHKG
NIMKFTEGAFKDWGYQLARDEFGGELIDGGPWVKINNPNTGKEIVIKDVIADAFLQQILLRPAEYDVIACMNLNGDY
ISDALAAQVGGIGIAPGANIGDECALFEATHGTAPKYAGQDKVNPGSIILSAEMMLRHMQWFEAADLIVKGMEGAIA
AKTVTYDFERLMEGAKLLKCSEFGDAIIANM

>Klebsiella variicola
MESKVVVPAEGQKITLQNGKLNVPHNPIIPFIEGDGIGVDVTPAMLKVVDAAVEKAYKGERKISWMEVYTGEKSTQV
YGQDVWLPAETLDLIRDYRVAIKGPLTTPVGGGIRSLNVALRQELDLYVCLRPVRYYQGTPSPVKHPELTDMVIFRE
NSEDIYAGIEWKADSAEADKVIKFLRDEMGVKKIRFPEHCGIGIKPCSEEGTKRLVRVAIEYAITNDRDSLTLVHKG
NIMKFTEGAFKDWGYQLAREEFGGELIDGGPWVKIKNPNTGKEIVVKDVIADAFLQQILLRPAEYDVIACMNLNGDY
ISDALAAQVGGIGIAPGANIGDECALFEATHGTAPKYAGQDKVNPGSIILSAEMMLRHMQWFEAADLIVKGMEGAIA
AKTVTYDFERLMEGAKLLKCSEFGDAIIANM

>Cronobacter turicensis
MESKVVVPAEGEKITAQNGKLNVPHNPIIPFIEGDGIGVDVTPAMLKVVDAAVEKAYKGERKISWMEIYTGEKSTQL
YGQDVWLPQETLDLIREYRVAIKGPLTTPVGGGIRSLNVALRQELDLYVCLRPVRYYQGTPSPVKHPELTDMVIFRE

NSEDIYAGIEWKADSADAEKVIKFLREEMGVKKIRFPEHCGIGIKPCSEEGTKRLVRAAIEYAITNDRESVTLVHKG
NIMKFTEGAFKDWGYQLAKEEFGGELIDGGPWMKIKNPNTGKEIIVKDVIADAFLQQILLRPAEYDVIACMNLNGDY
ISDALAAQVGGIGIAPGANIGDECALFEATHGTAPKYAGQDKVNPGSIILSAEMMLRHMEWFEAADLIVKGMEGAIN
NKTVTYDFERLMEGAKLLKCSEFGDAIIANM

>Enterobacter cloacae
MESKVVVPAEGKKITLQNGKINVPHNPIIPFIEGDGIGVDVTPAMLKVVDAAVEKAYKGERKISWMEIYTGEKSTQV
YGQDVWLPAETLDLIRDYRVAIKGPLTTPVGGGIRSLNVALRQELDLYVCLRPVRYYQGTPSPVKHPELTDMVIFRE
NSEDIYAGIEWKADSADAEKVIKFLREEMGVKKIRFPEHCGIGIKPCSEEGTKRLVRAAIEYAITNDRDSVTLVHKG
NIMKFTEGAFKDWGYQLAQEEFGGELIDGGPWQKIKNPNTGKEIIIKDVIADAFLQQILLRPAEYDVIACMNLNGDY
ISDALAAQVGGIGIAPGANIGDECALFEATHGTAPKYAGQDKVNPGSIILSAEMMLRHMEWFEAADLIVKGMEGAIN
AKTVTYDFERLMEGAKLLKCSEFGDAIIANM

>Phytobacter diazotrophicus
MESKVVVPAEGKKITLQNGKLNVPNNPIIPFIEGDGIGVDVTPAMLKVVDAAVEKAYKGERKISWMEIYTGEKSTQI
YGQDVWLPAETLDLIREYRVAIKGPLTTPVGGGIRSLNVALRQELDLYVCLRPVRYYQGTPSPVKHPELTDMVIFRE
NSEDIYAGIEWKADSADAEKVIKFLREEMGVKKIRFPEHCGIGIKPCSEEGTKRLVRAAIEYAITNDRDSLTLVHKG
NIMKFTEGAFKDWGYQLIREEFGGELIDGGPWQKIKNPNTGKEIIIKDVIADAFLQQILLRPAEYDVIACMNLNGDY
ISDALAAQVGGIGIAPGANIGDECALFEATHGTAPKYAGQDKVNPGSVILSAEMMLRHMQWFEAADLIVKGMEGAIA
AKTVTYDFERLMEGAKLLKCSEFGDAIIANM

>Kosakonia sacchari
MESKVVVPAEGKKITLQNGKLNVPNNPIIPFIEGDGIGVDVTPAMLKVVDAAVEKAYKGERKISWMEIYTGEKSTHV
YGQDVWLPPETLDLIRDYRVAIKGPLTTPVGGGIRSLNVALRQELDLYICLRPVRYYEGTPSPVKHPELTDMVIFRE
NSEDIYAGIEWKADSADAEKVIKFLREEMGVKKIRFPEHCGIGIKPCSEEGTKRLMRAAIEYAITNDRDSVTLVHKG
NIMKFTEGAFKDWGYQLAREEFGGELIDGGPWQKIKNPKTGKEIIIKDVIADAFLQQILLRPAEYDVIACMNLNGDY
ISDALAAQVGGIGIAPGANIGDECALFEATHGTAPKYAGQDKVNPGSVILSAEMMLRHMGWVEAADLIVKGMSGAIN
AKTVTYDFERLMEGAKLLKCSEFGDAIIANM

>Huaxiibacter chinensis
MESKVVVPAEGKKITLQNGKLNVPHNPVIPFIEGDGIGVDVTPAMLKVVDAAVEKAYKGERKISWMEIYTGEKSTQV
YGQDVWLPAETLDLIRDYRVAIKGPLTTPVGGGIRSLNVALRQELDLYVCLRPVRYYQGTPSPVKHPELTDMVIFRE
NSEDIYAGIEWKADSADAEKVIKFLREEMGVKKIRFPEHCGIGIKPCSEEGTKRLVRAAIEYAITNDRDSVTLVHKG
NIMKFTEGAFKDWGYQLATEEFGGELIDGGPWQKIKNPNTGKEIIIKDVIADAFLQQILLRPAEYDVIACMNLNGDY
ISDALAAQVGGIGIAPGANIGDECALFEATHGTAPKYAGQDKVNPGSIILSAEMMLRHMEWFEAADLIVKGMEGAIN
AKTVTYDFERLMDGAKLLKCSEFGDAIIANM

4. BioB

>Salmonella typhimurium
MARHPRWTLSQVTELFEKPLLELLFEAQQIHRQHFDPQQVQVSTLLSIKTGACPEDCKYCPQSSRYKTGLEAERLME
VEQVLDSARKAKNAGSTRFCMGAAWRNPHERDMPYLEKIVQGVKAMGLETCMTLGMLNESQAQRLANAGLDYYNHNL
DTSPEFYGNIITTRTYQERLDTLEKVREAGIKVCSGGIVGLGETVTDRAGLLLQLANLPTPPESVPINMLVKVKGTP
LADNDDVDAFDFIRTIAVARIMMPTSYVRLSAGREQMNEQTQAMCFMAGANSIFYGCKLLTTPNPAEDKDLQLFRKL
GLNPQQTRVLAGDNEQQQRLEQTLMTPDTDDYYNAAAL

>Escherichia coli
MAHRPRWTLSQVTELFEKPLLDLLFEAQQVHRQHFDPRQVQVSTLLSIKTGACPEDCKYCPQSSRYKTGLEAERLME
VEQVLESARKAKAAGSTRFCMGAAWKNPHERDMPYLEQMVQGVKAMGLEACMTLGTLSESQAQRLANAGLDYYNHNL
DTSPEFYGNIITTRTYQERLDTLEKVRDAGIKVCSGGIVGLGETVKDRAGLLLQLANLPTPPESVPINMLVKVKGTP
LADNDDVDAFDFIRTIAVARIMMPTSYVRLSAGREQMNEQTQAMCFMAGANSIFYGCKLLTTPNPEEDKDLQLFRKL
GLNPQQTAVLAGDNEQQQRLEQALMTPDTDEYYNAAAL

>Citrobacter rodentium
MTNHSRWTLSQVTELFEKPLLELLFEAQQIHRQHFDPRQVQVSTLLSIKTGACPEDCKYCPQSARYKTGLEAERLME
VEQVLDSARKAKNAGSTRFCMGAAWKNPHERDMPFLEQMVQGVKAMGLEACMTLGALSESQAQRLAAAGLDYYNHNL
DTSPEFYGNIITTRTYQERLDTLDKVRDAGIKVCSGGIVGLGETVKDRAGLLLQLANLPTPPESVPINMLVKVKGTP

LADNDDVDAFDFIRTIAVARIMMPASYVRLSAGREQMNEQTQAMCFMAGANSIFYGCKLLTTPNPEEDNDLRLFRKL
GLNPQQTAVLAGDNEQQQRLEQALRTPDTDAYYNAAAV

>Klebsiella variicola
MAHRARWTMSQVTELFNKPLIDLLFEAQQIHRQHFDPRQVQVSTLLSIKTGACPEDCKYCPQSARYKTGLETERLME
VEQVLESARQAKNAGSTRFCMGAAWKNPNDRDMPYLEQMVKGVKALGLESCMTLGTLTDSQAQRLAGAGLDYYNHNL
DTSPEFYGNIITTRTYQERLDTLDKVRDAGIKVCSGGIVGLGETVKDRAGLLLQLANLPTPPESVPINMLVKVKGTP
LADNDDVDAFDFIRTIAIARIMMPTSYVRLSAGREQMNEQTQAMCFMAGANSIFYGCKLLTTPNPEEDKDLQLFRKL
GINPQQTAVLEGDNEQQQRLEQALLTPDTEEYYNAAAL

>Cronobacter turicensis
MAHLSRWTLSQVTELFEKPLLDLLFEAQQAHRQHFDPRQVQVSTLLSIKTGACPEDCKYCPQSARYKTGLEAERLME
VDQVLDSARKAKAAGSTRFCMGAAWKNPNDRDMPYLEQMVQGVKALGLETCMTLGTLSDDQAQRLGEAGLDYYNHNL
DTSPEFYGNIITTRTYQERLDTLEKVREAGIKVCSGGIVGLGETVTDRAGLLLQLANLPTPPESVPINMLVKVKGTP
LADNDDVDAFDFIRTIAVARIMMPTSYVRLSAGREQMNEQTQAMCFMAGANSIFYGCKLLTTPNPEEDKDLQLFRKL
GINPQQTAVLAGDNEQQARLEHALRDADNPQYYNAAAV

>Enterobacter cloacae
MAHHARWTMSQVTELFNKPFLELMFEAQQVHRQHFDPRHVQVSTLLSIKTGACPEDCKYCPQSARYKTGLESERLME
VEQVLESARKAKNAGSTRFCMGAAWKNPHDRDMPYLEQMVKGVKEMGLEACMTLGTLNEEQAQRLSAAGLDYYNHNL
DTSPEFYGNIITTRTYQERLDTLDKVREAGIKVCSGGIVGLGETVKDRAGLLLQLANLPTPPESVPINMLVKVKGTP
LADNEDVDAFDFIRTIAVARIMMPTSYVRLSAGREQMNEQTQAMCFMAGANSIFYGCKLLTTPNPEEDKDVQLFRKL
GLNPHQTEVLAGDNEQQQQLEQQIFNADTEQFYNAASI

>Phytobacter diazotrophicus
MAHQTRWTLSQVTSLFEKPLLELLFEAQQVHRQHFDPRQVQVSTLLSIKTGACPEDCKYCPQSARYKTGLDAERLME
VEQVLDSARKAKNAGSTRFCMGAAWKNPNDRDMPYLEQMVEGVKAMGLEACMTLGTLTDSQAQRLAHAGLDYYNHNL
DTSPEFYGNIITTRTYQERLDTLDKVRDAGIKVCSGGIVGLGETVTDRAGLLLQLANLPTPPESVPINMLVKVKGTP
LAENEDVDAFDFIRTIAVARIMMPTSYVRLSAGREQMNEQTQAMCFMAGANSIFYGCKLLTTPNPEEDKDLQLFRKL
GLNPQQTGVLNGDRAQQEQLEQQLVHADTDQYYNAAAL

>Kosakonia sacchari
MAHHARWTMSQVTALFEKPLLELLFEAQQIHRQHFDPRQVQVSTLLSIKTGACPEDCKYCPQSARYKTGLESERLME
VEQVLESARQAKRAGSTRFCMGAAWKNPHDRDMPYLEKMVEGVKAMGLEACMTLGTLNENQAQRLASAGLDYYNHNL
DTSPEFYGNIITTRTYQERLDTLDKVRDAGIKVCSGGIVGLGETVNDRAGLLLQLANLPTPPESVPINMLVKVKGTP
LADNEDVDAFDFIRTIAVARIMMPTSHVRLSAGREQMNEQTQAMCFMAGANSIFYGCKLLTTPNPEEDKDLQLFRKL
GLNPQQTAVLNGDNEQQQQLEQQLLHADTDQYYNAAAV

>Huaxiibacter chinensis
MAHHARWTMSQVTELFEKPFLELMFEAQQIHRQHFDPRHVQVSTLLSIKTGACPEDCKYCPQSARYKTGLEAERLME
VEQVLDSARKAKNAGSTRFCMGAAWKNPHDRDMPYLEQMVQGVKAMGLEACMTLGTLDESQAQRLSAAGLDYYNHNL
DTSPEFYGNIITTRTYQERLDTLDKVREAGIKVCSGGIVGLGETVKDRAGLLLQLANLPTPPESVPINMLVKVKGTP
MADNEDVDAFDFIRTIAVARIMMPTSFVRLSAGREQMSEQTQAMCFMAGANSIFYGCKLLTTPNPEEDKDVQLFRKL
GLNPHQTEVLTGDNEQQQQLEQQLFNADTDQYYNAATV

5. NuoC

>Salmonella typhimurium
MVNNMTDLTAQDAAWSTRDHLDDPVIGELRNRFGPDAFTVQATRTGIPVVWVKREQLLEVGDFLKKLPKPYVMLFDL
HGMDERLRTHRDGLPAADFSVFYHLISIERNRDIMLKVALSENDLRVPTFTKLFPNANWYERETWEMFGIDIEGHPH
LTRIMMPQTWEGHPLRKDYPARATEFDPFELTKAKQDLEMEALTFKPEDWGMKRGTDNEDFMFLNLGPNHPSAHGAF
RIILQLDGEEIVDCVPDIGYHHRGAEKMGERQSWHSYIPYTDRIEYLGGCVNEMPYVLAVEKLAGITVPDRVNVIRV
MLSELFRINSHLLYISTFIQDVGAMTPVFFAFTDRQKIYDLVEAITGFRMHPAWFRIGGVAHDLPRGWDRLLREFLE
WMPKRLDSYEKAALRNTILKGRSQGVAAYGAKEALEWGTTGAGLRATGIDFDVRKWRPYSGYENFDFEVPVGGGVSD
CYTRVMLKVEELRQSLRILQQCLDNMPEGPFKADHPLTTPPPKERTLQHIETLITHFLQVSWGPVMPAQESFQMVEA
TKGINSYYLTSDGSTMSYRTRVRTPSFAHLQQIPSAIRGSLVSDLIVYLGSIDFVMSDVDR

```
>Escherichia coli
MVNNMTDLTAQEPAWQTRDHLDDPVIGELRNRFGPDAFTVQATRTGVPVVWIKREQLLEVGDFLKKLPKPYVMLFDL
HGMDERLRTHREGLPAADFSVFYHLISIDRNRDIMLKVALAENDLHVPTFTKLFPNANWYERETWDLFGITFDGHPN
LRRIMMPQTWKGHPLRKDYPARATEFSPFELTKAKQDLEMEALTFKPEEWGMKRGTENEDFMFLNLGPNHPSAHGAF
RIVLQLDGEEIVDCVPDIGYHHRGAEKMGERQSWHSYIPYTDRIEYLGGCVNEMPYVLAVEKLAGITVPDRVNVIRV
MLSELFRINSHLLYISTFIQDVGAMTPVFFAFTDRQKIYDLVEAITGFRMHPAWFRIGGVAHDLPRGWDRLLREFLD
WMPKRLASYEKAALQNTILKGRSQGVAAYGAKEALEWGTTGAGLRATGIDFDVRKARPYSGYENFDFEIPVGGGVSD
CYTRVMLKVEELRQSLRILEQCLNNMPEGPFKADHPLTTPPPKERTLQHIETLITHFLQVSWGPVMPANESFQMIEA
TKGINSYYLTSDGSTMSYRTRVRTPSFAHLQQIPAAIRGSLVSDLIVYLGSIDFVMSDVDR

>Citrobacter rodentium
MVNTMTDLTAQDAAWQTRDHLDDPVIGELRNRFGPDAFTVQATRTGVPVVWVKREQLLEVGDFLKKLPKPYVMLYDL
HGMDERLRTHREGLPAADFSVFYHLISIERNRDIMLKVALAENDLRVPTFTRLFPNANWYERETWEMFGIDIEGHPH
LTRIMMPNTWTGHPLRKDYPARATEFDPFELTKAKQDLEMEALTFKPEDWGMKRGTENEDFMFLNLGPNHPSAHGAF
RIILQLDGEEIVDCVPDIGYHHRGAEKMGERQSWHSYIPYTDRIEYLGGCVNEMPYVLAVEKLAGITVPDRVNVIRV
MLSELFRINSHLLYISTFIQDVGAMTPVFFAFTDRQKIYDLVEAITGYRMHPAWFRIGGVAHDLPRGWDRLLREFLD
WMPKRLASYEKAALRNTILKGRSQGVAAYGGKEALEWGTTGAGLRAAGIDFDVRKARPYSGYENFDFEVPVGGGVSD
CYTRVMLKVEELRQSLRILEQCLNNMPEGPFKADHPLTTPPPKERTLQHIETLITHFLQVSWGPVMPANESFQMVEA
TKGINSYYLTSDGSTMSYRTRVRTPSFAHLQQIPAAIRGSLVSDLIVYLGSIDFVMSDVDR

>Klebsiella variicola
MVNNMTDLTAHDAAPAWQTRDHLDDPVIGELRNRFGPDAFTVQPTRTGVPVVWVKREQLLEVGDFLKKLPKPYVMLF
DLHGMDERLRTHRDGLPAADFSVFYHLISIDRNRDIMLKVALSENDLHLPTFTKLFPNANWYERETWEMFGMTFDGH
PNLRRIMMPPTWEGHPLRKDYPARATEFDPFELTKAKQDLEMEALTFKPEEWGMKRSTDNEDFMFLNLGPNHPSAHG
AFRIILQLDGEEIVDCVPDIGYHHRGAEKMGERQSWHSYIPYTDRIEYLGGCVNEMPYVLAVEKLAGITVPDRVNVI
RVMLSELFRINSHLLYISTFIQDVGAMTPVFFAFTDRQKIYDLVEAITGFRMHPAWFRIGGVAHDLPRGWDRLLREF
LEWMPKRLDSYEKAALRNTILKGRSVGVAAYTAKEALEWGTTGAGLRATGIGFDVRKWRPYSGYENFDFEVPTGGGV
SDCYTRVMLKVEELRQSLRILQQCLDNMPEGPFKADHPLTTPPPKERTLQHIETLITHFLQVSWGPVMPANESFQMI
EATKGINSYYLTSDGSTMSYRTRVRTPSFAHLQQIPSAIRGSLVSDLIVYLGSIDFVMSDVDR

>Cronobacter turicensis
MVNNMTDLTAQDAVLPVWQTRDHLDDPVIGELRNRFGPDAFTVQATRTGVPVVWVKREQLLEVGEFLRKLPKPYVML
YDLHGMDERLRTHRNGLPAADFSVFYHLLSIDRNRDIMLKVALSENDLNVPTFTRLFPNANWYERETWEMFGITFTG
HPNLRRIMMPPTWEGHPLRKDYPARATEFDPFTLTKQKEELEMEALTFKPEEWGMKRGNDTEDFMFLNLGPNHPSAH
GAFRIILQLDGEEIVDCVPDIGYHHRGAEKMGERQSWHSYIPYTDRIEYLGGCVNEMPYVLAVEKLAGIQVPERVEV
IRVMLSELFRINSHLLYISTFIQDVGAMTPVFFAFTDRQKIYDLVEAITGFRMHPAWFRIGGVAHDLPRGWDRLLKE
FLEWMPKRLDSYVKAALKNTILKGRSQGVAAYDAKEALAWGTTGAGLRATGIDFDVRKARPYSGYQNFEFEVPVGGG
ISDCYTRVMLKVEEVRQSLRILEQCLKNMPEGPFKADHPLTTPPPKERTLQHIETLITHFLQVSWGPVMPANESFQM
IEATKGINSYYLTSDGSTMSYRTRVRTPSFPHLQQIPSVIRGSLVSDLIVYLGSIDFVMSDVDR

>Enterobacter cloacae
MVNNMTDLTAQEAAWQTRDHLDDPVIGELRNRFGPDAFTVQATRTGVPVVWVKREQLLEVVDFLKKLPKPYVMLFDL
HGMDERLRTHRQGLPAADFSVFYHLISIDRNTDIMLKVALSENDMHLPTLTKLFPNANWYERETWEMFGMTFDGHPH
LTRIMMPQTWTGHPLRKDYPARATEFDPFELTKAKQDLEMEALTFKPEDWGMKRGTENEDFMFLNLGPNHPSAHGAF
RIILQLDGEEIVDCVPDIGYHHRGAEKMGERQSWHSYIPYTDRIEYLGGCVNEMPYVLAVEKLAGITVPDRVNVIRV
MLSELFRINSHLLYISTFIQDVGAMTPVFFAFTDRQKIYDLVEAITGFRMHPAWFRIGGVAHDLPRGWDRLLREFLD
WMPKRLASYEKAALRNSILKGRSQGVAAYGAKEALEWGTTGAGLRATGIDFDVRKARPYSGYENFDFEVPVGGGVSD
CYTRVMLKVEELRQSLRILEQCLNNMPEGPFKADHPLTTPPPKERTLQHIETLITHFLQVSWGPVMPAQESFQMIEA
TKGINSYYLTSDGSTMSYRTRVRTPSFAHLQQIPSAIRGSLVSDLIVYLGSIDFVMSDVDR

>Phytobacter diazotrophicus
MVNNMTDLTAQEATWQTRDHLDDPVIGELRNRFGPDAFTVQATRTGVPVVWVKREQLLEVGDFLKKLPKPYVMLFDL
HGMDERLRTHRAGLPAADFSVFYHLISIERNRDIMLKVALSENDLRVPTFTKLFPNANWYERETWEMFGIDIEGHPH
LTRIMMPQTWTGHPLRKDYPARATEFDPFELTKAKQDLEMEALKFKPEDWGMKRGTDNEDFMFLNLGPNHPSAHGAF
RIILQLDGEEIVDCVPDIGYHHRGAEKMGERQSWHSYIPYTDRIEYLGGCVNEMPYVLAVEKLAGITVPDRVNVIRV
MLSELFRINSHLLYISTFIQDVGAMTPVFFAFTDRQKIYDLVEAITGFRMHPAWFRIGGVAHDLPRGWDRLLREFLD
WMPKRLASYEKAALRNTILKGRSQGVAAYGAKEALEWGTTGAGLRATGIDFDVRKARPYSGYENFDFEVPVGGGVSD
```

CYTRVMLKVEELRQSLRILEQCLNNMPEGPFKADHPLTTPPPKERTLQHIETLITHFLQVSWGPVMPAQESFQMIEA
TKGINSYYLTSDGSTMSYRTRVRTPSFAHLQQIPSAIRGSLVSDLIVYLGSIDFVMSDVDR

>Kosakonia sacchari
MVNNMTDLTAQEAAWQTRDHLDDPVIGELRNRFGPDAFTVQATRTGVPVVWVKREQLLEVVDFLKKLPKPYVMLFDL
HGMDERLRTHRAGLPAADFSVFYHFISIDRNRDIMLKVALAENDLNVPTITKLFPNANWYERETWEMFGVTFNGHPH
LTRIMMPQTWTGHPLRKDYPARATEFDPFELTKAKQDLEMEALTFKPEDWGMKRGTENEDFMFLNLGPNHPSAHGAF
RIILQLDGEEIVDCVPDIGYHHRGAEKMGERQSWHSYIPYTDRIEYLGGCVNEMPYVLAVEKLAGITVPDRVNVIRV
MLSELFRINSHLLYISTFIQDVGAMTPVFFAFTDRQKIYDLVEAITGFRMHPAWFRIGGVAHDLPRGWDRLLREFLD
WMPKRLASYEKAALRNTILKGRSQGVAAYNAKEALEWGTTGAGLRATGINFDVRKARPYSGYENFDFEIPVGGGVSD
CYTRVMLKVEELRQSLRILEQCLNNMPEGPFKADHPLTTPPPKERTLQHIETLITHFLQVSWGPVMPANESFQMIEA
TKGINSYYLTSDGSTMSYRTRVRTPSFAHLQQIPSAIRGSLVSDLIVYLGSIDFVMSDVDR

>Huaxiibacter chinensis
MVNNMTDLTAQAASLTRDHLDDPVIGELRNRFGPDAFTVQATRTGVPVVWVKREQLLEVVDFLKKLPKPYVMLFDLH
GMDERLRTHRQGLPAADFSVFYHLISIDRNTDIMLKVALSENDMHLPTITKLFPNANWYERETWEMFGMTFDGHPHL
TRIMMPQTWTGHPLRKDYPARATEFDPFELTKAKQDLEMEALTFKPEDWGMKRGTENEDFMFLNLGPNHPSAHGAFR
IILQLDGEEIVDCVPDIGYHHRGAEKMGERQSWHSYIPYTDRIEYLGGCVNEMPYVLAVEKLAGITTPDRVNVIRVM
LSELFRINSHLLYISTFIQDVGAMTPVFFAFTDRQKIYDLVEAITGFRMHPAWFRIGGVAHDLPRGWDRLLREFLDW
MPKRLASYEKAALRNTILIGRSKGVAAYGAKEALEWGTTGAGLRATGIDFDVRKARPYSGYENFDFEVPVGGGVSDC
YTRVMLKVEELRQSLRILEQCLNNMPEGPFKADHPLTTPPPKERTLQHIETLITHFLQVSWGPVMPAQESFQMIEAT
KGINSYYLTSDGSTMSYRTRVRTPSFAHLQQIPAAIRGSLVSDLIVYLGSIDFVMSDVDR

6. ThiL

>Salmonella typhimurium
MACGEFSLIARYFDRVRSSRLDVETGIGDDCALLNIPEKQTLAISTDTLVAGNHFLPDIDPADLAYKALAVNLSDLA
AMGADPAWLTLALTLPEVDEPWLEAFSDSLFALLNYYDMQLIGGDTTRGPLSMTLGIHGYIPAGRALKRSGAKPGDW
IYVTGTPGDSAAGLAVLQNRLQVSEETDAHYLIQRHLRPTPRILHGQALRDIASAAIDLSDGLISDLGHIVKASGCG
ARVDVDALPKSDAMMRHVDDGQALRWALSGGEDYELCFTVPELNRGALDVAIGQLGVPFTCIGQMSADIEGLNFVRD
GMPVTFDWKGYDHFATP

>Escherichia coli
MACGEFSLIARYFDRVRSSRLDVELGIGDDCALLNIPEKQTLAISTDTLVAGNHFLPDIDPADLAYKALAVNLSDLA
AMGADPAWLTLALTLPDVDEAWLESFSDSLFDLLNYYDMQLIGGDTTRGPLSMTLGIHGFVPMGRALTRSGAKPGDW
IYVTGTPGDSAAGLAILQNRLQVADAKDADYLIKRHLRPSPRILQGQALRDLANSAIDLSDGLISDLGHIVKASDCG
ARIDLALLPFSDALSRHVEPEQALRWALSGGEDYELCFTVPELNRGALDVALGHLGVPFTCIGQMTADIEGLCFIRD
GEPVTLDWKGYDHFATP

>Citrobacter rodentium
MACGEFSLIARYFDRVRSSRRDVETGIGDDCALLNIPEKQTLAISTDTLVAGNHFMPDIDPADLAYKALAVNLSDLA
AMGADPAWLTLALTLPEVDEAWLETFSDSLFDLLNYYDMQLIGGDTTRGPLSMTLGIHGYVPVGRALKRAGAKPGDW
IYVTGTPGDSAAGLAILQNRLQVADDVDNAYLLQRHLRPTPRILQGQALRDLASAAIDLSDGLISDLGHIVKASACG
ARIDVDALPYSQAMLRHVGAEQALRWALSGGEDYELCFTVPELNRGALDVALAHLGVPFTCIGQMSADVEGICFIRD
GEPVTFDWKGYDHFAAL

>Klebsiella variicola
MACGEFSLIARYFDRVKSARLDVETGIGDDCALLNIPEKKTLAISTDTLVAGNHFLPDIDPADLAYKALAVNLSDLA
AMGAEPAWLTLALTLPEVDETWLEAFSDSLFVQLDYYDMQLIGGDTTRGPLAMTLGIHGFVPPGRAMKRAGAKPGDW
IYVTGTPGDSAAGLAVLQNRLTVDEPSDADYLLARHLRPMPRVLQGQALRDLATSAIDLSDGLISDLGHILKASGCG
ARIDLDAMPYSDAILRQVEPEQALRWALAGGEDYELCFTVPELNRGALDVALGHLGARFTCIGQIAPESEGLQFIRD
GKPVALDLKGYDHFA

>Cronobacter turicensis
MSCGEFSLIARYFDRVRSSRLDVETGIGDDCALLTVPEKQTLAISTDTLVSGIHFLPDIDPRDLGYKALAVNVSDLA
AMGADPAWLTLALTLPEVNEPWLEAFSDSLFEQLNYYDMQLIGGDTTRGPLSMTLGIHGFVPAGRALKRSGARPGDW
VFITGTPGDSAAGLAILQKRLNVDNESDAGYLVNRHLRPTPRVLHGQALRGLASAAIDLSDGLISDLGHILKASDCG

ARIELNDLPYSEALSRHVEPEQALRWALSGGEDYELCFTVSEINRGALEVAVNHLGVPVTCIGQLTTASEGMVFLRD
GAPVTLDWKGYDHFETHV

>Enterobacter cloacae
MACGEFSLIARYFDRVRTSRLDVETGIGDDCALLNIPEKQTLAISTDTLVCGRHFLPDIDPADLAYKALAVNVSDLA
AMGADPAWLTLALTLPEVDEPWLEAFSDALFEQLNYYDMQLIGGDTTAGPLSMTLAIHGYVPAGRALKRSGAKPGDW
IYVTGTPGDSAAGLAILQDRLTVNDTDDAAYLVKRHLRPTPRILHGQALRERASSAIDLSDGLISDLGHILKASGVG
ARVDLDLFPLSEQLLRHVEPEQALRWALSGGEDYELCFTVPELNRGTLDVALAHLGAKFTCIGQIMPESEGLKFVKD
GAPVTLDWKGYDHFG

>Phytobacter diazotrophicus
MACGEFSLIARYFDRVTSSRRDVDTGIGDDCALLNVPEKQTLAISTDTLVSGIHFLPDIEPADLAYKALAVNISDLA
AMGADPAWLTLALTLPNVDEDWLKAFSDSLFEQLNYYDMQLIGGDTTRGPLSMTLGIHGYVPAGRALKRSGAKPGDW
IYVTGTPGDSAAGLAILQKQLQVADAQDAKYLLQRHLRPVPRVLHGQAMRNLASSAIDLSDGLISDLGHILKASGCG
ARVDLNALPYSQAMLRHVDAEQALRWALSGGEDYELCFTVPEINRGALEVAVGHLGVPVTCIGQMSADVEGLNFTRE
GKTVTLDWKGYDHFAAL

>Kosakonia sacchari
MACGEFSLIARYFDRVRNSRRDVETGIGDDCALLNVAEKQTLAISVDTLVAGNHFLPDIDPADLAYKAMASNLSDLA
AMGADPAWLTLALTLPEADEAWLQAFSDSLFEQLNYYDMQLIGGDTTRGPLSMTIGIHGYVPVGRAMKRSGAKPGDW
IYVTGTPGDSAAGLAILQQRLAVNDSADAQYLLKRHLRPTPRVLQGQALRNLASSAIDLSDGLISDLAHVLKASSCG
ARIDLDALPFSNAVRRHVMPDQALRWALSGGEDYELCFTVPELNRGALDVAIGQLGAPFTCIGQISADIEGLHFTRD
GKAVTLDWKGYDHFAAH

>Huaxiibacter chinensis
MACGEFSLIARYFDRVRSSRLDVETGIGDDCALLNIPEKQTLAISTDTLVCGRHFLPDIDPADLAYKALAVNVSDLA
AMGADPAWLTLALTLPSVDEAWLEAFSDALFEQLNYYDMQLIGGDTTAGPLSMTLAIHGYVPVGRALKRSGAKPGDW
IYVTGTPGDSAAGLAILQARLAVADAQDSAYLVKRHLRPTPRILQGQALRDRASSAIDLSDGLISDLGHILKASGVG
ARIDLDLFPLSEQILRHVEPEQALRWALSGGEDYELCFTVPELNRGTIDVALGHLGARFTCIGQIMPESEGLQFVKE
GAPVTLDWKGYDHFG

7. Cfa

>Salmonella typhimurium
MSSSCIEEVSVPDDNWYRIANELLSRADITINGSAPSDIRVKNPDFFKRVLQEGSLGLGESYMDGWWECERLDIFFS
KVLRAGLENQLPHHVKDTLRILGARLINLQSKKRAWIVGKEHYDLGNDLFSRMLDPYMQYSCAYWKDADTLEAAQQA
KLKLICEKLQLQPGMRVLDIGCGWGGLSQYMATHYGVSVVGVTISAEQQKMAQTRCEGLDVSILLEDYRDLNDQFDR
IVSVGMFEHVGPKNYNTYFEVVDRNLKPDGLFLLHTIGSKKTDHNVDPWINKYIFPNGCLPSVRQIAEASESHFVME
DWHNFGADYDTTLMAWHERFINAWPEIAGNYNERFKRMFSYYLNACAGAFRARDIQLWQVVFTRGVENGLRVPR

>Escherichia coli
MSSSCIEEVSVPDDNWYRIANELLSRAGIAINGSAPADIRVKNPDFFKRVLQEGSLGLGESYMDGWWECDRLDMFFS
KVLRAGLENQLPHHFKDTLRIAGARLFNLQSKKRAWIVGKEHYDLGNDLFSRMLDPFMQYSCAYWKDADNLESAQQA
KLKMICEKLQLKPGMRVLDIGCGWGGLAHYMASNYDVSVVGVTISAEQQKMAQERCEGLDVTILLQDYRDLNDQFDR
IVSVGMFEHVGPKNYDTYFAVVDRNLKPEGIFLLHTIGSKKTDLNVDPWINKYIFPNGCLPSVRQIAQSSEPHFVME
DWHNFGADYDTTLMAWYERFLAAWPEIADNYSERFKRMFTYYLNACAGAFRARDIQLWQVVFSRGVENGLRVAR

>Citrobacter rodentium
MSSSCIEEINVPDDDWYRIANELLSRAGIAINGSAPSDIHVNNPGFFKRVLQEGSLGLGESYMDGWWECDRLDMFFS
KVLRAGLENQLPHHFKDTLRIAGARLFNLQSKKRAWIVGKEHYDLGNDLFSRMLDPYMQYSCAYWKDADSLEAAQQA
KLQLICEKLQLRPGMRVLDIGCGWGGLAQYMATNFDVSVVGVTISAEQQKMAQARCAGLDVSILLEDYRDLRDQFDR
IVSVGMFEHVGPKNYNTYFEVVDRNLKADGIFLLHTIGSKKTDNNVDPWINKYIFPNGCLPSVRQIASASESHFVME
DWHNFGADYDTTLMAWYERFLAAWPQIADNYSERFKRMFTYYLNACAGAFRARDIQLWQVVFTRGVENGLRVAR

>Klebsiella variicola
MSSSCIEEVSVPNDDWYRIAAELLGRAGIEINGSAPSDLRIKNPLFFKRVLQEGSLGLGESYMDGWWDCERLDIFFH
KVLRAGLEKQLPHHFKDTLRIAGARLFNLQSKKRAWIVGKEHYDLGNDLFSRMLDPYMQYSCGYWKEAQTLEAAQQA

KLDLICRKLELEPGMRVLDIGCGWGGLAEYMARNYQVSVVGVTISAEQQKMAQARCADLDVEIRLQDYRDLHDSFDR
IVSVGMFEHVGPKNYATYFEVADRNLKPNGRFLLHTIGSKVTDHNVDPWIDKYIFPNGCLPSVRQIADASEKHFVME
DWHNFGADYDTTLMAWYERFLASWPEIADNYSERFKRMFSYYLNACAGAFRARDIQLWQVVFSRGIEHGLRIAR

>Cronobacter turicensis
MSSSCIEEVSIPDNHWYRIATELLSRAGIAVNGAAPSDIQVKNPDFFKRVLQEGSLGLGESYMDGWWECERLDLFFT
KVLRAGLEDQLPHHLKDTLRILSARLFNLQSKKRAWIVGKEHYDLGNDLFSRMLDPYMQYSCGYWKEADNLEDAQQA
KLKLICDKLALKPGMTLLDIGCGWGGLAAFAARHYGVSVTGVTISAEQQKMAQARCEGLDVTILLQDYRDLDSQFDR
IVSVGMFEHVGPKNYATYFDVADRNLKPDGLFLLHTIGSRKTDNNVDPWIDKYIFPNGCLPSARQIAAASEPHFVME
DWHNFGADYDKTLMAWYARFLDAWPEIADNYSERFKRMFTYYLNACAGAFRARDIQLWQVVFSRGVENGLRVAR

>Enterobacter cloacae
MSSSCIEEVSVPDDNWSRIVSELLGRAGITINGSSPSDPQVKHPDFFKRVLREGSLGLGESYMDGWWDCERLDIFFA
SVLRAGLENQLPRNIKDTLRVASARLFNLQSKKRAWIVGKEHYDLGNDLFSRMLDPFMQYSCAYWKKATTLEEAQQD
KLRLISEKLQLQPGMRVLDIGCGWGGLAYFMAKHYGVSVVGVTISAEQQKMARERCQGLDVDIRLQDYRDLNEQFDR
IVSVGMFEHVGPKNYDTYFEVVDRNLKPDGIFLLHTIGSKRTDNNVDPWINKYIFPNGCLPSVRQIANASESHFIME
DWHNFGADYDTTLMAWHERFQQAWPEIADNYSERFKRMFSYYLNACAGAFRARDIQLWQVVFSRGIEHGLRVPR

>Phytobacter diazotrophicus
MSSSCIEEVSVRDDNWFRIANELLSRAGILVNGPSASDIQVKNPDFFKRVLQEGSLGLGESYMDGWWECDRLDVFFT
KVLRAGLEKQLPRHLKDTLRIAGARLFNLQSKKRAWIVGKEHYDLGNDLFTRMLDPQMQYSCAYWKDAERLEDAQIA
KLRLICEKLQLKPGMRVLDIGCGWGGLAQFMAQNYAVSVVGVTISAEQQKHAQQRCLGLDVDIRLQDYRDLNDQFDR
IVSVGMFEHVGPKNYDTYFTVVDRNLKPDGLFLLHTIGSRRTDHNVDPWIDRYIFPNGCLPSVRQIANASESHFVME
DWHNFGADYDKTLMAWYERFLASWPEIADNYSERFKRMFSYYLNACAGAFRARDIQLWQVVFSRGIEHGLRVAR

>Kosakonia sacchari
MSSSCIEEVSVRDDNWFRIANELLGRAGIRVNGPAASDIQVKNPDFFKRVLQEGSLGLGESYMDGWWDCPRLDIFFT
KVLRAGLENQLPHHLKDTLRIAGARLFNLQSKKRAWIVGKEHYDLGNDLFTRMLDPQMQYSCAYWKDAETLDDAQRA
KLRLIGEKLKLKPGMRVLDIGCGWGGLAQFMAQNYGVSVVGVTISAEQQKLAQKRCEGLDVDIRLQDYRDLDEQFDR
IVSVGMFEHVGPKNYATYFRVVDHNLKPDGIFLLHTIGSKRTDHNVDPWINRYIFPNGCLPSVRQIADASESHFVME
DWHNFGADYDRTLMAWYERFLGAWPEIADNYSERFKRMFSYYLNACAGAFRARDIQLWQVVFSRGIEHGLRVAR

>Huaxiibacter chinensis
MSSSCIEEVSVRDDNWFRIANELLNRAGVAINGTAPSDIQVKHPDFFKRVLQEGSLGLGESYMDGWWDCERLDLFFT
QVLRAGLERQLPRHVKDTLRIAAARILNLQSKKRAWIVGKEHYDLGNDLFSRMLDPLMQYSCAYWKDADTLEEAQEA
KLRLICEKLQLAPGMRVLDIGCGWGGLAWFMAKHYGVSVVGVTISAEQQKMAQARCEGLDVTILLQDYRDLRDQFDR
IVSVGMFEHVGPKNYDTYFDVVDRNLKPEGLFLLHTIGSKITDNNVDPWINKYIFPNGCLPSVRQIANASEAHFVME
DWHNFGADYDTTLMAWYSRFLAAWPEIADNYSERFKRMFIYYLNACAGAFRARDIQLWQVVFSRGVENGLRVAR

8. PykF

>Salmonella typhimurium
MKKTKIVCTIGPKTESEEMLSKMLDAGMNVMRLNFSHGDYAEHGQRIQNLRNVMSKTGKKAAILLDTKGPEIRTIKL
EGGNDVSLKAGQTFTFTTDKSVVGNNEIVAVTYEGFTSDLSVGNTVLVDDGLIGMEVTAIEGNKVICKVLNNGDLGE
NKGVNLPGVSIALPALAEKDKQDLIFGCEQGVDFVAASFIRKRSDVVEIREHLKAHGGENIQIISKIENQEGLNNFD
EILEASDGIMVARGDLGVEIPVEEVIFAQKMMIEKCIRARKVVITATQMLDSMIKNPRPTRAEAGDVANAILDGTDA
VMLSGESAKGKYPLEAVSIMATICERTDRVMNSRLDYNNDSRKLRITEAVCRGAVETAEKLEAPLIVVATQGGKSAR
AVRKYFPDATILALTTNEVTARQLVLSKGVVSQLVKEINSTDDFYRLGKDVALQSGLAQKGDVVVMVSGALVPSGTT
NTASVHVL

>Escherichia coli
MKKTKIVCTIGPKTESEEMLAKMLDAGMNVMRLNFSHGDYAEHGQRIQNLRNVMSKTGKTAAILLDTKGPEIRTMKL
EGGNDVSLKAGQTFTFTTDKSVIGNSEMVAVTYEGFTTDLSVGNTVLVDDGLIGMEVTAIEGNKVICKVLNNGDLGE
NKGVNLPGVSIALPALAEKDKQDLIFGCEQGVDFVAASFIRKRSDVIEIREHLKAHGGENIHIISKIENQEGLNNFD
EILEASDGIMVARGDLGVEIPVEEVIFAQKMMIEKCIRARKVVITATQMLDSMIKNPRPTRAEAGDVANAILDGTDA
VMLSGESAKGKYPLEAVSIMATICERTDRVMNSRLEFNNDNRKLRITEAVCRGAVETAEKLDAPLIVVATQGGKSAR

AVRKYFPDATILALTTNEKTAHQLVLSKGVVPQLVKEITSTDDFYRLGKELALQSGLAHKGDVVVMVSGALVPSGTT
NTASVHVL

>Citrobacter rodentium
MKKTKIVCTIGPKTESEEMLTKMLDAGMNVMRLNFSHGDYAEHGQRIQNLRNVMSKTGKKAAILLDTKGPEIRTIKL
EGGNDVSLKAGQTFTFTTDKSVVGNNEIVAVTYEGFTSDLSVGNTVLVDDGLIGMEVTAIEGDKVICKVLNNGDLGE
NKGVNLPGVSIALPALAEKDKQDLIFGCEQGVDFVAASFIRKRSDVVEIREHLKAHGGENIQIISKIENQEGLNNFD
EILEASDGIMVARGDLGVEIPVEEVIFAQKMMIEKCIRARKVVITATQMLDSMIKNPRPTRAEAGDVANAILDGTDA
VMLSGESAKGKYPLEAVSIMATICERTDRVMSSRLDFNNDSRKLRITEAVCRGAVETAEKLEAPLIVVATQGGKSAR
AVRKYFPDATILALTTNEVTARQLVLSKGVVAQMVKEITSTDDFYRLGKEVALQSGLAQKGDVVVMVSGALVPSGTT
NTASVHVL

>Klebsiella variicola
MKKTKIVCTIGPKTESEEMLTKMLEAGMNVMRLNFSHGDYAEHGQRIQNLRNVMSKTGKKAAILLDTKGPEIRTIKL
EGGNDVSLKAGQTFTFTTDKSVVGNNEIVAVTYEGFTSDLTVGNTVLVDDGLIGMEVTAIEGNKVICKVLNNGDLGE
NKGVNLPGVSIALPALAEKDKQDLIFGCEQGVDFVAASFIRKRSDVVEIREHLKAHGGENIQIISKIENQEGLNNFD
EILEASDGIMVARGDMGVEIPVEEVIFAQKMIIEKCIRARKVVITATQMLDSMIKNPRPTRAEAGDVANAILDGTDA
VMLSGESAKGKYPLEAVTIMATICERTDRVMTSRLDFNNDNRKLRITEAVCRGAVETAEKLEAPLIVVATQGGKSAR
AVRKYFPDATILALTTNETTARQLVLSKGVVPQLVEEIASTDDFYHLGKDLALKSGLARKGDVVVMVSGALVPSGTT
NTASVHVL

>Cronobacter turicensis
MKKTKIVCTIGPKTESEEMLTKMLDAGMNVMRLNFSHGDYEEHGQRIKNLRNVLAKTGKQAAILLDTKGPEIRTIKL
EGGNDVSLKAGQTFTFTTDKSVVGNSETVAVTYEGFTKDLSVGNTVLVDDGLIGMEVTAIEGDKVICKVLNNGDLGE
NKGVNLPGVSIALPALAEKDKKDLIFGCEQGVDFVAASFIRKRSDVEEIRQHLKAHGGENIQIISKIENQEGLNNFD
EILEASDGIMVARGDLGVEIPVEEVIFAQKMMIEKCIRARKVVITATQMLDSMIKNPRPTRAEAGDVANAILDGTDA
VMLSGESAKGKYPLEAVTIMATICERTDRVMTSRLEFNNDSRKLRITEAVCRGAVETAEKLEAPLIVVATEGGKSAK
SVRKYFPDATILALTTNELTARQLVLSKGVVPQLVKEISSTDDFYRLGKDAALESGLAKKGDVVVMVSGALVPSGTT
NTASVHVL

>Enterobacter cloacae
MKKTKIVCTIGPKTESEEMLTKMLDAGMNVMRLNFSHGDYAEHGQRIQNLRNVMSKTGKKAAILLDTKGPEIRTIKL
EGGNDVSLKAGQTFTFTTDKSVVGNSEIVAVTYEGFTSDLSVGNTVLVDDGLIGMEVTAIEGNKVICKVLNNGDLGE
NKGVNLPGVSIALPALAEKDKQDLIFGCEQGVDFVAASFIRKRSDVVEIREHLKAHGGEKIQIISKIENQEGLNNFD
EILEASDGIMVARGDLGVEIPVEEVIFAQKMMIEKCVRARKVVITATQMLDSMIKNPRPTRAEAGDVANAILDGTDA
VMLSGESAKGKYPLEAVSIMATICERTDRVMTSRLDFNNDSRKLRITEAVCRGAVETAEKLEAPLIVVATQGGKSAR
AVRKYFPDATILALTTNETTARQLVLSKGVVAHLVKEIASTDDFYIQGKELALESGLAQKGDVVVMVSGALVPSGTT
NTASVHVL

>Phytobacter diazotrophicus
MKKTKIVCTIGPKTESEEMLAKMLDAGMNVMRLNFSHGDYAEHGQRIQNLRNVMSKTGKKAAILLDTKGPEIRTIKL
EGGNDVSLKAGQTFTFTTDKTVIGNTETVAVTYEGFTSDLQVGNTVLVDDGLIGMEVTAIEGNKVICKVLNNGDLGE
NKGVNLPGVSIALPALAEKDKQDLIFGCEQGVDFVAASFIRKRSDVEEIRQHLKAHGGENIQIISKIENQEGLNNFD
EILEASDGIMVARGDLGVEIPVEEVIFAQKMMIEKCIRARKVVITATQMLDSMIKNPRPTRAEAGDVANAILDGTDA
VMLSGESAKGKYPLEAVTIMATICERTDRVMTSRLDFNNDSRKLRITEAVCRGAVETAEKLDAPLIVVATQGGKSAR
AVRKYFPDATILALTTNETTARQLVLSKGVVAQLVEEIASTDDFYRLGKELAVQSGLAQKGDVVVMVSGALVSSGTT
NTASVHVL

>Kosakonia sacchari
MKKTKIVCTIGPKTESEEMLAKMLDAGMNVMRLNFSHGDYAEHGQRIKNLRNVMSKTGKKAAILLDTKGPEIRTIKL
EGGNDVSLKAGQTFTFTTDKSVVGNADTVAVTYEGFTNDLSVGNTVLVDDGLIGMEVTEIVGNKVICKVLNNGDLGE
NKGVNLPGVSIALPALAEKDKQDLIFGCEQGVDFVAASFIRKRSDVVEIREHLKAHGGENIQIISKIENQEGLNNFD
EILEASDGIMVARGDLGVEIPVEEVIFAQKMMIEKCIRARKVVITATQMLDSMIKNPRPTRAEAGDVANAILDGTDA
VMLSGESAKGKYPLEAVTIMATICERTDRVMTSRLDFNNDSRKLRITEAVCRGAVETAEKLEAPLIVVATQGGKSAR
AVRKYFPDATILALTTNEVTARQLVLSKGVIPQLVKEISSTDDFYRLGKEVALESGLASKGDVVVMVSGALVPSGTT
NTASVHVL

>Huaxiibacter chinensis
MKKTKIVCTIGPKTESEEMLTKMLDAGMNVMRLNFSHGDYAEHGQRIKNLRNVMSKTGKKAAILLDTKGPEIRTIKL
EGGNDVSLKAGQTFTFTTDKTVVGNNEIVAVTYEGFTSDLSVGNTVLVDDGLIGMEVTAIEGNNVICKVLNNGDLGE
NKGVNLPGVSIALPALAEKDKQDLIFGCEQGVDFVAASFIRKRSDVVEIREHLKAHGGENIQIISKIENQEGLNNFD
EILEASDGIMVARGDLGVEIPVEEVIFAQKMMIEKCVRARKVVITATQMLDSMIKNPRPTRAEAGDVANAILDGTDA
VMLSGESAKGKYPLEAVTIMATICERTDRVMTSRLDNNNDSRKLRITEAVCRGAVETAEKLEAPLIVVATQGGKSAK
AVRKYFPNATILALTTNETTARQLVLSKGVVPHLVKEIASTDDFYRLGKEVALQLVDRGLAQKGDVVVMVSGALVPS
GTTNTASVHVL

9. RpiA

>Salmonella typhimurium
MTQDELKKAVGWAALQYVQPGTIVGVGTGSTAAHFIDALGTMKGQIEGAVSSSDASTEKLKGLGIHVFDLNEVDSLG
IYVDGADEINGHMQMIKGGGAALTREKIIASVAEKFICIADASKQVDILGKFPLPVEVIPMARSAVARQLVKLGGRP
EYRQNVVTDNGNVILDVYGMEILDPIALENAINAIPGVVTVGLFANRGADVALIGTPDGVKTIVK

>Escherichia coli
MTQDELKKAVGWAALQYVQPGTIVGVGTGSTAAHFIDALGTMKGQIEGAVSSSDASTEKLKSLGIHVFDLNEVDSLG
IYVDGADEINGHMQMIKGGGAALTREKIIASVAEKFICIADASKQVDILGKFPLPVEVIPMARSAVARQLVKLGGRP
EYRQGVVTDNGNVILDVHGMEILDPIAMENAINAIPGVVTVGLFANRGADVALIGTPDGVKTIVK

>Citrobacter rodentium
MTQDELKKAVGWAALQYVQPGTIVGVGTGSTAAHFIDALGTMKGQIEGAVSSSDASTEKLKSLGIPVFDLNEVDSLG
VYVDGADEINPHMQMIKGGGAALTREKIIASVAKKFICIADASKEVDILGNFPLPVEVIPMARSAVARQLVKLGGRP
EYRQGVVTDNGNVILDVHGMEILDPVALENAINAIPGVVTVGLFANRGADVALIGTADGVKTIVK

>Klebsiella variicola
MTQDELKKAVGWAALQYVQPGTIVGVGTGSTAAHFIDALGTMKGQIEGAVSSSDASTEKLKSLGIPVFDLNSVDRLG
IYVDGADEINGHMQMIKGGGAALTREKIIASVADKFICIADASKQVDILGAFPLPVEVIPMARSAVARQLVKLGGRP
EYRQGVVTDNGNVILDVHGLEILDAVALENAINGIPGVVTVGLFANRGADVALIGTADGVKTIVK

>Cronobacter turicensis
MTQDELKKAVGWAALQYVEPGTIVGVGTGSTAAHFIDALGTMKNEIEGAVSSSDASTEKLKSLGITVFDLNEVDSLG
IYVDGADEINGQMQMIKGGGAALTREKIIASVAKKFICIADASKQVDVLGNFPLPVEVIPMARSAVARQLVKLGGRP
EYRQGVVTDNGNVILDVYGMAILDPIALENAINGIPGVVTVGLFANRGADVALIGTADGVKTIVK

>Enterobacter cloacae
MTQDELKKAVGWAALQYVQPGTIVGVGTGSTAAHFIDALGTMKGQIEGAVSSSDASTEKLKSLGITVFDLNEVDRLG
IYVDGADEINGHMQMIKGGGAALTREKIIASVADKFICIADASKQVDILGKFPLPVEVIPMARSAVARELVKLGGRP
EYRQGVVTDNGNVILDVHGLEILDAIALENAINGIPGVVTVGLFANRGADVALIGTADGVKTIVK

>Phytobacter diazotrophicus
MTQDELKKAVGWAALKYVEPGTIVGVGTGSTAAHFIDALGTMKGQIEGAVSSSDASTEKLKGLGITVFDLNEVDRLG
IYVDGADEINGQMQMIKGGGAALTREKIIASVADKFICIADASKQVEILGKFPLPVEVIPMARSAVARELVKLGGRP
EYRQNVVTDNGNVILDVFGLEIIDPVALENTINGIPGVVTVGLFANRGADVALIGSADGVKTITK

>Kosakonia sacchari
MTQDELKKAVGWAALKYVEPGTIVGVGTGSTAAHFIDALGTMKGQIEGAVSSSDASTEKLKSLGITVFDLNEVDRLG
IYVDGADEINGQMQMIKGGGAALTREKVIASVADKFICIADASKQVDILGKFPLPVEVIPMARSAVARELVKLGGRP
EYRQNVVTDNGNVILDVYGLEIIDPIALENSINALPGVVTVGLFANRGADVALIGTADGVKTIVK

>Huaxiibacter chinensis
MTQDELKKAVGWAALQYVQPGTIVGVGTGSTAAHFIDALGTMKGQIEGAVSSSDASTEKLKSLGITVFDLNEVDRLG
IYVDGADEINGHMQMIKGGGAALTREKIIASVADKFICIADASKQVDILGNFPLPVEVIPMARSAVARQLVRLGGRP
EYRQGVVTDNGNVILDVHGLEILDAVAMENAINAIPGVVTVGLFANRGADVALIGTADGVKTIVK

10. ThrC

```
>Salmonella typhimurium
MKLYNLKDHNEQVSFAQAVTQGLGKQQGLFFPHDLPEFSLTEIDEMLNQDFVSRSAKILSAFIGDEIPQQILEERVR
AAFAFPAPVAQVESDVGCLELFHGPTLAFKDFGGRFMAQMLTHISGDKPVTILTATSGDTGAAVAHAFYGLENVRVV
ILYPRGKISPLQEKLFCTLGGNIETVAIDGDFDACQALVKQAFDDEELKTALGLNSANSINISRLLAQICYYFEAVA
QLPQGARNQLVISVPSGNFGDLTAGLLAKSLGLPVKRFIAATNVNDTVPRFLHDGKWAPKATQATLSNAMDVSQPNN
WPRVEELFRRKIWRLTELGYAAVDDTTTQQTMRELKAKGYISEPHAAVAYRALRDQLNPGEYGLFLGTAHPAKFKES
VESILGETLALPEALAERADLPLLSHHLPADFAALRKLMMTRQ

>Escherichia coli
MKLYNLKDHNEQVSFAQAVTQGLGKNQGLFFPHDLPEFSLTEIDEMLKLDFVTRSAKILSAFIGDEIPQEILEERVR
AAFAFPAPVANVESDVGCLELFHGPTLAFKDFGGRFMAQMLTHIAGDKPVTILTATSGDTGAAVAHAFYGLPNVKVV
ILYPRGKISPLQEKLFCTLGGNIETVAIDGDFDACQALVKQAFDDEELKVALGLNSANSINISRLLAQICYYFEAVA
QLPQETRNQLVVSVPSGNFGDLTAGLLAKSLGLPVKRFIAATNVNDTVPRFLHDGQWSPKATQATLSNAMDVSQPNN
WPRVEELFRRKIWQLKELGYAAVDDETTQQTMRELKELGYTSEPHAAVAYRALRDQLNPGEYGLFLGTAHPAKFKES
VEAILGETLDLPKELAERADLPLLSHNLPADFAALRKLMMNHQ

>Citrobacter rodentium
MKLYNLKDHNEQVNFAQAITQGLGKNQGLFFPHDLPEFSLTEVDEMLSQDFVSRSAKILSAFIGDEIPQQILEERVR
AAFAFPAPVAQVESDVGCLELFHGPTLAFKDFGGRFMAQMLTQISGDRPVTILTATSGDTGAAVAHAFYGLPNVRVV
ILYPRGKISPLQEKLFCTLGGNIETVAIDGDFDACQALVKQAFDDHELKDALGLNSANSINISRLLAQICYYFEAVA
QLPQEARNQLVISVPSGNFGDLTAGLLAKSLGLPVKRFIAATNVNDTVPRYLQEGKWTPKATQATLSNAMDVSQPNN
WPRVEELFRRKIWRLNELGYAAVDDATTQETMRELQAKGYLSEPHAAVAYRALRDQLNPGEYGLFLGTAHPAKFKES
VEAILGETLELPKALAERADLPLLSHELPADFAALRKLMMSRR

>Klebsiella variicola
MKLYNLKDHNEQVSFAQAVTQGLGKHQGLFFPHDLPEFSLTEIDDMLAQDFVTRSAKILSAFIGDEIPQDVLQQRVR
AAFAFPAPVSKVQDDVGCLELFHGPTLAFKDFGGRFMAQMLTHIAGDKPVTILTATSGDTGAAVAHAFYGLPNVKVV
ILYPRGKISPLQEKLFCTLGGNIETVAIDGDFDACQALVKQAFDDEELKATLGLNSANSINISRLLAQICYYFEAAA
QLPQEARNQLVISVPSGNFGDLTAGLLAKSLGLPIKRFIAATNANDTVPRYLQGGEWAPKATQATLSNAMDVSQPNN
WPRVEELFRRKIWRLSELGYAAVDDETTKAAMRELKAIGYISEPHAAIAWRALRDQLQPGEYGLFLGTAHPAKFKES
VEEILQETLPLPKELADRADLPLLSHNLPADFAALRKLMMG

>Cronobacter turicensis
MKLYNLKDHNEQVSFAQAVTQGLGKNQGLFFPHDLPEFSLTEIDEMLEMDFVSRSSKILSAYIGDEIPADTLRQRVE
KAFTFPAPVSQVAEDIGCLELFHGPTLAFKDFGGRFMAQMLTQISGDKPVTILTATSGDTGAAVAHAFYGLKNVQVV
ILYPRGKISPLQEKLFCTLGGNIETVAIDGDFDACQALVKQAFDDEELKTKLGLNSANSINISRLLAQICYYFEAVA
QLPQEARNQLVVSVPSGNFGDLTAGLLAKSAGLPVKRFIAATNANDTVPRFLQHGEWLPNATVATLSNAMDVSQPNN
WPRVEELFRRKIWRLNELGVATIDDEITKATMRELRELGYLSEPHAAVAYRALRDQLRPGEYGLFLGTAHPAKFKES
VEEVLGETLELPAELAERVDLPLLSHSLPADFAALREFMMSKAP

>Enterobacter cloacae
MKLYNLKDHNEQVSFAQAVTQGLGKNQGLFFPHDLPEFQLTDIDELLKQDFVTRSTKILSAFIGDEIPQELLEERVR
AAFAFPAPVQQVEPDVGCLELFHGPTLAFKDFGGRFMAQMLTHISGDKPVTILTATSGDTGAAVAHAFYGLKNVRVV
ILYPKGKISPLQEKLFCTLGGNIETVAVDGDFDACQALVKQAFDDEELKAALGLNSANSINISRLLAQICYYFEAVA
QLPQEARNQLVVSVPSGNFGDLTAGLLAKSLGLPVKRFIAATNANDTVPRFLKDGKWAPNATQATLSNAMDVSQPNN
WPRVEELFRRKVWRLGDLGYAAVTDETTKSTMRELKAVGYTSEPHAAIAYRALRDQLNPGEYGLFLGTAHPAKFKES
VEEILGETLPLPKELADRADLPLLSHELPADFAALRKLMMTRA

>Phytobacter diazotrophicus
MKLYNLKDHNEQVSFAQAVTQGLGNNQGLFFPHDLPEFSLTEIDDMLTMDFVARSAKILGAFIGDEIPQDILEARVR
DAFAFPAPVQPVESDVGCLELFHGPTLAFKDFGGRFMAQMLTTISGDKPVTILTATSGDTGAAVAHAFYGLKNVRVV
ILYPNGKISPLQEKLFCTLGGNIETVAIGADFDACQALVKQAFDDEELKVALGLNSANSINISRLLAQICYYFEAVA
QLPQEARNQLVVSVPSGNFGDLTAGLLAKSLGLPIKRFIAATNANDTVPRFLQDGEWKPKATQATLSNAMDVSQPNN
WPRVEELFRRKIWRLNELGYAAVDDETTKETMRELKGKGYLSEPHAAIAYRALRDQLHPGEYGLFLGTAHPAKFKES
VEEILGETLPLPKELAERADLELLSHHLPADFAELRKLMMSKA
```

>Kosakonia sacchari
MKLYNLKDHNEQVSFAQAVTQGLGKNQGLFFPHDLPEFNLTEVDEMLEQDFVTRSAKILAAFIGDEIPEEQLKARVR
AAFAFPAPVKPVESDVGCLELFHGPTLAFKDFGGRFMAQMLSAISGDKPVTILTATSGDTGAAVAHAFYGMKNVRVV
ILYPQGKISPLQEKLFCTLGGNIETVAIDADFDACQALVKQAFDDEELKTALGLNSANSINISRLLAQICYYFEAVA
QLPQEARNQLVVSVPSGNFGDLTAGLLAKSLGLPIKRFIAATNANDTVPRFLKEGQWQPKATQATLSNAMDVSQPNN
WPRVEELFRRKIWRLTELGYAAVDDETTKATIRELKGKGYISEPHAAIAYRALRDQLQPGEYGLFLGTAHPAKFKES
VEAILGETLPLPKELADRADLPLLSHQLPADFSALRQLMMSKA

>Huaxiibacter chinensis
MKLYNLKDHNEQVSFAQAVTQGLGKNQGLFFPHDLPEFSLTEIDDMLKQDFVSRSAKIVSAFIGEEIPQELLEERIR
TAFTFPAPVKSVEPDIGCLELFHGPTLAFKDFGGRFMAQMLTHISGDKPVTILTATSGDTGAAVAHAFYGLKNVRVV
ILYPKGKISPLQEKLFCTLGGNIETVAIDGDFDACQALVKQAFDDEELKAALGLNSANSINISRLLAQICYYFEAVA
QLPQEARNQLVISVPSGNFGDLTAGLLAKSLGLPVKRFIAATNANDTVPRFLKDGTWAPNATKATLSNAMDVSQPNN
WPRVEELFRRKIWRLGDLGYAAVTDETTKATMRELKAVGYTSEPHAAIAYRALRDQLNPGEYGLFLGTAHPAKFKES
VDEILGESLPLPKELAERADLPLLSHELPADFAALRKLMMTRG

PART 2: AMINO ACID SEQUENCE ALIGNMENTS

****Note that *H. chinensis* is denoted as "Lelliottia" below. This is the historical designation for this species (it was re-classified as *H. chinensis* in 2022)*****

Gene Regulation:

1. H-NS

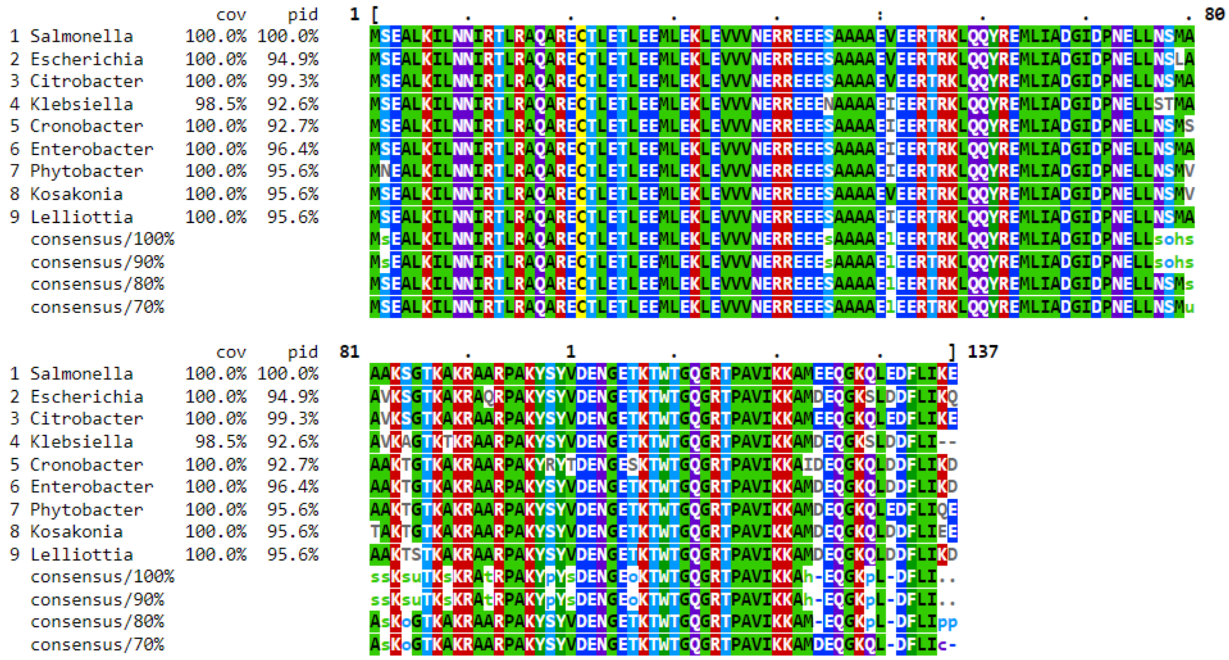


```
#  Percent Identity  Matrix - created by Clustal2.1
#
#
```

1: Salmonella	100.00	94.89	99.27	92.59	92.70	96.35	95.62	95.62	95.62
2: Escherichia	94.89	100.00	95.62	94.07	91.24	94.89	91.97	93.43	94.16
3: Citrobacter	99.27	95.62	100.00	93.33	91.97	95.62	94.89	94.89	94.89
4: Klebsiella	92.59	94.07	93.33	100.00	91.11	94.81	92.59	92.59	94.07
5: Cronobacter	92.70	91.24	91.97	91.11	100.00	96.35	93.43	93.43	95.62
6: Enterobacter	96.35	94.89	95.62	94.81	96.35	100.00	96.35	96.35	99.27
7: Phytobacter	95.62	91.97	94.89	92.59	93.43	96.35	100.00	96.35	95.62
8: Kosakonia	95.62	93.43	94.89	92.59	93.43	96.35	96.35	100.00	95.62
9: Lelliottia	95.62	94.16	94.89	94.07	95.62	99.27	95.62	95.62	100.00

2. HupB

```
                     cov    pid  1 [        .         .         .         .         :         .         .         . 80
1 Salmonella     100.0% 100.0%    MNKSQLIEKIAAGADISKAAAGRALDAIIASVTESLKEGDDVALVGFGTFAVKERAARTGRNPQTGKEITIAAAKVPSFR
2 Escherichia    100.0%  98.9%    MNKSQLIDKIAAGADISKAAAGRALDAIIASVTESLKEGDDVALVGFGTFAVKERAARTGRNPQTGKEITIAAAKVPSFR
3 Citrobacter    100.0%  96.7%    MNKSQLIDKIAAGADISKAAAGRALDAIIASVTESLKEGDDVALVGFGTFSVKERAARTGRNPQTGKEITIAAAKVPGFR
4 Klebsiella     100.0%  94.4%    MNKSQLIDKIAAGADISKAAAGRALDALIASVTESLQAGDDVALVGFGTFAVKERAARTGRNPQTGKEITIAAAKVPGFR
5 Cronobacter    100.0%  91.1%    MNKSQLIDKIAAGADISKAAAGRALDALIDSVTESLQSGDEVALVGFGTFAVRERAARTGRNPQTGKEITIAAAKVPGFR
6 Enterobacter   100.0%  94.4%    MNKSQLIDKIAAGADISKAAAGRALDALIASVTESLQAGDDVALVGFGTFAVKERAARTGRNPQTGKEITIAAAKVPGFR
7 Phytobacter    100.0%  96.7%    MNKSQLIDKIAAGADISKAAAGRALDALIASVTESLKEGDDVALVGFGTFAVKERAARTGRNPQTGKEITIAAAKVPGFR
8 Kosakonia      100.0%  87.8%    MNKSQLVDKIAADADISKAAAGRVLDAFIAAVTDSLKSGDDVALVGFGSFVVKERAARTGRNPQTGKEITIAAAKVPGFR
9 Lelliottia     100.0%  95.6%    MNKSQLIDKIAAGADISKAAAGRALDALIASVTESLQEGDDVALVGFGTFAVKERAARTGRNPQTGKEITIAAAKVPGFR
  consensus/100%                  MNKSQLl-KIAAsADISKAAAGRsLDAhIsuVT-SLptGD-VALVGFGoFsV+ERAARTGRNPQTGKEITIAAAKVPuFR
  consensus/90%                   MNKSQLl-KIAAsADISKAAAGRsLDAhIsuVT-SLptGD-VALVGFGoFsV+ERAARTGRNPQTGKEITIAAAKVPuFR
  consensus/80%                   MNKSQLIDKIAAGADISKAAAGRALDAlIASVTESLptGDDVALVGFGTFuVKERAARTGRNPQTGKEITIAAAKVPuFR
  consensus/70%                   MNKSQLIDKIAAGADISKAAAGRALDAlIASVTESLppGDDVALVGFGTFAVKERAARTGRNPQTGKEITIAAAKVPGFR

                     cov    pid 81          ] 90
1 Salmonella     100.0% 100.0%    AGKALKDAVN
2 Escherichia    100.0%  98.9%    AGKALKDAVN
3 Citrobacter    100.0%  96.7%    AGKALKDAVN
4 Klebsiella     100.0%  94.4%    AGKALKDAVN
5 Cronobacter    100.0%  91.1%    AGKALKDAVN
6 Enterobacter   100.0%  94.4%    AGKALKDAVN
7 Phytobacter    100.0%  96.7%    AGKALKDAVN
8 Kosakonia      100.0%  87.8%    AGKALKDAVN
9 Lelliottia     100.0%  95.6%    AGKALKDAVN
  consensus/100%                  AGKALKDAVN
  consensus/90%                   AGKALKDAVN
  consensus/80%                   AGKALKDAVN
  consensus/70%                   AGKALKDAVN
```

```
#  Percent Identity  Matrix - created by Clustal2.1
#
#
```

1: Salmonella	100.00	98.89	96.67	94.44	91.11	94.44	96.67	87.78	95.56
2: Escherichia	98.89	100.00	97.78	95.56	92.22	95.56	97.78	88.89	96.67
3: Citrobacter	96.67	97.78	100.00	95.56	92.22	95.56	97.78	90.00	96.67
4: Klebsiella	94.44	95.56	95.56	100.00	95.56	100.00	97.78	88.89	98.89
5: Cronobacter	91.11	92.22	92.22	95.56	100.00	95.56	94.44	86.67	95.56
6: Enterobacter	94.44	95.56	95.56	100.00	95.56	100.00	97.78	88.89	98.89
7: Phytobacter	96.67	97.78	97.78	97.78	94.44	97.78	100.00	90.00	98.89
8: Kosakonia	87.78	88.89	90.00	88.89	86.67	88.89	90.00	100.00	88.89
9: Lelliottia	95.56	96.67	96.67	98.89	95.56	98.89	98.89	88.89	100.00

3. Arac

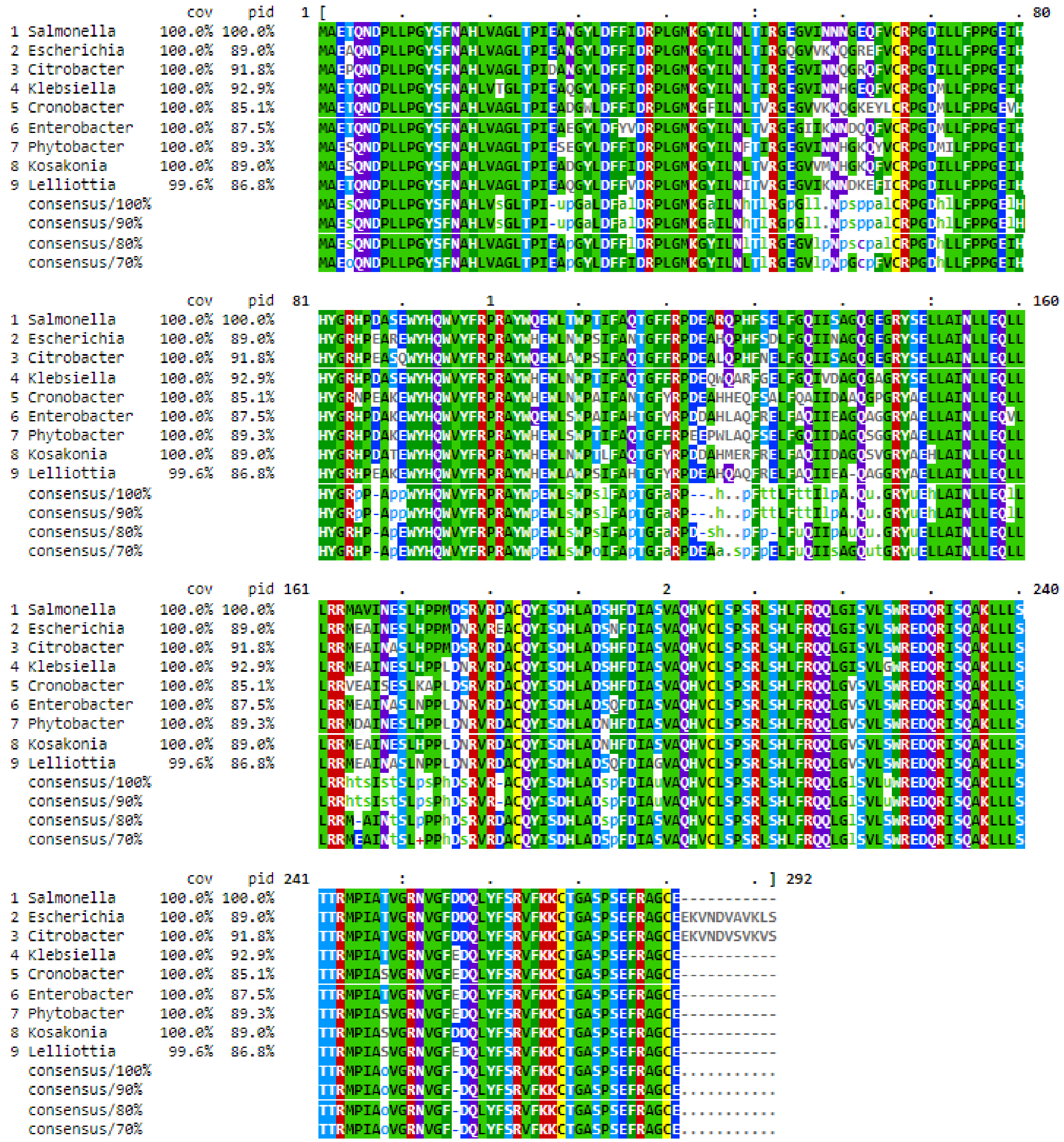

```
#  Percent Identity  Matrix - created by Clustal2.1
#
#
```

1: Salmonella	100.00	92.53	95.37	92.88	85.05	87.54	89.32	88.97	87.14
2: Escherichia	92.53	100.00	92.81	90.04	87.19	86.12	86.83	88.61	88.21
3: Citrobacter	95.37	92.81	100.00	91.10	84.70	86.83	87.54	87.90	87.86
4: Klebsiella	92.88	90.04	91.10	100.00	85.77	87.90	91.46	90.04	87.86
5: Cronobacter	85.05	87.19	84.70	85.77	100.00	86.12	86.48	87.90	88.21
6: Enterobacter	87.54	86.12	86.83	87.90	86.12	100.00	88.61	88.97	93.93
7: Phytobacter	89.32	86.83	87.54	91.46	86.48	88.61	100.00	90.75	88.21
8: Kosakonia	88.97	88.61	87.90	90.04	87.90	88.97	90.75	100.00	89.29
9: Lelliottia	87.14	88.21	87.86	87.86	88.21	93.93	88.21	89.29	100.00

4. SoxS

```
                     cov    pid    1 [         .         .         .         .         :         .         .         . 80
1 Salmonella      100.0% 100.0%     MSHQQIIQTLIEWIDEHIDQPLNIDVVAKKSGYSKWYLQRMFRTVTHQTLGEYIRQRRLLLAAVELRTTERPIFDIAMDL
2 Escherichia     100.0%  95.3%     MSHQKIIQDLIAWIDEHIDQPLNIDVVAKKSGYSKWYLQRMFRTVTHQTLGDYIRQRRLLLAAVELRTTERPIFDIAMDL
3 Citrobacter     100.0%  95.3%     MSHQKIIQDLIAWIDEHIDQPLNIDVVAKKSGYSKWYLQRMFRTVTHQTLGDYIRQRRLLLAAVELRTTERPIFDIAMDL
4 Klebsiella      100.0%  89.0%     MSHQDIIQTLIEWIDEHIDQPLNIDIVARKSGYSKWYLQRMFRTVMHQTLGDYIRQRRLLLAAEALRTTQRPIFDIAMDL
5 Cronobacter     100.0%  85.0%     MSHQEIIHALTQWIDEHIDQPLNIDVVAKKSGYSKWYLQRMFRTVMHQTLGEYIRKRRLQLAAQELRTTRRPIFDIAMDY
6 Enterobacter    100.0%  93.5%     MSHQQIIQTLIEWIDEHIDQPLNIDVVAKKSGYSKWYLQRMFRTVMHQTLGEYIRQRRLLLAAQALRSTQRPIFDIAMDL
7 Phytobacter     100.0%  90.7%     MSHQQIIQTLTEWIDEHIDQPLNIDVVARKSGYSKWYLQRMFRTVMHQTLGDYIRQRRLLMAAEALRTTRRPIFDIAMDL
8 Kosakonia       100.0%  91.6%     MSHQQIIQTLIEWIDEHIDQPMNIDVVARKSGYSKWYLQRMFRTVMHQTLGDYIRQRRLLLAAEALRTTQRPIFDIAMDL
9 Lelliottia      100.0%  92.6%     MSHQQIIQTLIEWIDEHIDQPLSIDTVAKKSGYSKWYLQRMFRTVMHQTLGEYIRQRRLLLAAQALRTTKRPIFDIAMDL
  consensus/100%                    MSHQpIIpsLhtWIDEHIDQPhsIDhVA+KSGYSKWYLQRMFRTVhHQTLG-YIRpRRL.hAA.tLRotpRPIFDIAMDh
  consensus/90%                     MSHQpIIpsLhtWIDEHIDQPhsIDhVA+KSGYSKWYLQRMFRTVhHQTLG-YIRpRRL.hAA.tLRotpRPIFDIAMDh
  consensus/80%                     MSHQpIIQsLhtWIDEHIDQPLNIDlVA+KSGYSKWYLQRMFRTVhHQTLG-YIRQRRLLLAA.tLRTTpRPIFDIAMDL
  consensus/70%                     MSHQpIIQsLIpWIDEHIDQPLNIDVVA+KSGYSKWYLQRMFRTVhHQTLG-YIRQRRLLLAA.tLRTTpRPIFDIAMDL

                     cov    pid   81          .         1         ] 109
1 Salmonella      100.0% 100.0%     GYVSQQTFSRVFRREFDRTPSDYRHRL--
2 Escherichia     100.0%  95.3%     GYVSQQTFSRVFRRQFDRTPSDYRHRL--
3 Citrobacter     100.0%  95.3%     GYVSQQTFSRVFRRQFDRTPSDYRHRL--
4 Klebsiella      100.0%  89.0%     GYVSQQTFSRVFRREFDRTPSDYRHQISA
5 Cronobacter     100.0%  85.0%     GYVSQQTFSRIFRRQFDRTPSDYRQSA--
6 Enterobacter    100.0%  93.5%     GYVSQQTFSRVFRREFDRTPSDYRHQLN-
7 Phytobacter     100.0%  90.7%     GYVSQQTFSRVFRREFDRTPSDYRHQA--
8 Kosakonia       100.0%  91.6%     GYVSQQTFSRVFRREFDRTPSDYRHQA--
9 Lelliottia      100.0%  92.6%     GYVSQQTFSRVFRREFDRTPSDYRHQLH-
  consensus/100%                    GYVSQQTFSRlFRRpFDRTPSDYRpph..
  consensus/90%                     GYVSQQTFSRlFRRpFDRTPSDYRpph..
  consensus/80%                     GYVSQQTFSRVFRRpFDRTPSDYRHph..
  consensus/70%                     GYVSQQTFSRVFRRpFDRTPSDYRHph..
```

```
#  Percent Identity  Matrix - created by Clustal2.1
#
#
```

1: Salmonella	100.00	95.33	95.33	90.65	85.05	94.39	90.65	91.59	93.46
2: Escherichia	95.33	100.00	100.00	88.79	85.05	89.72	87.85	88.79	88.79
3: Citrobacter	95.33	100.00	100.00	88.79	85.05	89.72	87.85	88.79	88.79
4: Klebsiella	90.65	88.79	88.79	100.00	82.24	92.59	94.39	96.26	91.67
5: Cronobacter	85.05	85.05	85.05	82.24	100.00	85.05	85.05	83.18	84.11
6: Enterobacter	94.39	89.72	89.72	92.59	85.05	100.00	92.52	94.39	95.37
7: Phytobacter	90.65	87.85	87.85	94.39	85.05	92.52	100.00	96.26	91.59
8: Kosakonia	91.59	88.79	88.79	96.26	83.18	94.39	96.26	100.00	92.52
9: Lelliottia	93.46	88.79	88.79	91.67	84.11	95.37	91.59	92.52	100.00

5. PurR

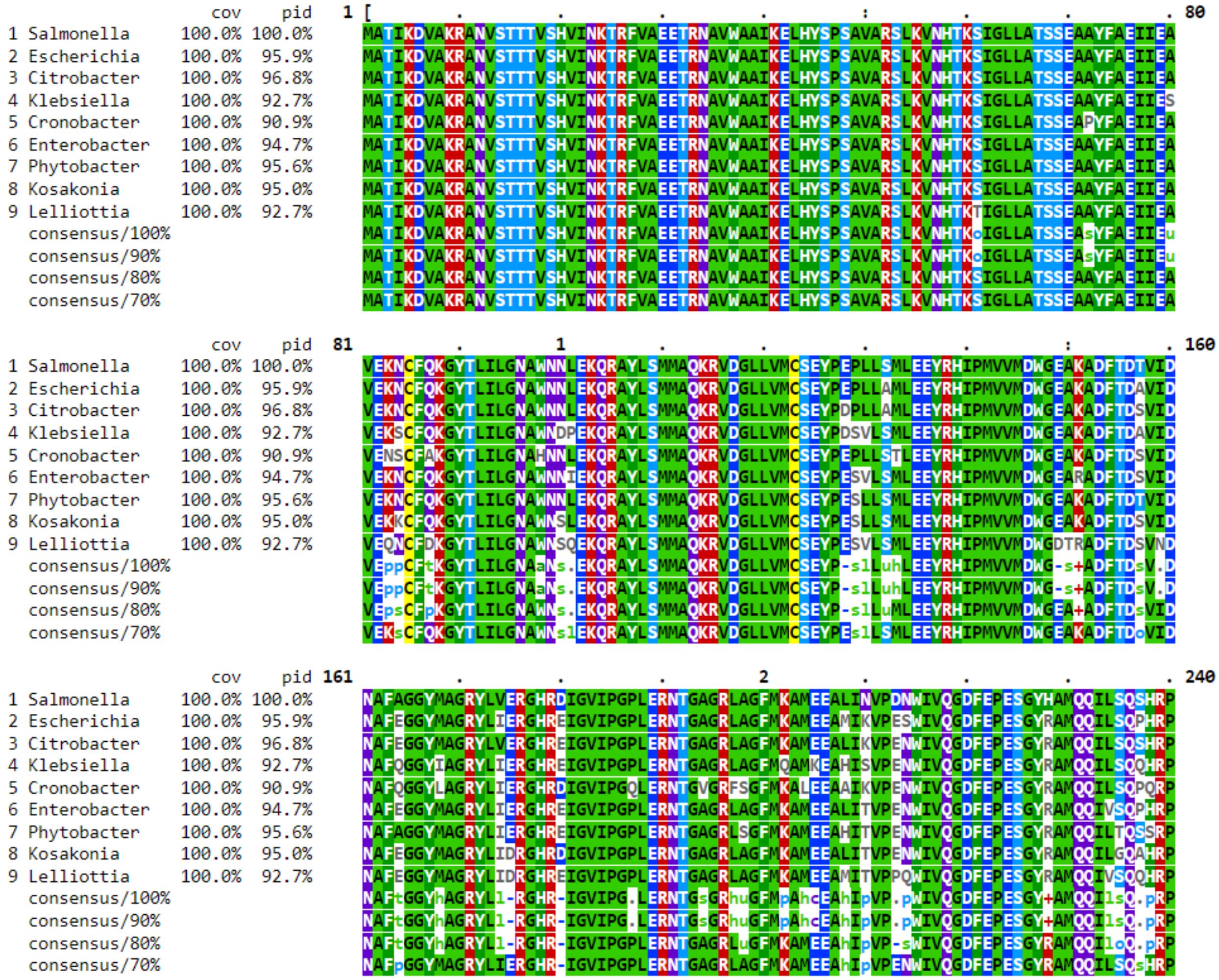

cov pid
1 Salmonella 100.0% 100.0%
2 Escherichia 100.0% 95.9%
3 Citrobacter 100.0% 96.8%
4 Klebsiella 100.0% 92.7%
5 Cronobacter 100.0% 90.9%
6 Enterobacter 100.0% 94.7%
7 Phytobacter 100.0% 95.6%
8 Kosakonia 100.0% 95.0%
9 Lelliottia 100.0% 92.7%
consensus/100%
consensus/90%
consensus/80%
consensus/70%

5. PurR (part 2)

```
                  cov    pid  241        :         .         .         .         .         3         .         . 320
1 Salmonella      100.0% 100.0%   TAVFCGGDIMAMGALCAADEMGLRVPQDVSVIGYDNVRNARYFTPALTTIHQPKDSLGETAFNMLLDRIVNKREESQSIE
2 Escherichia     100.0%  95.9%   TAVFCGGDIMAMGALCAADEMGLRVPQDVSLIGYDNVRNARYFTPALTTIHQPKDSLGETAFNMLLDRIVNKREEPQSIE
3 Citrobacter     100.0%  96.8%   TAVFCGGDIMAMGALCAADEMGLRVPQDVSLIGYDNVRNARFFTPALTTIHQPKDSLGETAFNMLLDRIVNKREESQSIE
4 Klebsiella      100.0%  92.7%   TAVFCGGDIMAMGAICAADEMGLRVPQDISLIGYDNVRNARYFSPALTTIHQPKDSLGEAAFNMLLDRIVNKREESQSIE
5 Cronobacter     100.0%  90.9%   TAVFCGGDIMAMGAICAADEMGLRVPQDISIIGYDNVRNARFFSPALTTIHQPKETLGQTAFNMLLDRIVNKREESQTIE
6 Enterobacter    100.0%  94.7%   TAVFCGGDIMAMGALCAADELGLRVPQDISVIGYDNVRNARFFTPALTTIHQPKDSLGETAFNMLMDRIVNKREESQSIE
7 Phytobacter     100.0%  95.6%   TAVFCGGDIMAMGALCAADEMGLRVPQDISVIGYDNVRNSRFFTPSLTTIHQPKDSLGETAFNMLLDRIVNKREESQSIE
8 Kosakonia       100.0%  95.0%   TAVFCGGDIMAMGALCAADEMGLRVPQDISLIGYDNVRNSRFFTPALTTIHQPKDSLGETAFTMLLDRIVNKREESQSIE
9 Lelliottia      100.0%  92.7%   TAVFCGGDIMAMGALCAADEMGLRVPQDISVIGYDNVRNARYFTPALTTIHQPKDSLGETAFNMLLDRIVNKREESQSIE
  consensus/100%                  TAVFCGGDIMAMGAlCAADEhGLRVPQDlSlIGYDNVRNuRaFoPuLTTIHQPK-oLGpsAFsMLhDRIVNKREEsQoIE
  consensus/90%                   TAVFCGGDIMAMGAlCAADEhGLRVPQDlSlIGYDNVRNuRaFoPuLTTIHQPK-oLGpsAFsMLhDRIVNKREEsQoIE
  consensus/80%                   TAVFCGGDIMAMGAlCAADEMGLRVPQDlSlIGYDNVRNuRaFoPALTTIHQPKDSLGETAFNMLLDRIVNKREESQSIE
  consensus/70%                   TAVFCGGDIMAMGALCAADEMGLRVPQDlSlIGYDNVRNARaFTPALTTIHQPKDSLGETAFNMLLDRIVNKREESQSIE
```

```
                  cov    pid  321        .         .] 341
1 Salmonella      100.0% 100.0%   VHPRLVERRSVADGPFRDYRR
2 Escherichia     100.0%  95.9%   VHPRLIERRSVADGPFRDYRR
3 Citrobacter     100.0%  96.8%   VHPRLIERRSVADGPFRDYRR
4 Klebsiella      100.0%  92.7%   VHPRLVERRSVADGPFVDYRR
5 Cronobacter     100.0%  90.9%   VHPRLIERRSVADGPFRDYRR
6 Enterobacter    100.0%  94.7%   VHPRLIERRSVADGPFRDYRR
7 Phytobacter     100.0%  95.6%   VHPRLIERRSVADGPFRDYRR
8 Kosakonia       100.0%  95.0%   VHPRLVERRSVADGPFRDYRR
9 Lelliottia      100.0%  92.7%   VHPRLIERRSVADGPFRDYRR
  consensus/100%                  VHPRLlERRSVADGPFhDYRR
  consensus/90%                   VHPRLlERRSVADGPFhDYRR
  consensus/80%                   VHPRLlERRSVADGPFRDYRR
  consensus/70%                   VHPRLlERRSVADGPFRDYRR
```

```
#  Percent Identity  Matrix - created by Clustal2.1
#
#

     1: Salmonella     100.00   95.89   96.77   92.67   90.91   94.72   95.60   95.01   92.67
     2: Escherichia     95.89  100.00   97.65   93.26   91.50   95.31   95.01   94.72   93.55
     3: Citrobacter     96.77   97.65  100.00   93.26   91.79   95.89   95.60   95.60   92.96
     4: Klebsiella      92.67   93.26   93.26  100.00   90.03   92.96   92.67   92.96   91.50
     5: Cronobacter     90.91   91.50   91.79   90.03  100.00   90.91   91.50   90.91   88.56
     6: Enterobacter    94.72   95.31   95.89   92.96   90.91  100.00   95.60   95.31   95.31
     7: Phytobacter     95.60   95.01   95.60   92.67   91.50   95.60  100.00   95.60   92.96
     8: Kosakonia       95.01   94.72   95.60   92.96   90.91   95.31   95.60  100.00   93.55
     9: Lelliottia      92.67   93.55   92.96   91.50   88.56   95.31   92.96   93.55  100.00
```

6. Crp

		cov	pid	1 [. . . . : . . . 80
1	Salmonella	100.0%	100.0%	MVLGKPQTDPTLEWFLSHCHIHKYPSKSTLIHQGEKAETLYYIVKGSVAVLIKDEEGKEMILSYLNQGDFIGELGLFEEG
2	Escherichia	100.0%	99.5%	MVLGKPQTDPTLEWFLSHCHIHKYPSKSTLIHQGEKAETLYYIVKGSVAVLIKDEEGKEMILSYLNQGDFIGELGLFEEG
3	Citrobacter	100.0%	100.0%	MVLGKPQTDPTLEWFLSHCHIHKYPSKSTLIHQGEKAETLYYIVKGSVAVLIKDEEGKEMILSYLNQGDFIGELGLFEEG
4	Klebsiella	100.0%	100.0%	MVLGKPQTDPTLEWFLSHCHIHKYPSKSTLIHQGEKAETLYYIVKGSVAVLIKDEEGKEMILSYLNQGDFIGELGLFEEG
5	Cronobacter	100.0%	100.0%	MVLGKPQTDPTLEWFLSHCHIHKYPSKSTLIHQGEKAETLYYIVKGSVAVLIKDEEGKEMILSYLNQGDFIGELGLFEEG
6	Enterobacter	100.0%	100.0%	MVLGKPQTDPTLEWFLSHCHIHKYPSKSTLIHQGEKAETLYYIVKGSVAVLIKDEEGKEMILSYLNQGDFIGELGLFEEG
7	Phytobacter	100.0%	100.0%	MVLGKPQTDPTLEWFLSHCHIHKYPSKSTLIHQGEKAETLYYIVKGSVAVLIKDEEGKEMILSYLNQGDFIGELGLFEEG
8	Kosakonia	100.0%	100.0%	MVLGKPQTDPTLEWFLSHCHIHKYPSKSTLIHQGEKAETLYYIVKGSVAVLIKDEEGKEMILSYLNQGDFIGELGLFEEG
9	Lelliottia	100.0%	99.5%	MVLGKPQTDPTLEWFLSHCHIHKYPSKSTLIHQGEKAETLYYIVKGSVAVLIKDEEGKEMILSYLNQGDFIGELGLFEEG
	consensus/100%			MVLGKPQTDPTLEWFLSHCHIHKYPSKSTLIHQGEKAETLYYIVKGSVAVLIKDEEGKEMILSYLNQGDFIGELGLFEEG
	consensus/90%			MVLGKPQTDPTLEWFLSHCHIHKYPSKSTLIHQGEKAETLYYIVKGSVAVLIKDEEGKEMILSYLNQGDFIGELGLFEEG
	consensus/80%			MVLGKPQTDPTLEWFLSHCHIHKYPSKSTLIHQGEKAETLYYIVKGSVAVLIKDEEGKEMILSYLNQGDFIGELGLFEEG
	consensus/70%			MVLGKPQTDPTLEWFLSHCHIHKYPSKSTLIHQGEKAETLYYIVKGSVAVLIKDEEGKEMILSYLNQGDFIGELGLFEEG

		cov	pid	81 . 1 . . . . : . 160
1	Salmonella	100.0%	100.0%	QERSAWVRAKTACEVAEISYKKFRQLIQVNPDILMRLSSQMARRLQVTSEKVGNLAFLDVTGRIAQTLLNLAKQPDAMTH
2	Escherichia	100.0%	99.5%	QERSAWVRAKTACEVAEISYKKFRQLIQVNPDILMRLSAQMARRLQVTSEKVGNLAFLDVTGRIAQTLLNLAKQPDAMTH
3	Citrobacter	100.0%	100.0%	QERSAWVRAKTACEVAEISYKKFRQLIQVNPDILMRLSSQMARRLQVTSEKVGNLAFLDVTGRIAQTLLNLAKQPDAMTH
4	Klebsiella	100.0%	100.0%	QERSAWVRAKTACEVAEISYKKFRQLIQVNPDILMRLSSQMARRLQVTSEKVGNLAFLDVTGRIAQTLLNLAKQPDAMTH
5	Cronobacter	100.0%	100.0%	QERSAWVRAKTACEVAEISYKKFRQLIQVNPDILMRLSSQMARRLQVTSEKVGNLAFLDVTGRIAQTLLNLAKQPDAMTH
6	Enterobacter	100.0%	100.0%	QERSAWVRAKTACEVAEISYKKFRQLIQVNPDILMRLSSQMARRLQVTSEKVGNLAFLDVTGRIAQTLLNLAKQPDAMTH
7	Phytobacter	100.0%	100.0%	QERSAWVRAKTACEVAEISYKKFRQLIQVNPDILMRLSSQMARRLQVTSEKVGNLAFLDVTGRIAQTLLNLAKQPDAMTH
8	Kosakonia	100.0%	100.0%	QERSAWVRAKTACEVAEISYKKFRQLIQVNPDILMRLSSQMARRLQVTSEKVGNLAFLDVTGRIAQTLLNLAKQPDAMTH
9	Lelliottia	100.0%	99.5%	QERSAWVRAKSACEVAEISYKKFRQLIQVNPDILMRLSSQMARRLQVTSEKVGNLAFLDVTGRIAQTLLNLAKQPDAMTH
	consensus/100%			QERSAWVRAKoACEVAEISYKKFRQLIQVNPDILMRLSuQMARRLQVTSEKVGNLAFLDVTGRIAQTLLNLAKQPDAMTH
	consensus/90%			QERSAWVRAKoACEVAEISYKKFRQLIQVNPDILMRLSuQMARRLQVTSEKVGNLAFLDVTGRIAQTLLNLAKQPDAMTH
	consensus/80%			QERSAWVRAKTACEVAEISYKKFRQLIQVNPDILMRLSSQMARRLQVTSEKVGNLAFLDVTGRIAQTLLNLAKQPDAMTH
	consensus/70%			QERSAWVRAKTACEVAEISYKKFRQLIQVNPDILMRLSSQMARRLQVTSEKVGNLAFLDVTGRIAQTLLNLAKQPDAMTH

		cov	pid	161 . . . 2] 210
1	Salmonella	100.0%	100.0%	PDGMQIKITRQEIGQIVGCSRETVGRILKMLEDQNLISAHGKTIVVYGTR
2	Escherichia	100.0%	99.5%	PDGMQIKITRQEIGQIVGCSRETVGRILKMLEDQNLISAHGKTIVVYGTR
3	Citrobacter	100.0%	100.0%	PDGMQIKITRQEIGQIVGCSRETVGRILKMLEDQNLISAHGKTIVVYGTR
4	Klebsiella	100.0%	100.0%	PDGMQIKITRQEIGQIVGCSRETVGRILKMLEDQNLISAHGKTIVVYGTR
5	Cronobacter	100.0%	100.0%	PDGMQIKITRQEIGQIVGCSRETVGRILKMLEDQNLISAHGKTIVVYGTR
6	Enterobacter	100.0%	100.0%	PDGMQIKITRQEIGQIVGCSRETVGRILKMLEDQNLISAHGKTIVVYGTR
7	Phytobacter	100.0%	100.0%	PDGMQIKITRQEIGQIVGCSRETVGRILKMLEDQNLISAHGKTIVVYGTR
8	Kosakonia	100.0%	100.0%	PDGMQIKITRQEIGQIVGCSRETVGRILKMLEDQNLISAHGKTIVVYGTR
9	Lelliottia	100.0%	99.5%	PDGMQIKITRQEIGQIVGCSRETVGRILKMLEDQNLISAHGKTIVVYGTR
	consensus/100%			PDGMQIKITRQEIGQIVGCSRETVGRILKMLEDQNLISAHGKTIVVYGTR
	consensus/90%			PDGMQIKITRQEIGQIVGCSRETVGRILKMLEDQNLISAHGKTIVVYGTR
	consensus/80%			PDGMQIKITRQEIGQIVGCSRETVGRILKMLEDQNLISAHGKTIVVYGTR
	consensus/70%			PDGMQIKITRQEIGQIVGCSRETVGRILKMLEDQNLISAHGKTIVVYGTR

```
#  Percent Identity  Matrix - created by Clustal2.1
#
#
```

1: Salmonella	100.00	99.52	100.00	100.00	100.00	100.00	100.00	100.00	99.52
2: Escherichia	99.52	100.00	99.52	99.52	99.52	99.52	99.52	99.52	99.05
3: Citrobacter	100.00	99.52	100.00	100.00	100.00	100.00	100.00	100.00	99.52
4: Klebsiella	100.00	99.52	100.00	100.00	100.00	100.00	100.00	100.00	99.52
5: Cronobacter	100.00	99.52	100.00	100.00	100.00	100.00	100.00	100.00	99.52
6: Enterobacter	100.00	99.52	100.00	100.00	100.00	100.00	100.00	100.00	99.52
7: Phytobacter	100.00	99.52	100.00	100.00	100.00	100.00	100.00	100.00	99.52
8: Kosakonia	100.00	99.52	100.00	100.00	100.00	100.00	100.00	100.00	99.52
9: Lelliottia	99.52	99.05	99.52	99.52	99.52	99.52	99.52	99.52	100.00

7. Fnr

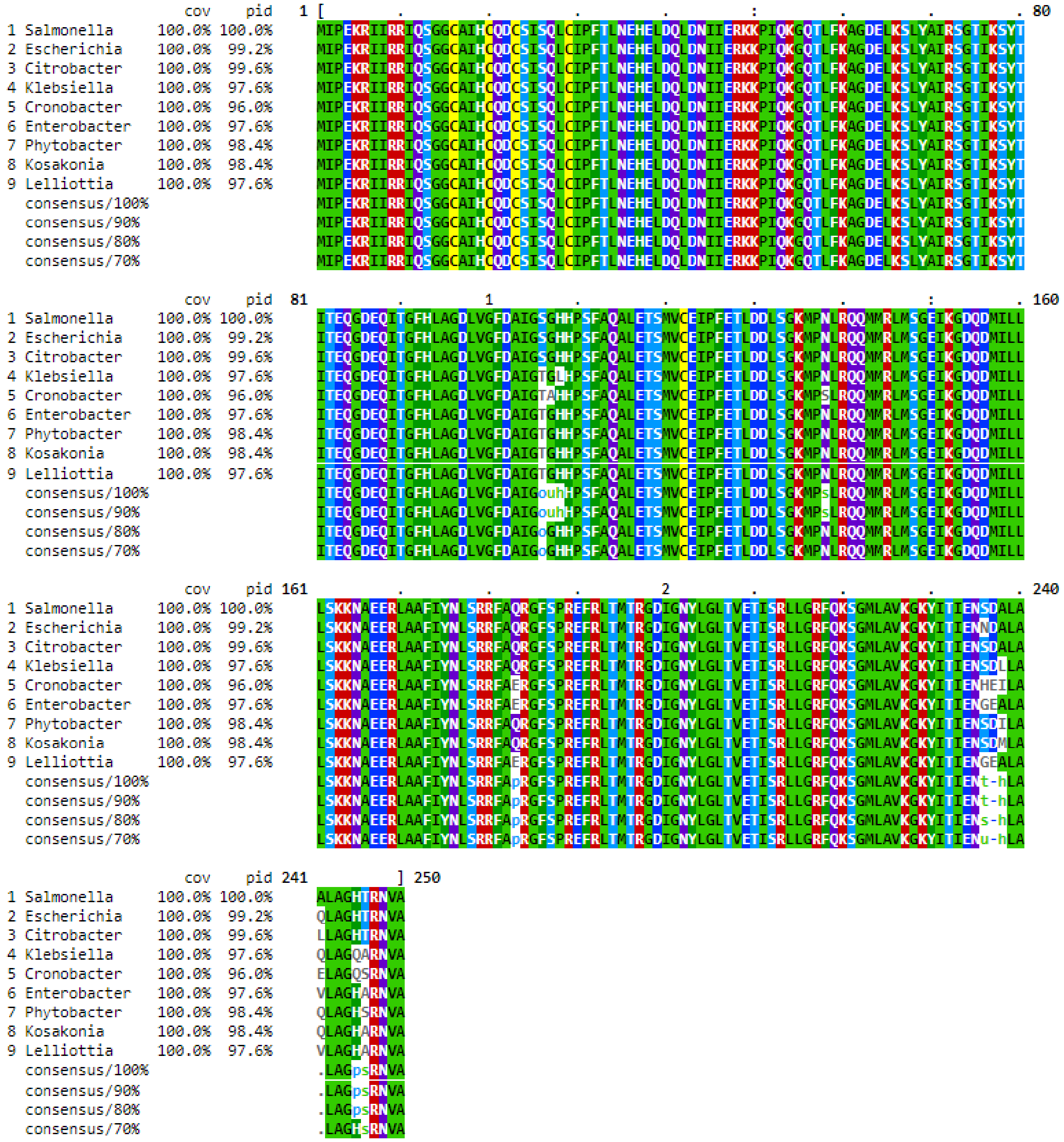

```
#  Percent Identity  Matrix - created by Clustal2.1
#
#
```

1: Salmonella	100.00	99.20	99.60	97.60	96.00	97.60	98.40	98.40	97.60
2: Escherichia	99.20	100.00	99.20	97.60	96.00	97.60	98.40	98.40	97.60
3: Citrobacter	99.60	99.20	100.00	97.60	96.00	97.60	98.40	98.40	97.60
4: Klebsiella	97.60	97.60	97.60	100.00	96.40	97.20	98.40	98.80	97.20
5: Cronobacter	96.00	96.00	96.00	96.40	100.00	97.20	97.20	96.40	97.20
6: Enterobacter	97.60	97.60	97.60	97.20	97.20	100.00	97.60	98.00	100.00
7: Phytobacter	98.40	98.40	98.40	98.40	97.20	97.60	100.00	99.20	97.60
8: Kosakonia	98.40	98.40	98.40	98.80	96.40	98.00	99.20	100.00	98.00
9: Lelliottia	97.60	97.60	97.60	97.20	97.20	100.00	97.60	98.00	100.00

8. FadR

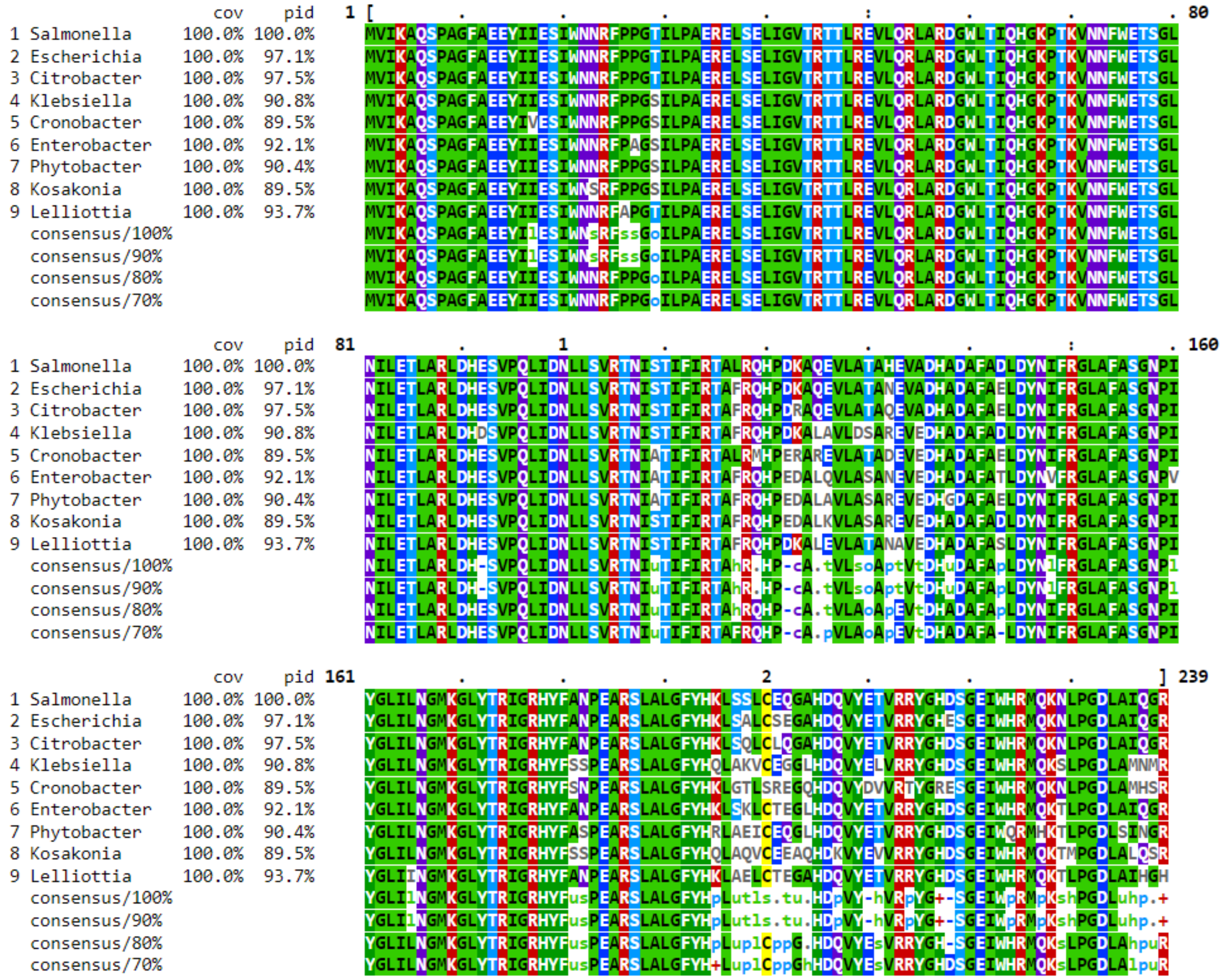

\# Percent Identity Matrix - created by Clustal2.1
\#
\#

1: Salmonella	100.00	97.07	97.49	90.79	89.54	92.05	90.38	89.54	93.72
2: Escherichia	97.07	100.00	97.49	89.96	90.38	92.89	89.96	89.12	94.56
3: Citrobacter	97.49	97.49	100.00	89.96	89.96	92.47	90.79	89.54	93.72
4: Klebsiella	90.79	89.96	89.96	100.00	87.45	90.38	92.05	92.89	89.96
5: Cronobacter	89.54	90.38	89.96	87.45	100.00	88.70	87.03	88.28	88.28
6: Enterobacter	92.05	92.89	92.47	90.38	88.70	100.00	92.47	90.79	92.47
7: Phytobacter	90.38	89.96	90.79	92.05	87.03	92.47	100.00	91.21	90.38
8: Kosakonia	89.54	89.12	89.54	92.89	88.28	90.79	91.21	100.00	89.12
9: Lelliottia	93.72	94.56	93.72	89.96	88.28	92.47	90.38	89.12	100.00

9. GntR

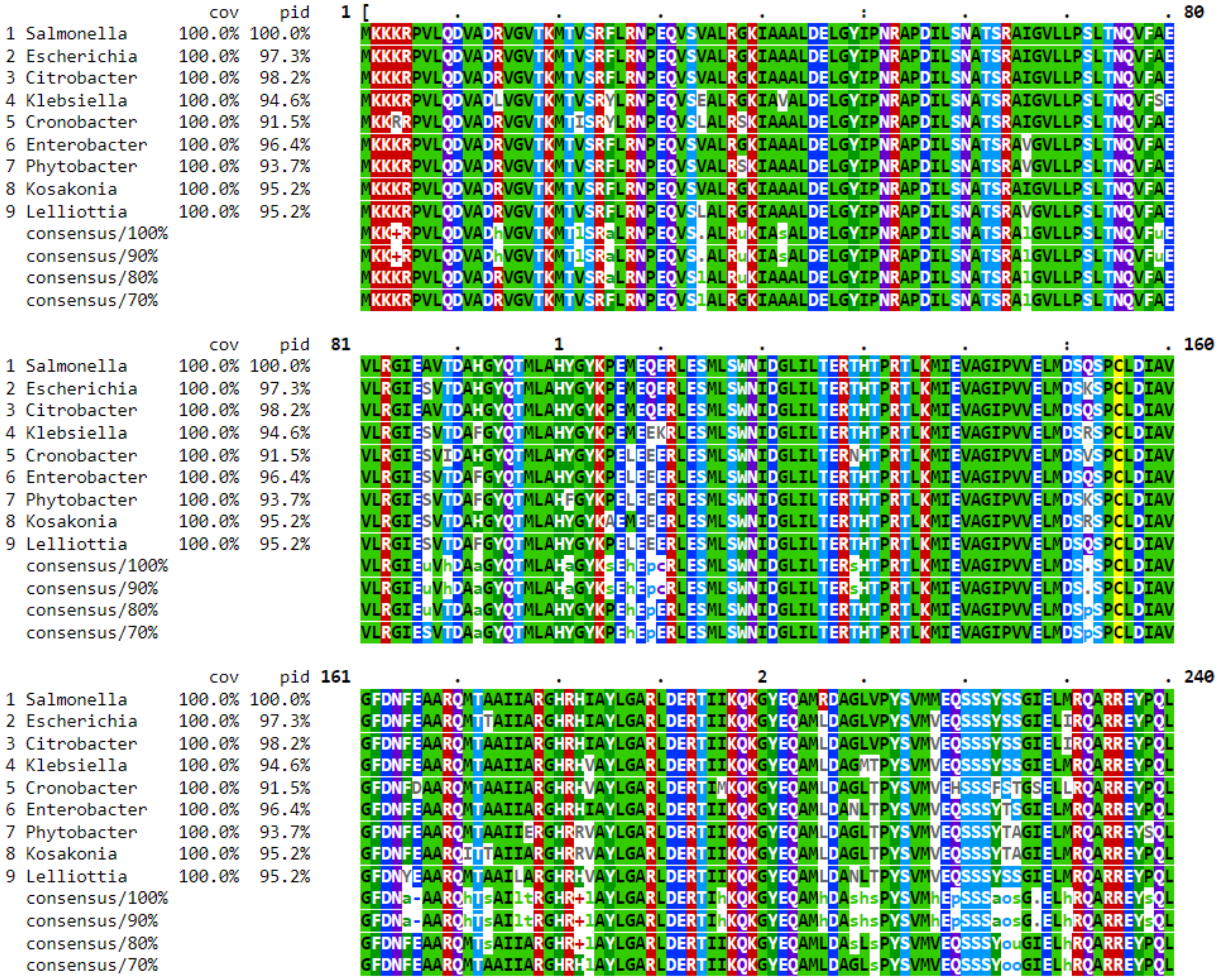
cov pid
1 Salmonella 100.0% 100.0%
2 Escherichia 100.0% 97.3%
3 Citrobacter 100.0% 98.2%
4 Klebsiella 100.0% 94.6%
5 Cronobacter 100.0% 91.5%
6 Enterobacter 100.0% 96.4%
7 Phytobacter 100.0% 93.7%
8 Kosakonia 100.0% 95.2%
9 Lelliottia 100.0% 95.2%
consensus/100%
consensus/90%
consensus/80%
consensus/70%

9. GntR (part 2)

```
                   cov    pid  241      :         .         .         .         .         3         .         . 320
1 Salmonella      100.0% 100.0%    DGIFCTNDDLAVGAAFECQRLGLKIPDDMAIAGFHGHDIGQVMEPRLASVLTPRERMGSIGAERLLARIRGETVTPKMLD
2 Escherichia     100.0%  97.3%    DGVFCTNDDLAVGAAFECQRLGLKVPDDMAIAGFHGHDIGQVMEPRLASVLTPRERMGSIGAERLLARIRGESVTPKMLD
3 Citrobacter     100.0%  98.2%    DGIFCTNDDLAVGAAFECQRLGLSIPRDMAIAGFHGHDIGQVMEPRLASVLTPRERMGSIGAERLLARIRGESVTPKMLD
4 Klebsiella      100.0%  94.6%    DGIFCTNDDLAVGAAFECQRLGLKIPDDMAIAGFHGHDIGQVMEPRLASVLTPRERMGRIGAERLLARIRGEAITPKMLD
5 Cronobacter     100.0%  91.5%    DSIFCTNDDLAIGAAFECQRLGLRIPDDMAIAGFHGHDIGQVMEPQLASVLTPRERMGRIGAERLLARIRGETVTPQMLD
6 Enterobacter    100.0%  96.4%    DGIFCTNDDLAVGAAFECQRLGLKIPDDMAIAGFHGHDIGQVMEPRLASVLTPRERMGRIGAERLLARIRGEAVTPKMLD
7 Phytobacter     100.0%  93.7%    DGIFCTNDDLAVGAAFECQRLGLKVPDDMAIAGFHGHDIGQVMEPRLASVLTPRERMGRIGAERLLARIRGESVTPKLLD
8 Kosakonia       100.0%  95.2%    DGIFCTNDDLAVGAAFECQRLGLKIPQDMAIAGFHGHDIGQVMEPRLASVLTPRERMGRIGAERLLARIRGESVTPKMLD
9 Lelliottia      100.0%  95.2%    DGIFCTNDDLAVGAAFECQRLGLKIPDDIAIAGFHGHDIGQVMEPRLASVLTPRERMGRIGAERLLARIRGEVVTPKMLD
  consensus/100%                   DulFCTNDDLAlGAAFECQRLGLplPpDhAIAGFHGHDIGQVMEPpLASVLTPRERMGpIGAERLLARIRGEslTPphLD
  consensus/90%                    DulFCTNDDLAlGAAFECQRLGLplPpDhAIAGFHGHDIGQVMEPpLASVLTPRERMGpIGAERLLARIRGEslTPphLD
  consensus/80%                    DGIFCTNDDLAVGAAFECQRLGL+lPcDMAIAGFHGHDIGQVMEPRLASVLTPRERMGpIGAERLLARIRGEsVTPKMLD
  consensus/70%                    DGIFCTNDDLAVGAAFECQRLGLKIPDDMAIAGFHGHDIGQVMEPRLASVLTPRERMGpIGAERLLARIRGEsVTPKMLD

                   cov    pid  321         .] 331
1 Salmonella      100.0% 100.0%    LGFTLSPGGSI
2 Escherichia     100.0%  97.3%    LGFTLSPGGSI
3 Citrobacter     100.0%  98.2%    LGFTLSPGGSI
4 Klebsiella      100.0%  94.6%    LGFTLSPGGSI
5 Cronobacter     100.0%  91.5%    LGFTLSPGGSI
6 Enterobacter    100.0%  96.4%    LGFTLSPGGSI
7 Phytobacter     100.0%  93.7%    LGFTLSPGGSI
8 Kosakonia       100.0%  95.2%    LGFTLSPGGSI
9 Lelliottia      100.0%  95.2%    LGFTLSPGGSI
  consensus/100%                   LGFTLSPGGSI
  consensus/90%                    LGFTLSPGGSI
  consensus/80%                    LGFTLSPGGSI
  consensus/70%                    LGFTLSPGGSI
```

```
#  Percent Identity  Matrix - created by Clustal2.1
#
#

     1: Salmonella     100.00   97.28   98.19   94.56   91.54   96.37   93.66   95.17   95.17
     2: Escherichia     97.28  100.00   97.89   94.26   91.24   95.77   94.56   95.77   94.56
     3: Citrobacter     98.19   97.89  100.00   94.26   91.54   96.07   93.66   95.47   94.86
     4: Klebsiella      94.56   94.26   94.26  100.00   91.54   95.77   93.66   94.86   95.17
     5: Cronobacter     91.54   91.24   91.54   91.54  100.00   92.15   91.24   91.54   92.15
     6: Enterobacter    96.37   95.77   96.07   95.77   92.15  100.00   96.37   96.07   97.89
     7: Phytobacter     93.66   94.56   93.66   93.66   91.24   96.37  100.00   95.77   95.17
     8: Kosakonia       95.17   95.77   95.47   94.86   91.54   96.07   95.77  100.00   94.86
     9: Lelliottia      95.17   94.56   94.86   95.17   92.15   97.89   95.17   94.86  100.00
```

10. DeoR

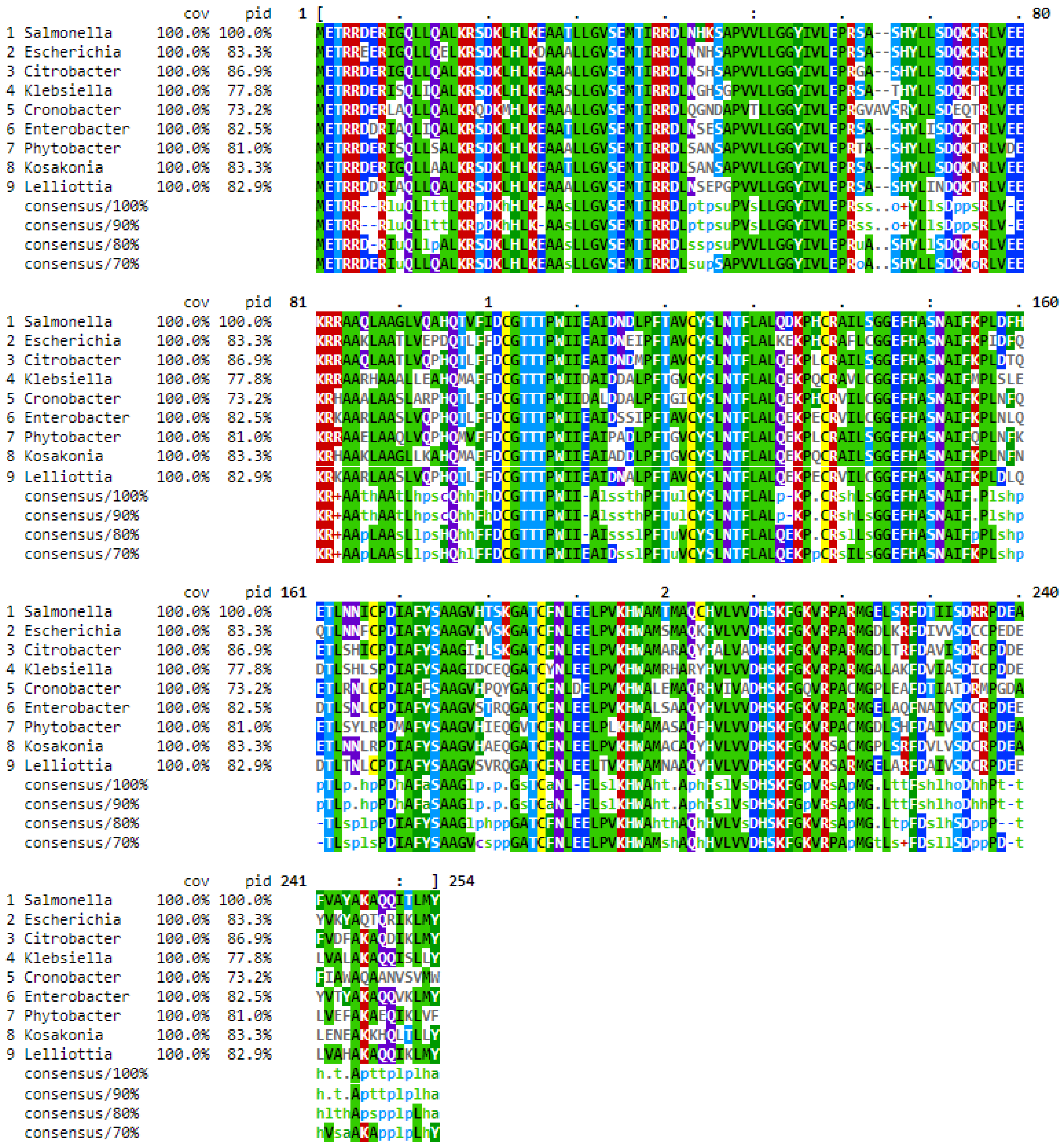

```
#  Percent Identity  Matrix - created by Clustal2.1
#
#
```

1: Salmonella	100.00	83.33	86.90	77.78	73.81	82.54	80.95	83.33	82.94
2: Escherichia	83.33	100.00	84.92	75.40	71.03	80.56	75.40	76.59	80.56
3: Citrobacter	86.90	84.92	100.00	78.57	73.02	81.75	80.56	78.17	82.54
4: Klebsiella	77.78	75.40	78.57	100.00	70.24	79.37	76.98	78.97	80.16
5: Cronobacter	73.81	71.03	73.02	70.24	100.00	73.41	71.03	72.22	73.02
6: Enterobacter	82.54	80.56	81.75	79.37	73.41	100.00	78.97	78.57	91.67
7: Phytobacter	80.95	75.40	80.56	76.98	71.03	78.97	100.00	84.13	78.97
8: Kosakonia	83.33	76.59	78.17	78.97	72.22	78.57	84.13	100.00	78.97
9: Lelliottia	82.94	80.56	82.54	80.16	73.02	91.67	78.97	78.97	100.00

11. TreR

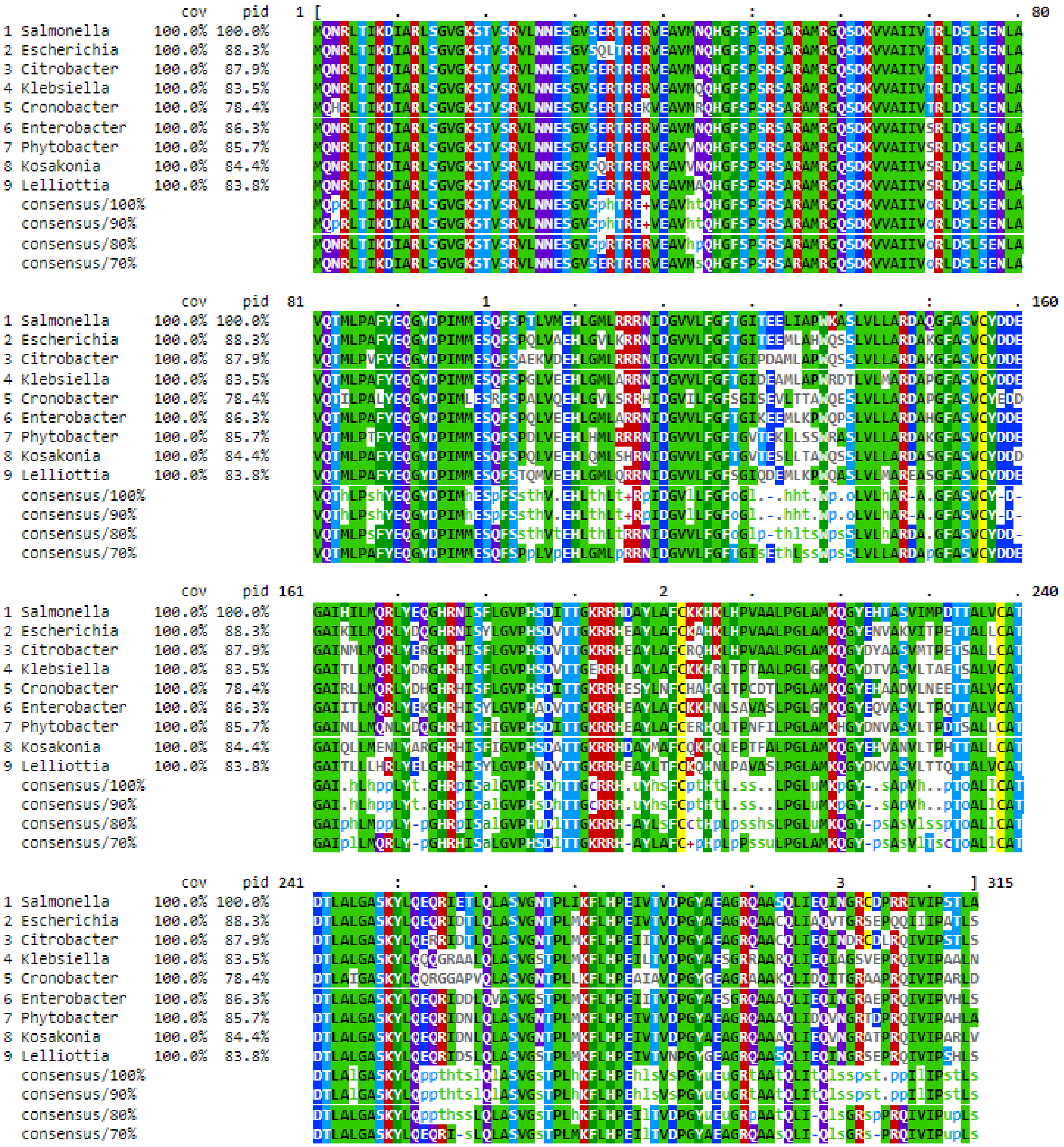

```
#   Percent Identity  Matrix - created by Clustal2.1
#
#
```

1: Salmonella	100.00	88.25	87.94	83.49	78.41	86.35	85.71	84.44	83.81
2: Escherichia	88.25	100.00	87.62	83.17	78.10	86.67	85.08	85.08	83.81
3: Citrobacter	87.94	87.62	100.00	84.13	77.14	86.03	85.08	82.86	84.76
4: Klebsiella	83.49	83.17	84.13	100.00	78.41	86.03	82.86	81.59	83.17
5: Cronobacter	78.41	78.10	77.14	78.41	100.00	77.14	77.14	78.10	75.56
6: Enterobacter	86.35	86.67	86.03	86.03	77.14	100.00	84.44	84.76	89.52
7: Phytobacter	85.71	85.08	85.08	82.86	77.14	84.44	100.00	88.57	82.22
8: Kosakonia	84.44	85.08	82.86	81.59	78.10	84.76	88.57	100.00	81.59
9: Lelliottia	83.81	83.81	84.76	83.17	75.56	89.52	82.22	81.59	100.00

12. IhfB

```
                  cov    pid  1 [         .         .         .         .         .         .         .         . 80
1 Salmonella     100.0% 100.0%   MTKSELIERLATQQSHIPAKAVEDAVKEMLEHMASTLAQGERIEIRGFGSFSLHYRAPRTGRNPKTGDKVELEGKYVPHF
2 Escherichia    100.0%  98.9%   MTKSELIERLATQQSHIPAKTVEDAVKEMLEHMASTLAQGERIEIRGFGSFSLHYRAPRTGRNPKTGDKVELEGKYVPHF
3 Citrobacter    100.0% 100.0%   MTKSELIERLATQQSHIPAKAVEDAVKEMLEHMASTLAQGERIEIRGFGSFSLHYRAPRTGRNPKTGDKVELEGKYVPHF
4 Klebsiella     100.0%  96.8%   MTKSELIERLASQQSHIPAKAVEDAVKEMLEHMASTLAQGERIEIRGFGSFSLHYRAPRTGRNPKTGDKVELEGKYVPHF
5 Cronobacter    100.0%  97.9%   MTKSELIERLASQHSHIPAKAVEDAVKEMLEHMASTLAQGERIEIRGFGSFSLHYRAPRTGRNPKTGDKVELEGKYVPHF
6 Enterobacter   100.0%  95.8%   MTKSELIERLASQQPHIPAKAVEDAVKEMLEHMATTLAQGERIEIRGFGSFSLHYRAPRTGRNPKTGDKVELEGKYVPHF
7 Phytobacter    100.0%  95.8%   MTKSELIERLASQQSHIPAKAVEDAVKEMLEHMASTLAQGERIEIRGFGSFSLHYRAPRTGRNPKTGDKVDLEGKYVPHF
8 Kosakonia      100.0%  96.8%   MTKSELIERLASQQSHIPAKAVEDAVKEMLEHMASTLAQGERIEIRGFGSFSLHYRAPRTGRNPKTGDKVDLEGKYVPHF
9 Lelliottia     100.0%  95.8%   MTKSELIERLASQQSHIPAKAVEDAVKEMLEHMASTLAQGERIEIRGFGSFSLHYRAPRTGRNPKTGDKVDLEGKYVPHF
  consensus/100%                 MTKSELIERLAoQpsHIPAKsVEDAVKEMLEHMAoTLAQGERIEIRGFGSFSLHYRAPRTGRNPKTGDKV-LEGKYVPHF
  consensus/90%                  MTKSELIERLAoQpsHIPAKsVEDAVKEMLEHMAoTLAQGERIEIRGFGSFSLHYRAPRTGRNPKTGDKV-LEGKYVPHF
  consensus/80%                  MTKSELIERLAoQQSHIPAKAVEDAVKEMLEHMASTLAQGERIEIRGFGSFSLHYRAPRTGRNPKTGDKV-LEGKYVPHF
  consensus/70%                  MTKSELIERLAoQQSHIPAKAVEDAVKEMLEHMASTLAQGERIEIRGFGSFSLHYRAPRTGRNPKTGDKV-LEGKYVPHF

                  cov    pid 81          .    ] 95
1 Salmonella     100.0% 100.0%   KPGKELRDRANIYG-
2 Escherichia    100.0%  98.9%   KPGKELRDRANIYG-
3 Citrobacter    100.0% 100.0%   KPGKELRDRANIYG-
4 Klebsiella     100.0%  96.8%   KPGKELRDRANIYEE
5 Cronobacter    100.0%  97.9%   KPGKELRDRANIYG-
6 Enterobacter   100.0%  95.8%   KPGKELRDRANIYGN
7 Phytobacter    100.0%  95.8%   KPGKELRDRANIYEG
8 Kosakonia      100.0%  96.8%   KPGKELRDRANIYE-
9 Lelliottia     100.0%  95.8%   KPGKELRDRANIYDN
  consensus/100%                 KPGKELRDRANIYt.
  consensus/90%                  KPGKELRDRANIYt.
  consensus/80%                  KPGKELRDRANIYt.
  consensus/70%                  KPGKELRDRANIYt.
```

```
#  Percent Identity  Matrix - created by Clustal2.1
#
#

     1: Salmonella     100.00   98.94  100.00   97.87   97.87   96.81   96.81   96.81   96.81
     2: Escherichia     98.94  100.00   98.94   96.81   96.81   95.74   95.74   95.74   95.74
     3: Citrobacter    100.00   98.94  100.00   97.87   97.87   96.81   96.81   96.81   96.81
     4: Klebsiella      97.87   96.81   97.87  100.00   97.87   95.79   97.89   98.94   96.84
     5: Cronobacter     97.87   96.81   97.87   97.87  100.00   96.81   96.81   96.81   96.81
     6: Enterobacter    96.81   95.74   96.81   95.79   96.81  100.00   94.74   95.74   95.79
     7: Phytobacter     96.81   95.74   96.81   97.89   96.81   94.74  100.00  100.00   97.89
     8: Kosakonia       96.81   95.74   96.81   98.94   96.81   95.74  100.00  100.00   98.94
     9: Lelliottia      96.81   95.74   96.81   96.84   96.81   95.79   97.89   98.94  100.00
```

13. RpoS

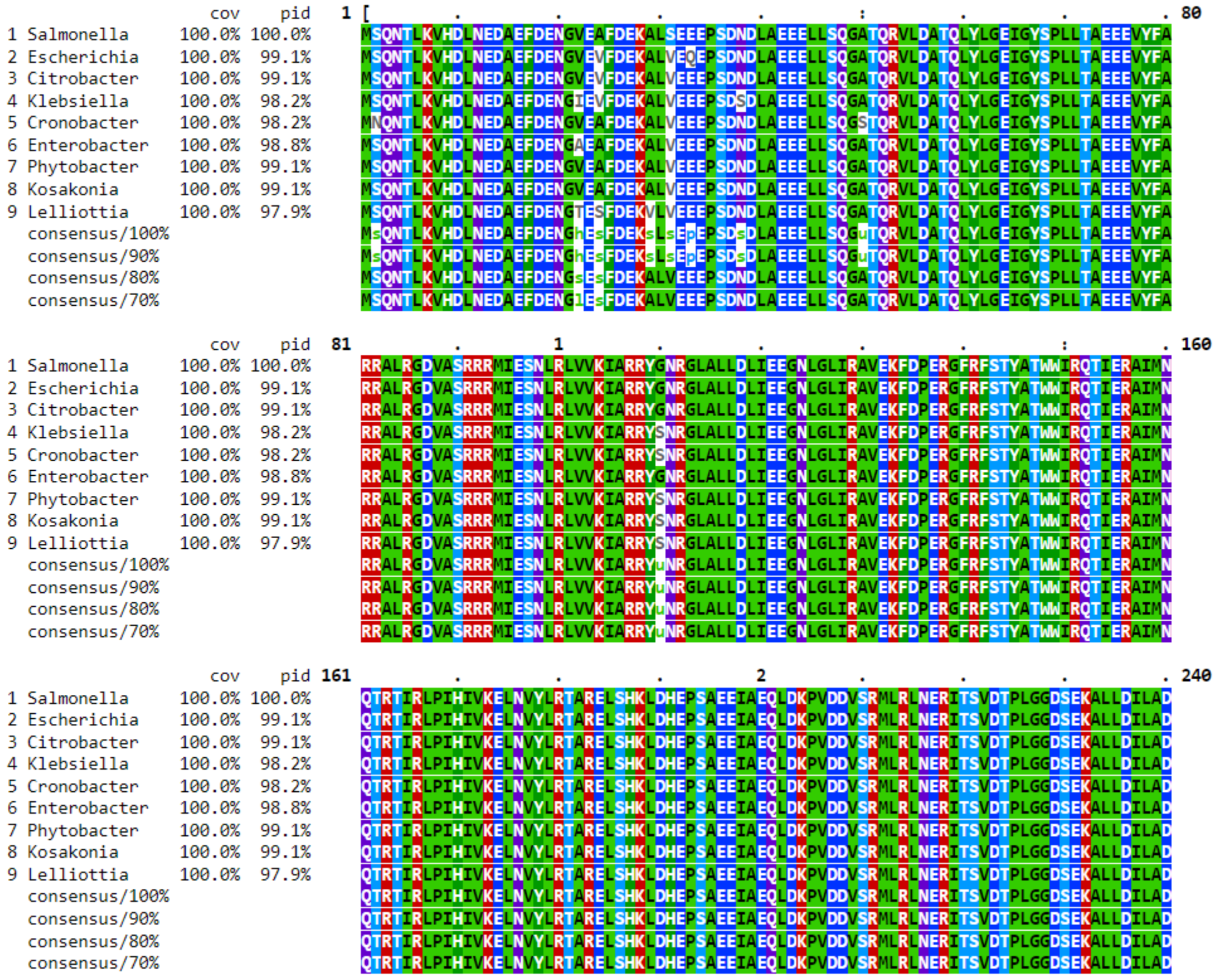

13. RpoS (part 2)

```
                      cov     pid  241         :         .         .         .         .         3         .         . 320
1 Salmonella       100.0% 100.0%      EKENGPEDTTQDDDMKQSIVKWLFELNAKQREVLARRFGLLGYEAATLEDVGREIGLTRERVRQIQVEGLRRLREILQTQ
2 Escherichia      100.0%  99.1%      EKENGPEDTTQDDDMKQSIVKWLFELNAKQREVLARRFGLLGYEAATLEDVGREIGLTRERVRQIQVEGLRRLREILQTQ
3 Citrobacter      100.0%  99.1%      EKDNGPEDTTQDDDMKQSIVKWLFELNAKQREVLARRFGLLGYEAATLEDVGREIGLTRERVRQIQVEGLRRLREILQTQ
4 Klebsiella       100.0%  98.2%      EKENGPEDTTQDDDMKQSIVKWLFELNAKQREVLARRFGLLGYEAATLEDVGREIGLTRERVRQIQVEGLRRLREILQGQ
5 Cronobacter      100.0%  98.2%      EKDNGPEDTTQDDDMKQSIVKWLFELNAKQREVLARRFGLLGYEAATLEDVGREIGLTRERVRQIQVEGLRRLREILQGQ
6 Enterobacter     100.0%  98.8%      EKDNGPEDTTQDDDMKQSIVKWLFELNAKQREVLARRFGLLGYEAATLEDVGREIGLTRERVRQIQVEGLRRLREILQGQ
7 Phytobacter      100.0%  99.1%      EKENGPEDTTQDDDMKQSIVKWLFELNAKQREVLARRFGLLGYEAATLEDVGREIGLTRERVRQIQVEGLRRLREILQAQ
8 Kosakonia        100.0%  99.1%      EKDNGPEDTTQDDDMKQSIVKWLFELNAKQREVLARRFGLLGYEAATLEDVGREIGLTRERVRQIQVEGLRRLREILQTQ
9 Lelliottia       100.0%  97.9%      EKDNGPEDTTQDDDMKQSIVKWLFELNAKQREVLARRFGLLGYEAATLEDVGREIGLTRERVRQIQVEGLRRLREILQGQ
  consensus/100%                      EK-NGPEDTTQDDDMKQSIVKWLFELNAKQREVLARRFGLLGYEAATLEDVGREIGLTRERVRQIQVEGLRRLREILQsQ
  consensus/90%                       EK-NGPEDTTQDDDMKQSIVKWLFELNAKQREVLARRFGLLGYEAATLEDVGREIGLTRERVRQIQVEGLRRLREILQsQ
  consensus/80%                       EK-NGPEDTTQDDDMKQSIVKWLFELNAKQREVLARRFGLLGYEAATLEDVGREIGLTRERVRQIQVEGLRRLREILQsQ
  consensus/70%                       EK-NGPEDTTQDDDMKQSIVKWLFELNAKQREVLARRFGLLGYEAATLEDVGREIGLTRERVRQIQVEGLRRLREILQsQ
```

```
                      cov     pid  321         ] 330
1 Salmonella       100.0% 100.0%      GLNIEALFRE
2 Escherichia      100.0%  99.1%      GLNIEALFRE
3 Citrobacter      100.0%  99.1%      GLNIEALFRE
4 Klebsiella       100.0%  98.2%      GLNIEALFRE
5 Cronobacter      100.0%  98.2%      GLNIEALFRE
6 Enterobacter     100.0%  98.8%      GLNIEALFRE
7 Phytobacter      100.0%  99.1%      GLNIEALFRE
8 Kosakonia        100.0%  99.1%      GLNIEALFRE
9 Lelliottia       100.0%  97.9%      GLNIEALFRE
  consensus/100%                      GLNIEALFRE
  consensus/90%                       GLNIEALFRE
  consensus/80%                       GLNIEALFRE
  consensus/70%                       GLNIEALFRE
```

Percent Identity Matrix - created by Clustal2.1
#
#

1: Salmonella	100.00	99.09	99.09	98.18	98.18	98.79	99.09	99.09	97.88
2: Escherichia	99.09	100.00	99.39	98.48	97.88	98.48	98.79	98.79	97.88
3: Citrobacter	99.09	99.39	100.00	98.48	98.48	99.09	98.79	99.39	98.48
4: Klebsiella	98.18	98.48	98.48	100.00	98.18	98.48	98.79	98.48	98.48
5: Cronobacter	98.18	97.88	98.48	98.18	100.00	98.79	98.79	99.09	98.48
6: Enterobacter	98.79	98.48	99.09	98.48	98.79	100.00	98.79	99.09	98.79
7: Phytobacter	99.09	98.79	98.79	98.79	98.79	98.79	100.00	99.39	98.48
8: Kosakonia	99.09	98.79	99.39	98.48	99.09	99.09	99.39	100.00	98.79
9: Lelliottia	97.88	97.88	98.48	98.48	98.48	98.79	98.48	98.79	100.00

14. ArgR

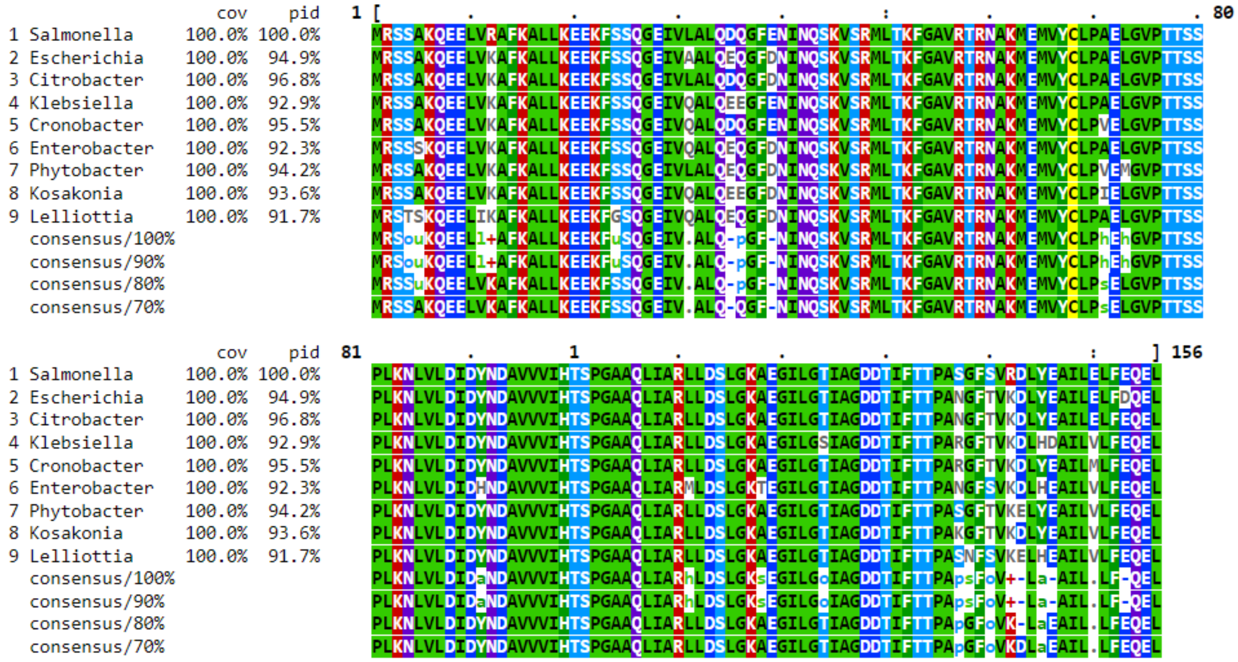


```
#  Percent Identity  Matrix - created by Clustal2.1
#
#
```

1: Salmonella	100.00	94.87	96.79	92.95	95.51	92.31	94.23	93.59	91.67
2: Escherichia	94.87	100.00	98.08	94.23	95.51	94.23	95.51	96.15	92.31
3: Citrobacter	96.79	98.08	100.00	94.23	96.79	94.23	96.15	96.15	92.31
4: Klebsiella	92.95	94.23	94.23	100.00	95.51	93.59	93.59	96.15	92.31
5: Cronobacter	95.51	95.51	96.79	95.51	100.00	92.95	95.51	96.15	91.67
6: Enterobacter	92.31	94.23	94.23	93.59	92.95	100.00	92.95	94.23	94.23
7: Phytobacter	94.23	95.51	96.15	93.59	95.51	92.95	100.00	96.15	93.59
8: Kosakonia	93.59	96.15	96.15	96.15	96.15	94.23	96.15	100.00	92.95
9: Lelliottia	91.67	92.31	92.31	92.31	91.67	94.23	93.59	92.95	100.00

15. GalR

```
                       cov    pid   1 [         .         .         .         .         :         .         .         . 80
1 Salmonella        100.0% 100.0%     MATIKDVARLAGVSVATVSRVINDSPKASEASRLAVTSAMESLSYHPNANARALAQQATETLGLVVGDVSDPFFGAMVKA
2 Escherichia       100.0%  90.4%     MATIKDVARLAGVSVATVSRVINNSPKASEASRLAVHSAMESLSYHPNANARALAQQTTETVGLVVGDVSDPFFGAMVKA
3 Citrobacter       100.0%  89.8%     MATIKDVARLAGVSVATVSRVINASPKASEASRLAVTSAMATLSYHPNANARALAQQSTETVGLVVGDVSDPFFGAMVKA
4 Klebsiella         98.8%  85.5%     MATIKDVARLAGVSVATVSRVINNSPKASEASRQSVGAAMETLNYHPNANARALAQQSTETVGLVVGDVSDPFFGAMVKA
5 Cronobacter        98.8%  85.8%     MPTIKDVARLAGVSVATVSRVINDSPKASDASRQAVLSAMEQLNYHPNANARALAQQTTETIGLVVGDVSDPFFGAMVKA
6 Enterobacter       98.8%  86.1%     MATIKDVARLAGVSVATVSRVINDSPKASDASRQAVQNAMESLNYHPNANARALAQQSTETIGLVVGDVSDPFFGAMVKA
7 Phytobacter        98.0%  88.1%     MATIKDVARLAGVSVATVSRVINDSPKASEASRLAVQTAMESLSYHPNANARALAQQSTETVGLVVGDVSDPFFGAMVKA
8 Kosakonia          97.7%  86.5%     MATIKDVARLAGVSVATVSRVINDSPKASESSRQAVQSAMDALNYHPNANARALAQQSTETVGLVVGDVSDPFFGAMVKA
9 Lelliottia         98.8%  84.0%     MATIKDVARMAGVSVATVSRVINHSPKASDTSRQAVMSAMETLNYHPNANARALAQQSTETVGLVVGDVSDPFFGAMVKA
  consensus/100%                      MsTIKDVARhAGVSVATVSRVINtSPKAS-sSR.uV.sAMttLsYHPNANARALAQQsTETlGLVVGDVSDPFFGAMVKA
  consensus/90%                       MsTIKDVARhAGVSVATVSRVINtSPKAS-sSR.uV.sAMttLsYHPNANARALAQQsTETlGLVVGDVSDPFFGAMVKA
  consensus/80%                       MATIKDVARLAGVSVATVSRVINsSPKAS-uSR.AV.sAM-sLsYHPNANARALAQQoTETlGLVVGDVSDPFFGAMVKA
  consensus/70%                       MATIKDVARLAGVSVATVSRVINsSPKAS-ASR.AVtoAMEoLsYHPNANARALAQQoTETlGLVVGDVSDPFFGAMVKA

                       cov    pid  81           .         1         .         .         .         .         :         . 160
1 Salmonella        100.0% 100.0%     VEQVAYHTGNFLLIGNGYHNEQKERQAIEQLIRHRCAALVVHAKMIPDADLASLMKQIPGMVLINRILPGLEHRCVALDD
2 Escherichia       100.0%  90.4%     VEQVAYHTGNFLLIGNGYHNEQKERQAIEQLIRHRCAALVVHAKMIPDADLASLMKQMPGMVLINRILPGFENRCIALDD
3 Citrobacter       100.0%  89.8%     VEQVAYHTGNFLLIGNGYHNEQKERQAIEQLIRHRCAALVVHAKMIPDADLTALMEQMPGMVLINRILPGFEQRCVALDD
4 Klebsiella         98.8%  85.5%     VEQVAYRTGNFLLIGNGYHNVQKERQAIEQLIRHRCAALVVHAKMIPDEELAGLMKQIPGMVLINRILPGYETRCVALDD
5 Cronobacter        98.8%  85.8%     VEQVAYHTGNFLLIGNGYHSEQKERQAIEQLIRHRCAALVVHAKTIPDEDLIPLMKQIPGMVLINRILPGFEARCVALDD
6 Enterobacter       98.8%  86.1%     VEQVSYQTGNFLLIGNGYHNEQKERQAIEQLIRHRCAALVVHAKMIPDAELIHLMKQIPGMVIINRIIPGFEKRCVALDD
7 Phytobacter        98.0%  88.1%     VEQVASSTGNFLLIGNGYHNEQKERNAIEQLIRHRCAALVVHAKMVPDAELIALMKQIPGMVLINRILPGFEQRCVSLDD
8 Kosakonia          97.7%  86.5%     VEQVASATGNFLLIGNGYHNEEKERKAIEQLIRHRCAALVVHAKTLPDDELASLMKQMPGMVLINRILPGFEQRCVALDD
9 Lelliottia         98.8%  84.0%     VEHVAYETGNFLLIGNGYHNEHKERQAIEQLIRHRCAALVVHAKVLPDEELLHLMKQIPGMVLINRIIPGYEKRCVALDD
  consensus/100%                      VEpVu.tTGNFLLIGNGYHs.pKERpAIEQLIRHRCAALVVHAKhlPDt-Lh.LMcQhPGMVlINRIlPGhEtRCluLDD
  consensus/90%                       VEpVu.tTGNFLLIGNGYHs.pKERpAIEQLIRHRCAALVVHAKhlPDt-Lh.LMcQhPGMVlINRIlPGhEtRCluLDD
  consensus/80%                       VEQVA.pTGNFLLIGNGYHNEpKERpAIEQLIRHRCAALVVHAKhlPDt-LhtLMKQhPGMVLINRIlPGaEpRCVALDD
  consensus/70%                       VEQVAYpTGNFLLIGNGYHNEQKERQAIEQLIRHRCAALVVHAKhlPDt-LhsLMKQhPGMVLINRILPGaEpRCVALDD

                       cov    pid 161           .         .         .         2         .         .         .         . 240
1 Salmonella        100.0% 100.0%     RYGAWLATRHLIQQGHTRIGYICSNHTISDAEDRLRGYYDALAESHIPANDRLVTFGEPDESGGEQAMTELLGRGRNFTA
2 Escherichia       100.0%  90.4%     RYGAWLATRHLIQQGHTRIGYLCSNHSISDAEDRLQGYYDALAESGIAANDRLVTFGEPDESGGEQAMTELLGRGRNFTA
3 Citrobacter       100.0%  89.8%     RYGAWLATRHLIQQGHTRIGYICSNHSISDAEDRLQGYYDALEESGIAVNDRLVTFGEPDESGGEQAMTELLGRGRNFTA
4 Klebsiella         98.8%  85.5%     RYGAWLATRHLIQQGHTRIGYLCSNHHISDAEDRLQGYYAALEESGLPCNDRLVAFAEPDESGGELAMTELLGRGRHFSA
5 Cronobacter        98.8%  85.8%     RYGAWLATRHLIQQGHTRIGYLCSNHAISDAEDRLQGYYDALREHDLPCNDRLVAWAEPDESGGEQAMTELLGQGKQFTA
6 Enterobacter       98.8%  86.1%     RYGAWLATRHLIQQGHTRIGYLCSNHPISDAEDRLQGYYDALRENGLPCNDRLVAYGEPDESGGEQAMTELLGRGRNFTA
7 Phytobacter        98.0%  88.1%     RYGAWLATRHLIQQGHTRIGYLCSNHAISDAEDRLQGYYDALKENGLPCNERLVTFGEPDESGGEQAMTELLGRGKNFTA
8 Kosakonia          97.7%  86.5%     RYGAWLATRHLIQQGHTKIGYLCSNHAISDAEDRLQGYYDALKENGLPCNDRLVTFAEPDESGGEQAMTELLGRGRNFTA
9 Lelliottia         98.8%  84.0%     RYGAWLATRHLIQQGHTRIGYLCSNHQISDAEDRLQGYHDALREHGLPSNERLVTYAEPDESGGEQAMTELLGRGKNFTA
  consensus/100%                      RYGAWLATRHLIQQGHT+IGYlCSNH.ISDAEDRLpGYasALtEptlssN-RLVsauEPDESGGE.AMTELLGpG+pFoA
  consensus/90%                       RYGAWLATRHLIQQGHT+IGYlCSNH.ISDAEDRLpGYasALtEptlssN-RLVsauEPDESGGE.AMTELLGpG+pFoA
  consensus/80%                       RYGAWLATRHLIQQGHTRIGYlCSNHtISDAEDRLQGYYDALtEpslssN-RLVsauEPDESGGEQAMTELLGRG+pFTA
  consensus/70%                       RYGAWLATRHLIQQGHTRIGYLCSNHsISDAEDRLQGYYDALcEsGlPsNDRLVsauEPDESGGEQAMTELLGRG+NFTA
```

15. GalR (part 2)

```
                       cov    pid 241         :         .         .         .         .         3         .         . 320
 1 Salmonella      100.0% 100.0%     VACYNDSMAAGAMGVLNDNGVGVPGEVSLIGFDDVLVSRYVRPRLTTIRYPIVTMATQAAELALALAGKCPTPEVTHVFS
 2 Escherichia     100.0%  90.4%     VACYNDSMAAGAMGVLNDNGIDVPGEISLIGFDDVLVSRYVRPRLTTVRYPIVTMATQAAELALALADNRPLPEITNVFS
 3 Citrobacter     100.0%  89.8%     VACYNDSMAAGAMGVLNDNGVEVPGEISLIGFDDVLVSRYVRPRLTTVRYPIVTMATQAAELALALAEKRPLPDITHVFS
 4 Klebsiella       98.8%  85.5%     VACYNDSMAAGAMGVLNDNGIDVPREISLIGFDDVLISRYVRPRLTTVRYPIVTMATQAAELALALAEYRPAPEITHLFS
 5 Cronobacter      98.8%  85.8%     VACYNDSMAAGAMGVLNDNGIAVPQEISLIGFDDVLVSRYVRPRLTTIRYPIVTMATQAAELALALADNLPPPEITNIFT
 6 Enterobacter     98.8%  86.1%     VASYNDSMAAGAMGVLNDNGIEVPGEISLIGFDDVLVSRYVRPRLTTVRYPIITMATQAAELALALAEQRQPPEITHLFS
 7 Phytobacter      98.0%  88.1%     IACYNDSMAAGAMGVLNDNGIAVPDEISLIGFDDVLISRYVRPRLTTVRYPIVTMATQAAELALALAENRPLPDVTHVFN
 8 Kosakonia        97.7%  86.5%     VACYNDSMAAGAMGVLNDNGIEVPREISLIGFDDVLVSRYVRPRLTTVRYPIVTMATQAAELALALAENRPLPEITHLFN
 9 Lelliottia       98.8%  84.0%     VACYNDSMAAGAMGVLNDNGIDVPAEISLIGFDDVLVSRYVRPRLTTVRYPIVTMATQAAELALALADNRPLPDITHLFS
   consensus/100%                    lAsYNDSMAAGAMGVLNDNGltVPtElSLIGFDDVLlSRYVRPRLTTlRYPIlTMATQAAELALALAt.h..P-lTplFs
   consensus/90%                     lAsYNDSMAAGAMGVLNDNGltVPtElSLIGFDDVLlSRYVRPRLTTlRYPIlTMATQAAELALALAt.h..P-lTplFs
   consensus/80%                     VACYNDSMAAGAMGVLNDNGltVPtEISLIGFDDVLlSRYVRPRLTTlRYPIVTMATQAAELALALA-ppP.P-lTplFs
   consensus/70%                     VACYNDSMAAGAMGVLNDNGItVPtEISLIGFDDVLVSRYVRPRLTTVRYPIVTMATQAAELALALA-pRPhP-ITHlFo

                       cov    pid 321         .         .  ] 343
 1 Salmonella      100.0% 100.0%     PTLVRRHSVSTPTDTGHLSTTD-
 2 Escherichia     100.0%  90.4%     PTLVRRHSVSTPSLEASHHATSD
 3 Citrobacter     100.0%  89.8%     PTLVRRHSVSSPAEPGPSASNE-
 4 Klebsiella       98.8%  85.5%     PTLVRRHSVVAPLEGDKS-----
 5 Cronobacter      98.8%  85.8%     PTLVRRHSVVAPAETDKS-----
 6 Enterobacter     98.8%  86.1%     PTLVRRHSVGAPAEAPDE-----
 7 Phytobacter      98.0%  88.1%     PTLVRRHSVVTPQDN--------
 8 Kosakonia        97.7%  86.5%     PTLVRRHSVIAPQE---------
 9 Lelliottia       98.8%  84.0%     PTLVRRHSVVAPPDAVSE-----
   consensus/100%                    PTLVRRHSV.sP...........
   consensus/90%                     PTLVRRHSV.sP...........
   consensus/80%                     PTLVRRHSVssP.-.........
   consensus/70%                     PTLVRRHSVssPt-ss.......
```

```
#  Percent Identity  Matrix - created by Clustal2.1
#
#
```

1: Salmonella	100.00	90.64	89.77	85.50	85.80	86.09	88.06	86.53	84.02
2: Escherichia	90.64	100.00	91.23	87.57	86.98	87.28	89.25	89.22	86.69
3: Citrobacter	89.77	91.23	100.00	87.57	85.50	87.57	89.25	89.22	85.80
4: Klebsiella	85.50	87.57	87.57	100.00	87.57	88.17	87.46	89.52	87.57
5: Cronobacter	85.80	86.98	85.50	87.57	100.00	88.17	86.57	88.02	86.98
6: Enterobacter	86.09	87.28	87.57	88.17	88.17	100.00	88.36	89.52	88.46
7: Phytobacter	88.06	89.25	89.25	87.46	86.57	88.36	100.00	91.02	87.76
8: Kosakonia	86.53	89.22	89.22	89.52	88.02	89.52	91.02	100.00	88.92
9: Lelliottia	84.02	86.69	85.80	87.57	86.98	88.46	87.76	88.92	100.00

Transport:

1. LysP

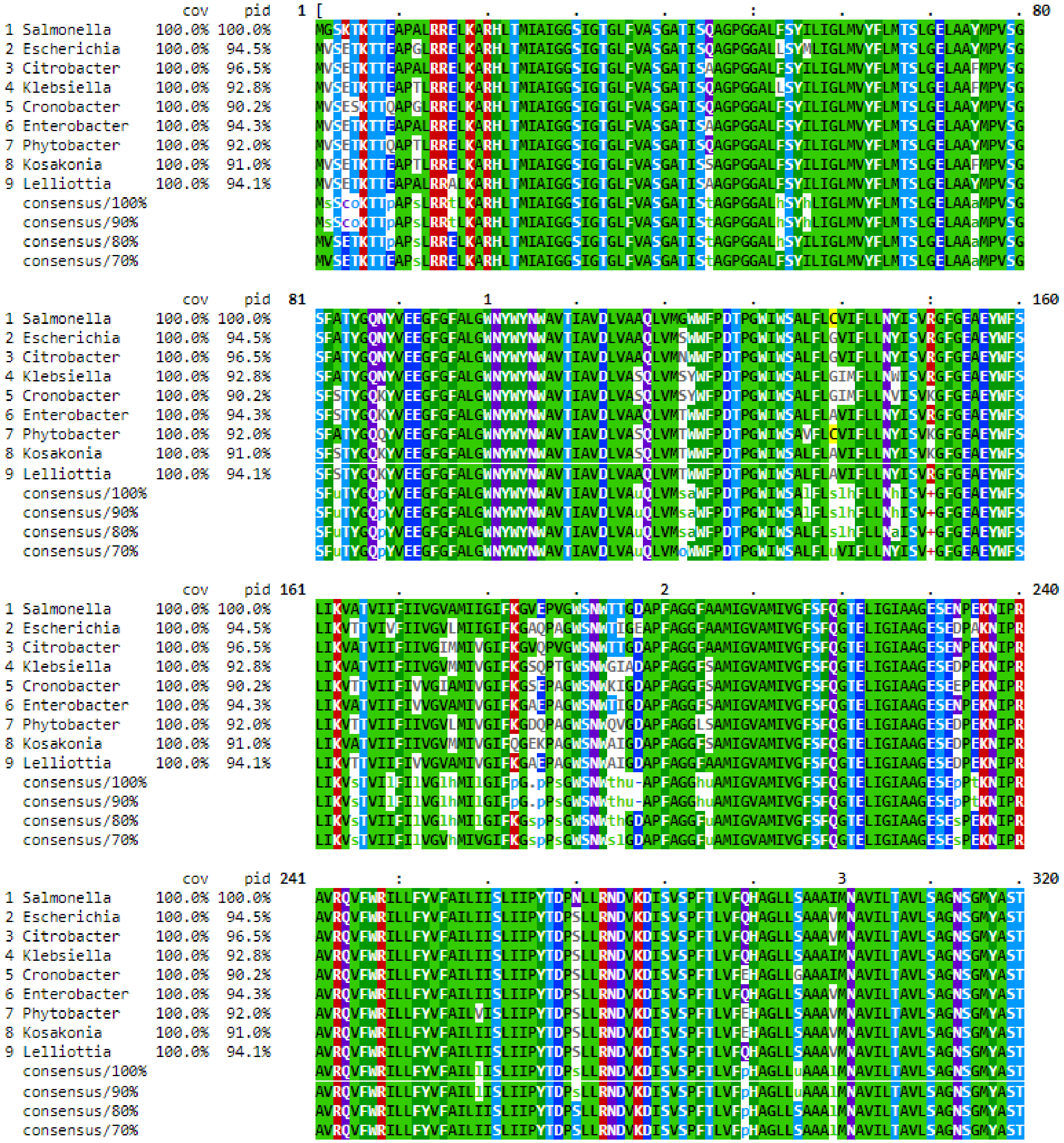

1. LysP (part 2)

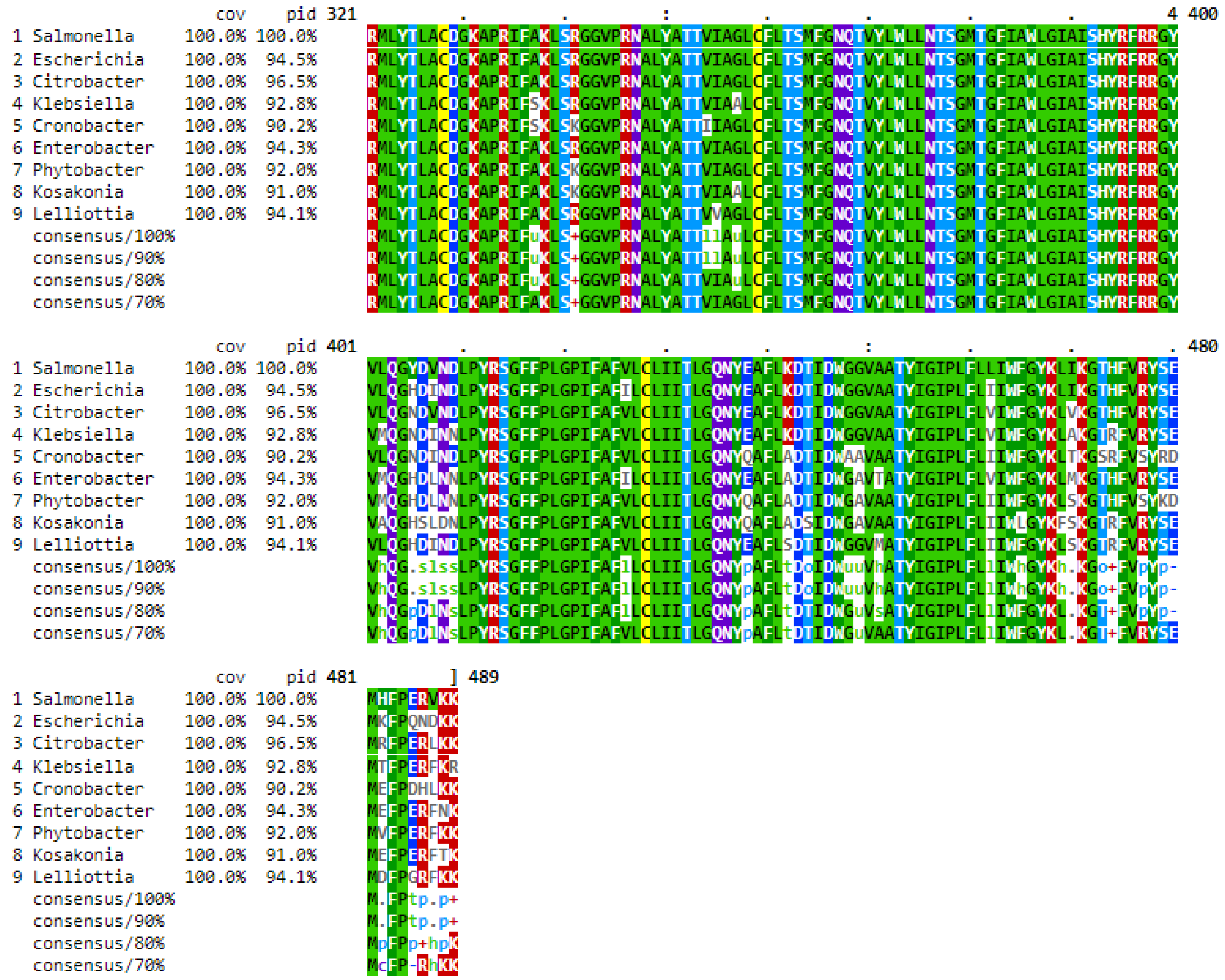

```
#  Percent Identity  Matrix - created by Clustal2.1
#
#
```

1: Salmonella	100.00	94.48	96.52	92.84	90.18	94.27	92.02	91.00	94.07
2: Escherichia	94.48	100.00	94.68	93.05	90.59	93.66	92.43	91.00	94.48
3: Citrobacter	96.52	94.68	100.00	94.27	90.80	94.89	92.43	92.23	94.48
4: Klebsiella	92.84	93.05	94.27	100.00	91.82	92.84	92.02	92.43	92.43
5: Cronobacter	90.18	90.59	90.80	91.82	100.00	91.41	92.23	91.00	91.82
6: Enterobacter	94.27	93.66	94.89	92.84	91.41	100.00	93.66	94.27	96.11
7: Phytobacter	92.02	92.43	92.43	92.02	92.23	93.66	100.00	94.07	92.84
8: Kosakonia	91.00	91.00	92.23	92.43	91.00	94.27	94.07	100.00	93.46
9: Lelliottia	94.07	94.48	94.48	92.43	91.82	96.11	92.84	93.46	100.00

2. BtuB

```
                    cov    pid    1 [         .         .         .         .         :         .         .         . 80
1 Salmonella       100.0% 100.0%    MIKKATLLTAFSVTAFSAWAQDTSPDTLVVTANRFQQPRSAVLAPVTIVTRQDIERWQSTSVNDVLRRLPGVDIAQSGGA
2 Escherichia      100.0%  87.3%    MIKKASLLTACSVTAFSAWAQDTSPDTLVVTANRFEQPRSTVLAPTTVVTRQDIDRWQSTSVNDVLRRLPGVDITQNGGS
3 Citrobacter       98.4%  56.3%    MIKKMSLLTALSVTAFSGWAQDATPDTLVVTANRFQQPVNTVLAPTDIVTRQDIERWQSRTVLDVMRRLPGVDVAQNGGM
4 Klebsiella        98.4%  54.8%    MIKKASLMTALSVTAFSGWAQDSNSDTLVVTANRFQQPVNTVLAPTDIVTRDDIDRWQSKDLNDVMRRLPGVDIARNGGM
5 Cronobacter       98.7%  52.5%    MIKKISLLTALSVTAFSGWAQDSGSNSLVVTANRFQQPVNTVLAPIDVVTREQIDKWQSKTVLDVLRRLPGVDISQNGGM
6 Enterobacter      99.2%  64.3%    MIKKVSLLTALSVTAFSGWAQDS-ADSLVVTANRFEQPAKTVLAPTSVVTREDIERWQAKSVVEIMSRLPGVDIAQSGGL
7 Phytobacter       70.0%  48.8%    MIKKASLLTALSVTAFSGWAQDGNPDTLVVTANRFQQPVNTVLAPTDVVTREDIQRWQSKDLNDVMRRLPGVDIAQYGGI
8 Kosakonia         98.7%  54.0%    MIKKASLLTALSVTAFSGWAQDSSSDNLVVTANRFQQPAKTVLAPTEVVTREDIDRWQSRSLVDVLRRLPGVDIAQNGGL
9 Lelliottia        98.2%  54.2%    MIKKVSLMTALSVTAFSGWAQDS-ADSLVVTANRFQQPVNTVLAPTDVVTREDIDRWQSRTVLDVMRRLPGVDIAQNGGM
  consensus/100%                    MIKKhoLhTAhSVTAFSuWAQDs.sssLVVTANRFpQPhpsVLAPhplVTRppIp+WQupsl.-lhpRLPGVDlsp.GG.
  consensus/90%                     MIKKhoLhTAhSVTAFSuWAQDs.sssLVVTANRFpQPhpsVLAPhplVTRppIp+WQupsl.-lhpRLPGVDlsp.GG.
  consensus/80%                     MIKKhSLhTAhSVTAFSuWAQDs.sDoLVVTANRFpQPhpTVLAPsslVTRpDI-RWQSpsl.DVhRRLPGVDIuQsGGh
  consensus/70%                     MIKKsSLLTALSVTAFSGWAQDossDoLVVTANRFQQPssTVLAPTslVTRpDI-RWQS+ol.DVhRRLPGVDIAQsGGh

                    cov    pid   81         .         1         .         .         .         .         :         . 160
1 Salmonella       100.0% 100.0%    GQNSSIFIRGTNSSHVLVLIDGVRLNLAGVSGSADLSQFPVSLVQRIEYIRGPRSAIYGSDAIGGVVNIITTRDNPGTEL
2 Escherichia      100.0%  87.3%    GQLSSIFIRGTNASHVLVLIDGVRLNLAGVSGSADLSQFPIALVQRVEYIRGPRSAVYGSDAIGGVVNIITTRDEPGTEI
3 Citrobacter       98.4%  56.3%    GQSSSLFVRGTEAKHVLVLVDGIPVARPGITNNSDIDQIPVSLVQRIEYIRGPRSAIYGSGAIGGVVNIITMAGEERSQI
4 Klebsiella        98.4%  54.8%    GQSASLYVRGTEARHVLVLIDGVPMARPGISNGVDISQIPISLVQRVEYIRGPRSAVYGSGAIGGVVNIITMTDSDRSQV
5 Cronobacter       98.7%  52.5%    GQLASVYVRGTDSRHALILMDGVPVSRPNLSNDSNLYQIPISLIQRIEFIRGPRSAVYGSGAIGGVINIITQSDDEVSRI
6 Enterobacter      99.2%  64.3%    GATSSTFIRGTESRHVLVLIDGVPLNSAGISNVPDLSQIPTSLIQRIEYVRGPRSALYGSDAIGGVINIITGRDKPGAEI
7 Phytobacter       70.0%  48.8%    GQSSSLFVRGTEARHVLVLIDGVPMARAGIGNAIDIGQIPVSLVQRIEYIRGPRSAVYGSGAIGGVVNIITMSDDERAQI
8 Kosakonia         98.7%  54.0%    GQTASVYVRGTEAKHLLLLIDGVPVARSGISNDPELNQIPLSLVQRIEYIRGPRSAVYGSGAIGGVVNVITMTGNEKSQI
9 Lelliottia        98.2%  54.2%    GQSSSLFVRGTEAKHVLVLVDGIPVARPGISNSSDIDQIPVSLVQRIEYIRGPRSAIYGSGAIGGVVNIITMTGEERSQI
  consensus/100%                    Gt.uShalRGTpupHhLlLhDGl.hs.sslsss.pl.QhPhuLlQRlEalRGPRSAlYGSsAIGGVlNlIT.tsp.hspl
  consensus/90%                     Gt.uShalRGTpupHhLlLhDGl.hs.sslsss.pl.QhPhuLlQRlEalRGPRSAlYGSsAIGGVlNlIT.tsp.hspl
  consensus/80%                     GQ.uSlalRGTpupHlLlLlDGl.hshsGlosss-lsQhPlSLlQRlEYIRGPRSAlYGSsAIGGVlNIIThpsp.tspl
  consensus/70%                     GQsuSlalRGT-u+HVLVLlDGVPlspsGlSNssDlsQIPlSLVQRIEYIRGPRSAlYGSsAIGGVVNIIThpsp.topI

                    cov    pid  161         .         .         .         2         .         .         .         . 240
1 Salmonella       100.0% 100.0%    TAGWGSNSYQNYDISTQQQLGENTRATLIGDYEYTKGFDVVAKGGTGMQAQPDRDGFLSKTLYGALEHTFSDRWSGFVRG
2 Escherichia      100.0%  87.3%    SAGWGSNSYQNYDVSTQQQLGDKTRVTLLGDYAHTHGYDVVAYGNTGTQAQTDNDGFLSKTLYGALEHNFTDAWSGFVRG
3 Citrobacter       98.4%  56.3%    NAGVGSKGYQQYDGAWRQRFGD-TVVTAAGAYQTTKGFDVQPRS--SYSGDGDRDGYRNKLFWGSVEHKFNDNFDGYFRG
4 Klebsiella        98.4%  54.8%    NVGMGSNGYQTYDGAINKRFGD-TVVTAAGAYQTTKGFNVQPNS--PYSGDSDRDGYRNKLFLGGVQHKFDDNFSGFFRG
5 Cronobacter       98.7%  52.5%    DAGVGSKGYQEYSGTLRQRFGD-TVVTAMGAYQTTHGFNVQPGS--TWRHDNDRDGYRNKTFFGSLQHHFSDNVDGFFRG
6 Enterobacter      99.2%  64.3%    SAGVGSKGYQTYDGAFQQVL-NKTKITMAGNYTYTRGFDIAAQD---APRQPDRDGFMSKSLYGSVEQQITDSVSGFFRG
7 Phytobacter       70.0%  48.8%    NAGMGTNGYQNYDGAINQRFGD-TMVTAAGAYQTTKGFNVQPDS--TYADDSDRDGYRNKLFWGGVQHKFNDSVSGFFRG
8 Kosakonia         98.7%  54.0%    NAGVGSKGYQTYDGTLRQRFGD-TVATAAGSYTSTRGFNVQPGS--TWDHDEDRDGYRNKTFWGSLQHKFNDNFDGFFRG
9 Lelliottia        98.2%  54.2%    NAGVGSKGYQEYDGTYRKRFDD-TVVTAAGAYQTTKGFNVQPHS--TYSGDNDRDGYRNKLFWGGIEHKFNDYFDGSFRG
  consensus/100%                    ssGhGopuYQpYshshpp.h.p.ThhThhGsYt.T+Gasl.s.s...h.tp.DpDGahsK.hhGulppphsD.hsG.hRG
  consensus/90%                     ssGhGopuYQpYshshpp.h.p.ThhThhGsYt.T+Gasl.s.s...h.tp.DpDGahsK.hhGulppphsD.hsG.hRG
  consensus/80%                     sAGhGSpuYQpYDsshppphs-.ThsThhGsYphT+GFsV.stu..shttpsDRDGahsKhhaGulpHpFsDthsGahRG
  consensus/70%                     sAGhGSpGYQpYDGshpQphGD.ThlThhGsYppT+GFsVtstu..shptpsDRDGahsKhhaGulpHpFsDshsGFFRG

                    cov    pid  241         :         .         .         .         .         3         .         . 320
1 Salmonella       100.0% 100.0%    YGYDNRTDYDAYYSPGSP-----LIDTRKLYSQSWDAGLHFNGERIQSQLVSSYSHSKDYNYDPHYGRYDTSATLDEMKQ
2 Escherichia      100.0%  87.3%    YGYDNRTNYDAYYSPGSP-----LLDTRKLYSQSWDAGLRYNGELIKSQLITSYSHSKDYNYDPHYGRYDSSATLDEMKQ
3 Citrobacter       98.4%  56.3%    YGYASNADYDQGN---WGYDG--GNDEHQNYTQSWDTGLRFNSGIYSSQLVANYQRIKDYNYSSLNGRYATGYSLDDMEQ
4 Klebsiella        98.4%  54.8%    YGYTANTDYDQGS---YGYVG--GNDERQNYTQSWDTGLRYSSGIYSSQLIANYQRIKDYNYSSDAGRYAAGATLDDMEQ
5 Cronobacter       98.7%  52.5%    YGYANNADYDVGS---PPFSPAYSADENQYDNQSWDTGLRYNADIYSSQLIASFQKLKSYNYSSLYGRYQDGTTLDRMEQ
6 Enterobacter      99.2%  64.3%    FGYDNRTAYDGYDHYDANFMVDGRPDTRQLYSQNWDTGLRYSQGIFQSQLVAGYGRSKDQNYDPKKGRYADSATMDDVKQ
7 Phytobacter       70.0%  48.8%    YGYSSNTDYDQGS---GGYAG--GGDESQNYTQSWDTGLNYHSGIYSSQLIANYQHLKNYNYNAQDGRYAASASLDNMEQ
8 Kosakonia         98.7%  54.0%    YNFSNNVDYDLGS---APFSADYSADERQLFVQGWDTGLNFAQGIYSSQLLASYQKSKDYNYSSIYGRYNDGTTLDDMEQ
9 Lelliottia        98.2%  54.2%    YGYASNADYDQGN---YGYDG--GNDEHQNYTQSWDAAMRFHSGIYSSQLVANYQRIKDYNYSSQTWRYAPGYSLDDMEQ
  consensus/100%                    asasspssYD.h.....s.......Dppp..sQsWDsuhpattthhpSQLlssat+.Ks.NYss..hRYtsuhohDphcQ
  consensus/90%                     asasspssYD.h.....s.......Dppp..sQsWDsuhpattthhpSQLlssat+.Ks.NYss..hRYtsuhohDphcQ
  consensus/80%                     YGYsspssYD.h.....s.......Dppp.asQuWDsGLpapttlhpSQLlusYp+.KsYNYss..GRYssuhoLDcMcQ
  consensus/70%                     YGYsspsDYDths....sa.s..tsDp+Q.YoQSWDsGL+asusIapSQLlAsYp+.KDYNYss.hGRYssusoLD-McQ
```

2. btuB (part 2)

```
                       cov    pid 321        .         .         :         .         .         .         .         4 400
1 Salmonella        100.0% 100.0%     YNVQWTNSVVVGHGNVGAGVDWQKQTTTPGTGYVPEGYDQRNTGVYLTGLQQ-LGDFTLEAAARSDDNSQFGRHGTWQTS
2 Escherichia       100.0%  87.3%     YTVQWANNVIVGHGSIGAGVDWQKQTTTPGTGYVEDGYDQRNTGIYLTGLQQ-VGDFTFEGAARSDDNSQFGRHGTWQTS
3 Citrobacter        98.4%  56.3%     RYIQWGNNVVVGHGAISAGVDWKQEKLMSFGEYGTDNYKRDNTGLYLTGQQQ-IDSVTLEASGREDHDEQFGWHGTWQTA
4 Klebsiella         98.4%  54.8%     RYIQWGNNIEVGHGAISGGVDWKQEKLTSSSTTLSDAYKRDTTGLYLTGQQQ-IDSVTLEASGREDHDEQFGWHGTWQTA
5 Cronobacter        98.7%  52.5%     RYIQWGNNLIVGHGSVSAGVDWKQEKLTSATQADTDRYKRDTTGIYLTGQQQVLEHVTLEASGREDHDEQFGWHGTWQTA
6 Enterobacter       99.2%  64.3%     YTTQWLNTIEVGHGNIGTGLDWQKQKTQAGTGYLEKGYEQRNTGVFASAMQQ-FNSVTLEAAARNDDNSNFGNHGTWQTS
7 Phytobacter        70.0%  48.8%     RYIQWGNNVEVGHGSVSGGVDWKQEKLQSSGTSSTDIYKRDTTGLYLTGQQQ-IDKVTLEASGREDHDEQFGWHGTWQTA
8 Kosakonia          98.7%  54.0%     RYIQWGNNVNVGKGSVGAGVDWKQERLVSSNSTTRDAYERENTGLYLNGMQQ-FGDVTLEASGREDKDDEFGWHGTWQTA
9 Lelliottia         98.2%  54.2%     RYIQWGNNVIVGHGAISGGVDWKQEKLMSLGTTSSDHYKRDNTGLYLTGQQQ-IDSVTLEASGREDHDEQFGWHGTWQTA
  consensus/100%                      h.hQWhNsl.VG+GslusGlDWpppph.s.st...c.YcpcsTGlahsu.QQ.htphThEuuuRpDcsppFG.HGTWQTu
  consensus/90%                       h.hQWhNsl.VG+GslusGlDWpppph.s.st...c.YcpcsTGlahsu.QQ.htphThEuuuRpDcsppFG.HGTWQTu
  consensus/80%                       hhlQWsNsl.VGHGsluuGVDWpppph.s.sth.p-tYcpcsTGlYLoG.QQ.hsphTLEAuuRpDcsppFGhHGTWQTu
  consensus/70%                       hhlQWuNNl.VGHGuluuGVDWppp+hhuusshspDtYcpcsTGlYLTG.QQ.lssVTLEAuuRpDcspQFGhHGTWQTu

                       cov    pid 401        .         .         .         .         :         .         .         . 480
1 Salmonella        100.0% 100.0%     AGWEFIEGYRFIASYGTSYKAPNLGQLY-----------GYYGNPNLNPEKSKQWEGAFEGLTAGVSWRISGYRNDINDM
2 Escherichia       100.0%  87.3%     AGWEFIEGYRFIASYGTSYKAPNLGQLY-----------GFYGNPNLDPEKSKQWEGAFEGLTAGVNWRISGYRNDVSDL
3 Citrobacter        98.4%  56.3%     AGWQFVDDYKVTLSYGTGFLAPSLGQQFGAERF------GIASNPNLKPEESKQWEAGLEGLTGPVDWRLSTYRYKIQNL
4 Klebsiella         98.4%  54.8%     AGWEFIDGYRATLSYGTGFLAPSLGQQYGATRFASFYGPGIASNPNLKPEESKQWEAGIDGLTGPLDWRISAYHYEIQNL
5 Cronobacter        98.7%  52.5%     AGWEFVDGYRATLSYGTGFLAPGLTQQFGSPQY------GIASNPNLQPEKSRQWEVGIEGITGPLDWRLSTWRYKIDNL
6 Enterobacter       99.2%  64.3%     AAWEFVDGYRVIASYGTAFKAPTMSQIHS----------ASYGNPDLKPEESKQWEGGFEGLTGPVNWRITGYRNDIDNL
7 Phytobacter        70.0%  48.8%     AGWEFVDDYRVTLLRDRIPGAIAWAAVWRGAVWHCF-QPE------F--------ETGRVEAMGSRA------------
8 Kosakonia          98.7%  54.0%     AGWEFVENYRLTVSYGTGFLAPSLGQQFGAKRF------GIASNPNLKPEESRQWEAGVEGLTGPLDWRLSAYRYEIENL
9 Lelliottia         98.2%  54.2%     AGWEFVEDYKVTLSYGTGFLAPSLGQQFGAERF------GISSNPNLKPEESKQWEAGLEGLTGPVDWRLSTYRYEIQNL
  consensus/100%                      AuWpFl-sY+hhh.hsp..hA.shst.a...........t......h........Esuh.thhushs.............
  consensus/90%                       AuWpFl-sY+hhh.hsp..hA.shst.a...........t......h........Esuh.thhushs.............
  consensus/80%                       AGWEFl-sY+hhhSYGTuahAPshuQ.a...........u..uNPsLpPEcS+QWEsuh-GlTuslsWRlosa+.clpsh
  consensus/70%                       AGWEFl-sYRhhhSYGTuahAPsLuQ.at..........GhhuNPNLpPEcS+QWEuGhEGLTGslsWRlSsYR.cIpsL

                       cov    pid 481        .         5         .         .         .         .         :         . 560
1 Salmonella        100.0% 100.0%     IDYDDHLQKYYNEGKARIKGIEATANFDTGPLTHTVSYDYVDARNAITDTPLPRRSKQMAKYQLDWDVYDFDWGMTYQYL
2 Escherichia       100.0%  87.3%     IDYDDHTLKYYNEGKARIKGVEATANFDTGPLTHTVSYDYVDARNAITDTPLLRRAKQQVKYQLDWQLYDFDWGITYQYL
3 Citrobacter        98.4%  56.3%     IDYNDN--TYFNVKSATIKGLEWTGNLTTGPVDHRLTLQYVDPRDDETDQQLYRRAKQQVKYELSGEVYELGWDVTYQYI
4 Klebsiella         98.4%  54.8%     IDYKNN--QYINVKSATIKGLEWTGNVTTGPVEHHLTLQYVDPRDDDTGKVLYRRAKQQVKYELTGQIYDLDWNVMYQYL
5 Cronobacter        98.7%  52.5%     ISYDND--AYYNVKSATIKGVEWTGNLTTGPVSHQLTLQYIDPRDDETHERLQRRARQQVKYNLSGEVSDFGWDITYQYI
6 Enterobacter       99.2%  64.3%     ISSDPHTYRYYNVDEARIKGVEATAQFETGPVGHQISYDYVDPRNAKTHEVLARRSKQQVKYQLDWQVWDLDWNLAYRYL
7 Phytobacter        70.0%  48.8%     --------------------------------------------------------------------------------
8 Kosakonia          98.7%  54.0%     ITYSDT--AYYNINSATIKGVEWTGSVDTGIFSHRVTLQYIDPRDDETNEVLPRRAKRQAKYQLDWSVLNVDMDLAWEYF
9 Lelliottia         98.2%  54.2%     IDYNNN--AYFNVKSATIKGLEWTANVTTGPVDHRLTLQYVDPRDDETGKQLYRRAKQQVKYDLSGQAYSLGWDVTWQYI
  consensus/100%                      ................................................................................
  consensus/90%                       ................................................................................
  consensus/80%                       Is.psp..tYhN.tpApIKGlEhTuphpTG.htHplohpYlDsRss.Ttp.L.RRu+p.sKYpLshph.phshshhapYh
  consensus/70%                       IsYssp..tYaN.tpApIKGlEhTushsTGPlsHplohpYlDsRss.Ttp.L.RRuKQQsKYpLshplhshsYslsapYl

                       cov    pid 561        .         .         .         6         .         .         .  ] 633
1 Salmonella        100.0% 100.0%     GSRYDSDYS--AYPYRTVKMGGVSLWDLTVAYPVTSHLTVRGKIANLFDKDYETVYGYQTAGREYTLSGSYTF
2 Escherichia       100.0%  87.3%     GTRYDKDYS--SYPYQTVKMGGVSLWDLAVAYPVTSHLTVRGKIANLFDKDYETVYGYQTAGREYTLSGSYTF
3 Citrobacter        98.4%  56.3%     GERYDYDYD--N--SRRVKMGGLSVWDIGLSYPVTSHLTVRGKIANLFDKDYETVYGYQTAGREYTLSGSYTF
4 Klebsiella         98.4%  54.8%     GKRYDDDYD--N--GRDVKMGGLSLWDVGLSYPVTSHLTVRGKIANLFDKDYETVYGYQSAGREYTLSGSYTF
5 Cronobacter        98.7%  52.5%     GEREDTNFN--TFPSEKVKLGSVSLWDVAVSYPVTSHLTVRGKIANLFDKDYETAYGYATAGREYNLSGSYTF
6 Enterobacter       99.2%  64.3%     GTRYDVAIDPDTYASERVKMGGVSLWDVAVSYPVTSHLTVRGKIANLFDKDYETVYGYQTAGREYTLSGSYTF
7 Phytobacter        70.0%  48.8%     -------------------------------------------------------------------------
8 Kosakonia          98.7%  54.0%     GKRYDNRTS--TYNPEQRILPSYSMVDFSASYPVTSHLTVRGKIANLFDKEYETAYGYQTAGREYTLSGSYTF
9 Lelliottia         98.2%  54.2%     GERYDYDYD--N--ARRVQMGGVSLWDVALSYPVTSHLTVRGKIANLFDKDYETVYGYQTAGREYTLSGSYTF
  consensus/100%                      .........................................................................
  consensus/90%                       .........................................................................
  consensus/80%                       GpR.D.ths..s...pph.hsuhShhDhshuYPVTSHLTVRGKIANLFDK-YETsYGYtoAGREYsLSGSYTF
  consensus/70%                       GpRYD.shs..s...cpVphGulSlWDluluYPVTSHLTVRGKIANLFDKDYETsYGYQTAGREYTLSGSYTF
```

```
#  Percent Identity  Matrix - created by Clustal2.1
#
#

 1: Salmonella     100.00   87.30   57.95   56.95   54.13   65.68   52.56   55.61   55.89
 2: Escherichia     87.30  100.00   57.28   58.61   56.27   66.34   53.02   55.61   56.88
 3: Citrobacter     57.95   57.28  100.00   81.21   72.22   57.85   74.43   69.93   89.69
 4: Klebsiella      56.95   58.61   81.21  100.00   70.59   58.84   77.43   69.77   82.16
 5: Cronobacter     54.13   56.27   72.22   70.59  100.00   55.67   65.98   70.78   71.85
 6: Enterobacter    65.68   66.34   57.85   58.84   55.67  100.00   53.36   57.31   57.52
 7: Phytobacter     52.56   53.02   74.43   77.43   65.98   53.36  100.00   65.30   74.37
 8: Kosakonia       55.61   55.61   69.93   69.77   70.78   57.31   65.30  100.00   70.54
 9: Lelliottia      55.89   56.88   89.69   82.16   71.85   57.52   74.37   70.54  100.00
```

3. GlnH

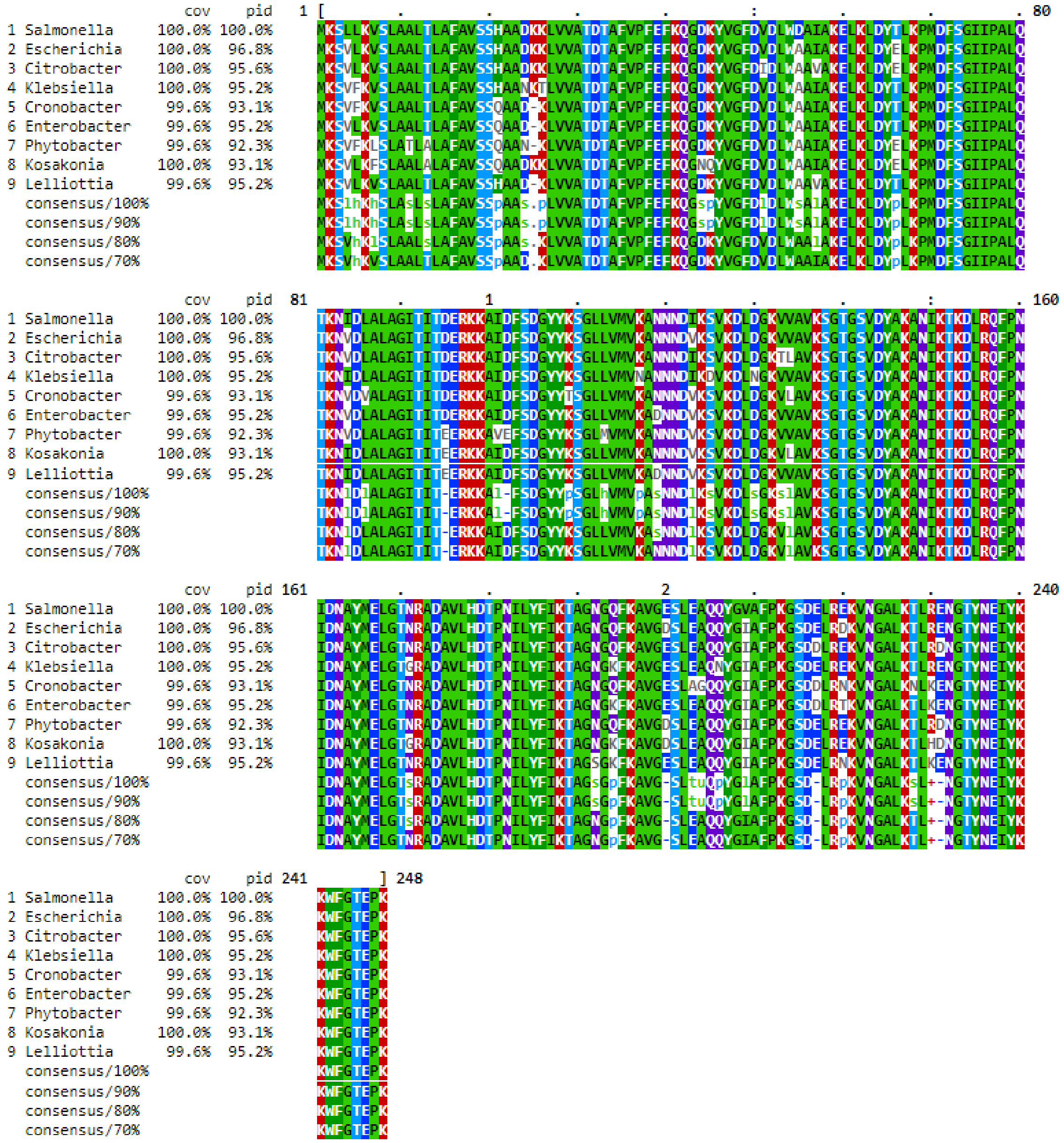

Percent Identity Matrix - created by Clustal2.1
#
#

1: Salmonella	100.00	96.77	95.56	95.16	93.52	95.55	92.71	93.15	95.55
2: Escherichia	96.77	100.00	96.37	94.35	94.74	96.76	95.14	94.76	95.95
3: Citrobacter	95.56	96.37	100.00	93.15	93.93	95.14	93.12	93.55	94.33
4: Klebsiella	95.16	94.35	93.15	100.00	91.90	93.93	91.90	92.34	93.93
5: Cronobacter	93.52	94.74	93.93	91.90	100.00	95.95	91.90	91.90	93.93
6: Enterobacter	95.55	96.76	95.14	93.93	95.95	100.00	93.12	93.93	97.17
7: Phytobacter	92.71	95.14	93.12	91.90	91.90	93.12	100.00	94.33	92.31
8: Kosakonia	93.15	94.76	93.55	92.34	91.90	93.93	94.33	100.00	93.93
9: Lelliottia	95.55	95.95	94.33	93.93	93.93	97.17	92.31	93.93	100.00

4. ModC

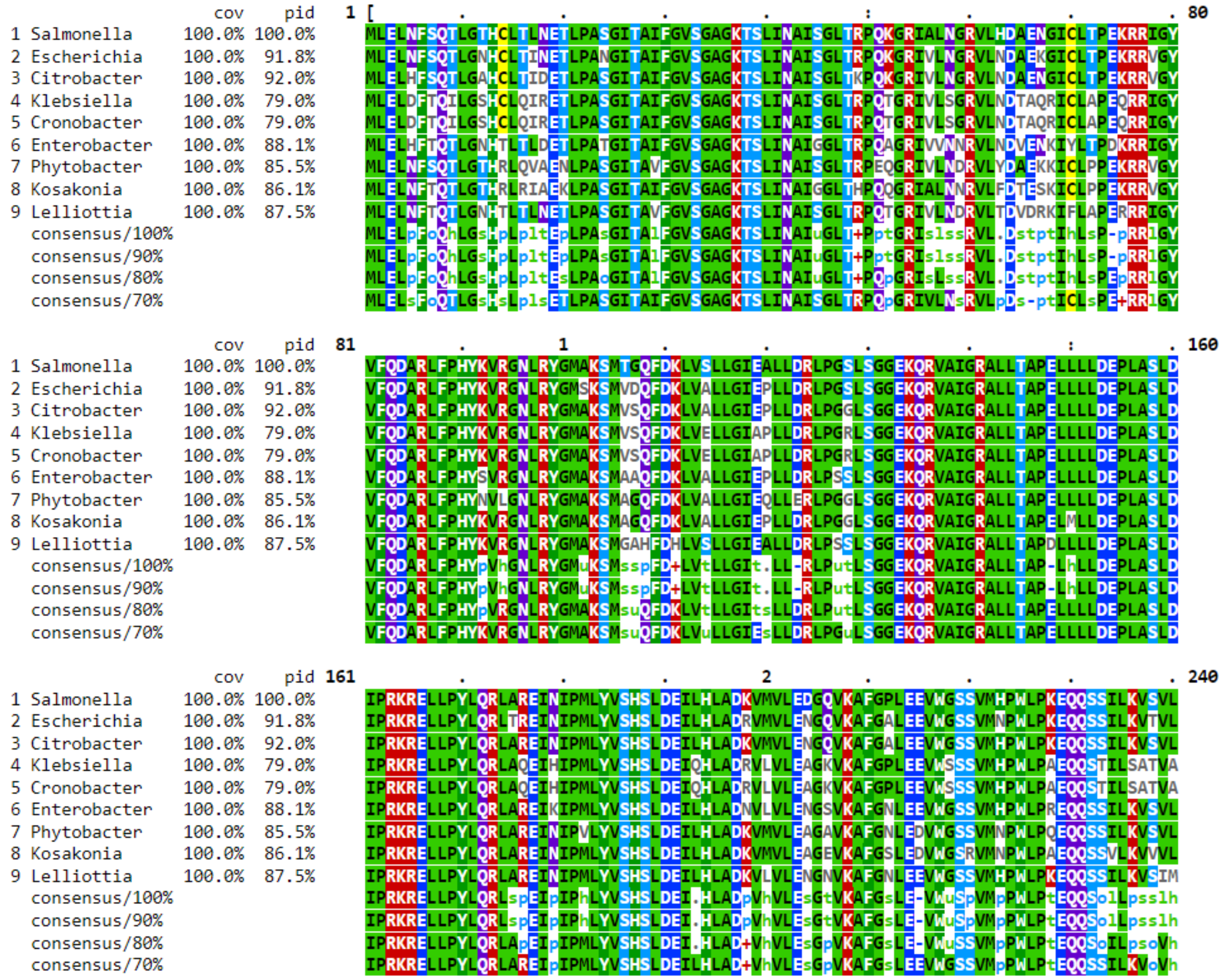
cov pid 1 80
1 Salmonella 100.0% 100.0%
2 Escherichia 100.0% 91.8%
3 Citrobacter 100.0% 92.0%
4 Klebsiella 100.0% 79.0%
5 Cronobacter 100.0% 79.0%
6 Enterobacter 100.0% 88.1%
7 Phytobacter 100.0% 85.5%
8 Kosakonia 100.0% 86.1%
9 Lelliottia 100.0% 87.5%
consensus/100%
consensus/90%
consensus/80%
consensus/70%
cov pid 81 160
cov pid 161 240

4. ModC (part 2)

```
                          cov     pid 241         :         .         .         .         .         3         .         . 320
1 Salmonella           100.0% 100.0%  EHHPHYAMTALALGDQHLWVNKLNQPLQSTLRIRIQASDVSLVLQPPQQTSIRNVLRAKVANCYDDNGQVEVQLEIGGRT
2 Escherichia          100.0%  91.8%  EHHPHYAMTALALGDQHLWVNKLDEPLQAALRIRIQASDVSLVLQPPQQTSIRNVLRAKVVNSYDDNGQVEVELEVGGKT
3 Citrobacter          100.0%  92.0%  EHHPHYAMTALALGDQHVWVNKLDEPLHTALRIRIQASDVSLVLQPPQQSSIRNVLRAKVANCYDVDGQVEVQLEVGGKT
4 Klebsiella           100.0%  79.0%  AQHPQYAMTALTLGDQLLWVNRLERPAGDTARIRIQASDVSLTLAQPSGTSIRNILRAEVAQCLEVNGQIEVQLRVSGRL
5 Cronobacter          100.0%  79.0%  AQHPQYAMTALTLGDQLLWVNRLERPAGDTARIRIQASDVSLTLAQPSGTSIRNILRAEVAQCLEVNGQIEVQLRVSGRL
6 Enterobacter         100.0%  88.1%  EHHPHYAMTALALGDQHLWVNKIDTPLQSALRIRIQASDVSLVLQPPLQTSIRNILRAKVAECFDDNGQVEVQLEVGSRT
7 Phytobacter          100.0%  85.5%  EQHPHYAMTALALGDQHIWVNRLDKPLQAAVRIRIQATDVSLVLQPSGNTSIRNTLRAKVVQNYDLDGQVEVQLDVSGRT
8 Kosakonia            100.0%  86.1%  EHHPHYAMTALALGDQHIWVNKVDLPLQSVLRIRIQATDVSLVLQPPMNTSIRNILRAKVAQCFDIDGQVEVQLEVGSRT
9 Lelliottia           100.0%  87.5%  EHHPHYAMTALALGDQHLWVNRIDKPLQTALRIRIQASDVSLVLQPPLQTSIRNILRAKVAQCFDDNGQVEVQLDVGSKT
  consensus/100%                      tpHPpYAMTALsLGDQhlWVN+lp.PhtsshRIRIQAoDVSLsLt.s.toSIRNhLRAcVspsh-.sGQlEVpLcluu+h
  consensus/90%                       tpHPpYAMTALsLGDQhlWVN+lp.PhtsshRIRIQAoDVSLsLt.s.toSIRNhLRAcVspsh-.sGQlEVpLcluu+h
  consensus/80%                       tpHPpYAMTALsLGDQhlWVN+l-pPhtsshRIRIQAoDVSLsLt.P.tTSIRNlLRAcVspsh-.sGQlEVQLcVuu+h
  consensus/70%                       EpHPHYAMTALALGDQHlWVN+l-pPLpsslRIRIQASDVSLVLQPP.pTSIRNlLRAKVApCaDssGQVEVQL-Vuu+T

                          cov     pid 321         .         .         : ] 352
1 Salmonella           100.0% 100.0%  LWARISPWARDELNIKPGLWLYAQVKSVSITA
2 Escherichia          100.0%  91.8%  LWARISPWARDELAIKPGLWLYAQIKSVSITA
3 Citrobacter          100.0%  92.0%  LWARISPWARDELTIKPGLWLYAQIKSVSITA
4 Klebsiella           100.0%  79.0%  LWARISPWARDDLAIAPGQQVFAQIKSVSIAA
5 Cronobacter          100.0%  79.0%  LWARISPWARDDLAIAPGQQVFAQIKSVSIAA
6 Enterobacter         100.0%  88.1%  LWARISPWARDELGIKPGLWLYAQIKSVSITT
7 Phytobacter          100.0%  85.5%  LWARISPWARDELNIKPGLWLYAQIKSVSITA
8 Kosakonia            100.0%  86.1%  LWARISPWARDELAVKPGQWLYAQIKSVSITA
9 Lelliottia           100.0%  87.5%  LWARISPWARDELGIKPGLWLYAQIKSVSITT
  consensus/100%                      LWARISPWARD-LsltPG..laAQlKSVSIss
  consensus/90%                       LWARISPWARD-LsltPG..laAQlKSVSIss
  consensus/80%                       LWARISPWARD-LsItPG..laAQIKSVSIss
  consensus/70%                       LWARISPWARDELsIKPG.WLYAQIKSVSITA
```

Percent Identity Matrix - created by Clustal2.1
#
#

1: Salmonella	100.00	91.76	92.05	78.98	78.98	88.07	85.51	86.08	87.50
2: Escherichia	91.76	100.00	93.47	78.69	78.69	87.50	85.80	85.23	86.08
3: Citrobacter	92.05	93.47	100.00	79.26	79.26	88.07	85.80	86.36	86.08
4: Klebsiella	78.98	78.69	79.26	100.00	100.00	77.27	75.85	76.99	77.56
5: Cronobacter	78.98	78.69	79.26	100.00	100.00	77.27	75.85	76.99	77.56
6: Enterobacter	88.07	87.50	88.07	77.27	77.27	100.00	82.67	85.80	90.34
7: Phytobacter	85.51	85.80	85.80	75.85	75.85	82.67	100.00	87.22	83.52
8: Kosakonia	86.08	85.23	86.36	76.99	76.99	85.80	87.22	100.00	83.24
9: Lelliottia	87.50	86.08	86.08	77.56	77.56	90.34	83.52	83.24	100.00

5. ZitB (ybgR in *Salmonella*)

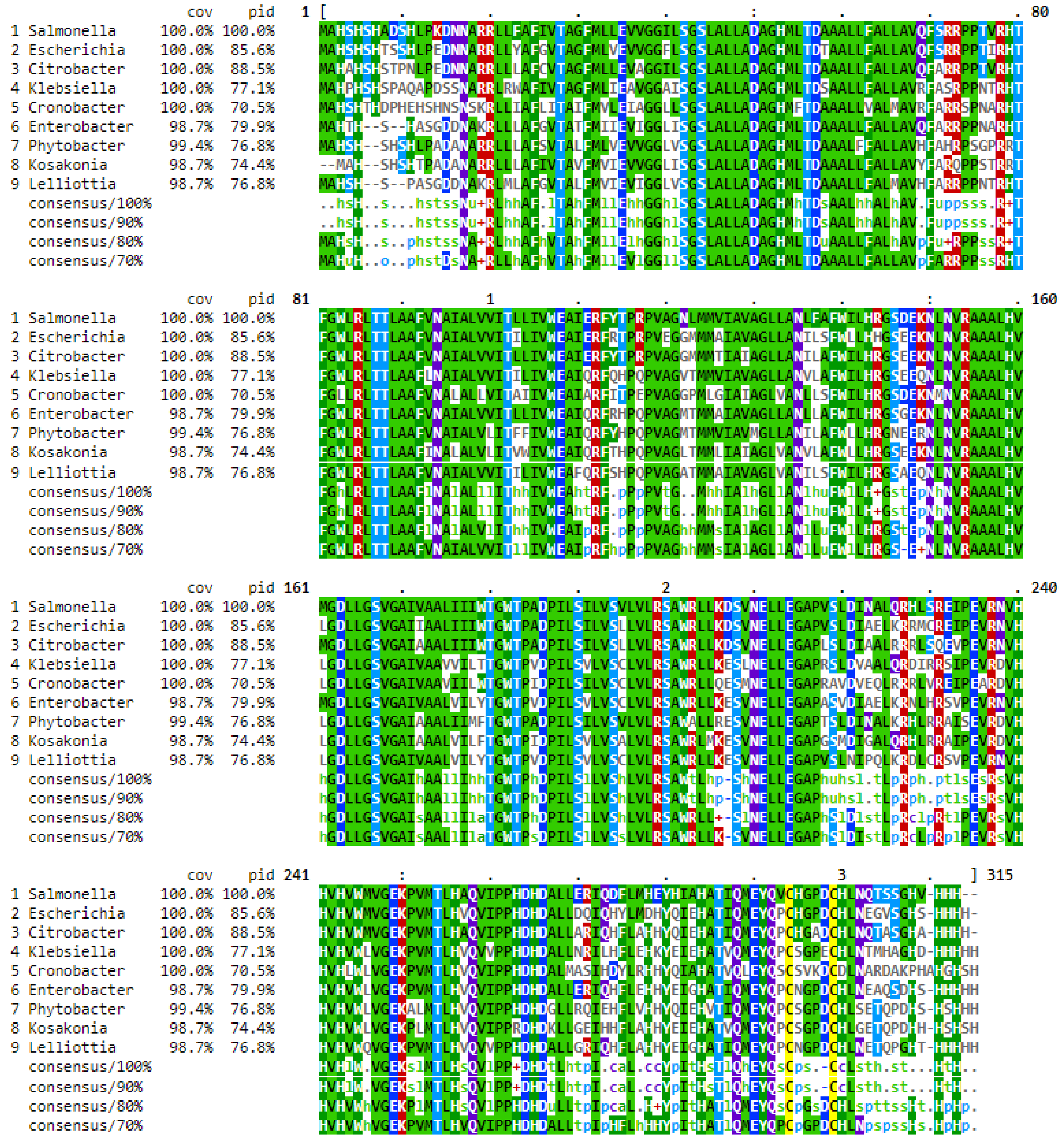

```
#  Percent Identity  Matrix - created by Clustal2.1
#
#

     1: Salmonella    100.00   85.90   88.78   77.56   71.15   81.49   77.74   75.32   78.25
     2: Escherichia    85.90  100.00   86.90   76.68   69.97   81.23   78.46   74.43   79.61
     3: Citrobacter    88.78   86.90  100.00   77.32   71.57   82.20   77.81   76.38   79.61
     4: Klebsiella     77.56   76.68   77.32  100.00   70.70   82.26   75.96   78.71   81.94
     5: Cronobacter    71.15   69.97   71.57   70.70  100.00   72.26   66.35   69.68   71.61
     6: Enterobacter   81.49   81.23   82.20   82.26   72.26  100.00   79.03   80.19   89.03
     7: Phytobacter    77.74   78.46   77.81   75.96   66.35   79.03  100.00   82.58   78.06
     8: Kosakonia      75.32   74.43   76.38   78.71   69.68   80.19   82.58  100.00   79.22
     9: Lelliottia     78.25   79.61   79.61   81.94   71.61   89.03   78.06   79.22  100.00
```

Cell envelope/cell surface related:

1. OmpA

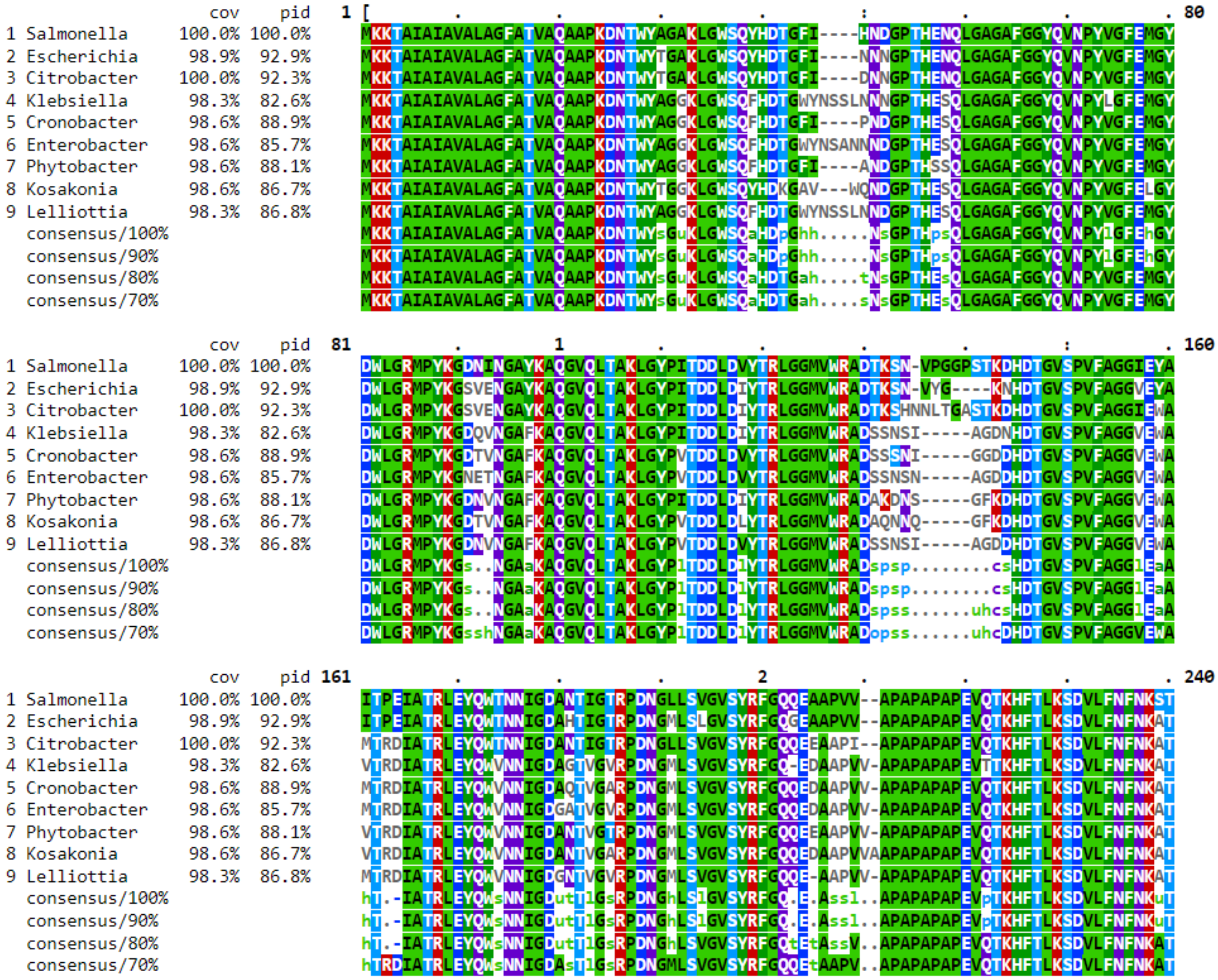

1. OmpA (part 2)

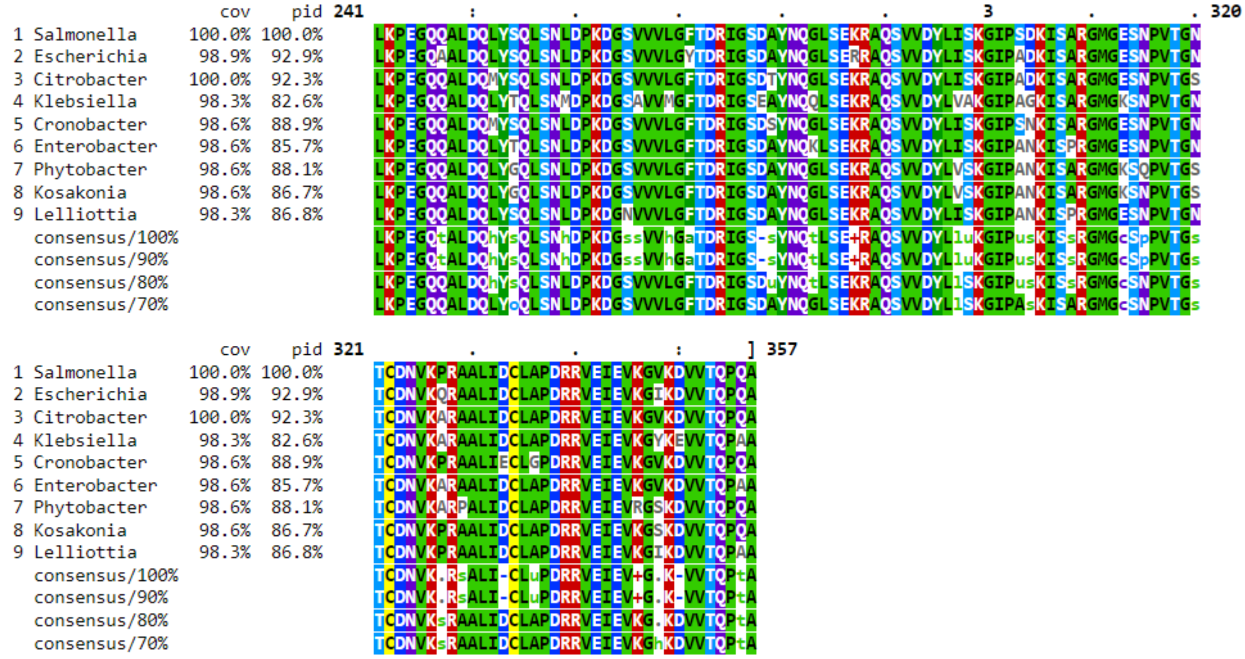


```
#  Percent Identity  Matrix - created by Clustal2.1
#
#
```

1: Salmonella	100.00	93.93	92.57	85.47	90.72	88.41	89.86	88.99	89.83
2: Escherichia	93.93	100.00	92.20	86.55	88.34	87.46	88.63	88.05	88.30
3: Citrobacter	92.57	92.20	100.00	86.38	90.17	89.02	90.46	89.31	88.99
4: Klebsiella	85.47	86.55	86.38	100.00	89.88	92.29	89.88	89.34	92.84
5: Cronobacter	90.72	88.34	90.17	89.88	100.00	93.37	91.64	92.51	94.51
6: Enterobacter	88.41	87.46	89.02	92.29	93.37	100.00	90.49	90.80	96.57
7: Phytobacter	89.86	88.63	90.46	89.88	91.64	90.49	100.00	94.24	91.62
8: Kosakonia	88.99	88.05	89.31	89.34	92.51	90.80	94.24	100.00	91.93
9: Lelliottia	89.83	88.30	88.99	92.84	94.51	96.57	91.62	91.93	100.00

2. ExbB

```
                         cov    pid   1 [        .         .         .         .         :         .         .         . 80
1 Salmonella          100.0% 100.0%    MGNNLMQTDLSVWGMYQHADIVVKCVMIGLILASVVTWAIFFSKSVEFFTQKRRLKREQLQLADARSLDQASDIAAGFSA
2 Escherichia         100.0%  89.8%    MGNNLMQTDLSVWGMYQHADIVVKCVMIGLILASVVTWAIFFSKSVEFFNQKRRLKREQQLLAEARSLNQANDIAADFGS
3 Citrobacter         100.0%  91.8%    MGNNLMQTDLSVWGMYQHADIVVKCVMIGLILASVVTWAIFFSKSVDFFSQRRRLKREQQQLAEARSLDQASDIAASFGA
4 Klebsiella           99.6%  88.1%    MGNNLMQADLSVWGMYHHADIVVKVVMIGLILASVVTWAIFFGKGAEILASKRRLKREQQQLAEARSLDQASDIASAFEA
5 Cronobacter         100.0%  86.9%    MGNNLMQTDLSVWGMYQHADIVVKIVMIGLILASVVTWAIFFSKSVELSGHKRRLKREQQELAGARTLDDAREMAERFGP
6 Enterobacter         99.6%  88.9%    MGNNLMQTDLSVWGMYHHADIVVKIVMIGLILASVITWAIFFGKSAELISQKRRLKREQQQLAEARSLDQASDMTSSFHA
7 Phytobacter         100.0%  90.6%    MGNNLMQADLSVWGMYQHADIVVKIVMIGLILASVVTWAIFFSKSAEMIAHKRRLKREQQLLADARSLDQASDIASAFHA
8 Kosakonia            99.6%  90.2%    MGNNLMQTDLSVWGMYQHADIVVKVVMIGLILASVVTWALFFSKSVEMISHKRRLKREQQQLADARTLDQASEIASAFHA
9 Lelliottia           99.6%  88.9%    MGNNLMQTDLSVWGMYHHADIVVKIVMIGLILASVITWAIFFSKSVELISQKRRLKREQQQLAEARSLDQASDMTSSFHA
  consensus/100%                       MGNNLMQsDLSVWGMYpHADIVVKhVMIGLILASVlTWAlFFuKus-h.sp+RRLKREQ..LAtARoLspAp-hsttFts
  consensus/90%                        MGNNLMQsDLSVWGMYpHADIVVKhVMIGLILASVlTWAlFFuKus-h.sp+RRLKREQ..LAtARoLspAp-hsttFts
  consensus/80%                        MGNNLMQsDLSVWGMYpHADIVVKhVMIGLILASVlTWAIFFuKSsEhhspKRRLKREQQ.LA-ARoLDQAs-hsusFtu
  consensus/70%                        MGNNLMQTDLSVWGMYpHADIVVKhVMIGLILASVVTWAIFFSKSsEhhupKRRLKREQQpLA-ARSLDQASDhAuuFtA

                         cov    pid  81          .         1         .         .         .         .         :         . 160
1 Salmonella          100.0% 100.0%    KSLSAQLINEAQNELELSQGSEDNEGIKERTGFRLERRVAAVGRYMGRGNGYLATIGAISPFVGLFGTVWGIMNSFIGIA
2 Escherichia         100.0%  89.8%    KSLSLHLLNEAQNELELSEGSDDNEGIKERTSFRLERRVAAVGRQMGRGNGYLATIGAISPFVGLFGTVWGIMNSFIGIA
3 Citrobacter         100.0%  91.8%    KSLNTQLLNEAQNELELSAGSEDNEGIKERTGFRLERRVAAAGRQMGRGNGYLATIGAISPFVGLFGTVWGIMNSFIGIA
4 Klebsiella           99.6%  88.1%    KSLTTQLINEAQNELELSAGAEDNEGIKERTGFRLERRVAAVGRHMGRGNGYLATIGAISPFVGLFGTVWGIMNSFIGIA
5 Cronobacter         100.0%  86.9%    KSLSARLIEEAQNERELSAGCEDNEGIKERTGFRLERRVAAAGRHMGRGNGYLATIGAISPFVGLFGTVWGIMNSFIGIA
6 Enterobacter         99.6%  88.9%    KSLTTLLVNEAQNELELSAGSEDNEGIKERTGFRLERRVAAVGRHMGRGNGYLATIGAISPFVGLFGTVWGIMNSFIGIA
7 Phytobacter         100.0%  90.6%    RSLSTQLINEAQNELELSAGSEDNEGIKERTGFRLERRVAATGRHMGRGNGYLATIGAISPFVGLFGTVWGIMNSFIGIA
8 Kosakonia            99.6%  90.2%    RSLSTMLINEAQNELELSAGSEDNEGIKERTGFRLERRVAATGRHMGRGNGYLATIGAISPFIGLFGTVWGIMNSFIGIA
9 Lelliottia           99.6%  88.9%    KSLTTLLVNEAQNELELSAGSEDNEGIKERTGFRLERRVAAIGRHMGRGNGYLATIGAISPFVGLFGTVWGIMNSFIGIA
  consensus/100%                       +SLsh.LlpEAQNEhELStGs-DNEGIKERTuFRLERRVAAhGR.MGRGNGYLATIGAISPFlGLFGTVWGIMNSFIGIA
  consensus/90%                        +SLsh.LlpEAQNEhELStGs-DNEGIKERTuFRLERRVAAhGR.MGRGNGYLATIGAISPFlGLFGTVWGIMNSFIGIA
  consensus/80%                        +SLos.LlNEAQNELELStGuEDNEGIKERTGFRLERRVAAsGRpMGRGNGYLATIGAISPFVGLFGTVWGIMNSFIGIA
  consensus/70%                        KSLos.LlNEAQNELELSAGSEDNEGIKERTGFRLERRVAAsGRaMGRGNGYLATIGAISPFVGLFGTVWGIMNSFIGIA

                         cov    pid 161          .         .         .         2         .         .         .         . 240
1 Salmonella          100.0% 100.0%    QTQTTNLAVVAPGIAEALLATAIGLVAAIPAVVIYNIFARQIGSYKATLGDVAAQVLLLQSRDLDLNASASAQPVRAAQK
2 Escherichia         100.0%  89.8%    QTQTTNLAVVAPGIAEALLATAIGLVAAIPAVVIYNVFARQIGGFKAMLGDVAAQVLLLQSRDLDLEASAAAHPVRVAQK
3 Citrobacter         100.0%  91.8%    QTQTTNLAVVAPGIAEALLATAIGLVAAIPAVVIYNIFARQIGGLKATLSDIAAQVLLLQSRDLDLDASASAHPVRTAQK
4 Klebsiella           99.6%  88.1%    QTQTTNLAVVAPGIAEALLATAIGLFAAIPAVVIYNIFARMIGSYKASLGDVAAQVLLLQSRDLDLSASG-VKPVRSAQK
5 Cronobacter         100.0%  86.9%    QSQTTNLAVVAPGIAEALLATAIGLIAAIPAVVIYNIFARTIGGYKATLGDVAAQVLLLQSRDLDLEASAHAQPVRTAQK
6 Enterobacter         99.6%  88.9%    QTQTTNLAVVAPGIAEALLATAIGLVAAIPAVVIYNVFARMIGSYKATLGDVAAQVLLLQSRDLDLNASA-AKPVHAASK
7 Phytobacter         100.0%  90.6%    QTQTTNLAVVAPGIAEALLATAIGLVAAIPAVVIYNIFARMIGGYKATLGDVAAQVLLLQSRDLDLAASAAAKPVHAAQK
8 Kosakonia            99.6%  90.2%    QTQTTNLAVVAPGIAEALLATAIGLVAAIPAVVIYNIFARMIGSYKATLGDVAAQVLLLQSRDLDLAASN-AQPVRTAQK
9 Lelliottia           99.6%  88.9%    QTQTTNLAVVAPGIAEALLATAIGLVAAIPAVVIYNIFARMIGSYKATLGDVAAQVLLLQSRDLDLNASS-AKPVHSASK
  consensus/100%                       QoQTTNLAVVAPGIAEALLATAIGLhAAIPAVVIYNlFAR.IGuhKA.LuDlAAQVLLLQSRDLDLtASs.spPV+sApK
  consensus/90%                        QoQTTNLAVVAPGIAEALLATAIGLhAAIPAVVIYNlFAR.IGuhKA.LuDlAAQVLLLQSRDLDLtASs.spPV+sApK
  consensus/80%                        QTQTTNLAVVAPGIAEALLATAIGLlAAIPAVVIYNlFAR.IGuaKAoLGDVAAQVLLLQSRDLDLtASu.ApPV+sApK
  consensus/70%                        QTQTTNLAVVAPGIAEALLATAIGLVAAIPAVVIYNIFAR.IGuYKATLGDVAAQVLLLQSRDLDLsASu.ApPV+sAQK

                         cov    pid 241    ] 244
1 Salmonella          100.0% 100.0%    LRVG
2 Escherichia         100.0%  89.8%    LRAG
3 Citrobacter         100.0%  91.8%    LRVG
4 Klebsiella           99.6%  88.1%    LRVG
5 Cronobacter         100.0%  86.9%    LRLG
6 Enterobacter         99.6%  88.9%    LRVG
7 Phytobacter         100.0%  90.6%    LRVG
8 Kosakonia            99.6%  90.2%    LRVG
9 Lelliottia           99.6%  88.9%    LRVG
  consensus/100%                       LRhG
  consensus/90%                        LRhG
  consensus/80%                        LRlG
  consensus/70%                        LRVG
```

Percent Identity Matrix - created by Clustal2.1
#
#

1: Salmonella	100.00	89.75	91.80	88.48	86.89	89.30	90.57	90.53	89.30
2: Escherichia	89.75	100.00	90.16	84.36	84.84	85.60	86.89	85.19	85.19
3: Citrobacter	91.80	90.16	100.00	86.83	85.66	88.07	88.52	88.48	88.89
4: Klebsiella	88.48	84.36	86.83	100.00	83.95	90.12	90.95	88.89	89.71
5: Cronobacter	86.89	84.84	85.66	83.95	100.00	84.77	86.89	88.07	85.60
6: Enterobacter	89.30	85.60	88.07	90.12	84.77	100.00	91.36	88.89	97.53
7: Phytobacter	90.57	86.89	88.52	90.95	86.89	91.36	100.00	93.83	90.95
8: Kosakonia	90.53	85.19	88.48	88.89	88.07	88.89	93.83	100.00	90.12
9: Lelliottia	89.30	85.19	88.89	89.71	85.60	97.53	90.95	90.12	100.00

3. DsbC

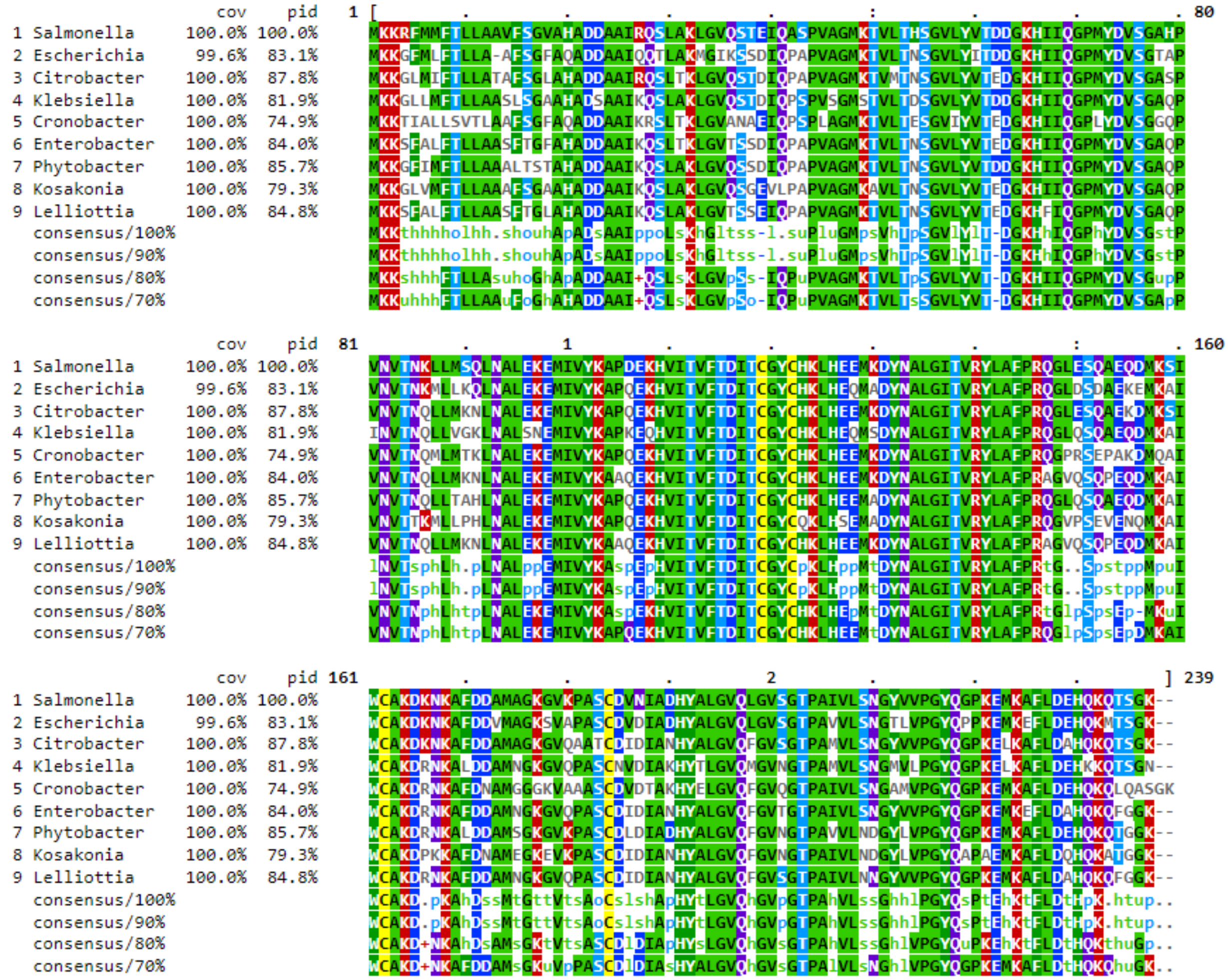

\# Percent Identity Matrix - created by Clustal2.1
\#
\#

1: Salmonella	100.00	83.47	87.76	81.86	75.53	83.97	85.65	79.32	84.81
2: Escherichia	83.47	100.00	82.20	75.85	73.73	80.51	82.63	77.97	79.24
3: Citrobacter	87.76	82.20	100.00	81.01	75.95	87.34	84.39	79.75	86.92
4: Klebsiella	81.86	75.85	81.01	100.00	71.73	80.17	83.54	74.26	79.75
5: Cronobacter	75.53	73.73	75.95	71.73	100.00	78.48	74.68	73.00	77.64
6: Enterobacter	83.97	80.51	87.34	80.17	78.48	100.00	86.92	80.59	97.05
7: Phytobacter	85.65	82.63	84.39	83.54	74.68	86.92	100.00	83.97	87.34
8: Kosakonia	79.32	77.97	79.75	74.26	73.00	80.59	83.97	100.00	81.86
9: Lelliottia	84.81	79.24	86.92	79.75	77.64	97.05	87.34	81.86	100.00

4. LolA

```
                        cov     pid    1 [         .         .         .         .         :         .         .         . 80
1 Salmonella         100.0% 100.0%     MKKMAIACALLSSVVASSVWADAASSLKSRLDKVSSFHATFTQKVTDGSGAAVQEGQGDLWVKRPNLFNWHMTQPDESIL
2 Escherichia        100.0%  94.1%     MKKIAITCALLSSLVASSVWADAASDLKSRLDKVSSFHASFTQKVTDGSGAAVQEGQGDLWVKRPNLFNWHMTQPDESIL
3 Citrobacter        100.0%  90.6%     MKKIAITCALLSGFVVSSVQADAAGDLKSRLDKVSSFHASFTQKVTDGSGAAVQEGQGDLWVKRPNLFNWHMTQPDESIL
4 Klebsiella         100.0%  89.2%     MKKLAITCALLSGMVVSQVWADAASDLKSRLDKVSSFHASFTQKVTDGSGNAVQDGQGDLWVKRPNLFNWHMTQPDESVL
5 Cronobacter        100.0%  86.8%     MKKIAVTCALLSAFAVSSVWADAAGDLKSRLDKVSSFHASFTQKVTDGSGAAVQEGQGDLWVKRPNLFNWHMTQPDESVL
6 Enterobacter       100.0%  90.6%     MKKIAIACALLTSFVASSVWADAASDLKSRLDKVSSFHASFTQKVTDGSGNAVQEGQGDLWVKRPNLFNWHMTQPDESIL
7 Phytobacter        100.0%  86.7%     MKKIAITCALIGSFMVSSVWADASSDLKSRLDKVSSFHASFTQKVTDGSGAAVQEGQGDLWVKRPNLFNWHMTQPDESVL
8 Kosakonia          100.0%  87.7%     MKRVALVCALMGSLLVSNVWADASSDLKSRLDKVSSFHATFTQKVTDGSGAAVQEGQGDLWVKRPNLFNWHMTQPDESVL
9 Lelliottia         100.0%  87.3%     MKKIAIVGALLTSFVASSVWADAASDLKNRLDKVSSFHASFTQKVTDGSGNAVQEGQGDLWVKRPNLFNWHMTQPDESVL
  consensus/100%                       MK+hAlssALhsuhhsSpV.ADAuusLKsRLDKVSSFHAoFTQKVTDGSGsAVQ-GQGDLWVKRPNLFNWHMTQPDESlL
  consensus/90%                        MK+hAlssALhsuhhsSpV.ADAuusLKsRLDKVSSFHAoFTQKVTDGSGsAVQ-GQGDLWVKRPNLFNWHMTQPDESlL
  consensus/80%                        MKKlAlsCALlsuhhsSsVWADAuuDLKSRLDKVSSFHAoFTQKVTDGSGsAVQEGQGDLWVKRPNLFNWHMTQPDESlL
  consensus/70%                        MKKlAIsCALLouhlsSSVWADAASDLKSRLDKVSSFHASFTQKVTDGSGsAVQEGQGDLWVKRPNLFNWHMTQPDESlL

                        cov     pid   81         .         1         .         .         .         .         :         . 160
1 Salmonella         100.0% 100.0%     VSDGKTLWFYNPFVEQATATWLKDATGNTPFMLIARNQASDWQQYNIKQDGDNFVLTPKASNGNLKQFTINVGRDGTIHQ
2 Escherichia        100.0%  94.1%     VSDGKTLWFYNPFVEQATATWLKDATGNTPFMLIARNQSSDWQQYNIKQNGDDFVLTPKASNGNLKQFTINVGRDGTIHQ
3 Citrobacter        100.0%  90.6%     VSDGKTLWFYNPFVEQATATWLKDATGNTPFMLIARNQSSDWQQYNIRQNGDDFVLTPKTGNGNLKQFTINVGRDGTIHQ
4 Klebsiella         100.0%  89.2%     VSDGKTLWFYNPFVEQATATWLKDATSNTPFMLIARNQSSDWQQYNIKQNGDDFVLTPKSGSGNLKQFTINVGRDGTIHQ
5 Cronobacter        100.0%  86.8%     ISDGKTLWFYNPFVEQASATWLKDATSNTPFMLIARNQSSDWQQYNIKQNGDDFVLMPKASSGNLKQFTINVSRDGTINQ
6 Enterobacter       100.0%  90.6%     VSDGKTLWFFNPFVEQATATWLKDATSNTPFMLIARNQASDWQQYNIKQNGDEFVLTPKGSNGNLKQFTINVSTNGTINQ
7 Phytobacter        100.0%  86.7%     VSDGKTLWFYNPFVEQATATWLKDATSNTPFMLIARNQSSDWQQYNIQQNGDDFVLTPKSSNGNLKQFTINVGSDGTIHQ
8 Kosakonia          100.0%  87.7%     VSDGKTLWFYNPFIEQATATWLKDATGNTPFMLIARNQASDWQQYNIEQKGDDFVLTPKGNNGNLKQFTINVGRDGTIHQ
9 Lelliottia         100.0%  87.3%     VSDGKTLWFYNPFVEQATATWLKDATSNTPFMLIARNQTSDWQQYNIKQTGDDFVLTPKAGNGNLKQFTINVSSNGTINQ
  consensus/100%                       lSDGKTLWFaNPFlEQAoATWLKDATuNTPFMLIARNQsSDWQQYNIpQpGDpFVLhPKsssGNLKQFTINVupsGTIpQ
  consensus/90%                        lSDGKTLWFaNPFlEQAoATWLKDATuNTPFMLIARNQsSDWQQYNIpQpGDpFVLhPKsssGNLKQFTINVupsGTIpQ
  consensus/80%                        VSDGKTLWFYNPFVEQATATWLKDATuNTPFMLIARNQuSDWQQYNIcQsGD-FVLTPKuusGNLKQFTINVupsGTIpQ
  consensus/70%                        VSDGKTLWFYNPFVEQATATWLKDATuNTPFMLIARNQuSDWQQYNI+QsGDDFVLTPKuuNGNLKQFTINVupDGTIpQ

                        cov     pid  161         .         .         .         2   ] 204
1 Salmonella         100.0% 100.0%     FSAVEQDDQRSAYQLKSQQNGAVDPSKFTFTPPQGVTIDDQRK-
2 Escherichia        100.0%  94.1%     FSAVEQDDQRSSYQLKSQQNGAVDAAKFTFTPPQGVTVDDQRK-
3 Citrobacter        100.0%  90.6%     FSAVEQDDQRSSYQLKSQQNGAVDAAKFTFTPPQGVTVDDQRK-
4 Klebsiella         100.0%  89.2%     FSAVEQDDQRSSYQLKSQQNGAVDASKFTFTPPKGVTVDDQRK-
5 Cronobacter        100.0%  86.8%     FSAVEQDDQRSNYELKSQQNGAVDMSKFTFTPPQGVTVDDQRNK
6 Enterobacter       100.0%  90.6%     FGAVEQDDQRSSYQLKSQQNGAVDASKFTFTPPQGVTVDDQRK-
7 Phytobacter        100.0%  86.7%     FSAIEQDDQKSNYQLKAQQNGTVDAAKFTFTPPKGVTVDDQRK-
8 Kosakonia          100.0%  87.7%     FSAIEQDDQRSSYQLKSQQNGTVDASKFTFTPPKGVTVDDQRK-
9 Lelliottia         100.0%  87.3%     FGAVEQDDQRSNYQLKTQQNGAVDASKFTFTPPQGVTVDDQRNK
  consensus/100%                       FuAlEQDDQ+SsYpLKsQQNGsVD.uKFTFTPPpGVTlDDQRp.
  consensus/90%                        FuAlEQDDQ+SsYpLKsQQNGsVD.uKFTFTPPpGVTlDDQRp.
  consensus/80%                        FuAlEQDDQRSsYQLKoQQNGsVDsuKFTFTPPpGVTVDDQRp.
  consensus/70%                        FSAVEQDDQRSsYQLKSQQNGAVDAuKFTFTPPpGVTVDDQRK.
```

```
#   Percent Identity  Matrix - created by Clustal2.1
#
#
```

1: Salmonella	100.00	94.09	90.64	89.16	87.19	90.64	86.70	87.68	87.68
2: Escherichia	94.09	100.00	96.06	93.10	90.64	92.61	91.63	89.16	90.15
3: Citrobacter	90.64	96.06	100.00	93.10	90.15	90.15	90.64	87.68	88.18
4: Klebsiella	89.16	93.10	93.10	100.00	89.66	89.66	90.15	88.18	88.67
5: Cronobacter	87.19	90.64	90.15	89.66	100.00	88.18	87.68	83.74	88.73
6: Enterobacter	90.64	92.61	90.15	89.66	88.18	100.00	87.68	85.71	93.10
7: Phytobacter	86.70	91.63	90.64	90.15	87.68	87.68	100.00	89.66	87.68
8: Kosakonia	87.68	89.16	87.68	88.18	83.74	85.71	89.66	100.00	84.24
9: Lelliottia	87.68	90.15	88.18	88.67	88.73	93.10	87.68	84.24	100.00

5. MtgA

```
                        cov    pid   1 [         .         .         .         .         :         .         .         . 80
1 Salmonella       100.0% 100.0%     -MSKRRIAPLTFLRRLLLRILAALAVFWGGGIALFSVVPVPFSAVMAERQISAWLGGEFGYVAHSDWVSMADISPWMGLA
2 Escherichia      100.0%  83.5%     -MSKSRLTVFSFVRRFLLRLMVVLAVFWGGGIALFSVAPVPFSAVMVERQVSAWLHGNFRYVAHSDWVSMDQISPWMGLA
3 Citrobacter       98.8%  84.5%     ----MKKGLIVFLRRLIVRVALALALFWGGGIALFSVAPVPFSAVMAERQIGAWLRGDFGYVAHSDWVSMDEISPWMGLA
4 Klebsiella        99.6%  82.2%     --MRLRVAPFALLKRLALRLLLIAAVFWGGGIALFSVLPVPFSAVMLERQVSAWLSGDVHYLAHSDWVGMDEISPWMGLA
5 Cronobacter       99.6%  79.3%     -MSKARGSLFVRLRRLALRAVLAVLGVWIAGILLFSVVPVPFSAVMVERQFSAWFSGDFSYVAHSDWVSMDEISPWMGLA
6 Enterobacter      99.6%  83.4%     --MSRKFGAGAWVKRALLRIVLVLAVFWGGGLALFSIMPVPFSAVMVERQLGAWLSGDFSYVAHSDWVSMDEISPFMGLA
7 Phytobacter      100.0%  74.0%     -MRGARGPLMAKIKKLLLRVVLALGIFWGGGILLFSFLPVPFSAVMVERQFGAWLTGDFGYVAHSDWVGMDEISPWAALA
8 Kosakonia        100.0%  74.9%     MRKWFCGPLKATLKRITLRILCVLALFWGGGIVLFSFLPVPFSAVMVERQLGAWFSGDFGYVAHSDWVGMDDISPWMALA
9 Lelliottia        99.6%  82.6%     --MSKRRSPLAWVKRVLLRLVLFFVVLWGGGIALFSVLPVPFSAVMVERQLGAWLTGNFSYVAHSDWTSMDAISPWMGLA
  consensus/100%                     .....phs.hshl++hhlRhhhhhhhhWhuGlhLFShhPVPFSAVMhERQhuAWhtGphtYlAHSDWsuMstISPahuLA
  consensus/90%                      .....phs.hshl++hhlRhhhhhhhhWhuGlhLFShhPVPFSAVMhERQhuAWhtGphtYlAHSDWsuMstISPahuLA
  consensus/80%                      ....h+hs.hshl+RhhLRlhhhhslhWGGGIhLFShhPVPFSAVMsERQhuAWhpGsFtYVAHSDWVuMDpISPWMuLA
  consensus/70%                      ...th+hs.hshl+RlhLRlllslulFWGGGIsLFSlhPVPFSAVMlERQluAWLsG-FuYVAHSDWVuMD-ISPWMGLA

                        cov    pid  81         .         1         .         .         .         .         :         . 160
1 Salmonella       100.0% 100.0%     VIAAEDQKFPEHWGFDVPAIEKALAHNERNESRIRGASTLSQQTAKNLFLWDGRSWVRKGLEAGLTLGIETVWSKKRILT
2 Escherichia      100.0%  83.5%     VIAAEDQKFPEHWGFDVASIEKALAHNERNENRIRGASTISQQTAKNLFLWDGRSWVRKGLEAGLTLGIETVWSKKRILT
3 Citrobacter       98.8%  84.5%     VIAAEDQHFPAHWGFDVAAIEKALSHNERHENRIRGASTLSQQTVKNLFLWDGRSWLRKGLEAGLTLGVETVWSKKRILT
4 Klebsiella        99.6%  82.2%     VIAAEDQKFPEHWGFDVSAIEKALAHNERHDTRIRGASTLSQQTAKNLFLWDGRSWLRKGLEAGLTVGIETVWSKKRILT
5 Cronobacter       99.6%  79.3%     VIAAEDQRFPEHWGFDVAAIQKAVSHNERS-SRIRGASTLSQQTAKNLFLWDGRSWLRKGLEAGLTLGIETVWSKRRILT
6 Enterobacter      99.6%  83.4%     VIAAEDQKFPEHWGFDVAAIEKALAHNERHENRVRGASTLSQQTAKNLFLWDGRSWVRKGLEAGLTLGMETVWSKKRILT
7 Phytobacter      100.0%  74.0%     VIAAEDQRFPDHWGLDFGAIEKALSHNEQHENRIRGASTISQQTIKNLLLWDGKSWVRKGFEAGLTLITEAVWTKRRILT
8 Kosakonia        100.0%  74.9%     VVAAEDQRFPDHWGLDFSAIEKALSHNERHENRIRGASTISQQTVKNLLLWDGKSWVRKGLEAGLTLGMEAVWTKRRILT
9 Lelliottia        99.6%  82.6%     VIAAEDQKFPEHWGFDVAAIEKALAHNERNETRVRGASTLSQQTAKNLFLWDGRSWLRKGLEAGLTVGIETVWSKKRILT
  consensus/100%                     VlAAEDQ+FPtHWGhDhsuIpKAluHNEpp.sRlRGASTlSQQThKNLhLWDG+SWlRKGhEAGLTlhhEsVWoK+RILT
  consensus/90%                      VlAAEDQ+FPtHWGhDhsuIpKAluHNEpp.sRlRGASTlSQQThKNLhLWDG+SWlRKGhEAGLTlhhEsVWoK+RILT
  consensus/80%                      VIAAEDQ+FP-HWGhDhuAIEKALuHNERp-sRlRGASTlSQQTsKNLhLWDG+SWlRKGLEAGLTlGhEsVWoK+RILT
  consensus/70%                      VIAAEDQ+FP-HWGFDVuAIEKALuHNERpEsRIRGASTlSQQTsKNLFLWDGRSWlRKGLEAGLTLGhETVWSK+RILT

                        cov    pid 161         .         .         .         2         .         .         .         . 240
1 Salmonella       100.0% 100.0%     VYLNIAEFGDGIFGVEAAAQRYFHKPASRLSLSEAALLAAVLPNPIRYKANAPSGYVRSRQAWIMRQMRQLGGESFMTRN
2 Escherichia      100.0%  83.5%     VYLNIAEFGDGVFGVEAAAQRYFHKPASKLTRSEAALLAAVLPNPLRFKVSSPSGYVRSRQAWILRQMYQLGGEPFMQQH
3 Citrobacter       98.8%  84.5%     VYLNIAEFGDGVFGVEAAAQRYFGKPASRLTQSEAALLAAVLPNPLRYKAAAPSGYVRSRQAWIMRQMRQLGGETFMTRN
4 Klebsiella        99.6%  82.2%     VYLNIAEFGDGIFGVEAAAQRYFHKPASQLTPGEAALLAAVLPNPIRYRADAPSGYVRSRQAWILRQMRQLGGEGFMREH
5 Cronobacter       99.6%  79.3%     VYLNIAEFGDGVFGVEEASQRYFNKPASRLSASEAALLAAVLPNPLRYKATAPSGYVRARQQWILRQMRQLGGESFMAQH
6 Enterobacter      99.6%  83.4%     VYLNIAEFGDGVFGVEAAAQRYFNKPASRLSMSEAALLAAVLPNPIRFKANAPSGYVRSRQAWIMRQMRQLGGEGFMQRN
7 Phytobacter      100.0%  74.0%     VYLNIAEFGDGIFGVEAASQRYFHKPASKLTMAEAALLAAVLPNPVRFRADAPSGYVRSRQAWIMRQMRQLGGEEFLERN
8 Kosakonia        100.0%  74.9%     VYLNIAEFGDGVFGVEEASQRYFHKPASRLTMSEAALLAAVLPNPIRFQAAAPSGYVRSRQAWILRQMRQLGGEAFLERN
9 Lelliottia        99.6%  82.6%     VYLNIAEFGEGVFGVEAAAQRYFNKPASRLTMSEAALLAAVLPNPIRFKANAPSGYVRSRQAWILRQMRQLGGEGFMARN
  consensus/100%                     VYLNIAEFG-GlFGVEtAuQRYFtKPASpLo.uEAALLAAVLPNPlRapssuPSGYVRuRQtWIhRQMhQLGGE.Fhtpp
  consensus/90%                      VYLNIAEFG-GlFGVEtAuQRYFtKPASpLo.uEAALLAAVLPNPlRapssuPSGYVRuRQtWIhRQMhQLGGE.Fhtpp
  consensus/80%                      VYLNIAEFGDGlFGVEtAuQRYFpKPAS+Lo.uEAALLAAVLPNPlRa+AsAPSGYVRSRQAWIhRQMRQLGGEsFhtpp
  consensus/70%                      VYLNIAEFGDGlFGVEAAuQRYFpKPAS+LohSEAALLAAVLPNPlRa+AsAPSGYVRSRQAWIhRQMRQLGGEsFMpcp

                        cov    pid 241   ] 243
1 Salmonella       100.0% 100.0%     QLN
2 Escherichia      100.0%  83.5%     QLD
3 Citrobacter       98.8%  84.5%     KLY
4 Klebsiella        99.6%  82.2%     KLY
5 Cronobacter       99.6%  79.3%     TLR
6 Enterobacter      99.6%  83.4%     KLM
7 Phytobacter      100.0%  74.0%     KLH
8 Kosakonia        100.0%  74.9%     KLY
9 Lelliottia        99.6%  82.6%     QLM
  consensus/100%                     pL.
  consensus/90%                      pL.
  consensus/80%                      pL.
  consensus/70%                      pLh
```

```
#  Percent Identity  Matrix - created by Clustal2.1
#
#
```

1: Salmonella	100.00	83.47	84.52	82.16	79.67	83.40	73.97	75.21	82.57
2: Escherichia	83.47	100.00	80.33	79.67	77.59	81.33	72.31	73.55	81.74
3: Citrobacter	84.52	80.33	100.00	80.33	79.83	83.68	76.99	79.08	79.92
4: Klebsiella	82.16	79.67	80.33	100.00	77.50	80.50	74.27	75.52	82.16
5: Cronobacter	79.67	77.59	79.83	77.50	100.00	77.08	72.61	73.44	77.92
6: Enterobacter	83.40	81.33	83.68	80.50	77.08	100.00	75.93	78.84	87.55
7: Phytobacter	73.97	72.31	76.99	74.27	72.61	75.93	100.00	83.47	73.44
8: Kosakonia	75.21	73.55	79.08	75.52	73.44	78.84	83.47	100.00	74.69
9: Lelliottia	82.57	81.74	79.92	82.16	77.92	87.55	73.44	74.69	100.00

Protein synthesis and translocation:

1. IleS

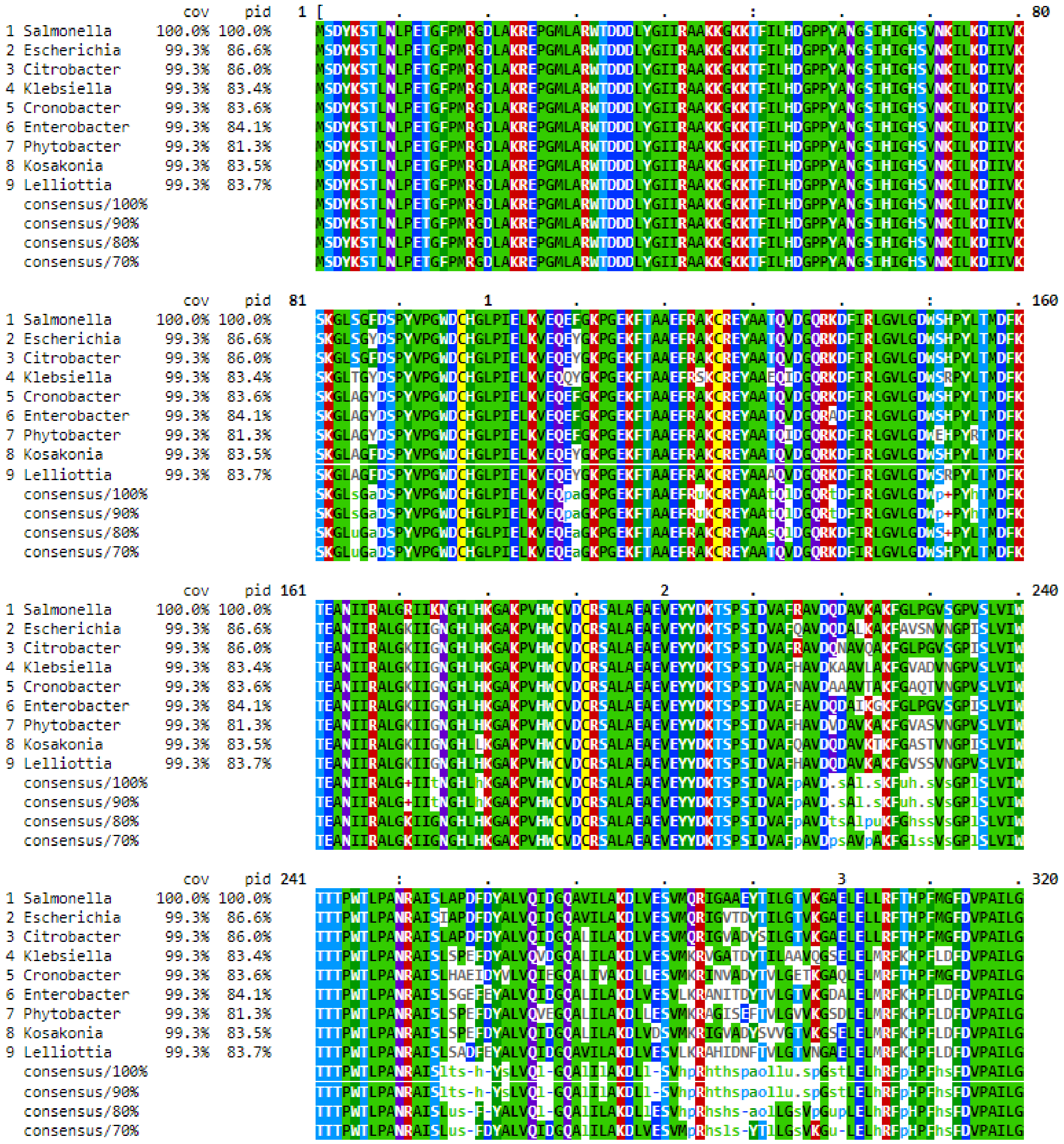

1. IleS (part 2)

```
                        cov    pid 321 .         .         :         .         .         .         .        4 400
1 Salmonella         100.0% 100.0%  DHVTLDAGTGAVHTAPGHGPDDYVIGQKYGLETANPVGPDGTYLPGTYPTLDGVNVFKANDIVIELLKEKGALLHVEKMQ
2 Escherichia         99.3%  86.6%  DHVTLDAGTGAVHTAPGHGPDDYVIGQKYGLETANPVGPDGTYLPGTYPTLDGVNVFKANDIVVALLQEKGALLHVEKMQ
3 Citrobacter         99.3%  86.0%  DHVTLDAGTGAVHTAGGHGPDDYTISLKYGLEIANPVGPDGAYLAGTYPGLDGVNVFKANDTIVELLREKGALLHVEKMQ
4 Klebsiella          99.3%  83.4%  DHVTLDAGTGAVHTAGGHGPDDYTISQKYGLEIANPVGPDGAYLPGTYPSLDGINVFKANDIIVEMLRDSGALLHVEKMQ
5 Cronobacter         99.3%  83.6%  EHVTLDAGTGAVHTAGGHGPDDYTISQKYGLEIANPVGPDGAYLPGTYPGLDGVNVFKANDQIVELLREKGALLNVAKMV
6 Enterobacter        99.3%  84.1%  DHVTLEAGTGAVHTAGGHGPDDYNISLKYGLEIANPVGPDGSYLPGTYPSLDGINVFKANDIIVEMLRERGALLHVEKMQ
7 Phytobacter         99.3%  81.3%  DHVTLEAGTGAVHTAGGHGPDDYTISQKYGLEIANPVGPDGAYLPGTYPILDGVNVFKANDIIVNLLRDNGSLLHVEKLQ
8 Kosakonia           99.3%  83.5%  DHVTLEAGTGAVHTAGGHGPDDYTISLKYGLEIANPVGPDGTYLPGTYPTLDGVNVFKANDLIVALLTEKGALLHVEKMQ
9 Lelliottia          99.3%  83.7%  DHVTLEAGTGAVHTAGGHGPDDYNISLKYGLEIANPVGPDGAYLPGTYPALDGINVFKANDIIVDILRTRGALLHVEKMQ
  consensus/100%                    -HVTL-AGTGAVHTAsGHGPDDYsIu.KYGLEhANPVGPDGsYLsGTYP.LDGlNVFKAND.llthLpppGuLLpVtKh.
  consensus/90%                     -HVTL-AGTGAVHTAsGHGPDDYsIu.KYGLEhANPVGPDGsYLsGTYP.LDGlNVFKAND.llthLpppGuLLpVtKh.
  consensus/80%                     DHVTL-AGTGAVHTAsGHGPDDYsIu.KYGLEhANPVGPDGsYLPGTYPsLDGlNVFKANDhlVthLp-pGALLHVEKMQ
  consensus/70%                     DHVTL-AGTGAVHTAGGHGPDDYsIS.KYGLEIANPVGPDGsYLPGTYPsLDGlNVFKANDlIVplL+-+GALLHVEKMQ
```

```
                        cov    pid 401 .         .         .         .         :         .         .        . 480
1 Salmonella         100.0% 100.0%  HSYPCCWRHKTPIIFRATPQWFVSMDKEGLRQQSLKEIKG-----------VQWIPDWGQARIESMVANRPDWCISRQRT
2 Escherichia         99.3%  86.6%  HSYPCCWRHKTPIIFRATPQWFVSMDQKGLRAQSLKEIKG-----------VQWIPDWGQARIESMVANRPDWCISRQRT
3 Citrobacter         99.3%  86.0%  HSYPCCWRHKSPIIFRATPQWFISMDQKGLRAQSLKEIKG-----------VQWIPDWGQARIESMVANRPDWCISRQRT
4 Klebsiella          99.3%  83.4%  HSYPCCWRHKSPIIFRATPQWFVSMDQKGLRAQSLKEIKG-----------VQWIPDWGQARIESMVANRPDWCISRQRT
5 Cronobacter         99.3%  83.6%  HSYPCCWRHKTPIIFRATPQWFVSMDQKGLRAQSLKEIKG-----------VQWIPDWGQARIESMVANRPDWCISRQRT
6 Enterobacter        99.3%  84.1%  HSYPCCWRHKTPIIFRATPQWFVSMDQKGLREQSLKEIKG-----------VQWIPDWGQARIESMVANRPDWCISRQRT
7 Phytobacter         99.3%  81.3%  HSYPCCWRHKSPIIFRATPQWFVSMDQKGLRIQSLKEIDRIEQEGLAKENLSGWIPAWGKARIESMVANRPDWCISRQRT
8 Kosakonia           99.3%  83.5%  HSYPCCWRHKSPIIFRATPQWFVSMDQKGLRAQSLSEIKG-----------VQWIPDWGQARIESMVANRPDWCISRQRT
9 Lelliottia          99.3%  83.7%  HSYPCCWRHKTPIIFRATPQWFISMDQKGLRAQSLKEIKG-----------VQWIPDWGQARIESMVANRPDWCISRQRT
  consensus/100%                    HSYPCCWRHKoPIIFRATPQWFlSMDpcGLR.QSLpEIct...........stWIPsWGpARIESMVANRPDWCISRQRT
  consensus/90%                     HSYPCCWRHKoPIIFRATPQWFlSMDpcGLR.QSLpEIct...........stWIPsWGpARIESMVANRPDWCISRQRT
  consensus/80%                     HSYPCCWRHKoPIIFRATPQWFlSMDQKGLRtQSLKEIKG...........VQWIPDWGQARIESMVANRPDWCISRQRT
  consensus/70%                     HSYPCCWRHKoPIIFRATPQWFVSMDQKGLRtQSLKEIKG...........VQWIPDWGQARIESMVANRPDWCISRQRT
```

```
                        cov    pid 481 .         5         .         .         .         .         :        . 560
1 Salmonella         100.0% 100.0%  WGVPMSLFVHKETQELLPIERTLAAMEEVAKRVEVDGIQAWWDLDPKEILGEDADQYEKVPDTLDVWFDSGSTSYSVVDA
2 Escherichia         99.3%  86.6%  WGVPMSLFVHKDTEELHPRT--LELMEEVAKRVEVDGIQAWWDLDAKEILGDEADQYVKVPDTLDVWFDSGSTHSSVVDV
3 Citrobacter         99.3%  86.0%  WGVPMALFVHKDTEELHPRA--VELMEEVAKRVEVDGIQAWWDLDPKAILGDEADQYVKVPDTLDVWFDSGSTHASVVDV
4 Klebsiella          99.3%  83.4%  WGVPMSLFVHKETHELHPRT--LELMEEVAKRVEVDGIQAWWDLDSRDILGDDADSYEKVPDTLDVWFDSGSTHSSVVDV
5 Cronobacter         99.3%  83.6%  WGVPMSLFVHKETQELHPRT--LELMEDVAKRVEQDGIQAWWDLDPRDIMGDDADVYEKVPDTLDVWFDSGSTHSSVVDV
6 Enterobacter        99.3%  84.1%  WGVPMSLFVHKETHELHPNT--LELMEEVAKRVEVDGIQAWWDLDARDILGADADNYEKVPDTLDVWFDSGSTHASVVDV
7 Phytobacter         99.3%  81.3%  WGVPMALFVHKDTEELHPRT--TELMEAVAKYVEKDGIQAWWDLDPREILGDDADNYVKVPDTLDVWFDSGSTSYSVVDA
8 Kosakonia           99.3%  83.5%  WGVPMSLFVHKDTEELHPRT--LELIEAVAKRVEVDGIQAWWDLDPRDIMGDDADNYVKVPDTLDVWFDSGSTHASVVDV
9 Lelliottia          99.3%  83.7%  WGVPMSLFVHKETQELHPNT--LELMEEVAKRVEVDGIQAWWDLDSRDILGADADSYEKVPDTLDVWFDSGSTHSSVVDV
  consensus/100%                    WGVPMuLFVHK-TpELhP.t..hthhEtVAKhVE.DGIQAWWDLDs+tIhGt-AD.Y.KVPDTLDVWFDSGSTp.SVVDs
  consensus/90%                     WGVPMuLFVHK-TpELhP.t..hthhEtVAKhVE.DGIQAWWDLDs+tIhGt-AD.Y.KVPDTLDVWFDSGSTp.SVVDs
  consensus/80%                     WGVPMuLFVHK-TpELHPps..lELMEtVAKRVEhDGIQAWWDLDs+-IhGs-ADpY.KVPDTLDVWFDSGSTp.SVVDs
  consensus/70%                     WGVPMSLFVHK-TpELHPpT..LELME-VAKRVEVDGIQAWWDLDs+-ILG-DADpY.KVPDTLDVWFDSGSTHuSVVDV
```

```
                        cov    pid 561 .         .         .         6         .         .         .        . 640
1 Salmonella         100.0% 100.0%  RPEFAGHAADMYLEGSDQHRGWFMSSLMISVAMKGKAPYRQVLTHGFTVDGQGRKMSKSIGNTVSPQDVMNKLGADILRL
2 Escherichia         99.3%  86.6%  RPEFAGHAADMYLEGSDQHRGWFMSSLMISTAMKGKAPYRQVLTHGFTVDGQGRKMSKSIGNTVSPQDVMNKLGADILRL
3 Citrobacter         99.3%  86.0%  RPEFAGHAADMYLEGSDQHRGWFMSSLMISTAMKGKAPYRQVLTHGFTVDGQGRKMSKSIGNTVSPQDVMNKLGADILRL
4 Klebsiella          99.3%  83.4%  RPEFAGHAADMYLEGSDQHRGWFMSSLMISTAMKGKAPYRQVLTHGFTVDGQGRKMSKSIGNTVSPQDVMNKLGADILRL
5 Cronobacter         99.3%  83.6%  RPEFSGHAADMYLEGSDQHRGWFMSSLMISTAMKGKAPYRQVLTHGFTVDGQGRKMSKSIGNTVSPQDVMNKLGADILRL
6 Enterobacter        99.3%  84.1%  RPEFAGHAADMYLEGSDQHRGWFMSSLMISTAMKGKAPYRQVLTHGFTVDGQGRKMSKSIGNTVSPQDVMNKLGADILRL
7 Phytobacter         99.3%  81.3%  RPEFGGHAADMYLEGSDQHRGWFMSSLMISVAMKGKAPYRQVLTHGFTVDGQGRKMSKSIGNTVSPQDVMNKLGADILRL
8 Kosakonia           99.3%  83.5%  RPEFAGHAADMYLEGSDQHRGWFMSSLMISTAMKGKAPYRQVLTHGFTVDGQGRKMSKSIGNTVSPQEVMNKLGADILRL
9 Lelliottia          99.3%  83.7%  RPEFAGHAADMYLEGSDQHRGWFMSSLMISTAMKGKAPYRQVLTHGFTVDGQGRKMSKSIGNTVSPQDVMNKLGADILRL
  consensus/100%                    RPEFuGHAADMYLEGSDQHRGWFMSSLMISsAMKGKAPYRQVLTHGFTVDGQGRKMSKSIGNTVSPQ-VMNKLGADILRL
  consensus/90%                     RPEFuGHAADMYLEGSDQHRGWFMSSLMISsAMKGKAPYRQVLTHGFTVDGQGRKMSKSIGNTVSPQ-VMNKLGADILRL
  consensus/80%                     RPEFuGHAADMYLEGSDQHRGWFMSSLMISsAMKGKAPYRQVLTHGFTVDGQGRKMSKSIGNTVSPQDVMNKLGADILRL
  consensus/70%                     RPEFAGHAADMYLEGSDQHRGWFMSSLMISTAMKGKAPYRQVLTHGFTVDGQGRKMSKSIGNTVSPQDVMNKLGADILRL
```

1. IleS (part 3)

```
                         cov    pid  641         :         .         .         .         .         7         .         . 720
 1 Salmonella         100.0% 100.0%  WVASTDYTGEMAVSDEILKRAADSYRRIRNTARFLLANLNGFNPATDMVKPEEMVVLDRWAVGCAKTAQQEILKAYEAYD
 2 Escherichia         99.3%  86.6%  WVASTDYTGEMAVSDEILKRAADSYRRIRNTARFLLANLNGFDPAKDMVKPEEMVVLDRWAVGCAKAAQEDILKAYEAYD
 3 Citrobacter         99.3%  86.0%  WVASTDYTGEMAVSDEILKRAADSYRRIRNTARFLLANLNGFDPVKDMVKPEEMVVLDRWAVGCAKAAQEDILKAYEDYD
 4 Klebsiella          99.3%  83.4%  WVASTDYTGEMAVSDEILKRAADSYRRIRNTARFLLANLNGFDPAKDMVKPEEMVVLDRWAVGCAQAAQEDILKAYESYD
 5 Cronobacter         99.3%  83.6%  WVASTDYTSEMAVSDEILKRAADSYRRIRNTARFLLANLNGFDPVKDMVKPEEMVVLDRWAVGCAKAAQDDIVKAYEAYD
 6 Enterobacter        99.3%  84.1%  WVASTDYTGEMAVSDEILKRAADSYRRIRNTARFLLANLNGFDPAKDMVKPEEMVVLDRWAVGCAKAAQEDIVKAYESYD
 7 Phytobacter         99.3%  81.3%  WVASTDYTGEMAVSDEILKRAADSYRRIRNTARFLLANLNGFDPAKDMVKPEEMVVLDRWAVGCAQAAQKDIISAYDSYD
 8 Kosakonia           99.3%  83.5%  WVASTDYTGEMAVSDEILKRAADSYRRIRNTARFLLANLNGFDPVKDMVKPEEMVVLDRWAVGCAKAAQEDIVKAYESYD
 9 Lelliottia          99.3%  83.7%  WVASTDYTGEMAVSDEILKRAADSYRRIRNTARFLLANLNGFDPVNDMVKPEEMVVLDRWAVGCAQAAQDDILKAYESYD
   consensus/100%                    WVASTDYTuEMAVSDEILKRAADSYRRIRNTARFLLANLNGFsPspDMVKPEEMVVLDRWAVGCApsAQp-IlpAY-sYD
   consensus/90%                     WVASTDYTuEMAVSDEILKRAADSYRRIRNTARFLLANLNGFsPspDMVKPEEMVVLDRWAVGCApsAQp-IlpAY-sYD
   consensus/80%                     WVASTDYTGEMAVSDEILKRAADSYRRIRNTARFLLANLNGFDPspDMVKPEEMVVLDRWAVGCApAAQcDIlKAYEuYD
   consensus/70%                     WVASTDYTGEMAVSDEILKRAADSYRRIRNTARFLLANLNGFDPsKDMVKPEEMVVLDRWAVGCApAAQ-DIlKAYEuYD
```

```
                         cov    pid  721         .         .         :         .         .         .         .       8 800
 1 Salmonella         100.0% 100.0%  FHEVVQRLMRFCSVEMGSFYLDIIKDRQYTAKADSVARRSCQTALYHIAEALVRWMAPIMSFTADEIWGYLPGEREKYVF
 2 Escherichia         99.3%  86.6%  FHEVVQRLMRFCSVEMGSFYLDIIKDRQYTAKADSVARRSCQTALYHIAEALVRWMAPILSFTADEVWGYLPGEREKYVF
 3 Citrobacter         99.3%  86.0%  FHEVVQRLMRFCSVEMGSFYLDIIKDRQYTAKADSVARRSCQSALYHIAEALVRWMAPIMSFTADEIWGYLPGEREKYVF
 4 Klebsiella          99.3%  83.4%  FHEVVQRLMRFCSIEMGSFYLDIIKDRQYTAKADSVARRSCQTALFHIVEALVRWMAPIMSFTADEIWGYLPGDREKYVF
 5 Cronobacter         99.3%  83.6%  FHEVVQRLMRFCSIEMGSFYLDIIKDRQYTAKADSVARRSCQTALYHISEALVRWMAPILSFTADEVWGYLPGEREKYVF
 6 Enterobacter        99.3%  84.1%  FHEVVQRLMRFCSIEMGSFYLDIIKDRQYTAKADSVARRSCQTALYHIAEALVRWMAPIMSFTADEIWGYLPGEREKYVF
 7 Phytobacter         99.3%  81.3%  FHEVVQRLMRFCSIEMGSFYLDIIKDRQYTAKADSVARRSCQTALYHIAEALVRWMAPIMSFTADEIWGYLPGEREKYVF
 8 Kosakonia           99.3%  83.5%  FHEVVQRLMRFCSIEMGSFYLDIIKDRQYTAKADSVARRSCQTALYHICEALVRWMAPIMSFTADEIWGYLPGSREKYVF
 9 Lelliottia          99.3%  83.7%  FHEVVQRLMRFCSIEMGSFYLDIIKDRQYTAKADSVARRSCQTALYHIAEALVRWMAPIMSFTADEIWGYLPGEREQYVF
   consensus/100%                    FHEVVQRLMRFCSlEMGSFYLDIIKDRQYTAKADSVARRSCQoALaHIsEALVRWMAPIhSFTADElWGYLPGpREpYVF
   consensus/90%                     FHEVVQRLMRFCSlEMGSFYLDIIKDRQYTAKADSVARRSCQoALaHIsEALVRWMAPIhSFTADElWGYLPGpREpYVF
   consensus/80%                     FHEVVQRLMRFCSlEMGSFYLDIIKDRQYTAKADSVARRSCQTALYHIsEALVRWMAPIhSFTADElWGYLPG-REKYVF
   consensus/70%                     FHEVVQRLMRFCSlEMGSFYLDIIKDRQYTAKADSVARRSCQTALYHIuEALVRWMAPIMSFTADEIWGYLPGEREKYVF
```

```
                         cov    pid  801         .         .         .         .         :         .         .         . 880
 1 Salmonella         100.0% 100.0%  TGEWYDGLFGLEENEEFNDAFWDDVRYIKDQVNKELENQKANG-IKSNLEAKVTLKYADDANGTIKKLKLLGEEVRFIFI
 2 Escherichia         99.3%  86.6%  TGEWYEGLFGLADSEAMNDAFWDELLKVRGEVNKVIEQARADKKVGGSLEAAVTLYAEP---ELSAKLTALGDELRFVLL
 3 Citrobacter         99.3%  86.0%  TGEWYEGLFGLGETEPMNDAFWDELLKVRGEVNKVIEQARADKKVGGSLEAAVTLYAEP---ELAAKLTALGDELRFVLL
 4 Klebsiella          99.3%  83.4%  TGEWYKGLFGLADDEAMNDGFWDELLKVRGEVNKVIEQARADKKVGGSLEAAVTLYADA---DLAAKLNALGDELRFVLL
 5 Cronobacter         99.3%  83.6%  TGEWYDGLFGLADTEAMNDAYWDALLKVRGEVNKVIEQARADKKVGGSLEAAVTLYAEP---ELAAKLTALGEELRFVLL
 6 Enterobacter        99.3%  84.1%  TGEWYEGLFDLSTTEAMNDAFWDELLKVRGEVNKVIEQARADKKVGGSLEAAVTLYAEP---ELAAKLTALGDELRFVLL
 7 Phytobacter         99.3%  81.3%  TGEWYQGLFGLAETEAMNDAYWEELLKVRGEVNKVIEQARADKKVGGSLEAAVTLFAEP---ELSAKLTALGDELRFVLL
 8 Kosakonia           99.3%  83.5%  TGEWYEGLFSLADSEAMNDAFWDELLKVRGEVNKVIEQARADKKVGGSLEAAVTLFAEP---ELAAKLTALGTELRFVLL
 9 Lelliottia          99.3%  83.7%  TGEWYEGLFGLADTEAMNDTFWDDLLKVRGEVNKVIEQARADKKVGGSLEAAVTLYAEP---ELAAKLTALGDELRFVLL
   consensus/100%                    TGEWYpGLFsLtpsE.hNDsaW-tlhhl+spVNK.lEpt+Ast.ltusLEAtVTLhhts...th.tKLphLGpElRFlhl
   consensus/90%                     TGEWYpGLFsLtpsE.hNDsaW-tlhhl+spVNK.lEpt+Ast.ltusLEAtVTLhhts...th.tKLphLGpElRFlhl
   consensus/80%                     TGEWYcGLFuLu-sEsMNDuaWD-LLKVRGEVNKVIEQARADKKVGGSLEAAVTLaA-s....-LuAKLsALG-ELRFVLL
   consensus/70%                     TGEWY-GLFGLu-oEAMNDAFWD-LLKVRGEVNKVIEQARADKKVGGSLEAAVTLaAEP...ELuAKLTALG-ELRFVLL
```

```
                         cov    pid  881         .         9         .         .         .         .         :     ] 956
 1 Salmonella         100.0% 100.0%  TSQFVISEQAGGIDDENIQYNAGNTTVQAVVTRAEGDKCPRCWHYTTDVGKVAEHADICGRCVSNIAGNGEQRKFA
 2 Escherichia         99.3%  86.6%  TSGATVADYNDAPADAQQ--SEVLKGLKVALSKAEGEKCPRCWHYTQDVGKVAEHAEICGRCVSNVAGDGEKRKFA
 3 Citrobacter         99.3%  86.0%  TSGAKVADYAEASADAQQ--SELLKGLKVALSKAEGEKCPRCWHYTTDVGKVAEHAEICGRCVSNVAGDGEKRKFA
 4 Klebsiella          99.3%  83.4%  TSGANVADYAQAPADAWQ--SDLLKGLKVVLSKAEGEKCPRCWHYTSDVGKVAEHAEICGRCVSNVAGNGEQRKFA
 5 Cronobacter         99.3%  83.6%  TSQAKVEDYASAAADAQQ--SELLKGLKVALAKAEGEKCPRCWHYTTDVGKVAEHAEICGRCVSNVAGDGEKRKFA
 6 Enterobacter        99.3%  84.1%  TSGAKVADYADASADAQQ--SELLKGLKVALSKAEGEKCPRCWHYTTDVGQVAEHADICGRCVSNVAGDGEKRKFA
 7 Phytobacter         99.3%  81.3%  TSGAKVEDYANACADAQQ--SELLKGLKVALSKAEGEKCPRCWHYTQDVGKVAEHAEICGRCVSNVAGDGEKRKFA
 8 Kosakonia           99.3%  83.5%  TSGAKVADYASASADAQQ--SELLKGLKVALSKAEGEKCPRCWHYTTDVGQVAEHADICGRCVSNVAGDGEKRKFA
 9 Lelliottia          99.3%  83.7%  TSGAQVADYAQASADAQQ--SELLKGLKVALSKADGEKCPRCWHYTTDIGQVAEHADICGRCVSNIAGDGEKRKFA
   consensus/100%                    TSth.lt-.stu.sDt....sth.pslpssls+A-G-KCPRCWHYTpDlGpVAEHA-ICGRCVSNlAGsGEpRKFA
   consensus/90%                     TSth.lt-.stu.sDt....sth.pslpssls+A-G-KCPRCWHYTpDlGpVAEHA-ICGRCVSNlAGsGEpRKFA
   consensus/80%                     TStApVtDYApAsADApQ..S-lLKGLKVsLoKAEGEKCPRCWHYTpDVGpVAEHA-ICGRCVSNlAGsGEpRKFA
   consensus/70%                     TSGApVuDYApAsADAQQ..SELLKGLKVALSKAEGEKCPRCWHYToDVGpVAEHA-ICGRCVSNVAGDGEKRKFA
```

```
#  Percent Identity  Matrix - created by Clustal2.1
#
#
```

1: Salmonella	100.00	87.30	86.77	84.10	84.31	84.85	82.92	84.20	84.42
2: Escherichia	87.30	100.00	94.67	91.58	91.79	92.43	90.41	93.18	91.79
3: Citrobacter	86.77	94.67	100.00	91.47	92.22	93.18	90.72	93.92	92.11
4: Klebsiella	84.10	91.58	91.47	100.00	90.83	92.43	91.04	91.79	92.64
5: Cronobacter	84.31	91.79	92.22	90.83	100.00	91.90	90.62	92.43	91.26
6: Enterobacter	84.85	92.43	93.18	92.43	91.90	100.00	91.36	94.14	94.67
7: Phytobacter	82.92	90.41	90.72	91.04	90.62	91.36	100.00	91.58	90.83
8: Kosakonia	84.20	93.18	93.92	91.79	92.43	94.14	91.58	100.00	92.86
9: Lelliottia	84.42	91.79	92.11	92.64	91.26	94.67	90.83	92.86	100.00

2. EngA

```
                        cov     pid   1 [         .         .         .         :         .         .         . 80
1 Salmonella         100.0%  100.0%    MVPVVALVGRPNVGKSTLFNRLTRTRDALVADFPGLTRDRKYGRAEVEGREFICIDTGGIDGTEDGVETRMAEQSLLAIE
2 Escherichia        100.0%   97.3%    MVPVVALVGRPNVGKSTLFNRLTRTRDALVADFPGLTRDRKYGRAEIEGREFICIDTGGIDGTEDGVETRMAEQSLLAIE
3 Citrobacter        100.0%   98.0%    MVPVVALVGRPNVGKSTLFNRLTRTRDALVADFPGLTRDRKYGRAEVEGREFICIDTGGIDGTEEGVETRMAEQSLLAIE
4 Klebsiella         100.0%   92.7%    MIPVVALVGRPNVGKSTLFNRLTRTRDALVADFPGLTRDRKYGRAEVEGREFICIDTGGIDGTEEGVETRMAEQSLLAIE
5 Cronobacter        100.0%   91.9%    MIPVVALVGRPNVGKSTLFNRLTRTRDALVADFPGLTRDRKYGRAEVEGREFICIDTGGIDGAEEGVETRMAEQSLLAIE
6 Enterobacter        99.8%   92.3%    MVPVVALVGRPNVGKSTLFNRLTRTRDALVADFPGLTRDRKYGRAEVEGREFICIDTGGIDGTEDGVETRMAEQSLLAIE
7 Phytobacter        100.0%   94.3%    MIPVVALVGRPNVGKSTLFNRLTRTRDALVADFPGLTRDRKYGRAEVEGREFICIDTGGIDGTEDGVETRMAEQSLLAIE
8 Kosakonia           99.8%   92.7%    MVPVVALVGRPNVGKSTLFNRLTRTRDALVADFPGLTRDRKYGRAEVEGREFICIDTGGIDGTEDGVETRMAEQSLLAIE
9 Lelliottia          99.6%   92.7%    MVPVVALVGRPNVGKSTLFNRLTRTRDALVADFPGLTRDRKYGRAEVEGREFICIDTGGIDGTEDGVETRMAEQSLLAIE
  consensus/100%                       MlPVVALVGRPNVGKSTLFNRLTRTRDALVADFPGLTRDRKYGRAElEGREFICIDTGGIDGsE-GVETRMAEQSLLAIE
  consensus/90%                        MlPVVALVGRPNVGKSTLFNRLTRTRDALVADFPGLTRDRKYGRAElEGREFICIDTGGIDGsE-GVETRMAEQSLLAIE
  consensus/80%                        MlPVVALVGRPNVGKSTLFNRLTRTRDALVADFPGLTRDRKYGRAEVEGREFICIDTGGIDGTE-GVETRMAEQSLLAIE
  consensus/70%                        MlPVVALVGRPNVGKSTLFNRLTRTRDALVADFPGLTRDRKYGRAEVEGREFICIDTGGIDGTE-GVETRMAEQSLLAIE
```

```
                        cov     pid  81           .         1         .         .         .         .         :         . 160
1 Salmonella         100.0%  100.0%    EADVVLFMVDARAGLMPADEAIAKHLRSREKPTFLVANKTDGLDPDQAVVDFYSLGLGEIYPIAASHGRGVLSLLEHVLL
2 Escherichia        100.0%   97.3%    EADVVLFMVDARAGLMPADEAIAKHLRSREKPTFLVANKTDGLDPDQAVVDFYSLGLGEIYPIAASHGRGVLSLLEHVLL
3 Citrobacter        100.0%   98.0%    EADVVLFMVDARAGLMPADEAIARHLRSREKPTFLVANKTDGLDPDQAVVDFYSLGLGEIHPIAASHGRGVLSLLEHVLL
4 Klebsiella         100.0%   92.7%    EADVVLFMVDARAGLMPADIAIAKHLRSREKPTFLVANKTDGIDVDQAMADFWSLGLGDIYPIAASHGRGVTSLLEQALL
5 Cronobacter        100.0%   91.9%    EADVVLFMVDARAGLMPADEAIAKHLRSRQKPTFLVANKTDGLDPDQAVADFYSLGLGEIHPIAASHGRGVTSLLEHVLV
6 Enterobacter        99.8%   92.3%    EADVVLFMVDARAGLMPADSAIAKHLRSREKPTFLVANKTDGIDADQAIADFWSLGLGDIYPIAASHGRGVTSLLETVLL
7 Phytobacter        100.0%   94.3%    EADVVLFMVDARAGLMPADEAIAKHLRSREKPTFLVANKTDGLDPDQAVVDFYSLGLGEIHPIAASHGRGVVSLLEHVLL
8 Kosakonia           99.8%   92.7%    EADVVLFMVDARAGLMPADSAIAKHLRSREKPTFLVANKTDGLDPDQAIADFYSLGLGEIHAIAASHGRGVTSLLEHVLM
9 Lelliottia          99.6%   92.7%    EADVVLFMVDARAGLMPADSAIAKHLRSREKPTFLVANKTDGIDADQAVADFWSLGLGDIYPIAASHGRGVTSLLETVLL
  consensus/100%                       EADVVLFMVDARAGLMPAD.AIA+HLRSRpKPTFLVANKTDGlDsDQAhsDFaSLGLG-IasIAASHGRGVhSLLEpsLh
  consensus/90%                        EADVVLFMVDARAGLMPAD.AIA+HLRSRpKPTFLVANKTDGlDsDQAhsDFaSLGLG-IasIAASHGRGVhSLLEpsLh
  consensus/80%                        EADVVLFMVDARAGLMPADpAIAKHLRSREKPTFLVANKTDGlDsDQAlsDFaSLGLG-IaPIAASHGRGVhSLLEpVLl
  consensus/70%                        EADVVLFMVDARAGLMPADpAIAKHLRSREKPTFLVANKTDGlDsDQAlsDFaSLGLG-IaPIAASHGRGVhSLLEpVLL
```

```
                        cov     pid 161           .         .         .         2         .         .         .         . 240
1 Salmonella         100.0%  100.0%    PWMDDVAPQEEVDEDAEYWAQFEAE--QNGEEAPEDDFDPQSLPIKLAIVGRPNVGKSTLTNRILGEERVVVYDMPGTTR
2 Escherichia        100.0%   97.3%    PWMEDLAPQEEVDEDAEYWAQFEAE--ENGEEEEEDDFDPQSLPIKLAIVGRPNVGKSTLTNRILGEERVVVYDMPGTTR
3 Citrobacter        100.0%   98.0%    PWMDDVAPQEEVDEDAEYWAQFEAD--ENGEEEPEDDFNPQDLPIKLAIVGRPNVGKSTLTNRILGEDRVVVYDMPGTTR
4 Klebsiella         100.0%   92.7%    PWVDEISPQEEVDEDAEYWAKFEAEQNGEAEEEPEDDFNPQDLPIKLAIVGRPNVGKSTLTNRILGEDRVVVYDMPGTTR
5 Cronobacter        100.0%   91.9%    PWMDDVDPPEEVDEEAEYWAQFEAEQNGEVVEEEEDDFNPQDLPIKLAIVGRPNVGKSTLTNRILGEDRVVVFDMPGTTR
6 Enterobacter        99.8%   92.3%    PWVDEVNPPEEVDEDAAYWAQFEEGEEGE-EEEPEETFNPQDLPIKLAIVGRPNVGKSTLTNRILGEERVVVYDMPGTTR
7 Phytobacter        100.0%   94.3%    PWMDEIDPPEELDEDAAYWAQFDEGKGEDGEEEPEDDFNPQDLPIKLAIVGRPNVGKSTLTNRILGEERVVVYDMPGTTR
8 Kosakonia           99.8%   92.7%    PWMDELNPPEVVDEEAEYWAQFEANEN-GEKEEPEDDFNPQDLPIKLAIVGRPNVGKSTLTNRILGEDRVVVYDMPGTTR
9 Lelliottia          99.6%   92.7%    PWVDEVNPQEEVDEDAAYWAQFEEGVEGA-E-EPEDDFNPQDLPIKLAIVGRPNVGKSTLTNRILGEERVVVYDMPGTTR
  consensus/100%                       PWh--lsP.E.lDE-AtYWApF-tt...t...t.E-sFsPQsLPIKLAIVGRPNVGKSTLTNRILGE-RVVVaDMPGTTR
  consensus/90%                        PWh--lsP.E.lDE-AtYWApF-tt...t...t.E-sFsPQsLPIKLAIVGRPNVGKSTLTNRILGE-RVVVaDMPGTTR
  consensus/80%                        PWhD-lsP.EEVDE-AtYWAQFEtt..tt.cEE.EDDFsPQsLPIKLAIVGRPNVGKSTLTNRILGE-RVVVYDMPGTTR
  consensus/70%                        PWhD-lsP.EEVDEDAtYWAQFEtt..tp.EEEPEDDFNPQDLPIKLAIVGRPNVGKSTLTNRILGE-RVVVYDMPGTTR
```

```
                        cov     pid 241           :         .         .         .         .         3         .         . 320
1 Salmonella         100.0%  100.0%    DSIYIPMERDEREYVLIDTAGVRKRGKITDAVEKFSVIKTLQAIEDANVVLLVIDAREGISDQDLSLLGFILNSGRSLVI
2 Escherichia        100.0%   97.3%    DSIYIPMERDGREYVLIDTAGVRKRGKITDAVEKFSVIKTLQAIEDANVVMLVIDAREGISDQDLSLLGFILNSGRSLVI
3 Citrobacter        100.0%   98.0%    DSIYIPMERDEREYVLIDTAGVRKRGKITDAVEKFSVIKTLQAIEDANVVLLVIDAREGISDQDLSLLGFILNSGRSLVI
4 Klebsiella         100.0%   92.7%    DSIYIPMQRDEREYVLIDTAGVRKRGKITDVVEKFSVIKTLQAIEDANVVLLVIDAREGISDQDLSLLGFILNSGRSLVI
5 Cronobacter        100.0%   91.9%    DSIYIPMERDGREFVLIDTAGVRKRGKITDVVEKFSVIKTLQAIEDANVVLLVIDAREGISDQDLSLLGFILNSGRSLVI
6 Enterobacter        99.8%   92.3%    DSIYIPMERDEREYVLIDTAGVRKRGKITDVVEKFSVIKTLQAIEDANVVLLVIDAREGISDQDLSLLGFILNSGRSLVI
7 Phytobacter        100.0%   94.3%    DSIYIPMERDGREYVLIDTAGVRKRGKITDAVEKFSVIKTLQAIEDANVVMLVIDAREGISDQDLSLLGFILNSGRSLVI
8 Kosakonia           99.8%   92.7%    DSIYIPMQRDEREYVLIDTAGVRKRGKITDVVEKFSVIKTLQAIEDANVVLLVIDAREGISDQDLSLLGFILNSGRSLVI
9 Lelliottia          99.6%   92.7%    DSIYIPMQRDEREYVLIDTAGVRKRGKITDVVEKFSVIKTLQAIEDANVVMLVIDAREGISDQDLSLLGFILNSGRSLVI
  consensus/100%                       DSIYIPMpRDtREaVLIDTAGVRKRGKITDsVEKFSVIKTLQAIEDANVVhLVIDAREGISDQDLSLLGFILNSGRSLVI
  consensus/90%                        DSIYIPMpRDtREaVLIDTAGVRKRGKITDsVEKFSVIKTLQAIEDANVVhLVIDAREGISDQDLSLLGFILNSGRSLVI
  consensus/80%                        DSIYIPMpRDtREYVLIDTAGVRKRGKITDsVEKFSVIKTLQAIEDANVVhLVIDAREGISDQDLSLLGFILNSGRSLVI
  consensus/70%                        DSIYIPMpRDtREYVLIDTAGVRKRGKITDsVEKFSVIKTLQAIEDANVVhLVIDAREGISDQDLSLLGFILNSGRSLVI
```

2. EngA (part 2)

```
                              cov    pid  321        .         .         :         .         .         .         .         4 400
 1 Salmonella            100.0% 100.0%       VVNKWDGLSQEVKEQVKETLDFRLGFIDFARVHFISALHGSGVGNLFESVREAYDSSTRRVSTAMLTRIMTMAVEDHQPP
 2 Escherichia           100.0%  97.3%       VVNKWDGLSQEVKEQVKETLDFRLGFIDFARVHFISALHGSGVGNLFESVREAYDSSTRRVGTSMLTRIMTMAVEDHQPP
 3 Citrobacter           100.0%  98.0%       VVNKWDGLSQEVKEQVKETLDFRLGFIDFARVHFISALHGSGVGNLFESVREAYDSSTRRVSTAMLTRIMTMAVEDHQPP
 4 Klebsiella            100.0%  92.7%       VVNKWDGLSQEVKEQVKETLDYRLGFIDFARVHFISALHGSGVGNLFESVREAYDSATRRVSTAMLTRIMNMAAEDHQPP
 5 Cronobacter           100.0%  91.9%       VVNKWDGLSQEVKDEVKETLDYRLGFIDFARVHFISALHGSGVGNLFESVREAYDSSTRRVSTALLTRIMKMAEEDHQPP
 6 Enterobacter           99.8%  92.3%       VVNKWDGLSNEVREQVKETLDFRLGFIDFARVHFISALHGSGVGNLFDSVREAYDSSTRRQSTAMLTRIMNMAAEDHQPP
 7 Phytobacter           100.0%  94.3%       VVNKWDGLSQEVKEQVKETLDFRLGFIDFARVHFISALHGSGVGNLFESVREAYDSSTRRQSTAMLTRIMNMASEDHQPP
 8 Kosakonia              99.8%  92.7%       VVNKWDGLSQEVKEQVKETLDYRLGFIDFARVHFISALHGSGVGNLFESVREAYDSSTRRVSTALLTRIMNMAAEDHQPP
 9 Lelliottia             99.6%  92.7%       VVNKWDGLSNDVREQVKEMLDFRLGFIDFARVHFISALHGSGVGNLFESVREAYDSSTRRQSTAMLTRIMTMAAEDHQPP
   consensus/100%                            VVNKWDGLSp-V+-pVKEhLDaRLGFIDFARVHFISALHGSGVGNLF-SVREAYDSuTRR.uTuhLTRIMpMA.EDHQPP
   consensus/90%                             VVNKWDGLSp-V+-pVKEhLDaRLGFIDFARVHFISALHGSGVGNLF-SVREAYDSuTRR.uTuhLTRIMpMA.EDHQPP
   consensus/80%                             VVNKWDGLSpEV+EQVKETLDaRLGFIDFARVHFISALHGSGVGNLFESVREAYDSSTRR.STAhLTRIMsMAsEDHQPP
   consensus/70%                             VVNKWDGLSQEVKEQVKETLDaRLGFIDFARVHFISALHGSGVGNLFESVREAYDSSTRR.STAMLTRIMsMAsEDHQPP

                              cov    pid  401        .         .         .         .         :         .         .         . 480
 1 Salmonella            100.0% 100.0%       LVRGRRVKLKYAHAGGYNPPIVVIHGNQVKDLPDSYKRYLMNYFRKSLEVMGTPIRIQFKEGENPYANKRNTLTPTQMRK
 2 Escherichia           100.0%  97.3%       LVRGRRVKLKYAHAGGYNPPIVVIHGNQVKDLPDSYKRYLMNYFRKSLDVMGSPIRIQFKEGENPYANKRNTLTPTQMRK
 3 Citrobacter           100.0%  98.0%       LVRGRRVKLKYAHAGGYNPPIVVIHGNQVKDLPDSYKRYLMNYFRKSLEVMGTPIRIQFKEGENPYANKRNTLTPTQMRK
 4 Klebsiella            100.0%  92.7%       LVRGRRVKLKYAHAGGYNPPIVVIHGNQVKDLPDSYKRYLMNYFRKSLDVMGTPIRIQFKEGENPFANKRNTLTPNQMRK
 5 Cronobacter           100.0%  91.9%       MVRGRRVKLKYAHAGGYNPPIVVIHGSQVKDLPDSYKRYLMNYFRKSLDVMGTPIRIQFKEGANPFANKRNTLTPNQLRK
 6 Enterobacter           99.8%  92.3%       LVRGRRVKLKYAHAGGYNPPIVVIHGNQVKDLPDSYKRYLMNYFRKSLDVMGTPIRIQFKEGDNPFANKRNTLTPNQMRK
 7 Phytobacter           100.0%  94.3%       LVRGRRVKLKYAHAGGYNPPIVVIHGNQVKDLPDSYKRYLMNYFRKSLDVMGTPIRIQFKEGENPYADKRNTLTPNQMRK
 8 Kosakonia              99.8%  92.7%       LVRGRRVKLKYAHAGGYNPPIVVIHGNQVKDLPDSYKRYLMNYFRKSLDVMGTPIRIQFKEGENPFAEKRNTLTPNQMRK
 9 Lelliottia             99.6%  92.7%       LVRGRRVKLKYAHAGGYNPPIVVIHGNQVKDLPDSYKRYLMNYFRKSLDVMGTPIRIQFKEGENPFANKRNTLTPNQMRK
   consensus/100%                            hVRGRRVKLKYAHAGGYNPPIVVIHGsQVKDLPDSYKRYLMNYFRKSL-VMGoPIRIQFKEGtNPaApKRNTLTPsQhRK
   consensus/90%                             hVRGRRVKLKYAHAGGYNPPIVVIHGsQVKDLPDSYKRYLMNYFRKSL-VMGoPIRIQFKEGtNPaApKRNTLTPsQhRK
   consensus/80%                             LVRGRRVKLKYAHAGGYNPPIVVIHGNQVKDLPDSYKRYLMNYFRKSL-VMGTPIRIQFKEG-NPaAsKRNTLTPsQMRK
   consensus/70%                             LVRGRRVKLKYAHAGGYNPPIVVIHGNQVKDLPDSYKRYLMNYFRKSLDVMGTPIRIQFKEGENPaANKRNTLTPsQMRK

                              cov    pid  481        . ] 492
 1 Salmonella            100.0% 100.0%       RKRLMKHIKKSK
 2 Escherichia           100.0%  97.3%       RKRLMKHIKKNK
 3 Citrobacter           100.0%  98.0%       RKRLIKHIKKSK
 4 Klebsiella            100.0%  92.7%       RKRLIKHIKKSK
 5 Cronobacter           100.0%  91.9%       RKRLIKHIKKSK
 6 Enterobacter           99.8%  92.3%       RKRLIKHIKKSK
 7 Phytobacter           100.0%  94.3%       RKRLIKHIKKSK
 8 Kosakonia              99.8%  92.7%       RKRLIKHIKKGK
 9 Lelliottia             99.6%  92.7%       RKRLIKHIKKSK
   consensus/100%                            RKRLhKHIKKsK
   consensus/90%                             RKRLhKHIKKsK
   consensus/80%                             RKRLhKHIKKuK
   consensus/70%                             RKRLIKHIKKSK
```

```
#  Percent Identity  Matrix - created by Clustal2.1
#
#
```

1: Salmonella	100.00	97.35	97.96	93.06	92.24	92.84	94.69	93.25	93.44
2: Escherichia	97.35	100.00	96.12	91.63	91.43	91.21	94.29	92.23	92.21
3: Citrobacter	97.96	96.12	100.00	93.67	93.27	92.84	95.31	94.07	93.44
4: Klebsiella	93.06	91.63	93.67	100.00	92.68	94.50	92.48	94.09	94.69
5: Cronobacter	92.24	91.43	93.27	92.68	100.00	91.04	92.07	93.48	90.61
6: Enterobacter	92.84	91.21	92.84	94.50	91.04	100.00	93.48	93.67	97.35
7: Phytobacter	94.69	94.29	95.31	92.48	92.07	93.48	100.00	93.28	93.67
8: Kosakonia	93.25	92.23	94.07	94.09	93.48	93.67	93.28	100.00	93.25
9: Lelliottia	93.44	92.21	93.44	94.69	90.61	97.35	93.67	93.25	100.00

3. SecY

```
                      cov    pid   1 [         .         .         .         .         :         .         .         . 80
1 Salmonella       100.0% 100.0%     MAKQPGLDFQSAKGGLGELKRRLLFVIGALIVFRIGSFIPIPGIDAAVLAKLLEQQRGTIIEMFNMFSGGALSRASIFAL
2 Escherichia      100.0%  99.8%     MAKQPGLDFQSAKGGLGELKRRLLFVIGALIVFRIGSFIPIPGIDAAVLAKLLEQQRGTIIEMFNMFSGGALSRASIFAL
3 Citrobacter      100.0% 100.0%     MAKQPGLDFQSAKGGLGELKRRLLFVIGALIVFRIGSFIPIPGIDAAVLAKLLEQQRGTIIEMFNMFSGGALSRASIFAL
4 Klebsiella       100.0%  97.7%     MAKQPGLDFQSAKGGLGELKRRLLFVVGALIVFRIGSFIPIPGIDAAVLAKLLEQQRGTIIEMFNMFSGGALSRASIFAL
5 Cronobacter      100.0%  99.1%     MAKQPGLDFQSAKGGLGELKRRLLFVIGALIVFRIGSFIPIPGIDAAVLAKLLEQQRGTIIEMFNMFSGGALSRASIFAL
6 Enterobacter     100.0%  98.9%     MAKQPGLDFQSAKGGFGELKRRLLFVIGALIVFRIGSFIPIPGIDAAVLAKLLEQQRGTIIEMFNMFSGGALSRASIFAL
7 Phytobacter      100.0%  98.4%     MAKQPGLDFQSAKGGLGELKRRLLFVIGALIVFRIGSFIPIPGIDAAVLAKLLEQQRGTIIEMFNMFSGGALSRASIFAL
8 Kosakonia        100.0%  99.1%     MAKQPGLDFQSAKGGIGELKRRLLFVIGALIVFRIGSFIPIPGIDAAVLAKLLEQQRGTIIEMFNMFSGGALSRASIFAL
9 Lelliottia       100.0%  98.6%     MAKQPGLDFQSAKGGFGELKRRLLFVVGALIVFRIGSFIPIPGIDAAVLAKLLEQQRGTIIEMFNMFSGGALSRASIFAL
  consensus/100%                     MAKQPGLDFQSAKGGhGELKRRLLFVlGALIVFRIGSFIPIPGIDAAVLAKLLEQQRGTIIEMFNMFSGGALSRASIFAL
  consensus/90%                      MAKQPGLDFQSAKGGhGELKRRLLFVlGALIVFRIGSFIPIPGIDAAVLAKLLEQQRGTIIEMFNMFSGGALSRASIFAL
  consensus/80%                      MAKQPGLDFQSAKGGhGELKRRLLFVlGALIVFRIGSFIPIPGIDAAVLAKLLEQQRGTIIEMFNMFSGGALSRASIFAL
  consensus/70%                      MAKQPGLDFQSAKGGlGELKRRLLFVIGALIVFRIGSFIPIPGIDAAVLAKLLEQQRGTIIEMFNMFSGGALSRASIFAL

                      cov    pid  81           .         1         .         .         .         .         :         . 160
1 Salmonella       100.0% 100.0%     GIMPYISASIIIQLLTVVHPTLAEIKKEGESGRRKISQYTRYGTLVLAIFQSIGIATGLPNMPGMQGLVMNPGFAFYFTA
2 Escherichia      100.0%  99.8%     GIMPYISASIIIQLLTVVHPTLAEIKKEGESGRRKISQYTRYGTLVLAIFQSIGIATGLPNMPGMQGLVINPGFAFYFTA
3 Citrobacter      100.0% 100.0%     GIMPYISASIIIQLLTVVHPTLAEIKKEGESGRRKISQYTRYGTLVLAIFQSIGIATGLPNMPGMQGLVMNPGFAFYFTA
4 Klebsiella       100.0%  97.7%     GIMPYISASIIVQLLTVVYQPLAELKKEGESGRRKISQYTRYGTLVLAIFQSIGIATGLPNMPGMQGLVINPGFAFYFTA
5 Cronobacter      100.0%  99.1%     GIMPYISASIIIQLLTVVHPTLAEMKKEGESGRRKISQYTRYGTLVLAIFQSIGIATGLPNMPGMQGLVINPGFAFYFTA
6 Enterobacter     100.0%  98.9%     GIMPYISASIIIQLLTVVHPALAELKKEGESGRRKISQYTRYGTLVLAIFQSIGIATGLPNMPGMQGLVLNPGFAFYFTA
7 Phytobacter      100.0%  98.4%     GIMPYISASIIIQLLTVVHPALAELKKEGESGRRKISQYTRYGTLVLAIFQATGIATGLPNMPGMQGLVINPGFAFYFTA
8 Kosakonia        100.0%  99.1%     GIMPYISASIIIQLLTVVHPALAELKKEGESGRRKISQYTRYGTLVLAIFQSIGIATGLPNMPGMQGLVMNPGFAFYFTA
9 Lelliottia       100.0%  98.6%     GIMPYISASIIIQLLTVVHPALAELKKEGESGRRKISQYTRYGTLVLAIFQSIGIATGLPNMPGMQGLVINPGFAFYFTA
  consensus/100%                     GIMPYISASIIlQLLTVVa.sLAEhKKEGESGRRKISQYTRYGTLVLAIFQuhGIATGLPNMPGMQGLVhNPGFAFYFTA
  consensus/90%                      GIMPYISASIIlQLLTVVa.sLAEhKKEGESGRRKISQYTRYGTLVLAIFQuhGIATGLPNMPGMQGLVhNPGFAFYFTA
  consensus/80%                      GIMPYISASIIIQLLTVVHPsLAElKKEGESGRRKISQYTRYGTLVLAIFQSIGIATGLPNMPGMQGLVhNPGFAFYFTA
  consensus/70%                      GIMPYISASIIIQLLTVVHPsLAElKKEGESGRRKISQYTRYGTLVLAIFQSIGIATGLPNMPGMQGLVhNPGFAFYFTA

                      cov    pid 161           .         .         .         2         .         .         .         . 240
1 Salmonella       100.0% 100.0%     VVSLVTGTMFLMWLGEQITERGIGNGISIIIFAGIVAGLPPAIAHTIEQARQGDLHFLVLLLVAVLVFAVTFFVVFVERG
2 Escherichia      100.0%  99.8%     VVSLVTGTMFLMWLGEQITERGIGNGISIIIFAGIVAGLPPAIAHTIEQARQGDLHFLVLLLVAVLVFAVTFFVVFVERG
3 Citrobacter      100.0% 100.0%     VVSLVTGTMFLMWLGEQITERGIGNGISIIIFAGIVAGLPPAIAHTIEQARQGDLHFLVLLLVAVLVFAVTFFVVFVERG
4 Klebsiella       100.0%  97.7%     VVSLVTGTMFLMWLGEQITERGIGNGISILIFAGIVAGLPPAIAHTIEQARQGDLHFLLLLLVAVLVFAVTFFVVFVERG
5 Cronobacter      100.0%  99.1%     VVSLVTGTMFLMWLGEQITERGIGNGISIIIFAGIVAGLPPAIGHTIEQARQGDLHFLLLLLVAVLVFAVTFFVVFVERG
6 Enterobacter     100.0%  98.9%     VVSLVTGTMFLMWLGEQITERGIGNGISIIIFAGIVAGLPPAIAHTIEQARQGDLHFLLLLLVAVLVFAVTFFVVFVERG
7 Phytobacter      100.0%  98.4%     VVSLVTGTMFLMWLGEQITERGIGNGISIIIFAGIVAGLPPAIAHTIEQARQGDLHFLLLLLVAVLVFAVTFFVVFVERG
8 Kosakonia        100.0%  99.1%     VVSLVTGTMFLMWLGEQITERGIGNGISIIIFAGIVAGLPPAIAHTIEQARQGDLHFLLLLLVAVLVFAVTFFVVFVERG
9 Lelliottia       100.0%  98.6%     VVSLVTGTMFLMWLGEQITERGIGNGISIIIFAGIVAGLPPAIAHTIEQARQGDLHFLLLLLVAVLVFAVTFFVVFVERG
  consensus/100%                     VVSLVTGTMFLMWLGEQITERGIGNGISIlIFAGIVAGLPPAIuHTIEQARQGDLHFLlLLLVAVLVFAVTFFVVFVERG
  consensus/90%                      VVSLVTGTMFLMWLGEQITERGIGNGISIlIFAGIVAGLPPAIuHTIEQARQGDLHFLlLLLVAVLVFAVTFFVVFVERG
  consensus/80%                      VVSLVTGTMFLMWLGEQITERGIGNGISIIIFAGIVAGLPPAIAHTIEQARQGDLHFLlLLLVAVLVFAVTFFVVFVERG
  consensus/70%                      VVSLVTGTMFLMWLGEQITERGIGNGISIIIFAGIVAGLPPAIAHTIEQARQGDLHFLlLLLVAVLVFAVTFFVVFVERG
```

3. SecY (part 2)

	cov	pid	241 : . . . . 3 . . 320
1 Salmonella	100.0%	100.0%	QRRIVVNYAKRQQGRRVYAAQSTHLPLKVNMAGVIPAIFASSIILFPATIASWFGGGTGWNWLTTISLYLQPGQPLYVLL
2 Escherichia	100.0%	99.8%	QRRIVVNYAKRQQGRRVYAAQSTHLPLKVNMAGVIPAIFASSIILFPATIASWFGGGTGWNWLTTISLYLQPGQPLYVLL
3 Citrobacter	100.0%	100.0%	QRRIVVNYAKRQQGRRVYAAQSTHLPLKVNMAGVIPAIFASSIILFPATIASWFGGGTGWNWLTTISLYLQPGQPLYVLL
4 Klebsiella	100.0%	97.7%	QRRIVVNYAKRQQGRRVYAAQSTHLPLKVNMAGVIPAIFASSIILFPATITSWFGGGTGWNWLTTISLYLQPGQPLYVLL
5 Cronobacter	100.0%	99.1%	QRRIVVNYAKRQQGRRVYAAQSTHLPLKVNMAGVIPAIFASSIILFPATIASWFGGGTGWNWLTTISLYLQPGQPLYVLL
6 Enterobacter	100.0%	98.9%	QRRIVVNYAKRQQGRRVYAAQSTHLPLKVNMAGVIPAIFASSIILFPATIASWFGGGTGWNWLTTISLYLQPGQPLYVLL
7 Phytobacter	100.0%	98.4%	QRRIVVNYAKRQQGRRVYAAQSTHLPLKVNMAGVIPAIFASSIILFPATIASWFGGGTGWNWLTTISLYLQPGQPLYVLL
8 Kosakonia	100.0%	99.1%	QRRIVVNYAKRQQGRRVYAAQSTHLPLKVNMAGVIPAIFASSIILFPATIASWFGGGTGWNWLTTISLYLQPGQPLYVLL
9 Lelliottia	100.0%	98.6%	QRRIVVNYAKRQQGRRVYAAQSTHLPLKVNMAGVIPAIFASSIILFPATIASWFGGGTGWNWLTTISLYLQPGQPLYVLL
consensus/100%			QRRIVVNYAKRQQGRRVYAAQSTHLPLKVNMAGVIPAIFASSIILFPATIsSWFGGGTGWNWLTTISLYLQPGQPLYVLL
consensus/90%			QRRIVVNYAKRQQGRRVYAAQSTHLPLKVNMAGVIPAIFASSIILFPATIsSWFGGGTGWNWLTTISLYLQPGQPLYVLL
consensus/80%			QRRIVVNYAKRQQGRRVYAAQSTHLPLKVNMAGVIPAIFASSIILFPATIASWFGGGTGWNWLTTISLYLQPGQPLYVLL
consensus/70%			QRRIVVNYAKRQQGRRVYAAQSTHLPLKVNMAGVIPAIFASSIILFPATIASWFGGGTGWNWLTTISLYLQPGQPLYVLL

	cov	pid	321 . . : 4 400
1 Salmonella	100.0%	100.0%	YASAIIFFCFFYTALVFNPRETADNLKKSGAFVPGIRPGEQTAKYIDKVMTRLTLVGALYITFICLIPEFMRDAMKVPFY
2 Escherichia	100.0%	99.8%	YASAIIFFCFFYTALVFNPRETADNLKKSGAFVPGIRPGEQTAKYIDKVMTRLTLVGALYITFICLIPEFMRDAMKVPFY
3 Citrobacter	100.0%	100.0%	YASAIIFFCFFYTALVFNPRETADNLKKSGAFVPGIRPGEQTAKYIDKVMTRLTLVGALYITFICLIPEFMRDAMKVPFY
4 Klebsiella	100.0%	97.7%	YASAIIFFCFFYTALVFNPRETADNLKKSGAFVPGIRPGEQTAKYIDKVMTRLTLVGALYITFICLIPEFMRDAMKVPFY
5 Cronobacter	100.0%	99.1%	YASAIIFFCFFYTALVFNPRETADNLKKSGAFVPGIRPGEQTAKYIDKVMTRLTLVGALYITFICLIPEFMRDAMKVPFY
6 Enterobacter	100.0%	98.9%	YASAIIFFCFFYTALVFNPRETADNLKKSGAFVPGIRPGEQTAKYIDKVMTRLTLVGALYITFICLIPEFMRDAMKVPFY
7 Phytobacter	100.0%	98.4%	YASAIIFFCFFYTALVFNPRETADNLKKSGAFVPGIRPGEQTAKYIDKVMTRLTLIGALYITFICLIPEFMRDAMKVPFY
8 Kosakonia	100.0%	99.1%	YASAIIFFCFFYTALVFNPRETADNLKKSGAFVPGIRPGEQTAKYIDKVMTRLTLVGALYITFICLIPEFMRDAMKVPFY
9 Lelliottia	100.0%	98.6%	YASAIIFFCFFYTALVFNPRETADNLKKSGAFVPGIRPGEQTAKYIDKVMTRLTLVGALYITFICLIPEFMRDAMKVPFY
consensus/100%			YASAIIFFCFFYTALVFNPRETADNLKKSGAFVPGIRPGEQTAKYIDKVMTRLTLlGALYITFICLIPEFMRDAMKVPFY
consensus/90%			YASAIIFFCFFYTALVFNPRETADNLKKSGAFVPGIRPGEQTAKYIDKVMTRLTLlGALYITFICLIPEFMRDAMKVPFY
consensus/80%			YASAIIFFCFFYTALVFNPRETADNLKKSGAFVPGIRPGEQTAKYIDKVMTRLTLVGALYITFICLIPEFMRDAMKVPFY
consensus/70%			YASAIIFFCFFYTALVFNPRETADNLKKSGAFVPGIRPGEQTAKYIDKVMTRLTLVGALYITFICLIPEFMRDAMKVPFY

	cov	pid	401 . . . .] 443
1 Salmonella	100.0%	100.0%	FGGTSLLIVVVVIMDFMAQVQTLMMSSQYESALKKANLKGYGR
2 Escherichia	100.0%	99.8%	FGGTSLLIVVVVIMDFMAQVQTLMMSSQYESALKKANLKGYGR
3 Citrobacter	100.0%	100.0%	FGGTSLLIVVVVIMDFMAQVQTLMMSSQYESALKKANLKGYGR
4 Klebsiella	100.0%	97.7%	FGGTSLLIVVVVIMDFMAQVQTLMMSSQYESALKKANLKGYGR
5 Cronobacter	100.0%	99.1%	FGGTSLLIVVVVIMDFMAQVQTLMMSSQYESALKKANLKGYGR
6 Enterobacter	100.0%	98.9%	FGGTSLLIVVVVIMDFMAQVQTLMMSSQYESALKKANLKGYGR
7 Phytobacter	100.0%	98.4%	FGGTSLLIVVVVIMDFMAQVQTLMMSSQYESALKKANLKGYGR
8 Kosakonia	100.0%	99.1%	FGGTSLLIVVVVIMDFMAQVQTLMMSSQYESALKKANLKGYGR
9 Lelliottia	100.0%	98.6%	FGGTSLLIVVVVIMDFMAQVQTLMMSSQYESALKKANLKGYGR
consensus/100%			FGGTSLLIVVVVIMDFMAQVQTLMMSSQYESALKKANLKGYGR
consensus/90%			FGGTSLLIVVVVIMDFMAQVQTLMMSSQYESALKKANLKGYGR
consensus/80%			FGGTSLLIVVVVIMDFMAQVQTLMMSSQYESALKKANLKGYGR
consensus/70%			FGGTSLLIVVVVIMDFMAQVQTLMMSSQYESALKKANLKGYGR

```
#  Percent Identity  Matrix - created by Clustal2.1
#
#

     1: Salmonella     100.00   99.77  100.00   97.74   99.10   98.87   98.42   99.10   98.65
     2: Escherichia     99.77  100.00   99.77   97.97   99.32   98.87   98.65   98.87   98.87
     3: Citrobacter    100.00   99.77  100.00   97.74   99.10   98.87   98.42   99.10   98.65
     4: Klebsiella      97.74   97.97   97.74  100.00   97.97   97.97   97.74   97.97   98.42
     5: Cronobacter     99.10   99.32   99.10   97.97  100.00   98.87   98.65   98.87   98.87
     6: Enterobacter    98.87   98.87   98.87   97.97   98.87  100.00   98.87   99.55   99.55
     7: Phytobacter     98.42   98.65   98.42   97.74   98.65   98.87  100.00   98.87   98.87
     8: Kosakonia       99.10   98.87   99.10   97.97   98.87   99.55   98.87  100.00   99.32
     9: Lelliottia      98.65   98.87   98.65   98.42   98.87   99.55   98.87   99.32  100.00
```

4. RbfA

```
                        cov    pid   1 [        .         .         .         .         :         .         .         . 80
1 Salmonella        100.0% 100.0%     MAKEFGRPQRVAQEMQKEIAIILQREIKDPRLGMMTTVSGVEMSRDLAYAKVFVTFLNDKDEDAVKAGIKALQEASGFIR
2 Escherichia       100.0%  97.7%     MAKEFGRPQRVAQEMQKEIALILQREIKDPRLGMMTTVSGVEMSRDLAYAKVYVTFLNDKDEDAVKAGIKALQEASGFIR
3 Citrobacter       100.0%  99.2%     MAKEFGRPQRVAQEMQKEIAIILQREIKDPRLGMMTTVSGVEMSRDLAYAKVYVTFLNDKDEDAVKAGIKALQEASGFIR
4 Klebsiella        100.0%  97.0%     MAKEFGRPQRVAQEMQKEIAIILQREIKDPRLGMMTTVSGVEMSRDLAYAKVYVTFLNDKDEAAVKAGIKALQEASGFIR
5 Cronobacter       100.0%  93.2%     MAKEFGRPQRVAQEMQKEIAIILQREIKDPRVGLMTTVSGVEVSRDLAYAKVFVTFLNDKDDAAVKAGIKALQDASGFIR
6 Enterobacter      100.0%  90.3%     MAKEFGRPQRVAQEMQKEIALILQREIKDPRVGMMTTVSGVEMSRDLAYAKVFVTFLNDQDEAAVKNGIKALQEASGFIR
7 Phytobacter       100.0%  91.0%     MAKEFGRPQRVAQEMQKEIAIILQREIKDPRVGMMTTVSGVEVSRDLAYAKVFVTFLNDKDEAAVKAGIKALQEASGFIR
8 Kosakonia         100.0%  94.7%     MAKEFGRPQRVAQELQKEIAIILQREIKDPRLGMMTTVSGVEVSRDLAYAKVFVTFLNDKDEASVKAGIKALQDASGFIR
9 Lelliottia        100.0%  90.3%     MAKEFGRPQRVGQEMQKEIALILQREIKDPRLGMMTTVSGVEMSRDLAYAKVFVTFLNDQDEAAVKNGIKALQEASGFIR
  consensus/100%                      MAKEFGRPQRVuQEhQKEIAlILQREIKDPRlGhMTTVSGVEhSRDLAYAKVaVTFLNDpD-suVKsGIKALQ-ASGFIR
  consensus/90%                       MAKEFGRPQRVuQEhQKEIAlILQREIKDPRlGhMTTVSGVEhSRDLAYAKVaVTFLNDpD-suVKsGIKALQ-ASGFIR
  consensus/80%                       MAKEFGRPQRVAQEMQKEIAlILQREIKDPRlGMMTTVSGVEhSRDLAYAKVaVTFLNDpDEsAVKsGIKALQ-ASGFIR
  consensus/70%                       MAKEFGRPQRVAQEMQKEIAlILQREIKDPRlGMMTTVSGVEhSRDLAYAKVaVTFLNDKDEsAVKAGIKALQEASGFIR

                        cov    pid  81          .         1         .         .         .  ] 134
1 Salmonella        100.0% 100.0%     SLLGKAMRLRIVPELTFFYDNSLVEGMRMSNLVTNVVKHDEERRVNPDDSKED-
2 Escherichia       100.0%  97.7%     SLLGKAMRLRIVPELTFFYDNSLVEGMRMSNLVTSVVKHDEERRVNPDDSKED-
3 Citrobacter       100.0%  99.2%     SLLGKAMRLRIVPELTFFYDNSLVEGMRMSNLVTNVVKHDEERRVNPDDSKED-
4 Klebsiella        100.0%  97.0%     SLLGKAMRLRIVPELTFFYDNSLVEGMRMSNLVTSVVKHDDERRVNPDDSKED-
5 Cronobacter       100.0%  93.2%     SLLGKAMRLRIVPELTFFYDNSLVEGMRMSNLVTSVVKHDEERRVNPDDDKEE-
6 Enterobacter      100.0%  90.3%     SLLGKAMRLRIVPELTFFYDNSLVEGMRMSNLVTSVVKHDDERRVNPADDSKED
7 Phytobacter       100.0%  91.0%     TLLGKAMRLRIVPELTFFYDNSLVEGMRMSNLVTSVVKHDDERRVNPTDDSKED
8 Kosakonia         100.0%  94.7%     SLLGKAMRLRIVPELTFFYDNSLVEGMRMSNLVTSVVKHDDERRVNPDDSKED-
9 Lelliottia        100.0%  90.3%     SLLGKAMRLRIVPELTFFYDNSLVEGMRMSNLVTSVVKHDDERRVNPADDSKED
  consensus/100%                      oLLGKAMRLRIVPELTFFYDNSLVEGMRMSNLVTsVVKHD-ERRVNPsDspc-.
  consensus/90%                       oLLGKAMRLRIVPELTFFYDNSLVEGMRMSNLVTsVVKHD-ERRVNPsDspc-.
  consensus/80%                       SLLGKAMRLRIVPELTFFYDNSLVEGMRMSNLVTsVVKHD-ERRVNPsDspc-.
  consensus/70%                       SLLGKAMRLRIVPELTFFYDNSLVEGMRMSNLVTSVVKHD-ERRVNPsDspc-.
```

```
#  Percent Identity  Matrix - created by Clustal2.1
#
#
```

1: Salmonella	100.00	97.74	99.25	96.99	93.23	90.98	91.73	94.74	90.98
2: Escherichia	97.74	100.00	98.50	97.74	92.48	91.73	90.98	93.98	91.73
3: Citrobacter	99.25	98.50	100.00	97.74	92.48	90.23	90.98	93.98	90.23
4: Klebsiella	96.99	97.74	97.74	100.00	93.23	92.48	93.23	96.24	92.48
5: Cronobacter	93.23	92.48	92.48	93.23	100.00	91.73	93.98	93.98	90.23
6: Enterobacter	90.98	91.73	90.23	92.48	91.73	100.00	95.52	90.23	98.51
7: Phytobacter	91.73	90.98	90.98	93.23	93.98	95.52	100.00	92.48	94.03
8: Kosakonia	94.74	93.98	93.98	96.24	93.98	90.23	92.48	100.00	90.23
9: Lelliottia	90.98	91.73	90.23	92.48	90.23	98.51	94.03	90.23	100.00

5. InfB

```
                          cov    pid    1 [         .         .         .         :         .         .         . 80
1 Salmonella            100.0% 100.0%     MTDVTLKALAAERQVSVDRLVQQFADAGIRKSADDSVSAQEKQTLLAHLNREAVSGPDKLTLQRKTRSTLNIPGTGGKSK
2 Escherichia            99.7%  96.6%     MTDVTIKTLAAERQTSVERLVQQFADAGIRKSADDSVSAQEKQTLIDHLNQK-NSGPDKLTLQRKTRSTLNIPGTGGKSK
3 Citrobacter           100.0%  97.3%     MTDVTVKALAAEIQTSVDRLVQQFADAGIPKSADDSVSAQEKQTLLAHLNRENGSGPDKLTLQRKTRSTLNIPGTGGKSK
4 Klebsiella            100.0%  95.1%     MTDVTIKALASEIQTSVDRLIQQFADAGIRKSADDSVTAQEKQTLLTHLNREHGSAPDKLTLQRKTRSTLNIPGTGGKSK
5 Cronobacter            99.7%  90.8%     MTDVTVKALAAEIQTSVDRLVQQFADAGIPKSAEDSVTAQEKQALLAHLNREHGSGPDKLTLQRKTRSTLNIQGTGGKSK
6 Enterobacter          100.0%  95.2%     MTDVTVKSLAAEIQTSVDRLVQQFADAGIPKTADDSVTANEKQTLLAHLNREHGSAPDKLTLQRKTRSTLNIPGTGGKSK
7 Phytobacter            99.7%  90.4%     MTDVTVKTLAAEIQTSVDRLVQQFADAGIRKSAEDSVTAQEKQTLLAHLNREHGSGPDKLTLQRKTRSTLSIQGTGGKSK
8 Kosakonia              99.7%  91.2%     MTDVTVKTLAAEIQTSVDRLVQQFADAGIRKSAEDSVTAQEKQTLLAHLNREHGSTPDKLTLQRKTRSTLSIQGTGGKSK
9 Lelliottia            100.0%  93.0%     MTDVTVKSLAAEIQTPVDRLVQQFADAGIPKSADDSVTAQEKQTLLAHLNREHGSTPDKLTLQRKTRSTLNIPGTGGKSK
  consensus/100%                          MTDVTlKsLAuEhQssV-RLlQQFADAGI.KoA-DSVoApEKQsLlsHLNpc.sSsPDKLTLQRKTRSTLsI.GTGGKSK
  consensus/90%                           MTDVTlKsLAuEhQssV-RLlQQFADAGI.KoA-DSVoApEKQsLlsHLNpc.sSsPDKLTLQRKTRSTLsI.GTGGKSK
  consensus/80%                           MTDVTlKsLAAEhQTSVDRLVQQFADAGI.KSA-DSVoAQEKQTLLsHLNREtsSsPDKLTLQRKTRSTLsI.GTGGKSK
  consensus/70%                           MTDVTlKsLAAEIQTSVDRLVQQFADAGI.KSA-DSVoAQEKQTLLAHLNREpGSuPDKLTLQRKTRSTLNI.GTGGKSK

                          cov    pid   81           .         1         .         .         .         .         :         . 160
1 Salmonella            100.0% 100.0%     SVQIEVRKKRTFVKRDPQEAERLAAEEQAQREAEEQARREAEEQAKREAQQKAEREA--------AEQAKREAAEKAKRE
2 Escherichia            99.7%  96.6%     SVQIEVRKKRTFVKRDPQEAERLAAEEQAQREAEEQARREAEESAKREAQQKAEREA--------AEQAKREAAEQAKRE
3 Citrobacter           100.0%  97.3%     SVQIEVRKKRTFVKRDPQEAERLAAEEQAQREAEEQARREAEEAAKREAQQKADREA--------AEQAKREAAEKAKRE
4 Klebsiella            100.0%  95.1%     SVQIEVRKKRTFVKRDPQEAERLAAEEQAQREAEEQARREAEEAAKREAQLKAEREA--------AEQAKREVADKAKRE
5 Cronobacter            99.7%  90.8%     SVQIEVRKKRTFVKRDPQEAERLAAEEQAKREAEEQARREAEEAAKREAEEKAKREAGDKAKREAEEQAKRDAADKAKRE
6 Enterobacter          100.0%  95.2%     SVQIEVRKTRTFVKRDPQEAERLAAEEQAQREAEEQAQREAEATAKREAELKAEREA--------AEKAKRDASEKVKRE
7 Phytobacter            99.7%  90.4%     SVQIEVRKKRTFVKRDPQEAERLAAEEEAKREAEDKARREAEEAAKREAEEKAKRDAEERAKREADEKAKRESADKAKRE
8 Kosakonia              99.7%  91.2%     SVQIEVRKKRTFVKRDPQEAERLAAEEEAMREAEEKARREAEEAAEREAEDKAKRDAEARAKREADEKAKRENAENAKRE
9 Lelliottia            100.0%  93.0%     SVQIEVRKTRTFVKRDPQEAERLAAEEQAQREAEEQAQREAEATAKREAELKAEREA--------AEKAKRDASEKAKRD
  consensus/100%                          SVQIEVRKpRTFVKRDPQEAERLAAEEpA.REAE-pApREAEttAcREAp.KAcR-A........tEpAKR-su-psKR-
  consensus/90%                           SVQIEVRKpRTFVKRDPQEAERLAAEEpA.REAE-pApREAEttAcREAp.KAcR-A........tEpAKR-su-psKR-
  consensus/80%                           SVQIEVRKpRTFVKRDPQEAERLAAEEpApREAEEpApREAEtsAKREAp.KAcR-A........sEpAKR-su-pAKRE
  consensus/70%                           SVQIEVRKKRTFVKRDPQEAERLAAEEQApREAEEQARREAEEsAKREAp.KAcREA........sEpAKR-uA-KAKRE

                          cov    pid  161           .         .         .         2         .         .         .         . 240
1 Salmonella            100.0% 100.0%     AAEKDKVSNQQTDDMTKTAQAEKARRENEAAELKRKAEEEARRKLEEEARRVAEEARRMAEEN--KWT--ATPEPVEDTS
2 Escherichia            99.7%  96.6%     AAEKDKVSNQQ-DDMTKNAQAEKARREQEAAELKRKAEEEARRKLEEEARRVAEEARRMAEEN--KWT--DNAEPTEDSS
3 Citrobacter           100.0%  97.3%     AAEKDKVSNQQTDDMTKTAQAEKVRRENEAAELKRKAEEEARRKLEEEARRVAEEARRMAEEN--KWT--DTAEPTEDSS
4 Klebsiella            100.0%  95.1%     AAEKDKVSNQHTDEMTKTAQAEKIRRENEAAELKRKSEEEARRKLEEEARRVAEEARRMAEENEKNWS--ETSDSPEDSS
5 Cronobacter            99.7%  90.8%     AAETSKVSNQQTDEVSKAAQAEKARREAEALDLKRKAEEEARRKLEENARRVAEEARRMAEENATKWESG-SEEESEDSS
6 Enterobacter          100.0%  95.2%     AAEKDKVSNQQTDEMTKTAQAEKARRENEAAELKRKAEEEARRKLEEEARRVAEEARRMAEENEKNGI--DTAEQSEDTS
7 Phytobacter            99.7%  90.4%     AAENSKVSNQQSDEMTRTAQGEKARREAEAQDLKRKAEEEARRKLEENARRVAEEARRMAEENESRWNASSD-K-EEESS
8 Kosakonia              99.7%  91.2%     AAENSKVSNQQSDEMTRTAQAEKARRESEAQELKRKAEEEARRKLEENARRVAEEARRMAEQNEANWTAPAS-EEEETTG
9 Lelliottia            100.0%  93.0%     AAEKDKVSNQQTDEMTKTAQTEKARRENEAAELKRKAEEEARRKLEEDARRVAEEARRMAEENAGVWAEQEKAKGDEDKS
  consensus/100%                          AAEpsKVSNQp.D-ho+sAQsEKhRREtEA.-LKRKuEEEARRKLEEpARRVAEEARRMAEpN...h....p.c...Eppu
  consensus/90%                           AAEpsKVSNQp.D-ho+sAQsEKhRREtEA.-LKRKuEEEARRKLEEpARRVAEEARRMAEpN...h....p.c...Eppu
  consensus/80%                           AAEpsKVSNQQoD-MT+sAQuEKsRREsEAt-LKRKAEEEARRKLEEpARRVAEEARRMAEEN..pWt..ts.c...E-oS
  consensus/70%                           AAEpsKVSNQQoD-MTKTAQAEKARREsEAtELKRKAEEEARRKLEEpARRVAEEARRMAEEN..pWs..ts.-.sEDoS

                          cov    pid  241           :         .         .         .         .         3         .         . 320
1 Salmonella            100.0% 100.0%     DYHVTTSQHARQAEDENDREVEGGRGRGRN-AKAARPAKKG-KHAESKADREEARAAVRGGKGGK-RKGSSLQQGFQKPA
2 Escherichia            99.7%  96.6%     DYHVTTSQHARQAEDESDREVEGGRGRGRN-AKAARP-KKGNKHAESKADREEARAAVRGGKGGK-RKGSSLQQGFQKPA
3 Citrobacter           100.0%  97.3%     DYHVTTSQHARQAEDENDREVEGGRSRSRS-TKAARPAKKGNKHAESKADREEARAAVRGGKGGKQRKGSALQQSFQKPA
4 Klebsiella            100.0%  95.1%     DYHVTTSQHARQAEDDNDREVEGGRGRSRS-SKAARPAKKGNKHAESKADREEARAAVRGGKGGKHRKGSALQQGFQKPA
5 Cronobacter            99.7%  90.8%     DYHVTTSQHARQAEDDSDREVEGGRSRARPAK--AARQKKSNKHSESKADREEARAAVRGGKGGK-RKGSSLQQGFNKPA
6 Enterobacter          100.0%  95.2%     DYHVTTSQHARQAEDDNDREVEGGRGRGRN-AKAARPAKKGNKHAESKADREEARAAVRGGKGGKQRKGSSLQQGFQKPA
7 Phytobacter            99.7%  90.4%     DYHVTTSHHARQAEDDSDREVEGGRGRGRNA-KAARPAKKGNKHAESKADREEARAAIRGGKGGKQRKGSALQQSFQKPA
8 Kosakonia              99.7%  91.2%     DYHVTTSQHARQAEDDNDREVEGGRGRSRTA-KAARP-KKGNKHAESKADREEARAAVRGGKGGRQRKGSALQQGFQKPA
9 Lelliottia            100.0%  93.0%     DYHVTTSQHARQAEDENDREVEAGRSRTRTAAKAARPQKKGNKHAESKADREEARAAGRGGKGGK-RKGSTLQQGFQKPA
  consensus/100%                          DYHVTTSpHARQAED-sDREVEuGRuRsRs....At..KKu.KHuESKADREEARAAhRGGKGG+.RKGSsLQQuFpKPA
  consensus/90%                           DYHVTTSpHARQAED-sDREVEuGRuRsRs....At..KKu.KHuESKADREEARAAhRGGKGG+.RKGSsLQQuFpKPA
  consensus/80%                           DYHVTTSQHARQAED-sDREVEGGRuRuRs..KAARP.KKGNKHAESKADREEARAAlRGGKGGK.RKGSuLQQuFQKPA
  consensus/70%                           DYHVTTSQHARQAED-sDREVEGGRuRuRs.tKAARPtKKGNKHAESKADREEARAAVRGGKGGK.RKGSuLQQGFQKPA
```

5. InfB (part 2)

```
                       cov    pid  321        .         .         :         .         .         .         .         4 400
1 Salmonella       100.0% 100.0%   QAVNRDVVIGETITVGELANKMAVKGSQVIKAMMKLGAMATINQVIDQETAQLVAEEMGHKVILRRENELEEAVMSDRDT
2 Escherichia       99.7%  96.6%   QAVNRDVVIGETITVGELANKMAVKGSQVIKAMMKLGAMATINQVIDQETAQLVAEEMGHKVILRRENELEEAVMSDRDT
3 Citrobacter      100.0%  97.3%   QAVNRDVVIGETITVGELANKMAVKGSQVIKAMMKLGAMATINQVIDQETAQLVAEEMGHKVILRRENELEEAVMSDRDT
4 Klebsiella       100.0%  95.1%   QAVNRDVIIGETITVGELANKMAVKGSQVIKAMMKLGAMATINQVIDQETAQLVAEEMGHKVILRRENELEEAVMSDRDT
5 Cronobacter       99.7%  90.8%   QAVNRDVVIGETITVGELANKMAVKGSQVIKAMMKLGAMATINQVIDQETAQLVAEEMGHKVILRRENELEEAVMSDRDM
6 Enterobacter     100.0%  95.2%   QAVNRDVVIGETITVGDLANKMAVKGSQVIKAMMKLGAMATINQVIDQETAQLVAEEMGHKVILRRENELEEAVMSDRDT
7 Phytobacter       99.7%  90.4%   QAVNRDVVIGETLTVGELANKMAVKGSQVIKAMMKLGAMATINQVIDQETAQLVAEEMGHKVILRRENELEEAVMSDRDT
8 Kosakonia         99.7%  91.2%   QAVNRDVVIGETLTVGELANKMAVKGSQVIKAMMKLGAMATINQVIDQETAQLVAEEMGHKVILRRENELEEAVMSDRDT
9 Lelliottia       100.0%  93.0%   QAVNRDVVIGETITVGELANKMAVKGSQVIKAMMKLGAMATINQVIDQETAQLVAEEMGHKVILRRENELEEAVMSDRDT
  consensus/100%                   QAVNRDVlIGETlTVG-LANKMAVKGSQVIKAMMKLGAMATINQVIDQETAQLVAEEMGHKVILRRENELEEAVMSDRDh
  consensus/90%                    QAVNRDVlIGETlTVG-LANKMAVKGSQVIKAMMKLGAMATINQVIDQETAQLVAEEMGHKVILRRENELEEAVMSDRDh
  consensus/80%                    QAVNRDVVIGETlTVGELANKMAVKGSQVIKAMMKLGAMATINQVIDQETAQLVAEEMGHKVILRRENELEEAVMSDRDT
  consensus/70%                    QAVNRDVVIGETITVGELANKMAVKGSQVIKAMMKLGAMATINQVIDQETAQLVAEEMGHKVILRRENELEEAVMSDRDT

                       cov    pid  401        .         .         .         .         :         .         .         . 480
1 Salmonella       100.0% 100.0%   GAAAEPRAPVVTIMGHVDHGKTSLLDYIRSTKVASGEAGGITQHIGAYHVETDNGMITFLDTPGHAAFTSMRARGAQATD
2 Escherichia       99.7%  96.6%   GAAAEPRAPVVTIMGHVDHGKTSLLDYIRSTKVASGEAGGITQHIGAYHVETENGMITFLDTPGHAAFTSMRARGAQATD
3 Citrobacter      100.0%  97.3%   GAAAEPRAPVVTIMGHVDHGKTSLLDYIRSTKVASGEAGGITQHIGAYHVETDNGMITFLDTPGHAAFTSMRARGAQATD
4 Klebsiella       100.0%  95.1%   GAAAEPRAPVVTIMGHVDHGKTSLLDYIRSTKVASGEAGGITQHIGAYHVETDNGMITFLDTPGHAAFTSMRARGAQATD
5 Cronobacter       99.7%  90.8%   GAQAEPRAPVVTIMGHVDHGKTSLLDYIRSTKVASGEAGGITQHIGAYHVQTDNGMITFLDTPGHAAFTAMRARGAQATD
6 Enterobacter     100.0%  95.2%   GAAAEPRAPVVTIMGHVDHGKTSLLDYIRSTKVASGEAGGITQHIGAYHVETDNGMITFLDTPGHAAFTSMRARGAQATD
7 Phytobacter       99.7%  90.4%   GAAAEPRAPVVTIMGHVDHGKTSLLDYIRSTKVASGEAGGITQHIGAYHVETENGMITFLDTPGHAAFTAMRARGAQATD
8 Kosakonia         99.7%  91.2%   GAAAEPRAPVVTIMGHVDHGKTSLLDYIRSTKVASGEAGGITQHIGAYHVETDNGMITFLDTPGHAAFTAMRARGAQATD
9 Lelliottia       100.0%  93.0%   GAAAEARAPVVTIMGHVDHGKTSLLDYIRSTKVASGEAGGITQHIGAYHVETENGMITFLDTPGHAAFTSMRARGAQATD
  consensus/100%                   GAtAEsRAPVVTIMGHVDHGKTSLLDYIRSTKVASGEAGGITQHIGAYHVpT-NGMITFLDTPGHAAFTuMRARGAQATD
  consensus/90%                    GAtAEsRAPVVTIMGHVDHGKTSLLDYIRSTKVASGEAGGITQHIGAYHVpT-NGMITFLDTPGHAAFTuMRARGAQATD
  consensus/80%                    GAAAEPRAPVVTIMGHVDHGKTSLLDYIRSTKVASGEAGGITQHIGAYHVET-NGMITFLDTPGHAAFTuMRARGAQATD
  consensus/70%                    GAAAEPRAPVVTIMGHVDHGKTSLLDYIRSTKVASGEAGGITQHIGAYHVET-NGMITFLDTPGHAAFTuMRARGAQATD

                       cov    pid  481        .         5         .         .         .         .         :         . 560
1 Salmonella       100.0% 100.0%   IVVLVVAADDGVMPQTIEAIQHAKAAGVPVVVAVNKIDKPEADPDRVKNELSQYGILPEEWGGESQFVHVSAKAGTGIDE
2 Escherichia       99.7%  96.6%   IVVLVVAADDGVMPQTIEAIQHAKAAQVPVVVAVNKIDKPEADPDRVKNELSQYGILPEEWGGESQFVHVSAKAGTGIDE
3 Citrobacter      100.0%  97.3%   IVVLVVAADDGVMPQTIEAIQHAKAAGVPVVVAVNKIDKPEADPDRVKNELSQYGILPEEWGGESQFVHVSAKAGTGIDE
4 Klebsiella       100.0%  95.1%   IVVLVVAADDGVMPQTIEAIQHAKAAQVPVVVAVNKIDKPEADPDRVKNELSQYGILPEEWGGESQFVHVSAKAGTGIDD
5 Cronobacter       99.7%  90.8%   IVVLVVAADDGVMPQTIEAIQHAKAAKVPVVVAVNKIDKPDADPDRVKNELSQHGIIPEEWGGESQFVHVSAKAGTGIDE
6 Enterobacter     100.0%  95.2%   IVVLVVAADDGVMPQTIEAIQHAKAAGVPVVVAVNKIDKPEADPDRVKNELSQYGILPEEWGGESQFVHVSAKAGTGIDE
7 Phytobacter       99.7%  90.4%   IVVLVVAADDGVMPQTIEAIQHAKAAQVPVVVAVNKIDKPEADPDRVKNELSQYGIMPEEWGGESQFVHVSAKAGTGIDE
8 Kosakonia         99.7%  91.2%   IVVLVVAADDGVMPQTIEAIQHAKAAQVPVVVAVNKIDKPEADPDRVKNELSQYGIIPEEWGGESQFVHVSAKAGTGIDE
9 Lelliottia       100.0%  93.0%   IVVLVVAADDGVMPQTIEAIQHAKAAQVPVVVAVNKIDKPEADMDRVKNELSQYGVMPEEWGGESQFIPVSAKAGTGIDD
  consensus/100%                   IVVLVVAADDGVMPQTIEAIQHAKAAtVPVVVAVNKIDKP-AD.DRVKNELSQaGlhPEEWGGESQFl.VSAKAGTGID-
  consensus/90%                    IVVLVVAADDGVMPQTIEAIQHAKAAtVPVVVAVNKIDKP-AD.DRVKNELSQaGlhPEEWGGESQFl.VSAKAGTGID-
  consensus/80%                    IVVLVVAADDGVMPQTIEAIQHAKAAtVPVVVAVNKIDKPEADPDRVKNELSQYGIhPEEWGGESQFVHVSAKAGTGID-
  consensus/70%                    IVVLVVAADDGVMPQTIEAIQHAKAAtVPVVVAVNKIDKPEADPDRVKNELSQYGIlPEEWGGESQFVHVSAKAGTGIDE

                       cov    pid  561        .         .         .         6         .         .         .         . 640
1 Salmonella       100.0% 100.0%   LLDAILLQAEVLELKAVRKGMASGAVIESFLDKGRGPVATVLVREGTLHKGDIVLCGFEYGRVRAMRNELGQEVLEAGPS
2 Escherichia       99.7%  96.6%   LLDAILLQAEVLELKAVRKGMASGAVIESFLDKGRGPVATVLVREGTLHKGDIVLCGFEYGRVRAMRNELGQEVLEAGPS
3 Citrobacter      100.0%  97.3%   LLDAILLQAEVLELKAVRKGMASGAVIESFLDKGRGPVATVLVREGTLNKGDIVLCGFEYGRVRAMRNELGQEVLEAGPS
4 Klebsiella       100.0%  95.1%   LLDAILLQAEVLELKAVRNGMASGAVIESFLDKGRGPVATVLVREGTLHKGDIVLCGFEYGRVRAMRDELGREVLEAGPS
5 Cronobacter       99.7%  90.8%   LLDAILLQSEVLELHAVRKGMATGVVIESFLDKGRGPVATVLVREGTLNKGDIVLCGFEYGRVRAMRNELNQEVQEAGPS
6 Enterobacter     100.0%  95.2%   LLDAILLQAEVLELKAIRNGMASGAVIESFLDKGRGPVATVLVREGTLHKGDIVLCGFEYGRVRAMRNELGQEVLEAGPS
7 Phytobacter       99.7%  90.4%   LLDAILLQAEVLELKAIRTGMASGVVIESFLDKGRGPVASVLVREGTLHKGDIVLCGFEYGRVRAMRDELGREVSEAGPS
8 Kosakonia         99.7%  91.2%   LLDAILLQAEVLELKAVRKGMASGVVIESFLDKGRGPVASVLVREGTLHKGDIVLCGFEYGRVRAMRDELGREVTEAGPS
9 Lelliottia       100.0%  93.0%   LLNAILLQAEVLELKAVRNGMASGAVIESFLDKGRGPVATVLVREGTLNKGDIVLCGFEYGRVRAMRNELGQEVLEAGPS
  consensus/100%                   LLsAILLQuEVLEL+AlRpGMAoGsVIESFLDKGRGPVAoVLVREGTLpKGDIVLCGFEYGRVRAMRsELspEV.EAGPS
  consensus/90%                    LLsAILLQuEVLEL+AlRpGMAoGsVIESFLDKGRGPVAoVLVREGTLpKGDIVLCGFEYGRVRAMRsELspEV.EAGPS
  consensus/80%                    LLDAILLQAEVLELKAlRpGMASGsVIESFLDKGRGPVAoVLVREGTLpKGDIVLCGFEYGRVRAMRsELGpEV.EAGPS
  consensus/70%                    LLDAILLQAEVLELKAVRpGMASGsVIESFLDKGRGPVATVLVREGTLpKGDIVLCGFEYGRVRAMRsELGpEVhEAGPS
```

5. InfB (part 3)

```
                         cov    pid  641          :         .         .         .         .         7         .         . 720
1 Salmonella          100.0% 100.0%  IPVEILGLSGVPAAGDEVTVVRDEKKAREVALYRQGKFREVKLARQQKSKLENMFANMTEGEVHEVNIVLKADVQGSVEA
2 Escherichia          99.7%  96.6%  IPVEILGLSGVPAAGDEVTVVRDEKKAREVALYRQGKFREVKLARQQKSKLENMFANMTEGEVHEVNIVLKADVQGSVEA
3 Citrobacter         100.0%  97.3%  IPVEILGLSGVPAAGDEVTVVRDEKKAREVALYRQGKFREVKLARQQKSKLENMFANMTEGEVHEVNIVLKADVQGSVEA
4 Klebsiella          100.0%  95.1%  IPVEILGLSGVPAAGDEVTVVRDEKKAREVALYRQGKFREVKLARQQKSKLENMFANMTEGEVHEVNIVLKADVQGSVEA
5 Cronobacter          99.7%  90.8%  IPVEILGLSGVPAAGDEVTVVRDEKKAREVALYRQGKFREVKLARQQKSKLENMFANMTEGEVHEVNIVLKADVQGSVEA
6 Enterobacter        100.0%  95.2%  IPVEILGLSGVPAAGDEVTVVRDEKKAREVALYRQGKFREVKLARQQKSKLENMFANMAEGEVHEVNVVLKADVQGSVEA
7 Phytobacter          99.7%  90.4%  IPVEILGLSGVPAAGDEATVVRDEKKAREVALYRQGKFREVKLARQQKSKLENMFANMTEGEVHEVNVVLKADVQGSVEA
8 Kosakonia            99.7%  91.2%  IPVEILGLSGVPAAGDEATVVRDEKKAREVALYRQGKFREVKLARQQKSKLENMFANMTEGEVHEVNIVLKADVQGSVEA
9 Lelliottia          100.0%  93.0%  IPVEILGLSGVPAAGDEVTVVRDEKKAREVALYRQGKFREVKLARQQKSKLENMFANMTDGEVHEVNVVLKADVQGSVEA
  consensus/100%                     IPVEILGLSGVPAAGDEsTVVRDEKKAREVALYRQGKFREVKLARQQKSKLENMFANMs-GEVHEVNlVLKADVQGSVEA
  consensus/90%                      IPVEILGLSGVPAAGDEsTVVRDEKKAREVALYRQGKFREVKLARQQKSKLENMFANMs-GEVHEVNlVLKADVQGSVEA
  consensus/80%                      IPVEILGLSGVPAAGDEsTVVRDEKKAREVALYRQGKFREVKLARQQKSKLENMFANMTEGEVHEVNlVLKADVQGSVEA
  consensus/70%                      IPVEILGLSGVPAAGDEVTVVRDEKKAREVALYRQGKFREVKLARQQKSKLENMFANMTEGEVHEVNlVLKADVQGSVEA
```

```
                         cov    pid  721          .         .         :         .         .         .         .         8 800
1 Salmonella          100.0% 100.0%  ISDSLLKLSTDEVKVKIIGSGVGGITETDATLAAASNAILVGFNVRADASARKVIESESLDLRYYSVIYNLIDEVKAAMS
2 Escherichia          99.7%  96.6%  ISDSLLKLSTDEVKVKIIGSGVGGITETDATLAAASNAILVGFNVRADASARKVIEAESLDLRYYSVIYNLIDEVKAAMS
3 Citrobacter         100.0%  97.3%  ISDSLLKLSTDEVKVKIIGSGVGGITETDATLAAASNAILVGFNVRADASARKVIEAENLDLRYYSVIYNLIDEVKAAMS
4 Klebsiella          100.0%  95.1%  ISDSLLKLSTDEVKVKIIGSGVGGITETDATLAAASNAILVGFNVRADASARKVIEAESLDLRYYSVIYNLIDEVKAAMS
5 Cronobacter          99.7%  90.8%  ISDSLLKLSTDEVKVKIVGSGVGGITETDATLAAASNAILVGFNVRADASARRVIEAESLDLRYYSVIYNLIDEVKAAMS
6 Enterobacter        100.0%  95.2%  ISDSLLKLSTDEVKVKIIGSGVGGITETDATLAAASNAILVGFNVRADASARRVIEAESLDLRYYSVIYNLIDEVKAAMS
7 Phytobacter          99.7%  90.4%  ITDSLLKLSTDEVKVKIVGSGVGGITETDATLATASNAILVGFNVRADASARRVIEAEGLDLRYYSVIYNLIDEVKAAMS
8 Kosakonia            99.7%  91.2%  ISDSLLKLSTDEVKVKIVGSGVGGITETDATLAAASNAILVGFNVRADASARRVIEAEGLDLRYYSVIYNLIDEVKAAMS
9 Lelliottia          100.0%  93.0%  ISDSLLKLSTDEVKVKIIGSGVGGITETDATLAAASNAILVGFNVRADASARKVIDAESLDLRYYSVIYHLIDEVKAAMS
  consensus/100%                     IoDSLLKLSTDEVKVKIlGSGVGGITETDATLAsASNAILVGFNVRADASAR+VI-uEsLDLRYYSVIYpLIDEVKAAMS
  consensus/90%                      IoDSLLKLSTDEVKVKIlGSGVGGITETDATLAsASNAILVGFNVRADASAR+VI-uEsLDLRYYSVIYpLIDEVKAAMS
  consensus/80%                      ISDSLLKLSTDEVKVKIlGSGVGGITETDATLAAASNAILVGFNVRADASAR+VIEAEuLDLRYYSVIYNLIDEVKAAMS
  consensus/70%                      ISDSLLKLSTDEVKVKIlGSGVGGITETDATLAAASNAILVGFNVRADASAR+VIEAEuLDLRYYSVIYNLIDEVKAAMS
```

```
                         cov    pid  801          .         .         .         .         :         .         .         . 880
1 Salmonella          100.0% 100.0%  GMLSPELKQQIIGLAEVRDVFKSPKFGAIAGCMVTEGTIKRHNPIRVLRDNVVIYEGELESLRRFKDDVNEVRNGMECGI
2 Escherichia          99.7%  96.6%  GMLSPELKQQIIGLAEVRDVFKSPKFGAIAGCMVTEGVVKRHNPIRVLRDNVVIYEGELESLRRFKDDVNEVRNGMECGI
3 Citrobacter         100.0%  97.3%  GMLSPELKQQIIGLAEVRDVFKSPKFGAIAGCMVTEGTIKRHNPIRVLRDNVVIYEGELESLRRFKDDVNEVRNGMECGI
4 Klebsiella          100.0%  95.1%  GMLSPELKQQIIGLAEVRDVFKSPKFGAIAGCMVTEGTIKRHNPIRVLRDNVVIYEGELESLRRFKDDVNEVRNGMECGI
5 Cronobacter          99.7%  90.8%  GMLSPELKQQIIGLAEVRDVFKSPKFGAIAGCMVTEGTIKRHNPIRVLRDNVVIYEGELESLRRFKDDVNEVRNGMECGI
6 Enterobacter        100.0%  95.2%  GMLSPELKQQIIGLAEVRDVFKSPKFGAIAGCMVTEGTIKRHNPIRVLRDNVVIYEGELESLRRFKDDVNEVRNGMECGI
7 Phytobacter          99.7%  90.4%  GMLSPELKQQIIGLAEVRDVFKSPKFGAIAGCMVTEGTIKRHNPIRVLRDNVVIYEGELESLRRFKDDVNEVRNGMECGI
8 Kosakonia            99.7%  91.2%  GMLSPELKQQIIGLAEVRDVFKSPKFGAIAGCMVTEGVVKRHNPIRVLRDNVVIYEGELESLRRFKDDVNEVRNGMECGI
9 Lelliottia          100.0%  93.0%  GMLSPELKQQIIGLAEVRDVFKSPKFGAIAGCMVTEGTIKRHNPIRVLRDNVVIYEGELESLRRFKDDVNEVRNGMECGI
  consensus/100%                     GMLSPELKQQIIGLAEVRDVFKSPKFGAIAGCMVTEGslKRHNPIRVLRDNVVIYEGELESLRRFKDDVNEVRNGMECGI
  consensus/90%                      GMLSPELKQQIIGLAEVRDVFKSPKFGAIAGCMVTEGslKRHNPIRVLRDNVVIYEGELESLRRFKDDVNEVRNGMECGI
  consensus/80%                      GMLSPELKQQIIGLAEVRDVFKSPKFGAIAGCMVTEGslKRHNPIRVLRDNVVIYEGELESLRRFKDDVNEVRNGMECGI
  consensus/70%                      GMLSPELKQQIIGLAEVRDVFKSPKFGAIAGCMVTEGTIKRHNPIRVLRDNVVIYEGELESLRRFKDDVNEVRNGMECGI
```

```
                         cov    pid  881          .         9      ] 907
1 Salmonella          100.0% 100.0%  GVKNYNDVRVGDMIEVFEIIEIQRTIA
2 Escherichia          99.7%  96.6%  GVKNYNDVRTGDVIEVFEIIEIQRTIA
3 Citrobacter         100.0%  97.3%  GVKNYNDVRVGDMIEVFEIIEIQRTIA
4 Klebsiella          100.0%  95.1%  GVKNYNDVRVGDMIEVFEIIEIQRSID
5 Cronobacter          99.7%  90.8%  GVKNYNDVRVGDMIEVFEIIEIQRSID
6 Enterobacter        100.0%  95.2%  GVKNYNDVRVGDMIEVFEIIEIQRSID
7 Phytobacter          99.7%  90.4%  GVKNYNDVRVGDMIEVFEIIEIQRSID
8 Kosakonia            99.7%  91.2%  GVKNYNDVRTGDMIEVFEIIEIQRTIE
9 Lelliottia          100.0%  93.0%  GVKNYNDVRVGDMIEVFEIIEIQRSIA
  consensus/100%                     GVKNYNDVRsGDhIEVFEIIEIQRoIt
  consensus/90%                      GVKNYNDVRsGDhIEVFEIIEIQRoIt
  consensus/80%                      GVKNYNDVRsGDMIEVFEIIEIQRoIs
  consensus/70%                      GVKNYNDVRVGDMIEVFEIIEIQRoIs
```

```
#  Percent Identity  Matrix - created by Clustal2.1
#
#
```

1: Salmonella	100.00	97.08	97.53	95.52	92.58	95.63	92.24	93.03	93.61
2: Escherichia	97.08	100.00	96.40	94.49	91.88	94.27	92.00	92.79	92.92
3: Citrobacter	97.53	96.40	100.00	95.75	93.26	95.30	92.59	93.15	94.06
4: Klebsiella	95.52	94.49	95.75	100.00	92.04	94.98	92.72	92.83	93.30
5: Cronobacter	92.58	91.88	93.26	92.04	100.00	92.26	92.11	91.78	90.84
6: Enterobacter	95.63	94.27	95.30	94.98	92.26	100.00	92.50	92.39	94.86
7: Phytobacter	92.24	92.00	92.59	92.72	92.11	92.50	100.00	95.24	90.84
8: Kosakonia	93.03	92.79	93.15	92.83	91.78	92.39	95.24	100.00	90.61
9: Lelliottia	93.61	92.92	94.06	93.30	90.84	94.86	90.84	90.61	100.00

DNA replication/repair and cell division:

1. RecA

```
                   cov    pid   1 [        .         .         .         :         .         .         . 80
1 Salmonella     100.0% 100.0%    MAIDENKQKALAAALGQIEKQFGKGSIMRLGEDRSMDVETISTGSLSLDIALGAGGLPMGRIVEIYGPESSGKTTLTLQV
2 Escherichia    100.0%  97.2%    MAIDENKQKALAAALGQIEKQFGKGSIMRLGEDRSMDVETISTGSLSLDIALGAGGLPMGRIVEIYGPESSGKTTLTLQV
3 Citrobacter    100.0%  98.3%    MAIDENKQKALAAALGQIEKQFGKGSIMRLGEDRSMDVETISTGSLSLDIALGAGGLPMGRIVEIYGPESSGKTTLTLQV
4 Klebsiella      99.4%  95.2%    MAIDENKQKALAAALGQIEKQFGKGSIMRLGEDRSMDVETISTGSLSLDIALGAGGLPMGRIVEIYGPESSGKTTLTLQV
5 Cronobacter    100.0%  92.9%    MAIDENKQKALAAALGQIEKQFGKGSIMRLGEDRTMDVETISTGSLSLDIALGAGGLPMGRIVEIYGPESSGKTTLTLQV
6 Enterobacter    99.4%  94.4%    MAIDENKQKALAAALGQIEKQFGKGSIMRLGEDRTMDVETISTGSLSLDIALGAGGLPMGRIVEIYGPESSGKTTLTLQV
7 Phytobacter    100.0%  96.6%    MAIDENKQKALAAALGQIEKQFGKGSIMRLGEDRSMDVETISTGSLSLDIALGAGGLPMGRIVEIYGPESSGKTTLTLQV
8 Kosakonia      100.0%  94.9%    MAIDENKQKALAAALGQIEKQFGKGSIMRLGEDRSMDVETISTGSLSLDIALGAGGLPMGRIVEIYGPESSGKTTLTLQV
9 Lelliottia      99.4%  93.5%    MAIDENKQKALAAALGQIEKQFGKGSIMRLGEDRSMDVETISTGSLSLDIALGAGGLPMGRIVEIYGPESSGKTTLTLQA
  consensus/100%                  MAIDENKQKALAAALGQIEKQFGKGSIMRLGEDRoMDVETISTGSLSLDIALGAGGLPMGRIVEIYGPESSGKTTLTLQs
  consensus/90%                   MAIDENKQKALAAALGQIEKQFGKGSIMRLGEDRoMDVETISTGSLSLDIALGAGGLPMGRIVEIYGPESSGKTTLTLQs
  consensus/80%                   MAIDENKQKALAAALGQIEKQFGKGSIMRLGEDRoMDVETISTGSLSLDIALGAGGLPMGRIVEIYGPESSGKTTLTLQV
  consensus/70%                   MAIDENKQKALAAALGQIEKQFGKGSIMRLGEDRSMDVETISTGSLSLDIALGAGGLPMGRIVEIYGPESSGKTTLTLQV

                   cov    pid  81          .         1         .         .         .         .         :         . 160
1 Salmonella     100.0% 100.0%    IAAAQREGKTCAFIDAEHALDPVYARKLGVDIDNLLCSQPDTGEQALEICDALARSGAVDVIVVDSVAALTPKAEIEGEI
2 Escherichia    100.0%  97.2%    IAAAQREGKTCAFIDAEHALDPIYARKLGVDIDNLLCSQPDTGEQALEICDALARSGAVDVIVVDSVAALTPKAEIEGEI
3 Citrobacter    100.0%  98.3%    IAAAQREGKTCAFIDAEHALDPVYARKLGVDIDNLLCSQPDTGEQALEICDALARSGAVDVIVVDSVAALTPKAEIEGEI
4 Klebsiella      99.4%  95.2%    IAAAQREGKTCAFIDAEHALDPVYARKLGVDIDNLLCSQPDTGEQALEICDALARSGAVDVIVVDSVAALTPKAEIEGEI
5 Cronobacter    100.0%  92.9%    IAAAQRAGKTCAFIDAEHALDPVYARKLGVDIDNLLCSQPDTGEQALEICDALARSGAVDVLVVDSVAALTPKAEIEGEI
6 Enterobacter    99.4%  94.4%    IAAAQREGKTCAFIDAEHALDPVYARKLGVDIDNLLCSQPDTGEQALEICDALARSGAVDVIVVDSVAALTPKAEIEGEI
7 Phytobacter    100.0%  96.6%    IAAAQREGKTCAFIDAEHALDPVYARKLGVDIDNLLCSQPDTGEQALEICDALARSGAVDVIVVDSVAALTPKAEIEGEI
8 Kosakonia      100.0%  94.9%    IAAAQREGKTCAFIDAEHALDPVYARKLGVDIDNLLCSQPDTGEQALEICDALARSGAVDVLVVDSVAALTPKAEIEGEI
9 Lelliottia      99.4%  93.5%    IAAAQRQGKTCAFIDAEHALDPIYARKLGVDIDNLLCSQPDTGEQALEICDALARSGAVDVIVVDSVAALTPKAEIEGEI
  consensus/100%                  IAAAQRtGKTCAFIDAEHALDPlYARKLGVDIDNLLCSQPDTGEQALEICDALARSGAVDVlVVDSVAALTPKAEIEGEI
  consensus/90%                   IAAAQRtGKTCAFIDAEHALDPlYARKLGVDIDNLLCSQPDTGEQALEICDALARSGAVDVlVVDSVAALTPKAEIEGEI
  consensus/80%                   IAAAQRpGKTCAFIDAEHALDPlYARKLGVDIDNLLCSQPDTGEQALEICDALARSGAVDVlVVDSVAALTPKAEIEGEI
  consensus/70%                   IAAAQREGKTCAFIDAEHALDPVYARKLGVDIDNLLCSQPDTGEQALEICDALARSGAVDVIVVDSVAALTPKAEIEGEI

                   cov    pid 161          .         .         .         2         .         .         .         . 240
1 Salmonella     100.0% 100.0%    GDSHMGLAARMMSQAMRKLAGNLKQSNTLLIFINQIRMKIGVMFGNPETTTGGNALKFYASVRLDIRRIGAVKEGDNVVG
2 Escherichia    100.0%  97.2%    GDSHMGLAARMMSQAMRKLAGNLKQSNTLLIFINQIRMKIGVMFGNPETTTGGNALKFYASVRLDIRRIGAVKEGENVVG
3 Citrobacter    100.0%  98.3%    GDSHMGLAARMMSQAMRKLAGNLKQSNTLLIFINQIRMKIGVMFGNPETTTGGNALKFYASVRLDIRRIGAVKEGDNVVG
4 Klebsiella      99.4%  95.2%    GDSHMGLAARMMSQAMRKLAGNLKQSNTLLIFINQIRMKIGVMFGNPETTTGGNALKFYASVRLDIRRIGAVKEGDNVVG
5 Cronobacter    100.0%  92.9%    GDSHMGLAARMMSQAMRKLAGNLKNSNTLLIFINQIRMKIGVMFGNPETTTGGNALKFYASVRLDIRRIGAVKEGEEVVG
6 Enterobacter    99.4%  94.4%    GDSHMGLAARMMSQAMRKLAGNLKQSNTLLIFINQIRMKIGVMFGNPETTTGGNALKFYASVRLDIRRIGAVKEGENVVG
7 Phytobacter    100.0%  96.6%    GDSHMGLAARMMSQAMRKLAGNLKQSNTLLIFINQIRMKIGVMFGNPETTTGGNALKFYASVRLDIRRVGAVKEGDNVVG
8 Kosakonia      100.0%  94.9%    GDSHMGLAARMMSQAMRKLAGNLKQSNTLLIFINQIRMKIGVMFGNPETTTGGNALKFYASVRLDIRRIGAVKEGDNVVG
9 Lelliottia      99.4%  93.5%    GDSHMGLAARMMSQAMRKLAGNLKQSNTLLIFINQIRMKIGVMFGNPETTTGGNALKFYASVRLDIRRIGAVKEGENVVG
  consensus/100%                  GDSHMGLAARMMSQAMRKLAGNLKpSNTLLIFINQIRMKIGVMFGNPETTTGGNALKFYASVRLDIRRlGAVKEG-pVVG
  consensus/90%                   GDSHMGLAARMMSQAMRKLAGNLKpSNTLLIFINQIRMKIGVMFGNPETTTGGNALKFYASVRLDIRRlGAVKEG-pVVG
  consensus/80%                   GDSHMGLAARMMSQAMRKLAGNLKQSNTLLIFINQIRMKIGVMFGNPETTTGGNALKFYASVRLDIRRIGAVKEG-NVVG
  consensus/70%                   GDSHMGLAARMMSQAMRKLAGNLKQSNTLLIFINQIRMKIGVMFGNPETTTGGNALKFYASVRLDIRRIGAVKEG-NVVG
```

1. RecA (part 2)

```
                       cov     pid 241          :         .         .         .         .         3         .         . 320
 1 Salmonella        100.0% 100.0%     SETRVKVVKNKIAAPFKQAEFQILYGEGINFYGELVDLGVKEKLIEKAGAWYSYNGEKIGQGKANATTWLKENPATAKEI
 2 Escherichia       100.0%  97.2%     SETRVKVVKNKIAAPFKQAEFQILYGEGINFYGELVDLGVKEKLIEKAGAWYSYKGEKIGQGKANATAWLKDNPETAKEI
 3 Citrobacter       100.0%  98.3%     SETRVKVVKNKIAAPFKQAEFQILYGEGINFYGELVDLGVKEKLIEKAGAWYSYNGEKIGQGKANATTWLKENPATAKEI
 4 Klebsiella         99.4%  95.2%     SETRVKVVKNKIAAPFKQAEFQILYGEGINFFGELVDLGVKEKLIEKAGAWYSYNGDKIGQGKANAITWLKENPAAAKEI
 5 Cronobacter       100.0%  92.9%     SETRVKVVKNKVAAPFKQAEFQILYGEGINFYGELVDLGVKHKLIEKAGAWYSYNGEKIGQGKANATNFLKENKPMADEI
 6 Enterobacter       99.4%  94.4%     SETRVKVVKNKIAAPFKQAEFQILYGEGINFFGELVDLGVKEKLIEKAGAWYSYNGDKIGQGKANAISWLKENPAAAKEI
 7 Phytobacter       100.0%  96.6%     SETRVKVVKNKIAAPFKQAEFQILYGEGINFYGELVDLGVKEKLIEKAGAWYSYNGEKIGQGKANATNWLKENPATAKEI
 8 Kosakonia         100.0%  94.9%     SETRVKVVKNKIAAPFKQAEFQILYGEGINFYGELVDLGVKEKLIEKAGAWYSYNGDKIGQGKANATTWLKENPVAAKEI
 9 Lelliottia         99.4%  93.5%     SETRVKVVKNKIAAPFKQAEFQILYGEGINFLGELVDLGVKEKLIEKAGAWYSYNGDKIGQGKANAISWLKENPAAAKEI
   consensus/100%                      SETRVKVVKNKlAAPFKQAEFQILYGEGINFhGELVDLGVKcKLIEKAGAWYSYpG-KIGQGKANAhsaLK-N..hAcEI
   consensus/90%                       SETRVKVVKNKlAAPFKQAEFQILYGEGINFhGELVDLGVKcKLIEKAGAWYSYpG-KIGQGKANAhsaLK-N..hAcEI
   consensus/80%                       SETRVKVVKNKIAAPFKQAEFQILYGEGINFaGELVDLGVKEKLIEKAGAWYSYNG-KIGQGKANAhsWLKENPssAKEI
   consensus/70%                       SETRVKVVKNKIAAPFKQAEFQILYGEGINFaGELVDLGVKEKLIEKAGAWYSYNG-KIGQGKANAhsWLKENPssAKEI
```

```
                       cov     pid 321          .         .         :   ] 354
 1 Salmonella        100.0% 100.0%     EKRVRELLLSNQNATPDFAVDDS-EGVAETNEDF
 2 Escherichia       100.0%  97.2%     EKKVRELLLSNPNSTPDFSVDDS-EGVAETNEDF
 3 Citrobacter       100.0%  98.3%     EKKVRELLLNNQDSTPDFSVDDG-EGVAETNEDF
 4 Klebsiella         99.4%  95.2%     EKKVRELLLNNQDSTPDFAVDGNDA--EETEQDF
 5 Cronobacter       100.0%  92.9%     EKKLREMLLNNQDATPDFTVDDNDGGVEETNEEF
 6 Enterobacter       99.4%  94.4%     EKKVRELLLNNQDSKPDFVVDGADA--EETNEDF
 7 Phytobacter       100.0%  96.6%     EKKVREILLSNPSSGADFSVDDSGEGVEETNEDF
 8 Kosakonia         100.0%  94.9%     EKKLREILLSNQNSSAEFTADGNDEDVEETNEDF
 9 Lelliottia         99.4%  93.5%     EKKVRELLLSNPDSKPDFVVDAADA--SESNEDF
   consensus/100%                      EK+lREhLLsN.suts-FssDss.t..tEopp-F
   consensus/90%                       EK+lREhLLsN.suts-FssDss.t..tEopp-F
   consensus/80%                       EKKlRElLLsN.supsDFsVDss.t..tETNEDF
   consensus/70%                       EKKVRElLLsN.sSsPDFsVDss.t..tETNEDF
```

```
#  Percent Identity  Matrix - created by Clustal2.1
#
#

     1: Salmonella     100.00   97.17   98.30   96.01   93.20   95.16   96.88   95.18   94.30
     2: Escherichia     97.17  100.00   97.17   94.30   92.35   94.59   96.60   94.05   94.87
     3: Citrobacter     98.30   97.17  100.00   96.87   93.77   96.30   97.17   95.18   94.87
     4: Klebsiella      96.01   94.30   96.87  100.00   92.61   97.73   94.60   95.45   95.45
     5: Cronobacter     93.20   92.35   93.77   92.61  100.00   93.18   92.37   92.66   91.19
     6: Enterobacter    95.16   94.59   96.30   97.73   93.18  100.00   94.60   94.89   97.16
     7: Phytobacter     96.88   96.60   97.17   94.60   92.37   94.60  100.00   95.20   94.03
     8: Kosakonia       95.18   94.05   95.18   95.45   92.66   94.89   95.20  100.00   93.47
     9: Lelliottia      94.30   94.87   94.87   95.45   91.19   97.16   94.03   93.47  100.00
```

2. MutS

```
                          cov     pid   1 [         .         .         .         .         :         .         .         . 80
1 Salmonella           100.0% 100.0%    MNESFDKDFSNHTPMMQQYLKLKAQHPEILLFYRMGDFYELFYDDAKRASQLLDISLTKRGASAGEPIPMAGIPHHAVEN
2 Escherichia           99.8%  94.5%    --MSAIENFDAHTPMMQQYLRLKAQHPEILLFYRMGDFYELFYDDAKRASQLLDISLTKRGASAGEPIPMAGIPYHAVEN
3 Citrobacter           99.8%  95.4%    --MSTLENLDAHTPMMQQYLKLKAQHPEILLFYRMGDFYELFYDDAKRASQLLDISLTKRGASAGEPIPMAGIPHHAVEN
4 Klebsiella            99.8%  93.3%    --MSTIDNLDAHTPMMQQYLKLKAQHPDILLFYRMGDFYELFYDDAKRASQLLDISLTKRGASAGEPIPMAGIPHHAVEN
5 Cronobacter           99.8%  90.2%    --MSTSETFDAHTPMMQQYLKLKAQHPDILLFYRMGDFYELFYDDAKRASQLLDISLTKRGASAGEPIPMAGVPHHAVEN
6 Enterobacter          99.8%  94.1%    --MSTLENFDAHTPMMQQYLKLKAQHPEILLFYRMGDFYELFYDDAKRASQLLDISLTKRGASAGEPIPMAGIPHHAVEN
7 Phytobacter           99.8%  93.0%    --MSTLENFDAHTPMMQQYLKLKAQHPDILLFYRMGDFYELFYDDAKRASQLMDISLTKRGASAGEPIPMAGVPHHAVEN
8 Kosakonia             99.8%  91.9%    --MSTLENFDAHTPMMQQYLKLKAQHPDILLFYRMGDFYELFYDDAKRASQLMDISLTKRGASAGEPIPMAGVPHHAVEN
9 Lelliottia            99.8%  92.6%    --MSTIDNLTAHTPMMQQYLKLKAQHPEILLFYRMGDFYELFYDDAKRASQLLDISLTKRGASAGEPIPMAGIPHHAVEN
  consensus/100%                        ...Sh.cshssHTPMMQQYL+LKAQHP-ILLFYRMGDFYELFYDDAKRASQLhDISLTKRGASAGEPIPMAGlPaHAVEN
  consensus/90%                         ...Sh.cshssHTPMMQQYL+LKAQHP-ILLFYRMGDFYELFYDDAKRASQLhDISLTKRGASAGEPIPMAGlPaHAVEN
  consensus/80%                         ..MSs.-shsAHTPMMQQYLKLKAQHP-ILLFYRMGDFYELFYDDAKRASQLhDISLTKRGASAGEPIPMAGlPHHAVEN
  consensus/70%                         ..MSTl-NhDAHTPMMQQYLKLKAQHP-ILLFYRMGDFYELFYDDAKRASQLLDISLTKRGASAGEPIPMAGlPHHAVEN

                          cov     pid  81         .         1         .         .         .         .         :         . 160
1 Salmonella           100.0% 100.0%    YLAKLVNQGESVAICEQIGDPATSKGPVERKVVRIVTPGTISDEALLQERQDNLLAAIWQDGKGYGYATLDISSGRFRLS
2 Escherichia           99.8%  94.5%    YLAKLVNQGESVAICEQIGDPATSKGPVERKVVRIVTPGTISDEALLQERQDNLLAAIWQDSKGFGYATLDISSGRFRLS
3 Citrobacter           99.8%  95.4%    YLAKLVNQGESVAICEQIGDPATSKGPVERKVVRIVTPGTISDEALLQERQDNLLAAIWKDSKGYGYATLDISSGRFRLS
4 Klebsiella            99.8%  93.3%    YLAKLVNQGESVAICEQIGDPATTKGPVERKVVRIVTPGTISDEALLQERQDNLLAAIWQDSKGFGYATLDISSGRFRLS
5 Cronobacter           99.8%  90.2%    YLAKLVNLGESVAICEQIGDPATSKGPVERKVVRIVTPGTISDEALLQERQDNLLAAIWQDGKGFGYATLDISSGRFRLT
6 Enterobacter          99.8%  94.1%    YLAKLVNQGESVAICEQIGDPATSKGPVERKVVRIVTPGTISDEALLQERQDNLLAALWQDGKGFGYATLDISSGRFRLS
7 Phytobacter           99.8%  93.0%    YLAKLVSLGESVAICEQIGDPATSKGPVERKVVRIVTPGTISDEALLQERQDNLLAAIWQDSKGFGYATLDISSGRFRVS
8 Kosakonia             99.8%  91.9%    YLAKLVSLGESVAICEQIGDPATSKGPVERKVVRIVTPGTISDEALLQERQDNLLAAVWQDSKGFGYATLDISSGRFRVS
9 Lelliottia            99.8%  92.6%    YLAKLVNQGESVAICEQIGDPATSKGPVERKVVRIVTPGTISDEALLHERQDNLLAAVWQDSKGFGYATLDISSGRFRLS
  consensus/100%                        YLAKLVs.GESVAICEQIGDPATokGPVERKVVRIVTPGTISDEALLpERQDNLLAAlWpDuKGaGYATLDISSGRFRlo
  consensus/90%                         YLAKLVs.GESVAICEQIGDPATokGPVERKVVRIVTPGTISDEALLpERQDNLLAAlWpDuKGaGYATLDISSGRFRlo
  consensus/80%                         YLAKLVs.GESVAICEQIGDPATSKGPVERKVVRIVTPGTISDEALLQERQDNLLAAlWQDuKGaGYATLDISSGRFRlS
  consensus/70%                         YLAKLVN.GESVAICEQIGDPATSKGPVERKVVRIVTPGTISDEALLQERQDNLLAAlWQDuKGFGYATLDISSGRFRLS

                          cov     pid 161         .         .         .         2         .         .         .         . 240
1 Salmonella           100.0% 100.0%    EPADRETMAAELQRTNPAELLYAEDFAEMALIEGRRGLRRRPLWEFEIDTARQQLNLQFGTRDLVGFGVENASRGLCAAG
2 Escherichia           99.8%  94.5%    EPADRETMAAELQRTNPAELLYAEDFAEMSLIEGRRGLRRRPLWEFEIDTARQQLNLQFGTRDLVGFGVENAPRGLCAAG
3 Citrobacter           99.8%  95.4%    EPADRETMAAELQRTNPAELLYAEDFSEMALIEGRRGLRRRPLWEFEIDTARQQLNLQFGTRDLVGFGVENAPRGLCAAG
4 Klebsiella            99.8%  93.3%    EPADRETMAAELQRTNPAELLYAEDFAESSLIEGRRGLRRRPLWEFEIDTARQQLNLQFGTRDLVGFGVENAPRGLCAAG
5 Cronobacter           99.8%  90.2%    EPQDRETMAAELQRTNPAELLYAEDFAEMALIEGRRGLRRRPLWEFELDTARQQLNLQFGTRDLVGFGVENAPRGLCAAG
6 Enterobacter          99.8%  94.1%    EPADRETMAAELQRTNPAELLYAEDFAEMALIEGRRGLRRRPLWEFEIDTARQQLNLQFGTRDLIGFGVENAPRGLCAAG
7 Phytobacter           99.8%  93.0%    EPADRETMAAELQRTNPAELLYAEDFAETSLIEGRRGLRRRPLWEFEIDTARQQLNLQFGTRDLVGFGVENAPRGLCAAG
8 Kosakonia             99.8%  91.9%    EPTDRDTMAAELQRTNPAELLYAEDFAETALIEGRRGLRRRPLWEFEIDTARQQLNMQFGTRDLVGFGVENAPRGLCAAG
9 Lelliottia            99.8%  92.6%    EPADRETMAAELQRTNPAELLYAEDFAEFALIEGRRGLRRRPLWEFEIDTARQQLNLQFGTRDLIGFGVENAPRGLCAAG
  consensus/100%                        EPtDR-TMAAELQRTNPAELLYAEDFuE.uLIEGRRGLRRRPLWEFElDTARQQLNhQFGTRDLlGFGVENAsRGLCAAG
  consensus/90%                         EPtDR-TMAAELQRTNPAELLYAEDFuE.uLIEGRRGLRRRPLWEFElDTARQQLNhQFGTRDLlGFGVENAsRGLCAAG
  consensus/80%                         EPsDRETMAAELQRTNPAELLYAEDFAEhuLIEGRRGLRRRPLWEFEIDTARQQLNLQFGTRDLlGFGVENAPRGLCAAG
  consensus/70%                         EPADRETMAAELQRTNPAELLYAEDFAEhuLIEGRRGLRRRPLWEFEIDTARQQLNLQFGTRDLVGFGVENAPRGLCAAG

                          cov     pid 241         :         .         .         .         .         3         .         . 320
1 Salmonella           100.0% 100.0%    CLLQYVKDTQRTSLPHIRSITMERQQDSIIMDAATRRNLEITQNLAGGVENTLAAVLDCTVTPMGSRMLKRWLHMPVRNT
2 Escherichia           99.8%  94.5%    CLLQYAKDTQRTTLPHIRSITMEREQDSIIMDAATRRNLEITQNLAGGAENTLASVLDCTVTPMGSRMLKRWLHMPVRDT
3 Citrobacter           99.8%  95.4%    CLLQYVKDTQRTSLPHIRSITMERQQDSIIMDAATRRNLEITQNLAGGVENTLASVLDCTVTPMGSRMLKRWLHMPVRDT
4 Klebsiella            99.8%  93.3%    CLLQYVKDTQRTSLPHIRSITMERQQDSIIMDAATRRNLEITQNLAGGTDNTLASVLDCTVTPMGSRMLKRWLHMPVRDT
5 Cronobacter           99.8%  90.2%    CLLQYVKDTQRTSLPHIRSITMERQQDGIIMDAATRRNLEITQNLAGGVENTLASVLDCTVTPMGSRMLKRWLHMPVRDA
6 Enterobacter          99.8%  94.1%    CLLQYVKDTQRTALPHIRSITMERQQDSIIMDAATRRNLEITQNLAGGVENTLASVLDNTVTPMGSRMLKRWLHMPVRDT
7 Phytobacter           99.8%  93.0%    CLLQYVKDTQRTALPHIRSITMERQQDSIIMDAATRRNLEITQNLAGGIENTLASVLDCTVTPMGSRMLKRWLHMPVRDT
8 Kosakonia             99.8%  91.9%    CLLQYVKDTQRTALPHIRSITMERQQDTIIMDAATRRNLEITQNLAGGVENTLSSVLDCTVTPMGSRMLKRWLHMPIRDT
9 Lelliottia            99.8%  92.6%    CLLQYVKDTQRTALPHIRSITMERQQDSIIMDAATRRNLEITQNLAGGIDNTLASVLDSTVTPMGSRMLKRWLHMPVRDT
  consensus/100%                        CLLQYsKDTQRTsLPHIRSITMERpQDsIIMDAATRRNLEITQNLAGGh-NTLuuVLDsTVTPMGSRMLKRWLHMPlRss
  consensus/90%                         CLLQYsKDTQRTsLPHIRSITMERpQDsIIMDAATRRNLEITQNLAGGh-NTLuuVLDsTVTPMGSRMLKRWLHMPlRss
  consensus/80%                         CLLQYVKDTQRTuLPHIRSITMERQQDoIIMDAATRRNLEITQNLAGGh-NTLASVLDsTVTPMGSRMLKRWLHMPVRDT
  consensus/70%                         CLLQYVKDTQRTuLPHIRSITMERQQDSIIMDAATRRNLEITQNLAGGlENTLASVLDCTVTPMGSRMLKRWLHMPVRDT
```

2. MutS (part 2)

```
                        cov     pid 321        .         .         :         .         .         .         .        4 400
1 Salmonella         100.0% 100.0%  DILRERQQTIGALQDTVSELQPVLRQVGDLERILARLALRTARPRDLARMRHAFQQLPELHAQLETVDSAPVQALRKKMG
2 Escherichia         99.8%  94.5%  RVLLERQQTIGALQDFTAGLQPVLRQVGDLERILARLALRTARPRDLARMRHAFQQLPELRAQLETVDSAPVQALREKMG
3 Citrobacter         99.8%  95.4%  RILTERQQTIGALQDLTAELQPVLRQVGDLERILARLALRTARPRDLARMRHAFQQLPELHAQLEGVDSAPVQMLREKMG
4 Klebsiella          99.8%  93.3%  AVLVERQQTIGALQERYTELQPVLRQVGDLERILARLALRTARPRDLARMRHALQQLPLLRELLADVDRQPVQKLREKMG
5 Cronobacter         99.8%  90.2%  SVLRHRQQAIAALMEYSTDIQPVLRQVGDLERILARLALRTARPRDLARMRHAFQQLPTLNTLLTDIDAGYVQTLREQMG
6 Enterobacter        99.8%  94.1%  DTLVCRQQTIAALQDRYTELQPVLRQVGDLERILARLALRTARPRDLARMRHAFQQLPELRAQLSDVDSAPVQKLRETMG
7 Phytobacter         99.8%  93.0%  KTLLERQQTIGALQENTGELQPVLRQVGDLERILARLALRTARPRDLARMRHAFQQLPELRAQLAQVKSEPVQNLREKMG
8 Kosakonia           99.8%  91.9%  KVLTERQQTISALQEHTSELQPVLRQVGDLERILARLALRTARPRDLARMRYAFQQLPELRSQLADIDSAPVQALREKMG
9 Lelliottia          99.8%  92.6%  QVLVSRQQTIGALQDRFTELQPVLRQVGDLERILARLALRTARPRDLARMRHAFQQLPELRAQLAEVDSAPVQKLRETMG
  consensus/100%                    thLhpRQQsIuAL.-..stlQPVLRQVGDLERILARLALRTARPRDLARMRaAhQQLP.Lpt.Lttlctt.VQ.LRcpMG
  consensus/90%                     thLhpRQQsIuAL.-..stlQPVLRQVGDLERILARLALRTARPRDLARMRaAhQQLP.Lpt.Lttlctt.VQ.LRcpMG
  consensus/80%                     phLhpRQQTIuALQ-hhs-LQPVLRQVGDLERILARLALRTARPRDLARMRHAFQQLPpL+s.LtplDutPVQtLREpMG
  consensus/70%                     plLhcRQQTIuALQ-hhsELQPVLRQVGDLERILARLALRTARPRDLARMRHAFQQLPEL+uQLtsVDSuPVQtLREpMG

                        cov     pid 401        .         .         .         .         :         .         .        . 480
1 Salmonella         100.0% 100.0%  DFAELRDLLERAIIDAPPVLVRDGGVIAPGYHEELDEWRALADGATDYLDRLEIRERERTGLDTLKVGYNAVHGYYIQIS
2 Escherichia         99.8%  94.5%  EFAELRDLLERAIIDTPPVLVRDGGVIASGYNEELDEWRALADGATDYLERLEVRERERTGLDTLKVGFNAVHGYYIQIS
3 Citrobacter         99.8%  95.4%  EFTELRELLERAIIDTPPVLVRDGGVIAPGYNEELDEWRALADGATDYLDKLEIRERERTGLDTLKVGFNAVHGYYIQIS
4 Klebsiella          99.8%  93.3%  EFTELRELLERSVIDAPPVLVRDGGVIAPGYSEELDEWRALADGATDYLDKLEIRERERLGLDTLKVGYNAVHGYYIQIS
5 Cronobacter         99.8%  90.2%  EFTELRDLLERAIIEAPPVLVRDGGVIAPGYHAELDEWRALADGATDYLDRLEIREREKLGIDTLKVGFNAVHGYFIQVS
6 Enterobacter        99.8%  94.1%  EFAELRELLERAIIDAPPVLVRDGGVIAPGYNEELDEWRALADGATDYLDKLEIRERERLGLDTLKVGYNAVHGYYIQIS
7 Phytobacter         99.8%  93.0%  EFTELRELLERAIIDAPPVLVRDGGVIAPGYNAELDEWRALADGATDYLDRLEIREREKLGLDTLKVGYNAVHGYYIQIS
8 Kosakonia           99.8%  91.9%  EFVELRELLERAIIEAPPVLVRDGGVIAPGYNAELDEWRGLADGATDYLDRLEVRERERLGLDTLKVGYNAVHGYFIQIS
9 Lelliottia          99.8%  92.6%  EFTELRELLERAVVEAPPVLVRDGGVIAPGYHQELDEWRALADGATDYLDKLEIRERERLGLDTLKVGYNAIHGYYIQIS
  consensus/100%                    -FsELR-LLERull-sPPVLVRDGGVIAsGYptELDEWRuLADGATDYL-+LElRERE+hGlDTLKVGaNAlHGYaIQlS
  consensus/90%                     -FsELR-LLERull-sPPVLVRDGGVIAsGYptELDEWRuLADGATDYL-+LElRERE+hGlDTLKVGaNAlHGYaIQlS
  consensus/80%                     EFsELR-LLERAlI-sPPVLVRDGGVIAPGYptELDEWRALADGATDYLD+LElRERE+hGLDTLKVGaNAVHGYaIQIS
  consensus/70%                     EFsELR-LLERAII-APPVLVRDGGVIAPGYptELDEWRALADGATDYLD+LEIRERERhGLDTLKVGaNAVHGYYIQIS

                        cov     pid 481        .         5         .         .         .         .         :        . 560
1 Salmonella         100.0% 100.0%  RGQSHLAPINYVRRQTLKNAERYIIPELKEYEDKVLTSKGKALALEKQLYDELFDLLLPHLADLQQSANALAELDVLVNL
2 Escherichia         99.8%  94.5%  RGQSHLAPINYMRRQTLKNAERYIIPELKEYEDKVLTSKGKALALEKQLYEELFDLLLPHLEALQQSASALAELDVLVNL
3 Citrobacter         99.8%  95.4%  RGQSHLAPINYTRRQTLKNAERYIIPELKEYEDKVLTSKGKALALEKQLYDELFDLLLPHLADLQQSASALAEIDVLVNL
4 Klebsiella          99.8%  93.3%  RGQSHLAPIHYVRRQTLKNAERYIIPELKEYEDKVLTSKGKALALEKQLYDELFDLLLPHLADLQTSASALAELDVLVNL
5 Cronobacter         99.8%  90.2%  RGQSHMVPIHYVRRQTLKNAERYIIPELKEYEDKVLTSKGKALALEKQLYDELFDLLLPHLAELQKSAAALAELDVLTNL
6 Enterobacter        99.8%  94.1%  RGQSHLAPIHYVRRQTLKNAERYIIPELKEYEDKVLTSKGKALALEKQLYDELFDMLMPHLADLQLSAGALAELDVLVNL
7 Phytobacter         99.8%  93.0%  RGQSHLAPIHYVRRQTLKNAERYIIPELKEYEDKVLTSKGKALALEKQLYDELFDKLLPHLGDLQQSATALAELDVLVNL
8 Kosakonia           99.8%  91.9%  RGQSHLAPIHYVRRQTLKNAERYIIPELKEYEDKVLTSKGKALALEKQLYEELFDLLMPHLADLQQSAAALAELDVLVNL
9 Lelliottia          99.8%  92.6%  RGQSHLAPIHYVRRQTLKNAERYIIPELKEYEDKVLSSRGKALALEKQLYEELFDLLMPHLGDLQQSASALAELDVLVNL
  consensus/100%                    RGQSHhsPIpYhRRQTLKNAERYIIPELKEYEDKVLoS+GKALALEKQLY-ELFDhLhPHLttLQ.SAsALAElDVLsNL
  consensus/90%                     RGQSHhsPIpYhRRQTLKNAERYIIPELKEYEDKVLoS+GKALALEKQLY-ELFDhLhPHLttLQ.SAsALAElDVLsNL
  consensus/80%                     RGQSHLAPIpYsRRQTLKNAERYIIPELKEYEDKVLTSKGKALALEKQLY-ELFDhLhPHLu-LQpSAsALAELDVLVNL
  consensus/70%                     RGQSHLAPIpYVRRQTLKNAERYIIPELKEYEDKVLTSKGKALALEKQLY-ELFDLLhPHLuDLQpSAuALAELDVLVNL

                        cov     pid 561        .         .         .         6         .         .         .        . 640
1 Salmonella         100.0% 100.0%  AERAWTLNYTCPTFTDKPGIRITEGRHPVVEQVLNEPFIANPLNLSPQRRMLIITGPNMGGKSTYMRQTALIALLAYIGS
2 Escherichia         99.8%  94.5%  AERAYTLNYTCPTFIDKPGIRITEGRHPVVEQVLNEPFIANPLNLSPQRRMLIITGPNMGGKSTYMRQTALIALMAYIGS
3 Citrobacter         99.8%  95.4%  AERAYTLNYTCPIFTDKPGIRITEGRHPVVEQVLNEPFIANPLDLSPQRRMLIITGPNMGGKSTYMRQTALIALLAYIGS
4 Klebsiella          99.8%  93.3%  AERAETLNYCCPTFSDKPGIRISEGRHPVVEQVLKEPFIANPLQLAPQRRMLIITGPNMGGKSTYMRQTALIALLAYIGS
5 Cronobacter         99.8%  90.2%  AERADTLNYHCPTLTDKPGVRLVEGRHPVVERVLNEPFIANPLSLSPQRRMLIITGPNMGGKSTYMRQTALIVLMAYIGS
6 Enterobacter        99.8%  94.1%  AERAETLNYTCPTFTDKPGIRITEGRHPVVEQVLNEPFIANPLSLSPQRRMLIITGPNMGGKSTYMRQTALIALLAYIGS
7 Phytobacter         99.8%  93.0%  AERADTLNYCCPTFSDKPGIRIVEGRHPVVERVLNEPFIANPLNLSPQRRMLIITGPNMGGKSTYMRQTALIALLAYIGS
8 Kosakonia           99.8%  91.9%  AERAYTLNYCCPTFSEKPGIRISEGRHPVVEQVLKEPFIANPLNLSPQRRMLIITGPNMGGKSTYMRQTALIALLAYIGS
9 Lelliottia          99.8%  92.6%  AERADALNYTCPTFIDKPGIRITEGRHPVVEQVLREPFIANPLSLSPQRRMLIITGPNMGGKSTYMRQTALIALLAYIGS
  consensus/100%                    AERA.sLNYpCPhh.-KPGlRlsEGRHPVVEpVLpEPFIANPLpLuPQRRMLIITGPNMGGKSTYMRQTALIsLhAYIGS
  consensus/90%                     AERA.sLNYpCPhh.-KPGlRlsEGRHPVVEpVLpEPFIANPLpLuPQRRMLIITGPNMGGKSTYMRQTALIsLhAYIGS
  consensus/80%                     AERA.TLNYsCPTF.DKPGIRIsEGRHPVVEpVLpEPFIANPLsLSPQRRMLIITGPNMGGKSTYMRQTALIALhAYIGS
  consensus/70%                     AERA.TLNYsCPTFoDKPGIRIoEGRHPVVEQVLpEPFIANPLsLSPQRRMLIITGPNMGGKSTYMRQTALIALLAYIGS
```

3. MutS (part 3)

```
                   cov    pid 641         :         .         .         .         .         7         .         . 720
1 Salmonella     100.0% 100.0%     YVPAQNVEIGPIDRIFTRVGAADDLASGRSTFMVEMTETANILHNATENSLVLMDEIGRGTSTYDGLSLAWACAENLANK
2 Escherichia     99.8%  94.5%     YVPAQKVEIGPIDRIFTRVGAADDLASGRSTFMVEMTETANILHNATEYSLVLMDEIGRGTSTYDGLSLAWACAENLANK
3 Citrobacter     99.8%  95.4%     YVPAQKVEIGPIDRIFTRVGAADDLASGRSTFMVEMTETANILHNATDNSLVLMDEIGRGTSTYDGLSLAWACAENLANK
4 Klebsiella      99.8%  93.3%     YVPAQKVEIGPIDRIFTRVGAADDLASGRSTFMVEMTETANILHNATEHSLVLMDEIGRGTSTYDGLSLAWACAENLANK
5 Cronobacter     99.8%  90.2%     FVPAEQAEIGPIDRIFTRVGAADDLASGRSTFMVEMTETANILHNATEHSLVLMDEIGRGTSTYDGLSLAWACAESLANR
6 Enterobacter    99.8%  94.1%     YVPAQKVEIGPIDRIFTRVGAADDLASGRSTFMVEMTETANILHNATEHSLVLMDEVGRGTSTYDGLSLAWACAENLANK
7 Phytobacter     99.8%  93.0%     YVPAQKVEIGPIDRIFTRVGAADDLASGRSTFMVEMTETANILHNATENSLVLMDEIGRGTSTYDGLSLAWACAENLANK
8 Kosakonia       99.8%  91.9%     YVPAQKVEIGPIDRIFTRVGAADDLASGRSTFMVEMTETANILHNATENSLVLMDEIGRGTSTYDGLSLAWACAENLANK
9 Lelliottia      99.8%  92.6%     YVPAQQVEIGPIDRIFTRVGAADDLASGRSTFMVEMTETANILHNATENSLVLMDEIGRGTSTYDGLSLAWACAESLANK
  consensus/100%                   aVPAppsEIGPIDRIFTRVGAADDLASGRSTFMVEMTETANILHNAT-.SLVLMDElGRGTSTYDGLSLAWACAEsLAN+
  consensus/90%                    aVPAppsEIGPIDRIFTRVGAADDLASGRSTFMVEMTETANILHNAT-.SLVLMDElGRGTSTYDGLSLAWACAEsLAN+
  consensus/80%                    YVPAQpVEIGPIDRIFTRVGAADDLASGRSTFMVEMTETANILHNATEpSLVLMDEIGRGTSTYDGLSLAWACAEsLANK
  consensus/70%                    YVPAQpVEIGPIDRIFTRVGAADDLASGRSTFMVEMTETANILHNATEpSLVLMDEIGRGTSTYDGLSLAWACAENLANK

                   cov    pid 721         .         .         :         .         .         .         .         8 800
1 Salmonella     100.0% 100.0%     IKALTLFATHYFELTQLPEKMEGVANVHLDALEHGDTIAFMHSVQDGAASKSYGLAVAALAGVPKEVIKRARQKLRELES
2 Escherichia     99.8%  94.5%     IKALTLFATHYFELTQLPEKMEGVANVHLDALEHGDTIAFMHSVQDGAASKSYGLAVAALAGVPKEVIKRARQKLRELES
3 Citrobacter     99.8%  95.4%     IKALTLFATHYFELTQLPEKMEGVANVHLDALEHGDTIAFMHSVQDGAASKSYGLAVAALAGVPKEVIKRARQKLRELES
4 Klebsiella      99.8%  93.3%     IKALTLFATHYFELTQLPEKMEGVANVHLDALEHGDTIAFMHSVQDGAASKSYGLAVAALAGVPKEVIKRARQKLRELES
5 Cronobacter     99.8%  90.2%     IKALTLFATHYFELTQLPEKMEGVANVHLDAIEHGDTIAFMHSVQDGAASKSYGLAVAALAGVPKEVIKRARQKLRELES
6 Enterobacter    99.8%  94.1%     IKAMTLFATHYFELTQLPEKMEGVANVHLDALEHGDTIAFMHTVQEGAASKSYGLAVAALAGVPKEVIKRARQKLRELES
7 Phytobacter     99.8%  93.0%     IKALTLFATHYFELTQLPEKMEGVANVHLDALEHGDTIAFMHSVQDGAASKSYGLAVAALAGVPKEVIKRARGKLRELES
8 Kosakonia       99.8%  91.9%     IKALTLFATHYFELTQLPEKMEGVANVHLDALEHGDTIAFMHSVQDGAASKSYGLAVAALAGVPKEVIKRARGKLRELES
9 Lelliottia      99.8%  92.6%     IKALTLFATHYFELTQLPEKMEGVANVHLDALEHGDTIAFMHTVQDGAASKSYGLAVAALAGVPKEVIKRARQKLRELET
  consensus/100%                   IKAhTLFATHYFELTQLPEKMEGVANVHLDAlEHGDTIAFMHoVQ-GAASKSYGLAVAALAGVPKEVIKRARtKLRELEo
  consensus/90%                    IKAhTLFATHYFELTQLPEKMEGVANVHLDAlEHGDTIAFMHoVQ-GAASKSYGLAVAALAGVPKEVIKRARtKLRELEo
  consensus/80%                    IKALTLFATHYFELTQLPEKMEGVANVHLDALEHGDTIAFMHoVQDGAASKSYGLAVAALAGVPKEVIKRARtKLRELES
  consensus/70%                    IKALTLFATHYFELTQLPEKMEGVANVHLDALEHGDTIAFMHSVQDGAASKSYGLAVAALAGVPKEVIKRARQKLRELES

                   cov    pid 801         .         .         .         .         :    ] 855
1 Salmonella     100.0% 100.0%     ISPNAAATQVDGTQMSLLAAPEETSPAVEALENLDPDSLTPRQALEWIYRLKSLV
2 Escherichia     99.8%  94.5%     ISPNAAATQVDGTQMSLLSVPEETSPAVEALENLDPDSLTPRQALEWIYRLKSLV
3 Citrobacter     99.8%  95.4%     ISPNAAATQVDGTQMSLLSIPEETSPAVEALENLDPDSLTPRQALEWIYRLKSLV
4 Klebsiella      99.8%  93.3%     ISPNAAATQVDGTQMSLLAAPEETSPAVEALENLDPDSLTPRQALEWIYRLKSLV
5 Cronobacter     99.8%  90.2%     ISGNAAATQVDGTQMSLLVAAEETSPAIEALENLDPDSLSPRQALEWIYRLKSLV
6 Enterobacter    99.8%  94.1%     LSPNAAATQIDGTQMSLLAPAEETSPAVEALENLDPDSLTPRQALEWIYRLKSLV
7 Phytobacter     99.8%  93.0%     LSPSAGATQIDGTQMSLLAVPEETSPAMEALENLDPDSLTPRQALEWIYRLKSLV
8 Kosakonia       99.8%  91.9%     LSPSAAATQIDGTQMSLLAAPEETNPAVEALENLDPDSLTPRQALEWIYRLKNLL
9 Lelliottia      99.8%  92.6%     LSPNAAATQVDGTQMSLLAPAEETSPAVEALENLDPDSLTPRQALEWIYRLKSLV
  consensus/100%                   lSssAuATQlDGTQMSLLs.sEETsPAhEALENLDPDSLoPRQALEWIYRLKsLl
  consensus/90%                    lSssAuATQlDGTQMSLLs.sEETsPAhEALENLDPDSLoPRQALEWIYRLKsLl
  consensus/80%                    lSPsAAATQlDGTQMSLLussEETSPAlEALENLDPDSLTPRQALEWIYRLKSLV
  consensus/70%                    lSPNAAATQlDGTQMSLLussEETSPAVEALENLDPDSLTPRQALEWIYRLKSLV
```

```
#  Percent Identity  Matrix - created by Clustal2.1
#
#

     1: Salmonella     100.00   94.49   95.43   93.32   90.15   94.14   92.97   91.91   92.61
     2: Escherichia     94.49  100.00   95.55   93.20   89.45   93.32   93.32   92.03   92.50
     3: Citrobacter     95.43   95.55  100.00   94.14   89.92   94.49   93.67   92.38   93.20
     4: Klebsiella      93.32   93.20   94.14  100.00   90.50   94.26   93.90   92.73   94.02
     5: Cronobacter     90.15   89.45   89.92   90.50  100.00   90.39   90.39   89.57   89.21
     6: Enterobacter    94.14   93.32   94.49   94.26   90.39  100.00   93.79   92.73   94.84
     7: Phytobacter     92.97   93.32   93.67   93.90   90.39   93.79  100.00   94.96   92.73
     8: Kosakonia       91.91   92.03   92.38   92.73   89.57   92.73   94.96  100.00   91.79
     9: Lelliottia      92.61   92.50   93.20   94.02   89.21   94.84   92.73   91.79  100.00
```

3. DnaC

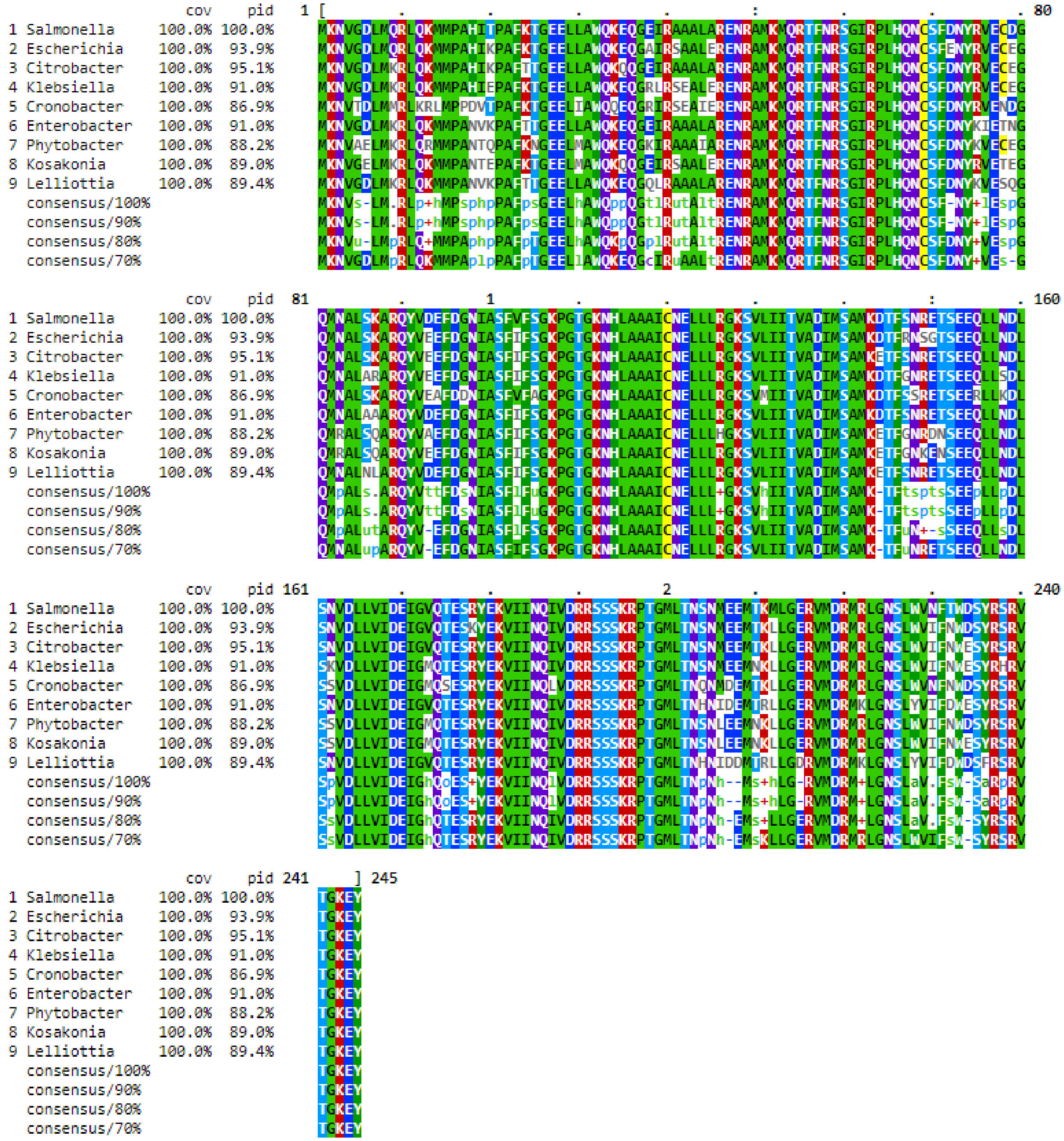


```
#  Percent Identity  Matrix - created by Clustal2.1
#
#

     1: Salmonella    100.00   93.88   95.10   91.02   86.94   91.02   88.16   88.98   89.39
     2: Escherichia    93.88  100.00   94.69   92.65   85.31   88.98   88.16   90.61   87.76
     3: Citrobacter    95.10   94.69  100.00   93.06   84.90   92.65   89.80   92.65   91.02
     4: Klebsiella     91.02   92.65   93.06  100.00   85.71   88.57   88.57   92.24   86.94
     5: Cronobacter    86.94   85.31   84.90   85.71  100.00   82.45   82.86   83.67   81.22
     6: Enterobacter   91.02   88.98   92.65   88.57   82.45  100.00   86.53   88.16   95.10
     7: Phytobacter    88.16   88.16   89.80   88.57   82.86   86.53  100.00   93.47   86.12
     8: Kosakonia      88.98   90.61   92.65   92.24   83.67   88.16   93.47  100.00   86.12
     9: Lelliottia     89.39   87.76   91.02   86.94   81.22   95.10   86.12   86.12  100.00
```

4. FtsA

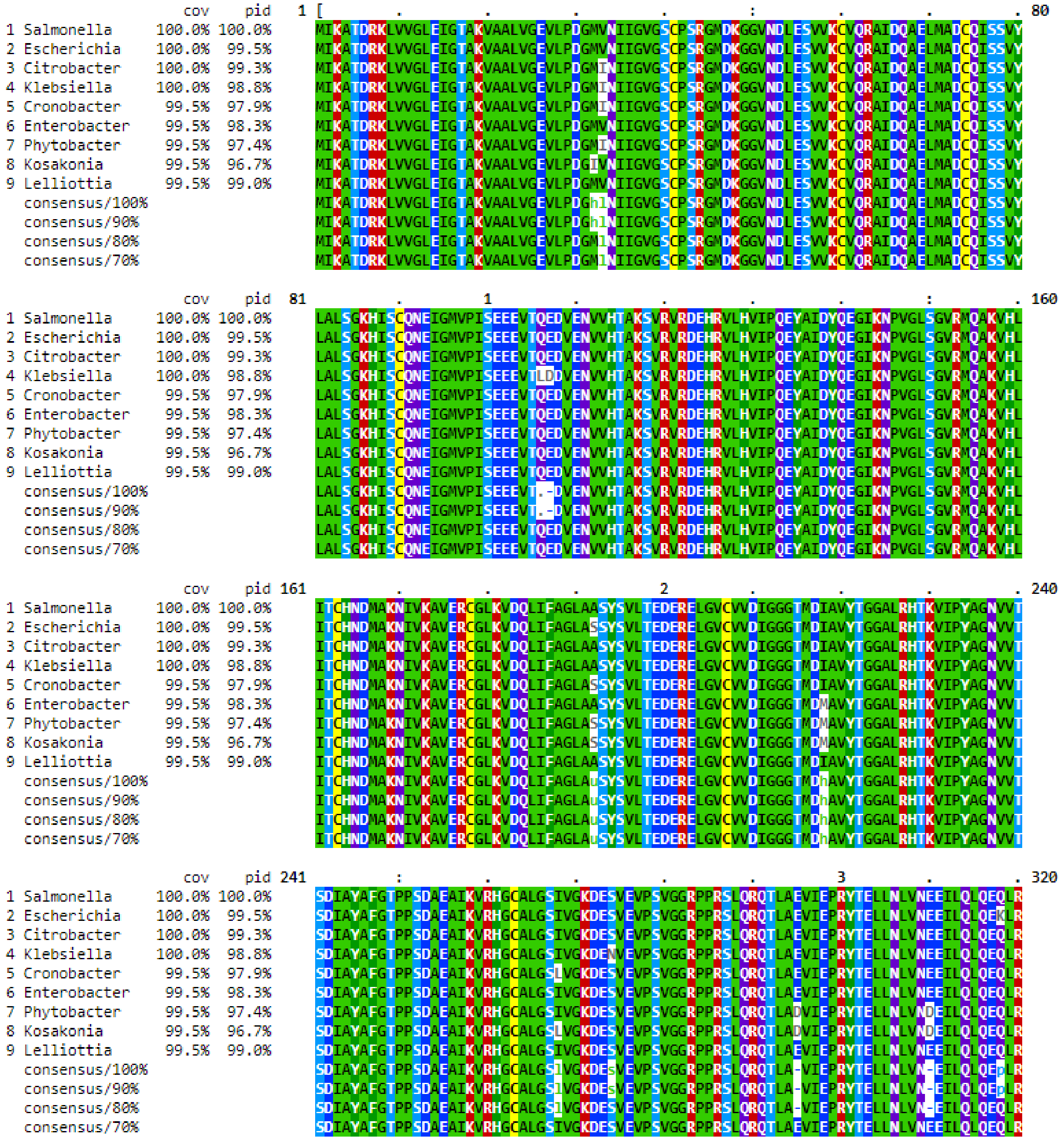
cov pid
1 Salmonella 100.0% 100.0%
2 Escherichia 100.0% 99.5%
3 Citrobacter 100.0% 99.3%
4 Klebsiella 100.0% 98.8%
5 Cronobacter 99.5% 97.9%
6 Enterobacter 99.5% 98.3%
7 Phytobacter 99.5% 97.4%
8 Kosakonia 99.5% 96.7%
9 Lelliottia 99.5% 99.0%
consensus/100%
consensus/90%
consensus/80%
consensus/70%
1 80
81 160
161 240
241 320

4. FtsA (part 2)

```
                    cov    pid 321         .         .         :         .         .         .         .       4 400
1 Salmonella      100.0% 100.0%    QQGVKHHLAAGIVLTGGAAQIEGLAACAQRVFHTQVRIGAPLNITGLTDYAQEPYYSTAVGLLHYGKESHLNGEAEVEKR
2 Escherichia     100.0%  99.5%    QQGVKHHLAAGIVLTGGAAQIEGLAACAQRVFHTQVRIGAPLNITGLTDYAQEPYYSTAVGLLHYGKESHLNGEAEVEKR
3 Citrobacter     100.0%  99.3%    QQGVKHHLAAGIVLTGGAAQIEGLAACAQRVFHTQVRIGAPLNITGLTDYAQEPYYSTAVGLLHYGKESHLSGEADVEKR
4 Klebsiella      100.0%  98.8%    QQGVKHHLAAGIVLTGGAAQIEGLAACAQRVFHTQVRIGAPLNITGLTDYAQEPYYSTAVGLLHYGKESHLSGEAEVEKR
5 Cronobacter      99.5%  97.9%    QQGVKHHLAAGIVLTGGAAQIEGLAACAQRVFHTQVRIGQPLNITGLTDYAQEPYYSTAVGLLHYGKESHLSGEAEVEKR
6 Enterobacter     99.5%  98.3%    QQGVKHHLAAGIVLTGGAAQIEGLAACAQRVFHTQVRIGAPLNITGLTDYAQEPYYSTAVGLLHYGKESHLSGEAEVEKR
7 Phytobacter      99.5%  97.4%    QQGVKHHLAAGIVLTGGAAQIEGLAACAQRVFHTQVRIGAPLNITGLTDYAQEPYYSTAVGLLHYGKETHLSGEAEVEKR
8 Kosakonia        99.5%  96.7%    QQGVKHHLAAGIVLTGGAAQMEGLAACAQRVFHTQVRIGAPLNITGLTDYAQEPYYSTAVGLLHYGKETHLSGEAEVEKR
9 Lelliottia       99.5%  99.0%    QQGVKHHLAAGIVLTGGAAQIEGLAACAQRVFHTQVRIGAPLNITGLTDYAQEPYYSTAVGLLHYGKESHLSGEAEVEKR
  consensus/100%                   QQGVKHHLAAGIVLTGGAAQhEGLAACAQRVFHTQVRIGtPLNITGLTDYAQEPYYSTAVGLLHYGKEoHLsGEA-VEKR
  consensus/90%                    QQGVKHHLAAGIVLTGGAAQhEGLAACAQRVFHTQVRIGtPLNITGLTDYAQEPYYSTAVGLLHYGKEoHLsGEA-VEKR
  consensus/80%                    QQGVKHHLAAGIVLTGGAAQIEGLAACAQRVFHTQVRIGAPLNITGLTDYAQEPYYSTAVGLLHYGKEoHLsGEAEVEKR
  consensus/70%                    QQGVKHHLAAGIVLTGGAAQIEGLAACAQRVFHTQVRIGAPLNITGLTDYAQEPYYSTAVGLLHYGKESHLSGEAEVEKR

                    cov    pid 401         .         ] 420
1 Salmonella      100.0% 100.0%    VTASVGSWIKRLNSWLRKEF
2 Escherichia     100.0%  99.5%    VTASVGSWIKRLNSWLRKEF
3 Citrobacter     100.0%  99.3%    VTASVGSWIKRLNSWLRKEF
4 Klebsiella      100.0%  98.8%    VTASVGSWIKRLNSWLRKEF
5 Cronobacter      99.5%  97.9%    --TSVGSWIKRINSWLRKEF
6 Enterobacter     99.5%  98.3%    --VSVGSWVKRLNNWLRKEF
7 Phytobacter      99.5%  97.4%    --ASVGSWFKRINSWLRKEF
8 Kosakonia        99.5%  96.7%    --VSVGSWFKRINSWLRKEF
9 Lelliottia       99.5%  99.0%    --ASVGSWVKRLNSWLRKEF
  consensus/100%                   ..sSVGSWhKRlNsWLRKEF
  consensus/90%                    ..sSVGSWhKRlNsWLRKEF
  consensus/80%                    ..sSVGSWhKRlNSWLRKEF
  consensus/70%                    ..sSVGSWlKRlNSWLRKEF
```

```
#  Percent Identity  Matrix - created by Clustal2.1
#
#

     1: Salmonella     100.00   99.52   99.29   98.81   98.33   98.80   97.85   97.13   99.52
     2: Escherichia     99.52  100.00   98.81   98.33   98.33   98.33   97.85   97.13   99.04
     3: Citrobacter     99.29   98.81  100.00   99.05   98.56   98.56   98.09   96.89   99.28
     4: Klebsiella      98.81   98.33   99.05  100.00   98.09   98.09   97.61   96.41   98.80
     5: Cronobacter     98.33   98.33   98.56   98.09  100.00   97.85   98.09   97.61   98.33
     6: Enterobacter    98.80   98.33   98.56   98.09   97.85  100.00   97.85   97.61   99.28
     7: Phytobacter     97.85   97.85   98.09   97.61   98.09   97.85  100.00   98.80   98.09
     8: Kosakonia       97.13   97.13   96.89   96.41   97.61   97.61   98.80  100.00   97.37
     9: Lelliottia      99.52   99.04   99.28   98.80   98.33   99.28   98.09   97.37  100.00
```

5. MinD

```
                        cov    pid   1 [         .         .         .         .         :         .         .         . 80
1 Salmonella         100.0% 100.0%     MARIIVVTSGKGGVGKTTSSAAIATGLAQKGKKTVVIDFDIGLRNLDLIMGCERRVVYDFVNVIQGDATLNQALIKDKRT
2 Escherichia        100.0%  97.4%     MARIIVVTSGKGGVGKTTSSAAIATGLAQKGKKTVVIDFDIGLRNLDLIMGCERRVVYDFVNVIQGDATLNQALIKDKRT
3 Citrobacter        100.0%  98.5%     MARIIVVTSGKGGVGKTTSSAAIATGLAQKGKKTVVIDFDIGLRNLDLIMGCERRVVYDFVNVIQGDATLNQALIKDKRT
4 Klebsiella         100.0%  96.3%     MARIIVVTSGKGGVGKTTSSAAIATGLAQKGKKTVVIDFDIGLRNLDLIMGCERRVVYDFVNVIQGDATLNQALIKDKRT
5 Cronobacter        100.0%  94.4%     MARIIVVTSGKGGVGKTTSSAAIATGLAQKGKKTVVIDFDIGLRNLDLIMGCERRVVYDFVNVIQGDATLNQALIRDKRT
6 Enterobacter       100.0%  97.0%     MARIIVVTSGKGGVGKTTSSAAIATGLAQKGKKTVVIDFDIGLRNLDLIMGCERRVVYDFVNVIQGDATLNQALIKDKRT
7 Phytobacter        100.0%  94.8%     MARIIVVTSGKGGVGKTTSSAAIATGLAQKGKKTVVIDFDIGLRNLDLIMGCERRVVYDFVNVIQGDATLNQALIKDKRT
8 Kosakonia          100.0%  95.2%     MARIIVVTSGKGGVGKTTSSAAIATGLAQKGKKTVVIDFDIGLRNLDLIMGCERRVVYDFVNVIQGDATLNQALIKDKRT
9 Lelliottia         100.0%  96.3%     MARIIVVTSGKGGVGKTTSSAAIATGLAQKGKKTVVIDFDIGLRNLDLIMGCERRVVYDFVNVIQGDATLNQAMIKDKRT
  consensus/100%                       MARIIVVTSGKGGVGKTTSSAAIATGLAQKGKKTVVIDFDIGLRNLDLIMGCERRVVYDFVNVIQGDATLNQAhI+DKRT
  consensus/90%                        MARIIVVTSGKGGVGKTTSSAAIATGLAQKGKKTVVIDFDIGLRNLDLIMGCERRVVYDFVNVIQGDATLNQAhI+DKRT
  consensus/80%                        MARIIVVTSGKGGVGKTTSSAAIATGLAQKGKKTVVIDFDIGLRNLDLIMGCERRVVYDFVNVIQGDATLNQALIKDKRT
  consensus/70%                        MARIIVVTSGKGGVGKTTSSAAIATGLAQKGKKTVVIDFDIGLRNLDLIMGCERRVVYDFVNVIQGDATLNQALIKDKRT

                        cov    pid  81           .         1         .         .         .         .         :         . 160
1 Salmonella         100.0% 100.0%     ENLFILPASQTRDKDALTREGVAKVLDSLKAMDFEFIVCDSPAGIETGALMALYFADEAIITTNPEVSSVRDSDRILGIL
2 Escherichia        100.0%  97.4%     ENLYILPASQTRDKDALTREGVAKVLDDLKAMDFEFIVCDSPAGIETGALMALYFADEAIITTNPEVSSVRDSDRILGIL
3 Citrobacter        100.0%  98.5%     ENLFILPASQTRDKDALTRDGVAKVLDDLKAMDFEFIVCDSPAGIETGALMALYFADEAIITTNPEVSSVRDSDRILGIL
4 Klebsiella         100.0%  96.3%     ENLYILPASQTRDKDALTREGVDKVLEELKKMDFDFIVCDSPAGIETGALMALYFADEAIITTNPEVSSVRDSDRILGIL
5 Cronobacter        100.0%  94.4%     ESLYILPASQTRDKDALTREGVEKVLDELKKMEFDFIVCDSPAGIETGALMALYFADEAIITTNPEVSSVRDSDRILGIL
6 Enterobacter       100.0%  97.0%     ENLYILPASQTRDKDALTREGVEKVLDELKKMEFDFIVCDSPAGIETGALMALYFADEAIITTNPEVSSVRDSDRILGIL
7 Phytobacter        100.0%  94.8%     ENLYILPASQTRDKDALTREGVEKVLDELQKMEFDFVICDSPAGIETGALMALYFADEAIITTNPEVSSVRDSDRILGIL
8 Kosakonia          100.0%  95.2%     ENLYILPASQTRDKDALTREGVDKVLEDLKKMDFDFIVCDSPAGIETGALMALYFADEAIITTNPEVSSVRDSDRILGIL
9 Lelliottia         100.0%  96.3%     ENLYILPASQTRDKDALTREGVEKVLDELKKMEFDFVVCDSPAGIETGALMALYFADEAIITTNPEVSSVRDSDRILGIL
  consensus/100%                       EsLaILPASQTRDKDALTR-GVtKVL-pLptM-F-FllCDSPAGIETGALMALYFADEAIITTNPEVSSVRDSDRILGIL
  consensus/90%                        EsLaILPASQTRDKDALTR-GVtKVL-pLptM-F-FllCDSPAGIETGALMALYFADEAIITTNPEVSSVRDSDRILGIL
  consensus/80%                        ENLaILPASQTRDKDALTREGVtKVL--LKtM-F-FlVCDSPAGIETGALMALYFADEAIITTNPEVSSVRDSDRILGIL
  consensus/70%                        ENLYILPASQTRDKDALTREGVtKVLD-LKtM-F-FIVCDSPAGIETGALMALYFADEAIITTNPEVSSVRDSDRILGIL

                        cov    pid 161           .         .         .         2         .         .         .         . 240
1 Salmonella         100.0% 100.0%     ASKSRRAENGEEPIKEHLLLTRYNPGRVNKGDMLSMEDVLEILRIKLVGVIPEDQSVLRASNQGEPVILDATADAGKAYA
2 Escherichia        100.0%  97.4%     ASKSRRAENGEEPIKEHLLLTRYNPGRVSRGDMLSMEDVLEILRIKLVGVIPEDQSVLRASNQGEPVILDINADAGKAYA
3 Citrobacter        100.0%  98.5%     ASKSRRAENGEEPIKEHLLLTRYNPGRVNKGDMLSMEDVLEILRIKLVGVIPEDQSVLRASNQGEPVILDSAADAGKAYA
4 Klebsiella         100.0%  96.3%     ASKSRRAENGEEPIKEHLLLTRYNPGRVNKGDMLSMEDVLEILRINLVGVIPEDQSVLRASNQGEPVILDAASDAGKAYA
5 Cronobacter        100.0%  94.4%     SSKSRRAENGEAPIKEHLLLTRYNPGRVSKGDMLSMEDVLEILRIPLVGVIPEDQSVLRASNQGEPVILDDTSDAGKAYA
6 Enterobacter       100.0%  97.0%     ASKSRRAENGEDPIKEHLLLTRYNPGRVNKGDMLSMEDVLEILRIKLVGVIPEDQSVLRASNQGEPVILDTTADAGKAYA
7 Phytobacter        100.0%  94.8%     ASKSRRAENGQEPIKEHLLLTRYNPGRVNKGDMLSMEDVLEILRIKLVGVIPEDQSVLRASNQGEPVILDTTADAGKAYA
8 Kosakonia          100.0%  95.2%     ASKSRRAENGEEPIKEHLLLTRYNPGRVNRGDMLSMEDVLEILRIKLIGVIPEDQSVLRASNQGEPVILDTNADAGKAYA
9 Lelliottia         100.0%  96.3%     ASKSRRAENGQEPIKEHLLLTRYNPGRVNKGDMLSMEDVLEILRIKLVGVIPEDQSVLRASNQGEPVILDSTADAGKAYA
  consensus/100%                       uSKSRRAENGptPIKEHLLLTRYNPGRVs+GDMLSMEDVLEILRI.LlGVIPEDQSVLRASNQGEPVILD.suDAGKAYA
  consensus/90%                        uSKSRRAENGptPIKEHLLLTRYNPGRVs+GDMLSMEDVLEILRI.LlGVIPEDQSVLRASNQGEPVILD.suDAGKAYA
  consensus/80%                        ASKSRRAENGp-PIKEHLLLTRYNPGRVs+GDMLSMEDVLEILRIpLVGVIPEDQSVLRASNQGEPVILDssuDAGKAYA
  consensus/70%                        ASKSRRAENGEEPIKEHLLLTRYNPGRVNKGDMLSMEDVLEILRIKLVGVIPEDQSVLRASNQGEPVILDssADAGKAYA

                        cov    pid 241           :         .         ] 270
1 Salmonella         100.0% 100.0%     DTVDRLLGEERPFRFIEEEKKGFLKRLFGG
2 Escherichia        100.0%  97.4%     DTVERLLGEERPFRFIEEEKKGFLKRLFGG
3 Citrobacter        100.0%  98.5%     DTVDRLLGEERPFRFIEEEKKGFLKRLFGG
4 Klebsiella         100.0%  96.3%     DTVERLLGEERPFRFIEEEKKGFLKRLFGG
5 Cronobacter        100.0%  94.4%     DTVDRLMGEERPFRFIEEEKKGFLKRLFGG
6 Enterobacter       100.0%  97.0%     DTVDRLLGEERPFRFIEEEKKGFLKRLFGG
7 Phytobacter        100.0%  94.8%     DTVERLLGEDRPFRFIDEEKKGFLKRLFGG
8 Kosakonia          100.0%  95.2%     DAVERLLGEDRPFRFIEEEKKGFLKRLFGG
9 Lelliottia         100.0%  96.3%     DTVDRLLGEERPFRFIEEEKKGFLKRLFGG
  consensus/100%                       DsV-RLhGE-RPFRFI-EEKKGFLKRLFGG
  consensus/90%                        DsV-RLhGE-RPFRFI-EEKKGFLKRLFGG
  consensus/80%                        DTV-RLLGE-RPFRFIEEEKKGFLKRLFGG
  consensus/70%                        DTV-RLLGEERPFRFIEEEKKGFLKRLFGG
```

```
#  Percent Identity  Matrix - created by Clustal2.1
#
#

     1: Salmonella     100.00   97.41   98.52   96.30   94.44   97.04   94.81   95.19   96.30
     2: Escherichia     97.41  100.00   97.41   95.93   94.07   95.93   94.44   96.67   95.19
     3: Citrobacter     98.52   97.41  100.00   95.93   93.70   96.30   94.07   95.19   95.93
     4: Klebsiella      96.30   95.93   95.93  100.00   95.19   96.67   95.19   96.67   95.93
     5: Cronobacter     94.44   94.07   93.70   95.19  100.00   96.67   94.07   92.96   95.56
     6: Enterobacter    97.04   95.93   96.30   96.67   96.67  100.00   97.04   95.93   98.15
     7: Phytobacter     94.81   94.44   94.07   95.19   94.07   97.04  100.00   95.19   97.41
     8: Kosakonia       95.19   96.67   95.19   96.67   92.96   95.93   95.19  100.00   94.81
     9: Lelliottia      96.30   95.19   95.93   95.93   95.56   98.15   97.41   94.81  100.00
```

Cellular homeostasis:

1. SodA

```
                      cov    pid    1 [         .         .         .         .         :         .         .         . 80
1 Salmonella      100.0% 100.0%      MSYTLPSLPYAYDALEPHFDKQTMEIHHTKHHQTYVNNANAALENLPEFASLPVEELITKLDQVPADKKTVLRNNAGGHA
2 Escherichia     100.0%  97.6%      MSYTLPSLPYAYDALEPHFDKQTMEIHHTKHHQTYVNNANAALESLPEFANLPVEELITKLDQLPADKKTVLRNNAGGHA
3 Citrobacter     100.0%  98.1%      MSYTLPSLPYAYDALEPHFDKQTMEIHHTKHHQTYVNNANAALESLPEFASLPVEELITKLDQVPADKKTVLRNNAGGHA
4 Klebsiella      100.0%  96.1%      MSYTLPSLPYAYDALEPHFDKQTMEIHHTKHHQTYVNNANAALESLPEFANLSAEELITKLDQLPADKKTVLRNNAGGHA
5 Cronobacter     100.0%  95.1%      MSYTLPSLPYAYDALEPHFDKQTMEIHHTKHHQTYVNNANAALESLPELANLPVEELIAKLDQVPADKKTVLRNNAGGHA
6 Enterobacter    100.0%  97.1%      MSYTLPSLPYAYDALEPHFDKQTMEIHHTKHHQTYVNNANAALESLPEFANLPVEELITKLDQLPADKKTVLRNNAGGHA
7 Phytobacter     100.0%  97.1%      MSYTLPSLPYAYDALEPHFDKQTMEIHHTKHHQTYVNNANAALESLPEFASLSAEELITKLDQLPADKKTVLRNNAGGHA
8 Kosakonia       100.0%  98.1%      MSYTLPALPYAYDALEPHFDKQTMEIHHTKHHQTYVNNANAALESLPEFASLPVEELITKLDQLPADKKTVLRNNAGGHA
9 Lelliottia      100.0%  97.1%      MSYTLPSLPYAYDALEPHFDKQTMEIHHTKHHQTYVNNANAALESLPEFASLPVEELITKLDQLPADKKTVLRNNAGGHA
  consensus/100%                     MSYTLPuLPYAYDALEPHFDKQTMEIHHTKHHQTYVNNANAALEsLPEhAsLssEELIsKLDQlPADKKTVLRNNAGGHA
  consensus/90%                      MSYTLPuLPYAYDALEPHFDKQTMEIHHTKHHQTYVNNANAALEsLPEhAsLssEELIsKLDQlPADKKTVLRNNAGGHA
  consensus/80%                      MSYTLPSLPYAYDALEPHFDKQTMEIHHTKHHQTYVNNANAALESLPEFAsLssEELITKLDQlPADKKTVLRNNAGGHA
  consensus/70%                      MSYTLPSLPYAYDALEPHFDKQTMEIHHTKHHQTYVNNANAALESLPEFAsLPVEELITKLDQlPADKKTVLRNNAGGHA
```

```
                      cov    pid   81           .         1         .         .         .         .         :         . 160
1 Salmonella      100.0% 100.0%      NHSLFWKGLKKGTTLQGDLKAAIERDFGSVDNFKAEFEKAAATRFGSGWAWLVLKGDKLAVVSTANQDSPLMGEAISGAS
2 Escherichia     100.0%  97.6%      NHSLFWKGLKKGTTLQGDLKAAIERDFGSVDNFKAEFEKAAASRFGSGWAWLVLKGDKLAVVSTANQDSPLMGEAISGAS
3 Citrobacter     100.0%  98.1%      NHSLFWKGLKKGTTLQGDLKAAIERDFGSVEKFKEEFEKAAATRFGSGWAWLVLKGDKLAVVSTANQDSPLMGEAISGAS
4 Klebsiella      100.0%  96.1%      NHSLFWKGLKTGTTLQGDLKAAIERDFGSVDNFKAEFEKAAATRFGSGWAWLVLKGDKLAVVSTANQDSPLMGEAISGAS
5 Cronobacter     100.0%  95.1%      NHSLFWKGLKKGTTLQGDLKAAIERDFGSVEKFKEEFEKAAATRFGSGWAWLVLKGDKLAVVSTANQDSPLMGEAISGAS
6 Enterobacter    100.0%  97.1%      NHSLFWKGLKTGTTLQGDLKAAIERDFGSVDNFKAEFEKAAATRFGSGWAWLVLKGDKLAVVSTANQDSPLMGEAISGAS
7 Phytobacter     100.0%  97.1%      NHSFFWKGLKKGTTLQGDLKAAIERDFGSVDNFKAEFEKAAATRFGSGWAWLVLKGDKLAVVSTANQDSPLMGEAISGAS
8 Kosakonia       100.0%  98.1%      NHSLFWKGLKKGTTLQGDLKAAIERDFGSVDNFKAEFEKAAATRFGSGWAWLVLKGDKLAVVSTANQDSPLMGEAISGAS
9 Lelliottia      100.0%  97.1%      NHSLFWKGLKTGTTLQGDLKAAIERDFGSVDNFKAEFEKAAATRFGSGWAWLVLKGDKLAVVSTANQDSPLMGEAISGAS
  consensus/100%                     NHShFWKGLKpGTTLQGDLKAAIERDFGSV-pFKtEFEKAAAoRFGSGWAWLVLKGDKLAVVSTANQDSPLMGEAISGAS
  consensus/90%                      NHShFWKGLKpGTTLQGDLKAAIERDFGSV-pFKtEFEKAAAoRFGSGWAWLVLKGDKLAVVSTANQDSPLMGEAISGAS
  consensus/80%                      NHSLFWKGLKpGTTLQGDLKAAIERDFGSV-pFKtEFEKAAATRFGSGWAWLVLKGDKLAVVSTANQDSPLMGEAISGAS
  consensus/70%                      NHSLFWKGLKpGTTLQGDLKAAIERDFGSVDNFKAEFEKAAATRFGSGWAWLVLKGDKLAVVSTANQDSPLMGEAISGAS
```

```
                      cov    pid  161           .         .         .         2     ] 206
1 Salmonella      100.0% 100.0%      GFPILGLDVWEHAYYLKFQNRRPDYIKEFWNVVNWDEAAARFAAKK
2 Escherichia     100.0%  97.6%      GFPIMGLDVWEHAYYLKFQNRRPDYIKEFWNVVNWDEAAARFAAKK
3 Citrobacter     100.0%  98.1%      GFPILGLDVWEHAYYLKFQNRRPDYIKEFWNVVNWDEAAARFAAKK
4 Klebsiella      100.0%  96.1%      GFPILGLDVWEHAYYLKFQNRRPDYIKAFWDVVNWDEAAARFAAKK
5 Cronobacter     100.0%  95.1%      GYPILGLDVWEHAYYLKFQNRRPDYIKEFWNVVNWDEAAARFASQK
6 Enterobacter    100.0%  97.1%      GFPILGLDVWEHAYYLKFQNRRPDYIKAFWDVVNWDEAAARFAAKK
7 Phytobacter     100.0%  97.1%      GFPIVGLDVWEHAYYLKFQNRRPDYIKEFWNVVNWDEAAARFAAKK
8 Kosakonia       100.0%  98.1%      GFPIVGLDVWEHAYYLKFQNRRPDYIKEFWNVVNWDEAAARFAAKK
9 Lelliottia      100.0%  97.1%      GFPIVGLDVWEHAYYLKFQNRRPDYIKAFWDVVNWDEAAARFAAKK
  consensus/100%                     GaPIhGLDVWEHAYYLKFQNRRPDYIKtFWsVVNWDEAAARFAupK
  consensus/90%                      GaPIhGLDVWEHAYYLKFQNRRPDYIKtFWsVVNWDEAAARFAupK
  consensus/80%                      GFPIlGLDVWEHAYYLKFQNRRPDYIKtFWsVVNWDEAAARFAAKK
  consensus/70%                      GFPIlGLDVWEHAYYLKFQNRRPDYIKtFWsVVNWDEAAARFAAKK
```

```
#  Percent Identity  Matrix - created by Clustal2.1
#
#

     1: Salmonella     100.00   97.57   98.06   96.12   95.15   97.09   97.09   98.06   97.09
     2: Escherichia     97.57  100.00   96.60   96.60   94.66   97.57   97.09   98.06   97.09
     3: Citrobacter     98.06   96.60  100.00   95.15   97.09   96.12   96.12   97.09   96.12
     4: Klebsiella      96.12   96.60   95.15  100.00   93.20   99.03   97.09   96.12   98.06
     5: Cronobacter     95.15   94.66   97.09   93.20  100.00   94.17   93.20   94.17   93.20
     6: Enterobacter    97.09   97.57   96.12   99.03   94.17  100.00   96.12   97.09   99.03
     7: Phytobacter     97.09   97.09   96.12   97.09   93.20   96.12  100.00   98.06   97.09
     8: Kosakonia       98.06   98.06   97.09   96.12   94.17   97.09   98.06  100.00   98.06
     9: Lelliottia      97.09   97.09   96.12   98.06   93.20   99.03   97.09   98.06  100.00
```

2. DnaK

```
                     cov    pid    1 [                                                                              80
1 Salmonella      100.0% 100.0%     MGKIIGIDLGTTNSCVAIMDGTQARVLENAEGDRTTPSIIAYTQDGETLVGQPAKRQAVTNPQNTLFAIKRLIGRRFQDE
2 Escherichia     100.0%  96.9%     MGKIIGIDLGTTNSCVAIMDGTTPRVLENAEGDRTTPSIIAYTQDGETLVGQPAKRQAVTNPQNTLFAIKRLIGRRFQDE
3 Citrobacter     100.0%  98.7%     MGKIIGIDLGTTNSCVAIMDGTQARVLENAEGDRTTPSIIAYTQDGETLVGQPAKRQAVTNPQNTLFAIKRLIGRRFQDE
4 Klebsiella      100.0%  97.6%     MGKIIGIDLGTTNSCVAIMDGTTARVLENAEGDRTTPSIIAYTQDGETLVGQPAKRQAVTNPQNTLFAIKRLIGRRFQDE
5 Cronobacter     100.0%  97.5%     MGKIIGIDLGTTNSCVAIMDGTQARVLENAEGDRTTPSIIAYTQDGETLVGQPAKRQAVTNPQNTLFAIKRLIGRRFQDE
6 Enterobacter     99.8%  97.6%     MGKIIGIDLGTTNSCVAIMDGTTARVLENAEGDRTTPSIIAYTQDGETLVGQPAKRQAVTNPQNTLFAIKRLIGRRFQDE
7 Phytobacter     100.0%  96.9%     MGKIIGIDLGTTNSCVAIMDGTTARVLENAEGDRTTPSIIAYTQDGETLVGQPAKRQAVTNPQNTLFAIKRLIGRRFQDE
8 Kosakonia        99.8%  96.1%     MGKIIGIDLGTTNSCVAIMDGAQARVLENAEGDRTTPSIIAYTQDGETLVGQPAKRQAVTNPQNTLFAIKRLIGRRFQDE
9 Lelliottia      100.0%  95.6%     MGKIIGIDLGTTNSCVAIMDGTTARVLENAEGDRTTPSIIAYTQDGETLVGQPAKRQAVTNPQNTLFAIKRLIGRRFQDE
  consensus/100%                    MGKIIGIDLGTTNSCVAIMDGspsRVLENAEGDRTTPSIIAYTQDGETLVGQPAKRQAVTNPQNTLFAIKRLIGRRFQDE
  consensus/90%                     MGKIIGIDLGTTNSCVAIMDGspsRVLENAEGDRTTPSIIAYTQDGETLVGQPAKRQAVTNPQNTLFAIKRLIGRRFQDE
  consensus/80%                     MGKIIGIDLGTTNSCVAIMDGTpARVLENAEGDRTTPSIIAYTQDGETLVGQPAKRQAVTNPQNTLFAIKRLIGRRFQDE
  consensus/70%                     MGKIIGIDLGTTNSCVAIMDGTpARVLENAEGDRTTPSIIAYTQDGETLVGQPAKRQAVTNPQNTLFAIKRLIGRRFQDE

                     cov    pid   81                   1                                                           160
1 Salmonella      100.0% 100.0%     EVQRDVSIMPYKIIGADNGDAWLDVKGQKMAPPQISAEVLKKMKKTAEDYLGEPVTEAVITVPAYFNDAQRQATKDAGRI
2 Escherichia     100.0%  96.9%     EVQRDVSIMPFKIIAADNGDAWVEVKGQKMAPPQISAEVLKKMKKTAEDYLGEPVTEAVITVPAYFNDAQRQATKDAGRI
3 Citrobacter     100.0%  98.7%     EVQRDVSIMPYKIIGADNGDAWLDVKGQKMAPPQISAEVLKKMKKTAEDYLGEPVTEAVITVPAYFNDAQRQATKDAGRI
4 Klebsiella      100.0%  97.6%     EVQRDVSIMPYKIVAADNGDAWLDVKGTKTAPPQISAEVLKKMKKTAEDYLGEPVTEAVITVPAYFNDAQRQATKDAGRI
5 Cronobacter     100.0%  97.5%     EVQRDESIMPYKIISADNGDAWLDVKGQKMAPPQISAEVLKKMKKTAEDYLGEPVTEAVITVPAYFNDAQRQATKDAGRI
6 Enterobacter     99.8%  97.6%     EVQRDVSIMPYKIIAADNGDAWLDVKGTKTAPPQISAEVLKKMKKTAEDYLGEPVTEAVITVPAYFNDAQRQATKDAGRI
7 Phytobacter     100.0%  96.9%     EVQRDVSIMPYKIIAADNGDAWLDVKGTKTAPPQISAEVLKKMKKTAEDYLGEPVTEAVITVPAYFNDAQRQATKDAGRI
8 Kosakonia        99.8%  96.1%     EVQRDESIMPYKIIAADNGDAWIDVKGTKMAPPQISAEVLKKMKKTAEDYLGEPVTEAVITVPAYFNDAQRQATKDAGRI
9 Lelliottia      100.0%  95.6%     EVQRDEAIMPYKIIGADNGDAWIDVKGQKMAPPQISAEVLKKMKKTAEDYLGEPVTEAVITVPAYFNDAQRQATKDAGRI
  consensus/100%                    EVQRD.uIMPaKIluADNGDAWl-VKGpKhAPPQISAEVLKKMKKTAEDYLGEPVTEAVITVPAYFNDAQRQATKDAGRI
  consensus/90%                     EVQRD.uIMPaKIluADNGDAWl-VKGpKhAPPQISAEVLKKMKKTAEDYLGEPVTEAVITVPAYFNDAQRQATKDAGRI
  consensus/80%                     EVQRD.SIMPYKIIuADNGDAWlDVKGpKhAPPQISAEVLKKMKKTAEDYLGEPVTEAVITVPAYFNDAQRQATKDAGRI
  consensus/70%                     EVQRD.SIMPYKIIuADNGDAWlDVKGpKhAPPQISAEVLKKMKKTAEDYLGEPVTEAVITVPAYFNDAQRQATKDAGRI

                     cov    pid  161                                         2                                     240
1 Salmonella      100.0% 100.0%     AGLEVKRIINEPTAAALAYGLDKEVGNRTIAVYDLGGGTFDISIIEIDEVDGEKTFEVLATNGDTHLGGEDFDTRLINYL
2 Escherichia     100.0%  96.9%     AGLEVKRIINEPTAAALAYGLDKGTGNRTIAVYDLGGGTFDISIIEIDEVDGEKTFEVLATNGDTHLGGEDFDSRLINYL
3 Citrobacter     100.0%  98.7%     AGLEVKRIINEPTAAALAYGLDKEVGNRTIAVYDLGGGTFDISIIEIDEVDGEKTFEVLATNGDTHLGGEDFDSRMINYL
4 Klebsiella      100.0%  97.6%     AGLEVKRIINEPTAAALAYGLDKEVGNRTIAVYDLGGGTFDISIIEIDEVDGEKTFEVLATNGDTHLGGEDFDTRLINYL
5 Cronobacter     100.0%  97.5%     AGLEVKRIINEPTAAALAYGLDKETGNRTIAVYDLGGGTFDISIIEIDEVDGEKTFEVLATNGDTHLGGEDFDSRLINYL
6 Enterobacter     99.8%  97.6%     AGLEVKRIINEPTAAALAYGLDKEVGNRTIAVYDLGGGTFDISIIEIDEVDGEKTFEVLATNGDTHLGGEDFDTRLINYL
7 Phytobacter     100.0%  96.9%     AGLEVKRIINEPTAAALAYGLDKEVGNRTIAVYDLGGGTFDISIIEIDEVDGEKTFEVLATNGDTHLGGEDFDSRLINYL
8 Kosakonia        99.8%  96.1%     AGLEVKRIINEPTAAALAYGLDKETGNRTIAVYDLGGGTFDISIIEIDEVDGEKTFEVLATNGDTHLGGEDFDSRLINYL
9 Lelliottia      100.0%  95.6%     AGLEVKRIINEPTAAALAYGLDKEVGNRTIAVYDLGGGTFDISIIEIDEVDGEKTFEVLATNGDTHLGGEDFDSRMINYL
  consensus/100%                    AGLEVKRIINEPTAAALAYGLDKtsGNRTIAVYDLGGGTFDISIIEIDEVDGEKTFEVLATNGDTHLGGEDFDoRhINYL
  consensus/90%                     AGLEVKRIINEPTAAALAYGLDKtsGNRTIAVYDLGGGTFDISIIEIDEVDGEKTFEVLATNGDTHLGGEDFDoRhINYL
  consensus/80%                     AGLEVKRIINEPTAAALAYGLDKEsGNRTIAVYDLGGGTFDISIIEIDEVDGEKTFEVLATNGDTHLGGEDFDoRhINYL
  consensus/70%                     AGLEVKRIINEPTAAALAYGLDKEsGNRTIAVYDLGGGTFDISIIEIDEVDGEKTFEVLATNGDTHLGGEDFDoRLINYL

                     cov    pid  241                                                             3                 320
1 Salmonella      100.0% 100.0%     VDEFKKDQGIDLRNDPLAMQRLKEAAEKAKIELSSAQQTDVNLPYITADATGPKHMNIKVTRAKLESLVEDLVNRSIEPL
2 Escherichia     100.0%  96.9%     VEEFKKDQGIDLRNDPLAMQRLKEAAEKAKIELSSAQQTDVNLPYITADATGPKHMNIKVTRAKLESLVEDLVNRSIEPL
3 Citrobacter     100.0%  98.7%     VEEFKKDQGIDLRNDPLAMQRLKEAAEKAKIELSSAQQTDVNLPYITADATGPKHMNIKVTRAKLESLVEDLVNRSIEPL
4 Klebsiella      100.0%  97.6%     VDEFKKDQGIDLRNDPLAMQRLKEAAEKAKIELSSAQQTDVNLPYITADATGPKHMNIKVTRAKLESLVEDLVNRSIEPL
5 Cronobacter     100.0%  97.5%     VEEFKKDQGIDLRNDPLAMQRLKEAAEKAKIELSSAQQTDVNLPYITADATGPKHMNIKVTRAKLESLVEDLVNRSIEPL
6 Enterobacter     99.8%  97.6%     VDEFKKDQGIDLRNDPLAMQRLKEAAEKAKIELSSAQQTDVNLPYITADATGPKHMNIKVTRAKLESLVEDLVNRSIEPL
7 Phytobacter     100.0%  96.9%     VEEFKKDQGIDLRNDPLAMQRLKEAAEKAKIELSSAQQTDVNLPYITADATGPKHMNIKVTRAKLESLVEDLVNRSIEPL
8 Kosakonia        99.8%  96.1%     VEEFKKDQGIDLRNDPLAMQRLKEAAEKAKIELSSAQQTDVNLPYITADATGPKHMNIKVTRAKLESLVEDLVNRSIEPL
9 Lelliottia      100.0%  95.6%     VDEFKKDQGIDLRNDPLAMQRLKEAAEKAKIELSSAQQTDVNLPYITADASGPKHMNIKVTRAKLESLVEDLVNRSIEPL
  consensus/100%                    V-EFKKDQGIDLRNDPLAMQRLKEAAEKAKIELSSAQQTDVNLPYITADAoGPKHMNIKVTRAKLESLVEDLVNRSIEPL
  consensus/90%                     V-EFKKDQGIDLRNDPLAMQRLKEAAEKAKIELSSAQQTDVNLPYITADAoGPKHMNIKVTRAKLESLVEDLVNRSIEPL
  consensus/80%                     V-EFKKDQGIDLRNDPLAMQRLKEAAEKAKIELSSAQQTDVNLPYITADATGPKHMNIKVTRAKLESLVEDLVNRSIEPL
  consensus/70%                     V-EFKKDQGIDLRNDPLAMQRLKEAAEKAKIELSSAQQTDVNLPYITADATGPKHMNIKVTRAKLESLVEDLVNRSIEPL
```

2. DnaK

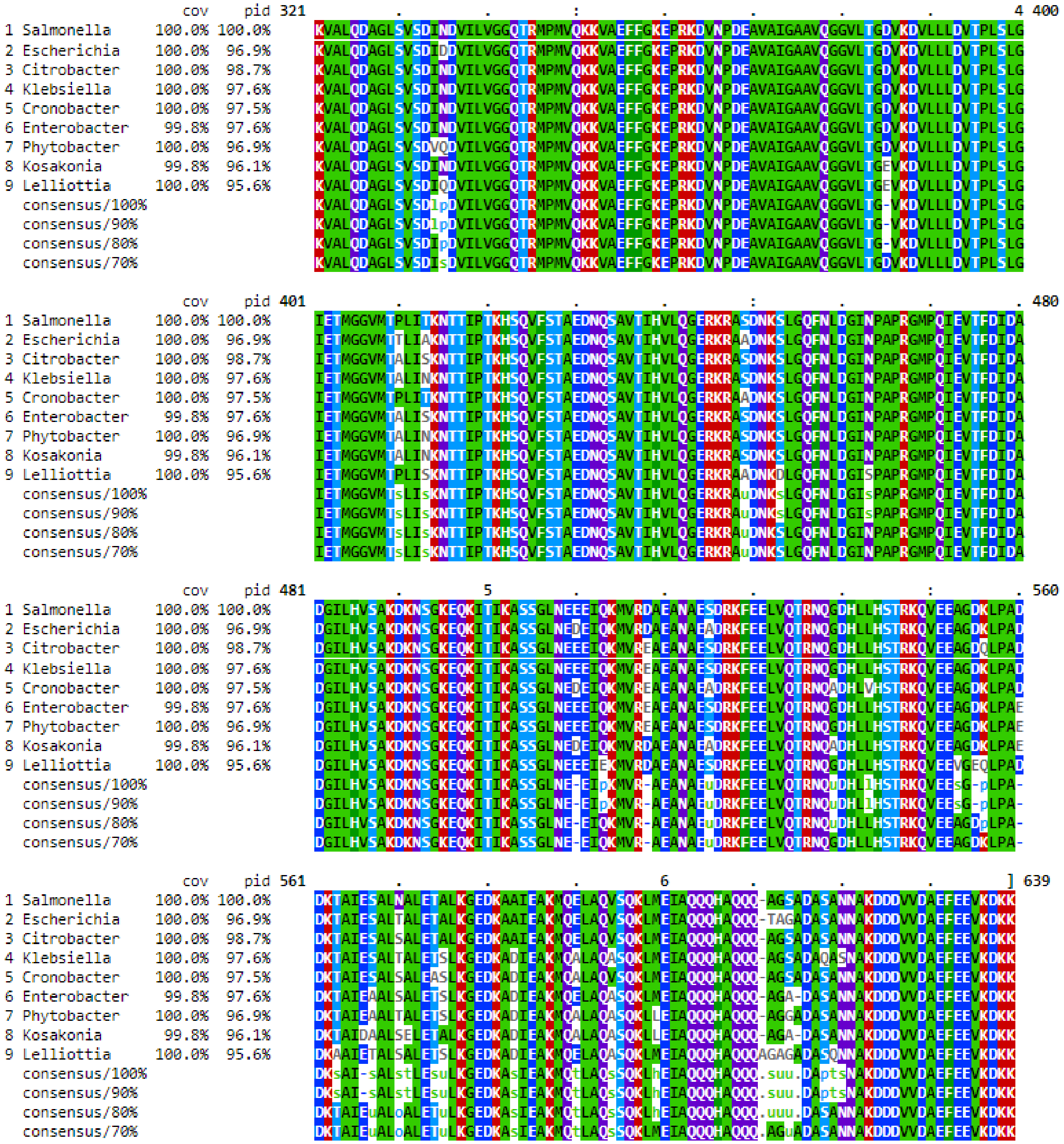

Percent Identity Matrix - created by Clustal2.1
#
#

1: Salmonella	100.00	96.87	98.75	97.65	97.49	97.80	96.87	96.23	95.77
2: Escherichia	96.87	100.00	96.71	95.77	96.55	95.92	96.08	95.76	94.98
3: Citrobacter	98.75	96.71	100.00	97.34	97.49	97.80	97.18	96.39	96.08
4: Klebsiella	97.65	95.77	97.34	100.00	96.71	98.59	98.28	96.23	94.98
5: Cronobacter	97.49	96.55	97.49	96.71	100.00	96.70	96.55	97.02	95.14
6: Enterobacter	97.80	95.92	97.80	98.59	96.70	100.00	98.59	97.02	95.76
7: Phytobacter	96.87	96.08	97.18	98.28	96.55	98.59	100.00	97.17	95.30
8: Kosakonia	96.23	95.76	96.39	96.23	97.02	97.02	97.17	100.00	94.66
9: Lelliottia	95.77	94.98	96.08	94.98	95.14	95.76	95.30	94.66	100.00

3. UspA

```
                     cov    pid    1 [         .         .         .         .         :         .         .         . 80
1 Salmonella       100.0% 100.0%     MAYKHILIAVDLSPESKVLVEKAVSMARPYNAKISLIHVDVNYSDLYTGLIDVNLGDMQKRISEETHHALTELSTNAGYP
2 Escherichia      100.0%  99.3%     MAYKHILIAVDLSPESKVLVEKAVSMARPYNAKVSLIHVDVNYSDLYTGLIDVNLGDMQKRISEETHHALTELSTNAGYP
3 Citrobacter      100.0%  99.3%     MAYKHILIAVDLSPESKVLVEKAVSMARPYNAKVSLIHVDVNYSDLYTGLIDVNLGDMQKRISEETHHALTELSTNAGYP
4 Klebsiella       100.0%  97.9%     MAYKHILIAVDLSPESKVLVEKAVSMARPYNAKVSLIHVDVNYSDLYTGLIDVNLGDMQKRISEETHHALTELSTNAGYP
5 Cronobacter      100.0%  96.6%     MAYKHILIAVDLSPESKVLVDKAVSMARPYNAKISLIHVDVNYSDLYTGLIDVNLGDMQKRISEETHHALTELSTNAGYP
6 Enterobacter     100.0%  96.6%     MAYKHILIAVDLSPESKVLVDKAVSMARPYNAKVSLIHVDVNYSDLYTGLIDVNLGDMQKRISEETHHALSELSTNAGYP
7 Phytobacter      100.0%  95.9%     MAYKHILIAVDLSPESKLLVEKAVSMARPYNAKVSLIHVDVNYSDLYTGLIDVNLGDMQKRISDETHHALTELSTNAGYP
8 Kosakonia        100.0%  97.2%     MAYKHILIAVDLSPESKLLVEKAVSMARPYNAKISLIHVDVNYSDLYTGLIDVNLGDMQKRISDETHQALTELSTNAGYP
9 Lelliottia       100.0%  96.6%     MAYKHILIAVDLSPESKVLVDKAVSMARPYNAKVSLIHVDVNYSDLYTGLIDVNLGDMQKRISEETHHALSELSTNAGYP
  consensus/100%                     MAYKHILIAVDLSPESKlLV-KAVSMARPYNAKlSLIHVDVNYSDLYTGLIDVNLGDMQKRIS-ETHpALoELSTNAGYP
  consensus/90%                      MAYKHILIAVDLSPESKlLV-KAVSMARPYNAKlSLIHVDVNYSDLYTGLIDVNLGDMQKRIS-ETHpALoELSTNAGYP
  consensus/80%                      MAYKHILIAVDLSPESKlLV-KAVSMARPYNAKlSLIHVDVNYSDLYTGLIDVNLGDMQKRIS-ETHHALoELSTNAGYP
  consensus/70%                      MAYKHILIAVDLSPESKVLV-KAVSMARPYNAKlSLIHVDVNYSDLYTGLIDVNLGDMQKRISEETHHALTELSTNAGYP

                     cov    pid   81         .         1         .         .         .         .    ] 145
1 Salmonella       100.0% 100.0%     ITETLSGSGDLGQVLVDAIKKYDMDLVVCGHHQDFWSKLMSSARQLINTVHVDMLIVPLRDEEE-
2 Escherichia      100.0%  99.3%     ITETLSGSGDLGQVLVDAIKKYDMDLVVCGHHQDFWSKLMSSARQLINTVHVDMLIVPLRDEEE-
3 Citrobacter      100.0%  99.3%     ITETLSGSGDLGQVLVDAIKKYDMDLVVCGHHQDFWSKLMSSARQLINTVHVDMLIVPLRDEEE-
4 Klebsiella       100.0%  97.9%     ITETLSGSGDLGQVLVDAIKKYDMDLVVCGHHQDFWSKLMSSARQLINTVHVDMLIVPLRDEEDE
5 Cronobacter      100.0%  96.6%     ITETLSGSGDLGQVLVDAIKKYDVDLVVCGHHQDFWSKLMSSARQLINTVHIDMLIVPLRDDEEA
6 Enterobacter     100.0%  96.6%     ITETLSGSGDLGQVLVDAIKKYDMDLVVCGHHQDFWSKLMSSARQLINTVHVDMLIVPLRDEEDE
7 Phytobacter      100.0%  95.9%     ITETLSGSGDLGQVLVDAIKKYDMDLVVCGHHQDFWSKLMSSARQLINTVHVDMLIVPLKDEDEE
8 Kosakonia        100.0%  97.2%     ITETLSGSGDLGQVLVDAIKKYDMDLVVCGHHQDFWSKLMSSARQLINTVHVDMLIVPLRDEEED
9 Lelliottia       100.0%  96.6%     ITETLSGSGDLGQVLVDAIKKYDMDLVVCGHHQDFWSKLMSSARQLINTVHVDMLIVPLRDEEDE
  consensus/100%                     ITETLSGSGDLGQVLVDAIKKYDhDLVVCGHHQDFWSKLMSSARQLINTVHlDMLIVPL+D---.
  consensus/90%                      ITETLSGSGDLGQVLVDAIKKYDhDLVVCGHHQDFWSKLMSSARQLINTVHlDMLIVPL+D---.
  consensus/80%                      ITETLSGSGDLGQVLVDAIKKYDMDLVVCGHHQDFWSKLMSSARQLINTVHVDMLIVPLRDEE-.
  consensus/70%                      ITETLSGSGDLGQVLVDAIKKYDMDLVVCGHHQDFWSKLMSSARQLINTVHVDMLIVPLRDEE-.
```

```
#  Percent Identity  Matrix - created by Clustal2.1
#
#
```

1: Salmonella	100.00	99.31	99.31	98.61	97.22	97.22	96.53	97.92	97.22
2: Escherichia	99.31	100.00	100.00	99.31	96.53	97.92	97.22	97.22	97.92
3: Citrobacter	99.31	100.00	100.00	99.31	96.53	97.92	97.22	97.22	97.92
4: Klebsiella	98.61	99.31	99.31	100.00	95.17	98.62	96.55	95.86	98.62
5: Cronobacter	97.22	96.53	96.53	95.17	100.00	95.17	93.10	94.48	95.17
6: Enterobacter	97.22	97.92	97.92	98.62	95.17	100.00	95.17	94.48	100.00
7: Phytobacter	96.53	97.22	97.22	96.55	93.10	95.17	100.00	96.55	95.17
8: Kosakonia	97.92	97.22	97.22	95.86	94.48	94.48	96.55	100.00	94.48
9: Lelliottia	97.22	97.92	97.92	98.62	95.17	100.00	95.17	94.48	100.00

4. CspD

```
                      cov     pid   1 [        .         .         .         .         :         .         .      ] 76
1 Salmonella      100.0% 100.0%     METGTVKWFNNAKGFGFICPEGGGEDIFAHYSTIQMDGYRTLKAGQSVRFDVHQGPKGNHASVIVPIEAEAVA---
2 Escherichia     100.0%  90.5%     MEKGTVKWFNNAKGFGFICPEGGGEDIFAHYSTIQMDGYRTLKAGQSVQFDVHQGPKGNHASVIVPVEVEAAVA--
3 Citrobacter     100.0%  97.3%     METGTVKWFNNAKGFGFICPEGGGEDIFAHYSTIQMDGYRTLKAGQPVQFDVHQGPKGNHASVIVPIEAEAVA---
4 Klebsiella      100.0%  93.2%     MEMGTVKWFNNAKGFGFICPEGGGEDIFAHYSTIQMDGYRTLKAGQAVRFDVHQGPKGNHASVIVPVEAETAA---
5 Cronobacter     100.0%  89.5%     METGTVKWFNNAKGFGFICPEGGGEDIFAHYSTIQMDGYRTLKAGQVVRFDVHQGPKGNHASVIVPLEAEVAPAVA
6 Enterobacter    100.0%  95.9%     MEMGTVKWFNNAKGFGFICPEGGGEDIFAHYSTIQMDGYRTLKAGQSVRFDVHQGPKGNHASLIVPIEAETVA---
7 Phytobacter     100.0%  95.9%     METGTVKWFNNAKGFGFICPEGGGEDIFAHYSTIQMDGYRTLKAGQTVRFDVHQGPKGNHASLIVPVEAEAVA---
8 Kosakonia       100.0%  94.5%     METGTVKWFNNAKGFGFICPEGGGEDIFAHYSTIQMDGYRTLKAGQTVRFDVHQGPKGNHASVIVPVEVEAMA---
9 Lelliottia      100.0%  94.5%     METGIVKWFNNAKGFGFICPEGGGEDIFAHYSTIQMDGYRTLKAGQSVRFDVHQGPKGNHASLIVPLEAETVA---
  consensus/100%                    MEhGhVKWFNNAKGFGFICPEGGGEDIFAHYSTIQMDGYRTLKAGQsVpFDVHQGPKGNHASlIVPlEsEshs...
  consensus/90%                     MEhGhVKWFNNAKGFGFICPEGGGEDIFAHYSTIQMDGYRTLKAGQsVpFDVHQGPKGNHASlIVPlEsEshs...
  consensus/80%                     MEhGTVKWFNNAKGFGFICPEGGGEDIFAHYSTIQMDGYRTLKAGQsVpFDVHQGPKGNHASlIVPlEsEsss...
  consensus/70%                     MEpGTVKWFNNAKGFGFICPEGGGEDIFAHYSTIQMDGYRTLKAGQsVRFDVHQGPKGNHASlIVPlEAEssA...
```

```
#  Percent Identity  Matrix - created by Clustal2.1
#
#
```

1: Salmonella	100.00	91.78	97.26	93.15	93.15	95.89	95.89	94.52	94.52
2: Escherichia	91.78	100.00	91.78	91.78	90.54	89.04	90.41	93.15	87.67
3: Citrobacter	97.26	91.78	100.00	91.78	91.78	93.15	94.52	93.15	91.78
4: Klebsiella	93.15	91.78	91.78	100.00	93.15	94.52	93.15	93.15	91.78
5: Cronobacter	93.15	90.54	91.78	93.15	100.00	90.41	91.78	91.78	91.78
6: Enterobacter	95.89	89.04	93.15	94.52	90.41	100.00	94.52	90.41	95.89
7: Phytobacter	95.89	90.41	94.52	93.15	91.78	94.52	100.00	95.89	94.52
8: Kosakonia	94.52	93.15	93.15	93.15	91.78	90.41	95.89	100.00	90.41
9: Lelliottia	94.52	87.67	91.78	91.78	91.78	95.89	94.52	90.41	100.00

5. Lon

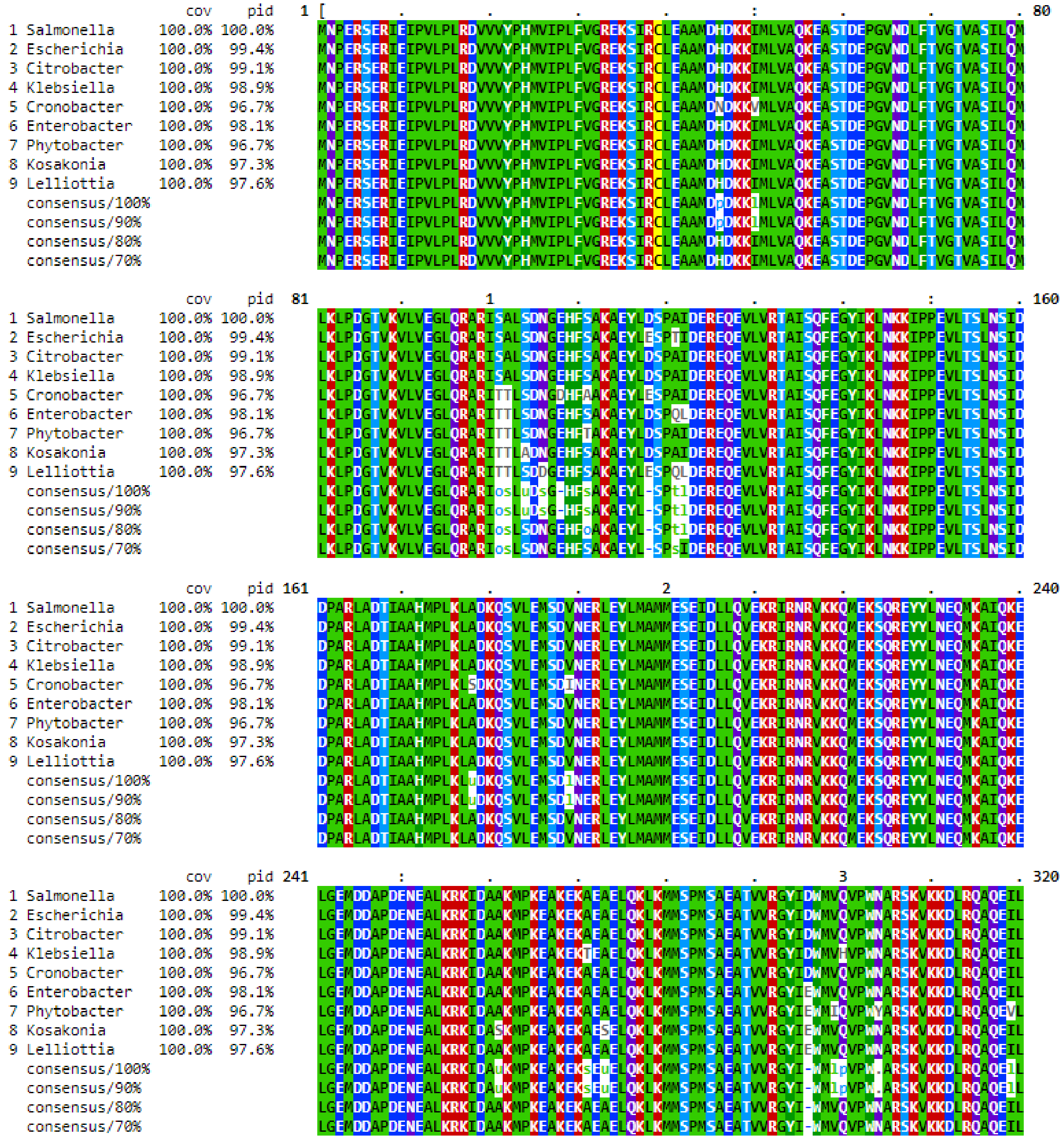
cov pid
1 Salmonella 100.0% 100.0%
2 Escherichia 100.0% 99.4%
3 Citrobacter 100.0% 99.1%
4 Klebsiella 100.0% 98.9%
5 Cronobacter 100.0% 96.7%
6 Enterobacter 100.0% 98.1%
7 Phytobacter 100.0% 96.7%
8 Kosakonia 100.0% 97.3%
9 Lelliottia 100.0% 97.6%
consensus/100%
consensus/90%
consensus/80%
consensus/70%

5. Lon (part 2)

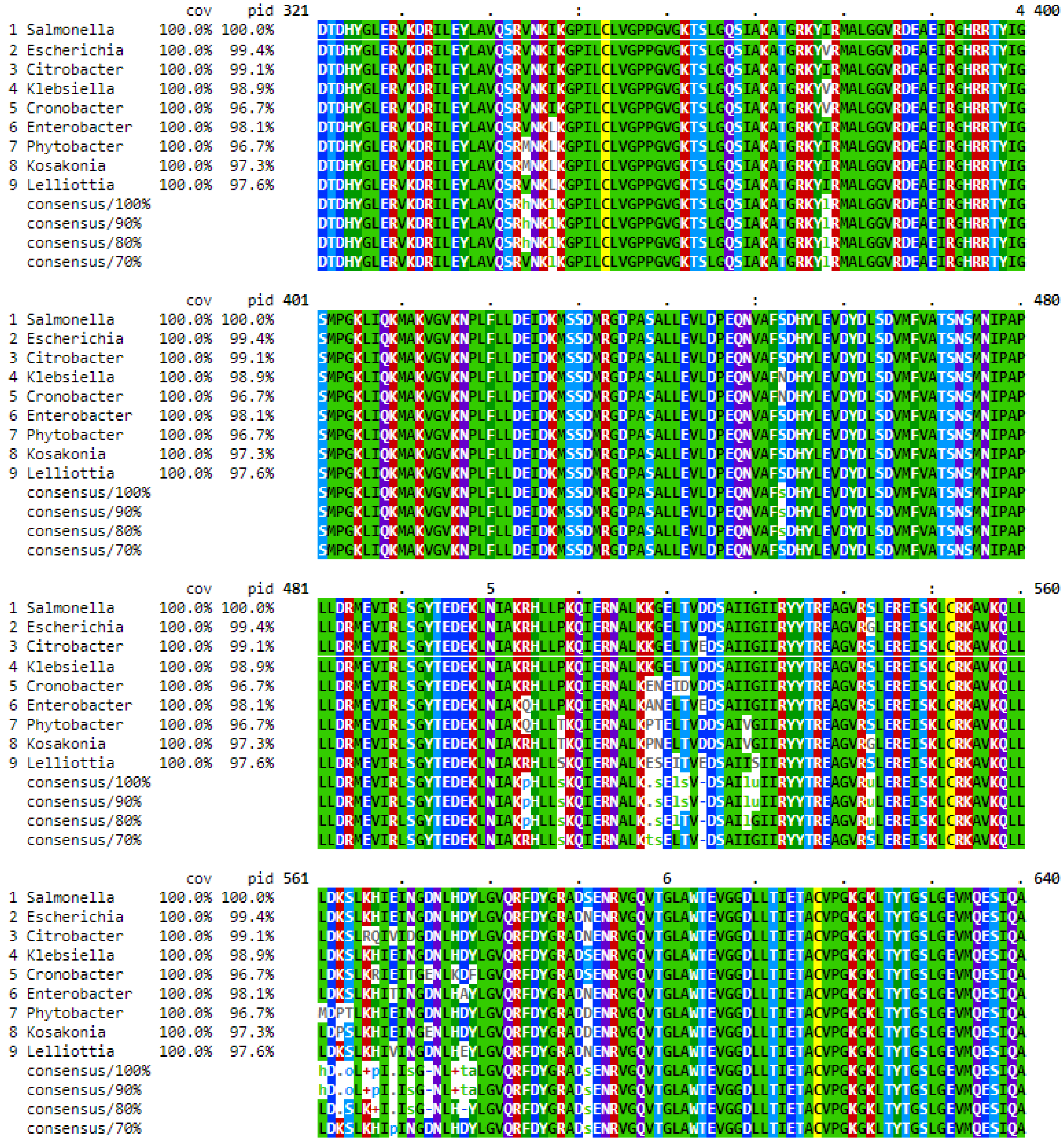
cov pid 321 400
1 Salmonella
2 Escherichia
3 Citrobacter
4 Klebsiella
5 Cronobacter
6 Enterobacter
7 Phytobacter
8 Kosakonia
9 Lelliottia
consensus/100%
consensus/90%
consensus/80%
consensus/70%
401 480
481 560
561 640

5. Lon (part 3)

```
                      cov     pid  641        :         .         .         .         .         7         .         . 720
1 Salmonella       100.0% 100.0%   ALTVVRARAEKLGINPDFYEKRDIHVHVPEGATPKDGPSAGIAMCTALVSCLTGNPVRADVAMTGEITLRGQVLPIGGLK
2 Escherichia      100.0%  99.4%   ALTVVRARAEKLGINPDFYEKRDIHVHVPEGATPKDGPSAGIAMCTALVSCLTGNPVRADVAMTGEITLRGQVLPIGGLK
3 Citrobacter      100.0%  99.1%   ALTVVRARAEKLGINPDFYEKRDIHVHVPEGATPKDGPSAGIAMCTALVSCLTGNPVRADVAMTGEITLRGQVLPIGGLK
4 Klebsiella       100.0%  98.9%   ALTVVRSRADKLGINADFYEKRDIHVHVPEGATPKDGPSAGIAMCTALVSCLTGNPVRADVAMTGEITLRGQVLPIGGLK
5 Cronobacter      100.0%  96.7%   ALTVVRARAEKLGINSDFYEKRDIHVHVPEGATPKDGPSAGIAMCTALVSCLTGNPVRADVAMTGEITLRGQVLPIGGLK
6 Enterobacter     100.0%  98.1%   ALTVVRARAEKLGINPDFYEKRDIHVHVPEGATPKDGPSAGIAMCTALVSCLTGNPVRADVAMTGEITLRGQVLPIGGLK
7 Phytobacter      100.0%  96.7%   ALTVVRARAEKLGINGDFYEKRDIHVHVPEGATPKDGPSAGIAMCTALVSCLTGNPVRADVAMTGEITLRGQVLPIGGLK
8 Kosakonia        100.0%  97.3%   ALTVVRARAEKLGINGDFYEKRDIHVHVPEGATPKDGPSAGIAMCTALVSCLTGNPVRADVAMTGEITLRGQVLPIGGLK
9 Lelliottia       100.0%  97.6%   ALTVVRARAEKLGINPDFYEKRDIHVHVPEGATPKDGPSAGIAMCTALVSCLTGNPVRADVAMTGEITLRGLVLPIGGLK
  consensus/100%                   ALTVVRuRA-KLGINsDFYEKRDIHVHVPEGATPKDGPSAGIAMCTALVSCLTGNPVRADVAMTGEITLRG.VLPIGGLK
  consensus/90%                    ALTVVRuRA-KLGINsDFYEKRDIHVHVPEGATPKDGPSAGIAMCTALVSCLTGNPVRADVAMTGEITLRG.VLPIGGLK
  consensus/80%                    ALTVVRARAEKLGINsDFYEKRDIHVHVPEGATPKDGPSAGIAMCTALVSCLTGNPVRADVAMTGEITLRGQVLPIGGLK
  consensus/70%                    ALTVVRARAEKLGINsDFYEKRDIHVHVPEGATPKDGPSAGIAMCTALVSCLTGNPVRADVAMTGEITLRGQVLPIGGLK
```

```
                      cov     pid  721        .         .         :         .         .         .    ] 784
1 Salmonella       100.0% 100.0%   EKLLAAHRGGIKTVLIPFENKRDLEEIPDNVIADLDIHPVKRIEEVLTLALQNEPSGMQVVTAK
2 Escherichia      100.0%  99.4%   EKLLAAHRGGIKTVLIPFENKRDLEEIPDNVIADLDIHPVKRIEEVLTLALQNEPSGMQVVTAK
3 Citrobacter      100.0%  99.1%   EKLLAAHRGGIKTVLIPYENKRDLEEIPDNVIADLDIHPVKRIEEVLTLALQNEPSGMQVVTAK
4 Klebsiella       100.0%  98.9%   EKLLAAHRGGIKTVLIPDENKRDLEEIPDNVIADLDIHPVKRIEEVLTLALQNEPFGMQVVTAK
5 Cronobacter      100.0%  96.7%   EKLLAAHRGGIKTVLIPDENKRDLEEIPENVIADLDIHPVKRIEEVLTLALQNAPYGMQVATAK
6 Enterobacter     100.0%  98.1%   EKLLAAHRGGIKTVLIPYENKRDLEEIPDNVIADLQIHPVKRIEEVLTLALQNEPSGMQVVTAK
7 Phytobacter      100.0%  96.7%   EKLLAAHRGGIKTVLIPYENKRDLEEIPENVIADLDIHPVKRIDEVLALALQNEPFGMQVATVK
8 Kosakonia        100.0%  97.3%   EKLLAAHRGGIKTVLIPYENKRDLEEIPDNVVADLDIHPVKRIEEVLALALQNEPFGMQVVTAK
9 Lelliottia       100.0%  97.6%   EKLLAAHRGGIKTVLIPHENKRDLEEIPDNVIADLDIHPVKRIEEVLTLALQNEPSGMQVVTAK
  consensus/100%                   EKLLAAHRGGIKTVLIP.ENKRDLEEIP-NVlADLpIHPVKRI-EVLsLALQNtP.GMQVsTsK
  consensus/90%                    EKLLAAHRGGIKTVLIP.ENKRDLEEIP-NVlADLpIHPVKRI-EVLsLALQNtP.GMQVsTsK
  consensus/80%                    EKLLAAHRGGIKTVLIP.ENKRDLEEIP-NVIADLDIHPVKRIEEVLsLALQNEP.GMQVsTAK
  consensus/70%                    EKLLAAHRGGIKTVLIPaENKRDLEEIPDNVIADLDIHPVKRIEEVLTLALQNEP.GMQVVTAK
```

Percent Identity Matrix - created by Clustal2.1
#
#

1: Salmonella	100.00	99.36	99.11	98.85	96.68	98.09	96.68	97.32	97.58
2: Escherichia	99.36	100.00	98.72	98.47	96.56	97.83	96.17	97.07	97.58
3: Citrobacter	99.11	98.72	100.00	98.09	96.17	98.09	96.17	96.81	97.58
4: Klebsiella	98.85	98.47	98.09	100.00	96.56	97.07	96.05	96.68	96.56
5: Cronobacter	96.68	96.56	96.17	96.56	100.00	95.79	95.15	95.54	95.79
6: Enterobacter	98.09	97.83	98.09	97.07	95.79	100.00	96.68	97.19	98.34
7: Phytobacter	96.68	96.17	96.17	96.05	95.15	96.68	100.00	97.70	95.92
8: Kosakonia	97.32	97.07	96.81	96.68	95.54	97.19	97.70	100.00	96.56
9: Lelliottia	97.58	97.58	97.58	96.56	95.79	98.34	95.92	96.56	100.00

Metabolism:

1. PurF

```
                       cov    pid   1 [         .         .         .         .         :         .         .         . 80
 1 Salmonella      100.0% 100.0%     MCGIVGIAGVMPVNQSIYDALTVLQHRGQDAAGIITIDANNCFRLRKANGLVNDIFEARHMQRLQGNMGIGHVRYPTAGS
 2 Escherichia     100.0%  96.2%     MCGIVGIAGVMPVNQSIYDALTVLQHRGQDAAGIITIDANNCFRLRKANGLVSDVFEARHMQRLQGNMGIGHVRYPTAGS
 3 Citrobacter     100.0%  97.4%     MCGIVGIAGVMPVNQSIYDALTVLQHRGQDAAGIITIDANNCFRLRKANGLVSDVFEARHMQRMQGNMGIGHVRYPTAGS
 4 Klebsiella      100.0%  97.0%     MCGIVGIAGVMPVNQSIYDALTVLQHRGQDAAGIITIDANNCFRLRKANGLVSDVFEARHMQRMQGNMGIGHVRYPTAGS
 5 Cronobacter     100.0%  95.0%     MCGIVGIAGVMPVNQSIYDALSVLQHRGQDAAGIITIDANNCFRLRKANGLVNDVFEARHMQRLQGNMGIGHVRYPTAGS
 6 Enterobacter    100.0%  96.4%     MCGIVGIAGFMPVNQSIYDALTVLQHRGQDAAGIITIDANNCFRLRKANGLVNDVFEARHMQRLQGNLGIGHVRYPTAGS
 7 Phytobacter     100.0%  97.0%     MCGIVGIAGVMPVNQSIYDALTVLQHRGQDAAGIITIDANNCFRLRKANGLVNDVFEARHMQRLQGNMGIGHVRYPTAGS
 8 Kosakonia       100.0%  96.4%     MCGIVGIAGFMPVNQSIYDALTVLQHRGQDAAGIITIDANNCFRLRKANGMVSDVFEARHMQRMQGNMGIGHVRYPTAGS
 9 Lelliottia      100.0%  96.0%     MCGIVGIAGFMPVNQSIYDALSVLQHRGQDAAGIITIDANNCFRLRKANGLVNDVFEARHMQRLQGNMGIGHVRYPTAGS
   consensus/100%                    MCGIVGIAGhMPVNQSIYDALoVLQHRGQDAAGIITIDANNCFRLRKANGhVsDlFEARHMQRhQGNhGIGHVRYPTAGS
   consensus/90%                     MCGIVGIAGhMPVNQSIYDALoVLQHRGQDAAGIITIDANNCFRLRKANGhVsDlFEARHMQRhQGNhGIGHVRYPTAGS
   consensus/80%                     MCGIVGIAGhMPVNQSIYDALoVLQHRGQDAAGIITIDANNCFRLRKANGLVsDVFEARHMQRhQGNMGIGHVRYPTAGS
   consensus/70%                     MCGIVGIAGhMPVNQSIYDALTVLQHRGQDAAGIITIDANNCFRLRKANGLVsDVFEARHMQRhQGNMGIGHVRYPTAGS

                       cov    pid  81           .         1         .         .         .         .         :         . 160
 1 Salmonella      100.0% 100.0%     SSASEAQPFYVNSPYGITLAHNGNLTNAHELRKKLFEEKRRHINTTSDSEILLNIFASELDNFRHYPLEADNIFAAIAAT
 2 Escherichia     100.0%  96.2%     SSASEAQPFYVNSPYGITLAHNGNLTNAHELRKKLFEEKRRHINTTSDSEILLNIFASELDNFRHYPLEADNIFAAIAAT
 3 Citrobacter     100.0%  97.4%     SSASEAQPFYVNSPYGITLAHNGNLTNAHELRKKLFEEKRRHINTTSDSEILLNIFASELDNFRHYPLEADNIFAAIAAT
 4 Klebsiella      100.0%  97.0%     SSASEAQPFYVNSPYGITLAHNGNLTNAHELRKKLFEEKRRHINTTSDSEILLNIFASELDNFRHYPLEADNIFAAIAAT
 5 Cronobacter     100.0%  95.0%     SSASEAQPFYVNSPYGITLAHNGNLTNAHELRKMLFEEKRRHINTTSDSEILLNIFASELDNFRHYPLEADNIFAAIAAM
 6 Enterobacter    100.0%  96.4%     SSASEAQPFYVNSPYGITLAHNGNLTNAHELRKKLFEEKRRHINTTSDSEILLNVFASELDNFRHYPLEADNIFAAVAAT
 7 Phytobacter     100.0%  97.0%     SSASEAQPFYVNSPYGITLAHNGNLTNAHELRKKLFEEKRRHINTTSDSEILLNIFASELDNFRHYPLEADNIFAAVAAT
 8 Kosakonia       100.0%  96.4%     SSASEAQPFYVNSPYGITLAHNGNLTNAHELRKKLFEEKRRHINTTSDSEILLNIFASELDNFRHYPLEADNIFAAIAAT
 9 Lelliottia      100.0%  96.0%     SSASEAQPFYVNSPYGITLAHNGNLTNAHELRKKLFEEKRRHINTTSDSEILLNIFASELDNFRHYPLEADNIFAAVAAT
   consensus/100%                    SSASEAQPFYVNSPYGITLAHNGNLTNAHELRKhLFEEKRRHINTTSDSEILLNlFASELDNFRHYPLEADNIFAAlAAh
   consensus/90%                     SSASEAQPFYVNSPYGITLAHNGNLTNAHELRKhLFEEKRRHINTTSDSEILLNlFASELDNFRHYPLEADNIFAAlAAh
   consensus/80%                     SSASEAQPFYVNSPYGITLAHNGNLTNAHELRKKLFEEKRRHINTTSDSEILLNIFASELDNFRHYPLEADNIFAAlAAT
   consensus/70%                     SSASEAQPFYVNSPYGITLAHNGNLTNAHELRKKLFEEKRRHINTTSDSEILLNIFASELDNFRHYPLEADNIFAAlAAT

                       cov    pid 161           .         .         .         2         .         .         .         . 240
 1 Salmonella      100.0% 100.0%     NRQIRGAYACVAMIIGHGMVAFRDPHGIRPLVLGKRDVGDGRTEYMVASESVALDTLGFEFLRDVAPGEAIYITEKGQLF
 2 Escherichia     100.0%  96.2%     NRLIRGAYACVAMIIGHGMVAFRDPNGIRPLVLGKRDIDENRTEYMVASESVALDTLGFDFLRDVAPGEAIYITEEGQLF
 3 Citrobacter     100.0%  97.4%     NRQIRGAYACVAMIIGHGMIAFRDPNGIRPLVLGKRDIGDGRTEYMVASESVALDTLGFEFLRDVAPGEAVYITEKGQLF
 4 Klebsiella      100.0%  97.0%     NRLIRGAYACVAMIIGHGMVAFRDPNGIRPLVLGKRDVGDGRTEYMVASESVALDTLGFEFLRDVAPGEAVYITEKGQLY
 5 Cronobacter     100.0%  95.0%     NRLIRGAYACVAMIIGHGMVAFRDPNGIRPLVLGKRDAGNGRAEYMVASESVALDTLGFEFLRDVAPGEAVYISEKGQLF
 6 Enterobacter    100.0%  96.4%     NRQIRGAYACVAMIIGHGMVAFRDPNGIRPLVLGKRDLGDGRTEYMVASESVALDTLGFEFLRDVAPGEAVYITEKGQLF
 7 Phytobacter     100.0%  97.0%     NRQIRGAYACVAMIIGHGMVAFRDPNGIRPLVLGKRDIGDGRTEYMVASESVALDTLGFEFLRDIAPGEAVYITEKGQLF
 8 Kosakonia       100.0%  96.4%     NRQIRGAYACVAMIIGHGMVAFRDPNGIRPLVLGKREIGDGRTEYMVASESVALDTLGFEFLRDIAPGEAVYITEKGQLF
 9 Lelliottia      100.0%  96.0%     NRQIRGAYACVAMIIGHGMVAFRDPNGIRPLVLGKRDLGDGRTEYMVASESVALDTLGFEFLRDVAPGEAVYIAEKGQLF
   consensus/100%                    NR.IRGAYACVAMIIGHGMlAFRDPpGIRPLVLGKR-hspsRsEYMVASESVALDTLGF-FLRDlAPGEAlYIsEcGQLa
   consensus/90%                     NR.IRGAYACVAMIIGHGMlAFRDPpGIRPLVLGKR-hspsRsEYMVASESVALDTLGF-FLRDlAPGEAlYIsEcGQLa
   consensus/80%                     NR.IRGAYACVAMIIGHGMVAFRDPNGIRPLVLGKRDlG-GRTEYMVASESVALDTLGFEFLRDlAPGEAlYIoEKGQLF
   consensus/70%                     NR.IRGAYACVAMIIGHGMVAFRDPNGIRPLVLGKRDlGDGRTEYMVASESVALDTLGFEFLRDVAPGEAVYITEKGQLF

                       cov    pid 241           :         .         .         .         .         3         .         . 320
 1 Salmonella      100.0% 100.0%     TRQCADNPVSNPCLFEYVYFARPDSFIDKISVYSARVNMGTKLGEKIAREWEDLDIDVVIPIPETSCDIALEIARILGKP
 2 Escherichia     100.0%  96.2%     TRQCADNPVSNPCLFEYVYFARPDSFIDKISVYSARVNMGTKLGEKIAREWEDLDIDVVIPIPETSCDIALEIARILGKP
 3 Citrobacter     100.0%  97.4%     TRQCAENPVSNPCLFEYVYFARPDSFIDKISVYSARVNMGTKLGEKIAREWEDLDIDVVIPIPETSCDIALEIARILGKP
 4 Klebsiella      100.0%  97.0%     TRQCADNPVSNPCLFEYVYFARPDSFIDKISVYSARVNMGTKLGEKIAREWEDLDIDVVIPIPETSCDIALEIARILDKP
 5 Cronobacter     100.0%  95.0%     TRQCADNPVSNPCLFEYVYFARPDSFIDKISVYSARVEMGKKLGEKIAREWEDLDIDVVIPIPETSCDIALEIARILNKP
 6 Enterobacter    100.0%  96.4%     TRQCADNPVSNPCLFEYVYFARPDSFIDKISVYSARVNMGTKLGEKIAREWDDLDIDVVIPIPETSCDIALEIARILDKP
 7 Phytobacter     100.0%  97.0%     TRQCADNPVSNPCLFEYVYFARPDSFIDKISVYSARVNMGTKLGEKIAREWEDLDIDVVIPIPETSCDIALEIARILGKP
 8 Kosakonia       100.0%  96.4%     SRQCADNPVSNPCLFEYVYFARPDSFIDKISVYSARVNMGTKLGEKIAREWEDLDIDVVIPIPETSCDIALEIARILGKP
 9 Lelliottia      100.0%  96.0%     TRQCADNPVSNPCLFEYVYFARPDSFIDKISVYSARVTMGTKLGEKIAREWDDLDIDVVIPIPETSCDIALEIARILDKP
   consensus/100%                    oRQCA-NPVSNPCLFEYVYFARPDSFIDKISVYSARVpMGpKLGEKIAREW-DLDIDVVIPIPETSCDIALEIARILsKP
   consensus/90%                     oRQCA-NPVSNPCLFEYVYFARPDSFIDKISVYSARVpMGpKLGEKIAREW-DLDIDVVIPIPETSCDIALEIARILsKP
   consensus/80%                     TRQCADNPVSNPCLFEYVYFARPDSFIDKISVYSARVsMGTKLGEKIAREW-DLDIDVVIPIPETSCDIALEIARILsKP
   consensus/70%                     TRQCADNPVSNPCLFEYVYFARPDSFIDKISVYSARVNMGTKLGEKIAREWEDLDIDVVIPIPETSCDIALEIARILsKP
```

2. PurF (part 2)

```
                           cov     pid 321         .         .         :         .         .         .         .         4 400
1 Salmonella          100.0% 100.0%    YRQGFVKNRYVGRTFIMPGQQLRRKSVRRKLNANRAEFRDKNVLLVDDSIVRGTTSEQIIEMAREAGAKKVYLASAAPEI
2 Escherichia         100.0%  96.2%    YRQGFVKNRYVGRTFIMPGQQLRRKSVRRKLNANRAEFRDKNVLLVDDSIVRGTTSEQIIEMAREAGAKKVYLASAAPEI
3 Citrobacter         100.0%  97.4%    YRQGFVKNRYVGRTFIMPGQQLRRKSVRRKLNANRAEFRDKNVLLVDDSIVRGTTSEQIIEMAREAGAKKVYLASAAPEI
4 Klebsiella          100.0%  97.0%    YRQGFVKNRYVGRTFIMPGQQLRRKSVRRKLNANRAEFRDKNVLLVDDSIVRGTTSEQIIEMAREAGAKKVYLASAAPEI
5 Cronobacter         100.0%  95.0%    YRQGFVKNRYVGRTFIMPGQHLRRKSVRRKLNANRAEFRDKNVLLVDDSIVRGTTSEQIIEMAREAGAKKVYLASAAPEI
6 Enterobacter        100.0%  96.4%    YRQGFVKNRYVGRTFIMPGQQLRRKSVRRKLNANRAEFRDKNVLLVDDSIVRGTTSEQIIEMAREAGAKKVYLASAAPEI
7 Phytobacter         100.0%  97.0%    YRQGFVKNRYVGRTFIMPGQQLRRKSVRRKLNANRAEFRDKNVLLVDDSIVRGTTSEQIIEMAREAGAKKVYLASAAPEI
8 Kosakonia           100.0%  96.4%    YRQGFVKNRYVGRTFIMPGQQLRRKSVRRKLNANRAEFRDKNVLLVDDSIVRGTTSEQIIEMAREAGAKKVYLASAAPEI
9 Lelliottia          100.0%  96.0%    YRQGFVKNRYVGRTFIMPGQQLRRKSVRRKLNANRAEFRDKNVLLVDDSIVRGTTSEQIIEMAREAGAKKVYLASAAPEI
  consensus/100%                       YRQGFVKNRYVGRTFIMPGQpLRRKSVRRKLNANRAEFRDKNVLLVDDSIVRGTTSEQIIEMAREAGAKKVYLASAAPEI
  consensus/90%                        YRQGFVKNRYVGRTFIMPGQpLRRKSVRRKLNANRAEFRDKNVLLVDDSIVRGTTSEQIIEMAREAGAKKVYLASAAPEI
  consensus/80%                        YRQGFVKNRYVGRTFIMPGQQLRRKSVRRKLNANRAEFRDKNVLLVDDSIVRGTTSEQIIEMAREAGAKKVYLASAAPEI
  consensus/70%                        YRQGFVKNRYVGRTFIMPGQQLRRKSVRRKLNANRAEFRDKNVLLVDDSIVRGTTSEQIIEMAREAGAKKVYLASAAPEI

                           cov     pid 401         .         .         .         .         :         .         .         . 480
1 Salmonella          100.0% 100.0%    RFPNVYGIDMPTANELIAHGREVDEIRQIIGADGLIFQDLNDLIEAVRAENPDIQQFECSVFNGIYVTKDVDQQYLDFLD
2 Escherichia         100.0%  96.2%    RFPNVYGIDMPSATELIAHGREVDEIRQIIGADGLIFQDLNDLIDAVRAENPDIQQFECSVFNGVYVTKDVDQGYLDFLD
3 Citrobacter         100.0%  97.4%    RFPNVYGIDMPTTNELIAHGREVDEIRQIIGADGLIFQDLNDLIEAVRAENPDIQQFECSVFNGVYVTKDVDQQYLDFLD
4 Klebsiella          100.0%  97.0%    RFPNVYGIDMPTANELIAHGREVDEIRQIIGADGLIFQDLNDLIDAVRAENPDIQQFECSVFNGVYVTRDVDQQYLDYLD
5 Cronobacter         100.0%  95.0%    RFPNVYGIDMPSANELIAHGREVDEIRQIIGADGLIFQDLDDLIEAVRAENPDIQQFECSVFNGVYVTKDVDHQYLEYLE
6 Enterobacter        100.0%  96.4%    RFPNVYGIDMPTANELIAHGREVDEIRQIIGADGLIFQDLNDLIDAVRAENPDIQQFECSVFNGVYVTKDVDQQYLDYLD
7 Phytobacter         100.0%  97.0%    RFPNVYGIDMPTANELIAHGREVDEIRQIIGADGLIFQDLTDLIDAVRAENPDIQQFECSVFNGIYVTKDVDQGYLDYLD
8 Kosakonia           100.0%  96.4%    RFPNVYGIDMPTANELIAHGREVDEIRQIIGADGLIFQDLNDLIDAVRAENPDIQQFECSVFNGVYVTKDVDQQYLDYLD
9 Lelliottia          100.0%  96.0%    RFPNVYGIDMPTANELIAHGREVDEIRQIIGADGLIFQDLDDLIEAVRAENPDIQQFECSVFNGIYVTKDVDQQYLDYLD
  consensus/100%                       RFPNVYGIDMPossELIAHGREVDEIRQIIGADGLIFQDLsDLI-AVRAENPDIQQFECSVFNGlYVT+DVDptYL-aL-
  consensus/90%                        RFPNVYGIDMPossELIAHGREVDEIRQIIGADGLIFQDLsDLI-AVRAENPDIQQFECSVFNGlYVT+DVDptYL-aL-
  consensus/80%                        RFPNVYGIDMPoANELIAHGREVDEIRQIIGADGLIFQDLsDLI-AVRAENPDIQQFECSVFNGlYVTKDVDQtYLDaLD
  consensus/70%                        RFPNVYGIDMPTANELIAHGREVDEIRQIIGADGLIFQDLsDLI-AVRAENPDIQQFECSVFNGlYVTKDVDQQYLDaLD

                           cov     pid 481         .         5    ] 505
1 Salmonella          100.0% 100.0%    SLRNDDAKAVLFQNEMENLEMHNEG
2 Escherichia         100.0%  96.2%    TLRNDDAKAVQRQNEVENLEMHNEG
3 Citrobacter         100.0%  97.4%    SLRNDDAKAVQRQNEVENLEMHNEG
4 Klebsiella          100.0%  97.0%    SLRNDDAKAVQLQNEVENLEMHNEG
5 Cronobacter         100.0%  95.0%    SLRNDDAKAVMRQNEVENLEMHNEG
6 Enterobacter        100.0%  96.4%    SLRNDDAKAVQLQNDLESLEMHNEG
7 Phytobacter         100.0%  97.0%    SLRNDDAKAVQMQNDLESLEMHNEG
8 Kosakonia           100.0%  96.4%    SLRNDDAKAVQLQNEVESLEMHNEG
9 Lelliottia          100.0%  96.0%    SLRNDDVKAIQMQNDLESLEMHNEG
  consensus/100%                       oLRNDDsKAl.hQN-hEsLEMHNEG
  consensus/90%                        oLRNDDsKAl.hQN-hEsLEMHNEG
  consensus/80%                        SLRNDDAKAV.hQN-lEsLEMHNEG
  consensus/70%                        SLRNDDAKAVQhQN-lEsLEMHNEG
```

```
#  Percent Identity  Matrix - created by Clustal2.1
#
#
```

1: Salmonella	100.00	96.24	97.43	97.03	95.05	96.44	97.03	96.44	96.04
2: Escherichia	96.24	100.00	96.83	96.63	94.65	95.25	96.04	96.04	94.06
3: Citrobacter	97.43	96.83	100.00	97.82	95.25	96.44	96.83	97.62	95.64
4: Klebsiella	97.03	96.63	97.82	100.00	95.45	97.23	96.83	97.82	95.84
5: Cronobacter	95.05	94.65	95.25	95.45	100.00	94.46	94.65	94.46	94.85
6: Enterobacter	96.44	95.25	96.44	97.23	94.46	100.00	97.82	97.23	97.82
7: Phytobacter	97.03	96.04	96.83	96.83	94.65	97.82	100.00	97.43	97.43
8: Kosakonia	96.44	96.04	97.62	97.82	94.46	97.23	97.43	100.00	95.84
9: Lelliottia	96.04	94.06	95.64	95.84	94.85	97.82	97.43	95.84	100.00

2. HisC

```
                       cov     pid   1 [         .         .         .         .         :         .         .         . 80
1 Salmonella        100.0% 100.0%    MSTENTLSVADLARENVRNLVPYQSARRLGGNGDVWLNANEFPTAVEFQLTQQTLNRYPECQPKAVIENYAQYAGVKPEQ
2 Escherichia        99.2%  88.8%    ---MSTVTITDLARENVRNLTPYQSARRLGGNGDVWLNANEYPTAVEFQLTQQTLNRYPECQPKAVIENYAQYAGVKPEQ
3 Citrobacter       100.0%  90.5%    MSTEKTFSVSELARENVRNLTPYQSARRLGGNGDVWLNANEFPTAVEFQLTAQTLNRYPECQPKAVIENYAQYAGVRPEQ
4 Klebsiella         98.3%  82.2%    ------MSIEDLARANVRALTPYQSARRLGGKGDVWLNANEFPTAVAFQLTAQTMNRYPEPQPKAVIESYARYADVKPEQ
5 Cronobacter        98.3%  82.2%    ------MSIEELARENVRNLTPYQSARRLGGNGDVWLNANEYPQAVEFQLTAQTLNRYPECQPKSVIERYAQYAGVKPEQ
6 Enterobacter       98.3%  85.3%    ------MNIEELARENVRRLTPYQSARRLGGNGDVWLNANEFPTAVQFELSQQTLNRYPECQPKAVIENYAQYAGVKPEQ
7 Phytobacter        98.3%  82.7%    ------MSIEELARANVRALTPYQSARRLGGKGDVWLNANEFPTAVEFNFTQQTLNRYPECQPKAVIEGYAQYAGLKPEQ
8 Kosakonia          98.3%  85.0%    ------MSIEELARKNVRELTPYQSARRLGGKGDVWLNANEFPTAVPFELTQQTLNRYPECQPKAVIESYAQYAGVKPEQ
9 Lelliottia         98.3%  85.8%    ------MSIEELARENVRRLTPYQSARRLGGNGDVWLNANEFPTAVSFALTQQTLNRYPECQPKAVIENYARYAGVKPEQ
  consensus/100%                     ......hslt-LARtNVRtLsPYQSARRLGGpGDVWLNANEaPpAV.FthotQThNRYPEsQPKuVIEtYApYAsl+PEQ
  consensus/90%                      ......hslt-LARtNVRtLsPYQSARRLGGpGDVWLNANEaPpAV.FthotQThNRYPEsQPKuVIEtYApYAsl+PEQ
  consensus/80%                      ......holp-LARtNVRtLTPYQSARRLGGpGDVWLNANEaPTAVtFpLTtQTLNRYPECQPKAVIEsYApYAGVKPEQ
  consensus/70%                      ......hSIp-LARcNVRpLTPYQSARRLGGpGDVWLNANEFPTAVpFpLTtQTLNRYPECQPKAVIEsYAQYAGVKPEQ
```

```
                       cov     pid  81         .         1         .         .         .         .         :         . 160
1 Salmonella        100.0% 100.0%    VLVSRGADEGIELVIRAFCEPGKDAILYCPPTYGMYSVSAETIGVERRTVPALENWQLDLQGISDNLDGTKVVFVCSPNN
2 Escherichia        99.2%  88.8%    VLVSRGADEGIELLIRAFCEPGKDAILYCPPTYGMYSVSAETIGVECRTVPTLDNWQLDLQGISDKLDGVKVVYVCSPNN
3 Citrobacter       100.0%  90.5%    VLVSRGADEGIELLIRAFCEPGKDAILYCPPTYGMYSVSAETIGVECRTVPTLADWQLDMQGIADKLDGVKVVYVCSPNN
4 Klebsiella         98.3%  82.2%    VLVSRGADEGIELLIRAFCEPGKDALLYCPPTYGMYSVSAETIGVECRTVPTLADWQLDLPGIEAQLDGVKVVFVCSPNN
5 Cronobacter        98.3%  82.2%    VLVSRGADEGIELLIRAFCEPGRDAVLYCPPTYGMYSVSAETIGVECRTVQATDGWQLDLPAIAGQLDGVKVIFVCSPNN
6 Enterobacter       98.3%  85.3%    VLVSRGADEGIELLIRAFCEPGKDAVMYCQPTYGMYSVSAETFGVACRNVLSLENWQLDLQGIADNLDGVKVVFVCSPNN
7 Phytobacter        98.3%  82.7%    VLVSRGADEGIELLIRAFCEPGKDAILFCPPTYGMYSVSAETFGVEYRTALTLDGWQLDLQNIADQLENVKLVYVCSPNN
8 Kosakonia          98.3%  85.0%    VLVSRGADEGIELLIRAFCEPGKDAVLFCPPTYGMYSVSAETLGVEYRTVPALSDWQLDLQGIADKLDGVKVIYVCSPNN
9 Lelliottia         98.3%  85.8%    VLVSRGADEGIELLIRAFCEPGKDAVMYCQPTYGMYSVSAETLGVECRNVLSLEDWQLDLPTITENLDGVKVVFVCSPNN
  consensus/100%                     VLVSRGADEGIELlIRAFCEPG+DAlhaC.PTYGMYSVSAEThGVthRss.shtsWQLDh.sIttpL-ssKllaVCSPNN
  consensus/90%                      VLVSRGADEGIELlIRAFCEPG+DAlhaC.PTYGMYSVSAEThGVthRss.shtsWQLDh.sIttpL-ssKllaVCSPNN
  consensus/80%                      VLVSRGADEGIELLIRAFCEPGKDAlhaC.PTYGMYSVSAEThGVEhRsV.sLtsWQLDL.sIsspLDGVKVlaVCSPNN
  consensus/70%                      VLVSRGADEGIELLIRAFCEPGKDAlLYCPPTYGMYSVSAETlGVEpRTV.sLpsWQLDL.uIu-pLDGVKVVaVCSPNN
```

```
                       cov     pid 161         .         .         .         2         .         .         .         . 240
1 Salmonella        100.0% 100.0%    PTGQLINPQDLRTLLELTRGKAIVVADEAYIEFCPQATLTGWLVEYPHLVILRTLSKAFALAGLRCGFTLANEEVINLLL
2 Escherichia        99.2%  88.8%    PTGQLINPQDFRTLLELTRGKAIVVADEAYIEFCPQASLAGWLAEYPHLAILRTLSKAFALAGLRCGFTLANEEVINLLM
3 Citrobacter       100.0%  90.5%    PTGQLINPQDLRTLLELTRGKAIVVADEAYIEFCPQATLAGWLADYPHLVVLRTLSKAFALAGLRCGFTLANEEVINLLL
4 Klebsiella         98.3%  82.2%    PTGQIIDPQSIRDLLEMTRDKAIVVADEAYIEFCPQATLAGWLSDYPHLVVVRTLSKAFALAGLRCGFTLANAEVINVLL
5 Cronobacter        98.3%  82.2%    PTGQLINPQDLRTLLEMARGKAIVVADEAYIEFCPQATLAGWLGEYPHLVVLRTLSKAFALAGLRCGFTLANEEVINLLL
6 Enterobacter       98.3%  85.3%    PTGQIINPQDIRTLLEMTRGKALVVADEAYIEFCPQATLAGWLEEYPHLVVLRTLSKAFALAGLRCGFTLANKAVIDLLL
7 Phytobacter        98.3%  82.7%    PTGQIINPQDIRAVLEMTRGKALVVADEAYIEFCPQATLAGWLEEYPHLVILRTLSKAFALAGLRCGFTLANEEVINLLM
8 Kosakonia          98.3%  85.0%    PTGQIINPQDFRVLLEMTRGKAIVVADEAYIEFCPQATLAGWISEYPNLVVLRTLSKAFALAGLRCGFTLANEDVIALLL
9 Lelliottia         98.3%  85.8%    PTGQIINPQDIRTLLELTRGKALVVADEAYIEFCPQATLAGWLEEYPHLVVLRTLSKAFALAGLRCGFTLANQEVINLLM
  consensus/100%                     PTGQlIsPQshRslLEhsRsKAlVVADEAYIEFCPQAoLsGWl.-YPpLsllRTLSKAFALAGLRCGFTLANttVIslLh
  consensus/90%                      PTGQlIsPQshRslLEhsRsKAlVVADEAYIEFCPQAoLsGWl.-YPpLsllRTLSKAFALAGLRCGFTLANttVIslLh
  consensus/80%                      PTGQlINPQDhRsLLEhTRGKAlVVADEAYIEFCPQATLAGWLt-YPHLVlLRTLSKAFALAGLRCGFTLANp-VIsLLh
  consensus/70%                      PTGQlINPQDlRsLLEhTRGKAlVVADEAYIEFCPQATLAGWLtEYPHLVlLRTLSKAFALAGLRCGFTLANcEVINLLh
```

```
                       cov     pid 241         :         .         .         .         .         3         .         . 320
1 Salmonella        100.0% 100.0%    KVIAPYPLSTPVADIAAQALCPQGINAMRDRVAQTVQERQYLVNALQQTACVEHVFDSETNYILARFTASSSVFKSLWDQ
2 Escherichia        99.2%  88.8%    KVIAPYPLSTPVADIAAQALSPQGIVAMRERVAQIIAEREYLIAALKEIPCVEQVFDSETNYILARFKASSAVFKSLWDQ
3 Citrobacter       100.0%  90.5%    KVIAPYPLSTPVADIAAQALSPQGINAMRERVAQILAERQYLVNALRDIPCVEQVFDSETNYILARFTASSSVFKSLWDQ
4 Klebsiella         98.3%  82.2%    KVIAPYPLSTPVADIAAQALSAEGIAAMRQRVAQILDERRYLVEQLRGIACVEQVFDSEANYVLARITASSAVFKSLWDQ
5 Cronobacter        98.3%  82.2%    KVIAPYPLSTPVADIAAQALSPEGIAAMRDRVAQVLANRDALIAGLRETPCVEAIFDSETNYVLARITASSAVFKSLWDQ
6 Enterobacter       98.3%  85.3%    KVIAPYPLSTPVADIAAQALTLQGINAMRERVAQILEERQYLVTALKAIPCVEQVFDSETNYLLVRFTASSAVFKSLWDQ
7 Phytobacter        98.3%  82.7%    KVIAPYPLSTPVADIAAQALSPEGIVAMRERVAQIVAEREYLSTSLRDIPCVETVFDSETNYILVRFKASSAVFKSLWDQ
8 Kosakonia          98.3%  85.0%    KVIAPYPLSTPVADIAAQALSTQGIVAMRERVAQILVERQYLVTALRDIPCVEQVFDSETNYILVRLTASSAVFKSLWDQ
9 Lelliottia         98.3%  85.8%    KVIAPYPLSTPVADIAAQALTPQGINAMRERVAQILLERQYLSNALKEIPCVEQVFESETNYILVRFTASSAVFKSLWDQ
  consensus/100%                     KVIAPYPLSTPVADIAAQALs.pGIsAMRpRVAQhl.pRphL.ttLpthsCVEtlF-SEsNYlLsRhpASSuVFKSLWDQ
  consensus/90%                      KVIAPYPLSTPVADIAAQALs.pGIsAMRpRVAQhl.pRphL.ttLpthsCVEtlF-SEsNYlLsRhpASSuVFKSLWDQ
  consensus/80%                      KVIAPYPLSTPVADIAAQALospGIsAMR-RVAQll.ERpYL.suL+thsCVEpVFDSETNYlLsRhpASSuVFKSLWDQ
  consensus/70%                      KVIAPYPLSTPVADIAAQALospGIsAMR-RVAQIltERpYLlsuL+pIPCVEpVFDSETNYlLsRhTASSAVFKSLWDQ
```

2. HisC (part 2)

```
                     cov    pid 321         .         .         :         .     ] 366
1 Salmonella      100.0% 100.0%     GIILRDQNKQPSLSGCLRITVGTRQENQRVIDALRAEPV-------
2 Escherichia      99.2%  88.8%     GIILRDQNKQPSLSGCLRITVGTREESQRVIDALRAEQV-------
3 Citrobacter     100.0%  90.5%     GIILRDQNKQPSLSGCLRITVGTREESQRVIDALRAEQV-------
4 Klebsiella       98.3%  82.2%     GIILRDQNKQPSLSGCLRITIGTRAESQRVIDALTAENV-------
5 Cronobacter      98.3%  82.2%     GIILRDQNRQPTLSGCLRITVGTREECQRVIDALRDQPGLAATERV
6 Enterobacter     98.3%  85.3%     GIILRDQNKQPSLSGCLRITVGTRSESQRLIDALKAEKV-------
7 Phytobacter      98.3%  82.7%     GIILRDQNKQPTLSGCLRITIGTREESQRVIDALRAESV-------
8 Kosakonia        98.3%  85.0%     GIILRDQNKQPSLSGCLRITIGTRAESQRVIDALRAEPV-------
9 Lelliottia       98.3%  85.8%     GIILRDQNKQPSLSGCLRITVGTREESQRVIDALKAEKV-------
  consensus/100%                    GIILRDQN+QPoLSGCLRITlGTRtEsQRlIDALpsp.s.......
  consensus/90%                     GIILRDQN+QPoLSGCLRITlGTRtEsQRlIDALpsp.s.......
  consensus/80%                     GIILRDQNKQPoLSGCLRITlGTRtEsQRVIDAL+AE.V.......
  consensus/70%                     GIILRDQNKQPSLSGCLRITlGTRpESQRVIDAL+AE.V.......
```

3. Icd (IcdA)

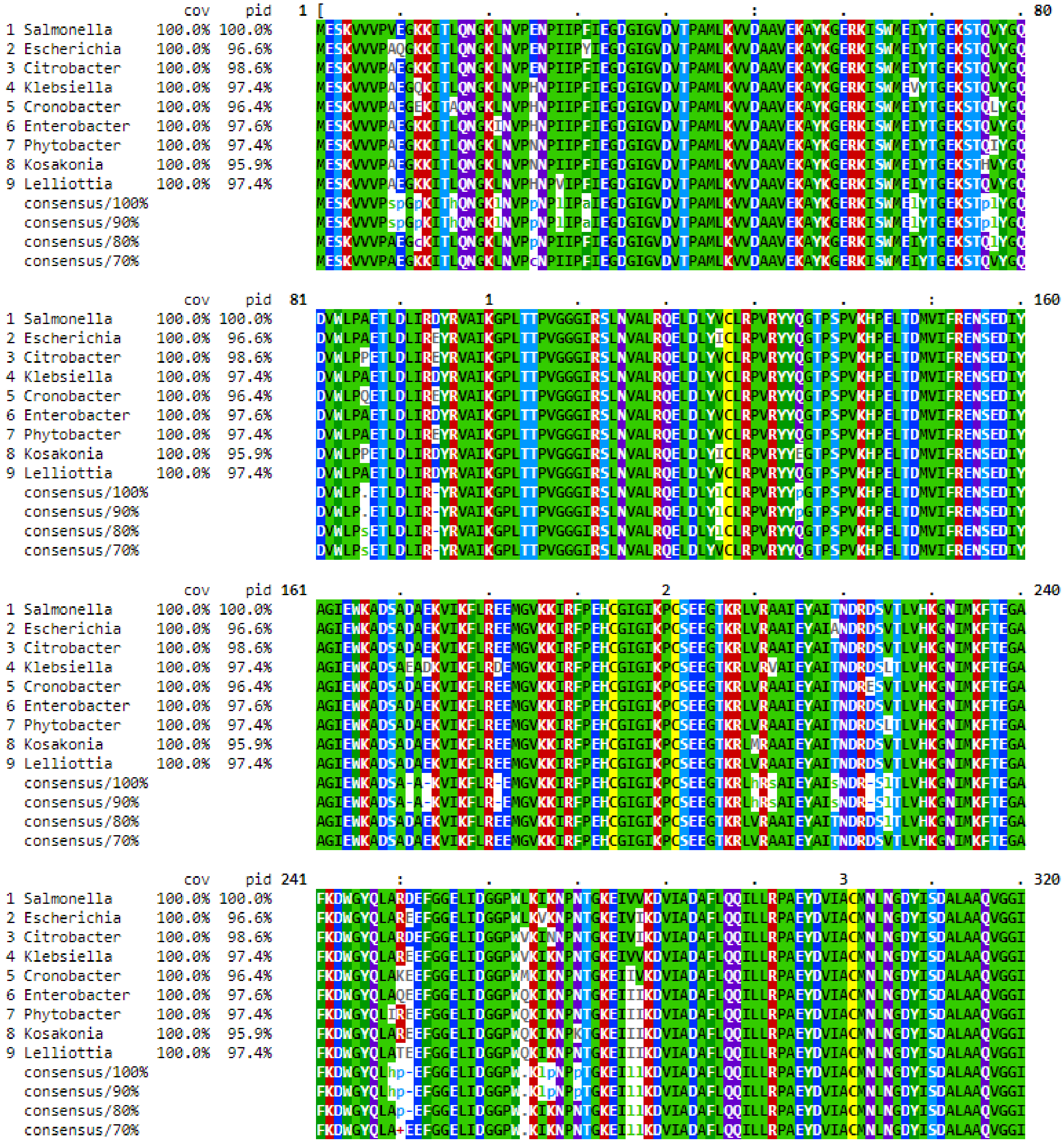

3. Icd (part 2)

```
                          cov    pid 321         .         .         :         .         .         .         .        4 400
1 Salmonella          100.0% 100.0%    GIAPGANIGDECALFEATHGTAPKYAGQDKVNPGSIILSAEMMLRHMQWFEAADLIVKGMEGAIAAKTVTYDFERLMEGA
2 Escherichia         100.0%  96.6%    GIAPGANIGDECALFEATHGTAPKYAGQDKVNPGSIILSAEMMLRHMGWTEAADLIVKGMEGAINAKTVTYDFERLMDGA
3 Citrobacter         100.0%  98.6%    GIAPGANIGDECALFEATHGTAPKYAGQDKVNPGSIILSAEMMLRHMQWFEAADLIVKGMEGAIAAKTVTYDFERLMEGA
4 Klebsiella          100.0%  97.4%    GIAPGANIGDECALFEATHGTAPKYAGQDKVNPGSIILSAEMMLRHMQWFEAADLIVKGMEGAIAAKTVTYDFERLMEGA
5 Cronobacter         100.0%  96.4%    GIAPGANIGDECALFEATHGTAPKYAGQDKVNPGSIILSAEMMLRHMEWFEAADLIVKGMEGAINNKTVTYDFERLMEGA
6 Enterobacter        100.0%  97.6%    GIAPGANIGDECALFEATHGTAPKYAGQDKVNPGSIILSAEMMLRHMEWFEAADLIVKGMEGAINAKTVTYDFERLMEGA
7 Phytobacter         100.0%  97.4%    GIAPGANIGDECALFEATHGTAPKYAGQDKVNPGSVILSAEMMLRHMQWFEAADLIVKGMEGAIAAKTVTYDFERLMEGA
8 Kosakonia           100.0%  95.9%    GIAPGANIGDECALFEATHGTAPKYAGQDKVNPGSVILSAEMMLRHMGWVEAADLIVKGMSGAINAKTVTYDFERLMEGA
9 Lelliottia          100.0%  97.4%    GIAPGANIGDECALFEATHGTAPKYAGQDKVNPGSIILSAEMMLRHMEWFEAADLIVKGMEGAINAKTVTYDFERLMDGA
  consensus/100%                       GIAPGANIGDECALFEATHGTAPKYAGQDKVNPGSlILSAEMMLRHMtWhEAADLIVKGMpGAIssKTVTYDFERLM-GA
  consensus/90%                        GIAPGANIGDECALFEATHGTAPKYAGQDKVNPGSlILSAEMMLRHMtWhEAADLIVKGMpGAIssKTVTYDFERLM-GA
  consensus/80%                        GIAPGANIGDECALFEATHGTAPKYAGQDKVNPGSlILSAEMMLRHMtWhEAADLIVKGMEGAIsAKTVTYDFERLM-GA
  consensus/70%                        GIAPGANIGDECALFEATHGTAPKYAGQDKVNPGSIILSAEMMLRHMpWFEAADLIVKGMEGAIsAKTVTYDFERLMEGA

                          cov    pid 401         .     ] 416
1 Salmonella          100.0% 100.0%    KLLKCSEFGDAIIANM
2 Escherichia         100.0%  96.6%    KLLKCSEFGDAIIENM
3 Citrobacter         100.0%  98.6%    KLLKCSEFGDAIIANM
4 Klebsiella          100.0%  97.4%    KLLKCSEFGDAIIANM
5 Cronobacter         100.0%  96.4%    KLLKCSEFGDAIIANM
6 Enterobacter        100.0%  97.6%    KLLKCSEFGDAIIANM
7 Phytobacter         100.0%  97.4%    KLLKCSEFGDAIIANM
8 Kosakonia           100.0%  95.9%    KLLKCSEFGDAIIANM
9 Lelliottia          100.0%  97.4%    KLLKCSEFGDAIIANM
  consensus/100%                       KLLKCSEFGDAIItNM
  consensus/90%                        KLLKCSEFGDAIItNM
  consensus/80%                        KLLKCSEFGDAIIANM
  consensus/70%                        KLLKCSEFGDAIIANM
```

```
#  Percent Identity  Matrix - created by Clustal2.1
#
#
```

1: Salmonella	100.00	96.63	98.56	97.36	96.39	97.60	97.36	95.91	97.36
2: Escherichia	96.63	100.00	96.63	94.95	95.19	96.39	95.91	95.67	96.63
3: Citrobacter	98.56	96.63	100.00	96.88	96.39	97.36	97.60	96.15	97.12
4: Klebsiella	97.36	94.95	96.88	100.00	95.67	96.63	96.63	94.71	96.39
5: Cronobacter	96.39	95.19	96.39	95.67	100.00	97.36	96.39	95.19	97.12
6: Enterobacter	97.60	96.39	97.36	96.63	97.36	100.00	97.60	96.88	99.04
7: Phytobacter	97.36	95.91	97.60	96.63	96.39	97.60	100.00	96.63	97.36
8: Kosakonia	95.91	95.67	96.15	94.71	95.19	96.88	96.63	100.00	96.63
9: Lelliottia	97.36	96.63	97.12	96.39	97.12	99.04	97.36	96.63	100.00

4. BioB

```
                    cov    pid   1 [         .         .         .         .         :         .         .         . 80
1 Salmonella     100.0% 100.0%    MARHPRWTLSQVTELFEKPLLELLFEAQQIHRQHFDPQQVQVSTLLSIKTGACPEDCKYCPQSSRYKTGLEAERLMEVEQ
2 Escherichia    100.0%  94.5%    MAHRPRWTLSQVTELFEKPLLDLLFEAQQVHRQHFDPRQVQVSTLLSIKTGACPEDCKYCPQSSRYKTGLEAERLMEVEQ
3 Citrobacter    100.0%  92.8%    MTNHSRWTLSQVTELFEKPLLELLFEAQQIHRQHFDPRQVQVSTLLSIKTGACPEDCKYCPQSARYKTGLEAERLMEVEQ
4 Klebsiella     100.0%  89.6%    MAHRARWTMSQVTELFNKPLIDLLFEAQQIHRQHFDPRQVQVSTLLSIKTGACPEDCKYCPQSARYKTGLETERLMEVEQ
5 Cronobacter    100.0%  90.2%    MAHLSRWTLSQVTELFEKPLLDLLFEAQQAHRQHFDPRQVQVSTLLSIKTGACPEDCKYCPQSARYKTGLEAERLMEVDQ
6 Enterobacter   100.0%  88.2%    MAHHARWTMSQVTELFNKPFLELMFEAQQVHRQHFDPRHVQVSTLLSIKTGACPEDCKYCPQSARYKTGLESERLMEVEQ
7 Phytobacter    100.0%  89.9%    MAHQTRWTLSQVTSLFEKPLLELLFEAQQVHRQHFDPRQVQVSTLLSIKTGACPEDCKYCPQSARYKTGLDAERLMEVEQ
8 Kosakonia      100.0%  90.5%    MAHHARWTMSQVTALFEKPLLELLFEAQQIHRQHFDPRQVQVSTLLSIKTGACPEDCKYCPQSARYKTGLESERLMEVEQ
9 Lelliottia     100.0%  89.6%    MAHHARWTMSQVTELFEKPFLELMFEAQQIHRQHFDPRHVQVSTLLSIKTGACPEDCKYCPQSARYKTGLEAERLMEVEQ
  consensus/100%                  Msp.sRWThSQVTtLFpKPhl-LhFEAQQhHRQHFDPppVQVSTLLSIKTGACPEDCKYCPQSuRYKTGL-sERLMEV-Q
  consensus/90%                   Msp.sRWThSQVTtLFpKPhl-LhFEAQQhHRQHFDPppVQVSTLLSIKTGACPEDCKYCPQSuRYKTGL-sERLMEV-Q
  consensus/80%                   MA+psRWThSQVTpLFpKPhL-LhFEAQQlHRQHFDPRpVQVSTLLSIKTGACPEDCKYCPQSuRYKTGLEuERLMEVEQ
  consensus/70%                   MAH+sRWThSQVTELFEKPLL-LLFEAQQlHRQHFDPRQVQVSTLLSIKTGACPEDCKYCPQSARYKTGLEuERLMEVEQ

                    cov    pid  81           .         1         .         .         .         .         :         . 160
1 Salmonella     100.0% 100.0%    VLDSARKAKNAGSTRFCMGAAWRNPHERDMPYLEKIVQGVKAMGLETCMTLGMLNESQAQRLANAGLDYYNHNLDTSPEF
2 Escherichia    100.0%  94.5%    VLESARKAKAAGSTRFCMGAAWKNPHERDMPYLEQMVQGVKAMGLEACMTLGTLSESQAQRLANAGLDYYNHNLDTSPEF
3 Citrobacter    100.0%  92.8%    VLDSARKAKNAGSTRFCMGAAWKNPHERDMPFLEQMVQGVKAMGLEACMTLGALSESQAQRLAAAGLDYYNHNLDTSPEF
4 Klebsiella     100.0%  89.6%    VLESARQAKNAGSTRFCMGAAWKNPNDRDMPYLEQMVKGVKALGLESCMTLGTLTDSQAQRLAGAGLDYYNHNLDTSPEF
5 Cronobacter    100.0%  90.2%    VLDSARKAKAAGSTRFCMGAAWKNPNDRDMPYLEQMVQGVKALGLETCMTLGTLSDDQAQRLGEAGLDYYNHNLDTSPEF
6 Enterobacter   100.0%  88.2%    VLESARKAKNAGSTRFCMGAAWKNPHDRDMPYLEQMVKGVKEMGLEACMTLGTLNEEQAQRLSAAGLDYYNHNLDTSPEF
7 Phytobacter    100.0%  89.9%    VLDSARKAKNAGSTRFCMGAAWKNPNDRDMPYLEQMVEGVKAMGLEACMTLGTLTDSQAQRLAHAGLDYYNHNLDTSPEF
8 Kosakonia      100.0%  90.5%    VLESARQAKRAGSTRFCMGAAWKNPHDRDMPYLEKMVEGVKAMGLEACMTLGTLNENQAQRLASAGLDYYNHNLDTSPEF
9 Lelliottia     100.0%  89.6%    VLDSARKAKNAGSTRFCMGAAWKNPHDRDMPYLEQMVQGVKAMGLEACMTLGTLDESQAQRLSAAGLDYYNHNLDTSPEF
  consensus/100%                  VL-SARpAKtAGSTRFCMGAAW+NPp-RDMPaLEphVpGVKthGLEsCMTLGhLs-pQAQRLutAGLDYYNHNLDTSPEF
  consensus/90%                   VL-SARpAKtAGSTRFCMGAAW+NPp-RDMPaLEphVpGVKthGLEsCMTLGhLs-pQAQRLutAGLDYYNHNLDTSPEF
  consensus/80%                   VL-SARpAKsAGSTRFCMGAAWKNPp-RDMPYLEpMVpGVKAhGLEsCMTLGsLs-sQAQRLutAGLDYYNHNLDTSPEF
  consensus/70%                   VL-SARKAKsAGSTRFCMGAAWKNPp-RDMPYLEQMVpGVKAMGLEuCMTLGTLs-sQAQRLusAGLDYYNHNLDTSPEF

                    cov    pid 161           .         .         .         2         .         .         .         . 240
1 Salmonella     100.0% 100.0%    YGNIITTRTYQERLDTLEKVREAGIKVCSGGIVGLGETVTDRAGLLLQLANLPTPPESVPINMLVKVKGTPLADNDDVDA
2 Escherichia    100.0%  94.5%    YGNIITTRTYQERLDTLEKVRDAGIKVCSGGIVGLGETVKDRAGLLLQLANLPTPPESVPINMLVKVKGTPLADNDDVDA
3 Citrobacter    100.0%  92.8%    YGNIITTRTYQERLDTLDKVRDAGIKVCSGGIVGLGETVKDRAGLLLQLANLPTPPESVPINMLVKVKGTPLADNDDVDA
4 Klebsiella     100.0%  89.6%    YGNIITTRTYQERLDTLDKVRDAGIKVCSGGIVGLGETVKDRAGLLLQLANLPTPPESVPINMLVKVKGTPLADNDDVDA
5 Cronobacter    100.0%  90.2%    YGNIITTRTYQERLDTLEKVREAGIKVCSGGIVGLGETVTDRAGLLLQLANLPTPPESVPINMLVKVKGTPLADNDDVDA
6 Enterobacter   100.0%  88.2%    YGNIITTRTYQERLDTLDKVREAGIKVCSGGIVGLGETVKDRAGLLLQLANLPTPPESVPINMLVKVKGTPLADNEDVDA
7 Phytobacter    100.0%  89.9%    YGNIITTRTYQERLDTLDKVRDAGIKVCSGGIVGLGETVTDRAGLLLQLANLPTPPESVPINMLVKVKGTPLAENEDVDA
8 Kosakonia      100.0%  90.5%    YGNIITTRTYQERLDTLDKVRDAGIKVCSGGIVGLGETVNDRAGLLLQLANLPTPPESVPINMLVKVKGTPLADNEDVDA
9 Lelliottia     100.0%  89.6%    YGNIITTRTYQERLDTLDKVREAGIKVCSGGIVGLGETVKDRAGLLLQLANLPTPPESVPINMLVKVKGTPMADNEDVDA
  consensus/100%                  YGNIITTRTYQERLDTL-KVR-AGIKVCSGGIVGLGETVpDRAGLLLQLANLPTPPESVPINMLVKVKGTPhA-N-DVDA
  consensus/90%                   YGNIITTRTYQERLDTL-KVR-AGIKVCSGGIVGLGETVpDRAGLLLQLANLPTPPESVPINMLVKVKGTPhA-N-DVDA
  consensus/80%                   YGNIITTRTYQERLDTL-KVR-AGIKVCSGGIVGLGETVpDRAGLLLQLANLPTPPESVPINMLVKVKGTPLADN-DVDA
  consensus/70%                   YGNIITTRTYQERLDTL-KVR-AGIKVCSGGIVGLGETVpDRAGLLLQLANLPTPPESVPINMLVKVKGTPLADN-DVDA

                    cov    pid 241           :         .         .         .         .         3         .         . 320
1 Salmonella     100.0% 100.0%    FDFIRTIAVARIMMPTSYVRLSAGREQMNEQTQAMCFMAGANSIFYGCKLLTTPNPAEDKDLQLFRKLGLNPQQTRVLAG
2 Escherichia    100.0%  94.5%    FDFIRTIAVARIMMPTSYVRLSAGREQMNEQTQAMCFMAGANSIFYGCKLLTTPNPEEDKDLQLFRKLGLNPQQTAVLAG
3 Citrobacter    100.0%  92.8%    FDFIRTIAVARIMMPASYVRLSAGREQMNEQTQAMCFMAGANSIFYGCKLLTTPNPEEDNDLRLFRKLGLNPQQTAVLAG
4 Klebsiella     100.0%  89.6%    FDFIRTIAIARIMMPTSYVRLSAGREQMNEQTQAMCFMAGANSIFYGCKLLTTPNPEEDKDLQLFRKLGINPQQTAVLEG
5 Cronobacter    100.0%  90.2%    FDFIRTIAVARIMMPTSYVRLSAGREQMNEQTQAMCFMAGANSIFYGCKLLTTPNPEEDKDLQLFRKLGINPQQTAVLAG
6 Enterobacter   100.0%  88.2%    FDFIRTIAVARIMMPTSYVRLSAGREQMNEQTQAMCFMAGANSIFYGCKLLTTPNPEEDKDVQLFRKLGLNPHQTEVLAG
7 Phytobacter    100.0%  89.9%    FDFIRTIAVARIMMPTSYVRLSAGREQMNEQTQAMCFMAGANSIFYGCKLLTTPNPEEDKDLQLFRKLGLNPQQTGVLNG
8 Kosakonia      100.0%  90.5%    FDFIRTIAVARIMMPTSHVRLSAGREQMNEQTQAMCFMAGANSIFYGCKLLTTPNPEEDKDLQLFRKLGLNPQQTAVLNG
9 Lelliottia     100.0%  89.6%    FDFIRTIAVARIMMPTSFVRLSAGREQMSEQTQAMCFMAGANSIFYGCKLLTTPNPEEDKDVQLFRKLGLNPHQTEVLTG
  consensus/100%                  FDFIRTIAlARIMMPsSaVRLSAGREQMsEQTQAMCFMAGANSIFYGCKLLTTPNPtEDpDlpLFRKLGlNPpQTtVLtG
  consensus/90%                   FDFIRTIAlARIMMPsSaVRLSAGREQMsEQTQAMCFMAGANSIFYGCKLLTTPNPtEDpDlpLFRKLGlNPpQTtVLtG
  consensus/80%                   FDFIRTIAVARIMMPTSaVRLSAGREQMNEQTQAMCFMAGANSIFYGCKLLTTPNPEEDKDlQLFRKLGlNPpQTtVLsG
  consensus/70%                   FDFIRTIAVARIMMPTSYVRLSAGREQMNEQTQAMCFMAGANSIFYGCKLLTTPNPEEDKDLQLFRKLGLNPQQTtVLsG
```

4. BioB (part 2)

```
                      cov     pid 321          .          .     ] 346
1 Salmonella      100.0% 100.0%     DNEQQQRLEQTLMTPDTDDYYNAAAL
2 Escherichia     100.0%  94.5%     DNEQQQRLEQALMTPDTDEYYNAAAL
3 Citrobacter     100.0%  92.8%     DNEQQQRLEQALRTPDTDAYYNAAAV
4 Klebsiella      100.0%  89.6%     DNEQQQRLEQALLTPDTEEYYNAAAL
5 Cronobacter     100.0%  90.2%     DNEQQARLEHALRDADNPQYYNAAAV
6 Enterobacter    100.0%  88.2%     DNEQQQQLEQQIFNADTEQFYNAASI
7 Phytobacter     100.0%  89.9%     DRAQQEQLEQQLVHADTDQYYNAAAL
8 Kosakonia       100.0%  90.5%     DNEQQQQLEQQLLHADTDQYYNAAAV
9 Lelliottia      100.0%  89.6%     DNEQQQQLEQQLFNADTDQYYNAATV
  consensus/100%                    DptQQtpLEptlhpsDs.taYNAAsl
  consensus/90%                     DptQQtpLEptlhpsDs.taYNAAsl
  consensus/80%                     DNEQQppLEQtLhpsDT-pYYNAAul
  consensus/70%                     DNEQQQpLEQtLhssDT-pYYNAAAl
```

```
#  Percent Identity  Matrix - created by Clustal2.1
#
#
```

1: Salmonella	100.00	94.51	92.77	89.60	90.17	88.15	89.88	90.46	89.60
2: Escherichia	94.51	100.00	94.51	93.35	92.49	89.88	91.62	91.91	90.46
3: Citrobacter	92.77	94.51	100.00	91.04	91.04	89.02	90.46	91.33	91.04
4: Klebsiella	89.60	93.35	91.04	100.00	91.04	89.60	90.46	91.62	89.02
5: Cronobacter	90.17	92.49	91.04	91.04	100.00	87.86	90.46	89.60	89.02
6: Enterobacter	88.15	89.88	89.02	89.60	87.86	100.00	89.60	91.91	95.09
7: Phytobacter	89.88	91.62	90.46	90.46	90.46	89.60	100.00	92.77	90.75
8: Kosakonia	90.46	91.91	91.33	91.62	89.60	91.91	92.77	100.00	92.49
9: Lelliottia	89.60	90.46	91.04	89.02	89.02	95.09	90.75	92.49	100.00

5. NuoC

```
                        cov    pid   1 [         .         .         .         .         :         .         .         . 80
1 Salmonella        100.0% 100.0%    MVNNMTDLTAQD---AAWSTRDHLDDPVIGELRNRFGPDAFTVQATRTGIPVVWVKREQLLEVGDFLKKLPKPYVMLFDL
2 Escherichia       100.0%  94.8%    MVNNMTDLTAQE---PAWQTRDHLDDPVIGELRNRFGPDAFTVQATRTGVPVVWIKREQLLEVGDFLKKLPKPYVMLFDL
3 Citrobacter       100.0%  96.7%    MVNTMTDLTAQD---AAWQTRDHLDDPVIGELRNRFGPDAFTVQATRTGVPVVWVKREQLLEVGDFLKKLPKPYVMLYDL
4 Klebsiella        100.0%  95.8%    MVNNMTDLTAHDA-APAWQTRDHLDDPVIGELRNRFGPDAFTVQPTRTGVPVVWVKREQLLEVGDFLKKLPKPYVMLFDL
5 Cronobacter       100.0%  92.2%    MVNNMTDLTAQDAVLPVWQTRDHLDDPVIGELRNRFGPDAFTVQATRTGVPVVWVKREQLLEVGEFLRKLPKPYVMLYDL
6 Enterobacter      100.0%  96.0%    MVNNMTDLTAQE---AAWQTRDHLDDPVIGELRNRFGPDAFTVQATRTGVPVVWVKREQLLEVVDFLKKLPKPYVMLFDL
7 Phytobacter       100.0%  97.8%    MVNNMTDLTAQE---ATWQTRDHLDDPVIGELRNRFGPDAFTVQATRTGVPVVWVKREQLLEVGDFLKKLPKPYVMLFDL
8 Kosakonia         100.0%  95.7%    MVNNMTDLTAQE---AAWQTRDHLDDPVIGELRNRFGPDAFTVQATRTGVPVVWVKREQLLEVVDFLKKLPKPYVMLFDL
9 Lelliottia         99.8%  95.2%    MVNNMTDLTAQA---A-SLTRDHLDDPVIGELRNRFGPDAFTVQATRTGVPVVWVKREQLLEVVDFLKKLPKPYVMLFDL
  consensus/100%                     MVNsMTDLTApt...s...TRDHLDDPVIGELRNRFGPDAFTVQsTRTGlPVVWlKREQLLEVs-FL+KLPKPYVMLaDL
  consensus/90%                      MVNsMTDLTApt...s...TRDHLDDPVIGELRNRFGPDAFTVQsTRTGlPVVWlKREQLLEVs-FL+KLPKPYVMLaDL
  consensus/80%                      MVNNMTDLTAQ-...ssWpTRDHLDDPVIGELRNRFGPDAFTVQATRTGVPVVWVKREQLLEVsDFLKKLPKPYVMLaDL
  consensus/70%                      MVNNMTDLTAQ-...ssWQTRDHLDDPVIGELRNRFGPDAFTVQATRTGVPVVWVKREQLLEVsDFLKKLPKPYVMLFDL

                        cov    pid  81         .         1         .         .         .         .         :         . 160
1 Salmonella        100.0% 100.0%    HGMDERLRTHRDGLPAADFSVFYHLISIERNRDIMLKVALSENDLRVPTFTKLFPNANWYERETWEMFGIDIEGHPHLTR
2 Escherichia       100.0%  94.8%    HGMDERLRTHREGLPAADFSVFYHLISIDRNRDIMLKVALAENDLHVPTFTKLFPNANWYERETWDLFGITFDGHPNLRR
3 Citrobacter       100.0%  96.7%    HGMDERLRTHREGLPAADFSVFYHLISIERNRDIMLKVALAENDLRVPTFTRLFPNANWYERETWEMFGIDIEGHPHLTR
4 Klebsiella        100.0%  95.8%    HGMDERLRTHRDGLPAADFSVFYHLISIDRNRDIMLKVALSENDLHLPTFTKLFPNANWYERETWEMFGMTFDGHPNLRR
5 Cronobacter       100.0%  92.2%    HGMDERLRTHRNGLPAADFSVFYHLLSIDRNRDIMLKVALSENDLNVPTFTRLFPNANWYERETWEMFGITFTGHPNLRR
6 Enterobacter      100.0%  96.0%    HGMDERLRTHRQGLPAADFSVFYHLISIDRNTDIMLKVALSENDMHLPTLTKLFPNANWYERETWEMFGMTFDGHPHLTR
7 Phytobacter       100.0%  97.8%    HGMDERLRTHRAGLPAADFSVFYHLISIERNRDIMLKVALSENDLRVPTFTKLFPNANWYERETWEMFGIDIEGHPHLTR
8 Kosakonia         100.0%  95.7%    HGMDERLRTHRAGLPAADFSVFYHFISIDRNRDIMLKVALAENDLNVPTITKLFPNANWYERETWEMFGVTFNGHPHLTR
9 Lelliottia         99.8%  95.2%    HGMDERLRTHRQGLPAADFSVFYHLISIDRNTDIMLKVALSENDMHLPTITKLFPNANWYERETWEMFGMTFDGHPHLTR
  consensus/100%                     HGMDERLRTHRtGLPAADFSVFYHhlSI-RNpDIMLKVALuENDhplPThT+LFPNANWYERETW-hFGhshpGHPpLpR
  consensus/90%                      HGMDERLRTHRtGLPAADFSVFYHhlSI-RNpDIMLKVALuENDhplPThT+LFPNANWYERETW-hFGhshpGHPpLpR
  consensus/80%                      HGMDERLRTHRtGLPAADFSVFYHLISI-RNpDIMLKVALuENDhplPThT+LFPNANWYERETWEMFGhshpGHPpLpR
  consensus/70%                      HGMDERLRTHRpGLPAADFSVFYHLISI-RNRDIMLKVALuENDL+lPThTKLFPNANWYERETWEMFGhsh-GHPpLpR

                        cov    pid 161         .         .         .         2         .         .         .         . 240
1 Salmonella        100.0% 100.0%    IMMPQTWEGHPLRKDYPARATEFDPFELTKAKQDLEMEALTFKPEDWGMKRGTDNEDFMFLNLGPNHPSAHGAFRIILQL
2 Escherichia       100.0%  94.8%    IMMPQTWKGHPLRKDYPARATEFSPFELTKAKQDLEMEALTFKPEEWGMKRGTENEDFMFLNLGPNHPSAHGAFRIVLQL
3 Citrobacter       100.0%  96.7%    IMMPNTWTGHPLRKDYPARATEFDPFELTKAKQDLEMEALTFKPEDWGMKRGTENEDFMFLNLGPNHPSAHGAFRIILQL
4 Klebsiella        100.0%  95.8%    IMMPPTWEGHPLRKDYPARATEFDPFELTKAKQDLEMEALTFKPEEWGMKRSTDNEDFMFLNLGPNHPSAHGAFRIILQL
5 Cronobacter       100.0%  92.2%    IMMPPTWEGHPLRKDYPARATEFDPFTLTKQKEELEMEALTFKPEEWGMKRGNDTEDFMFLNLGPNHPSAHGAFRIILQL
6 Enterobacter      100.0%  96.0%    IMMPQTWTGHPLRKDYPARATEFDPFELTKAKQDLEMEALTFKPEDWGMKRGTENEDFMFLNLGPNHPSAHGAFRIILQL
7 Phytobacter       100.0%  97.8%    IMMPQTWTGHPLRKDYPARATEFDPFELTKAKQDLEMEALKFKPEDWGMKRGTDNEDFMFLNLGPNHPSAHGAFRIILQL
8 Kosakonia         100.0%  95.7%    IMMPQTWTGHPLRKDYPARATEFDPFELTKAKQDLEMEALTFKPEDWGMKRGTENEDFMFLNLGPNHPSAHGAFRIILQL
9 Lelliottia         99.8%  95.2%    IMMPQTWTGHPLRKDYPARATEFDPFELTKAKQDLEMEALTFKPEDWGMKRGTENEDFMFLNLGPNHPSAHGAFRIILQL
  consensus/100%                     IMMP.TWpGHPLRKDYPARATEFsPFpLTKtKp-LEMEALpFKPE-WGMKRus-sEDFMFLNLGPNHPSAHGAFRIlLQL
  consensus/90%                      IMMP.TWpGHPLRKDYPARATEFsPFpLTKtKp-LEMEALpFKPE-WGMKRus-sEDFMFLNLGPNHPSAHGAFRIlLQL
  consensus/80%                      IMMP.TWpGHPLRKDYPARATEFDPFELTKAKQDLEMEALTFKPE-WGMKRGT-NEDFMFLNLGPNHPSAHGAFRIILQL
  consensus/70%                      IMMPpTWpGHPLRKDYPARATEFDPFELTKAKQDLEMEALTFKPE-WGMKRGT-NEDFMFLNLGPNHPSAHGAFRIILQL

                        cov    pid 241         :         .         .         .         .         3         .         . 320
1 Salmonella        100.0% 100.0%    DGEEIVDCVPDIGYHHRGAEKMGERQSWHSYIPYTDRIEYLGGCVNEMPYVLAVEKLAGITVPDRVNVIRVMLSELFRIN
2 Escherichia       100.0%  94.8%    DGEEIVDCVPDIGYHHRGAEKMGERQSWHSYIPYTDRIEYLGGCVNEMPYVLAVEKLAGITVPDRVNVIRVMLSELFRIN
3 Citrobacter       100.0%  96.7%    DGEEIVDCVPDIGYHHRGAEKMGERQSWHSYIPYTDRIEYLGGCVNEMPYVLAVEKLAGITVPDRVNVIRVMLSELFRIN
4 Klebsiella        100.0%  95.8%    DGEEIVDCVPDIGYHHRGAEKMGERQSWHSYIPYTDRIEYLGGCVNEMPYVLAVEKLAGITVPDRVNVIRVMLSELFRIN
5 Cronobacter       100.0%  92.2%    DGEEIVDCVPDIGYHHRGAEKMGERQSWHSYIPYTDRIEYLGGCVNEMPYVLAVEKLAGIQVPERVEVIRVMLSELFRIN
6 Enterobacter      100.0%  96.0%    DGEEIVDCVPDIGYHHRGAEKMGERQSWHSYIPYTDRIEYLGGCVNEMPYVLAVEKLAGITVPDRVNVIRVMLSELFRIN
7 Phytobacter       100.0%  97.8%    DGEEIVDCVPDIGYHHRGAEKMGERQSWHSYIPYTDRIEYLGGCVNEMPYVLAVEKLAGITVPDRVNVIRVMLSELFRIN
8 Kosakonia         100.0%  95.7%    DGEEIVDCVPDIGYHHRGAEKMGERQSWHSYIPYTDRIEYLGGCVNEMPYVLAVEKLAGITVPDRVNVIRVMLSELFRIN
9 Lelliottia         99.8%  95.2%    DGEEIVDCVPDIGYHHRGAEKMGERQSWHSYIPYTDRIEYLGGCVNEMPYVLAVEKLAGITTPDRVNVIRVMLSELFRIN
  consensus/100%                     DGEEIVDCVPDIGYHHRGAEKMGERQSWHSYIPYTDRIEYLGGCVNEMPYVLAVEKLAGIpsP-RVpVIRVMLSELFRIN
  consensus/90%                      DGEEIVDCVPDIGYHHRGAEKMGERQSWHSYIPYTDRIEYLGGCVNEMPYVLAVEKLAGIpsP-RVpVIRVMLSELFRIN
  consensus/80%                      DGEEIVDCVPDIGYHHRGAEKMGERQSWHSYIPYTDRIEYLGGCVNEMPYVLAVEKLAGITVPDRVNVIRVMLSELFRIN
  consensus/70%                      DGEEIVDCVPDIGYHHRGAEKMGERQSWHSYIPYTDRIEYLGGCVNEMPYVLAVEKLAGITVPDRVNVIRVMLSELFRIN
```

5. NuoC (part 2)

```
                         cov    pid  321        .         .         :         .         .         .         .        4 400
  1 Salmonella         100.0% 100.0%     SHLLYISTFIQDVGAMTPVFFAFTDRQKIYDLVEAITGFRMHPAWFRIGGVAHDLPRGWDRLLREFLEWMPKRLDSYEKA
  2 Escherichia        100.0%  94.8%     SHLLYISTFIQDVGAMTPVFFAFTDRQKIYDLVEAITGFRMHPAWFRIGGVAHDLPRGWDRLLREFLDWMPKRLASYEKA
  3 Citrobacter        100.0%  96.7%     SHLLYISTFIQDVGAMTPVFFAFTDRQKIYDLVEAITGYRMHPAWFRIGGVAHDLPRGWDRLLREFLDWMPKRLASYEKA
  4 Klebsiella         100.0%  95.8%     SHLLYISTFIQDVGAMTPVFFAFTDRQKIYDLVEAITGFRMHPAWFRIGGVAHDLPRGWDRLLREFLEWMPKRLDSYEKA
  5 Cronobacter        100.0%  92.2%     SHLLYISTFIQDVGAMTPVFFAFTDRQKIYDLVEAITGFRMHPAWFRIGGVAHDLPRGWDRLLKEFLEWMPKRLDSYVKA
  6 Enterobacter       100.0%  96.0%     SHLLYISTFIQDVGAMTPVFFAFTDRQKIYDLVEAITGFRMHPAWFRIGGVAHDLPRGWDRLLREFLDWMPKRLASYEKA
  7 Phytobacter        100.0%  97.8%     SHLLYISTFIQDVGAMTPVFFAFTDRQKIYDLVEAITGFRMHPAWFRIGGVAHDLPRGWDRLLREFLDWMPKRLASYEKA
  8 Kosakonia          100.0%  95.7%     SHLLYISTFIQDVGAMTPVFFAFTDRQKIYDLVEAITGFRMHPAWFRIGGVAHDLPRGWDRLLREFLDWMPKRLASYEKA
  9 Lelliottia          99.8%  95.2%     SHLLYISTFIQDVGAMTPVFFAFTDRQKIYDLVEAITGFRMHPAWFRIGGVAHDLPRGWDRLLREFLDWMPKRLASYEKA
    consensus/100%                       SHLLYISTFIQDVGAMTPVFFAFTDRQKIYDLVEAITGaRMHPAWFRIGGVAHDLPRGWDRLL+EFL-WMPKRLsSY.KA
    consensus/90%                        SHLLYISTFIQDVGAMTPVFFAFTDRQKIYDLVEAITGaRMHPAWFRIGGVAHDLPRGWDRLL+EFL-WMPKRLsSY.KA
    consensus/80%                        SHLLYISTFIQDVGAMTPVFFAFTDRQKIYDLVEAITGFRMHPAWFRIGGVAHDLPRGWDRLLREFL-WMPKRLsSYEKA
    consensus/70%                        SHLLYISTFIQDVGAMTPVFFAFTDRQKIYDLVEAITGFRMHPAWFRIGGVAHDLPRGWDRLLREFL-WMPKRLsSYEKA

                         cov    pid  401        .         .         .         .         :         .         .         . 480
  1 Salmonella         100.0% 100.0%     ALRNTILKGRSQGVAAYGAKEALEWGTTGAGLRATGIDFDVRKWRPYSGYENFDFEVPVGGGVSDCYTRVMLKVEELRQS
  2 Escherichia        100.0%  94.8%     ALQNTILKGRSQGVAAYGAKEALEWGTTGAGLRATGIDFDVRKARPYSGYENFDFEIPVGGGVSDCYTRVMLKVEELRQS
  3 Citrobacter        100.0%  96.7%     ALRNTILKGRSQGVAAYGGKEALEWGTTGAGLRAAGIDFDVRKARPYSGYENFDFEVPVGGGVSDCYTRVMLKVEELRQS
  4 Klebsiella         100.0%  95.8%     ALRNTILKGRSVGVAAYTAKEALEWGTTGAGLRATGIGFDVRKWRPYSGYENFDFEVPTGGGVSDCYTRVMLKVEELRQS
  5 Cronobacter        100.0%  92.2%     ALKNTILKGRSQGVAAYDAKEALAWGTTGAGLRATGIDFDVRKARPYSGYQNFEFEVPVGGGISDCYTRVMLKVEEVRQS
  6 Enterobacter       100.0%  96.0%     ALRNSILKGRSQGVAAYGAKEALEWGTTGAGLRATGIDFDVRKARPYSGYENFDFEVPVGGGVSDCYTRVMLKVEELRQS
  7 Phytobacter        100.0%  97.8%     ALRNTILKGRSQGVAAYGAKEALEWGTTGAGLRATGIDFDVRKARPYSGYENFDFEVPVGGGVSDCYTRVMLKVEELRQS
  8 Kosakonia          100.0%  95.7%     ALRNTILKGRSQGVAAYNAKEALEWGTTGAGLRATGINFDVRKARPYSGYENFDFEIPVGGGVSDCYTRVMLKVEELRQS
  9 Lelliottia          99.8%  95.2%     ALRNTILIGRSKGVAAYGAKEALEWGTTGAGLRATGIDFDVRKARPYSGYENFDFEVPVGGGVSDCYTRVMLKVEELRQS
    consensus/100%                       ALpNoILhGRS.GVAAYsuKEALtWGTTGAGLRAsGIsFDVRKhRPYSGYpNF-FElPsGGGlSDCYTRVMLKVEElRQS
    consensus/90%                        ALpNoILhGRS.GVAAYsuKEALtWGTTGAGLRAsGIsFDVRKhRPYSGYpNF-FElPsGGGlSDCYTRVMLKVEElRQS
    consensus/80%                        AL+NTILKGRSpGVAAYsAKEALEWGTTGAGLRATGIsFDVRKhRPYSGYENFDFElPVGGGVSDCYTRVMLKVEELRQS
    consensus/70%                        ALRNTILKGRSQGVAAYsAKEALEWGTTGAGLRATGIDFDVRKARPYSGYENFDFEVPVGGGVSDCYTRVMLKVEELRQS

                         cov    pid  481        .         5         .         .         .         .         :         . 560
  1 Salmonella         100.0% 100.0%     LRILQQCLDNMPEGPFKADHPLTTPPPKERTLQHIETLITHFLQVSWGPVMPAQESFQMVEATKGINSYYLTSDGSTMSY
  2 Escherichia        100.0%  94.8%     LRILEQCLNNMPEGPFKADHPLTTPPPKERTLQHIETLITHFLQVSWGPVMPANESFQMIEATKGINSYYLTSDGSTMSY
  3 Citrobacter        100.0%  96.7%     LRILEQCLNNMPEGPFKADHPLTTPPPKERTLQHIETLITHFLQVSWGPVMPANESFQMVEATKGINSYYLTSDGSTMSY
  4 Klebsiella         100.0%  95.8%     LRILQQCLDNMPEGPFKADHPLTTPPPKERTLQHIETLITHFLQVSWGPVMPANESFQMIEATKGINSYYLTSDGSTMSY
  5 Cronobacter        100.0%  92.2%     LRILEQCLKNMPEGPFKADHPLTTPPPKERTLQHIETLITHFLQVSWGPVMPANESFQMIEATKGINSYYLTSDGSTMSY
  6 Enterobacter       100.0%  96.0%     LRILEQCLNNMPEGPFKADHPLTTPPPKERTLQHIETLITHFLQVSWGPVMPAQESFQMIEATKGINSYYLTSDGSTMSY
  7 Phytobacter        100.0%  97.8%     LRILEQCLNNMPEGPFKADHPLTTPPPKERTLQHIETLITHFLQVSWGPVMPAQESFQMIEATKGINSYYLTSDGSTMSY
  8 Kosakonia          100.0%  95.7%     LRILEQCLNNMPEGPFKADHPLTTPPPKERTLQHIETLITHFLQVSWGPVMPANESFQMIEATKGINSYYLTSDGSTMSY
  9 Lelliottia          99.8%  95.2%     LRILEQCLNNMPEGPFKADHPLTTPPPKERTLQHIETLITHFLQVSWGPVMPAQESFQMIEATKGINSYYLTSDGSTMSY
    consensus/100%                       LRILpQCLpNMPEGPFKADHPLTTPPPKERTLQHIETLITHFLQVSWGPVMPApESFQMlEATKGINSYYLTSDGSTMSY
    consensus/90%                        LRILpQCLpNMPEGPFKADHPLTTPPPKERTLQHIETLITHFLQVSWGPVMPApESFQMlEATKGINSYYLTSDGSTMSY
    consensus/80%                        LRILpQCLsNMPEGPFKADHPLTTPPPKERTLQHIETLITHFLQVSWGPVMPApESFQMlEATKGINSYYLTSDGSTMSY
    consensus/70%                        LRILEQCLsNMPEGPFKADHPLTTPPPKERTLQHIETLITHFLQVSWGPVMPApESFQMIEATKGINSYYLTSDGSTMSY

                         cov    pid  561        .         .         .         6  ] 603
  1 Salmonella         100.0% 100.0%     RTRVRTPSFAHLQQIPSAIRGSLVSDLIVYLGSIDFVMSDVDR
  2 Escherichia        100.0%  94.8%     RTRVRTPSFAHLQQIPAAIRGSLVSDLIVYLGSIDFVMSDVDR
  3 Citrobacter        100.0%  96.7%     RTRVRTPSFAHLQQIPAAIRGSLVSDLIVYLGSIDFVMSDVDR
  4 Klebsiella         100.0%  95.8%     RTRVRTPSFAHLQQIPSAIRGSLVSDLIVYLGSIDFVMSDVDR
  5 Cronobacter        100.0%  92.2%     RTRVRTPSFPHLQQIPSVIRGSLVSDLIVYLGSIDFVMSDVDR
  6 Enterobacter       100.0%  96.0%     RTRVRTPSFAHLQQIPSAIRGSLVSDLIVYLGSIDFVMSDVDR
  7 Phytobacter        100.0%  97.8%     RTRVRTPSFAHLQQIPSAIRGSLVSDLIVYLGSIDFVMSDVDR
  8 Kosakonia          100.0%  95.7%     RTRVRTPSFAHLQQIPSAIRGSLVSDLIVYLGSIDFVMSDVDR
  9 Lelliottia          99.8%  95.2%     RTRVRTPSFAHLQQIPAAIRGSLVSDLIVYLGSIDFVMSDVDR
    consensus/100%                       RTRVRTPSFsHLQQIPusIRGSLVSDLIVYLGSIDFVMSDVDR
    consensus/90%                        RTRVRTPSFsHLQQIPusIRGSLVSDLIVYLGSIDFVMSDVDR
    consensus/80%                        RTRVRTPSFAHLQQIPuAIRGSLVSDLIVYLGSIDFVMSDVDR
    consensus/70%                        RTRVRTPSFAHLQQIPuAIRGSLVSDLIVYLGSIDFVMSDVDR
```

```
#  Percent Identity  Matrix - created by Clustal2.1
#
#
```

1: Salmonella	100.00	94.83	96.67	96.17	92.67	96.00	97.83	95.67	95.33
2: Escherichia	94.83	100.00	95.67	95.33	92.67	96.17	96.00	96.50	95.49
3: Citrobacter	96.67	95.67	100.00	94.00	92.17	95.83	97.33	96.17	95.33
4: Klebsiella	96.17	95.33	94.00	100.00	93.19	95.50	95.00	95.00	94.82
5: Cronobacter	92.67	92.67	92.17	93.19	100.00	92.00	92.67	92.33	91.32
6: Enterobacter	96.00	96.17	95.83	95.50	92.00	100.00	97.33	97.50	98.50
7: Phytobacter	97.83	96.00	97.33	95.00	92.67	97.33	100.00	97.17	96.49
8: Kosakonia	95.67	96.50	96.17	95.00	92.33	97.50	97.17	100.00	96.66
9: Lelliottia	95.33	95.49	95.33	94.82	91.32	98.50	96.49	96.66	100.00

6. ThiL

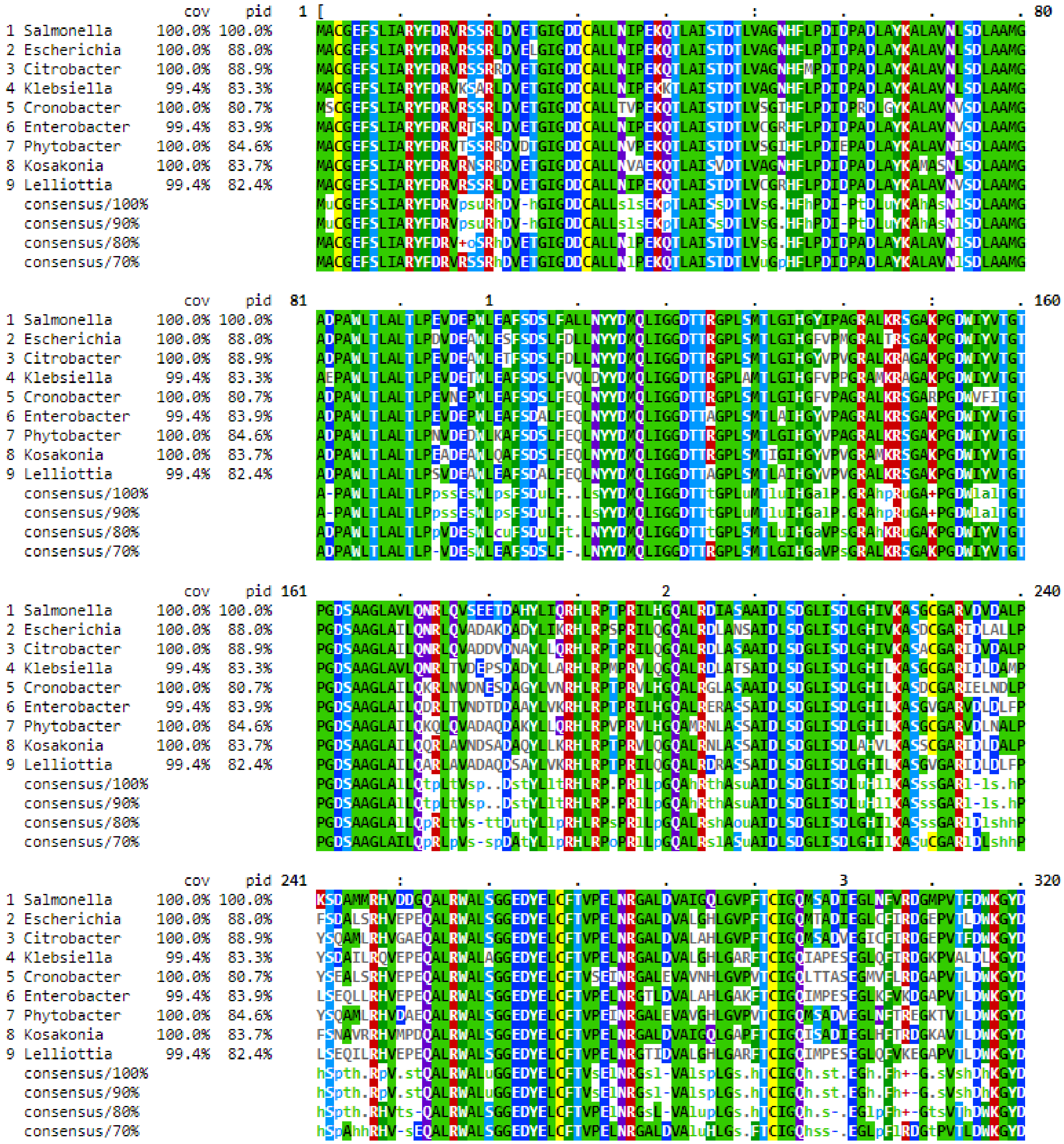
cov pid
1 Salmonella 100.0% 100.0%
2 Escherichia 100.0% 88.0%
3 Citrobacter 100.0% 88.9%
4 Klebsiella 99.4% 83.3%
5 Cronobacter 100.0% 80.7%
6 Enterobacter 99.4% 83.9%
7 Phytobacter 100.0% 84.6%
8 Kosakonia 100.0% 83.7%
9 Lelliottia 99.4% 82.4%
consensus/100%
consensus/90%
consensus/80%
consensus/70%

6. ThiL (part 2)

```
                   cov    pid 321      ] 326
1 Salmonella     100.0% 100.0%     HFATP-
2 Escherichia    100.0%  88.0%     HFATP-
3 Citrobacter    100.0%  88.9%     HFAAL-
4 Klebsiella      99.4%  83.3%     HFA---
5 Cronobacter    100.0%  80.7%     HFETHV
6 Enterobacter    99.4%  83.9%     HFG---
7 Phytobacter    100.0%  84.6%     HFAAL-
8 Kosakonia      100.0%  83.7%     HFAAH-
9 Lelliottia      99.4%  82.4%     HFG---
  consensus/100%                   HFt...
  consensus/90%                    HFt...
  consensus/80%                    HFu...
  consensus/70%                    HFu...
```

```
#  Percent Identity  Matrix - created by Clustal2.1
#
#

     1: Salmonella    100.00   88.00   88.92   83.28   80.92   83.90   84.62   83.69   82.35
     2: Escherichia    88.00  100.00   89.23   85.14   82.15   83.90   83.38   84.00   85.14
     3: Citrobacter    88.92   89.23  100.00   83.90   80.00   82.97   85.85   84.31   83.90
     4: Klebsiella     83.28   85.14   83.90  100.00   79.57   82.97   80.80   82.35   83.90
     5: Cronobacter    80.92   82.15   80.00   79.57  100.00   82.35   82.77   78.77   80.50
     6: Enterobacter   83.90   83.90   82.97   82.97   82.35  100.00   81.73   82.04   94.12
     7: Phytobacter    84.62   83.38   85.85   80.80   82.77   81.73  100.00   83.69   82.04
     8: Kosakonia      83.69   84.00   84.31   82.35   78.77   82.04   83.69  100.00   82.35
     9: Lelliottia     82.35   85.14   83.90   83.90   80.50   94.12   82.04   82.35  100.00
```

7. Cfa

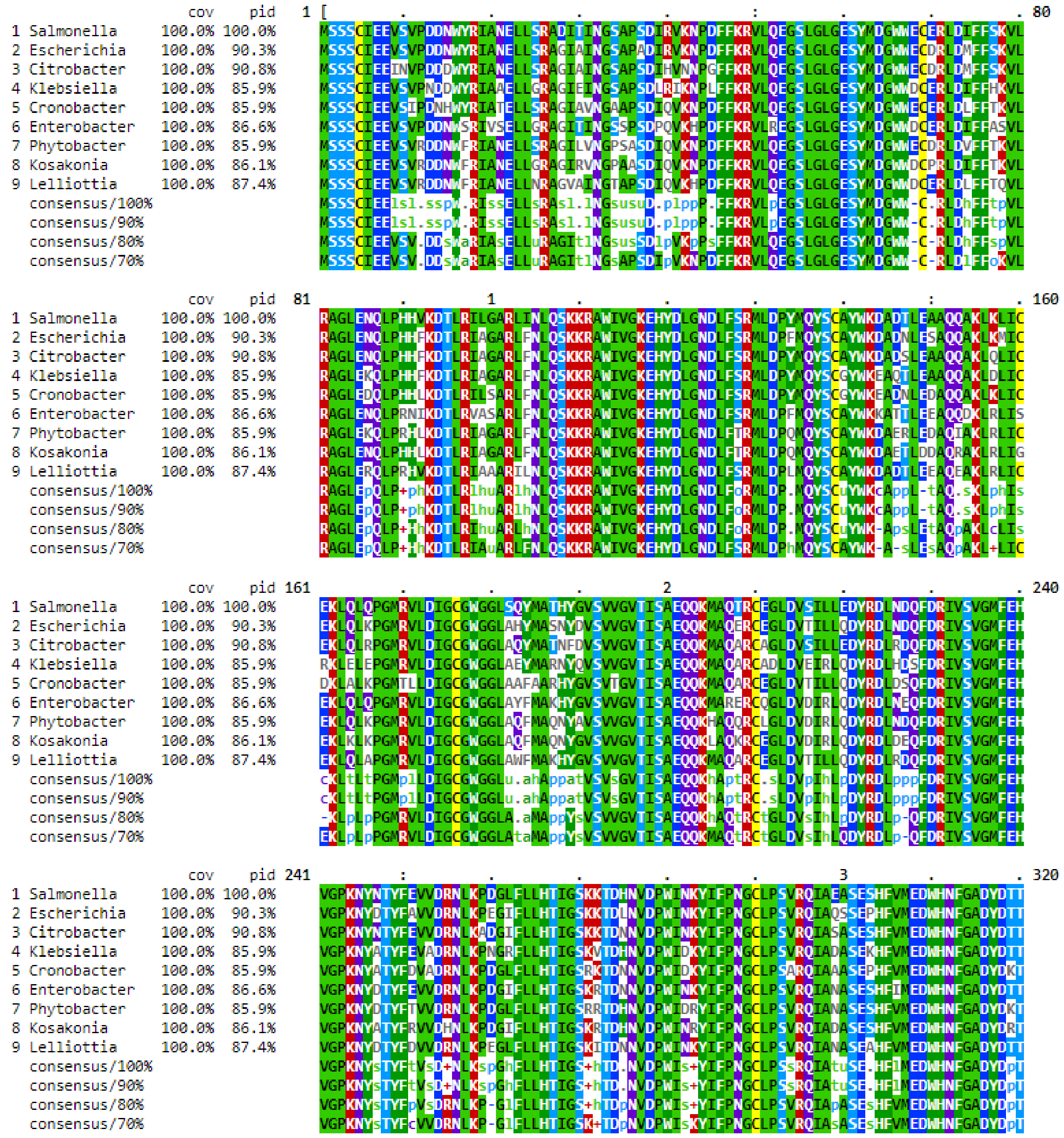
cov pid
1 Salmonella 100.0% 100.0%
2 Escherichia 100.0% 90.3%
3 Citrobacter 100.0% 90.8%
4 Klebsiella 100.0% 85.9%
5 Cronobacter 100.0% 85.9%
6 Enterobacter 100.0% 86.6%
7 Phytobacter 100.0% 85.9%
8 Kosakonia 100.0% 86.1%
9 Lelliottia 100.0% 87.4%
consensus/100%
consensus/90%
consensus/80%
consensus/70%

7. Cfa (part 2)

```
                      cov    pid 321        .         .         :         .         .         . ] 382
1 Salmonella     100.0% 100.0%     LMAWHERFINAWPEIAGNYNERFKRMFSYYLNACAGAFRARDIQLWQVVFTRGVENGLRVPR
2 Escherichia    100.0%  90.3%     LMAWYERFLAAWPEIADNYSERFKRMFTYYLNACAGAFRARDIQLWQVVFSRGVENGLRVAR
3 Citrobacter    100.0%  90.8%     LMAWYERFLAAWPQIADNYSERFKRMFTYYLNACAGAFRARDIQLWQVVFTRGVENGLRVAR
4 Klebsiella     100.0%  85.9%     LMAWYERFLASWPEIADNYSERFKRMFSYYLNACAGAFRARDIQLWQVVFSRGIEHGLRIAR
5 Cronobacter    100.0%  85.9%     LMAWYARFLDAWPEIADNYSERFKRMFTYYLNACAGAFRARDIQLWQVVFSRGVENGLRVAR
6 Enterobacter   100.0%  86.6%     LMAWHERFQQAWPEIADNYSERFKRMFSYYLNACAGAFRARDIQLWQVVFSRGIEHGLRVPR
7 Phytobacter    100.0%  85.9%     LMAWYERFLASWPEIADNYSERFKRMFSYYLNACAGAFRARDIQLWQVVFSRGIEHGLRVAR
8 Kosakonia      100.0%  86.1%     LMAWYERFLGAWPEIADNYSERFKRMFSYYLNACAGAFRARDIQLWQVVFSRGIEHGLRVAR
9 Lelliottia     100.0%  87.4%     LMAWYSRFLAAWPEIADNYSERFKRMFIYYLNACAGAFRARDIQLWQVVFSRGVENGLRVAR
  consensus/100%                   LMAWatRF.tuWPpIAsNYsERFKRMF.YYLNACAGAFRARDIQLWQVVFoRGlEpGLRlsR
  consensus/90%                    LMAWatRF.tuWPpIAsNYsERFKRMF.YYLNACAGAFRARDIQLWQVVFoRGlEpGLRlsR
  consensus/80%                    LMAWapRFlsuWPEIADNYSERFKRMFoYYLNACAGAFRARDIQLWQVVFoRGlEpGLRVsR
  consensus/70%                    LMAWYERFLsAWPEIADNYSERFKRMFoYYLNACAGAFRARDIQLWQVVFSRGlEpGLRVAR
```

```
#  Percent Identity  Matrix - created by Clustal2.1
#
#
```

1: Salmonella	100.00	90.31	90.84	85.86	85.86	86.65	85.86	86.13	87.43
2: Escherichia	90.31	100.00	91.88	86.65	87.43	85.60	87.70	86.91	89.01
3: Citrobacter	90.84	91.88	100.00	86.65	85.34	83.77	85.86	85.34	86.91
4: Klebsiella	85.86	86.65	86.65	100.00	84.55	84.03	85.34	85.60	84.29
5: Cronobacter	85.86	87.43	85.34	84.55	100.00	81.94	85.34	84.82	86.65
6: Enterobacter	86.65	85.60	83.77	84.03	81.94	100.00	85.86	86.65	86.91
7: Phytobacter	85.86	87.70	85.86	85.34	85.34	85.86	100.00	92.41	87.43
8: Kosakonia	86.13	86.91	85.34	85.60	84.82	86.65	92.41	100.00	86.91
9: Lelliottia	87.43	89.01	86.91	84.29	86.65	86.91	87.43	86.91	100.00

8. PykF

```
                     cov    pid   1 [         .         .         .         .         :         .         .         . 80
1 Salmonella      100.0% 100.0%     MKKTKIVCTIGPKTESEEMLSKMLDAGMNVMRLNFSHGDYAEHGQRIQNLRNVMSKTGKKAAILLDTKGPEIRTIKLEGG
2 Escherichia     100.0%  95.7%     MKKTKIVCTIGPKTESEEMLAKMLDAGMNVMRLNFSHGDYAEHGQRIQNLRNVMSKTGKTAAILLDTKGPEIRTMKLEGG
3 Citrobacter     100.0%  98.3%     MKKTKIVCTIGPKTESEEMLTKMLDAGMNVMRLNFSHGDYAEHGQRIQNLRNVMSKTGKKAAILLDTKGPEIRTIKLEGG
4 Klebsiella      100.0%  96.4%     MKKTKIVCTIGPKTESEEMLTKMLEAGMNVMRLNFSHGDYAEHGQRIQNLRNVMSKTGKKAAILLDTKGPEIRTIKLEGG
5 Cronobacter     100.0%  94.5%     MKKTKIVCTIGPKTESEEMLTKMLDAGMNVMRLNFSHGDYEEHGQRIKNLRNVLAKTGKQAAILLDTKGPEIRTIKLEGG
6 Enterobacter    100.0%  96.8%     MKKTKIVCTIGPKTESEEMLTKMLDAGMNVMRLNFSHGDYAEHGQRIQNLRNVMSKTGKKAAILLDTKGPEIRTIKLEGG
7 Phytobacter     100.0%  95.7%     MKKTKIVCTIGPKTESEEMLAKMLDAGMNVMRLNFSHGDYAEHGQRIQNLRNVMSKTGKKAAILLDTKGPEIRTIKLEGG
8 Kosakonia       100.0%  96.4%     MKKTKIVCTIGPKTESEEMLAKMLDAGMNVMRLNFSHGDYAEHGQRIKNLRNVMSKTGKKAAILLDTKGPEIRTIKLEGG
9 Lelliottia      100.0%  95.8%     MKKTKIVCTIGPKTESEEMLTKMLDAGMNVMRLNFSHGDYAEHGQRIKNLRNVMSKTGKKAAILLDTKGPEIRTIKLEGG
  consensus/100%                    MKKTKIVCTIGPKTESEEMLsKML-AGMNVMRLNFSHGDYtEHGQRIpNLRNVhuKTGKpAAILLDTKGPEIRThKLEGG
  consensus/90%                     MKKTKIVCTIGPKTESEEMLsKML-AGMNVMRLNFSHGDYtEHGQRIpNLRNVhuKTGKpAAILLDTKGPEIRThKLEGG
  consensus/80%                     MKKTKIVCTIGPKTESEEMLsKMLDAGMNVMRLNFSHGDYAEHGQRIpNLRNVMSKTGKpAAILLDTKGPEIRTIKLEGG
  consensus/70%                     MKKTKIVCTIGPKTESEEMLsKMLDAGMNVMRLNFSHGDYAEHGQRIpNLRNVMSKTGKKAAILLDTKGPEIRTIKLEGG
```

```
                     cov    pid  81           .         1         .         .         .         .         :         . 160
1 Salmonella      100.0% 100.0%     NDVSLKAGQTFTFTTDKSVVGNNEIVAVTYEGFTSDLSVGNTVLVDDGLIGMEVTAIEGNKVICKVLNNGDLGENKGVNL
2 Escherichia     100.0%  95.7%     NDVSLKAGQTFTFTTDKSVIGNSEMVAVTYEGFTTDLSVGNTVLVDDGLIGMEVTAIEGNKVICKVLNNGDLGENKGVNL
3 Citrobacter     100.0%  98.3%     NDVSLKAGQTFTFTTDKSVVGNNEIVAVTYEGFTSDLSVGNTVLVDDGLIGMEVTAIEGDKVICKVLNNGDLGENKGVNL
4 Klebsiella      100.0%  96.4%     NDVSLKAGQTFTFTTDKSVVGNNEIVAVTYEGFTSDLTVGNTVLVDDGLIGMEVTAIEGNKVICKVLNNGDLGENKGVNL
5 Cronobacter     100.0%  94.5%     NDVSLKAGQTFTFTTDKSVVGNSETVAVTYEGFTKDLSVGNTVLVDDGLIGMEVTAIEGDKVICKVLNNGDLGENKGVNL
6 Enterobacter    100.0%  96.8%     NDVSLKAGQTFTFTTDKSVVGNSEIVAVTYEGFTSDLSVGNTVLVDDGLIGMEVTAIEGNKVICKVLNNGDLGENKGVNL
7 Phytobacter     100.0%  95.7%     NDVSLKAGQTFTFTTDKTVIGNTETVAVTYEGFTSDLQVGNTVLVDDGLIGMEVTAIEGNKVICKVLNNGDLGENKGVNL
8 Kosakonia       100.0%  96.4%     NDVSLKAGQTFTFTTDKSVVGNADTVAVTYEGFTNDLSVGNTVLVDDGLIGMEVTEIVGNKVICKVLNNGDLGENKGVNL
9 Lelliottia      100.0%  95.8%     NDVSLKAGQTFTFTTDKTVVGNNEIVAVTYEGFTSDLSVGNTVLVDDGLIGMEVTAIEGNNVICKVLNNGDLGENKGVNL
  consensus/100%                    NDVSLKAGQTFTFTTDKoVlGNs-hVAVTYEGFTpDLpVGNTVLVDDGLIGMEVTtI.GspVICKVLNNGDLGENKGVNL
  consensus/90%                     NDVSLKAGQTFTFTTDKoVlGNs-hVAVTYEGFTpDLpVGNTVLVDDGLIGMEVTtI.GspVICKVLNNGDLGENKGVNL
  consensus/80%                     NDVSLKAGQTFTFTTDKoVlGNsEhVAVTYEGFTsDLoVGNTVLVDDGLIGMEVTAIEGsKVICKVLNNGDLGENKGVNL
  consensus/70%                     NDVSLKAGQTFTFTTDKSVVGNsEhVAVTYEGFToDLSVGNTVLVDDGLIGMEVTAIEGNKVICKVLNNGDLGENKGVNL
```

```
                     cov    pid 161           .         .         .         2         .         .         .         . 240
1 Salmonella      100.0% 100.0%     PGVSIALPALAEKDKQDLIFGCEQGVDFVAASFIRKRSDVVEIREHLKAHGGENIQIISKIENQEGLNNFDEILEASDGI
2 Escherichia     100.0%  95.7%     PGVSIALPALAEKDKQDLIFGCEQGVDFVAASFIRKRSDVIEIREHLKAHGGENIHIISKIENQEGLNNFDEILEASDGI
3 Citrobacter     100.0%  98.3%     PGVSIALPALAEKDKQDLIFGCEQGVDFVAASFIRKRSDVVEIREHLKAHGGENIQIISKIENQEGLNNFDEILEASDGI
4 Klebsiella      100.0%  96.4%     PGVSIALPALAEKDKQDLIFGCEQGVDFVAASFIRKRSDVVEIREHLKAHGGENIQIISKIENQEGLNNFDEILEASDGI
5 Cronobacter     100.0%  94.5%     PGVSIALPALAEKDKKDLIFGCEQGVDFVAASFIRKRSDVEEIRQHLKAHGGENIQIISKIENQEGLNNFDEILEASDGI
6 Enterobacter    100.0%  96.8%     PGVSIALPALAEKDKQDLIFGCEQGVDFVAASFIRKRSDVVEIREHLKAHGGEKIQIISKIENQEGLNNFDEILEASDGI
7 Phytobacter     100.0%  95.7%     PGVSIALPALAEKDKQDLIFGCEQGVDFVAASFIRKRSDVEEIRQHLKAHGGENIQIISKIENQEGLNNFDEILEASDGI
8 Kosakonia       100.0%  96.4%     PGVSIALPALAEKDKQDLIFGCEQGVDFVAASFIRKRSDVVEIREHLKAHGGENIQIISKIENQEGLNNFDEILEASDGI
9 Lelliottia      100.0%  95.8%     PGVSIALPALAEKDKQDLIFGCEQGVDFVAASFIRKRSDVVEIREHLKAHGGENIQIISKIENQEGLNNFDEILEASDGI
  consensus/100%                    PGVSIALPALAEKDKpDLIFGCEQGVDFVAASFIRKRSDV.EIRpHLKAHGGEpIpIISKIENQEGLNNFDEILEASDGI
  consensus/90%                     PGVSIALPALAEKDKpDLIFGCEQGVDFVAASFIRKRSDV.EIRpHLKAHGGEpIpIISKIENQEGLNNFDEILEASDGI
  consensus/80%                     PGVSIALPALAEKDKQDLIFGCEQGVDFVAASFIRKRSDV.EIRpHLKAHGGENIQIISKIENQEGLNNFDEILEASDGI
  consensus/70%                     PGVSIALPALAEKDKQDLIFGCEQGVDFVAASFIRKRSDVlEIREHLKAHGGENIQIISKIENQEGLNNFDEILEASDGI
```

```
                     cov    pid 241           :         .         .         .         .         3         .         . 320
1 Salmonella      100.0% 100.0%     MVARGDLGVEIPVEEVIFAQKMMIEKCIRARKVVITATQMLDSMIKNPRPTRAEAGDVANAILDGTDAVMLSGESAKGKY
2 Escherichia     100.0%  95.7%     MVARGDLGVEIPVEEVIFAQKMMIEKCIRARKVVITATQMLDSMIKNPRPTRAEAGDVANAILDGTDAVMLSGESAKGKY
3 Citrobacter     100.0%  98.3%     MVARGDLGVEIPVEEVIFAQKMMIEKCIRARKVVITATQMLDSMIKNPRPTRAEAGDVANAILDGTDAVMLSGESAKGKY
4 Klebsiella      100.0%  96.4%     MVARGDMGVEIPVEEVIFAQKMIIEKCIRARKVVITATQMLDSMIKNPRPTRAEAGDVANAILDGTDAVMLSGESAKGKY
5 Cronobacter     100.0%  94.5%     MVARGDLGVEIPVEEVIFAQKMMIEKCIRARKVVITATQMLDSMIKNPRPTRAEAGDVANAILDGTDAVMLSGESAKGKY
6 Enterobacter    100.0%  96.8%     MVARGDLGVEIPVEEVIFAQKMMIEKCVRARKVVITATQMLDSMIKNPRPTRAEAGDVANAILDGTDAVMLSGESAKGKY
7 Phytobacter     100.0%  95.7%     MVARGDLGVEIPVEEVIFAQKMMIEKCIRARKVVITATQMLDSMIKNPRPTRAEAGDVANAILDGTDAVMLSGESAKGKY
8 Kosakonia       100.0%  96.4%     MVARGDLGVEIPVEEVIFAQKMMIEKCIRARKVVITATQMLDSMIKNPRPTRAEAGDVANAILDGTDAVMLSGESAKGKY
9 Lelliottia      100.0%  95.8%     MVARGDLGVEIPVEEVIFAQKMMIEKCVRARKVVITATQMLDSMIKNPRPTRAEAGDVANAILDGTDAVMLSGESAKGKY
  consensus/100%                    MVARGDhGVEIPVEEVIFAQKMhIEKClRARKVVITATQMLDSMIKNPRPTRAEAGDVANAILDGTDAVMLSGESAKGKY
  consensus/90%                     MVARGDhGVEIPVEEVIFAQKMhIEKClRARKVVITATQMLDSMIKNPRPTRAEAGDVANAILDGTDAVMLSGESAKGKY
  consensus/80%                     MVARGDLGVEIPVEEVIFAQKMMIEKClRARKVVITATQMLDSMIKNPRPTRAEAGDVANAILDGTDAVMLSGESAKGKY
  consensus/70%                     MVARGDLGVEIPVEEVIFAQKMMIEKCIRARKVVITATQMLDSMIKNPRPTRAEAGDVANAILDGTDAVMLSGESAKGKY
```

8. PykF (part 2)

```
                       cov    pid 321         .         .         :         .         .         .         .       4 400
1 Salmonella        100.0% 100.0%     PLEAVSIMATICERTDRVMNSRLDYNNDSRKLRITEAVCRGAVETAEKLEAPLIVVATQGGKSARAVRKYFPDATILALT
2 Escherichia       100.0%  95.7%     PLEAVSIMATICERTDRVMNSRLEFNNDNRKLRITEAVCRGAVETAEKLDAPLIVVATQGGKSARAVRKYFPDATILALT
3 Citrobacter       100.0%  98.3%     PLEAVSIMATICERTDRVMSSRLDFNNDSRKLRITEAVCRGAVETAEKLEAPLIVVATQGGKSARAVRKYFPDATILALT
4 Klebsiella        100.0%  96.4%     PLEAVTIMATICERTDRVMTSRLDFNNDNRKLRITEAVCRGAVETAEKLEAPLIVVATQGGKSARAVRKYFPDATILALT
5 Cronobacter       100.0%  94.5%     PLEAVTIMATICERTDRVMTSRLEFNNDSRKLRITEAVCRGAVETAEKLEAPLIVVATEGGKSAKSVRKYFPDATILALT
6 Enterobacter      100.0%  96.8%     PLEAVSIMATICERTDRVMTSRLDFNNDSRKLRITEAVCRGAVETAEKLEAPLIVVATQGGKSARAVRKYFPDATILALT
7 Phytobacter       100.0%  95.7%     PLEAVTIMATICERTDRVMTSRLDFNNDSRKLRITEAVCRGAVETAEKLDAPLIVVATQGGKSARAVRKYFPDATILALT
8 Kosakonia         100.0%  96.4%     PLEAVTIMATICERTDRVMTSRLDFNNDSRKLRITEAVCRGAVETAEKLEAPLIVVATQGGKSARAVRKYFPDATILALT
9 Lelliottia        100.0%  95.8%     PLEAVTIMATICERTDRVMTSRLDNNNDSRKLRITEAVCRGAVETAEKLEAPLIVVATQGGKSAKAVRKYFPNATILALT
  consensus/100%                      PLEAVoIMATICERTDRVMsSRL-.NNDsRKLRITEAVCRGAVETAEKL-APLIVVATpGGKSA+uVRKYFPsATILALT
  consensus/90%                       PLEAVoIMATICERTDRVMsSRL-.NNDsRKLRITEAVCRGAVETAEKL-APLIVVATpGGKSA+uVRKYFPsATILALT
  consensus/80%                       PLEAVoIMATICERTDRVMsSRL-aNNDsRKLRITEAVCRGAVETAEKL-APLIVVATQGGKSA+AVRKYFPDATILALT
  consensus/70%                       PLEAVoIMATICERTDRVMoSRLDFNNDSRKLRITEAVCRGAVETAEKLEAPLIVVATQGGKSARAVRKYFPDATILALT

                       cov    pid 401         .         .         .         .         :         .        .] 473
1 Salmonella        100.0% 100.0%     TNEVTARQLVLSKGVVSQLVKEINSTDDFYRLGKDVAL---QSGLAQKGDVVVMVSGALVPSGTTNTASVHVL
2 Escherichia       100.0%  95.7%     TNEKTAHQLVLSKGVVPQLVKEITSTDDFYRLGKELAL---QSGLAHKGDVVVMVSGALVPSGTTNTASVHVL
3 Citrobacter       100.0%  98.3%     TNEVTARQLVLSKGVVAQMVKEITSTDDFYRLGKEVAL---QSGLAQKGDVVVMVSGALVPSGTTNTASVHVL
4 Klebsiella        100.0%  96.4%     TNETTARQLVLSKGVVPQLVEEIASTDDFYHLGKDLAL---KSGLARKGDVVVMVSGALVPSGTTNTASVHVL
5 Cronobacter       100.0%  94.5%     TNELTARQLVLSKGVVPQLVKEISSTDDFYRLGKDAAL---ESGLAKKGDVVVMVSGALVPSGTTNTASVHVL
6 Enterobacter      100.0%  96.8%     TNETTARQLVLSKGVVAHLVKEIASTDDFYIQGKELAL---ESGLAQKGDVVVMVSGALVPSGTTNTASVHVL
7 Phytobacter       100.0%  95.7%     TNETTARQLVLSKGVVAQLVEEIASTDDFYRLGKELAV---QSGLAQKGDVVVMVSGALVSSGTTNTASVHVL
8 Kosakonia         100.0%  96.4%     TNEVTARQLVLSKGVIPQLVKEISSTDDFYRLGKEVAL---ESGLASKGDVVVMVSGALVPSGTTNTASVHVL
9 Lelliottia        100.0%  95.8%     TNETTARQLVLSKGVVPHLVKEIASTDDFYRLGKEVALQLVDRGLAQKGDVVVMVSGALVPSGTTNTASVHVL
  consensus/100%                      TNEhTA+QLVLSKGVlsphVcEIsSTDDFYh.GK-hAl...ppGLApKGDVVVMVSGALVsSGTTNTASVHVL
  consensus/90%                       TNEhTA+QLVLSKGVlsphVcEIsSTDDFYh.GK-hAl...ppGLApKGDVVVMVSGALVsSGTTNTASVHVL
  consensus/80%                       TNEhTARQLVLSKGVVspLVcEIsSTDDFY+LGK-lAL...pSGLApKGDVVVMVSGALVPSGTTNTASVHVL
  consensus/70%                       TNEsTARQLVLSKGVVsQLVKEIsSTDDFYRLGK-lAL...pSGLApKGDVVVMVSGALVPSGTTNTASVHVL
```

```
#  Percent Identity  Matrix - created by Clustal2.1
#
#
```

1: Salmonella	100.00	95.74	98.30	96.38	94.47	96.81	95.74	96.38	96.38
2: Escherichia	95.74	100.00	95.74	94.68	93.83	95.11	95.32	94.89	93.83
3: Citrobacter	98.30	95.74	100.00	96.17	94.68	97.23	95.96	96.38	96.38
4: Klebsiella	96.38	94.68	96.17	100.00	94.04	96.38	95.74	95.32	95.74
5: Cronobacter	94.47	93.83	94.68	94.04	100.00	94.26	94.04	95.11	94.26
6: Enterobacter	96.81	95.11	97.23	96.38	94.26	100.00	95.96	95.74	96.81
7: Phytobacter	95.74	95.32	95.96	95.74	94.04	95.96	100.00	95.32	95.32
8: Kosakonia	96.38	94.89	96.38	95.32	95.11	95.74	95.32	100.00	95.74
9: Lelliottia	96.38	93.83	96.38	95.74	94.26	96.81	95.32	95.74	100.00

9. RpiA

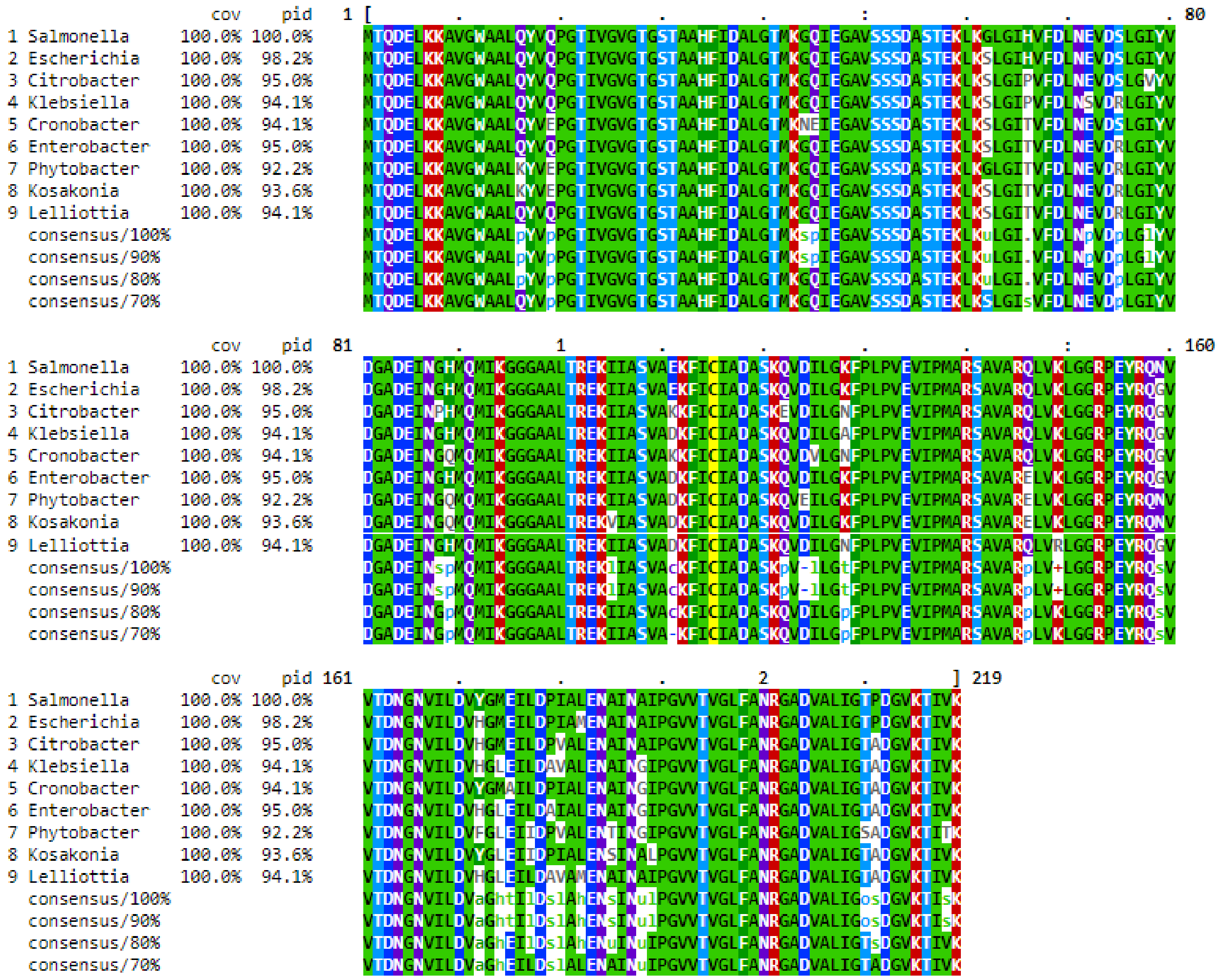

\# Percent Identity Matrix - created by Clustal2.1
\#
\#

1: Salmonella	100.00	98.17	94.98	94.06	94.06	94.98	92.24	93.61	94.06
2: Escherichia	98.17	100.00	95.89	94.98	94.06	95.89	90.87	92.69	95.89
3: Citrobacter	94.98	95.89	100.00	95.43	94.06	94.52	90.41	91.32	95.43
4: Klebsiella	94.06	94.98	95.43	100.00	93.15	97.72	92.69	92.69	97.26
5: Cronobacter	94.06	94.06	94.06	93.15	100.00	94.06	91.32	92.69	93.15
6: Enterobacter	94.98	95.89	94.52	97.72	94.06	100.00	94.06	94.98	97.26
7: Phytobacter	92.24	90.87	90.41	92.69	91.32	94.06	100.00	95.43	92.24
8: Kosakonia	93.61	92.69	91.32	92.69	92.69	94.98	95.43	100.00	93.15
9: Lelliottia	94.06	95.89	95.43	97.26	93.15	97.26	92.24	93.15	100.00

10. ThrC

```
                      cov    pid   1 [         .         .         .         .         :         .         .         . 80
 1 Salmonella      100.0% 100.0%    MKLYNLKDHNEQVSFAQAVTQGLGKQQGLFFPHDLPEFSLTEIDEMLNQDFVSRSAKILSAFIGDEIPQQILEERVRAAF
 2 Escherichia     100.0%  93.5%    MKLYNLKDHNEQVSFAQAVTQGLGKNQGLFFPHDLPEFSLTEIDEMLKLDFVTRSAKILSAFIGDEIPQEILEERVRAAF
 3 Citrobacter     100.0%  93.9%    MKLYNLKDHNEQVNFAQAITQGLGKNQGLFFPHDLPEFSLTEVDEMLSQDFVSRSAKILSAFIGDEIPQQILEERVRAAF
 4 Klebsiella       99.5%  90.1%    MKLYNLKDHNEQVSFAQAVTQGLGKHQGLFFPHDLPEFSLTEIDDMLAQDFVTRSAKILSAFIGDEIPQDVLQQRVRAAF
 5 Cronobacter     100.0%  86.7%    MKLYNLKDHNEQVSFAQAVTQGLGKNQGLFFPHDLPEFSLTEIDEMLEMDFVSRSSKILSAYIGDEIPADTLRQRVEKAF
 6 Enterobacter    100.0%  91.4%    MKLYNLKDHNEQVSFAQAVTQGLGKNQGLFFPHDLPEFQLTDIDELLKQDFVTRSTKILSAFIGDEIPQELLEERVRAAF
 7 Phytobacter     100.0%  90.2%    MKLYNLKDHNEQVSFAQAVTQGLGNNQGLFFPHDLPEFSLTEIDDMLTMDFVARSAKILGAFIGDEIPQDILEARVRDAF
 8 Kosakonia       100.0%  89.5%    MKLYNLKDHNEQVSFAQAVTQGLGKNQGLFFPHDLPEFNLTEVDEMLEQDFVTRSAKILAAFIGDEIPEEQLKARVRAAF
 9 Lelliottia      100.0%  90.7%    MKLYNLKDHNEQVSFAQAVTQGLGKNQGLFFPHDLPEFSLTEIDDMLKQDFVSRSAKIVSAFIGEEIPQELLEERIRTAF
   consensus/100%                   MKLYNLKDHNEQVsFAQAlTQGLGppQGLFFPHDLPEFpLT-lD-hLt.DFVsRSsKIluAaIG-EIPtp.LptRlctAF
   consensus/90%                    MKLYNLKDHNEQVsFAQAlTQGLGppQGLFFPHDLPEFpLT-lD-hLt.DFVsRSsKIluAaIG-EIPtp.LptRlctAF
   consensus/80%                    MKLYNLKDHNEQVSFAQAVTQGLGKpQGLFFPHDLPEFsLTElD-MLp.DFVoRSuKILuAFIGDEIPpphLctRVRsAF
   consensus/70%                    MKLYNLKDHNEQVSFAQAVTQGLGKNQGLFFPHDLPEFSLTEID-MLp.DFVoRSAKILSAFIGDEIPQ-lLcpRVRsAF

                      cov    pid  81         .         1         .         .         .         .         :         . 160
 1 Salmonella      100.0% 100.0%    AFPAPVAQVESDVGCLELFHGPTLAFKDFGGRFMAQMLTHISGDKPVTILTATSGDTGAAVAHAFYGLENVRVVILYPRG
 2 Escherichia     100.0%  93.5%    AFPAPVANVESDVGCLELFHGPTLAFKDFGGRFMAQMLTHIAGDKPVTILTATSGDTGAAVAHAFYGLPNVKVVILYPRG
 3 Citrobacter     100.0%  93.9%    AFPAPVAQVESDVGCLELFHGPTLAFKDFGGRFMAQMLTQISGDRPVTILTATSGDTGAAVAHAFYGLPNVRVVILYPRG
 4 Klebsiella       99.5%  90.1%    AFPAPVSKVQDDVGCLELFHGPTLAFKDFGGRFMAQMLTHIAGDKPVTILTATSGDTGAAVAHAFYGLPNVKVVILYPRG
 5 Cronobacter     100.0%  86.7%    TFPAPVSQVAEDIGCLELFHGPTLAFKDFGGRFMAQMLTQISGDKPVTILTATSGDTGAAVAHAFYGLKNVQVVILYPRG
 6 Enterobacter    100.0%  91.4%    AFPAPVQQVEPDVGCLELFHGPTLAFKDFGGRFMAQMLTHISGDKPVTILTATSGDTGAAVAHAFYGLKNVRVVILYPKG
 7 Phytobacter     100.0%  90.2%    AFPAPVQPVESDVGCLELFHGPTLAFKDFGGRFMAQMLTTISGDKPVTILTATSGDTGAAVAHAFYGLKNVRVVILYPNG
 8 Kosakonia       100.0%  89.5%    AFPAPVKPVESDVGCLELFHGPTLAFKDFGGRFMAQMLSAISGDKPVTILTATSGDTGAAVAHAFYGMKNVRVVILYPQG
 9 Lelliottia      100.0%  90.7%    TFPAPVKSVEPDIGCLELFHGPTLAFKDFGGRFMAQMLTHISGDKPVTILTATSGDTGAAVAHAFYGLKNVRVVILYPKG
   consensus/100%                   sFPAPVt.Vt.DlGCLELFHGPTLAFKDFGGRFMAQMLotIuGD+PVTILTATSGDTGAAVAHAFYGh.NVpVVILYPpG
   consensus/90%                    sFPAPVt.Vt.DlGCLELFHGPTLAFKDFGGRFMAQMLotIuGD+PVTILTATSGDTGAAVAHAFYGh.NVpVVILYPpG
   consensus/80%                    sFPAPVt.VpsDlGCLELFHGPTLAFKDFGGRFMAQMLTpIuGDKPVTILTATSGDTGAAVAHAFYGL.NV+VVILYPpG
   consensus/70%                    AFPAPVtpVEsDVGCLELFHGPTLAFKDFGGRFMAQMLTpISGDKPVTILTATSGDTGAAVAHAFYGL.NV+VVILYP+G

                      cov    pid 161         .         .         .         2         .         .         .         . 240
 1 Salmonella      100.0% 100.0%    KISPLQEKLFCTLGGNIETVAIDGDFDACQALVKQAFDDEELKTALGLNSANSINISRLLAQICYYFEAVAQLPQGARNQ
 2 Escherichia     100.0%  93.5%    KISPLQEKLFCTLGGNIETVAIDGDFDACQALVKQAFDDEELKVALGLNSANSINISRLLAQICYYFEAVAQLPQETRNQ
 3 Citrobacter     100.0%  93.9%    KISPLQEKLFCTLGGNIETVAIDGDFDACQALVKQAFDDHELKDALGLNSANSINISRLLAQICYYFEAVAQLPQEARNQ
 4 Klebsiella       99.5%  90.1%    KISPLQEKLFCTLGGNIETVAIDGDFDACQALVKQAFDDEELKATLGLNSANSINISRLLAQICYYFEAAAQLPQEARNQ
 5 Cronobacter     100.0%  86.7%    KISPLQEKLFCTLGGNIETVAIDGDFDACQALVKQAFDDEELKTKLGLNSANSINISRLLAQICYYFEAVAQLPQEARNQ
 6 Enterobacter    100.0%  91.4%    KISPLQEKLFCTLGGNIETVAVDGDFDACQALVKQAFDDEELKAALGLNSANSINISRLLAQICYYFEAVAQLPQEARNQ
 7 Phytobacter     100.0%  90.2%    KISPLQEKLFCTLGGNIETVAIGADFDACQALVKQAFDDEELKVALGLNSANSINISRLLAQICYYFEAVAQLPQEARNQ
 8 Kosakonia       100.0%  89.5%    KISPLQEKLFCTLGGNIETVAIDADFDACQALVKQAFDDEELKTALGLNSANSINISRLLAQICYYFEAVAQLPQEARNQ
 9 Lelliottia      100.0%  90.7%    KISPLQEKLFCTLGGNIETVAIDGDFDACQALVKQAFDDEELKAALGLNSANSINISRLLAQICYYFEAVAQLPQEARNQ
   consensus/100%                   KISPLQEKLFCTLGGNIETVAlsuDFDACQALVKQAFDDcELKstLGLNSANSINISRLLAQICYYFEAsAQLPQtsRNQ
   consensus/90%                    KISPLQEKLFCTLGGNIETVAlsuDFDACQALVKQAFDDcELKstLGLNSANSINISRLLAQICYYFEAsAQLPQtsRNQ
   consensus/80%                    KISPLQEKLFCTLGGNIETVAIDuDFDACQALVKQAFDDEELKssLGLNSANSINISRLLAQICYYFEAVAQLPQEARNQ
   consensus/70%                    KISPLQEKLFCTLGGNIETVAIDGDFDACQALVKQAFDDEELKsALGLNSANSINISRLLAQICYYFEAVAQLPQEARNQ

                      cov    pid 241         :         .         .         .         .         3         .         . 320
 1 Salmonella      100.0% 100.0%    LVISVPSGNFGDLTAGLLAKSLGLPVKRFIAATNVNDTVPRFLHDGKWAPKATQATLSNAMDVSQPNNWPRVEELFRRKI
 2 Escherichia     100.0%  93.5%    LVVSVPSGNFGDLTAGLLAKSLGLPVKRFIAATNVNDTVPRFLHDGQWSPKATQATLSNAMDVSQPNNWPRVEELFRRKI
 3 Citrobacter     100.0%  93.9%    LVISVPSGNFGDLTAGLLAKSLGLPVKRFIAATNVNDTVPRYLQEGKWTPKATQATLSNAMDVSQPNNWPRVEELFRRKI
 4 Klebsiella       99.5%  90.1%    LVISVPSGNFGDLTAGLLAKSLGLPIKRFIAATNANDTVPRYLQGGEWAPKATQATLSNAMDVSQPNNWPRVEELFRRKI
 5 Cronobacter     100.0%  86.7%    LVVSVPSGNFGDLTAGLLAKSAGLPVKRFIAATNANDTVPRFLQHGEWLPNATVATLSNAMDVSQPNNWPRVEELFRRKI
 6 Enterobacter    100.0%  91.4%    LVVSVPSGNFGDLTAGLLAKSLGLPVKRFIAATNANDTVPRFLKDGKWAPNATQATLSNAMDVSQPNNWPRVEELFRRKV
 7 Phytobacter     100.0%  90.2%    LVVSVPSGNFGDLTAGLLAKSLGLPIKRFIAATNANDTVPRFLQDGEWKPKATQATLSNAMDVSQPNNWPRVEELFRRKI
 8 Kosakonia       100.0%  89.5%    LVVSVPSGNFGDLTAGLLAKSLGLPIKRFIAATNANDTVPRFLKEGQWQPKATQATLSNAMDVSQPNNWPRVEELFRRKI
 9 Lelliottia      100.0%  90.7%    LVISVPSGNFGDLTAGLLAKSLGLPVKRFIAATNANDTVPRFLKDGTWAPNATKATLSNAMDVSQPNNWPRVEELFRRKI
   consensus/100%                   LVlSVPSGNFGDLTAGLLAKShGLPlKRFIAATNsNDTVPRaLptGpW.PpAT.ATLSNAMDVSQPNNWPRVEELFRRKl
   consensus/90%                    LVlSVPSGNFGDLTAGLLAKShGLPlKRFIAATNsNDTVPRaLptGpW.PpAT.ATLSNAMDVSQPNNWPRVEELFRRKl
   consensus/80%                    LVlSVPSGNFGDLTAGLLAKSLGLPlKRFIAATNsNDTVPRaLpcGpWtPpATpATLSNAMDVSQPNNWPRVEELFRRKI
   consensus/70%                    LVlSVPSGNFGDLTAGLLAKSLGLPlKRFIAATNsNDTVPRFLp-GpWtPpATQATLSNAMDVSQPNNWPRVEELFRRKI
```

10. ThrC (part 2)

```
                       cov     pid 321         .         .         :         .         .         .         .         4 400
 1 Salmonella      100.0% 100.0%     WRLTELGYAAVDDTTTQQTMRELKAKGYISEPHAAVAYRALRDQLNPGEYGLFLGTAHPAKFKESVESILGETLALPEAL
 2 Escherichia     100.0%  93.5%     WQLKELGYAAVDDETTQQTMRELKELGYTSEPHAAVAYRALRDQLNPGEYGLFLGTAHPAKFKESVEAILGETLDLPKEL
 3 Citrobacter     100.0%  93.9%     WRLNELGYAAVDDATTQETMRELQAKGYLSEPHAAVAYRALRDQLNPGEYGLFLGTAHPAKFKESVEAILGETLELPKAL
 4 Klebsiella       99.5%  90.1%     WRLSELGYAAVDDETTKAAMRELKAIGYISEPHAAIAWRALRDQLQPGEYGLFLGTAHPAKFKESVEEILQETLPLPKEL
 5 Cronobacter     100.0%  86.7%     WRLNELGVATIDDEITKATMRELRELGYLSEPHAAVAYRALRDQLRPGEYGLFLGTAHPAKFKESVEEVLGETLELPAEL
 6 Enterobacter    100.0%  91.4%     WRLGDLGYAAVTDETTKSTMRELKAVGYTSEPHAAIAYRALRDQLNPGEYGLFLGTAHPAKFKESVEEILGETLPLPKEL
 7 Phytobacter     100.0%  90.2%     WRLNELGYAAVDDETTKETMRELKGKGYLSEPHAAIAYRALRDQLHPGEYGLFLGTAHPAKFKESVEEILGETLPLPKEL
 8 Kosakonia       100.0%  89.5%     WRLTELGYAAVDDETTKATIRELKGKGYISEPHAAIAYRALRDQLQPGEYGLFLGTAHPAKFKESVEAILGETLPLPKEL
 9 Lelliottia      100.0%  90.7%     WRLGDLGYAAVTDETTKATMRELKAVGYTSEPHAAIAYRALRDQLNPGEYGLFLGTAHPAKFKESVDEILGESLPLPKEL
   consensus/100%                    WpLt-LGhAslsDthTptshRELpthGYhSEPHAAlAaRALRDQLpPGEYGLFLGTAHPAKFKESV-tlLtEoL.LPttL
   consensus/90%                     WpLt-LGhAslsDthTptshRELpthGYhSEPHAAlAaRALRDQLpPGEYGLFLGTAHPAKFKESV-tlLtEoL.LPttL
   consensus/80%                     WRLs-LGYAAVsDpTTptTMREL+thGYhSEPHAAlAYRALRDQLpPGEYGLFLGTAHPAKFKESVEtILGETL.LPctL
   consensus/70%                     WRLsELGYAAVDDETTptTMRELKuhGYhSEPHAAlAYRALRDQLpPGEYGLFLGTAHPAKFKESVEtILGETLsLPKEL

                       cov     pid 401         .         .        ] 429
 1 Salmonella      100.0% 100.0%     AERADLPLLSHHLPADFAALRKLMMTRQ-
 2 Escherichia     100.0%  93.5%     AERADLPLLSHNLPADFAALRKLMMNHQ-
 3 Citrobacter     100.0%  93.9%     AERADLPLLSHELPADFAALRKLMMSRR-
 4 Klebsiella       99.5%  90.1%     ADRADLPLLSHNLPADFAALRKLMMG---
 5 Cronobacter     100.0%  86.7%     AERVDLPLLSHSLPADFAALREFMMSKAP
 6 Enterobacter    100.0%  91.4%     ADRADLPLLSHELPADFAALRKLMMTRA-
 7 Phytobacter     100.0%  90.2%     AERADLELLSHHLPADFAELRKLMMSKA-
 8 Kosakonia       100.0%  89.5%     ADRADLPLLSHQLPADFSALRQLMMSKA-
 9 Lelliottia      100.0%  90.7%     AERADLPLLSHELPADFAALRKLMMTRG-
   consensus/100%                    A-RsDL.LLSHpLPADFutLRphMMs...
   consensus/90%                     A-RsDL.LLSHpLPADFutLRphMMs...
   consensus/80%                     A-RADLPLLSHpLPADFAALRcLMMs+t.
   consensus/70%                     A-RADLPLLSHpLPADFAALRKLMMo+t.
```

```
#  Percent Identity  Matrix - created by Clustal2.1
#
#
```

1: Salmonella	100.00	93.46	93.93	90.14	86.92	91.36	90.19	89.49	90.65
2: Escherichia	93.46	100.00	91.82	90.61	87.15	91.12	90.42	89.72	89.95
3: Citrobacter	93.93	91.82	100.00	89.20	87.15	89.72	89.95	89.02	89.02
4: Klebsiella	90.14	90.61	89.20	100.00	87.09	90.14	90.14	89.67	89.67
5: Cronobacter	86.92	87.15	87.15	87.09	100.00	86.68	88.08	86.68	86.92
6: Enterobacter	91.36	91.12	89.72	90.14	86.68	100.00	90.19	90.19	94.39
7: Phytobacter	90.19	90.42	89.95	90.14	88.08	90.19	100.00	91.59	89.49
8: Kosakonia	89.49	89.72	89.02	89.67	86.68	90.19	91.59	100.00	88.55
9: Lelliottia	90.65	89.95	89.02	89.67	86.92	94.39	89.49	88.55	100.00
